# Viral integration drives multifocal HCC during the occult HBV infection

**DOI:** 10.1186/s13046-019-1273-1

**Published:** 2019-06-14

**Authors:** Xiao-Ping Chen, Xin Long, Wen-long Jia, Han-Jie Wu, Jing Zhao, Hui-Fang Liang, Arian Laurence, Jun Zhu, Dong Dong, Yan Chen, Long Lin, Yu-Dong Xia, Wei-Yang Li, Gui-Bo Li, Zhi-Kun Zhao, Kui Wu, Yong Hou, Jing-Jing Yu, Wei Xiao, Guo-Ping Wang, Peng-Cheng Zhu, Wei Chen, Ming-Zhou Bai, Yi-Xing Jian, Karsten Kristiansen, Qian Chen

**Affiliations:** 1 The Hepatic Surgery Centre at Tongji Hospital, Tongji Medical College, HUST; Hubei Province for the Clinical Medicine Research Center of Hepatic Surgery, Key Laboratory of Organ Transplantation, Ministry of Education and Ministry of Public Health, Wuhan, 430030 China; 2 Department of Computer Science, City University of Hong Kong, Hong Kong, People’s Republic of China; 3 School of Biomedical Engineering, University of Technology Sydney, Sydney, NSW2007 Australia; 4 Department of Biology, Laboratory of Genomics and Molecular Biomedicine, University of Copenhagen, Universitesparken 13, 2100 Copenhagen, Denmark; 5 The Newcastle upon Tyne Hospitals NHS Foundation Trust at Freeman Hospital, Newcastle, UK; 6 Department of Genetics and Genomic Sciences, Icahn Institute of Genomics and Multiscale Biology, Icahn School of Medicine at Mount Sinai, 1425 Madison Avenue, New York, NY USA; 7 Laboratory of Molecular Ecology and Evolution, Institute of Estuarine and Coastal Research, East China Normal University, Shanghai, China; 8 School of Bioscience and Bioengineering at South China University of Technology, Guangzhou, China; 9 School of Biological Science and Medical Engineering at Southeast University, Nanjing, China; 10 The Department of Pathology at Tongji Hospital, Tongji Medical College, HUST, Wuhan, 430030 China; 11 The Division of Gastroenterology, Department of Internal Medicine at Tongji Hospital, Tongji Medical College, Huazhong University of Science and Technology (HUST), Wuhan, 430030 China

**Keywords:** Hepatitis B, Hepatocellular carcinoma, Single-cell sequencing, Viral integration, Virome capture sequencing, Whole genome sequencing

## Abstract

**Background & Aims:**

Although the prognosis of patients with occult hepatitis B virus (HBV) infection (OBI) is usually benign, a small portion may undergo cirrhosis and subsequently hepatocellular carcinoma (HCC). We studied the mechanism of life-long Integration of virus DNA into OBI host’s genome, of which may induce hepatocyte transformation.

**Methods:**

We applied HBV capture sequencing on single cells from an OBI patient who, developed multiple HCC tumors and underwent liver resection in May 2013 at Tongji Hospital in China. Despite with the undetectable virus DNA in serum, we determined the pattern of viral integration in tumor cells and adjacent non-tumor cells and obtained the details of the viral arrangement in host genome, and furthermore the HBV integrated region in cancer genome.

**Results:**

HBV captured sequencing of tissues and individual cells revealed that samples from multiple tumors shared two viral integration sites that could affect three host genes, including *CSMD2* on chr1 and *MED30*/*EXT1* on chr8. Whole genome sequencing further indicated one hybrid chromosome formed by HBV integrations between chr1 and chr8 that was shared by multiple tumors. Additional 50 poorly differentiated liver tumors and the paired adjacent non-tumors were evaluated and functional studies suggested up-regulated *EXT1* expression promoted HCC growth. We further observed that the most somatic mutations within the tumor cell genome were common among the multiple tumors, suggesting that HBV associated, multifocal HCC is monoclonal in origin.

**Conclusion:**

Through analyzing the HBV integration sites in multifocal HCC, our data suggested that the tumor cells were monoclonal in origin and formed in the absence of active viral replication, whereas the affected host genes may subsequently contribute to carcinogenesis.

**Electronic supplementary material:**

The online version of this article (10.1186/s13046-019-1273-1) contains supplementary material, which is available to authorized users.

## Background

Hepatitis B virus is a common cause of chronic liver infection throughout the world. Chronic HBV infection often leads to the development of hepatocellular carcinoma (HCC) [1]. The overall prognosis is poor as many patients present with multifocal disease. Furthermore, radical liver resection is typically ineffective as new tumors commonly appear in the remnant liver. This highlights a longstanding question in HCC carcinogenesis: when patients present with multifocal liver disease, are the individual tumors of a monoclonal or multiclonal origin [2, 3]? Many clinical studies favor a monoclonal origin theory where multiple small tumors in the liver are derived from a primary HCC tumor through invading the inflow or outflow of the hepatic vascular tree with subsequent intrahepatic spread [4, 5]. Conversely a diffuse or multifocal theory of HCC has been proposed, in which multiple HCC tumors are derived independently from de novo mutations in a liver subject to a genetic field change caused by chronic viral infection [6, 7].

Viral replication and viral DNA integration into host chromosomes are two distinctive pathogenic mechanisms of HBV-associated HCC [8–11]. In order to study malignant transformation driven solely through viral integration, the tumor tissues should be obtained from patients who have entirely cleared HBV surface antigen (HBsAg) or HBV e antigen (HBeAg). Occult hepatitis B virus infection (OBI) is characterized by the persistence of HBV-DNA in the liver tissue of individuals who successfully clear the virus from the blood as suggested by the loss of HBV antigens and the appearance of anti-HBV antibodies [12]. While in the OBI state, the replication-competent viruses are strongly suppressed in their activities by the host’s defense mechanisms. Therefore, the prognosis of OBI is usually benign characterized by the normal ALT levels and frequent loss of HBV-DNA in peripheral, commonly showing the minimal risk of cirrhosis, decompensation, and HCC, as well as the improved survival [12, 13]. Yet a small proportion of these patients do develop HCC despite being free of actively replicating virus [14].

Here, we studied one such OBI patient whom we believe this scenario provides a valuable model to exclusively examine effects of viral integration on liver carcinogenesis and tumor progression.

## Methods

### Study subject

The study was approved by the Institutional Review Board of Tongji Hospital, Tongji Medical College of HUST, in Hubei province, China. More detailed description of the clinicopathologic features of the patient and specimens can be found in the Additional file 1.

### Single cell collection and amplification

The procedure can be found in the Additional file 1.

### The HBV integration breakpoint detection procedure

We applied FuseSV (in-house software) to gather reads mapped to individual HBV genome and detect HBV integrations (Additional file 1: Figure S1).

### Cell lines and cell culture

These were described in detail in the Additional file 1 and our previous study [15].

## Results

### Characterization of an OBI patient with multiple tumors in liver with vascular metastatic invasion

**Fig. 1:**
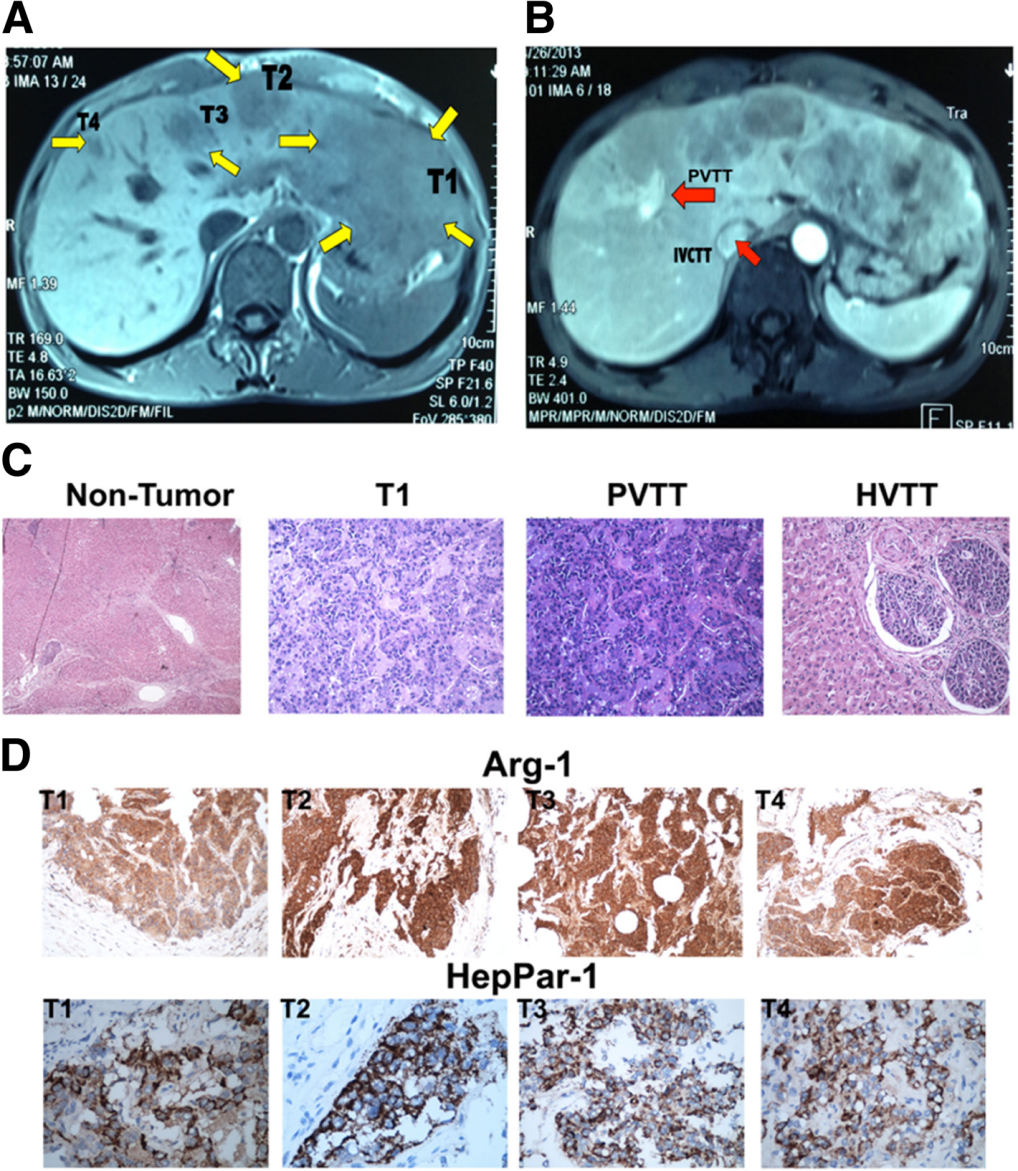
Detection of multiple hepatocellular carcinomas (HCC) in the patient. Magnetic resonance imaging (MRI) reveals a 15 cm × 10 cm larger lesion in the left hepatic lobe and multiple smaller lesions in the right hepatic lobe, all less than 3 cm in diameter (**a**). Yellow arrows indicate multiple tumor foci of various sizes. MRI with contrast enhancement reveals tumor thrombosis involving the inferior vena cava (IVCTT), and the right portal vein branch (PVTT), indicated by the red arrows, respectively in **b**, suggesting intrahepatic and extrahepatic vascular spread of HCC. **c** shows representative photomicrographs of the multifocal invasive HCC that was profiled in this study. Of these foci, hematoxylin and eosin staining is shown for the large tumor, T1, its adjacent cirrhosis tissue (Non-Tumor), the tumor invading portal vein (PVTT), and peripheral hepatic vein (HVTT). Histologically, the HCC are of poor-to-moderate [16–18] differentiation with trabecular and solid patterns further evaluated by immunohistochemical (IHC) staining with Arginase-1 (Arg-1; Upper panel in **d**) and Hepatocyte Paraffin-1 (HepPar-1; Lower panel in **d**)

The OBI patient in this study was a 47-year-old Chinese male (Additional file 1: Table S1). Liver magnetic resonance imaging (MRI) revealed a main lesion in the left hepatic lobe, three smaller lesions in the right hepatic lobe, tumor thrombi in right portal vein branch (PVTT) and inferior vena cava (IVCTT/HVTT). These findings indicated both intrahepatic and extrahepatic vascular spread of HCC (Fig. 1).

### Identifying all samples with the integrated HBV-B2 DNA by virome capture sequencing (VCS)

We examined ten surgical specimens: T1 obtained from the largest tumor site and three smaller liver tumors found in the right hepatic lobe (T2-T4), biopsies from the adjacent non-tumor liver tissue (N1-N4) as germline controls, and embolic metastatic deposits of HCC in the hepatic and portal veins (HVTT and PVTT). VCS data identified the presence of the HBV_B2 subtype in all six tumor samples with partial HBV genome covered (T1-T4, HVTT and PVTT, mean depth = 79.16X, range: 47X-93X; mean coverage = 79.72%, range: 79–82%; Additional file 1: Table S2, Additional file 1: Figure S2 and Additional file 1: Figure S3). We identified HBV_B2 signals in all the four control samples (N1-N4, mean depth = 16X, range: 9X-33X). Furthermore, 61 single nucleotide variants (SNVs) within the HBV_B2 genome were identified being shared by the six tumor samples (Additional file 1: Table S3). We further noted all six tumor samples were absent of the viral coding region for the HBV X gene (Additional file 1: Figure S3), highlighting an identical structure and alterations of HBV genome shared by all lesions.

### Seeking HBV integrations and viral rearrangements

**Fig. 2:**
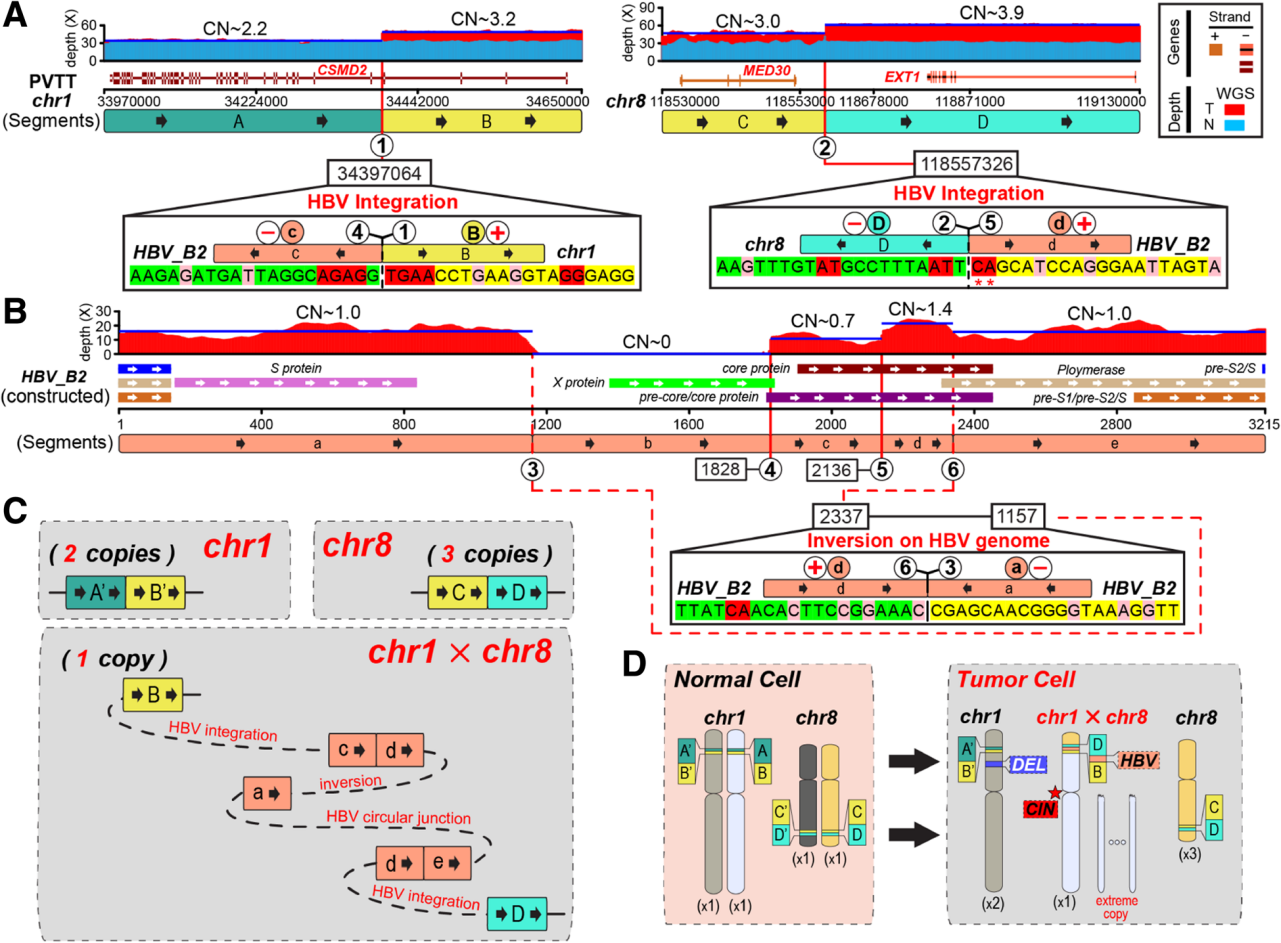
Local haplotype of HBV-integrated genomic region in sample PVTT. **a** Human genomic region of chr1 and chr8 flanking HBV integrations are divided into segments (A~D) by viral insertions. Breakpoints are noted by circled numbers. Sequencing depth spectrum (red for tumor, light-blue for control) are displayed with copy numbers of segments. Dark-blue lines denote average depth of segments. For each segment junction, micro-homologies in bilateral twenty base-pairs (pink for one-bp size; red for larger). Mutations on HBV genome were denoted by red asterisks. Connection orientation of segments are noted by circled plus or minus symbol in red. **b** Constructed *HBV_B2* genome is segmented (a~e) by breakpoints with circled numbers. **c** Resolved alleles of local haplotype are indicated as coloured segments connected string with copy times. The “circular junction” means HBV genome circular loop site. **d** Hybrid chromosome and copy number shift of chr1 and chr8 from normal cell to tumor cell. Parental homologous chromosomes are in different colors

To explore the underlying mechanism by which the HBV X gene is truncated, we validated two HBV integration sites in host genome (chr1:34397064 and chr8:118557326, Fig. 2a**,** Additional file 1: Table S2). One is located in the intronic region of gene *CSMD2* on chr1 and the other is located in the intergenic region of genes *MED30*/*EXT1* on chr8, respectively. These two integrations repeatedly occurred in all six tumors, suggesting a monoclonal origin. We further discovered micro-homologous bases from the junction sequences (Fig. 2a). In particular, at the chr8 integration site, the adjacent two downstream bases became micro-homologous due to the SNVs on HBV_B2 genome (Fig. 2a**,** Additional file 1: Figure S3).

We found the two HBV integration sites resided on different chromosomes. Only one viral breakpoint of HBV integration met the 3-prime end (HBV_B2:1828) of the truncated region on the constructed HBV genome (Additional file 1: Figure S3). We found one inverse rearrangement on HBV genome whose one junction position was just the 5-prime end (HBV_B2:1157) of the truncated viral region (Fig. 2b**,** Additional file 1: Figure S3). In all six tumors, the HBV genomic region, from the viral breakpoint (HBV_B2:2136) in chr8 to the viral breakpoint (HBV_B2:2337) with the inverse rearrangement, displayed a greater sequencing depth compared with other parts of the viral genome (Fig. 2b**,** Additional file 1: Figure S3). This suggested that the host integrations and the viral inversion are phased together, resulting in a bridge between chr1 and chr8 by HBV genome.

### Single cell VCS data identifies heterogeneity within tumors

We next combined single cell sequencing with a virus-capture approach to detect the presence of fragments of viral DNA in individual cells. 264 cells were selected from six tumors for DNA extraction and single cell VCS analysis (Additional file 1: Table S2). We successfully identified 229 cells with detectable HBV DNA (86.74%). We detected the presence of the HBV_B2 genome in 219 out of 229 viral signal positive cells (95.63%, mean depth = 117.61X, range, 1X-7443X, mean coverage = 67.62%, range, 27–79%) (Additional file 1: Table S4). Consistent with the VCS data, we recalled 61 SNVs within the HBV_B2 genome with a maximum coverage of 79.38% in tumor tissues.

Within this HBV_B2 population, we identified 51.60% (113/219) of tumor cells showing viral integrations on chr1, 58.90% (129/219) on chr8, and 59.36% (130/219) with the inversion of the HBV_B2 genome, respectively. We identified HBV_B2 integrations that had uneven distribution among cells from different tissues suggesting tumor heterogeneity (Additional file 1: Table S4). Despite this, it was possible to identify “major events” that were shared by all tumor tissues (Additional file 1: Table S5). We further identified 11 “minor events” characterized by genetic changes in at least two cells (Additional file 1: Table S5). Among them, 10 minor viral integrations were located within five kilobases from the major events. All minor events showed prevalent enrichment of the large-size micro-homologies (Additional file 1: Table S5), a phenomenon has been discovered in chimeric DNA rearrangements during single cell Multiple Displacement Amplification (MDA) [19, 20].

### Revealing a common ‘local haplotype’ at HBV-integrated loci within tumors by whole genome sequencing (WGS)

Next we performed WGS on all ten tissues. Consistent with the VCS data, WGS data confirmed the presence of HBV_B2 viral DNA in all six tumor samples and again identified HBV integrations at chr1 and chr8 (Additional file 1: Table S2).

**Fig. 3:**
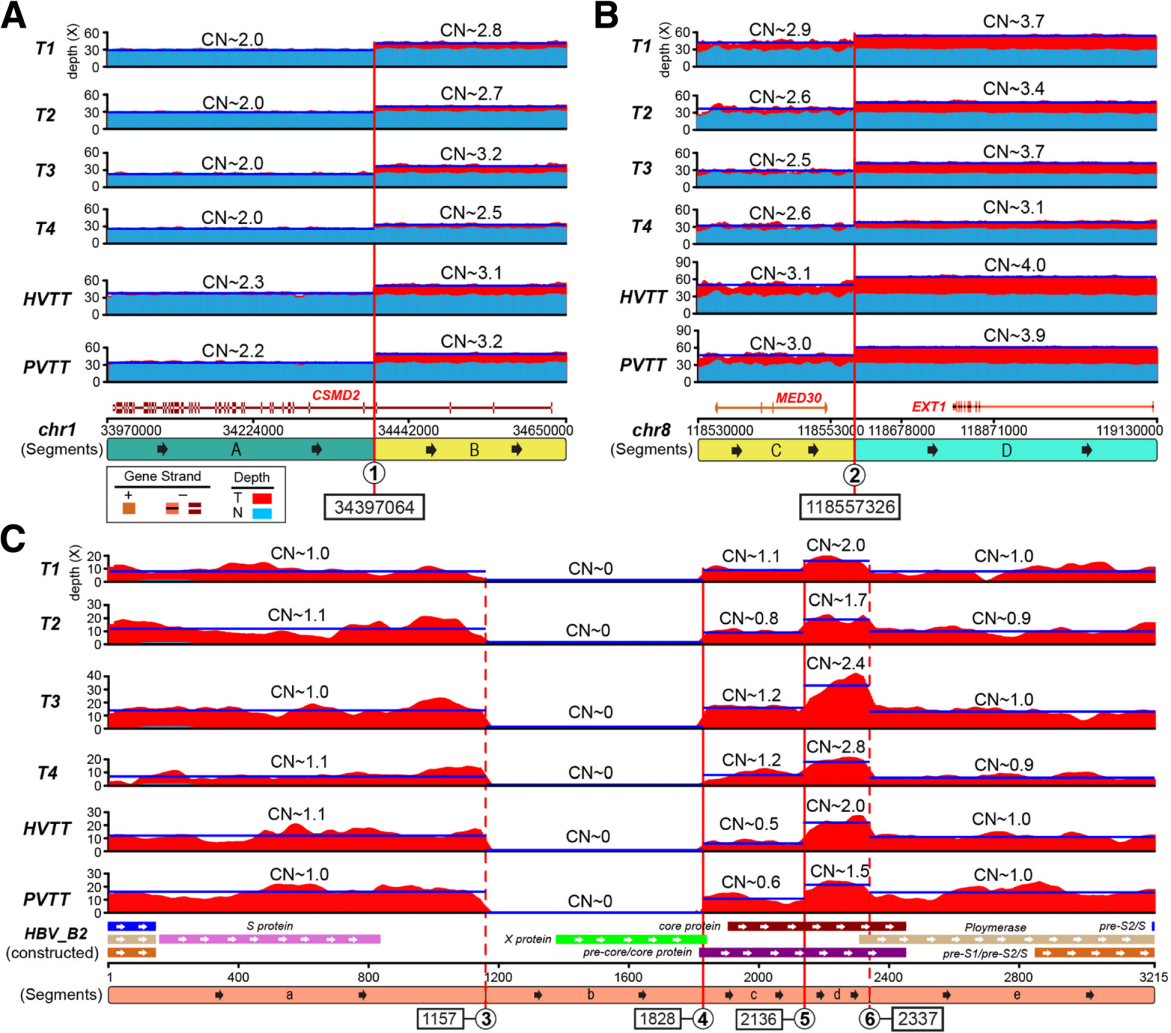
Copy number comparison of HBV-integrated genomic region among six tumors. Segmentations of HBV-integrated human genomic region and HBV genome in six tumor tissue samples were shown with copy number counts as Fig. 2. **a** chr1; **b**, chr8; **c**, HBV_B2 genome

For copy number variation (CNV) analysis, we next selected sample N1 as the global control, which showed the minimum depth of HBV_B2 genome (Additional file 1: Table S2). In all tumor samples, we found that human genomic segments flanked the HBV_B2 integrated sites (chr1:34397064 and chr8:118557326) with a consistent increase in copy number (CN, Additional file 1: Figure S5). In the PVTT sample there were two copies of adjacent genomic region upstream of the chr1-integrated position, three for the downstream genome segment, and three and four copies for those regions on chr8, respectively. Moreover, six tumor samples shared this CN pattern near the HBV_B2 integrations with a high Pearson correlation coefficient (mean = 0.962, range: 0.875–0.997, Fig. 3a&b). Although the slight CN difference could be caused by tumor cell purity (Additional file 1: Table S2), these six tumor samples showed relatively similar CN distribution along the HBV_B2 genome (Fig. 3c), suggesting a monoclonal origin.

To resolve the “local haplotype” of the HBV_B2 integration sites, we next analyzed the breakage of two integrations on cancer genome and the fusion of chr 1 and 8 linked by a viral DNA bridge (Fig. 2). The shaped “bridge” contained five viral segments, lacked “b” segment but had a duplicated “d” HBV segment. Via this HBV “bridge”, the tail (3-prime) of 8q was concatenated with chr 1p in the reverse orientation, replacing the head (5-prime) of 1p. This process generated one hybrid chromosome, whose short arm consisted of a small region from 8q (the tail) and a large region from 1p (Fig. 2c&d). Moreover, heterozygosity analysis showed most of 1p and the whole of 1q harbored minor frequency alleles. While the head (5-prime) of 1p, the region replaced by tail of 8q and the whole of chr8 indicated by loss of heterozygosity (LOH), were potentially induced by HBV_B2 integrations and dis-regulation of chromosome segregation during mitosis. Note that one frequent LOH (chr1:53468857 to chr1:67082731) existed on 1p among all tumor samples, (Additional file 1: Figure S5). Together we hypothesize a model that the integration of HBV_B2 results in formation of a hybrid chromosome between one homologous chromosome pair of chr1 and chr8. The remaining homologous chr1 and chr8 lack of viral integration subsequently became duplicated and triplicated to form two and three copies within the tumor cell. Based on the CN count, the large deletion on 1p should phase with the non-HBV-integrated chr1 (Fig. 2d). This was compounded by multiple duplications of the region of 1q from the hybrid chromosome in tumor cells (Fig. 2d), suggesting the potential chromosome instability (CIN) at the centromere of the hybrid chromosome, as there was a step change in CN at 1q21.1 and 1q21.3 (Additional file 1: Figure S5).

### Common genome-wide somatic alterations reveal a monoclonal origin of multifocal HCC

**Fig. 4:**
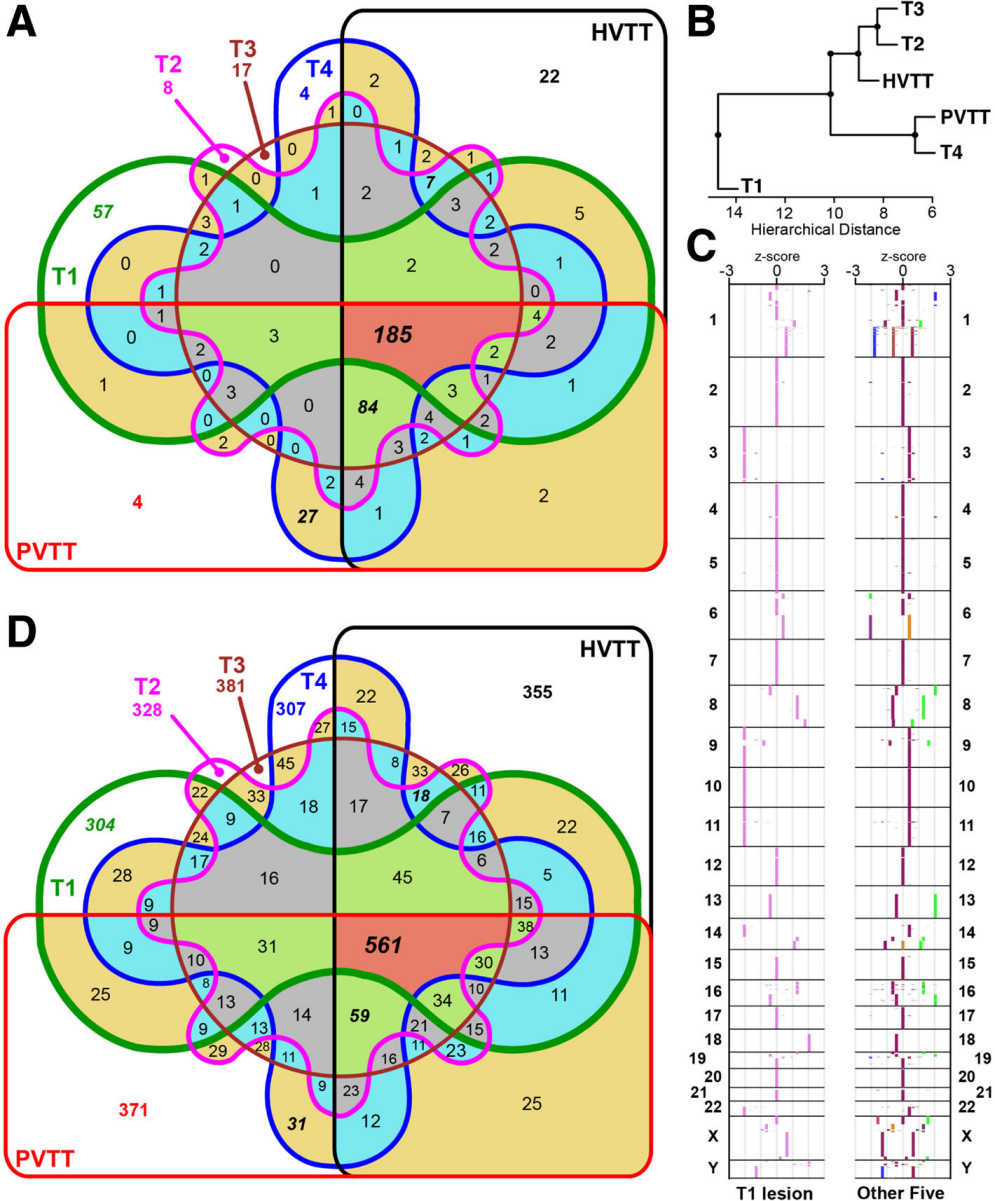
Comparison of genome-wide somatic alterations among six tumors. **a** Venn diagram comparing the somatic SNVs in genetic region among the six tumors. **b** Hierarchical clustering based on genetic somatic SNVs. **c** Distribution of copy number z-score along the genome of T1 and the other five tumor tissues. **d** Venn diagram comparing the somatic SVs among the six tumors

To further understand the origin of tumor cells, we conducted mutation analysis by calling genome-wide somatic mutations from WGS data. We identified 35,165 somatic SNVs and among them 495 SNVs located in genetic regions. There were 12,110 SNVs (34.44%) shared by six tumor tissues (unpublished data & Additional file 1: Figure S4A). However, each individual tumor harboured abundant unique SNVs, suggesting their heterogeneity of development. T1 in comparison to other lesions had the largest tumor size and correspondingly, contained the most unique SNVs (4042). The T2-T4, PVTT and HVTT samples shared 4341 somatic SNVs, revealing their close relationship in evolution. Moreover, the T4 and PVTT samples, which were located close to each other, shared more unique SNVs than any other pairwise tumor samples. Through hierarchical clustering, the six tumor lesions were divided into three clusters (T1, T4/PVTT, and T2/T3/HVTT; Additional file 1: Figure S4B), of which were further confirmed by comparing their genetic SNVs (Fig. 4a&b).

We detected that all six tumors gained global copy number that on average covered 91.41% of the genomic region (Additional file 1: Figure S5A), generating a high-level ploidy within tumor cells (mean = 7.73, Additional file 1: Table S2). Furthermore, the copy number patterns were consistent among all lesions (Additional file 1: Figure S5). Overall arm level duplications were common along the cancer genome, including among chromosomes 1q, 2, 4p, 5, 6, 7, 12, 15–22, X and Y, of which chr 1q had the highest copy number. For each CNV region along the cancer genome, we calculated the mean and SD of the copy number of the six tumor samples. Subsequently, a z-score could be calculated for individual sample and its every given CNV region, reflecting how much it has shifted from the average. With the exception of T1, other five tumor samples had low z-scores (− 1 to 1) of copy number variation throughout the majority of their genomic region (mean ratio = 97.97%, Fig. 4c). In contrast, only 35.35% of the genomic region of T1 demonstrated a low copy number variation, with large z-scores (− 3 to − 1 or 1 to 3) measured at chromosomes 3, 9, 10, 11, 14, 18, and 22, respectively (Fig. 4c). We next identified 3711 somatic structure variations (SVs) and among them, 561 cases shared by all six lesions (Fig. 4d & unpublished data). While comparing the number of unique somatic SVs versus SNVs, we found individual tumor samples contained higher proportion of unique somatic SVs (mean = 9.19%, range: 8.19–10.00%) than that of somatic SNVs (mean = 3.76%, range: 1.40–11.49%). Among all samples, T1 had the greatest number of unique somatic SNVs but comparable number of unique somatic SVs.

### HBV integration leads to altered expression of *CSMD2* and *EXT1*

**Fig. 5:**
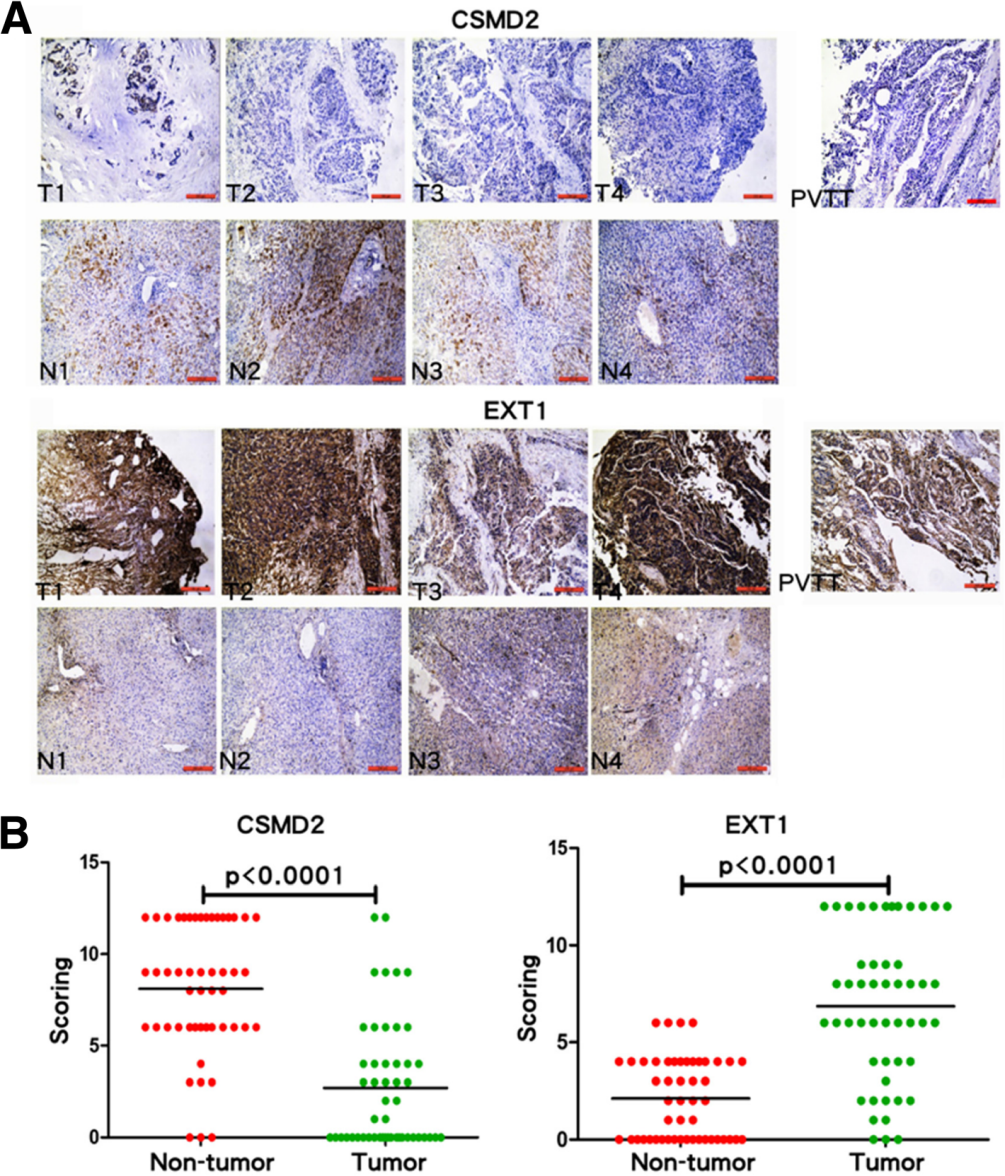
Detection of CSMD2 and EXT1 expression in liver by IHC. Upper and lower panels show the analysis of CSMD2 and EXT1 expression by IHC staining in tumors, non-tumors, and PVTT from the same patient. CSMD2 is down-regulated and EXT1 is up-regulated in tumors and PVTT compared to non-tumor tissues (**a**). Additional 50 poorly differentiated liver tumors and the paired adjacent non-tumor tissues were acquired to confirm the findings (**b**). Wilcoxon signed rank test is used to determine the difference between tumors and non-tumors in CSMD2 expression (left panel) and EXT1 expression (right panel). CSDM2 scoring for adjacent non-tumor group as follows: *n* = 50, Median = 9, 25% Percentile = 6, 75% Percentile = 12, Mean = 8.1, SD = 3.5, SEM = 0.50; versus for tumor group as follows: n = 50, Median = 1, 25% Percentile = 0, 75% Percentile = 4, Mean = 2.7, SD = 3.4, SEM = 0.49. EXT1 scoring for non-tumor group as follows: n = 50, Median = 2, 25% Percentile = 0, 75% Percentile = 4, Mean = 2.12, SD = 2.06, SEM = 0.29; versus for tumor group as follows: n = 50, Median = 7, 25% Percentile = 4, 75% Percentile = 9.75, Mea*n* = 6.86, SD = 3.86, SEM = 0.55

We further studied the two integration sites that were common in all six tumors: the intronic region of *CSMD2* on chr1 and the intergenic region of *MED30-EXT1* on chr8. The *CSMD2* gene only retained a 3-prime remnant of its genetic locus, and *MED30* gene was entirely deleted with the lost part of chr8. In contrast, *EXT1* gene was retained with the integrated HBV_B2 segment located upstream. Immunohistochemistry (IHC) analysis of tumor biopsy samples confirmed a loss of *CSMD2* and enhanced *EXT1* expression in tumor and PVTT cells (Fig. 5a), whereas *MED30* expression was unchanged (data not shown). To evaluate the significance of this finding, we conducted IHC staining on additional 50 poorly differentiated liver tumors and the paired adjacent non-tumors. The data revealed that the expression of *CSMD2* and *EXT1* were significantly altered between non-tumors and tumors in a large number of patients with HCC (Fig. 5b, Wilcoxon signed rank test, *P* < 0.0001).

### EXT1 promotes HCC cell growth in vitro *and* in vivo

**Fig. 6:**
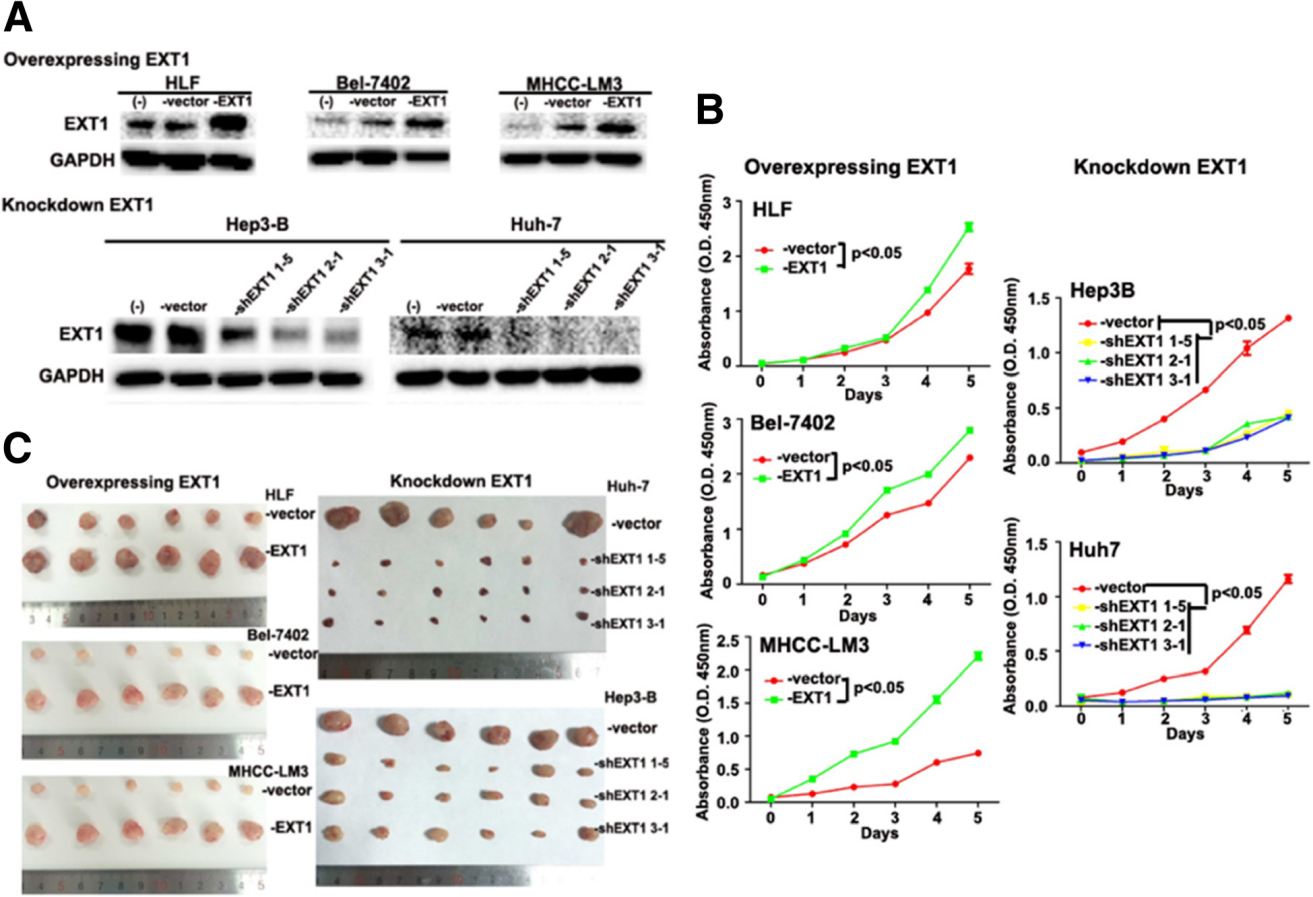
EXT1 promotes HCC cell growth in vitro and in vivo. **a** Western blot analysis shows HLF, Bel-7402, and MHCC-LM3 cells stably overexpressed with EXT1, versus cells without transfection or transfected with control vector, respectively (upper panel). In lower panel, western blot analysis showed Huh-7 and Hep3-B cells stably knocked down with EXT1, versus cells without transfection or transfected with control vector, respectively. Three shRNA sequences were tested including shEXT1 1–5, 2–1, and 3–1 (Additional file). GAPDH protein level was used as an internal control. **b** CCK-8 assay was performed for the analysis of proliferation ability of the HCC cell lines stably overexpressed or knocked down with EXT1. Values represent the mean ± SEM of three independent transfection experiments (*p* < 0.05). **c** The indicated cells were subjected to In vivo subcutaneous tumor growth curves. 2~4 × 10^6^ tumor cells were injected subcutaneously into the flank of nude mice (*n* = 6). Mice were sacrificed when tumor volume grew into around 1000 mm^3^ and tumor samples were collected, measured, and photographed. HCC cell lines stably overexpressed with EXT1 showed enhanced tumor growth, whereas knockdown of EXT1 in vitro and in vivo both inhibited tumor growth

To further explore the significance of elevated *EXT1* expression in HCC, we analysed a panel of hepatic or HCC cell lines for *EXT1* expression by western blotting (Additional file 1: Figure S6). We used retroviral transduction to overexpress *EXT1* in low *EXT1*-expressing cell lines, including HLF, Bel-7402, and MHCC-LM3. We then knocked down *EXT1* through transfecting lentiviruses carrying a doxycycline-inducible *EXT1*-targeting short hairpin RNA (shRNA) in high *EXT1*-expressing cell lines, named Hep3B-shEXT1 and Huh7-shEXT1. Three shRNA sequences were tested including shEXT1 1–5, 2–1, and 3–1 (Additional file 1). The success of the targeted overexpression or knockdown in HCC cell lines was confirmed by western blotting (Fig. 6a). Cell Counting Kit-8 (CCK-8) and colony formation assays revealed that overexpressing *EXT1* in HLF, Bel-7402, and MHCC-LM3 cells promoted cell growth (Fig. 6b and Additional file 1: Figure S7). Furthermore, in vitro tumorigenicity assays showed that shRNA knockdown of *EXT1* significantly decreased tumor growth of Hep3B and Huh7 cells, compared with cells treated with a scrambled shRNA control. We next used a xenograft animal model to assess the effect of altering *EXT1* expression on HCC cell line proliferation in vivo. Consistent with in vitro studies, there was a significant reduction in tumor volume in mice that had received *EXT1* deficient HCC cells compared with animals that received control Hep3B and Huh7 cells (−vector). By contrast, transplanted *EXT1* overexpressing HCC cells resulted in significantly larger tumors compared with animals that received control HLF, Bel-7402, and MHCC-LM3 cells (−vector) (Fig. 6c).

## Discussion

We identify a patient with multifocal HCC without active virus replication. He had successfully cleared viral DNA from his blood and became immune to the virus, yet years later subsequently developed HCC with the evidence of HBV viral integration. Single cell sequencing and virus DNA capture have the capacity to detect every individual somatic event in parallel for comprehensively identifying viral insertions and quantifying their frequencies, providing us with the opportunity to interrogate the global extent of viral impact on the human genome.

Previous studies based on parallel exome sequencing of HBsAg-seropositive HCC, observed that the virus-affected genes including *VCAM1* and *CDK14* only presented in PVTT but not in the primary tumor, suggesting that genes perturbed by viral integration may contribute to tumor invasive capacity [9–11]. Here we compared the pattern of HBV integrations and genome-wide somatic alterations between primary and metastatic tumor cells through VCS and WGS. The multiple anatomically separate HCC lesions did not share same histological appearance, but there was a strikingly similar pattern of HBV integrations and somatic mutations. These included two HBV integrations together with one inversion structure variation on HBV genome induced the formation of hybrid chromosome between chr1 and chr8. Yet we found significant differences between tumour cells. Profiles of genome-wide somatic alterations allowed us to group the six lesions into three clusters (T1, T2/T3/HVTT, and T4/PVTT), reflecting their evolutionary relationship and supporting that a monoclonal origin located at the site of the largest tumor, T1.

Our functional studies confirmed that one of these genes affected by two HBV_B2 integrations at chr1 and chr8, *CSMD2*, showed a reduced expression in HCC samples compared with the adjacent non-tumor tissues by IHC (Fig. 5). *CSMD2* encoding a member of the C1r/C1s, Uegf, Bmp1 (CUB) and sushi multiple domain protein (CSMD) family – is a candidate TSG associated with colorectal cancer [21]. The altered expression of CSMD2 in HCC samples may indicate a possible role in driving hepatocarcinogenesis. However, CSMD2 is fairly large protein with approximately 380KDa molecular mass and therefore, at the time of our manuscript preparation, we are unable to transduce its expression in HCC cell lines to further address its functions. Conversely we found a viral integration that enhanced the expression of the human exostosin 1 (*EXT1)* gene. Germline mutations of *EXTs* have been linked with cause skeletal abnormalities, short stature and malignant transformation from exostoses to chondrosarcomas [22]. In one report, knockdown of *EXT1* in multiple myeloma cells suppressed tumor growth, resulting in a significantly extended survival in animal model [23]. Although EXT1’s function in HCC is not clear, it has been reported as one of three Interferon-alpha (IFN-α)/5-fluorouracil (5-FU) therapy sensitizing genes in HCC [24]. It is suggested that in the presence of chemotherapy reagents, EXT1 as an endoplasmic reticulum (ER)-resident protein may sensitize HCC cells to 5-FU through ER stress, which is induced by alternating heparin sulfate posttranslational modification. In the current study, we for the first time show EXT1 levels are elevated in human HCC samples compared to the adjacent non-tumor. Furthermore, overexpressing EXT1 in HCC induces the tumor proliferation whereas knockdown EXT1 reduced tumor growth in vitro and in vivo (Fig. 6). Although our functional studies fail to observe that EXT1 plays roles in tumor metastasis (Additional file 1: Figure S8), it may suggest a carcinogenic role of EXT1 in continuing expansion of one of few malignantly altered hepatocytes in the development of multiple HCC, whereas further intra- or extra- hepatic spreading may require the distinctive mechanisms. Future studies will be ensured to address the underlying mechanisms and also determine whether EXT1 level in HCC tissues could serve as a prognostic marker for cancer therapy.

Frequently, the direct roles of HBV in liver carcinogenesis have been suggested. Among those, the HBV X gene shows a transactivating effect linked to HCC via controlling cell growth and apoptosis [25]. Previous studies mostly based on studying HBsAg-positive HCC persons suggest that the usage of this strategic viral breakpoint may facilitate HBV insertions, leading to the formation of chimeric human fusion genes, subsequently interfering with tumor suppressors or imposing *cis*-regulatory effects on the expression of downstream genes [9]. In contrast, the OBI patient in our study with HBsAg-negative serology and undetectable viral DNA in peripheral shows no integration of HBV X gene in host genome. Similarly, Toyoda and colleagues studies the HBV X gene integration in serologically HBV-negative patients. Though detecting the integration of HBV X gene sequence into liver genome in 9 of the 39 patients, they conclude that there is no evidence that HBV-X integration directly plays a role in HCC developments in these serologically HBV-negative patients [26]. The discrepancy may suggest that HBV X gene integration only occurs in some of those serologically positive and viral replicating period in association with a high risk for HCC. However, for those OBI states, the replication-competent viruses are strongly suppressed in their activities by the host’s defense mechanisms, the alternative viral breakpoints may play dominant roles in carcinogenesis.

## Conclusions

In summary, this study provides deep insight into clonal evolution of HCC in the absence of viral replication, supporting a monoclonal HCC origin theory. The continuing expansion of one of few malignantly altered hepatocytes further promotes intra- or extra- hepatic spreading, leading to the development of multiple HCC. It suggests that viral integration and viral active replication may serve as two separate mechanisms for the initiation and subsequently maintenance of the transformed stage in HCC.

## Additional file

Additional file 1: **Figure S1.** HBV breakpoint detection workflow from sequencing reads in FuseSV. **Figure S2.** HBV sub-genotype tree in host by VCS analysis. **Figure S3.** Constructing HBV genomes in tumors by VCS analysis. **Figure S4.** Comparison of genome-wide somatic SNVs among the six tumor specimens. **Figure S5.** Comparison of genome-wide copy number in six tumor specimens. **Figure S6.** EXT1 expression in hepatic or HCC cell lines by western blot**. Figure S7.** EXT1 affects HCC growth in vitro*.* **Figure S8.** EXT1 does not affect HCC cell migration in vitro*.* **Table S1.** Clinicopathologic features of the HBV patient with multiple HCC. **Table S2.** Sequencing data summary and HBV genome details of ten specimens. **Table S3.** VCS data summary and constructed HBV genome details of 264 single cells. **Table S4.** Alterations on HBV_B2 genome in ten specimens. **Table S5.** Homologous sequences at the junction sites of minor viral integration and HBV rearrangement cases. **Table S6.** Copy number alterations of six tumor specimens. **Table S7.** Somatic structure variations of six tumor specimens. (PDF 5584 kb)

## Data Availability

All data generated or analyzed during this study are included either in this article or in the additional files.

## Abbreviations

5-FU: 5-fluorouracil
CCK-8: Cell Counting Kit-8
CIN: Chromosome instability
CNV: Copy number variation
CSMD: CUB and sushi multiple domain
EXT1: Exostosin 1
HBeAg: HBV e antigen
HBsAg: HBV surface antigen
HBV: Hepatitis B virus
HCC: Hepatocellular carcinoma
IFN-α: Interferon-alpha
IHC: Immunohistochemistry
IVCTT: Inferior vena cava tumor thrombi
LOH: Loss of heterozygosity
MDA: Multiple Displacement Amplification
MRI: Magnetic resonance imaging
OBI: Occult HBV infection
PVTT: Portal vein tumor thrombi
shRNA: Short hairpin RNA
SNVs: Single nucleotide variants
VCS: Virome capture sequencing
WGS: Whole genome sequencing
