# Correction to: Viral integration drives multifocal HCC during the occult HBV infection

**DOI:** 10.1186/s13046-019-1484-5

**Published:** 2019-12-04

**Authors:** Xiao-Ping Chen, Xin Long, Wen-long Jia, Han-Jie Wu, Jing Zhao, Hui-Fang Liang, Arian Laurence, Jun Zhu, Dong Dong, Yan Chen, Long Lin, Yu-Dong Xia, Wei-Yang Li, Gui-Bo Li, Zhi-Kun Zhao, Kui Wu, Yong Hou, Jing-Jing Yu, Wei Xiao, Guo-Ping Wang, Peng-Cheng Zhu, Wei Chen, Ming-Zhou Bai, Yi-Xing Jian, Karsten Kristiansen, Qian Chen

**Affiliations:** 1 The Hepatic Surgery Centre at Tongji Hospital, Tongji Medical College, HUST; Hubei Province for the Clinical Medicine Research Center of Hepatic Surgery, Key Laboratory of Organ Transplantation, Ministry of Education and Ministry of Public Health, Wuhan, 430030 China; 2 Department of Computer Science, City University of Hong Kong, Hong Kong, People’s Republic of China; 3 School of Biomedical Engineering, University of Technology Sydney, Sydney, NSW2007 Australia; 4 Department of Biology, Laboratory of Genomics and Molecular Biomedicine, University of Copenhagen, Universitesparken 13, 2100 Copenhagen, Denmark; 5 The Newcastle upon Tyne Hospitals NHS Foundation Trust at Freeman Hospital, Newcastle, UK; 6 Department of Genetics and Genomic Sciences, Icahn Institute of Genomics and Multiscale Biology, Icahn School of Medicine at Mount Sinai, 1425 Madison Avenue, New York, NY USA; 7 Laboratory of Molecular Ecology and Evolution, Institute of Estuarine and Coastal Research, East China Normal University, Shanghai, China; 8 School of Bioscience and Bioengineering at South China University of Technology, Guangzhou, China; 9 School of Biological Science and Medical Engineering at Southeast University, Nanjing, China; 10 The Department of Pathology at Tongji Hospital, Tongji Medical College, HUST, Wuhan, 430030 China; 11 The Division of Gastroenterology, Department of Internal Medicine at Tongji Hospital, Tongji Medical College, Huazhong University of Science and Technology (HUST), Wuhan, 430030 China

**Correction to: J Exp Clin Cancer Res (2019) 38:261**

**https://doi.org/10.1186/s13046-019-1273-1**

In the original publication of this article [1], Fig. 6 is wrong and the updated figure is shown below.

**Figure 1:**
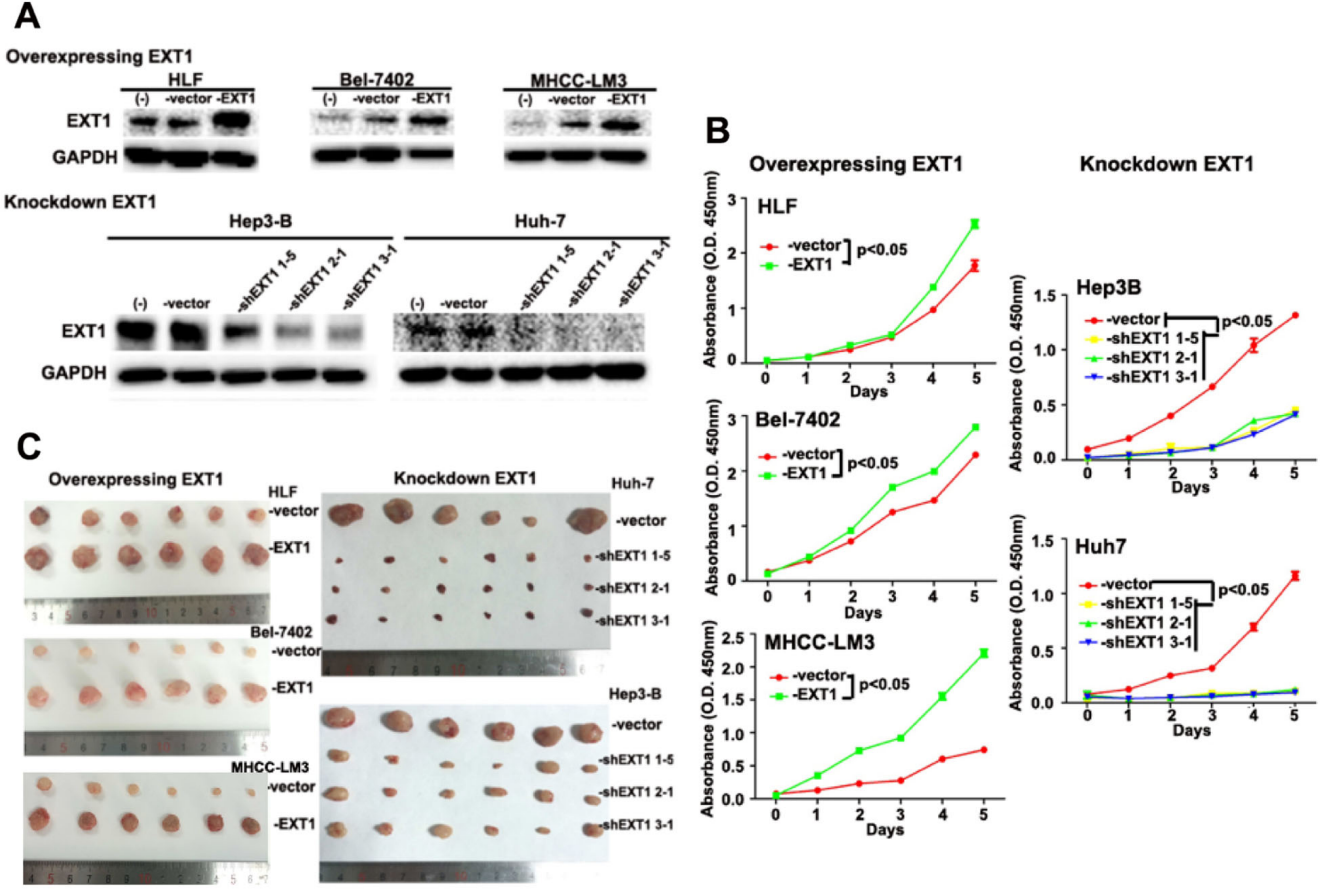
Fig. 6

The authors sincerely apologize for the inconvenience caused to the readers.
