# Supplementary material for: Viral integration drives multifocal HCC during the occult HBV infection
Source: J Exp Clin Cancer Res. 2019 Jun 14;38:261. doi: 10.1186/s13046-019-1273-1 (PMC6570863; doi:10.1186/s13046-019-1273-1)
Supplement: Supplementary file 1 — Figure S1. HBV breakpoint detection workflow from sequencing reads in FuseSV. Figure S2. HBV sub-genotype tree in host by VCS analysis. Figure S3. Constructing HBV genomes in tumors by VCS analysis. Figure S4. Comparison of genome-wide somatic SNVs among the six tumor specimens. Figure S5. Comparison of genome-wide copy number in six tumor specimens. Figure S6. EXT1 expression in hepatic or HCC cell lines by western blot. Figure S7. EXT1 affects HCC growth in vitro. Figure S8. EXT1 does not affect HCC cell migration in vitro. Table S1. Clinicopathologic features of the HBV patient with multiple HCC. Table S2. Sequencing data summary and HBV genome details of ten specimens. Table S3. VCS data summary and constructed HBV genome details of 264 single cells. Table S4. Alterations on HBV_B2 genome in ten specimens. Table S5. Homologous sequences at the junction sites of minor viral integration and HBV rearrangement cases. Table S6. Copy number alterations of six tumor specimens. Table S7. Somatic structure variations of six tumor specimens. (PDF 5584 kb) [file 13046_2019_1273_MOESM1_ESM.pdf]

Supplementary Materials

Viral integration drives multifocal HCC during the occult HBV infection

Xiao-Ping Chen[1#], Xin Long[1,#], Wen-long Jia[2,#], Han-Jie Wu[3,#], Jing Zhao[4], Hui-Fang Liang[1], Arian Laurence[5], Jun Zhu[6], Dong Dong[7], Yan Chen[1], Long Lin[1], Yu-Dong Xia[1], Wei-Yang Li[1], Gui-Bo Li[8], Zhi-Kun Zhao[9], Kui Wu[1], Yong Hou[1], Jing-Jing Yu[1], Wei Xiao[1], Guo-Ping Wang[10], Peng-Cheng Zhu[10], Wei Chen[1], Ming-Zhou Bai[1], Yi-Xing Jian[1], Karsten Kristiansen[4] & Qian Chen[11*]

[1]The Hepatic Surgery Centre at Tongji Hospital, Tongji Medical College, HUST; Hubei Province for the Clinical Medicine Research Center of Hepatic Surgery; Key Laboratory of Organ Transplantation, Ministry of Education and Ministry of Public Health, Wuhan, 430030, China.

[2]Department of Computer Science, City University of Hong Kong, Hong Kong, P.R. China

[3]School of Biomedical Engineering, University of Technology Sydney, Sydney, NSW2007, Australia

[4]Laboratory of Genomics and Molecular Biomedicine, Department of Biology, University of Copenhagen, Universitesparken 13, 2100 Copenhagen, Denmark

[5]The Newcastle upon Tyne Hospitals NHS Foundation Trust at Freeman Hospital, Newcastle, U.K

[6]Department of Genetics and Genomic Sciences, Icahn Institute of Genomics and Multiscale Biology, Icahn School of Medicine at Mount Sinai, 1425 Madison Avenue, New York, NY, U.S

[7]Laboratory of Molecular Ecology and Evolution, Institute of Estuarine and Coastal Research, East China Normal University, Shanghai, China

[8]School of Bioscience and Bioengineering at South China University of Technology, Guangzhou, China

[9]School of Biological Science and Medical Engineering at Southeast University, Nanjing, China

[10]The Department of Pathology at Tongji Hospital, Tongji Medical College, HUST, Wuhan, 430030, China

[11]The Division of Gastroenterology, Department of Internal Medicine at Tongji Hospital, Tongji Medical College, Huazhong University of Science and Technology (HUST), Wuhan, 430030, China

[#]These authors contributed equally

[*]Corresponding author

*Corresponding authors: Professor Qian Chen, Mailing address: The Division of Gastroenterology, Department of Internal Medicine at Tongji Hospital, HUST, Jie Fang Avenue 1095, Wuhan 430030, P.R. China; Tel: +86 27-83663500; Fax: +86 27-83663500; Email: chenqian201579@yahoo.com

Contents

Experimental Procedure Notes

1. The clinicopathologic features of the HBV patient with multiple HCC

2. Evaluation of CSMD2 and EXT1 expression by IHC

3. HBV variant assignment

4. The HBV integration breakpoint detection procedure in FuseSV

5. HBV-human conjunct sequence enrichment and sequencing

6. Detection of somatic alterations in six tumors

7. Construction of HBV-integrated local haplotype

8. shRNA oligos

9. Generation of stable EXT1 overexpression or knockdown cell lines

10. Cell proliferation, migration, colony formation and soft agar clonogenic assays

Supplementary Tables and Figure Legends

Figure S1: **HBV breakpoint detection workflow from sequencing reads in FuseSV**

Figure S2: **HBV sub-genotype tree in host by VCS analysis**

Figure S3: **Constructing HBV genomes in tumors by VCS analysis**

Figure S4: **Comparison of genome-wide somatic SNVs among the six tumor specimens**

Figure S5: **Comparison of genome-wide copy number in six tumor specimens**

Figure S6: **EXT1 expression in hepatic or HCC cell lines by western blot**

Figure S7: **EXT1 affects HCC growth *in vitro***

Figure S8: **EXT1 does not affect HCC cell migration *in vitro***

Table S1: **Clinicopathologic features of the HBV patient with multiple HCC**

Table S2: **Sequencing data summary and HBV genome details of ten specimens**

Table S3: **VCS data summary and constructed HBV genome details of 264 single cells**

Table S4: **Alterations on HBV_B2 genome in ten specimens**

Table S5: **Homologous sequences at the junction sites of minor viral integration and HBV rearrangement cases**

Table S6: **Copy number alterations of six tumor specimens**

Table S7: **Somatic structure variations of six tumor specimens**

1. The clinicopathologic features of the HBV patient with multiple HCC

The study was approved by the Institutional Review Board of Tongji Hospital, Tongji Medical College of HUST, in Hubei province, China. The signed written informed consent was obtained before patients' recruitment, according to the regulations of the institutional ethics review boards. Initially, five patients were recruited for this study. Information about risk factors for HCC and other clinical pathologic characteristics was retrieved from patients' medical records. Thorough evaluation with physical examination, liver function tests, coagulation, and nutritional assessment for degree of baseline hepatic insufficiency were conducted to check that the patient was eligible for a liver resection. Imaging of the liver was undertaken for the detection and characterization of suspected multiple HCC, prior to planning a surgery. Only one case matched to our research design referring that the patient had occult HBV infected phase and the multiple HCC tissues were available during the surgery. The case was selected for studying the pattern of HBV integration in single cell level. Information about the duration of follow-up and status at the date of last information, were obtained from this patient's medical records.

One patient who matched to our research design was a 47-year-old Chinese male who underwent liver resection in May 2013, for what was originally diagnosed as HCC by an abdominal computer tomography (CT) scan. Medical history was obtained indicating he had no history of alcohol abuse, recognized acute hepatitis, mother-to-child transmission of HBV, blood transfusion, or injection drug use. Hepatitis serology tests

showed that the patient had an HBs Ab level of 884.5 mIU/mL, was HBs Ag-negative, HBc Ab-positive, HBe Ab-positive, HCV Ab-negative and had an undetectable blood HBV DNA copy number, all of which was consistent with occult HBV infection (Table S1). He has normal ALT (7 U/L) and mildly elevated AST (71 U/L). As part of the preoperative evaluation process, the serum Alpha-fetoprotein (AFP) concentration, a biomarker for HCC, was found to be elevated at 1210 ng/mL. Liver magnetic resonance imaging (MRI) revealed a 15cm x 10cm main lesion in the left hepatic lobe and multiple smaller lesions in the right hepatic lobe, all under 3 cm in diameter. MRI with contrast enhancement showed tumor thrombi involved in the right portal vein branch (PVTT) and inferior vena cava (IVCTT), indicating the intrahepatic and extrahepatic vascular spreading of HCC (Figure 1).

Multiple HCC were determined as consisting of a primary large tumor, multiple smaller satellite nodules surrounding the tumor, and invasive tumor thrombi (TT) in the hepatic portal vein (PV) and inferior vena cava (IVC), respectively. HCC specimens, including the multiple tumors in liver, their adjacent non-tumorous liver tissues, right portal vein tumor thrombosis (PVTT), as well as inferior vena cava tumor thrombosis (IVCTT) were obtained surgically from this patient, whose tissues were examined histologically and subsequently shipped to the Liver Surgery and Transplantation Department at Tongji Hospital in Wuhan, where pathologic analysis confirmed HCC status and indicated that adjacent tissues were primarily cirrhotic (Figure 1C&D). Immunohistochemistry for Arginase-1 (Arg-1) was performed using a polyclonal rabbit antihuman antibody (HPA003595, Sigma-Aldrich, St Louis, MO), and for Hepatocyte Paraffin-1 (HepPar-1)

was performed using a monoclonal mouse antihuman hepatocyte antibody (clone OCH1E5; DakoCytomation, Carpinteria, CA). A DAKO staining system was used to stain the tissues (DAKO, Carpinteria, CA) according to the manufacturer's protocol. Two pathologists (G-P.W and P-C.Z) independently evaluated the immunostained slides.

Tumors were diagnosed by light microscopy with criteria established from malignant tumors of the liver according to the World Health Organization classification system[1]. Tumors were classified into 4 grades (well, moderate, poor, and undifferentiated/anaplastic) based on a modified Edmondson and Steiner grading system[1]. The patient was staged according to the 2002 American Joint Commission on Cancer TNM staging system[2].

A pathology report following hepatectomy revealed the presence of multiple, moderately to poorly differentiated hepatocellular carcinomas, trabecular and solid type, with involvement of the PV and HV (Figure 1D). There was evidence of regional lymph node involvement. The remainder of the liver parenchyma showed cirrhotic changes. The patient was subsequently staged as T4N1M0, Stage IV hepatocellular carcinoma. Follow-up CT scans after 7 months showed recurrence of the disease with the development of many new nodules scattered throughout the remaining liver together with peritoneal metastases. Despite further treatment with chemotherapy, the patient died 10 months after the surgery.

2. Evaluation of CSMD2 and EXT1 expression by IHC

For validation study, 50 poorly-differentiated HCC tissues and their paired adjacent non-tumor tissues were acquired from patients who were performed liver resection during 2013 to 2014, at Hepatic surgery Center, Tongji Hospital, Tongji Mecdical college, HUST (Wuhan, China). Paraffin-embedded (FFPE) tissue sections were first placed in 65°C incubator for 30 minutes to deparaffinating, then transferred into xylene for 10 minutes, dipped into 100% absolute ethanol, 90% ethanol, 80% ethanol, 70% ethanol, and washed by distilled water and PBS for hydrating. Liver tissues immerged in citrate buffer were heated in microwave on high for 8 minutes and low for 10 minutes for heat-induced epitope retrieval. Liver tissues were washed by distilled water and PBS and then immerged into 3% H_2O_2 in the dark room for blocking endogenous peroxidase. 5% BSA (Bovine Serum Albumin, Sigma-Aldrich, USA) was used to block non-specific epitope. 50 µl primary rabbit anti-CSMD2 antibody (1:200, Abcam, Cambridge, UK) and rabbit anti-EXT1 antibody (ab126305, 1:100) were added to each liver sample and the same amount of rabbit IgG isotype (A7016, Beyotime, Shanghai, China) was set as negative control for each slide with incubation at 4°C overnight. Each slide was incubated with 50 µl the second antibody complex (DAKO, Denmark) for 30 minutes at room temperature after three times washing and followed by DAB (DAKO, Denmark) dyeing. The slides were then stained by hematoxylin for 5 minutes, differentiated by hydrochloric acid alcohol for 5 seconds and dipped into diluted ammonia water for 8 seconds. All slides were rinsed in 70% ethanol, 80% ethanol, 90% ethanol, 100% absolute ethanol and xylene one by one. Neutral balsam was used to enclose the liver tissue before covered by cover glass for microscopic examination[3].

The protocol for scoring slides has been described previously[3, 4]. Briefly, the total score of each slide = score of staining intensity x score of number of positive staining cells. Staining intensity was grade as follows: 0, negative; 1, light yellow; 2, light brown; 3, brown. The number of positive cells was scored as 0 (0-10%), 1 (11%-25%), 2 (26%-50%), 3 (51%-75%), 4 (76%-100%). The scoring was performed independently by three pathologists without clinical knowledge of the patients.

3. HBV variant assignment

HBV variant was identified from sequencing data. Reads were firstly aligned against human genome reference (hg19). Unmapped reads and soft-clipped reads were extracted from alignment results. Then we aligned these reads to HBV reference database downloaded from NCBI nucleotide database. The database of reference HBV variants includes: HBV_A1 (AY738139.1), HBV_A2 (HE576989.1), HBV_A3 (AB194951.1), HBV_A4 (AY934764.1), HBV_A5 (FJ692613.1), HBV_A6 (GQ331047.1), HBV_A7 (FN545833.1), HBV_B1 (AB642091.1), HBV_B2 (FJ899779.1), HBV_B3 (GQ924617.1), HBV_B4 (GQ924626.1), HBV_B5 (GQ924640.1), HBV_B6 (JN792893.1), HBV_B7 (GQ358137.1), HBV_B8 (GQ358147.1), HBV_B9 (GQ358149.1), HBV_C1 (AB697490.1), HBV_C10 (AB540583.1), HBV_C11 (AB554019.1), HBV_C12 (AB554025.1), HBV_C13 (AB644280.1), HBV_C14 (AB644284.1), HBV_C15 (AB644286.1), HBV_C16 (AB644287.1), HBV_C2 (GQ358158.1), HBV_C3 (DQ089801.1), HBV_C4 (HM011493.1), HBV_C5 (EU410080.1), HBV_C6 (EU670263.1), HBV_C7 (GU721029.1), HBV_C8 (AP011106.1), HBV_C9 (AP011108.1), HBV_D1 (GU456636.1), HBV_D2 (GQ477452.1),

HBV_D3 (EU594434.1), HBV_D4 (GQ922003.1), HBV_D5 (GQ205377.1), HBV_D6 (KF170740.1), HBV_D7 (FJ904442.1), HBV_D8 (FN594770.1), HBV_D9 (JN664942.1), HBV_E (FN594748.1), HBV_F1a (AY090459.1), HBV_F1b (FJ709464.2), HBV_F2a (DQ899142.1), HBV_F2b (DQ899146.1), HBV_F3 (AB036920.1), HBV_F4 (AF223965.1), HBV_G (GU563556.1), HBV_H (AB516393.1), HBV_I1 (FJ023659.1), HBV_I2 (FJ023664.1), HBV_J (AB486012.1). The HBV variant with most uniquely aligned reads and at least 20% coverage was selected as first HBV variant. As the HBV variant of individual sample could have plenty of mutations diffed from HBV variant references downloaded online. We introduced iterative process to construct individual HBV genomes. In this process, we applied the detected mutations (SNV and InDel) to modify corresponding HBV variant genome. Next, we remapped the candidate reads again to this modified new HBV variant reference, and tested whether new mutations could be found out. If there are new mutations, viral genome modification will be done again, and reads will also be remapped to the newest modified HBV variant genome, till there is no new mutation could be identified any more. All mutations used to modify the HBV genomes were gathered in the final mutation list (Table S4).

4. The HBV integration breakpoint detection procedure in FuseSV

A framework for the virus presence and viral integration in the host genome was constructed. The framework can deal with either the raw sequencing data or the alignment result in Sequence Alignment/Map (SAM) format. Paired-end reads related with the virus are extracted to determine the connecting partner candidate. And

unmapped reads are defined as candidate junction reads that may likely cover the breakpoint. Then the partial exhaustion algorithm is applied to construct the putative junction sequence library. Candidate junction reads are all aligned to the putative junction sequence library to support the initial integrations. Following filtrations are implemented on the initial virus integrations. The framework consists of six parts (Figure S1): basic information, reads process, obtain candidate, obtain integration, reads refinement, and result outputs.

From the NCBI-Nucleotide/Viruses database, we obtained the reference genome ("basic information" in Figure S1). All reads falling into the virus genomic region are extracted via reading indexed bam alignment result by some tools ("reads process" in Figure S1). From the read group, we could select the discordantly mapped reads and the soft-clipped reads. The discordantly mapping cases include aligning in same strand, locating at reversed positions or even on different chromosomes, and having the obviously abnormal insert size. Soft-clipped reads were searched for the location of virus breakpoint. The reads covering the virus junction position could only be aligned to one partner as long as discarding the part of read sequence that from the other partner. After trimming, the involved paired-end reads may become to a pair of discordantly mapped reads. We use partial exhaustion algorithm, to reconstruct the putative junction sequence with the paired soft-clipped reads as the span-reads ("obtain integration" in Figure S1). The partial exhaustion algorithm firstly calculates the region of breakpoint location from the span-reads information and the insert size of their corresponding library. Then, as the soft-clipped reads should be closed to the breakpoint, it splits the

span-reads' region by the soft-clipped reads: covered region by soft-clipped reads is called credible-region, and the other uncovered region is called potential-region. Finally, it concatenates each base from the two partners in pairwise way with the only limitation that two bases could not come from potential-region simultaneously. Putative junction sequence library is constructed as the reference for the next realignment of soft-clipped and unmapped reads. From the realignment results, we can know which putative integration cases are supported. The micro-homology and inner insertion at the virus breakpoints are relevant to the integration mechanisms. We applied another module specialize to ensure accuracy of the breakpoint detection. After obtaining integration sites supported by spanning reads and junction reads, the whole human-virus segment could be generated based on the breakpoint and the supporting reads. Then, realign all supporting reads to the segments could refine the breakpoint ("reads refinement" in Figure S1). For example, the homozygous continuous mismatch could be transfer to the inner insertion. As the virus breakpoint become accurate, the feature around it will be available and credible.

5. HBV-human conjunct sequence enrichment and sequencing

The small insert-size DNA libraries were constructed following the instruction of Illumina. 45 qualified single-cell amplification products from each lesion (T1-T4, PVTT and HVTT) were selected to construct the sequencing libraries. The qualified single cell amplification DNA was sheared into about 180 bp fragments by Covaris E-210. These fragments were end blunted, "A" base tailed and adaptor ligated. 10 cycles PCR with an 8

bases barcode of primers were performed after the 160-200 bp size selection steps. The insert size and concentration of libraries were analyzed by Bioanalyzer 2100 (Agilent). Then the HBV genome capture process was conducted according to the MyGenostics GenCapTM target Enrichment Protocol. HBV probes fabricated from full length of HBV genome were hybridized with DNA libraries at 65°C for 24 hours and washed to remove the unhybridized fragments. The eluted libraries were amplified by 8 cycles PCR to produce enough DNA products for the next generation sequencing[5]. After the concentration and insert size were measured, libraries were processed for the pair-end 101 base pair sequencing on the Illumina Hiseq 2000 platform according to the manufacturer's instructions, with the average data amount of 1 Giga base pairs per library.

6. Detection of somatic alterations in six tumors

The high quality WGS reads were aligned to the human reference genome (hg19) using BWA (v0.5.9) [6] with the default parameters. Picard (v1.54) was employed to mark duplicates. We used MuTect2[7] to detect somatic SNVs and short InDels. The minimum depth was set to 10X for tumor genomes, and the minimum number of mutation supporting reads in tumor was set to 3X. All high confident mutations were annotated with wANNOVAR[8]. Copy number alterations were detected by Patchwork[9]. Meerkat[10] was applied to identify structural variations. Minimum count of supporting reads pair and split read were both set to three.

7. Construction of HBV-integrated local haplotype À

Copy number alterations (CNAs) were detected by tool Patchwork[9] from WGS data. We found the genomic regions nearby the HBV integrations showed copy number changes (Figure S5). Moreover, the edges of the duplicated genomic regions were very close to the break-points of HBV integrations (Figure 2 and Figure 3). These CNAs relevant to the duplicated genomic regions inferred the genomic instability adjacent to HBV integrated sites, consistent with previous reports[11, 12]. Further analysis of the CNAs determined that the edges of these duplicated genomic regions were exactly the break-points of HBV integrations (Figure 2A and Figure 3A&B).

Considering cancer genomic region flanking the HBV 18 integration sites in HeLa cell line has been well characterized, and the local genomic map of HBV integrations in several other cancer cell lines were also reported in details. We resolved the linear structure of the genomic regions around HBV integrations in tumor cells of this samples, as reported in HPV analysis[13, 14], called as "local haplotype".

Firstly, the genomic region was divided into several segments (named by upper cases in Figure 2A and Figure 3A&B) based on the break-points of HBV integrations, and the individual HBV genome was also partitioned into several segments (named by lower cases in Figure 2B and Figure 3C) based on the viral break-points corresponding to the HBV integrations.

Secondly, from WGS data of tumor and control samples, the average depth of each segment was calculated using 'depth' command of SamTools [v1.3, [15]] with minimum base and mapping quality set as 5 and 10, respectively. To convert the average depth

from WGS data of tumor sample, we introduced purity and ploidy of tumor-cell in tumor sample calculated by tool ABSOLUTE [[16]; Table S2]. Call the purity of tumor-cell in tumor sample as $\boldsymbol{Purity_T}$, the average ploidy of pure tumor-cell in tumor sample as $\boldsymbol{Ploidy_T}$, and the average ploidy of pure normal cell as $\boldsymbol{Ploidy_N}$ (assumed as 2). So the average ratio of the part from normal cells ($\boldsymbol{Ratio_N}$) in each genomic region was calculated as below:

$$\boldsymbol{Ratio_N} = 1 - \frac{\boldsymbol{Purity_T} * \boldsymbol{Ploidy_T}}{\boldsymbol{Purity_T} * \boldsymbol{Ploidy_T} + (1 - \boldsymbol{Purity_T}) * \boldsymbol{Ploidy_N}}$$

Then for each segment, we could also know its germline copy ratio from WGS data of control sample ($\boldsymbol{Copy\_ratio_N}$). So the depth of this segment from pure tumor-cell in tumor sample ($\boldsymbol{Detph_T}$) could be obtained from this segment's original average depth ($\boldsymbol{Detph_O}$) and whole genome average depth ($\boldsymbol{Detph_W}$) via formula below:

$$\boldsymbol{Detph_T} = \boldsymbol{Detph_O} - \boldsymbol{Detph_W} * \boldsymbol{Ratio_N} * \boldsymbol{Copy\_ratio_N}$$

Thirdly, from CNA results reported by Patchwork[9], we could know the total and minor copy number of chromosome arms and CNA segments. The most bilateral regions in "local haplotype" or normalized depth of control samples were properly used to calculate the depth of single copy number. The copy times of HBV integrated or normal local haplotype were determined manually according to the copy number result from Patchwork. Then, the copy number of segments in "local haplotypes" could be determined (Figure 2A). Based on the segment connection relationship provided by viral integrations and SVs, we applied "Linear Programming" and "Weighted Oriented

Eulerian Path" algorithms (developed in-house software) to construct continuous segments path as single HBV integrated local haplotype with different copy times, which cost minimum changes on copy number of segments (Figure 2C).

8. shRNA oligos

The EXT1 shRNA oligonucleotides were annealed and inserted into the AgeI / EcoRI site of the small hairpin RNA (shRNA) expression vector Tet-pLKO-puro (Addgene plasmid 21915).

Scramble shRNA was used as a negative control. shRNA oligo sequences from Sigma as follows:

Sequence 1:

CCGGCAATTGTGAGGACATTCTCATCTCGAGATGAGAATGTCCTCACAATTGTTTTTG;

Sequence 2:

CCGGCCCAACTTTGATGTTTCTATTCTCGAGAATAGAAACATCAAAGTTGGGTTTTTG;

Sequence 3:

CCGGCCTTCGTTCCTTGGGATCAATCTCGAGATTGATCCCAAGGAACGAAGGTTTTTG

9. Generation of stable EXT1 overexpression or knockdown cell lines

Human EXT1 CDNA was produced from EXT1 pENTER vector (CH895686, ViGene Biosciences, Rockville, MD, USA) using primers:

GCGGATCCATTATTATTCGCCACCATGCAGGCCAAAAAACGCTATTT,

GGAATTCTCACTTATCGTCGTCATCCTTGTAATCAAGTCGCTCAATGTCTCGGTATT.

For overexpression, full-length human EXT1 with flag or empty vector was cloned into pBABE-puro vector (Addgene plasmid 1764) digested by BamHI and EcoRI. Retroviral supernatants were produced by transfection of 293FT cells, a kindly gift from Dr. Zhanguo Zhang (Hepatic Surgery Center, Tongji Hospital, Huazhong University of Science and Technology), using X-tremeGene HP DNA Transfectant (06366236001, Roche, Basle, Switzerland).

For shRNA known-down, Scrambled or EXT1-targeting shRNAs were cloned as annealed oligonucleotides (Sigma) into Tet-pLKO-puro (Addgene plasmid 21915) digested with AgeI and EcoRI according to provided protocol from Addgene. Ten clones of each sequence were selected for sequencing. PLKO-Tet-On EXT1 shRNA constructs were confirmed and named as shEXT1 1-5, shEXT1 2-1 and shEXT1 3-1. Sequence-verified clones were used to produce lentiviral particles according to the protocols. Lentiviral supernatants were produced by transfection of 293FT cells using X-tremeGene HP DNA Transfectant.

The collected supernatants were filtered through a 0.45 μm filter (PALL, Port Washington, NY, USA), and subsequently concentrated by Centricon Plus 70 (Merck Millipore, Darmstadt, Germany) according to the manufacturer's instructions. Concentrated retrovirus or lentivirus supernatants were added on the HCC cell lines with a multiplicity of infection (MOI) ranging from 30 to 50 in the presence of polybrene

(8 μ g/ml) for 48 hours (retrovirus) or 72 hours (lentivirus). For both overexpression and knockdown cells, stable pools were selected in growth medium containing 5 μ g/mL puromycin (Sigma-Aldrich) for 2 weeks, and stable lines were maintained in selection media. The optimal doxycycline dose for inducible knockdown was determined to be 100ng/ml, which was the dose that resulted in maximal knockdown of EXT1 after 48h.

The effect of EXT1 overexpression or knockdown was assessed by western blot using antibody to EXT1 (abcam, ab126305) or GAPDH (Santa Cruz Biotechnology, sc-25778), and horseradish peroxidase (HRP)-conjugated secondary antibodies, followed by signal detection with chemiluminescence (GE Healthcare Life Sciences).

10. Cell proliferation, migration, colony formation and soft agar clonogenic assays

Cell proliferation was measured using the Cell Counting Kit-8 (CCK-8) (Beyotime Institute of Biotechnology, China) according to the manufacturer's instructions. HCC cell lines were incubated with CCK-8 for 2 hour at indicated time points. Cell proliferation rate was assessed by measuring the absorbance at 450 nm with the Universal Microplate Reader (Bio-Tek Elx 800, USA).

For migration assays, cells at a density of 5×10^4 in 250μl of DMEM medium containing 0.2% FBS were seeded into the upper chamber of the 24-well 8-μM pore transwell plate (Corning, NY, USA). 500μl of medium containing 10% FBS were added to the lower chambers. After 24 hours, cells on the top side of each insert were scraped off and the wells were fixed in 4% phosphate-buffered neutral formalin (PH7.4), stained by crystal

violet and the transwell membrane was visualized under Nikon Digital ECLIPSE C1 system (Nikon Corporation). Photographs of 5 random fields across three replicate wells were captured for quantification analysis. Migrated cells were counted by the Image-Pro Plus v6.0 software (Media Cybernetics Inc.).

For colony formation assay, cells were plated in 6-well plates at a density of $0.5\sim2\times10^3$ cells per well. After 14 days, the colonies were stained with crystal violet for 30 minutes and rinsed with deionized distilled water, then photographed using a Nikon D80 digital camera (10 Mega-pixel; Nikon Corp., Tokyo, Japan) and scored using AlphaEase FC software (Alpha Innotech Inc., San Leandro, CA, USA).

For soft agar colony formation assay, cells were plated in 6-well plates at a density of $2\times10^3\sim2\times10^4$ cells per well. Cells suspended in 1.0 mL of top agar (0.4% agar in culture medium) were overlaid onto a layer of 1.0 mL of bottom agar (0.8% agar in the same culture medium). After 14 days, the colonies were photographed and scored by inverted phase-contrast microscopy without fixation and staining.

Reference:

1. Xu X, Hou Y, Yin X, Bao L, Tang A, Song L, Li F, Tsang S, Wu K, Wu H, He W, Zeng L, *et al*. Single-cell exome sequencing reveals single-nucleotide mutation characteristics of a kidney tumor. *Cell* 2012;**148**:886-95.

2. El-Serag HB. Hepatocellular carcinoma. *N Engl J Med* 2011;**365**:1118-27.

3. Ding ZY, Jin GN, Wang W, Chen WX, Wu YH, Ai X, Chen L, Zhang WG, Liang HF, Laurence A, Zhang MZ, Datta PK, *et al*. Reduced expression of transcriptional intermediary factor 1 gamma promotes metastasis and indicates poor prognosis of hepatocellular carcinoma. *Hepatology* 2014;**60**:1620-36.

4. Tao ZH, Wan JL, Zeng LY, Xie L, Sun HC, Qin LX, Wang L, Zhou J, Ren ZG, Li YX, Fan J, Wu WZ. miR-612 suppresses the invasive-metastatic cascade in hepatocellular carcinoma. *J Exp Med* 2013;**210**:789-803.

5. Li W, Zeng X, Lee NP, Liu X, Chen S, Guo B, Yi S, Zhuang X, Chen F, Wang G, Poon RT, Fan ST*, et al*. HIVID: an efficient method to detect HBV integration using low coverage sequencing. *Genomics* 2013;**102**:338-44.

6. Li H, Durbin R. Fast and accurate short read alignment with Burrows-Wheeler transform. *Bioinformatics* 2009;**25**:1754-60.

7. Cibulskis K, Lawrence MS, Carter SL, Sivachenko A, Jaffe D, Sougnez C, Gabriel S, Meyerson M, Lander ES, Getz G. Sensitive detection of somatic point mutations in impure and heterogeneous cancer samples. *Nat Biotechnol* 2013;**31**:213-9.

8. Wang K, Li M, Hakonarson H. ANNOVAR: functional annotation of genetic variants from high-throughput sequencing data. *Nucleic Acids Res* 2010;**38**:e164.

9. Mayrhofer M, DiLorenzo S, Isaksson A. Patchwork: allele-specific copy number analysis of whole-genome sequenced tumor tissue. *Genome Biol* 2013;**14**:R24,2013-14-3-r24.

10. Yang L, Luquette LJ, Gehlenborg N, Xi R, Haseley PS, Hsieh CH, Zhang C, Ren X, Protopopov A, Chin L, Kucherlapati R, Lee C*, et al*. Diverse mechanisms of somatic structural variations in human cancer genomes. *Cell* 2013;**153**:919-29.

11. Neuveut C, Wei Y, Buendia MA. Mechanisms of HBV-related hepatocarcinogenesis. *J Hepatol* 2010;**52**:594-604.

12. Sung WK, Zheng H, Li S, Chen R, Liu X, Li Y, Lee NP, Lee WH, Ariyaratne PN, Tennakoon C, Mulawadi FH, Wong KF*, et al*. Genome-wide survey of recurrent HBV integration in hepatocellular carcinoma. *Nat Genet* 2012;**44**:765-9.

13. Adey A, Burton JN, Kitzman JO, Hiatt JB, Lewis AP, Martin BK, Qiu R, Lee C, Shendure J. The haplotype-resolved genome and epigenome of the aneuploid HeLa cancer cell line. *Nature* 2013;**500**:207-11.

14. Akagi K, Li J, Broutian TR, Padilla-Nash H, Xiao W, Jiang B, Rocco JW, Teknos TN, Kumar B, Wangsa D, He D, Ried T*, et al*. Genome-wide analysis of HPV integration in human cancers reveals recurrent, focal genomic instability. *Genome Res* 2014;**24**:185-99.

15. Li H, Handsaker B, Wysoker A, Fennell T, Ruan J, Homer N, Marth G, Abecasis G, Durbin R, 1000 Genome Project Data Processing Subgroup. The Sequence Alignment/Map format and SAMtools. *Bioinformatics* 2009;**25**:2078-9.

16. Carter SL, Cibulskis K, Helman E, McKenna A, Shen H, Zack T, Laird PW, Onofrio RC, Winckler W, Weir BA, Beroukhim R, Pellman D, *et al*. Absolute quantification of somatic DNA alterations in human cancer. *Nat Biotechnol* 2012;**30**:413-21.

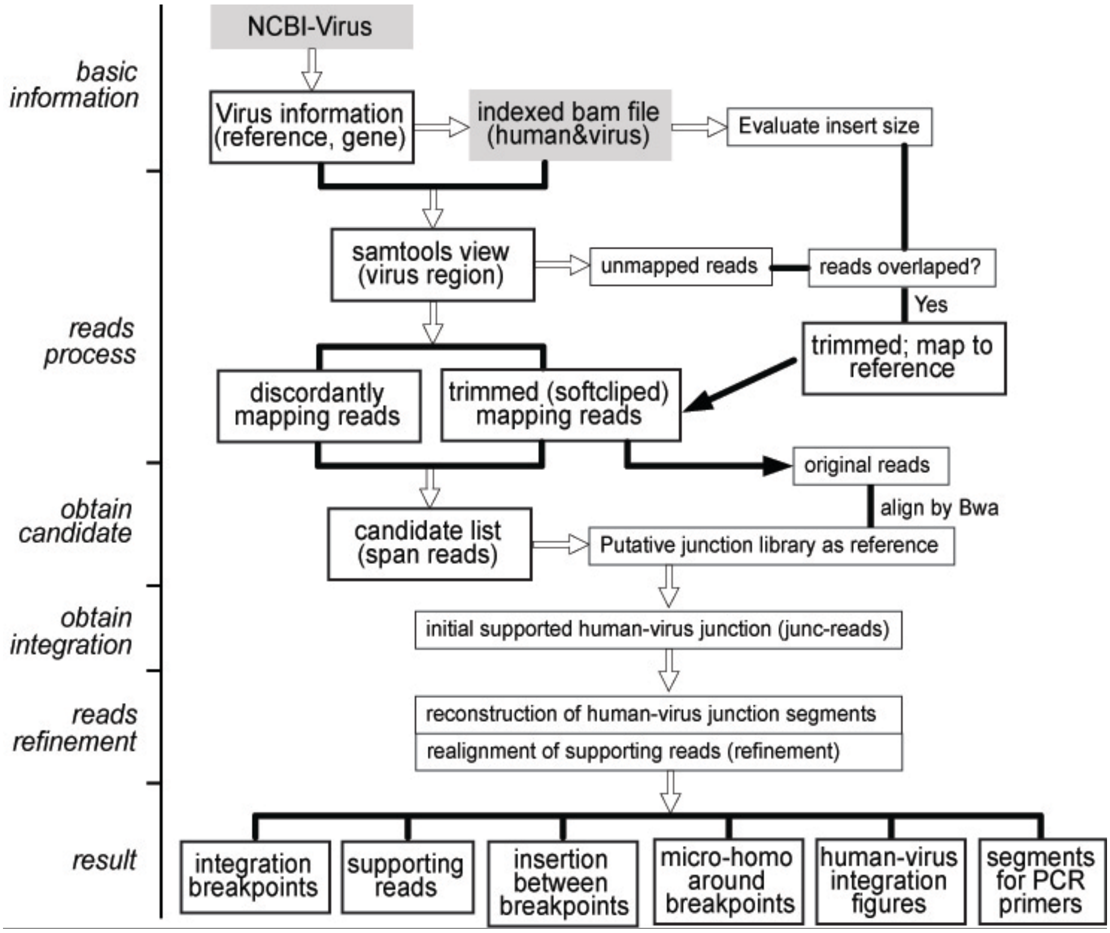

Figure S1. HBV breakpoint detection workflow from sequencing reads in FuseSV. The six parts of VCS data analysis pipeline in FuseSV: basic information set by users; reads are processed and classified from alignment file; obtain the candidate list based on the span-reads; obtain integration via the putative junction library and junc-reads; supporting reads are refined and filtered; generate the result.

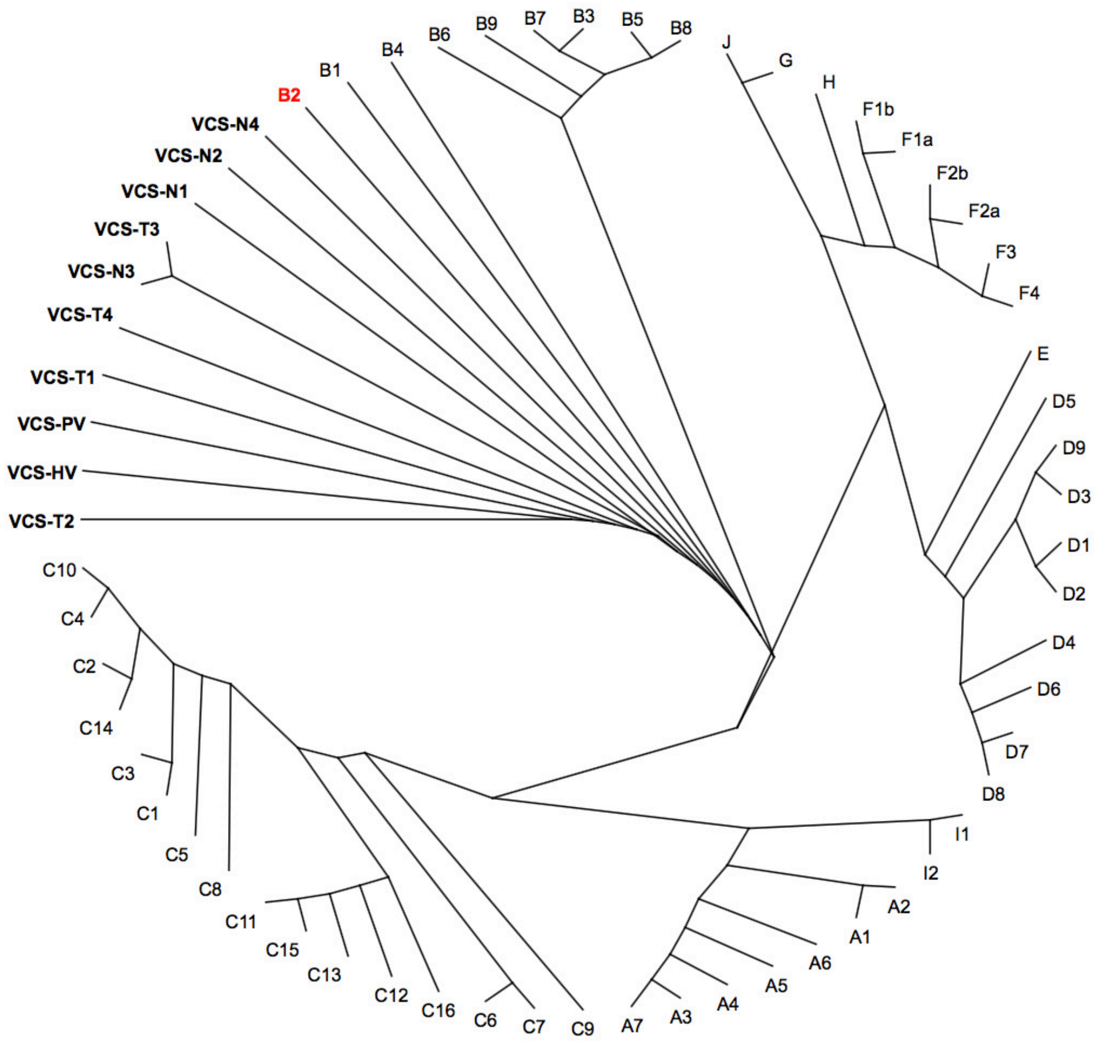

Figure S2. HBV sub-genotype tree in host by VCS analysis. HBV subtypes identified from VCS data of ten tissue samples were classified with B2 subtype.

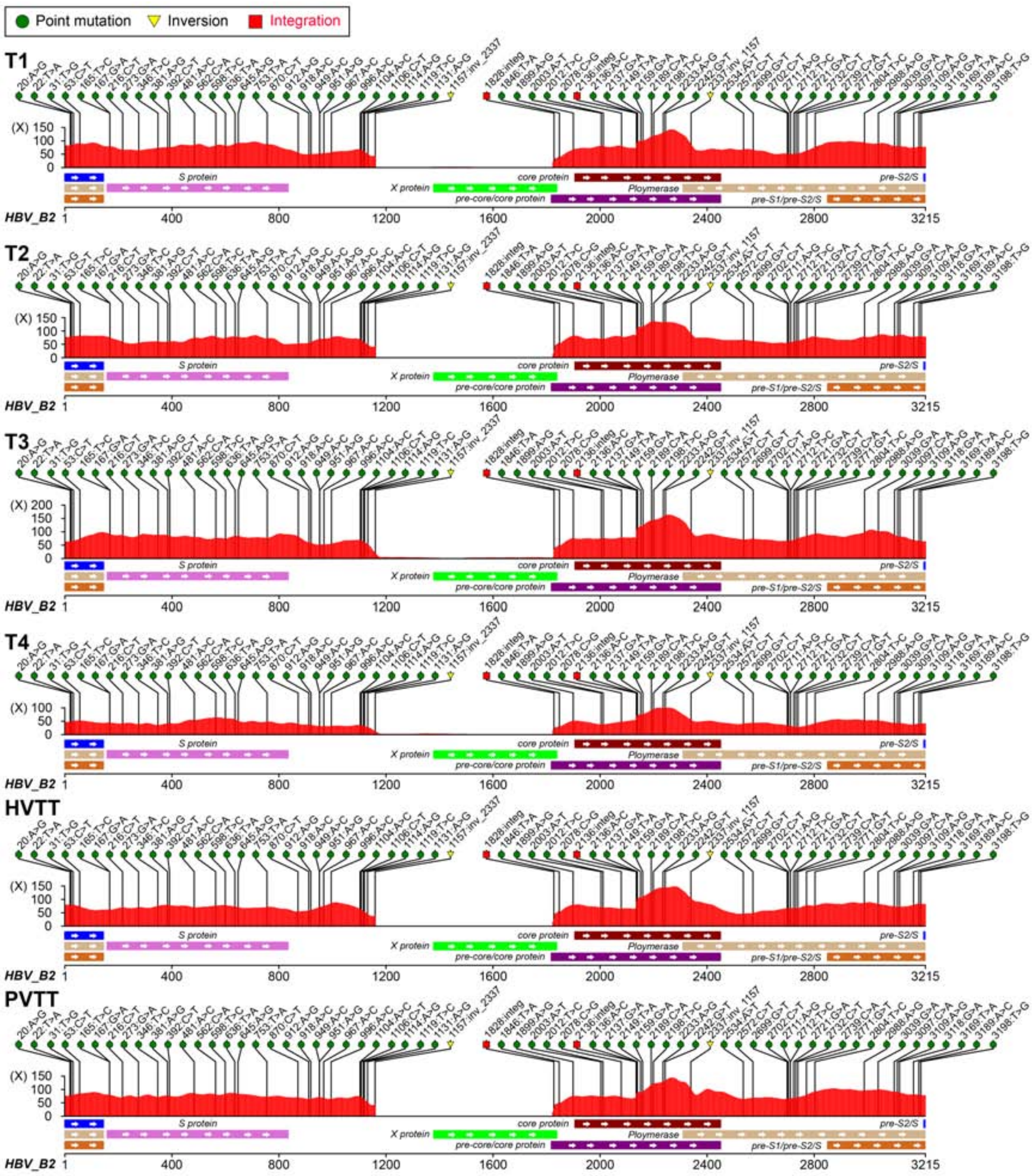

Figure S3. Constructing HBV genomes in tumors by VCS analysis. Depth distribution along HBV genomes were displayed with mutations used to construct individual HBV genomes based on VCS data. The integration breakpoints were also plotted

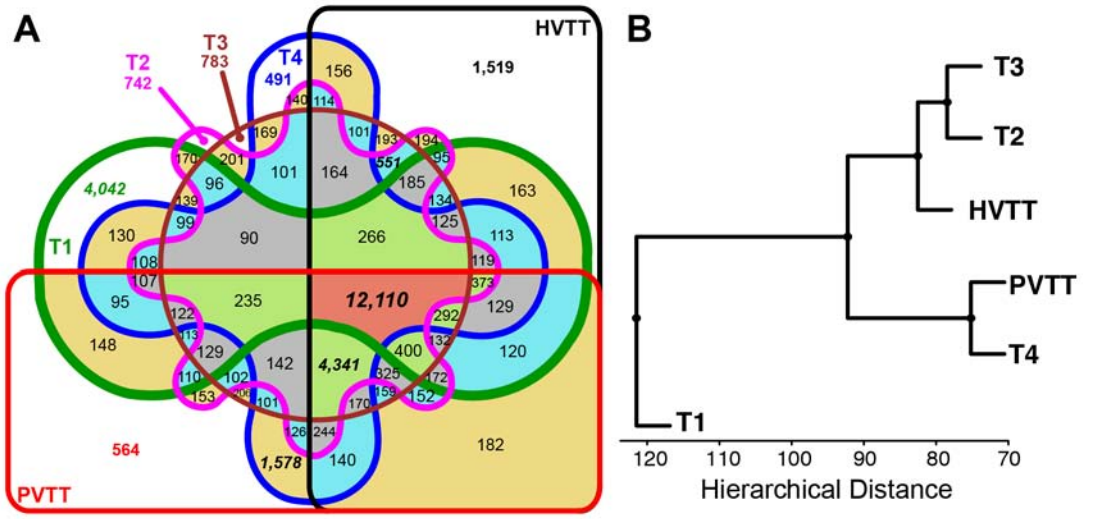

Figure S4. Comparison of genome-wide somatic SNVs among the six tumor specimens. (A) Venn diagram comparing the somatic SNVs among the six tumors. (B) Hierarchical clustering based on somatic SNVs.

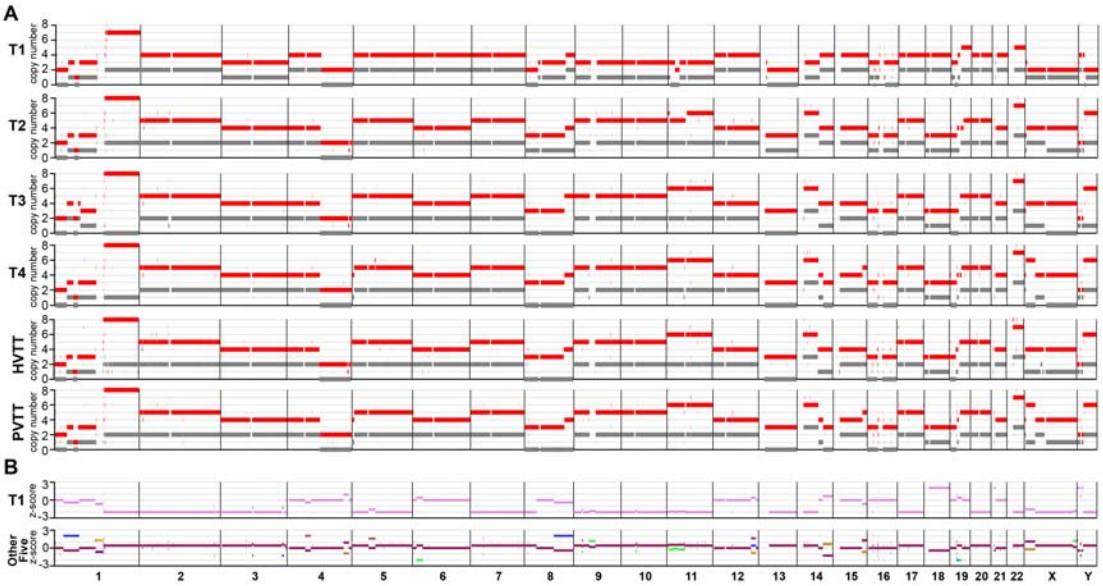

Figure S5. Comparison of genome-wide copy number in six tumor specimens. (A) Copy numbers along the cancer genome of each tumor were drawn. Red line, copy number; grey line, minor copy number. (B) Distribution of copy number z-score along the genome of T1 and the other five tumors.

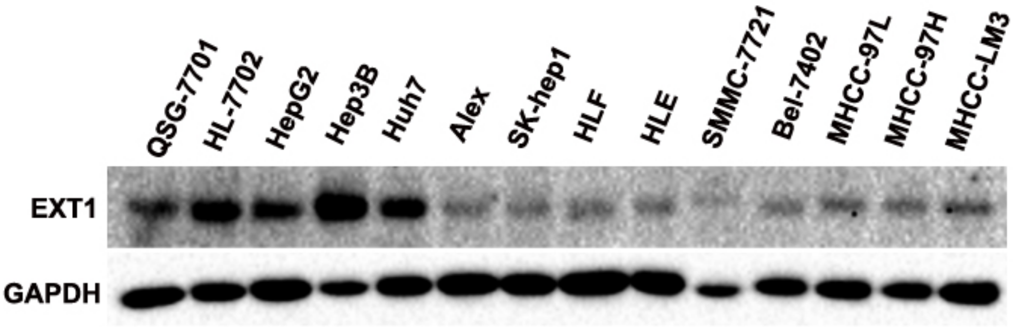

Figure S6. EXT1 expression in hepatic or HCC cell lines by western blot. Among the tested cell lines, QSG-7701, HL-7702, HepG2, and Huh7 showed strong EXT1 protein expression. In contrast, there were lower EXT1 expression in Alex, Sk-hep1, HLF, HLE, SMMC-7721, Bel-7402, MHCC-97L, MHCC-97H, and MHCC-LM3, respectively.

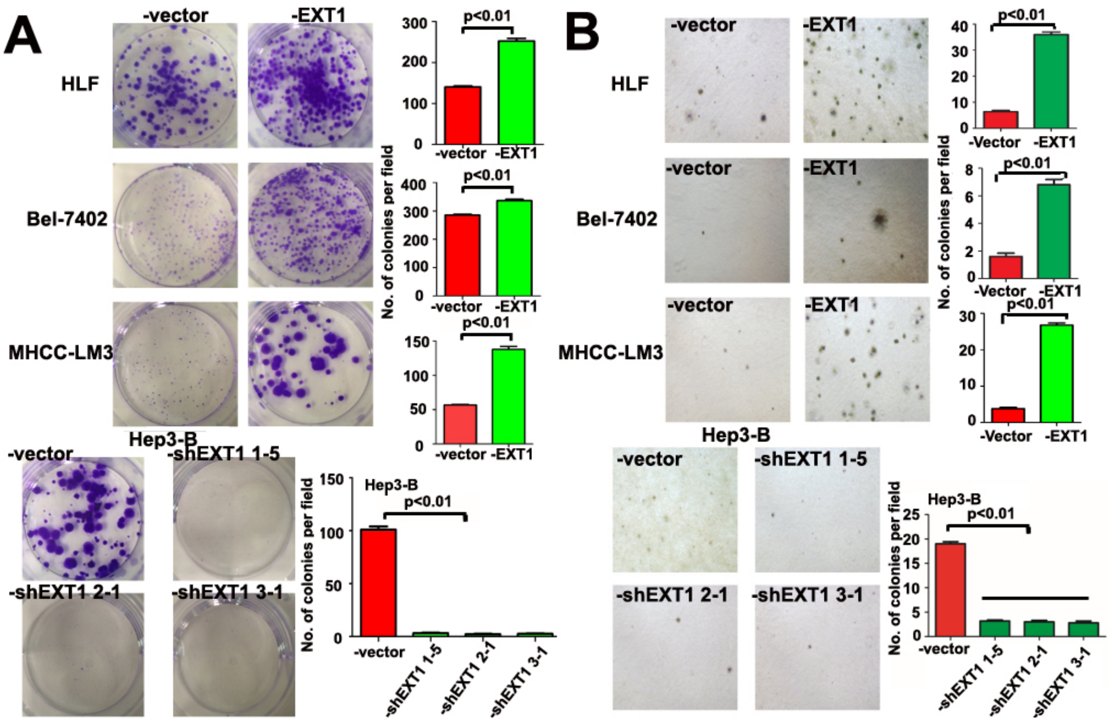

Figure S7. EXT1 affects HCC growth *in vitro*. To evaluate the effects of EXT1 overexpression or knockdown on HCC cell proliferation, colony formation (A) and soft agar clonogenic assays (B) were carried out. HCC cell lines, including HLF, Bel-7402, and MHCC-LM3 were stably overexpressed with EXT1 or control vector. Hep3-B cells were stably knocked down with EXT1. Anchorage-dependent and -independent clonogenic growth was greatly increased, resulting in increased colony size and number in the EXT1 overexpressing cell. In contrast, the clonogenic growth was remarkably reduced in the EXT1 knockdown cells.

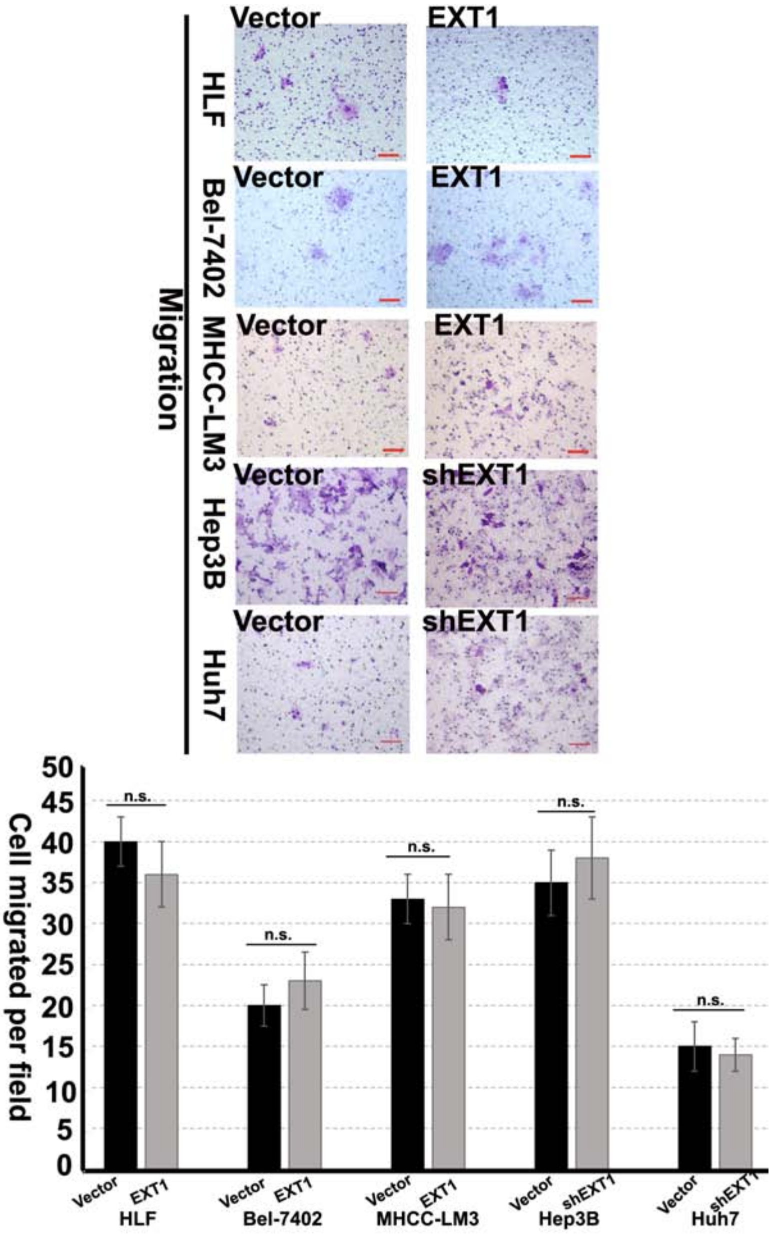

Figure S8. EXT1 does not affect HCC migration *in vitro*. To evaluate the effects of EXT1 overexpression or knockdown on HCC cell migration, HCC cell lines, including HLF, Bel-7402, and MHCC-LM3 were stably overexpressed with EXT1 or control vector. Hep3-B and Huh7 cells were stably knocked down with EXT1. The altered EXT1 expression in these HCC cell lines does not affect cell migration in comparison with their control in transwell assays (n=3). Scale bar, 100 μm. n.s. not significant.

.

Table S1: Clinicopathologic features of the HBV patient with multiple HCC

**Age, y/Sex**	**47/M**
**Etiology**	
Alcohol Abuse/Drug Abuse	-
Mother-to-child Transmission of Hepatitis	-
Blood Transfusion	-
**Virus Infection**	
HBsAg expression	-
HBsAb expression	+
HBcAb expression	+
HBeAb expression	+
HBV-DNA	Undetectable
HCV-Ab expression	-
**Tumor marker, AFP (ng/mL)**	1210
**Pathological Features**	
No. of Tumors	4
Size of Largest Tumor, cm	15cm x 10cm
Histologic Type of Tumors	Trabecular, Solid
Histology of Non-tumor Tissue	Cirrhosis
Tumor Grade	Moderately, Poorly
Intrahepatic Vessels Invasion	+, PVTT
Extrahepatic Vessels Invasion	+, IVCTT
TNM Stage	Stage IV

HCC, hepatocellular carcinoma; HBsAg, hepatitis B virus surface antigen; HBsAb, hepatitis B virus surface antibody; HBcAb, hepatitis B core antibody; HBeAb, hepatitis B e antibody; HCV Ab, hepatitis C virus antibody; AFP, alpha-fetoprotein; PVTT, portal vein tumor thrombosis; IVCTT, inferior vena cava tumor thrombosis. Hepatitis serology testing showed that the patient was HBsAb positive, HBsAg negative, HBcAb positive, HBeAb positive, HCV Ab negative and had no detectable blood HBV DNA copy number, all of which is consistent with the definition of an occult HBV infection.

Table S2. Sequencing data summary and HBV genome details in ten specimens.

1. "Purity" and "Ploidy" was calculated by ABSOLUTE, with N1 sample as the global control.

2. "JR" means "junction reads" supporting relative inversion or integration.

3. "v_inv" refers the inversion on HBV_B2 genome.

4. "-" in table cell means "not detected".

	VCS data summary						WGS data summary									
Sample ID	Read Length(bp)	Clean Reads	Clean Bases (bp)	Q20 percent	Q30 percent	GC percent	Read Length(bp)	Reads Aligned	Uniquely Align rate	Clean Reads	Average depth(X)	Whole Genome Coverage ≥4x	≥10x	≥20x	Tumor Purity	Tumor Ploidy
N1	150;150	45222948	4522294800	96.66%	90.45%	41.24%	150;150	803796250	95.88%	838364050	35.25	99.44%	98.60%	93.61%	-	-
N2	150;150	45087074	4508707400	96.30%	89.58%	44.12%	150;150	825593185	95.85%	861327538	36.02	99.46%	98.63%	93.83%	-	-
N3	150;150	45716730	4571673000	96.46%	90.01%	42.65%	150;150	883277002	95.87%	921285592	38.39	99.49%	98.72%	94.47%	-	-
N4	150;150	40619358	4061935800	96.48%	89.96%	43.24%	150;150	828518430	95.69%	865837242	35.95	99.45%	98.63%	93.84%	-	-
T1	150;150	17346806	1734680600	96.52%	90.09%	42.58%	150;150	1288500681	95.91%	1343497276	55.66	99.63%	99.14%	95.77%	0.39	9.02
T2	150;150	23141900	2314190000	96.45%	89.91%	42.27%	150;150	1471435134	95.73%	1537124646	63.85	99.64%	99.20%	98.27%	0.36	8.08
T3	150;150	50742664	5074266400	96.56%	90.15%	42.81%	150;150	1511571662	95.87%	1576702560	65.66	99.65%	99.26%	98.48%	0.28	4.36
T4	150;150	45576888	4557688800	96.40%	89.73%	42.70%	150;150	1369166476	95.87%	1428156368	59.77	99.63%	99.17%	98.22%	0.33	5.46
HVTT	150;150	53391244	5339124400	96.70%	90.45%	42.30%	150;150	1595783256	95.71%	1667299146	68.42	99.65%	99.25%	98.09%	1.00	9.58
PVTT	150;150	35840538	3584053800	96.56%	90.19%	42.99%	150;150	1565991825	95.72%	1636093152	67.35	99.64%	99.22%	98.08%	0.83	9.87

Major	upstream (5')			downstream (3')			inner	PCR primers		validation (Y/N)									
Integ NO.	segment	strand	bkpos	segment	strand	bkpos	insertion	left	right	N1	N2	N3	N4	T1	T2	T3	T4	HVTT	PVTT
1	chr1	-	34397064	HBV_B2	+	1828	NONE	CAAAGGCTGGGAGAGAATAA	GGAAAGAAGTCAGAAGGCAA	N	N	N	N	Y	Y	Y	Y	Y	Y
2	chr8	-	118557326	HBV_B2	+	2136	NONE	AGGCAATTATGCCAGTTTTC	CAGGAAATGTGAAACCACAA	N	N	Y	N	Y	Y	Y	Y	Y	Y
3	HBV_B2	+	1157	HBV_B2	-	2337	NONE	TTGGTGTCTTTTGGAGTGTG	ACAGGCTTTTACTTTCTCGC	N	N	Y	N	Y	Y	Y	Y	Y	Y

Details of constructed virus genomes from VCS data

Sample	Virus	Average	Mutations				Coverage						major_integ (JR)			minor_integ (Y/N)		
ID	Type	Depth(X)	SUM	SNP	INS	DEL	>=1X	>=4X	>=10X	>=30X	>=50X	>=100X	v_inv	chr1	chr8	chr1	chr8	others
N1	HBV_B2	9.04	49	49	0	0	87.87%	78.76%	32.60%	0.84%	0.00%	0.00%	9	2	5	N	N	N
N2	HBV_B2	11.01	55	55	0	0	94.93%	80.00%	57.73%	1.03%	0.00%	0.00%	13	4	2	N	N	N
N3	HBV_B2	33.33	61	61	0	0	99.47%	89.83%	79.76%	68.53%	9.77%	0.99%	24	24	21	N	N	N
N4	HBV_B2	9.42	48	48	0	0	97.48%	76.33%	42.83%	2.06%	0.00%	0.00%	8	4	7	N	N	N
T1	HBV_B2	78.20	61	61	0	0	83.27%	79.32%	79.32%	79.32%	76.20%	8.64%	51	35	39	N	N	N
T2	HBV_B2	81.07	61	61	0	0	79.35%	79.32%	79.32%	79.32%	78.29%	7.93%	50	52	47	N	N	N
T3	HBV_B2	86.86	61	61	0	0	97.08%	81.71%	79.31%	79.31%	78.42%	10.42%	58	51	50	N	N	N
T4	HBV_B2	47.32	61	61	0	0	89.83%	79.32%	79.32%	77.26%	27.37%	4.51%	35	31	28	N	N	N
HVTT	HBV_B2	87.53	61	61	0	0	79.32%	79.32%	79.32%	79.32%	76.27%	7.62%	46	49	35	N	N	N
PVTT	HBV_B2	93.98	61	61	0	0	79.38%	79.31%	79.31%	79.31%	79.10%	11.07%	53	51	42	N	N	N

Details of constructed virus genomes from WGS data

Sample	Virus	Average	Mutations				Coverage						major_integ (JR)			minor_integ (Y/N)		
ID	Type	Depth(X)	SUM	SNP	INS	DEL	>=1X	>=4X	>=10X	>=30X	>=50X	>=100X	v_inv	chr1	chr8	chr1	chr8	others
N1	-	-	-	-	-	-	-	-	-	-	-	-	-	-	-	-	-	-
N2	-	-	-	-	-	-	-	-	-	-	-	-	-	-	-	-	-	-
N3	HBV_B2	3.27	7	7	0	0	87.47%	1.12%	0.15%	0.00%	0.00%	0.00%	N	N	N	N	N	N
N4	-	-	-	-	-	-	-	-	-	-	-	-	-	-	-	-	-	-
T1	HBV_B2	9.48	53	53	0	0	79.32%	3.01%	5.17%	0.00%	0.00%	0.00%	6	8	4	N	N	N
T2	HBV_B2	11.93	61	61	0	0	79.32%	3.11%	9.79%	0.00%	0.00%	0.00%	5	9	4	N	N	N
T3	HBV_B2	14.72	61	61	0	0	87.71%	2.81%	14.53%	19.97%	0.00%	0.00%	6	15	6	N	N	N
T4	HBV_B2	8.40	43	43	0	0	79.75%	2.97%	2.88%	0.00%	0.00%	0.00%	11	5	3	N	N	N
HVTT	HBV_B2	12.22	61	61	0	0	79.32%	3.10%	11.43%	0.00%	0.00%	0.00%	9	6	8	N	N	N
PVTT	HBV_B2	16.10	61	61	0	0	79.32%	3.11%	18.92%	0.00%	0.00%	0.00%	13	8	4	N	N	N

Table S3. Alterations on HBV_B2 genome in ten specimens.
1. "Fwd" means forward aligned reads that support one alteration.
2. "Rvs" means reversed-complemented aligned reads that support one alteration.
3. "-" means not detected in WGS data.

						details in VCS data								details in WGS data							
Sample	HBV	HBV	Ref	Alt	Alt	Ref_Supports			Alt_Supports			Alt-Shared Samples (VCS)		Ref_Supports			Alt_Supports			Alt-Shared Samples (WGS)	
ID	Type	Position	Allele	Type	Allele	Sum	Fow	Rev	Sum	Fwd	Rev	Count	Details	Sum	Fow	Rev	Sum	Fwd	Rev	Count	Details
N1	HBV_B2	20	A	SNV	G	0	0	0	8	6	2	10	HVTT,N1,N2,N3,N4,PVTT,T1,T2,T3,T4	-	-	-	-	-	-	-	-
N1	HBV_B2	22	T	SNV	A	0	0	0	9	6	3	10	HVTT,N1,N2,N3,N4,PVTT,T1,T2,T3,T4	-	-	-	-	-	-	-	-
N1	HBV_B2	31	T	SNV	G	0	0	0	8	5	3	10	HVTT,N1,N2,N3,N4,PVTT,T1,T2,T3,T4	-	-	-	-	-	-	-	-
N1	HBV_B2	53	C	SNV	T	0	0	0	8	3	5	9	HVTT,N1,N2,N3,PVTT,T1,T2,T3,T4	-	-	-	-	-	-	-	-
N1	HBV_B2	165	T	SNV	C	0	0	0	5	4	1	10	HVTT,N1,N2,N3,N4,PVTT,T1,T2,T3,T4	-	-	-	-	-	-	-	-
N1	HBV_B2	167	G	SNV	A	0	0	0	4	3	1	10	HVTT,N1,N2,N3,N4,PVTT,T1,T2,T3,T4	-	-	-	-	-	-	-	-
N1	HBV_B2	216	C	SNV	T	0	0	0	4	2	2	10	HVTT,N1,N2,N3,N4,PVTT,T1,T2,T3,T4	-	-	-	-	-	-	-	-
N1	HBV_B2	273	G	SNV	A	0	0	0	6	4	2	10	HVTT,N1,N2,N3,N4,PVTT,T1,T2,T3,T4	-	-	-	-	-	-	-	-
N1	HBV_B2	562	C	SNV	A	0	0	0	10	5	5	10	HVTT,N1,N2,N3,N4,PVTT,T1,T2,T3,T4	-	-	-	-	-	-	-	-
N1	HBV_B2	598	T	SNV	C	0	0	0	8	4	4	10	HVTT,N1,N2,N3,N4,PVTT,T1,T2,T3,T4	-	-	-	-	-	-	-	-
N1	HBV_B2	636	T	SNV	A	0	0	0	7	3	4	10	HVTT,N1,N2,N3,N4,PVTT,T1,T2,T3,T4	-	-	-	-	-	-	-	-
N1	HBV_B2	645	A	SNV	G	0	0	0	5	3	2	10	HVTT,N1,N2,N3,N4,PVTT,T1,T2,T3,T4	-	-	-	-	-	-	-	-
N1	HBV_B2	912	A	SNV	G	0	0	0	5	3	2	9	HVTT,N1,N2,N3,PVTT,T1,T2,T3,T4	-	-	-	-	-	-	-	-
N1	HBV_B2	918	A	SNV	C	0	0	0	5	3	2	10	HVTT,N1,N2,N3,N4,PVTT,T1,T2,T3,T4	-	-	-	-	-	-	-	-
N1	HBV_B2	949	A	SNV	C	0	0	0	5	3	2	10	HVTT,N1,N2,N3,N4,PVTT,T1,T2,T3,T4	-	-	-	-	-	-	-	-
N1	HBV_B2	951	A	SNV	G	0	0	0	5	3	2	10	HVTT,N1,N2,N3,N4,PVTT,T1,T2,T3,T4	-	-	-	-	-	-	-	-
N1	HBV_B2	1104	A	SNV	C	0	0	0	6	5	1	9	HVTT,N1,N2,N3,PVTT,T1,T2,T3,T4	-	-	-	-	-	-	-	-
N1	HBV_B2	1106	C	SNV	T	0	0	0	8	7	1	9	HVTT,N1,N2,N3,PVTT,T1,T2,T3,T4	-	-	-	-	-	-	-	-
N1	HBV_B2	1114	A	SNV	G	0	0	0	9	8	1	9	HVTT,N1,N2,N3,PVTT,T1,T2,T3,T4	-	-	-	-	-	-	-	-
N1	HBV_B2	1119	T	SNV	C	0	0	0	9	8	1	9	HVTT,N1,N2,N3,PVTT,T1,T2,T3,T4	-	-	-	-	-	-	-	-
N1	HBV_B2	1131	A	SNV	G	0	0	0	9	8	1	10	HVTT,N1,N2,N3,N4,PVTT,T1,T2,T3,T4	-	-	-	-	-	-	-	-
N1	HBV_B2	1899	A	SNV	G	0	0	0	7	5	2	10	HVTT,N1,N2,N3,N4,PVTT,T1,T2,T3,T4	-	-	-	-	-	-	-	-
N1	HBV_B2	2003	A	SNV	T	0	0	0	6	3	3	10	HVTT,N1,N2,N3,N4,PVTT,T1,T2,T3,T4	-	-	-	-	-	-	-	-
N1	HBV_B2	2012	T	SNV	C	0	0	0	7	5	2	10	HVTT,N1,N2,N3,N4,PVTT,T1,T2,T3,T4	-	-	-	-	-	-	-	-
N1	HBV_B2	2078	C	SNV	G	0	0	0	7	5	2	10	HVTT,N1,N2,N3,N4,PVTT,T1,T2,T3,T4	-	-	-	-	-	-	-	-
N1	HBV_B2	2136	A	SNV	C	0	0	0	17	13	4	10	HVTT,N1,N2,N3,N4,PVTT,T1,T2,T3,T4	-	-	-	-	-	-	-	-
N1	HBV_B2	2137	G	SNV	A	0	0	0	18	13	5	10	HVTT,N1,N2,N3,N4,PVTT,T1,T2,T3,T4	-	-	-	-	-	-	-	-
N1	HBV_B2	2149	T	SNV	A	0	0	0	19	14	5	10	HVTT,N1,N2,N3,N4,PVTT,T1,T2,T3,T4	-	-	-	-	-	-	-	-
N1	HBV_B2	2159	G	SNV	A	0	0	0	19	15	4	10	HVTT,N1,N2,N3,N4,PVTT,T1,T2,T3,T4	-	-	-	-	-	-	-	-
N1	HBV_B2	2189	C	SNV	A	0	0	0	21	13	8	10	HVTT,N1,N2,N3,N4,PVTT,T1,T2,T3,T4	-	-	-	-	-	-	-	-
N1	HBV_B2	2198	T	SNV	C	0	0	0	20	12	8	10	HVTT,N1,N2,N3,N4,PVTT,T1,T2,T3,T4	-	-	-	-	-	-	-	-
N1	HBV_B2	2233	A	SNV	G	0	0	0	25	14	11	10	HVTT,N1,N2,N3,N4,PVTT,T1,T2,T3,T4	-	-	-	-	-	-	-	-
N1	HBV_B2	2242	G	SNV	T	0	0	0	24	12	12	10	HVTT,N1,N2,N3,N4,PVTT,T1,T2,T3,T4	-	-	-	-	-	-	-	-
N1	HBV_B2	2534	A	SNV	T	0	0	0	7	3	4	10	HVTT,N1,N2,N3,N4,PVTT,T1,T2,T3,T4	-	-	-	-	-	-	-	-
N1	HBV_B2	2572	C	SNV	T	0	0	0	8	5	3	8	HVTT,N1,N3,PVTT,T1,T2,T3,T4	-	-	-	-	-	-	-	-
N1	HBV_B2	2699	G	SNV	T	0	0	0	9	5	4	9	HVTT,N1,N2,N3,PVTT,T1,T2,T3,T4	-	-	-	-	-	-	-	-
N1	HBV_B2	2702	C	SNV	T	0	0	0	8	5	3	9	HVTT,N1,N2,N3,PVTT,T1,T2,T3,T4	-	-	-	-	-	-	-	-
N1	HBV_B2	2711	A	SNV	G	0	0	0	9	7	2	9	HVTT,N1,N2,N3,PVTT,T1,T2,T3,T4	-	-	-	-	-	-	-	-
N1	HBV_B2	2712	T	SNV	C	0	0	0	9	7	2	9	HVTT,N1,N2,N3,PVTT,T1,T2,T3,T4	-	-	-	-	-	-	-	-
N1	HBV_B2	2721	G	SNV	A	0	0	0	10	7	3	10	HVTT,N1,N2,N3,N4,PVTT,T1,T2,T3,T4	-	-	-	-	-	-	-	-
N1	HBV_B2	2732	C	SNV	T	0	0	0	9	7	2	9	HVTT,N1,N3,N4,PVTT,T1,T2,T3,T4	-	-	-	-	-	-	-	-
N1	HBV_B2	2739	C	SNV	A	0	0	0	10	8	2	9	HVTT,N1,N3,N4,PVTT,T1,T2,T3,T4	-	-	-	-	-	-	-	-
N1	HBV_B2	2771	G	SNV	T	0	0	0	8	6	2	10	HVTT,N1,N2,N3,N4,PVTT,T1,T2,T3,T4	-	-	-	-	-	-	-	-
N1	HBV_B2	2804	T	SNV	C	0	0	0	12	7	5	10	HVTT,N1,N2,N3,N4,PVTT,T1,T2,T3,T4	-	-	-	-	-	-	-	-
N1	HBV_B2	2988	A	SNV	G	0	0	0	10	6	4	10	HVTT,N1,N2,N3,N4,PVTT,T1,T2,T3,T4	-	-	-	-	-	-	-	-
N1	HBV_B2	3039	G	SNV	A	0	0	0	10	3	7	10	HVTT,N1,N2,N3,N4,PVTT,T1,T2,T3,T4	-	-	-	-	-	-	-	-
N1	HBV_B2	3118	G	SNV	A	0	0	0	6	4	2	10	HVTT,N1,N2,N3,N4,PVTT,T1,T2,T3,T4	-	-	-	-	-	-	-	-
N1	HBV_B2	3169	T	SNV	A	0	0	0	9	6	3	10	HVTT,N1,N2,N3,N4,PVTT,T1,T2,T3,T4	-	-	-	-	-	-	-	-
N1	HBV_B2	3198	T	SNV	G	0	0	0	7	5	2	10	HVTT,N1,N2,N3,N4,PVTT,T1,T2,T3,T4	-	-	-	-	-	-	-	-
N2	HBV_B2	20	A	SNV	G	0	0	0	13	7	6	10	HVTT,N1,N2,N3,N4,PVTT,T1,T2,T3,T4	-	-	-	-	-	-	-	-
N2	HBV_B2	22	T	SNV	A	0	0	0	14	8	6	10	HVTT,N1,N2,N3,N4,PVTT,T1,T2,T3,T4	-	-	-	-	-	-	-	-
N2	HBV_B2	31	T	SNV	G	6	4	2	7	4	3	10	HVTT,N1,N2,N3,N4,PVTT,T1,T2,T3,T4	-	-	-	-	-	-	-	-
N2	HBV_B2	53	C	SNV	T	0	0	0	17	11	6	9	HVTT,N1,N2,N3,PVTT,T1,T2,T3,T4	-	-	-	-	-	-	-	-
N2	HBV_B2	165	T	SNV	C	0	0	0	9	2	7	10	HVTT,N1,N2,N3,N4,PVTT,T1,T2,T3,T4	-	-	-	-	-	-	-	-
N2	HBV_B2	167	G	SNV	A	0	0	0	10	3	7	10	HVTT,N1,N2,N3,N4,PVTT,T1,T2,T3,T4	-	-	-	-	-	-	-	-
N2	HBV_B2	216	C	SNV	T	0	0	0	9	6	3	10	HVTT,N1,N2,N3,N4,PVTT,T1,T2,T3,T4	-	-	-	-	-	-	-	-
N2	HBV_B2	273	G	SNV	A	0	0	0	10	8	2	10	HVTT,N1,N2,N3,N4,PVTT,T1,T2,T3,T4	-	-	-	-	-	-	-	-
N2	HBV_B2	346	T	SNV	C	0	0	0	10	4	6	9	HVTT,N2,N3,N4,PVTT,T1,T2,T3,T4	-	-	-	-	-	-	-	-
N2	HBV_B2	381	A	SNV	G	0	0	0	7	2	5	9	HVTT,N2,N3,N4,PVTT,T1,T2,T3,T4	-	-	-	-	-	-	-	-
N2	HBV_B2	392	C	SNV	T	0	0	0	6	3	3	9	HVTT,N2,N3,N4,PVTT,T1,T2,T3,T4	-	-	-	-	-	-	-	-
N2	HBV_B2	481	A	SNV	C	0	0	0	11	9	2	9	HVTT,N2,N3,N4,PVTT,T1,T2,T3,T4	-	-	-	-	-	-	-	-
N2	HBV_B2	562	C	SNV	A	0	0	0	10	4	6	10	HVTT,N1,N2,N3,N4,PVTT,T1,T2,T3,T4	-	-	-	-	-	-	-	-
N2	HBV_B2	598	T	SNV	C	0	0	0	10	7	3	10	HVTT,N1,N2,N3,N4,PVTT,T1,T2,T3,T4	-	-	-	-	-	-	-	-
N2	HBV_B2	636	T	SNV	A	0	0	0	9	7	2	10	HVTT,N1,N2,N3,N4,PVTT,T1,T2,T3,T4	-	-	-	-	-	-	-	-
N2	HBV_B2	645	A	SNV	G	0	0	0	8	6	2	10	HVTT,N1,N2,N3,N4,PVTT,T1,T2,T3,T4	-	-	-	-	-	-	-	-
N2	HBV_B2	912	A	SNV	G	0	0	0	6	4	2	9	HVTT,N1,N2,N3,PVTT,T1,T2,T3,T4	-	-	-	-	-	-	-	-
N2	HBV_B2	918	A	SNV	C	0	0	0	6	5	1	10	HVTT,N1,N2,N3,N4,PVTT,T1,T2,T3,T4	-	-	-	-	-	-	-	-
N2	HBV_B2	949	A	SNV	C	0	0	0	7	5	2	10	HVTT,N1,N2,N3,N4,PVTT,T1,T2,T3,T4	-	-	-	-	-	-	-	-
N2	HBV_B2	951	A	SNV	G	0	0	0	5	4	1	10	HVTT,N1,N2,N3,N4,PVTT,T1,T2,T3,T4	-	-	-	-	-	-	-	-
N2	HBV_B2	967	A	SNV	C	0	0	0	7	6	1	9	HVTT,N2,N3,N4,PVTT,T1,T2,T3,T4	-	-	-	-	-	-	-	-
N2	HBV_B2	996	A	SNV	C	0	0	0	7	4	3	9	HVTT,N2,N3,N4,PVTT,T1,T2,T3,T4	-	-	-	-	-	-	-	-
N2	HBV_B2	1104	A	SNV	C	0	0	0	11	8	3	9	HVTT,N1,N2,N3,PVTT,T1,T2,T3,T4	-	-	-	-	-	-	-	-
N2	HBV_B2	1106	C	SNV	T	0	0	0	12	9	3	9	HVTT,N1,N2,N3,PVTT,T1,T2,T3,T4	-	-	-	-	-	-	-	-
N2	HBV_B2	1114	A	SNV	G	0	0	0	12	9	3	9	HVTT,N1,N2,N3,PVTT,T1,T2,T3,T4	-	-	-	-	-	-	-	-
N2	HBV_B2	1119	T	SNV	C	0	0	0	13	9	4	9	HVTT,N1,N2,N3,PVTT,T1,T2,T3,T4	-	-	-	-	-	-	-	-
N2	HBV_B2	1131	A	SNV	G	0	0	0	13	9	4	10	HVTT,N1,N2,N3,N4,PVTT,T1,T2,T3,T4	-	-	-	-	-	-	-	-
N2	HBV_B2	1899	A	SNV	G	0	0	0	13	10	3	10	HVTT,N1,N2,N3,N4,PVTT,T1,T2,T3,T4	-	-	-	-	-	-	-	-
N2	HBV_B2	2003	A	SNV	T	0	0	0	14	9	5	10	HVTT,N1,N2,N3,N4,PVTT,T1,T2,T3,T4	-	-	-	-	-	-	-	-
N2	HBV_B2	2012	T	SNV	C	0	0	0	16	12	4	10	HVTT,N1,N2,N3,N4,PVTT,T1,T2,T3,T4	-	-	-	-	-	-	-	-
N2	HBV_B2	2078	C	SNV	G	0	0	0	16	9	7	10	HVTT,N1,N2,N3,N4,PVTT,T1,T2,T3,T4	-	-	-	-	-	-	-	-
N2	HBV_B2	2136	A	SNV	C	0	0	0	18	9	9	10	HVTT,N1,N2,N3,N4,PVTT,T1,T2,T3,T4	-	-	-	-	-	-	-	-
N2	HBV_B2	2137	G	SNV	A	0	0	0	18	7	11	10	HVTT,N1,N2,N3,N4,PVTT,T1,T2,T3,T4	-	-	-	-	-	-	-	-
N2	HBV_B2	2149	T	SNV	A	0	0	0	19	10	9	10	HVTT,N1,N2,N3,N4,PVTT,T1,T2,T3,T4	-	-	-	-	-	-	-	-
N2	HBV_B2	2159	G	SNV	A	0	0	0	19	10	9	10	HVTT,N1,N2,N3,N4,PVTT,T1,T2,T3,T4	-	-	-	-	-	-	-	-
N2	HBV_B2	2189	C	SNV	A	0	0	0	17	11	6	10	HVTT,N1,N2,N3,N4,PVTT,T1,T2,T3,T4	-	-	-	-	-	-	-	-
N2	HBV_B2	2198	T	SNV	C	0	0	0	17	11	6	10	HVTT,N1,N2,N3,N4,PVTT,T1,T2,T3,T4	-	-	-	-	-	-	-	-
N2	HBV_B2	2233	A	SNV	G	0	0	0	19	13	6	10	HVTT,N1,N2,N3,N4,PVTT,T1,T2,T3,T4	-	-	-	-	-	-	-	-
N2	HBV_B2	2242	G	SNV	T	0	0	0	20	14	6	10	HVTT,N1,N2,N3,N4,PVTT,T1,T2,T3,T4	-	-	-	-	-	-	-	-
N2	HBV_B2	2534	A	SNV	T	0	0	0	5	3	2	10	HVTT,N1,N2,N3,N4,PVTT,T1,T2,T3,T4	-	-	-	-	-	-	-	-
N2	HBV_B2	2699	G	SNV	T	0	0	0	19	3	16	9	HVTT,N1,N2,N3,PVTT,T1,T2,T3,T4	-	-	-	-	-	-	-	-

N2	HBV_B2	2702	C	SNV	T	0	0	0	20	3	17	9	HVTT,N1,N2,N3,PVTT,T1,T2,T3,T4	-	-	-	-	-	-	-	-
N2	HBV_B2	2711	A	SNV	G	0	0	0	22	5	17	9	HVTT,N1,N2,N3,PVTT,T1,T2,T3,T4	-	-	-	-	-	-	-	-
N2	HBV_B2	2712	T	SNV	C	0	0	0	22	5	17	9	HVTT,N1,N2,N3,PVTT,T1,T2,T3,T4	-	-	-	-	-	-	-	-
N2	HBV_B2	2721	G	SNV	A	0	0	0	23	6	17	10	HVTT,N1,N2,N3,N4,PVTT,T1,T2,T3,T4	-	-	-	-	-	-	-	-
N2	HBV_B2	2771	G	SNV	T	0	0	0	7	7	0	10	HVTT,N1,N2,N3,N4,PVTT,T1,T2,T3,T4	-	-	-	-	-	-	-	-
N2	HBV_B2	2804	T	SNV	C	0	0	0	12	9	3	10	HVTT,N1,N2,N3,N4,PVTT,T1,T2,T3,T4	-	-	-	-	-	-	-	-
N2	HBV_B2	2988	A	SNV	G	0	0	0	7	5	2	10	HVTT,N1,N2,N3,N4,PVTT,T1,T2,T3,T4	-	-	-	-	-	-	-	-
N2	HBV_B2	3039	G	SNV	A	0	0	0	7	4	3	10	HVTT,N1,N2,N3,N4,PVTT,T1,T2,T3,T4	-	-	-	-	-	-	-	-
N2	HBV_B2	3097	C	SNV	A	0	0	0	9	6	3	9	HVTT,N2,N3,N4,PVTT,T1,T2,T3,T4	-	-	-	-	-	-	-	-
N2	HBV_B2	3109	A	SNV	G	0	0	0	10	6	4	9	HVTT,N2,N3,N4,PVTT,T1,T2,T3,T4	-	-	-	-	-	-	-	-
N2	HBV_B2	3118	G	SNV	A	0	0	0	10	6	4	10	HVTT,N1,N2,N3,N4,PVTT,T1,T2,T3,T4	-	-	-	-	-	-	-	-
N2	HBV_B2	3169	T	SNV	A	0	0	0	10	4	6	10	HVTT,N1,N2,N3,N4,PVTT,T1,T2,T3,T4	-	-	-	-	-	-	-	-
N2	HBV_B2	3189	A	SNV	C	0	0	0	10	4	6	9	HVTT,N2,N3,N4,PVTT,T1,T2,T3,T4	-	-	-	-	-	-	-	-
N2	HBV_B2	3198	T	SNV	G	0	0	0	12	6	6	10	HVTT,N1,N2,N3,N4,PVTT,T1,T2,T3,T4	-	-	-	-	-	-	-	-
N3	HBV_B2	20	A	SNV	G	0	0	0	44	23	21	10	HVTT,N1,N2,N3,N4,PVTT,T1,T2,T3,T4	-	-	-	-	-	-	-	-
N3	HBV_B2	22	T	SNV	A	0	0	0	43	24	19	10	HVTT,N1,N2,N3,N4,PVTT,T1,T2,T3,T4	-	-	-	-	-	-	-	-
N3	HBV_B2	31	T	SNV	G	0	0	0	45	24	21	10	HVTT,N1,N2,N3,N4,PVTT,T1,T2,T3,T4	-	-	-	-	-	-	-	-
N3	HBV_B2	53	C	SNV	T	0	0	0	40	21	19	9	HVTT,N1,N2,N3,PVTT,T1,T2,T3,T4	-	-	-	-	-	-	-	-
N3	HBV_B2	165	T	SNV	C	0	0	0	33	16	17	10	HVTT,N1,N2,N3,N4,PVTT,T1,T2,T3,T4	-	-	-	-	-	-	-	-
N3	HBV_B2	167	G	SNV	A	0	0	0	33	16	17	10	HVTT,N1,N2,N3,N4,PVTT,T1,T2,T3,T4	-	-	-	-	-	-	-	-
N3	HBV_B2	216	C	SNV	T	0	0	0	23	14	9	10	HVTT,N1,N2,N3,N4,PVTT,T1,T2,T3,T4	-	-	-	-	-	-	-	-
N3	HBV_B2	273	G	SNV	A	0	0	0	26	12	14	10	HVTT,N1,N2,N3,N4,PVTT,T1,T2,T3,T4	-	-	-	-	-	-	-	-
N3	HBV_B2	346	T	SNV	C	0	0	0	28	17	11	9	HVTT,N2,N3,N4,PVTT,T1,T2,T3,T4	-	-	-	-	-	-	-	-
N3	HBV_B2	381	A	SNV	G	0	0	0	29	17	12	9	HVTT,N2,N3,N4,PVTT,T1,T2,T3,T4	-	-	-	-	-	-	-	-
N3	HBV_B2	392	C	SNV	T	0	0	0	28	16	12	9	HVTT,N2,N3,N4,PVTT,T1,T2,T3,T4	-	-	-	-	-	-	-	-
N3	HBV_B2	481	A	SNV	C	0	0	0	39	27	12	9	HVTT,N2,N3,N4,PVTT,T1,T2,T3,T4	0	0	0	5	2	3	7	HVTT,N3,PVTT,T1,T2,T3,T4
N3	HBV_B2	562	C	SNV	A	0	0	0	40	19	21	10	HVTT,N1,N2,N3,N4,PVTT,T1,T2,T3,T4	-	-	-	-	-	-	-	-
N3	HBV_B2	598	T	SNV	C	0	0	0	32	18	14	10	HVTT,N1,N2,N3,N4,PVTT,T1,T2,T3,T4	-	-	-	-	-	-	-	-
N3	HBV_B2	636	T	SNV	A	0	0	0	27	14	13	10	HVTT,N1,N2,N3,N4,PVTT,T1,T2,T3,T4	-	-	-	-	-	-	-	-
N3	HBV_B2	645	A	SNV	G	0	0	0	27	14	13	10	HVTT,N1,N2,N3,N4,PVTT,T1,T2,T3,T4	-	-	-	-	-	-	-	-
N3	HBV_B2	753	T	SNV	A	0	0	0	30	22	8	7	HVTT,N3,PVTT,T1,T2,T3,T4	-	-	-	-	-	-	-	-
N3	HBV_B2	870	C	SNV	T	0	0	0	18	6	12	7	HVTT,N3,PVTT,T1,T2,T3,T4	-	-	-	-	-	-	-	-
N3	HBV_B2	912	A	SNV	G	0	0	0	15	9	6	9	HVTT,N1,N2,N3,PVTT,T1,T2,T3,T4	-	-	-	-	-	-	-	-
N3	HBV_B2	918	A	SNV	C	0	0	0	17	11	6	10	HVTT,N1,N2,N3,N4,PVTT,T1,T2,T3,T4	-	-	-	-	-	-	-	-
N3	HBV_B2	949	A	SNV	C	0	0	0	21	17	4	10	HVTT,N1,N2,N3,N4,PVTT,T1,T2,T3,T4	-	-	-	-	-	-	-	-
N3	HBV_B2	951	A	SNV	G	0	0	0	22	19	3	10	HVTT,N1,N2,N3,N4,PVTT,T1,T2,T3,T4	-	-	-	-	-	-	-	-
N3	HBV_B2	967	A	SNV	C	0	0	0	24	19	5	9	HVTT,N2,N3,N4,PVTT,T1,T2,T3,T4	-	-	-	-	-	-	-	-
N3	HBV_B2	996	A	SNV	C	0	0	0	26	18	8	9	HVTT,N2,N3,N4,PVTT,T1,T2,T3,T4	-	-	-	-	-	-	-	-
N3	HBV_B2	1104	A	SNV	C	0	0	0	34	21	13	9	HVTT,N1,N2,N3,PVTT,T1,T2,T3,T4	-	-	-	-	-	-	-	-
N3	HBV_B2	1106	C	SNV	T	0	0	0	34	22	12	9	HVTT,N1,N2,N3,PVTT,T1,T2,T3,T4	-	-	-	-	-	-	-	-
N3	HBV_B2	1114	A	SNV	G	0	0	0	54	43	11	9	HVTT,N1,N2,N3,PVTT,T1,T2,T3,T4	-	-	-	-	-	-	-	-
N3	HBV_B2	1119	T	SNV	C	0	0	0	54	42	12	9	HVTT,N1,N2,N3,PVTT,T1,T2,T3,T4	-	-	-	-	-	-	-	-
N3	HBV_B2	1131	A	SNV	G	0	0	0	72	60	12	10	HVTT,N1,N2,N3,N4,PVTT,T1,T2,T3,T4	-	-	-	-	-	-	-	-
N3	HBV_B2	1846	T	SNV	A	0	0	0	37	24	13	8	HVTT,N3,N4,PVTT,T1,T2,T3,T4	-	-	-	-	-	-	-	-
N3	HBV_B2	1899	A	SNV	G	0	0	0	38	21	17	10	HVTT,N1,N2,N3,N4,PVTT,T1,T2,T3,T4	-	-	-	-	-	-	-	-
N3	HBV_B2	2003	A	SNV	T	0	0	0	33	20	13	10	HVTT,N1,N2,N3,N4,PVTT,T1,T2,T3,T4	-	-	-	-	-	-	-	-
N3	HBV_B2	2012	T	SNV	C	0	0	0	35	23	12	10	HVTT,N1,N2,N3,N4,PVTT,T1,T2,T3,T4	-	-	-	-	-	-	-	-
N3	HBV_B2	2078	C	SNV	G	0	0	0	36	22	14	10	HVTT,N1,N2,N3,N4,PVTT,T1,T2,T3,T4	-	-	-	-	-	-	-	-
N3	HBV_B2	2136	A	SNV	C	0	0	0	56	29	27	10	HVTT,N1,N2,N3,N4,PVTT,T1,T2,T3,T4	-	-	-	-	-	-	-	-
N3	HBV_B2	2137	G	SNV	A	0	0	0	57	30	27	10	HVTT,N1,N2,N3,N4,PVTT,T1,T2,T3,T4	-	-	-	-	-	-	-	-
N3	HBV_B2	2149	T	SNV	A	0	0	0	61	32	29	10	HVTT,N1,N2,N3,N4,PVTT,T1,T2,T3,T4	0	0	0	6	4	2	7	HVTT,N3,PVTT,T1,T2,T3,T4
N3	HBV_B2	2159	G	SNV	A	0	0	0	62	32	30	10	HVTT,N1,N2,N3,N4,PVTT,T1,T2,T3,T4	0	0	0	8	5	3	7	HVTT,N3,PVTT,T1,T2,T3,T4
N3	HBV_B2	2189	C	SNV	A	0	0	0	55	30	25	10	HVTT,N1,N2,N3,N4,PVTT,T1,T2,T3,T4	0	0	0	10	6	4	7	HVTT,N3,PVTT,T1,T2,T3,T4
N3	HBV_B2	2198	T	SNV	C	0	0	0	54	33	21	10	HVTT,N1,N2,N3,N4,PVTT,T1,T2,T3,T4	0	0	0	10	6	4	7	HVTT,N3,PVTT,T1,T2,T3,T4
N3	HBV_B2	2233	A	SNV	G	0	0	0	51	30	21	10	HVTT,N1,N2,N3,N4,PVTT,T1,T2,T3,T4	0	0	0	12	7	5	7	HVTT,N3,PVTT,T1,T2,T3,T4
N3	HBV_B2	2242	G	SNV	T	0	0	0	50	26	24	10	HVTT,N1,N2,N3,N4,PVTT,T1,T2,T3,T4	0	0	0	12	6	6	7	HVTT,N3,PVTT,T1,T2,T3,T4
N3	HBV_B2	2534	A	SNV	T	0	0	0	34	20	14	10	HVTT,N1,N2,N3,N4,PVTT,T1,T2,T3,T4	-	-	-	-	-	-	-	-
N3	HBV_B2	2572	C	SNV	T	0	0	0	30	18	12	8	HVTT,N1,N3,PVTT,T1,T2,T3,T4	-	-	-	-	-	-	-	-
N3	HBV_B2	2699	G	SNV	T	0	0	0	23	16	7	9	HVTT,N1,N2,N3,PVTT,T1,T2,T3,T4	-	-	-	-	-	-	-	-
N3	HBV_B2	2702	C	SNV	T	0	0	0	23	16	7	9	HVTT,N1,N2,N3,PVTT,T1,T2,T3,T4	-	-	-	-	-	-	-	-
N3	HBV_B2	2711	A	SNV	G	0	0	0	26	18	8	9	HVTT,N1,N2,N3,PVTT,T1,T2,T3,T4	-	-	-	-	-	-	-	-
N3	HBV_B2	2712	T	SNV	C	0	0	0	26	18	8	9	HVTT,N1,N2,N3,PVTT,T1,T2,T3,T4	-	-	-	-	-	-	-	-
N3	HBV_B2	2721	G	SNV	A	0	0	0	25	20	5	10	HVTT,N1,N2,N3,N4,PVTT,T1,T2,T3,T4	-	-	-	-	-	-	-	-
N3	HBV_B2	2732	C	SNV	T	0	0	0	25	21	4	9	HVTT,N1,N3,N4,PVTT,T1,T2,T3,T4	-	-	-	-	-	-	-	-
N3	HBV_B2	2739	C	SNV	A	0	0	0	25	21	4	9	HVTT,N1,N3,N4,PVTT,T1,T2,T3,T4	-	-	-	-	-	-	-	-
N3	HBV_B2	2771	G	SNV	T	0	0	0	31	26	5	10	HVTT,N1,N2,N3,N4,PVTT,T1,T2,T3,T4	-	-	-	-	-	-	-	-
N3	HBV_B2	2804	T	SNV	C	0	0	0	40	24	16	10	HVTT,N1,N2,N3,N4,PVTT,T1,T2,T3,T4	-	-	-	-	-	-	-	-
N3	HBV_B2	2988	A	SNV	G	0	0	0	31	17	14	10	HVTT,N1,N2,N3,N4,PVTT,T1,T2,T3,T4	-	-	-	-	-	-	-	-
N3	HBV_B2	3039	G	SNV	A	0	0	0	33	19	14	10	HVTT,N1,N2,N3,N4,PVTT,T1,T2,T3,T4	-	-	-	-	-	-	-	-
N3	HBV_B2	3097	C	SNV	A	0	0	0	34	24	10	9	HVTT,N2,N3,N4,PVTT,T1,T2,T3,T4	-	-	-	-	-	-	-	-
N3	HBV_B2	3109	A	SNV	G	0	0	0	31	20	11	9	HVTT,N2,N3,N4,PVTT,T1,T2,T3,T4	-	-	-	-	-	-	-	-
N3	HBV_B2	3118	G	SNV	A	0	0	0	34	20	14	10	HVTT,N1,N2,N3,N4,PVTT,T1,T2,T3,T4	-	-	-	-	-	-	-	-
N3	HBV_B2	3169	T	SNV	A	0	0	0	39	22	17	10	HVTT,N1,N2,N3,N4,PVTT,T1,T2,T3,T4	-	-	-	-	-	-	-	-
N3	HBV_B2	3189	A	SNV	C	0	0	0	34	20	14	9	HVTT,N2,N3,N4,PVTT,T1,T2,T3,T4	-	-	-	-	-	-	-	-
N3	HBV_B2	3198	T	SNV	G	0	0	0	34	18	16	10	HVTT,N1,N2,N3,N4,PVTT,T1,T2,T3,T4	-	-	-	-	-	-	-	-
N4	HBV_B2	20	A	SNV	G	0	0	0	5	2	3	10	HVTT,N1,N2,N3,N4,PVTT,T1,T2,T3,T4	-	-	-	-	-	-	-	-
N4	HBV_B2	22	T	SNV	A	0	0	0	5	2	3	10	HVTT,N1,N2,N3,N4,PVTT,T1,T2,T3,T4	-	-	-	-	-	-	-	-
N4	HBV_B2	31	T	SNV	G	0	0	0	6	3	3	10	HVTT,N1,N2,N3,N4,PVTT,T1,T2,T3,T4	-	-	-	-	-	-	-	-
N4	HBV_B2	165	T	SNV	C	0	0	0	12	8	4	10	HVTT,N1,N2,N3,N4,PVTT,T1,T2,T3,T4	-	-	-	-	-	-	-	-
N4	HBV_B2	167	G	SNV	A	0	0	0	12	8	4	10	HVTT,N1,N2,N3,N4,PVTT,T1,T2,T3,T4	-	-	-	-	-	-	-	-
N4	HBV_B2	216	C	SNV	T	0	0	0	12	6	6	10	HVTT,N1,N2,N3,N4,PVTT,T1,T2,T3,T4	-	-	-	-	-	-	-	-
N4	HBV_B2	273	G	SNV	A	0	0	0	12	6	6	10	HVTT,N1,N2,N3,N4,PVTT,T1,T2,T3,T4	-	-	-	-	-	-	-	-
N4	HBV_B2	346	T	SNV	C	0	0	0	10	6	4	9	HVTT,N2,N3,N4,PVTT,T1,T2,T3,T4	-	-	-	-	-	-	-	-
N4	HBV_B2	381	A	SNV	G	0	0	0	9	4	5	9	HVTT,N2,N3,N4,PVTT,T1,T2,T3,T4	-	-	-	-	-	-	-	-
N4	HBV_B2	392	C	SNV	T	0	0	0	9	5	4	9	HVTT,N2,N3,N4,PVTT,T1,T2,T3,T4	-	-	-	-	-	-	-	-
N4	HBV_B2	481	A	SNV	C	0	0	0	11	7	4	9	HVTT,N2,N3,N4,PVTT,T1,T2,T3,T4	-	-	-	-	-	-	-	-
N4	HBV_B2	562	C	SNV	A	0	0	0	10	6	4	10	HVTT,N1,N2,N3,N4,PVTT,T1,T2,T3,T4	-	-	-	-	-	-	-	-
N4	HBV_B2	598	T	SNV	C	0	0	0	7	5	2	10	HVTT,N1,N2,N3,N4,PVTT,T1,T2,T3,T4	-	-	-	-	-	-	-	-
N4	HBV_B2	636	T	SNV	A	0	0	0	7	5	2	10	HVTT,N1,N2,N3,N4,PVTT,T1,T2,T3,T4	-	-	-	-	-	-	-	-
N4	HBV_B2	645	A	SNV	G	0	0	0	6	3	3	10	HVTT,N1,N2,N3,N4,PVTT,T1,T2,T3,T4	-	-	-	-	-	-	-	-
N4	HBV_B2	918	A	SNV	C	0	0	0	7	5	2	10	HVTT,N1,N2,N3,N4,PVTT,T1,T2,T3,T4	-	-	-	-	-	-	-	-
N4	HBV_B2	949	A	SNV	C	0	0	0	7	3	4	10	HVTT,N1,N2,N3,N4,PVTT,T1,T2,T3,T4	-	-	-	-	-	-	-	-
N4	HBV_B2	951	A	SNV	G	0	0	0	7	2	5	10	HVTT,N1,N2,N3,N4,PVTT,T1,T2,T3,T4	-	-	-	-	-	-	-	-
N4	HBV_B2	967	A	SNV	C	0	0	0	7	2	5	9	HVTT,N2,N3,N4,PVTT,T1,T2,T3,T4	-	-	-	-	-	-	-	-
N4	HBV_B2	996	A	SNV	C	0	0	0	6	2	4	9	HVTT,N2,N3,N4,PVTT,T1,T2,T3,T4	-	-	-	-	-	-	-	-
N4	HBV_B2	1131	A	SNV	G	0	0	0	41	39	2	10	HVTT,N1,N2,N3,N4,PVTT,T1,T2,T3,T4	-	-	-	-	-	-	-	-
N4	HBV_B2	1846	T	SNV	A	0	0	0	8	3	5	8	HVTT,N3,N4,PVTT,T1,T2,T3,T4	-	-	-	-	-	-	-	-

N4	HBV_B2	1899	A	SNV	G	0	0	0	8	4	4	10	HVTT,N1,N2,N3,N4,PVTT,T1,T2,T3,T4	-	-	-	-	-	-	-	-
N4	HBV_B2	2003	A	SNV	T	0	0	0	9	5	4	10	HVTT,N1,N2,N3,N4,PVTT,T1,T2,T3,T4	-	-	-	-	-	-	-	-
N4	HBV_B2	2012	T	SNV	C	0	0	0	6	3	3	10	HVTT,N1,N2,N3,N4,PVTT,T1,T2,T3,T4	-	-	-	-	-	-	-	-
N4	HBV_B2	2078	C	SNV	G	0	0	0	8	6	2	10	HVTT,N1,N2,N3,N4,PVTT,T1,T2,T3,T4	-	-	-	-	-	-	-	-
N4	HBV_B2	2136	A	SNV	C	0	0	0	17	9	8	10	HVTT,N1,N2,N3,N4,PVTT,T1,T2,T3,T4	-	-	-	-	-	-	-	-
N4	HBV_B2	2137	G	SNV	A	0	0	0	17	9	8	10	HVTT,N1,N2,N3,N4,PVTT,T1,T2,T3,T4	-	-	-	-	-	-	-	-
N4	HBV_B2	2149	T	SNV	A	0	0	0	18	8	10	10	HVTT,N1,N2,N3,N4,PVTT,T1,T2,T3,T4	-	-	-	-	-	-	-	-
N4	HBV_B2	2159	G	SNV	A	0	0	0	17	7	10	10	HVTT,N1,N2,N3,N4,PVTT,T1,T2,T3,T4	-	-	-	-	-	-	-	-
N4	HBV_B2	2189	C	SNV	A	0	0	0	15	10	5	10	HVTT,N1,N2,N3,N4,PVTT,T1,T2,T3,T4	-	-	-	-	-	-	-	-
N4	HBV_B2	2198	T	SNV	C	0	0	0	14	9	5	10	HVTT,N1,N2,N3,N4,PVTT,T1,T2,T3,T4	-	-	-	-	-	-	-	-
N4	HBV_B2	2233	A	SNV	G	0	0	0	16	9	7	10	HVTT,N1,N2,N3,N4,PVTT,T1,T2,T3,T4	-	-	-	-	-	-	-	-
N4	HBV_B2	2242	G	SNV	T	0	0	0	15	8	7	10	HVTT,N1,N2,N3,N4,PVTT,T1,T2,T3,T4	-	-	-	-	-	-	-	-
N4	HBV_B2	2534	A	SNV	T	0	0	0	7	4	3	10	HVTT,N1,N2,N3,N4,PVTT,T1,T2,T3,T4	-	-	-	-	-	-	-	-
N4	HBV_B2	2721	G	SNV	A	0	0	0	4	4	0	10	HVTT,N1,N2,N3,N4,PVTT,T1,T2,T3,T4	-	-	-	-	-	-	-	-
N4	HBV_B2	2732	C	SNV	T	0	0	0	4	4	0	9	HVTT,N1,N3,N4,PVTT,T1,T2,T3,T4	-	-	-	-	-	-	-	-
N4	HBV_B2	2739	C	SNV	A	0	0	0	4	4	0	9	HVTT,N1,N3,N4,PVTT,T1,T2,T3,T4	-	-	-	-	-	-	-	-
N4	HBV_B2	2771	G	SNV	T	0	0	0	5	3	2	10	HVTT,N1,N2,N3,N4,PVTT,T1,T2,T3,T4	-	-	-	-	-	-	-	-
N4	HBV_B2	2804	T	SNV	C	0	0	0	9	7	2	10	HVTT,N1,N2,N3,N4,PVTT,T1,T2,T3,T4	-	-	-	-	-	-	-	-
N4	HBV_B2	2988	A	SNV	G	0	0	0	13	9	4	10	HVTT,N1,N2,N3,N4,PVTT,T1,T2,T3,T4	-	-	-	-	-	-	-	-
N4	HBV_B2	3039	G	SNV	A	0	0	0	15	8	7	10	HVTT,N1,N2,N3,N4,PVTT,T1,T2,T3,T4	-	-	-	-	-	-	-	-
N4	HBV_B2	3097	C	SNV	A	0	0	0	13	8	5	9	HVTT,N2,N3,N4,PVTT,T1,T2,T3,T4	-	-	-	-	-	-	-	-
N4	HBV_B2	3109	A	SNV	G	0	0	0	14	7	7	9	HVTT,N2,N3,N4,PVTT,T1,T2,T3,T4	-	-	-	-	-	-	-	-
N4	HBV_B2	3118	G	SNV	A	0	0	0	13	5	8	10	HVTT,N1,N2,N3,N4,PVTT,T1,T2,T3,T4	-	-	-	-	-	-	-	-
N4	HBV_B2	3169	T	SNV	A	0	0	0	10	4	6	10	HVTT,N1,N2,N3,N4,PVTT,T1,T2,T3,T4	-	-	-	-	-	-	-	-
N4	HBV_B2	3189	A	SNV	C	0	0	0	7	4	3	9	HVTT,N2,N3,N4,PVTT,T1,T2,T3,T4	-	-	-	-	-	-	-	-
N4	HBV_B2	3198	T	SNV	G	0	0	0	6	2	4	10	HVTT,N1,N2,N3,N4,PVTT,T1,T2,T3,T4	-	-	-	-	-	-	-	-
T1	HBV_B2	20	A	SNV	G	0	0	0	76	48	28	10	HVTT,N1,N2,N3,N4,PVTT,T1,T2,T3,T4	0	0	0	12	3	9	5	HVTT,PVTT,T1,T2,T3
T1	HBV_B2	22	T	SNV	A	0	0	0	76	49	27	10	HVTT,N1,N2,N3,N4,PVTT,T1,T2,T3,T4	0	0	0	12	3	9	5	HVTT,PVTT,T1,T2,T3
T1	HBV_B2	31	T	SNV	G	0	0	0	83	51	32	10	HVTT,N1,N2,N3,N4,PVTT,T1,T2,T3,T4	0	0	0	13	4	9	5	HVTT,PVTT,T1,T2,T3
T1	HBV_B2	53	C	SNV	T	0	0	0	94	49	45	9	HVTT,N1,N2,N3,PVTT,T1,T2,T3,T4	0	0	0	12	4	8	5	HVTT,PVTT,T1,T2,T3
T1	HBV_B2	165	T	SNV	C	0	0	0	64	28	36	10	HVTT,N1,N2,N3,N4,PVTT,T1,T2,T3,T4	0	0	0	7	4	3	6	HVTT,PVTT,T1,T2,T3,T4
T1	HBV_B2	167	G	SNV	A	0	0	0	67	31	36	10	HVTT,N1,N2,N3,N4,PVTT,T1,T2,T3,T4	0	0	0	7	4	3	6	HVTT,PVTT,T1,T2,T3,T4
T1	HBV_B2	216	C	SNV	T	0	0	0	63	31	32	10	HVTT,N1,N2,N3,N4,PVTT,T1,T2,T3,T4	0	0	0	8	4	4	6	HVTT,PVTT,T1,T2,T3,T4
T1	HBV_B2	273	G	SNV	A	0	0	0	54	37	17	10	HVTT,N1,N2,N3,N4,PVTT,T1,T2,T3,T4	0	0	0	10	6	4	5	HVTT,PVTT,T1,T2,T3
T1	HBV_B2	346	T	SNV	C	0	0	0	67	37	30	9	HVTT,N2,N3,N4,PVTT,T1,T2,T3,T4	0	0	0	10	6	4	5	HVTT,PVTT,T1,T2,T3
T1	HBV_B2	381	A	SNV	G	0	0	0	69	41	28	9	HVTT,N2,N3,N4,PVTT,T1,T2,T3,T4	0	0	0	15	10	5	5	HVTT,PVTT,T1,T2,T3
T1	HBV_B2	392	C	SNV	T	0	0	0	66	40	26	9	HVTT,N2,N3,N4,PVTT,T1,T2,T3,T4	0	0	0	13	9	4	5	HVTT,PVTT,T1,T2,T3
T1	HBV_B2	481	A	SNV	C	0	0	0	72	40	32	9	HVTT,N2,N3,N4,PVTT,T1,T2,T3,T4	0	0	0	14	7	7	7	HVTT,N3,PVTT,T1,T2,T3,T4
T1	HBV_B2	562	C	SNV	A	0	0	0	74	40	34	10	HVTT,N1,N2,N3,N4,PVTT,T1,T2,T3,T4	0	0	0	10	3	7	5	HVTT,PVTT,T1,T2,T3
T1	HBV_B2	598	T	SNV	C	0	0	0	71	47	24	10	HVTT,N1,N2,N3,N4,PVTT,T1,T2,T3,T4	0	0	0	8	3	5	5	HVTT,PVTT,T1,T2,T3
T1	HBV_B2	636	T	SNV	A	0	0	0	75	47	28	10	HVTT,N1,N2,N3,N4,PVTT,T1,T2,T3,T4	0	0	0	6	4	2	6	HVTT,PVTT,T1,T2,T3,T4
T1	HBV_B2	645	A	SNV	G	0	0	0	80	50	30	10	HVTT,N1,N2,N3,N4,PVTT,T1,T2,T3,T4	0	0	0	6	4	2	6	HVTT,PVTT,T1,T2,T3,T4
T1	HBV_B2	753	T	SNV	A	0	0	0	74	40	34	7	HVTT,N3,PVTT,T1,T2,T3,T4	0	0	0	8	3	5	6	HVTT,PVTT,T1,T2,T3,T4
T1	HBV_B2	870	C	SNV	T	0	0	0	47	19	28	7	HVTT,N3,PVTT,T1,T2,T3,T4	0	0	0	4	2	2	6	HVTT,PVTT,T1,T2,T3,T4
T1	HBV_B2	912	A	SNV	G	0	0	0	43	22	21	9	HVTT,N1,N2,N3,PVTT,T1,T2,T3,T4	0	0	0	6	4	2	6	HVTT,PVTT,T1,T2,T3,T4
T1	HBV_B2	918	A	SNV	C	0	0	0	41	23	18	10	HVTT,N1,N2,N3,N4,PVTT,T1,T2,T3,T4	0	0	0	7	4	3	6	HVTT,PVTT,T1,T2,T3,T4
T1	HBV_B2	949	A	SNV	C	0	0	0	46	28	18	10	HVTT,N1,N2,N3,N4,PVTT,T1,T2,T3,T4	0	0	0	9	6	3	6	HVTT,PVTT,T1,T2,T3,T4
T1	HBV_B2	951	A	SNV	G	0	0	0	47	29	18	10	HVTT,N1,N2,N3,N4,PVTT,T1,T2,T3,T4	0	0	0	7	6	1	6	HVTT,PVTT,T1,T2,T3,T4
T1	HBV_B2	967	A	SNV	C	0	0	0	50	33	17	9	HVTT,N2,N3,N4,PVTT,T1,T2,T3,T4	0	0	0	10	6	4	6	HVTT,PVTT,T1,T2,T3,T4
T1	HBV_B2	996	A	SNV	C	0	0	0	46	31	15	9	HVTT,N2,N3,N4,PVTT,T1,T2,T3,T4	0	0	0	10	7	3	6	HVTT,PVTT,T1,T2,T3,T4
T1	HBV_B2	1104	A	SNV	C	0	0	0	68	44	24	9	HVTT,N1,N2,N3,PVTT,T1,T2,T3,T4	0	0	0	6	4	2	6	HVTT,PVTT,T1,T2,T3,T4
T1	HBV_B2	1106	C	SNV	T	0	0	0	69	44	25	9	HVTT,N1,N2,N3,PVTT,T1,T2,T3,T4	0	0	0	6	4	2	6	HVTT,PVTT,T1,T2,T3,T4
T1	HBV_B2	1114	A	SNV	G	0	0	0	98	71	27	9	HVTT,N1,N2,N3,PVTT,T1,T2,T3,T4	0	0	0	5	3	2	6	HVTT,PVTT,T1,T2,T3,T4
T1	HBV_B2	1119	T	SNV	C	0	0	0	132	79	53	9	HVTT,N1,N2,N3,PVTT,T1,T2,T3,T4	-	-	-	-	-	-	-	-
T1	HBV_B2	1131	A	SNV	G	0	0	0	198	102	96	10	HVTT,N1,N2,N3,N4,PVTT,T1,T2,T3,T4	-	-	-	-	-	-	-	-
T1	HBV_B2	1846	T	SNV	A	0	0	0	44	33	11	8	HVTT,N3,N4,PVTT,T1,T2,T3,T4	0	0	0	9	3	6	5	HVTT,PVTT,T1,T2,T3
T1	HBV_B2	1899	A	SNV	G	0	0	0	60	35	25	10	HVTT,N1,N2,N3,N4,PVTT,T1,T2,T3,T4	0	0	0	10	2	8	6	HVTT,PVTT,T1,T2,T3,T4
T1	HBV_B2	2003	A	SNV	T	0	0	0	73	45	28	10	HVTT,N1,N2,N3,N4,PVTT,T1,T2,T3,T4	0	0	0	8	4	4	6	HVTT,PVTT,T1,T2,T3,T4
T1	HBV_B2	2012	T	SNV	C	0	0	0	69	42	27	10	HVTT,N1,N2,N3,N4,PVTT,T1,T2,T3,T4	0	0	0	7	4	3	6	HVTT,PVTT,T1,T2,T3,T4
T1	HBV_B2	2078	C	SNV	G	0	0	0	68	38	30	10	HVTT,N1,N2,N3,N4,PVTT,T1,T2,T3,T4	0	0	0	9	6	3	6	HVTT,PVTT,T1,T2,T3,T4
T1	HBV_B2	2136	A	SNV	C	0	0	0	92	52	40	10	HVTT,N1,N2,N3,N4,PVTT,T1,T2,T3,T4	0	0	0	16	10	6	6	HVTT,PVTT,T1,T2,T3,T4
T1	HBV_B2	2137	G	SNV	A	0	0	0	92	52	40	10	HVTT,N1,N2,N3,N4,PVTT,T1,T2,T3,T4	0	0	0	17	10	7	6	HVTT,PVTT,T1,T2,T3,T4
T1	HBV_B2	2149	T	SNV	A	0	0	0	96	59	37	10	HVTT,N1,N2,N3,N4,PVTT,T1,T2,T3,T4	0	0	0	15	9	6	7	HVTT,N3,PVTT,T1,T2,T3,T4
T1	HBV_B2	2159	G	SNV	A	0	0	0	97	57	40	10	HVTT,N1,N2,N3,N4,PVTT,T1,T2,T3,T4	0	0	0	17	10	7	7	HVTT,N3,PVTT,T1,T2,T3,T4
T1	HBV_B2	2189	C	SNV	A	0	0	0	103	68	35	10	HVTT,N1,N2,N3,N4,PVTT,T1,T2,T3,T4	0	0	0	19	11	8	7	HVTT,N3,PVTT,T1,T2,T3,T4
T1	HBV_B2	2198	T	SNV	C	0	0	0	103	66	37	10	HVTT,N1,N2,N3,N4,PVTT,T1,T2,T3,T4	0	0	0	20	11	9	7	HVTT,N3,PVTT,T1,T2,T3,T4
T1	HBV_B2	2233	A	SNV	G	0	0	0	115	70	45	10	HVTT,N1,N2,N3,N4,PVTT,T1,T2,T3,T4	0	0	0	19	10	9	7	HVTT,N3,PVTT,T1,T2,T3,T4
T1	HBV_B2	2242	G	SNV	T	0	0	0	119	69	50	10	HVTT,N1,N2,N3,N4,PVTT,T1,T2,T3,T4	0	0	0	18	11	7	7	HVTT,N3,PVTT,T1,T2,T3,T4
T1	HBV_B2	2534	A	SNV	T	0	0	0	58	30	28	10	HVTT,N1,N2,N3,N4,PVTT,T1,T2,T3,T4	0	0	0	9	3	6	6	HVTT,PVTT,T1,T2,T3,T4
T1	HBV_B2	2572	C	SNV	T	0	0	0	59	28	31	8	HVTT,N1,N3,PVTT,T1,T2,T3,T4	0	0	0	7	3	4	6	HVTT,PVTT,T1,T2,T3,T4
T1	HBV_B2	2699	G	SNV	T	0	0	0	45	26	19	9	HVTT,N1,N2,N3,PVTT,T1,T2,T3,T4	-	-	-	-	-	-	-	-
T1	HBV_B2	2702	C	SNV	T	0	0	0	45	25	20	9	HVTT,N1,N2,N3,PVTT,T1,T2,T3,T4	-	-	-	-	-	-	-	-
T1	HBV_B2	2711	A	SNV	G	0	0	0	47	27	20	9	HVTT,N1,N2,N3,PVTT,T1,T2,T3,T4	-	-	-	-	-	-	-	-
T1	HBV_B2	2712	T	SNV	C	0	0	0	47	27	20	9	HVTT,N1,N2,N3,PVTT,T1,T2,T3,T4	-	-	-	-	-	-	-	-
T1	HBV_B2	2721	G	SNV	A	0	0	0	49	31	18	10	HVTT,N1,N2,N3,N4,PVTT,T1,T2,T3,T4	-	-	-	-	-	-	-	-
T1	HBV_B2	2732	C	SNV	T	0	0	0	47	31	16	9	HVTT,N1,N3,N4,PVTT,T1,T2,T3,T4	0	0	0	5	3	2	6	HVTT,PVTT,T1,T2,T3,T4
T1	HBV_B2	2739	C	SNV	A	0	0	0	47	32	15	9	HVTT,N1,N3,N4,PVTT,T1,T2,T3,T4	0	0	0	5	3	2	6	HVTT,PVTT,T1,T2,T3,T4
T1	HBV_B2	2771	G	SNV	T	0	0	0	57	42	15	10	HVTT,N1,N2,N3,N4,PVTT,T1,T2,T3,T4	0	0	0	7	5	2	6	HVTT,PVTT,T1,T2,T3,T4
T1	HBV_B2	2804	T	SNV	C	0	0	0	74	55	19	10	HVTT,N1,N2,N3,N4,PVTT,T1,T2,T3,T4	0	0	0	10	8	2	6	HVTT,PVTT,T1,T2,T3,T4
T1	HBV_B2	2988	A	SNV	G	0	0	0	77	49	28	10	HVTT,N1,N2,N3,N4,PVTT,T1,T2,T3,T4	0	0	0	11	7	4	6	HVTT,PVTT,T1,T2,T3,T4
T1	HBV_B2	3039	G	SNV	A	0	0	0	80	41	39	10	HVTT,N1,N2,N3,N4,PVTT,T1,T2,T3,T4	0	0	0	14	8	6	6	HVTT,PVTT,T1,T2,T3,T4
T1	HBV_B2	3097	C	SNV	A	0	0	0	69	39	30	9	HVTT,N2,N3,N4,PVTT,T1,T2,T3,T4	0	0	0	8	3	5	5	HVTT,PVTT,T1,T2,T3
T1	HBV_B2	3109	A	SNV	G	0	0	0	72	37	35	9	HVTT,N2,N3,N4,PVTT,T1,T2,T3,T4	0	0	0	8	3	5	5	HVTT,PVTT,T1,T2,T3
T1	HBV_B2	3118	G	SNV	A	0	0	0	71	36	35	10	HVTT,N1,N2,N3,N4,PVTT,T1,T2,T3,T4	0	0	0	8	3	5	5	HVTT,PVTT,T1,T2,T3
T1	HBV_B2	3169	T	SNV	A	0	0	0	64	32	32	10	HVTT,N1,N2,N3,N4,PVTT,T1,T2,T3,T4	-	-	-	-	-	-	-	-
T1	HBV_B2	3189	A	SNV	C	0	0	0	70	43	27	9	HVTT,N2,N3,N4,PVTT,T1,T2,T3,T4	0	0	0	11	2	9	6	HVTT,PVTT,T1,T2,T3,T4
T1	HBV_B2	3198	T	SNV	G	0	0	0	68	40	28	10	HVTT,N1,N2,N3,N4,PVTT,T1,T2,T3,T4	0	0	0	11	3	8	6	HVTT,PVTT,T1,T2,T3,T4
T2	HBV_B2	20	A	SNV	G	0	0	0	68	42	26	10	HVTT,N1,N2,N3,N4,PVTT,T1,T2,T3,T4	0	0	0	16	9	7	5	HVTT,PVTT,T1,T2,T3
T2	HBV_B2	22	T	SNV	A	0	0	0	71	42	29	10	HVTT,N1,N2,N3,N4,PVTT,T1,T2,T3,T4	0	0	0	17	9	8	5	HVTT,PVTT,T1,T2,T3
T2	HBV_B2	31	T	SNV	G	0	0	0	75	43	32	10	HVTT,N1,N2,N3,N4,PVTT,T1,T2,T3,T4	0	0	0	18	10	8	5	HVTT,PVTT,T1,T2,T3
T2	HBV_B2	53	C	SNV	T	0	0	0	79	44	35	9	HVTT,N1,N2,N3,PVTT,T1,T2,T3,T4	0	0	0	19	11	8	5	HVTT,PVTT,T1,T2,T3
T2	HBV_B2	165	T	SNV	C	0	0	0	61	31	30	10	HVTT,N1,N2,N3,N4,PVTT,T1,T2,T3,T4	0	0	0	14	7	7	6	HVTT,PVTT,T1,T2,T3,T4
T2	HBV_B2	167	G	SNV	A	0	0	0	61	30	31	10	HVTT,N1,N2,N3,N4,PVTT,T1,T2,T3,T4	0	0	0	14	7	7	6	HVTT,PVTT,T1,T2,T3,T4
T2	HBV_B2	216	C	SNV	T	0	0	0	47	26	21	10	HVTT,N1,N2,N3,N4,PVTT,T1,T2,T3,T4	0	0	0	13	4	9	6	HVTT,PVTT,T1,T2,T3,T4
T2	HBV_B2	273	G	SNV	A	0	0	0	45	27	18	10	HVTT,N1,N2,N3,N4,PVTT,T1,T2,T3,T4	0	0	0	10	4	6	5	HVTT,PVTT,T1,T2,T3
T2	HBV_B2	346	T	SNV	C	0	0	0	47	29	18	9	HVTT,N2,N3,N4,PVTT,T1,T2,T3,T4	0	0	0	9	3	6	5	HVTT,PVTT,T1,T2,T3
T2	HBV_B2	381	A	SNV	G	0	0	0	45	24	21	9	HVTT,N2,N3,N4,PVTT,T1,T2,T3,T4	0	0	0	9	3	6	5	HVTT,PVTT,T1,T2,T3

T2	HBV_B2	392	C	SNV	T	0	0	0	47	27	20	9	HVTT,N2,N3,N4,PVTT,T1,T2,T3,T4	0	0	0	8	2	6	5	HVTT,PVTT,T1,T2,T3
T2	HBV_B2	481	A	SNV	C	0	0	0	57	45	12	9	HVTT,N2,N3,N4,PVTT,T1,T2,T3,T4	0	0	0	8	4	4	7	HVTT,N3,PVTT,T1,T2,T3,T4
T2	HBV_B2	562	C	SNV	A	0	0	0	65	36	29	10	HVTT,N1,N2,N3,N4,PVTT,T1,T2,T3,T4	0	0	0	9	5	4	5	HVTT,PVTT,T1,T2,T3
T2	HBV_B2	598	T	SNV	C	0	0	0	59	32	27	10	HVTT,N1,N2,N3,N4,PVTT,T1,T2,T3,T4	0	0	0	6	3	3	5	HVTT,PVTT,T1,T2,T3
T2	HBV_B2	636	T	SNV	A	0	0	0	63	40	23	10	HVTT,N1,N2,N3,N4,PVTT,T1,T2,T3,T4	0	0	0	6	4	2	6	HVTT,PVTT,T1,T2,T3,T4
T2	HBV_B2	645	A	SNV	G	0	0	0	69	43	26	10	HVTT,N1,N2,N3,N4,PVTT,T1,T2,T3,T4	0	0	0	7	5	2	6	HVTT,PVTT,T1,T2,T3,T4
T2	HBV_B2	753	T	SNV	A	0	0	0	60	28	32	7	HVTT,N3,PVTT,T1,T2,T3,T4	0	0	0	15	10	5	6	HVTT,PVTT,T1,T2,T3,T4
T2	HBV_B2	870	C	SNV	T	0	0	0	46	31	15	7	HVTT,N3,PVTT,T1,T2,T3,T4	0	0	0	12	7	5	6	HVTT,PVTT,T1,T2,T3,T4
T2	HBV_B2	912	A	SNV	G	0	0	0	46	30	16	9	HVTT,N1,N2,N3,PVTT,T1,T2,T3,T4	0	0	0	13	7	6	6	HVTT,PVTT,T1,T2,T3,T4
T2	HBV_B2	918	A	SNV	C	0	0	0	47	30	17	10	HVTT,N1,N2,N3,N4,PVTT,T1,T2,T3,T4	0	0	0	14	8	6	6	HVTT,PVTT,T1,T2,T3,T4
T2	HBV_B2	949	A	SNV	C	0	0	0	53	39	14	10	HVTT,N1,N2,N3,N4,PVTT,T1,T2,T3,T4	0	0	0	20	11	9	6	HVTT,PVTT,T1,T2,T3,T4
T2	HBV_B2	951	A	SNV	G	0	0	0	54	38	16	10	HVTT,N1,N2,N3,N4,PVTT,T1,T2,T3,T4	0	0	0	21	12	9	6	HVTT,PVTT,T1,T2,T3,T4
T2	HBV_B2	967	A	SNV	C	0	0	0	59	40	19	9	HVTT,N2,N3,N4,PVTT,T1,T2,T3,T4	0	0	0	20	11	9	6	HVTT,PVTT,T1,T2,T3,T4
T2	HBV_B2	996	A	SNV	C	0	0	0	61	40	21	9	HVTT,N2,N3,N4,PVTT,T1,T2,T3,T4	0	0	0	20	8	12	6	HVTT,PVTT,T1,T2,T3,T4
T2	HBV_B2	1104	A	SNV	C	0	0	0	63	34	29	9	HVTT,N1,N2,N3,PVTT,T1,T2,T3,T4	0	0	0	10	4	6	6	HVTT,PVTT,T1,T2,T3,T4
T2	HBV_B2	1106	C	SNV	T	0	0	0	67	38	29	9	HVTT,N1,N2,N3,PVTT,T1,T2,T3,T4	0	0	0	10	4	6	6	HVTT,PVTT,T1,T2,T3,T4
T2	HBV_B2	1114	A	SNV	G	0	0	0	169	141	28	9	HVTT,N1,N2,N3,PVTT,T1,T2,T3,T4	0	0	0	9	4	5	6	HVTT,PVTT,T1,T2,T3,T4
T2	HBV_B2	1119	T	SNV	C	0	0	0	190	142	48	9	HVTT,N1,N2,N3,PVTT,T1,T2,T3,T4	0	0	0	8	3	5	5	HVTT,PVTT,T2,T3,T4
T2	HBV_B2	1131	A	SNV	G	0	0	0	288	205	83	10	HVTT,N1,N2,N3,N4,PVTT,T1,T2,T3,T4	0	0	0	7	3	4	5	HVTT,PVTT,T2,T3,T4
T2	HBV_B2	1846	T	SNV	A	0	0	0	61	44	17	8	HVTT,N3,N4,PVTT,T1,T2,T3,T4	0	0	0	6	3	3	5	HVTT,PVTT,T1,T2,T3
T2	HBV_B2	1899	A	SNV	G	0	0	0	66	37	29	10	HVTT,N1,N2,N3,N4,PVTT,T1,T2,T3,T4	0	0	0	10	5	5	6	HVTT,PVTT,T1,T2,T3,T4
T2	HBV_B2	2003	A	SNV	T	0	0	0	61	41	20	10	HVTT,N1,N2,N3,N4,PVTT,T1,T2,T3,T4	0	0	0	8	4	4	6	HVTT,PVTT,T1,T2,T3,T4
T2	HBV_B2	2012	T	SNV	C	0	0	0	59	38	21	10	HVTT,N1,N2,N3,N4,PVTT,T1,T2,T3,T4	0	0	0	11	4	7	6	HVTT,PVTT,T1,T2,T3,T4
T2	HBV_B2	2078	C	SNV	G	0	0	0	66	38	28	10	HVTT,N1,N2,N3,N4,PVTT,T1,T2,T3,T4	0	0	0	7	3	4	6	HVTT,PVTT,T1,T2,T3,T4
T2	HBV_B2	2136	A	SNV	C	0	0	0	109	73	36	10	HVTT,N1,N2,N3,N4,PVTT,T1,T2,T3,T4	0	0	0	21	9	12	6	HVTT,PVTT,T1,T2,T3,T4
T2	HBV_B2	2137	G	SNV	A	0	0	0	110	74	36	10	HVTT,N1,N2,N3,N4,PVTT,T1,T2,T3,T4	0	0	0	19	9	10	6	HVTT,PVTT,T1,T2,T3,T4
T2	HBV_B2	2149	T	SNV	A	0	0	0	114	77	37	10	HVTT,N1,N2,N3,N4,PVTT,T1,T2,T3,T4	0	0	0	20	8	12	7	HVTT,N3,PVTT,T1,T2,T3,T4
T2	HBV_B2	2159	G	SNV	A	0	0	0	109	75	34	10	HVTT,N1,N2,N3,N4,PVTT,T1,T2,T3,T4	0	0	0	19	8	11	7	HVTT,N3,PVTT,T1,T2,T3,T4
T2	HBV_B2	2189	C	SNV	A	0	0	0	111	64	47	10	HVTT,N1,N2,N3,N4,PVTT,T1,T2,T3,T4	0	0	0	19	10	9	7	HVTT,N3,PVTT,T1,T2,T3,T4
T2	HBV_B2	2198	T	SNV	C	0	0	0	108	64	44	10	HVTT,N1,N2,N3,N4,PVTT,T1,T2,T3,T4	0	0	0	22	12	10	7	HVTT,N3,PVTT,T1,T2,T3,T4
T2	HBV_B2	2233	A	SNV	G	0	0	0	115	61	54	10	HVTT,N1,N2,N3,N4,PVTT,T1,T2,T3,T4	0	0	0	18	10	8	7	HVTT,N3,PVTT,T1,T2,T3,T4
T2	HBV_B2	2242	G	SNV	T	0	0	0	111	62	49	10	HVTT,N1,N2,N3,N4,PVTT,T1,T2,T3,T4	0	0	0	16	10	6	7	HVTT,N3,PVTT,T1,T2,T3,T4
T2	HBV_B2	2534	A	SNV	T	0	0	0	51	33	18	10	HVTT,N1,N2,N3,N4,PVTT,T1,T2,T3,T4	0	0	0	10	5	5	6	HVTT,PVTT,T1,T2,T3,T4
T2	HBV_B2	2572	C	SNV	T	0	0	0	51	29	22	8	HVTT,N1,N3,PVTT,T1,T2,T3,T4	0	0	0	12	4	8	6	HVTT,PVTT,T1,T2,T3,T4
T2	HBV_B2	2699	G	SNV	T	0	0	0	44	28	16	9	HVTT,N1,N2,N3,PVTT,T1,T2,T3,T4	0	0	0	8	2	6	4	HVTT,PVTT,T2,T3
T2	HBV_B2	2702	C	SNV	T	0	0	0	42	27	15	9	HVTT,N1,N2,N3,PVTT,T1,T2,T3,T4	0	0	0	8	2	6	4	HVTT,PVTT,T2,T3
T2	HBV_B2	2711	A	SNV	G	0	0	0	48	35	13	9	HVTT,N1,N2,N3,PVTT,T1,T2,T3,T4	0	0	0	7	2	5	4	HVTT,PVTT,T2,T3
T2	HBV_B2	2712	T	SNV	C	0	0	0	48	35	13	9	HVTT,N1,N2,N3,PVTT,T1,T2,T3,T4	0	0	0	7	2	5	4	HVTT,PVTT,T2,T3
T2	HBV_B2	2721	G	SNV	A	0	0	0	50	34	16	10	HVTT,N1,N2,N3,N4,PVTT,T1,T2,T3,T4	0	0	0	7	2	5	5	HVTT,PVTT,T2,T3,T4
T2	HBV_B2	2732	C	SNV	T	0	0	0	53	35	18	9	HVTT,N1,N3,N4,PVTT,T1,T2,T3,T4	0	0	0	7	2	5	6	HVTT,PVTT,T1,T2,T3,T4
T2	HBV_B2	2739	C	SNV	A	0	0	0	50	36	14	9	HVTT,N1,N3,N4,PVTT,T1,T2,T3,T4	0	0	0	6	2	4	6	HVTT,PVTT,T1,T2,T3,T4
T2	HBV_B2	2771	G	SNV	T	0	0	0	60	44	16	10	HVTT,N1,N2,N3,N4,PVTT,T1,T2,T3,T4	0	0	0	5	2	3	6	HVTT,PVTT,T1,T2,T3,T4
T2	HBV_B2	2804	T	SNV	C	0	0	0	64	40	24	10	HVTT,N1,N2,N3,N4,PVTT,T1,T2,T3,T4	0	0	0	6	4	2	6	HVTT,PVTT,T1,T2,T3,T4
T2	HBV_B2	2988	A	SNV	G	0	0	0	63	41	22	10	HVTT,N1,N2,N3,N4,PVTT,T1,T2,T3,T4	0	0	0	8	6	2	6	HVTT,PVTT,T1,T2,T3,T4
T2	HBV_B2	3039	G	SNV	A	0	0	0	71	44	27	10	HVTT,N1,N2,N3,N4,PVTT,T1,T2,T3,T4	0	0	0	7	5	2	6	HVTT,PVTT,T1,T2,T3,T4
T2	HBV_B2	3097	C	SNV	A	0	0	0	68	40	28	9	HVTT,N2,N3,N4,PVTT,T1,T2,T3,T4	0	0	0	9	5	4	5	HVTT,PVTT,T1,T2,T3
T2	HBV_B2	3109	A	SNV	G	0	0	0	69	43	26	9	HVTT,N2,N3,N4,PVTT,T1,T2,T3,T4	0	0	0	9	5	4	5	HVTT,PVTT,T1,T2,T3
T2	HBV_B2	3118	G	SNV	A	0	0	0	67	41	26	10	HVTT,N1,N2,N3,N4,PVTT,T1,T2,T3,T4	0	0	0	9	5	4	5	HVTT,PVTT,T1,T2,T3
T2	HBV_B2	3169	T	SNV	A	0	0	0	68	33	35	10	HVTT,N1,N2,N3,N4,PVTT,T1,T2,T3,T4	0	0	0	15	9	6	5	HVTT,PVTT,T2,T3,T4
T2	HBV_B2	3189	A	SNV	C	0	0	0	70	38	32	9	HVTT,N2,N3,N4,PVTT,T1,T2,T3,T4	0	0	0	14	8	6	6	HVTT,PVTT,T1,T2,T3,T4
T2	HBV_B2	3198	T	SNV	G	0	0	0	67	39	28	10	HVTT,N1,N2,N3,N4,PVTT,T1,T2,T3,T4	0	0	0	17	8	9	6	HVTT,PVTT,T1,T2,T3,T4
T3	HBV_B2	20	A	SNV	G	0	0	0	59	33	26	10	HVTT,N1,N2,N3,N4,PVTT,T1,T2,T3,T4	0	0	0	12	9	3	5	HVTT,PVTT,T1,T2,T3
T3	HBV_B2	22	T	SNV	A	0	0	0	61	34	27	10	HVTT,N1,N2,N3,N4,PVTT,T1,T2,T3,T4	0	0	0	12	9	3	5	HVTT,PVTT,T1,T2,T3
T3	HBV_B2	31	T	SNV	G	0	0	0	60	34	26	10	HVTT,N1,N2,N3,N4,PVTT,T1,T2,T3,T4	0	0	0	14	10	4	5	HVTT,PVTT,T1,T2,T3
T3	HBV_B2	53	C	SNV	T	0	0	0	73	48	25	9	HVTT,N1,N2,N3,PVTT,T1,T2,T3,T4	0	0	0	13	10	3	5	HVTT,PVTT,T1,T2,T3
T3	HBV_B2	165	T	SNV	C	0	0	0	80	43	37	10	HVTT,N1,N2,N3,N4,PVTT,T1,T2,T3,T4	0	0	0	15	9	6	6	HVTT,PVTT,T1,T2,T3,T4
T3	HBV_B2	167	G	SNV	A	0	0	0	80	42	38	10	HVTT,N1,N2,N3,N4,PVTT,T1,T2,T3,T4	0	0	0	16	9	7	6	HVTT,PVTT,T1,T2,T3,T4
T3	HBV_B2	216	C	SNV	T	0	0	0	78	43	35	10	HVTT,N1,N2,N3,N4,PVTT,T1,T2,T3,T4	0	0	0	17	6	11	6	HVTT,PVTT,T1,T2,T3,T4
T3	HBV_B2	273	G	SNV	A	0	0	0	72	44	28	10	HVTT,N1,N2,N3,N4,PVTT,T1,T2,T3,T4	0	0	0	15	7	8	5	HVTT,PVTT,T1,T2,T3
T3	HBV_B2	346	T	SNV	C	0	0	0	72	38	34	9	HVTT,N2,N3,N4,PVTT,T1,T2,T3,T4	0	0	0	13	5	8	5	HVTT,PVTT,T1,T2,T3
T3	HBV_B2	381	A	SNV	G	0	0	0	71	41	30	9	HVTT,N2,N3,N4,PVTT,T1,T2,T3,T4	0	0	0	16	7	9	5	HVTT,PVTT,T1,T2,T3
T3	HBV_B2	392	C	SNV	T	0	0	0	72	40	32	9	HVTT,N2,N3,N4,PVTT,T1,T2,T3,T4	0	0	0	16	7	9	5	HVTT,PVTT,T1,T2,T3
T3	HBV_B2	481	A	SNV	C	0	0	0	65	38	27	9	HVTT,N2,N3,N4,PVTT,T1,T2,T3,T4	0	0	0	11	6	5	7	HVTT,N3,PVTT,T1,T2,T3,T4
T3	HBV_B2	562	C	SNV	A	0	0	0	61	39	22	10	HVTT,N1,N2,N3,N4,PVTT,T1,T2,T3,T4	0	0	0	11	5	6	5	HVTT,PVTT,T1,T2,T3
T3	HBV_B2	598	T	SNV	C	0	0	0	67	43	24	10	HVTT,N1,N2,N3,N4,PVTT,T1,T2,T3,T4	0	0	0	15	8	7	5	HVTT,PVTT,T1,T2,T3
T3	HBV_B2	636	T	SNV	A	0	0	0	69	46	23	10	HVTT,N1,N2,N3,N4,PVTT,T1,T2,T3,T4	0	0	0	17	10	7	6	HVTT,PVTT,T1,T2,T3,T4
T3	HBV_B2	645	A	SNV	G	0	0	0	67	39	28	10	HVTT,N1,N2,N3,N4,PVTT,T1,T2,T3,T4	0	0	0	17	10	7	6	HVTT,PVTT,T1,T2,T3,T4
T3	HBV_B2	753	T	SNV	A	0	0	0	66	44	22	7	HVTT,N3,PVTT,T1,T2,T3,T4	0	0	0	9	5	4	6	HVTT,PVTT,T1,T2,T3,T4
T3	HBV_B2	870	C	SNV	T	0	0	0	68	32	36	7	HVTT,N3,PVTT,T1,T2,T3,T4	0	0	0	14	8	6	6	HVTT,PVTT,T1,T2,T3,T4
T3	HBV_B2	912	A	SNV	G	0	0	0	53	23	30	9	HVTT,N1,N2,N3,PVTT,T1,T2,T3,T4	0	0	0	14	6	8	6	HVTT,PVTT,T1,T2,T3,T4
T3	HBV_B2	918	A	SNV	C	0	0	0	53	25	28	10	HVTT,N1,N2,N3,N4,PVTT,T1,T2,T3,T4	0	0	0	16	7	9	6	HVTT,PVTT,T1,T2,T3,T4
T3	HBV_B2	949	A	SNV	C	0	0	0	41	26	15	10	HVTT,N1,N2,N3,N4,PVTT,T1,T2,T3,T4	0	0	0	20	10	10	6	HVTT,PVTT,T1,T2,T3,T4
T3	HBV_B2	951	A	SNV	G	0	0	0	41	26	15	10	HVTT,N1,N2,N3,N4,PVTT,T1,T2,T3,T4	0	0	0	20	10	10	6	HVTT,PVTT,T1,T2,T3,T4
T3	HBV_B2	967	A	SNV	C	0	0	0	41	29	12	9	HVTT,N2,N3,N4,PVTT,T1,T2,T3,T4	0	0	0	21	12	9	6	HVTT,PVTT,T1,T2,T3,T4
T3	HBV_B2	996	A	SNV	C	0	0	0	49	31	18	9	HVTT,N2,N3,N4,PVTT,T1,T2,T3,T4	0	0	0	23	14	9	6	HVTT,PVTT,T1,T2,T3,T4
T3	HBV_B2	1104	A	SNV	C	0	0	0	65	43	22	9	HVTT,N1,N2,N3,PVTT,T1,T2,T3,T4	0	0	0	15	9	6	6	HVTT,PVTT,T1,T2,T3,T4
T3	HBV_B2	1106	C	SNV	T	0	0	0	67	45	22	9	HVTT,N1,N2,N3,PVTT,T1,T2,T3,T4	0	0	0	14	8	6	6	HVTT,PVTT,T1,T2,T3,T4
T3	HBV_B2	1114	A	SNV	G	0	0	0	221	187	34	9	HVTT,N1,N2,N3,PVTT,T1,T2,T3,T4	0	0	0	14	8	6	6	HVTT,PVTT,T1,T2,T3,T4
T3	HBV_B2	1119	T	SNV	C	0	0	0	467	234	233	9	HVTT,N1,N2,N3,PVTT,T1,T2,T3,T4	0	0	0	15	8	7	5	HVTT,PVTT,T2,T3,T4
T3	HBV_B2	1131	A	SNV	G	0	0	0	755	442	313	10	HVTT,N1,N2,N3,N4,PVTT,T1,T2,T3,T4	0	0	0	15	8	7	5	HVTT,PVTT,T2,T3,T4
T3	HBV_B2	1846	T	SNV	A	0	0	0	50	32	18	8	HVTT,N3,N4,PVTT,T1,T2,T3,T4	0	0	0	15	6	9	5	HVTT,PVTT,T1,T2,T3
T3	HBV_B2	1899	A	SNV	G	0	0	0	63	36	27	10	HVTT,N1,N2,N3,N4,PVTT,T1,T2,T3,T4	0	0	0	18	9	9	6	HVTT,PVTT,T1,T2,T3,T4
T3	HBV_B2	2003	A	SNV	T	0	0	0	63	41	22	10	HVTT,N1,N2,N3,N4,PVTT,T1,T2,T3,T4	0	0	0	16	10	6	6	HVTT,PVTT,T1,T2,T3,T4
T3	HBV_B2	2012	T	SNV	C	0	0	0	61	39	22	10	HVTT,N1,N2,N3,N4,PVTT,T1,T2,T3,T4	0	0	0	15	9	6	6	HVTT,PVTT,T1,T2,T3,T4
T3	HBV_B2	2078	C	SNV	G	0	0	0	66	42	24	10	HVTT,N1,N2,N3,N4,PVTT,T1,T2,T3,T4	0	0	0	15	7	8	6	HVTT,PVTT,T1,T2,T3,T4
T3	HBV_B2	2136	A	SNV	C	0	0	0	107	66	41	10	HVTT,N1,N2,N3,N4,PVTT,T1,T2,T3,T4	0	0	0	21	9	12	6	HVTT,PVTT,T1,T2,T3,T4
T3	HBV_B2	2137	G	SNV	A	0	0	0	107	66	41	10	HVTT,N1,N2,N3,N4,PVTT,T1,T2,T3,T4	0	0	0	21	9	12	6	HVTT,PVTT,T1,T2,T3,T4
T3	HBV_B2	2149	T	SNV	A	0	0	0	113	69	44	10	HVTT,N1,N2,N3,N4,PVTT,T1,T2,T3,T4	0	0	0	22	11	11	7	HVTT,N3,PVTT,T1,T2,T3,T4
T3	HBV_B2	2159	G	SNV	A	0	0	0	112	70	42	10	HVTT,N1,N2,N3,N4,PVTT,T1,T2,T3,T4	0	0	0	22	11	11	7	HVTT,N3,PVTT,T1,T2,T3,T4
T3	HBV_B2	2189	C	SNV	A	0	0	0	117	80	37	10	HVTT,N1,N2,N3,N4,PVTT,T1,T2,T3,T4	0	0	0	26	12	14	7	HVTT,N3,PVTT,T1,T2,T3,T4
T3	HBV_B2	2198	T	SNV	C	0	0	0	115	76	39	10	HVTT,N1,N2,N3,N4,PVTT,T1,T2,T3,T4	0	0	0	29	15	14	7	HVTT,N3,PVTT,T1,T2,T3,T4
T3	HBV_B2	2233	A	SNV	G	0	0	0	137	83	54	10	HVTT,N1,N2,N3,N4,PVTT,T1,T2,T3,T4	0	0	0	38	20	18	7	HVTT,N3,PVTT,T1,T2,T3,T4
T3	HBV_B2	2242	G	SNV	T	0	0	0	141	87	54	10	HVTT,N1,N2,N3,N4,PVTT,T1,T2,T3,T4	0	0	0	38	20	18	7	HVTT,N3,PVTT,T1,T2,T3,T4
T3	HBV_B2	2534	A	SNV	T	0	0	0	57	34	23	10	HVTT,N1,N2,N3,N4,PVTT,T1,T2,T3,T4	0	0	0	13	5	8	6	HVTT,PVTT,T1,T2,T3,T4
T3	HBV_B2	2572	C	SNV	T	0	0	0	59	35	24	8	HVTT,N1,N3,PVTT,T1,T2,T3,T4	0	0	0	11	4	7	6	HVTT,PVTT,T1,T2,T3,T4
T3	HBV_B2	2699	G	SNV	T	0	0	0	56	33	23	9	HVTT,N1,N2,N3,PVTT,T1,T2,T3,T4	0	0	0	14	8	6	4	HVTT,PVTT,T2,T3
T3	HBV_B2	2702	C	SNV	T	0	0	0	55	30	25	9	HVTT,N1,N2,N3,PVTT,T1,T2,T3,T4	0	0	0	14	8	6	4	HVTT,PVTT,T2,T3

T3	HBV_B2	2711	A	SNV	G	0	0	0	57	36	21	9	HVTT,N1,N2,N3,PVTT,T1,T2,T3,T4	0	0	0	15	7	8	4	HVTT,PVTT,T2,T3
T3	HBV_B2	2712	T	SNV	C	0	0	0	58	37	21	9	HVTT,N1,N2,N3,PVTT,T1,T2,T3,T4	0	0	0	15	7	8	4	HVTT,PVTT,T2,T3
T3	HBV_B2	2721	G	SNV	A	0	0	0	58	39	19	10	HVTT,N1,N2,N3,N4,PVTT,T1,T2,T3,T4	0	0	0	16	8	8	5	HVTT,PVTT,T2,T3,T4
T3	HBV_B2	2732	C	SNV	T	0	0	0	63	41	22	9	HVTT,N1,N3,N4,PVTT,T1,T2,T3,T4	0	0	0	15	8	7	6	HVTT,PVTT,T1,T2,T3,T4
T3	HBV_B2	2739	C	SNV	A	0	0	0	67	42	25	9	HVTT,N1,N3,N4,PVTT,T1,T2,T3,T4	0	0	0	15	8	7	6	HVTT,PVTT,T1,T2,T3,T4
T3	HBV_B2	2771	G	SNV	T	0	0	0	72	51	21	10	HVTT,N1,N2,N3,N4,PVTT,T1,T2,T3,T4	0	0	0	13	5	8	6	HVTT,PVTT,T1,T2,T3,T4
T3	HBV_B2	2804	T	SNV	C	0	0	0	77	52	25	10	HVTT,N1,N2,N3,N4,PVTT,T1,T2,T3,T4	0	0	0	13	5	8	6	HVTT,PVTT,T1,T2,T3,T4
T3	HBV_B2	2988	A	SNV	G	0	0	0	78	58	20	10	HVTT,N1,N2,N3,N4,PVTT,T1,T2,T3,T4	0	0	0	10	9	1	6	HVTT,PVTT,T1,T2,T3,T4
T3	HBV_B2	3039	G	SNV	A	0	0	0	87	44	43	10	HVTT,N1,N2,N3,N4,PVTT,T1,T2,T3,T4	0	0	0	7	3	4	6	HVTT,PVTT,T1,T2,T3,T4
T3	HBV_B2	3097	C	SNV	A	0	0	0	78	40	38	9	HVTT,N2,N3,N4,PVTT,T1,T2,T3,T4	0	0	0	10	3	7	5	HVTT,PVTT,T1,T2,T3
T3	HBV_B2	3109	A	SNV	G	0	0	0	73	33	40	9	HVTT,N2,N3,N4,PVTT,T1,T2,T3,T4	0	0	0	10	3	7	5	HVTT,PVTT,T1,T2,T3
T3	HBV_B2	3118	G	SNV	A	0	0	0	69	35	34	10	HVTT,N1,N2,N3,N4,PVTT,T1,T2,T3,T4	0	0	0	10	3	7	5	HVTT,PVTT,T1,T2,T3
T3	HBV_B2	3169	T	SNV	A	0	0	0	57	29	28	10	HVTT,N1,N2,N3,N4,PVTT,T1,T2,T3,T4	0	0	0	12	6	6	5	HVTT,PVTT,T2,T3,T4
T3	HBV_B2	3189	A	SNV	C	0	0	0	55	28	27	9	HVTT,N2,N3,N4,PVTT,T1,T2,T3,T4	0	0	0	13	8	5	6	HVTT,PVTT,T1,T2,T3,T4
T3	HBV_B2	3198	T	SNV	G	0	0	0	59	30	29	10	HVTT,N1,N2,N3,N4,PVTT,T1,T2,T3,T4	0	0	0	13	8	5	6	HVTT,PVTT,T1,T2,T3,T4
T4	HBV_B2	20	A	SNV	G	0	0	0	40	26	14	10	HVTT,N1,N2,N3,N4,PVTT,T1,T2,T3,T4	-	-	-	-	-	-	-	-
T4	HBV_B2	22	T	SNV	A	0	0	0	41	28	13	10	HVTT,N1,N2,N3,N4,PVTT,T1,T2,T3,T4	-	-	-	-	-	-	-	-
T4	HBV_B2	31	T	SNV	G	0	0	0	44	27	17	10	HVTT,N1,N2,N3,N4,PVTT,T1,T2,T3,T4	-	-	-	-	-	-	-	-
T4	HBV_B2	53	C	SNV	T	0	0	0	49	27	22	9	HVTT,N1,N2,N3,PVTT,T1,T2,T3,T4	-	-	-	-	-	-	-	-
T4	HBV_B2	165	T	SNV	C	0	0	0	36	22	14	10	HVTT,N1,N2,N3,N4,PVTT,T1,T2,T3,T4	0	0	0	9	4	5	6	HVTT,PVTT,T1,T2,T3,T4
T4	HBV_B2	167	G	SNV	A	0	0	0	35	22	13	10	HVTT,N1,N2,N3,N4,PVTT,T1,T2,T3,T4	0	0	0	9	4	5	6	HVTT,PVTT,T1,T2,T3,T4
T4	HBV_B2	216	C	SNV	T	0	0	0	32	18	14	10	HVTT,N1,N2,N3,N4,PVTT,T1,T2,T3,T4	0	0	0	11	5	6	6	HVTT,PVTT,T1,T2,T3,T4
T4	HBV_B2	273	G	SNV	A	0	0	0	31	19	12	10	HVTT,N1,N2,N3,N4,PVTT,T1,T2,T3,T4	-	-	-	-	-	-	-	-
T4	HBV_B2	346	T	SNV	C	0	0	0	31	15	16	9	HVTT,N2,N3,N4,PVTT,T1,T2,T3,T4	-	-	-	-	-	-	-	-
T4	HBV_B2	381	A	SNV	G	0	0	0	36	20	16	9	HVTT,N2,N3,N4,PVTT,T1,T2,T3,T4	-	-	-	-	-	-	-	-
T4	HBV_B2	392	C	SNV	T	0	0	0	38	25	13	9	HVTT,N2,N3,N4,PVTT,T1,T2,T3,T4	-	-	-	-	-	-	-	-
T4	HBV_B2	481	A	SNV	C	0	0	0	41	28	13	9	HVTT,N2,N3,N4,PVTT,T1,T2,T3,T4	0	0	0	7	2	5	7	HVTT,N3,PVTT,T1,T2,T3,T4
T4	HBV_B2	562	C	SNV	A	0	0	0	46	34	12	10	HVTT,N1,N2,N3,N4,PVTT,T1,T2,T3,T4	-	-	-	-	-	-	-	-
T4	HBV_B2	598	T	SNV	C	0	0	0	47	26	21	10	HVTT,N1,N2,N3,N4,PVTT,T1,T2,T3,T4	-	-	-	-	-	-	-	-
T4	HBV_B2	636	T	SNV	A	0	0	0	47	26	21	10	HVTT,N1,N2,N3,N4,PVTT,T1,T2,T3,T4	0	0	0	7	2	5	6	HVTT,PVTT,T1,T2,T3,T4
T4	HBV_B2	645	A	SNV	G	0	0	0	45	26	19	10	HVTT,N1,N2,N3,N4,PVTT,T1,T2,T3,T4	0	0	0	8	3	5	6	HVTT,PVTT,T1,T2,T3,T4
T4	HBV_B2	753	T	SNV	A	0	0	0	38	22	16	7	HVTT,N3,PVTT,T1,T2,T3,T4	0	0	0	9	5	4	6	HVTT,PVTT,T1,T2,T3,T4
T4	HBV_B2	870	C	SNV	T	0	0	0	33	23	10	7	HVTT,N3,PVTT,T1,T2,T3,T4	0	0	0	8	6	2	6	HVTT,PVTT,T1,T2,T3,T4
T4	HBV_B2	912	A	SNV	G	0	0	0	27	13	14	9	HVTT,N1,N2,N3,PVTT,T1,T2,T3,T4	0	0	0	7	3	4	6	HVTT,PVTT,T1,T2,T3,T4
T4	HBV_B2	918	A	SNV	C	0	0	0	28	14	14	10	HVTT,N1,N2,N3,N4,PVTT,T1,T2,T3,T4	0	0	0	7	3	4	6	HVTT,PVTT,T1,T2,T3,T4
T4	HBV_B2	949	A	SNV	C	0	0	0	25	13	12	10	HVTT,N1,N2,N3,N4,PVTT,T1,T2,T3,T4	0	0	0	9	3	6	6	HVTT,PVTT,T1,T2,T3,T4
T4	HBV_B2	951	A	SNV	G	0	0	0	25	15	10	10	HVTT,N1,N2,N3,N4,PVTT,T1,T2,T3,T4	0	0	0	9	3	6	6	HVTT,PVTT,T1,T2,T3,T4
T4	HBV_B2	967	A	SNV	C	0	0	0	28	16	12	9	HVTT,N2,N3,N4,PVTT,T1,T2,T3,T4	0	0	0	9	2	7	6	HVTT,PVTT,T1,T2,T3,T4
T4	HBV_B2	996	A	SNV	C	0	0	0	27	16	11	9	HVTT,N2,N3,N4,PVTT,T1,T2,T3,T4	0	0	0	12	5	7	6	HVTT,PVTT,T1,T2,T3,T4
T4	HBV_B2	1104	A	SNV	C	0	0	0	33	21	12	9	HVTT,N1,N2,N3,PVTT,T1,T2,T3,T4	0	0	0	16	10	6	6	HVTT,PVTT,T1,T2,T3,T4
T4	HBV_B2	1106	C	SNV	T	0	0	0	32	21	11	9	HVTT,N1,N2,N3,PVTT,T1,T2,T3,T4	0	0	0	16	10	6	6	HVTT,PVTT,T1,T2,T3,T4
T4	HBV_B2	1114	A	SNV	G	0	0	0	35	26	9	9	HVTT,N1,N2,N3,PVTT,T1,T2,T3,T4	0	0	0	15	10	5	6	HVTT,PVTT,T1,T2,T3,T4
T4	HBV_B2	1119	T	SNV	C	0	0	0	137	86	51	9	HVTT,N1,N2,N3,PVTT,T1,T2,T3,T4	0	0	0	14	9	5	5	HVTT,PVTT,T2,T3,T4
T4	HBV_B2	1131	A	SNV	G	0	0	0	139	84	55	10	HVTT,N1,N2,N3,N4,PVTT,T1,T2,T3,T4	0	0	0	13	7	6	5	HVTT,PVTT,T2,T3,T4
T4	HBV_B2	1846	T	SNV	A	0	0	0	34	22	12	8	HVTT,N3,N4,PVTT,T1,T2,T3,T4	-	-	-	-	-	-	-	-
T4	HBV_B2	1899	A	SNV	G	0	0	0	43	26	17	10	HVTT,N1,N2,N3,N4,PVTT,T1,T2,T3,T4	0	0	0	6	4	2	6	HVTT,PVTT,T1,T2,T3,T4
T4	HBV_B2	2003	A	SNV	T	0	0	0	33	18	15	10	HVTT,N1,N2,N3,N4,PVTT,T1,T2,T3,T4	0	0	0	13	6	7	6	HVTT,PVTT,T1,T2,T3,T4
T4	HBV_B2	2012	T	SNV	C	0	0	0	34	19	15	10	HVTT,N1,N2,N3,N4,PVTT,T1,T2,T3,T4	0	0	0	13	6	7	6	HVTT,PVTT,T1,T2,T3,T4
T4	HBV_B2	2078	C	SNV	G	0	0	0	39	26	13	10	HVTT,N1,N2,N3,N4,PVTT,T1,T2,T3,T4	0	0	0	10	3	7	6	HVTT,PVTT,T1,T2,T3,T4
T4	HBV_B2	2136	A	SNV	C	0	0	0	66	43	23	10	HVTT,N1,N2,N3,N4,PVTT,T1,T2,T3,T4	0	0	0	14	7	7	6	HVTT,PVTT,T1,T2,T3,T4
T4	HBV_B2	2137	G	SNV	A	0	0	0	66	42	24	10	HVTT,N1,N2,N3,N4,PVTT,T1,T2,T3,T4	0	0	0	14	7	7	6	HVTT,PVTT,T1,T2,T3,T4
T4	HBV_B2	2149	T	SNV	A	0	0	0	73	46	27	10	HVTT,N1,N2,N3,N4,PVTT,T1,T2,T3,T4	0	0	0	14	6	8	7	HVTT,N3,PVTT,T1,T2,T3,T4
T4	HBV_B2	2159	G	SNV	A	0	0	0	75	45	30	10	HVTT,N1,N2,N3,N4,PVTT,T1,T2,T3,T4	0	0	0	15	7	8	7	HVTT,N3,PVTT,T1,T2,T3,T4
T4	HBV_B2	2189	C	SNV	A	0	0	0	73	49	24	10	HVTT,N1,N2,N3,N4,PVTT,T1,T2,T3,T4	0	0	0	19	9	10	7	HVTT,N3,PVTT,T1,T2,T3,T4
T4	HBV_B2	2198	T	SNV	C	0	0	0	75	51	24	10	HVTT,N1,N2,N3,N4,PVTT,T1,T2,T3,T4	0	0	0	19	9	10	7	HVTT,N3,PVTT,T1,T2,T3,T4
T4	HBV_B2	2233	A	SNV	G	0	0	0	84	54	30	10	HVTT,N1,N2,N3,N4,PVTT,T1,T2,T3,T4	0	0	0	19	10	9	7	HVTT,N3,PVTT,T1,T2,T3,T4
T4	HBV_B2	2242	G	SNV	T	0	0	0	80	52	28	10	HVTT,N1,N2,N3,N4,PVTT,T1,T2,T3,T4	0	0	0	19	10	9	7	HVTT,N3,PVTT,T1,T2,T3,T4
T4	HBV_B2	2534	A	SNV	T	0	0	0	31	20	11	10	HVTT,N1,N2,N3,N4,PVTT,T1,T2,T3,T4	0	0	0	6	2	4	6	HVTT,PVTT,T1,T2,T3,T4
T4	HBV_B2	2572	C	SNV	T	0	0	0	36	23	13	8	HVTT,N1,N3,PVTT,T1,T2,T3,T4	0	0	0	8	3	5	6	HVTT,PVTT,T1,T2,T3,T4
T4	HBV_B2	2699	G	SNV	T	0	0	0	28	14	14	9	HVTT,N1,N2,N3,PVTT,T1,T2,T3,T4	-	-	-	-	-	-	-	-
T4	HBV_B2	2702	C	SNV	T	0	0	0	27	13	14	9	HVTT,N1,N2,N3,PVTT,T1,T2,T3,T4	-	-	-	-	-	-	-	-
T4	HBV_B2	2711	A	SNV	G	0	0	0	25	14	11	9	HVTT,N1,N2,N3,PVTT,T1,T2,T3,T4	-	-	-	-	-	-	-	-
T4	HBV_B2	2712	T	SNV	C	0	0	0	26	15	11	9	HVTT,N1,N2,N3,PVTT,T1,T2,T3,T4	-	-	-	-	-	-	-	-
T4	HBV_B2	2721	G	SNV	A	0	0	0	26	14	12	10	HVTT,N1,N2,N3,N4,PVTT,T1,T2,T3,T4	0	0	0	8	6	2	5	HVTT,PVTT,T2,T3,T4
T4	HBV_B2	2732	C	SNV	T	0	0	0	24	15	9	9	HVTT,N1,N3,N4,PVTT,T1,T2,T3,T4	0	0	0	9	6	3	6	HVTT,PVTT,T1,T2,T3,T4
T4	HBV_B2	2739	C	SNV	A	0	0	0	23	14	9	9	HVTT,N1,N3,N4,PVTT,T1,T2,T3,T4	0	0	0	9	6	3	6	HVTT,PVTT,T1,T2,T3,T4
T4	HBV_B2	2771	G	SNV	T	0	0	0	24	17	7	10	HVTT,N1,N2,N3,N4,PVTT,T1,T2,T3,T4	0	0	0	8	5	3	6	HVTT,PVTT,T1,T2,T3,T4
T4	HBV_B2	2804	T	SNV	C	0	0	0	35	27	8	10	HVTT,N1,N2,N3,N4,PVTT,T1,T2,T3,T4	0	0	0	8	3	5	6	HVTT,PVTT,T1,T2,T3,T4
T4	HBV_B2	2988	A	SNV	G	0	0	0	42	26	16	10	HVTT,N1,N2,N3,N4,PVTT,T1,T2,T3,T4	0	0	0	10	6	4	6	HVTT,PVTT,T1,T2,T3,T4
T4	HBV_B2	3039	G	SNV	A	0	0	0	44	26	18	10	HVTT,N1,N2,N3,N4,PVTT,T1,T2,T3,T4	0	0	0	8	5	3	6	HVTT,PVTT,T1,T2,T3,T4
T4	HBV_B2	3097	C	SNV	A	0	0	0	37	18	19	9	HVTT,N2,N3,N4,PVTT,T1,T2,T3,T4	-	-	-	-	-	-	-	-
T4	HBV_B2	3109	A	SNV	G	0	0	0	35	19	16	9	HVTT,N2,N3,N4,PVTT,T1,T2,T3,T4	-	-	-	-	-	-	-	-
T4	HBV_B2	3118	G	SNV	A	0	0	0	33	18	15	10	HVTT,N1,N2,N3,N4,PVTT,T1,T2,T3,T4	-	-	-	-	-	-	-	-
T4	HBV_B2	3169	T	SNV	A	0	0	0	32	20	12	10	HVTT,N1,N2,N3,N4,PVTT,T1,T2,T3,T4	0	0	0	5	2	3	5	HVTT,PVTT,T2,T3,T4
T4	HBV_B2	3189	A	SNV	C	0	0	0	34	24	10	9	HVTT,N2,N3,N4,PVTT,T1,T2,T3,T4	0	0	0	5	2	3	6	HVTT,PVTT,T1,T2,T3,T4
T4	HBV_B2	3198	T	SNV	G	0	0	0	36	24	12	10	HVTT,N1,N2,N3,N4,PVTT,T1,T2,T3,T4	0	0	0	5	2	3	6	HVTT,PVTT,T1,T2,T3,T4
HVTT	HBV_B2	20	A	SNV	G	0	0	0	68	40	28	10	HVTT,N1,N2,N3,N4,PVTT,T1,T2,T3,T4	0	0	0	13	5	8	5	HVTT,PVTT,T1,T2,T3
HVTT	HBV_B2	22	T	SNV	A	0	0	0	67	39	28	10	HVTT,N1,N2,N3,N4,PVTT,T1,T2,T3,T4	0	0	0	12	5	7	5	HVTT,PVTT,T1,T2,T3
HVTT	HBV_B2	31	T	SNV	G	0	0	0	71	36	35	10	HVTT,N1,N2,N3,N4,PVTT,T1,T2,T3,T4	0	0	0	12	5	7	5	HVTT,PVTT,T1,T2,T3
HVTT	HBV_B2	53	C	SNV	T	0	0	0	69	32	37	9	HVTT,N1,N2,N3,PVTT,T1,T2,T3,T4	0	0	0	10	5	5	5	HVTT,PVTT,T1,T2,T3
HVTT	HBV_B2	165	T	SNV	C	0	0	0	49	33	16	10	HVTT,N1,N2,N3,N4,PVTT,T1,T2,T3,T4	0	0	0	11	5	6	6	HVTT,PVTT,T1,T2,T3,T4
HVTT	HBV_B2	167	G	SNV	A	0	0	0	49	33	16	10	HVTT,N1,N2,N3,N4,PVTT,T1,T2,T3,T4	0	0	0	10	5	5	6	HVTT,PVTT,T1,T2,T3,T4
HVTT	HBV_B2	216	C	SNV	T	0	0	0	57	36	21	10	HVTT,N1,N2,N3,N4,PVTT,T1,T2,T3,T4	0	0	0	11	4	7	6	HVTT,PVTT,T1,T2,T3,T4
HVTT	HBV_B2	273	G	SNV	A	0	0	0	56	37	19	10	HVTT,N1,N2,N3,N4,PVTT,T1,T2,T3,T4	0	0	0	10	5	5	5	HVTT,PVTT,T1,T2,T3
HVTT	HBV_B2	346	T	SNV	C	0	0	0	57	39	18	9	HVTT,N2,N3,N4,PVTT,T1,T2,T3,T4	0	0	0	7	2	5	5	HVTT,PVTT,T1,T2,T3
HVTT	HBV_B2	381	A	SNV	G	0	0	0	62	37	25	9	HVTT,N2,N3,N4,PVTT,T1,T2,T3,T4	0	0	0	7	3	4	5	HVTT,PVTT,T1,T2,T3
HVTT	HBV_B2	392	C	SNV	T	0	0	0	67	44	23	9	HVTT,N2,N3,N4,PVTT,T1,T2,T3,T4	0	0	0	7	3	4	5	HVTT,PVTT,T1,T2,T3
HVTT	HBV_B2	481	A	SNV	C	0	0	0	58	34	24	9	HVTT,N2,N3,N4,PVTT,T1,T2,T3,T4	0	0	0	15	11	4	7	HVTT,N3,PVTT,T1,T2,T3,T4
HVTT	HBV_B2	562	C	SNV	A	0	0	0	59	45	14	10	HVTT,N1,N2,N3,N4,PVTT,T1,T2,T3,T4	0	0	0	21	15	6	5	HVTT,PVTT,T1,T2,T3
HVTT	HBV_B2	598	T	SNV	C	0	0	0	62	41	21	10	HVTT,N1,N2,N3,N4,PVTT,T1,T2,T3,T4	0	0	0	17	12	5	5	HVTT,PVTT,T1,T2,T3
HVTT	HBV_B2	636	T	SNV	A	0	0	0	55	32	23	10	HVTT,N1,N2,N3,N4,PVTT,T1,T2,T3,T4	0	0	0	12	9	3	6	HVTT,PVTT,T1,T2,T3,T4
HVTT	HBV_B2	645	A	SNV	G	0	0	0	54	32	22	10	HVTT,N1,N2,N3,N4,PVTT,T1,T2,T3,T4	0	0	0	13	9	4	6	HVTT,PVTT,T1,T2,T3,T4
HVTT	HBV_B2	753	T	SNV	A	0	0	0	55	35	20	7	HVTT,N3,PVTT,T1,T2,T3,T4	0	0	0	18	6	12	6	HVTT,PVTT,T1,T2,T3,T4
HVTT	HBV_B2	870	C	SNV	T	0	0	0	45	24	21	7	HVTT,N3,PVTT,T1,T2,T3,T4	0	0	0	14	7	7	6	HVTT,PVTT,T1,T2,T3,T4
HVTT	HBV_B2	912	A	SNV	G	0	0	0	46	30	16	9	HVTT,N1,N2,N3,PVTT,T1,T2,T3,T4	0	0	0	11	6	5	6	HVTT,PVTT,T1,T2,T3,T4
HVTT	HBV_B2	918	A	SNV	C	0	0	0	53	35	18	10	HVTT,N1,N2,N3,N4,PVTT,T1,T2,T3,T4	0	0	0	11	6	5	6	HVTT,PVTT,T1,T2,T3,T4
HVTT	HBV_B2	949	A	SNV	C	0	0	0	57	43	14	10	HVTT,N1,N2,N3,N4,PVTT,T1,T2,T3,T4	0	0	0	11	7	4	6	HVTT,PVTT,T1,T2,T3,T4

HVTT	HBV_B2	951	A	SNV	G	0	0	0	59	45	14	10	HVTT,N1,N2,N3,N4,PVTT,T1,T2,T3,T4	0	0	0	11	7	4	6	HVTT,PVTT,T1,T2,T3,T4
HVTT	HBV_B2	967	A	SNV	C	0	0	0	62	45	17	9	HVTT,N2,N3,N4,PVTT,T1,T2,T3,T4	0	0	0	11	7	4	6	HVTT,PVTT,T1,T2,T3,T4
HVTT	HBV_B2	996	A	SNV	C	0	0	0	66	48	18	9	HVTT,N2,N3,N4,PVTT,T1,T2,T3,T4	0	0	0	10	6	4	6	HVTT,PVTT,T1,T2,T3,T4
HVTT	HBV_B2	1104	A	SNV	C	0	0	0	59	31	28	9	HVTT,N1,N2,N3,PVTT,T1,T2,T3,T4	0	0	0	13	6	7	6	HVTT,PVTT,T1,T2,T3,T4
HVTT	HBV_B2	1106	C	SNV	T	0	0	0	59	32	27	9	HVTT,N1,N2,N3,PVTT,T1,T2,T3,T4	0	0	0	14	6	8	6	HVTT,PVTT,T1,T2,T3,T4
HVTT	HBV_B2	1114	A	SNV	G	0	0	0	127	98	29	9	HVTT,N1,N2,N3,PVTT,T1,T2,T3,T4	0	0	0	17	8	9	6	HVTT,PVTT,T1,T2,T3,T4
HVTT	HBV_B2	1119	T	SNV	C	0	0	0	303	190	113	9	HVTT,N1,N2,N3,PVTT,T1,T2,T3,T4	0	0	0	17	8	9	5	HVTT,PVTT,T2,T3,T4
HVTT	HBV_B2	1131	A	SNV	G	0	0	0	501	374	127	10	HVTT,N1,N2,N3,N4,PVTT,T1,T2,T3,T4	0	0	0	17	8	9	5	HVTT,PVTT,T2,T3,T4
HVTT	HBV_B2	1846	T	SNV	A	0	0	0	63	42	21	8	HVTT,N3,N4,PVTT,T1,T2,T3,T4	0	0	0	7	3	4	5	HVTT,PVTT,T1,T2,T3
HVTT	HBV_B2	1899	A	SNV	G	0	0	0	66	34	32	10	HVTT,N1,N2,N3,N4,PVTT,T1,T2,T3,T4	0	0	0	6	2	4	6	HVTT,PVTT,T1,T2,T3,T4
HVTT	HBV_B2	2003	A	SNV	T	0	0	0	54	33	21	10	HVTT,N1,N2,N3,N4,PVTT,T1,T2,T3,T4	0	0	0	7	4	3	6	HVTT,PVTT,T1,T2,T3,T4
HVTT	HBV_B2	2012	T	SNV	C	0	0	0	57	35	22	10	HVTT,N1,N2,N3,N4,PVTT,T1,T2,T3,T4	0	0	0	7	4	3	6	HVTT,PVTT,T1,T2,T3,T4
HVTT	HBV_B2	2078	C	SNV	G	0	0	0	53	30	23	10	HVTT,N1,N2,N3,N4,PVTT,T1,T2,T3,T4	0	0	0	5	2	3	6	HVTT,PVTT,T1,T2,T3,T4
HVTT	HBV_B2	2136	A	SNV	C	0	0	0	85	58	27	10	HVTT,N1,N2,N3,N4,PVTT,T1,T2,T3,T4	0	0	0	14	6	8	6	HVTT,PVTT,T1,T2,T3,T4
HVTT	HBV_B2	2137	G	SNV	A	0	0	0	85	57	28	10	HVTT,N1,N2,N3,N4,PVTT,T1,T2,T3,T4	0	0	0	14	6	8	6	HVTT,PVTT,T1,T2,T3,T4
HVTT	HBV_B2	2149	T	SNV	A	0	0	0	97	61	36	10	HVTT,N1,N2,N3,N4,PVTT,T1,T2,T3,T4	0	0	0	16	7	9	7	HVTT,N3,PVTT,T1,T2,T3,T4
HVTT	HBV_B2	2159	G	SNV	A	0	0	0	99	65	34	10	HVTT,N1,N2,N3,N4,PVTT,T1,T2,T3,T4	0	0	0	16	8	8	7	HVTT,N3,PVTT,T1,T2,T3,T4
HVTT	HBV_B2	2189	C	SNV	A	0	0	0	103	70	33	10	HVTT,N1,N2,N3,N4,PVTT,T1,T2,T3,T4	0	0	0	21	11	10	7	HVTT,N3,PVTT,T1,T2,T3,T4
HVTT	HBV_B2	2198	T	SNV	C	0	0	0	109	75	34	10	HVTT,N1,N2,N3,N4,PVTT,T1,T2,T3,T4	0	0	0	22	11	11	7	HVTT,N3,PVTT,T1,T2,T3,T4
HVTT	HBV_B2	2233	A	SNV	G	0	0	0	119	75	44	10	HVTT,N1,N2,N3,N4,PVTT,T1,T2,T3,T4	0	0	0	20	11	9	7	HVTT,N3,PVTT,T1,T2,T3,T4
HVTT	HBV_B2	2242	G	SNV	T	0	0	0	117	72	45	10	HVTT,N1,N2,N3,N4,PVTT,T1,T2,T3,T4	0	0	0	21	12	9	7	HVTT,N3,PVTT,T1,T2,T3,T4
HVTT	HBV_B2	2534	A	SNV	T	0	0	0	39	26	13	10	HVTT,N1,N2,N3,N4,PVTT,T1,T2,T3,T4	0	0	0	10	4	6	6	HVTT,PVTT,T1,T2,T3,T4
HVTT	HBV_B2	2572	C	SNV	T	0	0	0	35	22	13	8	HVTT,N1,N3,PVTT,T1,T2,T3,T4	0	0	0	11	5	6	6	HVTT,PVTT,T1,T2,T3,T4
HVTT	HBV_B2	2699	G	SNV	T	0	0	0	53	39	14	9	HVTT,N1,N2,N3,PVTT,T1,T2,T3,T4	0	0	0	14	7	7	4	HVTT,PVTT,T2,T3
HVTT	HBV_B2	2702	C	SNV	T	0	0	0	54	37	17	9	HVTT,N1,N2,N3,PVTT,T1,T2,T3,T4	0	0	0	13	7	6	4	HVTT,PVTT,T2,T3
HVTT	HBV_B2	2711	A	SNV	G	0	0	0	57	41	16	9	HVTT,N1,N2,N3,PVTT,T1,T2,T3,T4	0	0	0	15	9	6	4	HVTT,PVTT,T2,T3
HVTT	HBV_B2	2712	T	SNV	C	0	0	0	56	40	16	9	HVTT,N1,N2,N3,PVTT,T1,T2,T3,T4	0	0	0	15	9	6	4	HVTT,PVTT,T2,T3
HVTT	HBV_B2	2721	G	SNV	A	0	0	0	59	37	22	10	HVTT,N1,N2,N3,N4,PVTT,T1,T2,T3,T4	0	0	0	14	8	6	5	HVTT,PVTT,T2,T3,T4
HVTT	HBV_B2	2732	C	SNV	T	0	0	0	57	37	20	9	HVTT,N1,N3,N4,PVTT,T1,T2,T3,T4	0	0	0	15	9	6	6	HVTT,PVTT,T1,T2,T3,T4
HVTT	HBV_B2	2739	C	SNV	A	0	0	0	57	36	21	9	HVTT,N1,N3,N4,PVTT,T1,T2,T3,T4	0	0	0	15	9	6	6	HVTT,PVTT,T1,T2,T3,T4
HVTT	HBV_B2	2771	G	SNV	T	0	0	0	59	44	15	10	HVTT,N1,N2,N3,N4,PVTT,T1,T2,T3,T4	0	0	0	13	10	3	6	HVTT,PVTT,T1,T2,T3,T4
HVTT	HBV_B2	2804	T	SNV	C	0	0	0	65	41	24	10	HVTT,N1,N2,N3,N4,PVTT,T1,T2,T3,T4	0	0	0	11	8	3	6	HVTT,PVTT,T1,T2,T3,T4
HVTT	HBV_B2	2988	A	SNV	G	0	0	0	70	45	25	10	HVTT,N1,N2,N3,N4,PVTT,T1,T2,T3,T4	0	0	0	13	5	8	6	HVTT,PVTT,T1,T2,T3,T4
HVTT	HBV_B2	3039	G	SNV	A	0	0	0	69	39	30	10	HVTT,N1,N2,N3,N4,PVTT,T1,T2,T3,T4	0	0	0	10	5	5	6	HVTT,PVTT,T1,T2,T3,T4
HVTT	HBV_B2	3097	C	SNV	A	0	0	0	62	37	25	9	HVTT,N2,N3,N4,PVTT,T1,T2,T3,T4	0	0	0	6	2	4	5	HVTT,PVTT,T1,T2,T3
HVTT	HBV_B2	3109	A	SNV	G	0	0	0	61	37	24	9	HVTT,N2,N3,N4,PVTT,T1,T2,T3,T4	0	0	0	6	3	3	5	HVTT,PVTT,T1,T2,T3
HVTT	HBV_B2	3118	G	SNV	A	0	0	0	56	33	23	10	HVTT,N1,N2,N3,N4,PVTT,T1,T2,T3,T4	0	0	0	5	2	3	5	HVTT,PVTT,T1,T2,T3
HVTT	HBV_B2	3169	T	SNV	A	0	0	0	63	42	21	10	HVTT,N1,N2,N3,N4,PVTT,T1,T2,T3,T4	0	0	0	11	4	7	5	HVTT,PVTT,T2,T3,T4
HVTT	HBV_B2	3189	A	SNV	C	0	0	0	68	46	22	9	HVTT,N2,N3,N4,PVTT,T1,T2,T3,T4	0	0	0	12	5	7	6	HVTT,PVTT,T1,T2,T3,T4
HVTT	HBV_B2	3198	T	SNV	G	0	0	0	70	43	27	10	HVTT,N1,N2,N3,N4,PVTT,T1,T2,T3,T4	0	0	0	13	5	8	6	HVTT,PVTT,T1,T2,T3,T4
PVTT	HBV_B2	20	A	SNV	G	0	0	0	68	40	28	10	HVTT,N1,N2,N3,N4,PVTT,T1,T2,T3,T4	0	0	0	17	8	9	5	HVTT,PVTT,T1,T2,T3
PVTT	HBV_B2	22	T	SNV	A	0	0	0	73	43	30	10	HVTT,N1,N2,N3,N4,PVTT,T1,T2,T3,T4	0	0	0	18	9	9	5	HVTT,PVTT,T1,T2,T3
PVTT	HBV_B2	31	T	SNV	G	0	0	0	77	46	31	10	HVTT,N1,N2,N3,N4,PVTT,T1,T2,T3,T4	0	0	0	16	8	8	5	HVTT,PVTT,T1,T2,T3
PVTT	HBV_B2	53	C	SNV	T	0	0	0	81	47	34	9	HVTT,N1,N2,N3,PVTT,T1,T2,T3,T4	0	0	0	18	7	11	5	HVTT,PVTT,T1,T2,T3
PVTT	HBV_B2	165	T	SNV	C	0	0	0	70	34	36	10	HVTT,N1,N2,N3,N4,PVTT,T1,T2,T3,T4	0	0	0	13	4	9	6	HVTT,PVTT,T1,T2,T3,T4
PVTT	HBV_B2	167	G	SNV	A	0	0	0	72	36	36	10	HVTT,N1,N2,N3,N4,PVTT,T1,T2,T3,T4	0	0	0	13	4	9	6	HVTT,PVTT,T1,T2,T3,T4
PVTT	HBV_B2	216	C	SNV	T	0	0	0	57	38	19	10	HVTT,N1,N2,N3,N4,PVTT,T1,T2,T3,T4	0	0	0	12	4	8	6	HVTT,PVTT,T1,T2,T3,T4
PVTT	HBV_B2	273	G	SNV	A	0	0	0	63	39	24	10	HVTT,N1,N2,N3,N4,PVTT,T1,T2,T3,T4	0	0	0	11	5	6	5	HVTT,PVTT,T1,T2,T3
PVTT	HBV_B2	346	T	SNV	C	0	0	0	60	35	25	9	HVTT,N2,N3,N4,PVTT,T1,T2,T3,T4	0	0	0	12	9	3	5	HVTT,PVTT,T1,T2,T3
PVTT	HBV_B2	381	A	SNV	G	0	0	0	65	33	32	9	HVTT,N2,N3,N4,PVTT,T1,T2,T3,T4	0	0	0	13	9	4	5	HVTT,PVTT,T1,T2,T3
PVTT	HBV_B2	392	C	SNV	T	0	0	0	62	31	31	9	HVTT,N2,N3,N4,PVTT,T1,T2,T3,T4	0	0	0	14	10	4	5	HVTT,PVTT,T1,T2,T3
PVTT	HBV_B2	481	A	SNV	C	0	0	0	65	46	19	9	HVTT,N2,N3,N4,PVTT,T1,T2,T3,T4	0	0	0	20	15	5	7	HVTT,N3,PVTT,T1,T2,T3,T4
PVTT	HBV_B2	562	C	SNV	A	0	0	0	71	41	30	10	HVTT,N1,N2,N3,N4,PVTT,T1,T2,T3,T4	0	0	0	21	11	10	5	HVTT,PVTT,T1,T2,T3
PVTT	HBV_B2	598	T	SNV	C	0	0	0	73	47	26	10	HVTT,N1,N2,N3,N4,PVTT,T1,T2,T3,T4	0	0	0	20	9	11	5	HVTT,PVTT,T1,T2,T3
PVTT	HBV_B2	636	T	SNV	A	0	0	0	69	44	25	10	HVTT,N1,N2,N3,N4,PVTT,T1,T2,T3,T4	0	0	0	22	9	13	6	HVTT,PVTT,T1,T2,T3,T4
PVTT	HBV_B2	645	A	SNV	G	0	0	0	67	38	29	10	HVTT,N1,N2,N3,N4,PVTT,T1,T2,T3,T4	0	0	0	20	8	12	6	HVTT,PVTT,T1,T2,T3,T4
PVTT	HBV_B2	753	T	SNV	A	0	0	0	63	39	24	7	HVTT,N3,PVTT,T1,T2,T3,T4	0	0	0	16	7	9	6	HVTT,PVTT,T1,T2,T3,T4
PVTT	HBV_B2	870	C	SNV	T	0	0	0	59	31	28	7	HVTT,N3,PVTT,T1,T2,T3,T4	0	0	0	19	10	9	6	HVTT,PVTT,T1,T2,T3,T4
PVTT	HBV_B2	912	A	SNV	G	0	0	0	54	34	20	9	HVTT,N1,N2,N3,PVTT,T1,T2,T3,T4	0	0	0	19	10	9	6	HVTT,PVTT,T1,T2,T3,T4
PVTT	HBV_B2	918	A	SNV	C	0	0	0	54	32	22	10	HVTT,N1,N2,N3,N4,PVTT,T1,T2,T3,T4	0	0	0	19	10	9	6	HVTT,PVTT,T1,T2,T3,T4
PVTT	HBV_B2	949	A	SNV	C	0	0	0	56	37	19	10	HVTT,N1,N2,N3,N4,PVTT,T1,T2,T3,T4	0	0	0	17	9	8	6	HVTT,PVTT,T1,T2,T3,T4
PVTT	HBV_B2	951	A	SNV	G	0	0	0	56	36	20	10	HVTT,N1,N2,N3,N4,PVTT,T1,T2,T3,T4	0	0	0	18	10	8	6	HVTT,PVTT,T1,T2,T3,T4
PVTT	HBV_B2	967	A	SNV	C	0	0	0	56	35	21	9	HVTT,N2,N3,N4,PVTT,T1,T2,T3,T4	0	0	0	19	11	8	6	HVTT,PVTT,T1,T2,T3,T4
PVTT	HBV_B2	996	A	SNV	C	0	0	0	51	31	20	9	HVTT,N2,N3,N4,PVTT,T1,T2,T3,T4	0	0	0	17	8	9	6	HVTT,PVTT,T1,T2,T3,T4
PVTT	HBV_B2	1104	A	SNV	C	0	0	0	62	47	15	9	HVTT,N1,N2,N3,PVTT,T1,T2,T3,T4	0	0	0	20	7	13	6	HVTT,PVTT,T1,T2,T3,T4
PVTT	HBV_B2	1106	C	SNV	T	0	0	0	64	48	16	9	HVTT,N1,N2,N3,PVTT,T1,T2,T3,T4	0	0	0	20	7	13	6	HVTT,PVTT,T1,T2,T3,T4
PVTT	HBV_B2	1114	A	SNV	G	0	0	0	204	99	105	9	HVTT,N1,N2,N3,PVTT,T1,T2,T3,T4	0	0	0	18	7	11	6	HVTT,PVTT,T1,T2,T3,T4
PVTT	HBV_B2	1119	T	SNV	C	0	0	0	300	122	178	9	HVTT,N1,N2,N3,PVTT,T1,T2,T3,T4	0	0	0	18	7	11	5	HVTT,PVTT,T2,T3,T4
PVTT	HBV_B2	1131	A	SNV	G	0	0	0	530	245	285	10	HVTT,N1,N2,N3,N4,PVTT,T1,T2,T3,T4	0	0	0	17	7	10	5	HVTT,PVTT,T2,T3,T4
PVTT	HBV_B2	1846	T	SNV	A	0	0	0	59	39	20	8	HVTT,N3,N4,PVTT,T1,T2,T3,T4	0	0	0	10	1	9	5	HVTT,PVTT,T1,T2,T3
PVTT	HBV_B2	1899	A	SNV	G	0	0	0	61	33	28	10	HVTT,N1,N2,N3,N4,PVTT,T1,T2,T3,T4	0	0	0	15	2	13	6	HVTT,PVTT,T1,T2,T3,T4
PVTT	HBV_B2	2003	A	SNV	T	0	0	0	64	41	23	10	HVTT,N1,N2,N3,N4,PVTT,T1,T2,T3,T4	0	0	0	11	3	8	6	HVTT,PVTT,T1,T2,T3,T4
PVTT	HBV_B2	2012	T	SNV	C	0	0	0	62	39	23	10	HVTT,N1,N2,N3,N4,PVTT,T1,T2,T3,T4	0	0	0	10	3	7	6	HVTT,PVTT,T1,T2,T3,T4
PVTT	HBV_B2	2078	C	SNV	G	0	0	0	58	33	25	10	HVTT,N1,N2,N3,N4,PVTT,T1,T2,T3,T4	0	0	0	6	4	2	6	HVTT,PVTT,T1,T2,T3,T4
PVTT	HBV_B2	2136	A	SNV	C	0	0	0	80	49	31	10	HVTT,N1,N2,N3,N4,PVTT,T1,T2,T3,T4	0	0	0	16	9	7	6	HVTT,PVTT,T1,T2,T3,T4
PVTT	HBV_B2	2137	G	SNV	A	0	0	0	80	46	34	10	HVTT,N1,N2,N3,N4,PVTT,T1,T2,T3,T4	0	0	0	17	9	8	6	HVTT,PVTT,T1,T2,T3,T4
PVTT	HBV_B2	2149	T	SNV	A	0	0	0	88	54	34	10	HVTT,N1,N2,N3,N4,PVTT,T1,T2,T3,T4	0	0	0	17	8	9	7	HVTT,N3,PVTT,T1,T2,T3,T4
PVTT	HBV_B2	2159	G	SNV	A	0	0	0	93	60	33	10	HVTT,N1,N2,N3,N4,PVTT,T1,T2,T3,T4	0	0	0	18	9	9	7	HVTT,N3,PVTT,T1,T2,T3,T4
PVTT	HBV_B2	2189	C	SNV	A	0	0	0	96	71	25	10	HVTT,N1,N2,N3,N4,PVTT,T1,T2,T3,T4	0	0	0	21	9	12	7	HVTT,N3,PVTT,T1,T2,T3,T4
PVTT	HBV_B2	2198	T	SNV	C	0	0	0	98	71	27	10	HVTT,N1,N2,N3,N4,PVTT,T1,T2,T3,T4	0	0	0	24	11	13	7	HVTT,N3,PVTT,T1,T2,T3,T4
PVTT	HBV_B2	2233	A	SNV	G	0	0	0	105	69	36	10	HVTT,N1,N2,N3,N4,PVTT,T1,T2,T3,T4	0	0	0	24	11	13	7	HVTT,N3,PVTT,T1,T2,T3,T4
PVTT	HBV_B2	2242	G	SNV	T	0	0	0	108	71	37	10	HVTT,N1,N2,N3,N4,PVTT,T1,T2,T3,T4	0	0	0	24	11	13	7	HVTT,N3,PVTT,T1,T2,T3,T4
PVTT	HBV_B2	2534	A	SNV	T	0	0	0	59	34	25	10	HVTT,N1,N2,N3,N4,PVTT,T1,T2,T3,T4	0	0	0	9	5	4	6	HVTT,PVTT,T1,T2,T3,T4
PVTT	HBV_B2	2572	C	SNV	T	0	0	0	56	38	18	8	HVTT,N1,N3,PVTT,T1,T2,T3,T4	0	0	0	16	12	4	6	HVTT,PVTT,T1,T2,T3,T4
PVTT	HBV_B2	2699	G	SNV	T	0	0	0	55	30	25	9	HVTT,N1,N2,N3,PVTT,T1,T2,T3,T4	0	0	0	20	11	9	4	HVTT,PVTT,T2,T3
PVTT	HBV_B2	2702	C	SNV	T	0	0	0	56	29	27	9	HVTT,N1,N2,N3,PVTT,T1,T2,T3,T4	0	0	0	19	10	9	4	HVTT,PVTT,T2,T3
PVTT	HBV_B2	2711	A	SNV	G	0	0	0	58	35	23	9	HVTT,N1,N2,N3,PVTT,T1,T2,T3,T4	0	0	0	16	8	8	4	HVTT,PVTT,T2,T3
PVTT	HBV_B2	2712	T	SNV	C	0	0	0	58	34	24	9	HVTT,N1,N2,N3,PVTT,T1,T2,T3,T4	0	0	0	16	8	8	4	HVTT,PVTT,T2,T3
PVTT	HBV_B2	2721	G	SNV	A	0	0	0	57	36	21	10	HVTT,N1,N2,N3,N4,PVTT,T1,T2,T3,T4	0	0	0	15	7	8	5	HVTT,PVTT,T2,T3,T4
PVTT	HBV_B2	2732	C	SNV	T	0	0	0	58	36	22	9	HVTT,N1,N3,N4,PVTT,T1,T2,T3,T4	0	0	0	20	12	8	6	HVTT,PVTT,T1,T2,T3,T4
PVTT	HBV_B2	2739	C	SNV	A	0	0	0	63	43	20	9	HVTT,N1,N3,N4,PVTT,T1,T2,T3,T4	0	0	0	19	10	9	6	HVTT,PVTT,T1,T2,T3,T4
PVTT	HBV_B2	2771	G	SNV	T	0	0	0	63	46	17	10	HVTT,N1,N2,N3,N4,PVTT,T1,T2,T3,T4	0	0	0	17	10	7	6	HVTT,PVTT,T1,T2,T3,T4
PVTT	HBV_B2	2804	T	SNV	C	0	0	0	79	56	23	10	HVTT,N1,N2,N3,N4,PVTT,T1,T2,T3,T4	0	0	0	19	10	9	6	HVTT,PVTT,T1,T2,T3,T4
PVTT	HBV_B2	2988	A	SNV	G	0	0	0	79	48	31	10	HVTT,N1,N2,N3,N4,PVTT,T1,T2,T3,T4	0	0	0	14	5	9	6	HVTT,PVTT,T1,T2,T3,T4
PVTT	HBV_B2	3039	G	SNV	A	0	0	0	84	47	37	10	HVTT,N1,N2,N3,N4,PVTT,T1,T2,T3,T4	0	0	0	13	6	7	6	HVTT,PVTT,T1,T2,T3,T4
PVTT	HBV_B2	3097	C	SNV	A	0	0	0	76	40	36	9	HVTT,N2,N3,N4,PVTT,T1,T2,T3,T4	0	0	0	14	7	7	5	HVTT,PVTT,T1,T2,T3
PVTT	HBV_B2	3109	A	SNV	G	0	0	0	75	41	34	9	HVTT,N2,N3,N4,PVTT,T1,T2,T3,T4	0	0	0	14	8	6	5	HVTT,PVTT,T1,T2,T3

PVTT	HBV_B2	3118	G	SNV	A	0	0	0	77	41	36	10	HVTT,N1,N2,N3,N4,PVTT,T1,T2,T3,T4	0	0	0	11	7	4	5	HVTT,PVTT,T1,T2,T3
PVTT	HBV_B2	3169	T	SNV	A	0	0	0	71	36	35	10	HVTT,N1,N2,N3,N4,PVTT,T1,T2,T3,T4	0	0	0	16	8	8	5	HVTT,PVTT,T2,T3,T4
PVTT	HBV_B2	3189	A	SNV	C	0	0	0	70	41	29	9	HVTT,N2,N3,N4,PVTT,T1,T2,T3,T4	0	0	0	17	9	8	6	HVTT,PVTT,T1,T2,T3,T4
PVTT	HBV_B2	3198	T	SNV	G	0	0	0	68	41	27	10	HVTT,N1,N2,N3,N4,PVTT,T1,T2,T3,T4	0	0	0	17	9	8	6	HVTT,PVTT,T1,T2,T3,T4

Table S4. VCS data summary and constructed HBV genome details of 264 single cells.
1. Whole line of "-" means no enough viral signals detected in related cell which may be single normal cell.
2. Integration and rearrangement were detected in cells with >=5X average depth of constructed HBV genome.
3. "JR" means "junction reads" supporting relative inversion or integration.
4. In "major_case" columns, one case is noted with "UC" or "C" when no good supporting reads detected.
5. "UC" means break-point has zero depth, while "C" means break-point has certain low depth.

Sample	Cell	Read	Clean	Clean	Q20	Q30	GC	Virus	Average	Mutations				Coverage						major_case (JR)			has_minor_case? (Y/N)			
ID	NO.	Length(bp)	Reads	Bases (bp)	percent	percent	percent	Type	Depth(X)	SUM	SNP	INS	DEL	>=1X	>=4X	>=10X	>=30X	>=50X	>=100X	v_inv	chr1	chr8	chr1	chr8	o_chr	virus
T1	1	150;150	19699228	1969922800	93.20%	85.11%	43.63%	HBV_B2	13.36	49	49	0	0	79.32%	79.32%	54.96%	1.59%	0.00%	0.00%	6	10	3	N	N	N	N
T1	2	150;150	11009092	1100909200	93.28%	85.11%	44.76%	HBV_B2	62.52	61	61	0	0	79.32%	79.32%	79.32%	78.63%	55.12%	4.82%	41	18	25	N	N	N	N
T1	3	150;150	17342454	1734245400	93.16%	85.00%	44.67%	HBV_B2	2.45	0	0	0	0	26.97%	5.72%	0.00%	0.00%	0.00%	0.00%	-	-	-	-	-	-	-
T1	4	150;150	11547700	1154770000	93.10%	84.88%	44.68%	HBV_B2	174.23	61	61	0	0	79.35%	79.32%	79.32%	79.32%	79.32%	76.86%	66	63	51	N	N	N	N
T1	5	150;150	17771536	1777153600	93.50%	85.49%	43.54%	-	-	-	-	-	-	-	-	-	-	-	-	-	-	-	-	-	-	-
T1	6	150;150	18117530	1811753000	93.36%	85.27%	44.09%	HBV_B2	29.69	61	61	0	0	79.32%	79.32%	79.32%	28.68%	4.92%	0.00%	18	10	8	N	N	N	N
T1	10	150;150	16553722	1655372200	93.48%	85.48%	43.00%	HBV_B2	7.73	36	36	0	0	79.32%	70.54%	24.79%	0.00%	0.00%	0.00%	4	6	4	N	N	N	N
T1	11	150;150	2853428	285342800	93.03%	84.80%	44.56%	HBV_B2	3.27	10	10	0	0	76.52%	29.24%	0.12%	0.00%	0.00%	0.00%	-	-	-	-	-	-	-
T1	13	150;150	15149448	1514944800	93.16%	85.05%	44.53%	HBV_B2	1.79	0	0	0	0	44.76%	3.98%	0.00%	0.00%	0.00%	0.00%	-	-	-	-	-	-	-
T1	16	150;150	14755526	1475552600	93.04%	84.81%	44.02%	-	-	-	-	-	-	-	-	-	-	-	-	-	-	-	-	-	-	-
T1	18	150;150	16477878	1647787800	92.89%	84.63%	45.46%	HBV_B2	1.64	0	0	0	0	31.17%	0.00%	0.00%	0.00%	0.00%	0.00%	-	-	-	-	-	-	-
T1	21	150;150	13455284	1345528400	93.40%	85.44%	43.67%	***HBV_B1***	1.27	0	0	0	0	34.23%	0.00%	0.00%	0.00%	0.00%	0.00%	-	-	-	-	-	-	-
T1	22	150;150	13438736	1343873600	93.43%	85.51%	42.85%	HBV_B2	7.16	30	30	0	0	79.32%	63.02%	11.20%	1.25%	0.99%	0.00%	5	2	2	N	N	N	N
T1	23	150;150	12861442	1286144200	93.72%	85.94%	42.01%	HBV_B2	16.33	59	59	0	0	79.32%	79.32%	68.43%	2.98%	0.78%	0.00%	10	9	3	N	N	N	N
T1	24	150;150	14843194	1484319400	93.69%	85.86%	42.56%	HBV_B2	2.89	2	2	0	0	64.57%	17.85%	0.00%	0.00%	0.00%	0.00%	-	-	-	-	-	-	-
T1	25	150;150	11767290	1176729000	93.62%	85.85%	41.82%	HBV_B2	59.18	61	61	0	0	79.35%	79.32%	79.32%	78.85%	57.98%	4.17%	33	26	23	N	N	N	N
T1	27	150;150	14245634	1424563400	93.78%	86.04%	42.02%	HBV_B2	15.76	50	50	0	0	79.32%	79.01%	66.03%	2.40%	0.00%	0.00%	9	3	4	N	N	N	N
T1	29	150;150	12528160	1252816000	93.85%	86.13%	42.45%	HBV_B2	437.25	61	61	0	0	79.38%	79.31%	79.31%	79.31%	79.31%	79.31%	95	111	109	Y	N	N	N
T1	30	150;150	12514318	1251431800	93.22%	85.09%	43.11%	HBV_B2	143.89	61	61	0	0	79.35%	79.32%	79.32%	79.32%	79.32%	69.43%	63	52	53	Y	N	N	N
T1	31	150;150	13941966	1394196600	93.85%	86.17%	41.79%	HBV_B2	13.58	46	46	0	0	79.35%	78.23%	58.16%	1.18%	1.15%	0.00%	12	3	6	N	N	N	N
T1	32	150;150	15517330	1551733000	93.64%	85.82%	42.49%	HBV_B2	1.5	0	0	0	0	36.58%	1.34%	0.00%	0.00%	0.00%	0.00%	-	-	-	-	-	-	-
T1	33	150;150	14392854	1439285400	93.45%	85.59%	42.62%	HBV_B2	1.69	0	0	0	0	30.23%	2.52%	0.00%	0.00%	0.00%	0.00%	-	-	-	-	-	-	-
T1	34	150;150	13914106	1391410600	93.93%	86.18%	41.86%	HBV_B2	1.31	0	0	0	0	26.94%	0.00%	0.00%	0.00%	0.00%	0.00%	-	-	-	-	-	-	-
T1	37	150;150	13342904	1334290400	94.07%	86.45%	41.97%	HBV_B2	9.84	39	39	0	0	79.32%	75.12%	40.22%	0.99%	0.00%	0.00%	7	3	2	N	N	N	N
T1	38	150;150	11414014	1141401400	93.82%	86.10%	42.29%	HBV_B2	6.75	28	28	0	0	79.32%	58.60%	12.19%	1.37%	0.00%	0.00%	5	2	4	N	N	N	N
T1	39	150;150	12951936	1295193600	93.78%	86.06%	42.14%	***HBV_B5***	1.96	0	0	0	0	37.42%	0.93%	0.00%	0.00%	0.00%	0.00%	-	-	-	-	-	-	-
T1	41	150;150	13441290	1344129000	93.59%	85.74%	43.40%	HBV_B2	1.56	0	0	0	0	32.32%	0.50%	0.00%	0.00%	0.00%	0.00%	-	-	-	-	-	-	-
T1	42	150;150	17902144	1790214400	96.43%	89.88%	41.31%	HBV_B2	2.67	2	2	0	0	69.64%	20.22%	0.00%	0.00%	0.00%	0.00%	-	-	-	-	-	-	-
T1	43	150;150	19522858	1952285800	96.30%	89.58%	42.78%	HBV_B2	8.18	28	28	0	0	79.22%	67.03%	31.13%	0.00%	0.00%	0.00%	1	5	1	N	N	N	N
T1	44	150;150	20011668	2001166800	96.37%	89.75%	42.09%	HBV_B2	3.57	2	2	0	0	70.98%	31.54%	1.68%	0.00%	0.00%	0.00%	-	-	-	-	-	-	-
T1	45	150;150	14903022	1490302200	96.29%	89.52%	42.35%	HBV_B2	54.42	61	61	0	0	79.32%	79.32%	79.32%	73.99%	41.09%	5.04%	30	26	17	N	N	N	N
T1	46	150;150	20809500	2080950000	96.28%	89.58%	41.99%	HBV_B2	10.2	43	43	0	0	79.32%	72.38%	43.67%	0.00%	0.00%	0.00%	7	5	3	N	N	N	N
T1	47	150;150	20810828	2081082800	96.25%	89.50%	42.31%	HBV_B2	4.29	8	8	0	0	75.37%	45.13%	0.00%	0.00%	0.00%	0.00%	-	-	-	-	-	-	-
T1	48	150;150	19941440	1994144000	96.41%	89.81%	41.56%	HBV_B2	418.49	61	61	0	0	79.38%	79.31%	79.31%	79.31%	79.31%	79.31%	87	100	98	N	N	N	N
T1	49	150;150	19208808	1920880800	96.39%	89.82%	40.90%	***HBV_B4***	2.53	4	4	0	0	63.64%	12.54%	0.00%	0.00%	0.00%	0.00%	-	-	-	-	-	-	-
T1	50	150;150	25123078	2512307800	96.43%	89.86%	41.46%	HBV_B2	131.17	61	61	0	0	79.32%	79.32%	79.32%	79.32%	79.28%	61.74%	63	45	48	N	N	N	N
T1	53	150;150	21447694	2144769400	96.30%	89.56%	42.13%	HBV_B2	4.86	10	10	0	0	77.54%	52.97%	4.51%	0.00%	0.00%	0.00%	-	-	-	-	-	-	-
T1	54	150;150	21587242	2158724200	96.35%	89.63%	42.44%	HBV_B2	5.59	23	23	0	0	78.54%	55.93%	9.14%	0.00%	0.00%	0.00%	8	2	2	N	N	N	N
T1	55	150;150	20803786	2080378600	96.34%	89.61%	42.57%	HBV_B2	4.37	14	14	0	0	75.12%	37.08%	7.56%	0.00%	0.00%	0.00%	-	-	-	-	-	-	-
T1	56	150;150	23461716	2346171600	96.27%	89.45%	42.30%	HBV_B2	100.5	61	61	0	0	79.32%	79.32%	79.32%	79.32%	72.41%	33.15%	54	42	23	N	N	N	N
T1	58	150;150	28838870	2883887000	96.38%	89.73%	41.68%	HBV_B2	1271.12	61	61	0	0	79.38%	79.38%	79.35%	79.31%	79.31%	79.31%	100	134	128	Y	N	N	N
T1	59	150;150	20874124	2087412400	96.55%	90.06%	41.12%	HBV_B2	2.89	2	2	0	0	70.36%	19.44%	0.00%	0.00%	0.00%	0.00%	-	-	-	-	-	-	-
T1	60	150;150	22107462	2210746200	96.38%	89.72%	42.11%	HBV_B2	3.6	8	8	0	0	72.81%	28.43%	2.21%	0.00%	0.00%	0.00%	-	-	-	-	-	-	-
T1	66	150;150	2017128	201712800	96.77%	90.73%	44.56%	HBV_B2	1.5	0	0	0	0	46.75%	0.50%	0.00%	0.00%	0.00%	0.00%	-	-	-	-	-	-	-
T1	67	150;150	23980212	2398021200	96.08%	89.09%	43.74%	HBV_B2	8.05	31	31	0	0	79.32%	70.88%	28.34%	0.00%	0.00%	0.00%	4	5	9	N	N	N	N
T2	3	150;150	13137352	1313735200	98.10%	94.00%	39.14%	HBV_B2	146.7	57	57	0	0	79.38%	79.31%	72.72%	70.48%	68.68%	62.89%	69	25	61	N	N	N	N
T2	13	150;150	14173340	1417334000	97.77%	93.19%	41.60%	HBV_B2	12.73	47	47	0	0	79.04%	75.37%	46.31%	0.62%	0.00%	0.00%	11	3	5	N	N	N	N
T2	16	150;150	17679256	1767925600	97.77%	93.25%	41.47%	HBV_B2	14.61	47	47	0	0	79.32%	73.84%	56.36%	2.18%	0.00%	0.00%	6	4	6	N	N	N	N
T2	34	150;150	11589654	1158965400	97.05%	91.59%	46.63%	HBV_B2	13.28	46	46	0	0	79.04%	72.29%	55.55%	3.20%	0.00%	0.00%	9	3	11	N	N	N	N
T2	42	150;150	12964622	1296462200	97.07%	91.70%	45.89%	HBV_B2	208.15	61	61	0	0	79.38%	79.31%	79.31%	79.31%	79.31%	78.88%	74	73	73	N	N	N	N
T2	74	150;150	10988314	1098831400	97.77%	93.26%	40.98%	HBV_B2	10.62	44	44	0	0	79.35%	77.48%	40.59%	0.00%	0.00%	0.00%	3	5	3	N	N	N	N
T2	75	150;150	13783656	1378365600	97.70%	93.08%	41.86%	HBV_B2	12.04	38	38	0	0	76.58%	66.88%	48.61%	2.99%	0.00%	0.00%	12	1	2	N	N	N	N
T2	76	150;150	17521056	1752105600	97.63%	92.92%	41.99%	HBV_B2	14.73	49	49	0	0	76.77%	73.90%	58.35%	4.41%	0.00%	0.00%	6	4	6	N	N	N	N
T2	77	150;150	12516838	1251683800	97.73%	93.15%	41.38%	HBV_B2	12.33	48	48	0	0	79.32%	74.40%	49.39%	0.68%	0.00%	0.00%	8	1	4	N	N	N	N
T2	78	150;150	14585416	1458541600	97.65%	92.97%	41.98%	HBV_B2	26.27	55	55	0	0	79.32%	77.76%	74.03%	27.44%	2.27%	1.25%	18	10	11	N	N	N	N
T2	79	150;150	17750270	1775027000	97.98%	93.68%	39.46%	HBV_B2	10.94	33	33	0	0	79.28%	73.41%	43.42%	0.00%	0.00%	0.00%	2	8	4	N	N	N	N
T2	81	150;150	14633034	1463303400	98.07%	93.94%	38.56%	HBV_B2	10.13	37	37	0	0	78.63%	72.94%	42.34%	0.00%	0.00%	0.00%	8	2	6	N	N	N	N
T2	82	150;150	7716218	771621800	98.18%	94.19%	38.06%	HBV_B2	7.85	27	27	0	0	77.92%	70.73%	25.84%	0.00%	0.00%	0.00%	6	3	4	N	N	N	N
T2	83	150;150	11391272	1139127200	97.88%	93.43%	40.35%	HBV_B2	12.71	47	47	0	0	79.28%	74.59%	49.77%	0.00%	0.00%	0.00%	8	3	7	N	N	N	N
T2	84	150;150	12561746	1256174600	97.59%	92.84%	42.27%	HBV_B2	10.96	37	37	0	0	77.17%	72.85%	37.17%	0.00%	0.00%	0.00%	3	3	6	N	N	N	N
T2	85	150;150	10643414	1064341400	97.54%	92.68%	43.36%	HBV_B2	8.38	18	18	0	0	73.97%	64.01%	28.65%	0.00%	0.00%	0.00%	3	3	3	N	N	N	N
T2	86	150;150	7541424	754142400	97.80%	93.30%	41.32%	HBV_B2	138.56	60	60	0	0	79.38%	79.31%	77.39%	75.31%	73.87%	60.15%	67	49	40	Y	N	N	N
T2	88	150;150	9593208	959320800	97.70%	93.01%	42.57%	HBV_B2	7.99	32	32	0	0	77.70%	66.60%	27.06%	0.00%	0.00%	0.00%	2	4	4	N	N	N	N
T2	89	150;150	17203490	1720349000	97.90%	93.54%	40.08%	HBV_B2	18.13	52	52	0	0	79.00%	76.24%	64.14%	7.90%	0.03%	0.00%	10	7	9	N	N	N	N
T2	90	150;150	9100662	910066200	97.66%	92.95%	41.96%	HBV_B2	8.69	32	32	0	0	79.19%	70.58%	26.32%	0.00%	0.00%	0.00%	12	4	1	N	N	N	N
T2	91	150;150	10961088	1096108800	97.74%	93.18%	41.19%	HBV_B2	9.97	40	40	0	0	78.66%	68.12%	37.36%	1.49%	0.00%	0.00%	6	2	5	N	N	N	N
T2	93	150;150	12093242	1209324200	97.62%	92.84%	42.72%	HBV_B2	11.18	40	40	0	0	79.22%	73.59%	37.64%	1.77%	0.00%	0.00%	5	4	4	N	N	N	N
T2	94	150;150	13792192	1379219200	97.79%	93.28%	41.22%	HBV_B2	13.66	47	47	0	0	77.67%	73.87%	54.90%	3.08%	0.00%	0.00%	10	4	5	N	N	N	N
T2	95	150;150	15249666	1524966600	97.66%	92.92%	42.00%	HBV_B2	91.04	58	58	0	0	79.38%	79.35%	76.51%	72.23%	69.99%	20.15%	39	42	32	N	N	N	N
T2	96	150;150	15181512	1518151200	97.59%	92.72%	43.20%	HBV_B2	338.29	61	61	0	0	79.38%	79.31%	79.31%	75.65%	74.22%	71.26%	80	97	88	N	N	N	N
T2	97	150;150	14665414	1466541400	97.72%	93.07%	41.39%	HBV_B2	1572.12	61	61	0	0	79.38%	79.38%	79.35%	79.31%	79.31%	74.99%	98	127	128	Y	N	N	N
T2	98	150;150	17427736	1742773600	97.61%	92.78%	42.44%	HBV_B2	1914.84	61	61	0	0	79.38%	79.38%	79.35%	79.31%	79.31%	79.31%	104	134	131	Y	N	N	N
T2	99	150;150	11396278	1139627800	97.13%	91.75%	42.72%	HBV_B2	2.98	0	0	0	0	54.62%	11.07%	1.80%	0.00%	0.00%	0.00%	-	-	-	-	-	-	-
T2	101	150;150	13549586	1354958600	96.99%	91.34%	42.36%	HBV_B2	1.5	0	0	0	0	32.19%	0.00%	0.00%	0.00%	0.00%	0.00%	-	-	-	-	-	-	-
T2	102	150;150	52334822	5233482200	97.47%	92.63%	38.55%	HBV_B2	96.87	60	60	0	0	79.35%	79.10%	78.32%	74.74%	67.87%	18.63%	59	21	42	N	Y	N	N
T2	103	150;150	40578992	4057899200	97.43%	92.49%	39.87%	HBV_B2	4.33	19	19	0	0	59.56%	40.16%	0.06%	0.00%	0.00%	0.00%	-	-	-	-	-	-	-
T2	104	150;150	7837178	783717800	97.20%	91.90%	41.11%	HBV_B2	6.22	26	26	0	0	55.61%	39.50%	12.01%	0.00%	0.00%	0.00%	6	UC	5	N	N	N	N
T2	105	150;150	9657994	965799400	97.10%	91.72%	41.84%	HBV_B2	1009.88	56	56	0	0	79.00%	77.91%	76.11%	71.51%	55.00%	44.01%	99	15	127	N	Y	N	N
T2	107	150;150	10849502	1084950200	97.12%	91.75%	42.11%	HBV_B2	8.48	20	20	0	0	49.61%	37.95%	15.65%	1.09%	0.00%	0.00%	9	UC	2	N	N	N	N
T2	108	150;150	10391018	1039101800	97.26%	92.07%	41.21%	HBV_B2	1.6	0	0	0	0	30.17%	0.00%	0.00%	0.00%	0.00%	0.00%	-	-	-	-	-	-	-
T2	109	150;150	12105444	1210544400	97.09%	91.68%	42.60%	HBV_B2	3.2	3	3	0	0	63.89%	21.89%	0.15%	0.00%	0.00%	0.00%	-	-	-	-	-	-	-
T2	110	150;150	25363556	2536355600	97.30%	92.18%	40.86%	HBV_B2	421.34	61	61	0	0	79.35%	79.35%	79.32%	78.54%	75.18%	72.69%	89	104	101	N	Y	N	N
T2	111	150;150	18274768	1827476800	97.22%	91.92%	41.94%	HBV_B2	59.06	56	56	0	0	78.13%	76.74%	74.52%	63.95%	51.17%	5.66%	50	8	28	N	N	N	N
T2	112	150;150	23994332	2399433200	97.24%	91.99%	42.18%	HBV_B2	4.36	12	12	0	0	53.78%	30.57%	1.93%	0.00%	0.00%	0.00%	-	-	-	-	-	-	-
T2	114	150;150	21879496	2187949600	97.28%	92.08%	41.32%	HBV_B2	2.79	2	2	0	0	56.73%	15.02%	0.00%	0.00%	0.00%	0.00%	-	-	-	-	-	-	-
T2	115	150;150	22840498	2284049800	97.01%	91.42%	42.21%	HBV_B2	2.99	1	1	0	0	49.95%	18.20%	0.00%	0.00%	0.00%	0.00%	-	-	-	-	-	-	-
T2	117	150;150	15793346	1579334600	97.26%	92.03%	41.80%	HBV_B2	2.08	2	2	0	0	60.16%	6.88%	0.00%	0.00%	0.00%	0.00%	-	-	-	-	-	-	-
T2	118	150;150	23785164	2378516400	97.17%	91.81%	42.53%	HBV_B2	3.11	8	8	0	0	60.47%	22.33%	1.28%	0.00%	0.00%	0.00%	-	-	-	-	-	-	-
T2	119	150;150	20454868	2045486800	97.16%	91.83%	42.09%	HBV_B2	91.39	57	57	0	0	79.32%	79.32%	77.82%	74.99%	71.69%	27.59%	49	29	34	N	N	N	N
T2	120	150;150	18675460	1867546000	97.20%	91.94%	40.26%	HBV_B2	169.72	61	61	0	0	79.35%	79.35%	77.73%	74.21%	72.04%	68.15%	72	51	60	N	N	N	N
T3	13	150;150	9144992	914499200	96.57%	90.08%	49.85%	HBV_B2	7.8	22	22	0	0	79.16%	64.23%	14.80%	1.15%	1.15%	0.00%	5	3	C	N	N	N	N
T3	37	150;150	9861998	986199800	97.01%	91.43%	46.09%	HBV_B2	3.03	4	4	0	0	72.04%	24.60%	0.59%	0.00%	0.00%	0.00%	-	-	-	-	-	-	-
T3	41	150;150	8786588	878658800	96.84%	91.07%	47.79%	HBV_B2	68.94	61	61	0	0	79.38%	79.31%	79.31%	69.21%	32.72%	2.77%	37	19	13	N	N	N	N
T3	47	150;150	11712398	1171239800	96.97%	91.49%	45.67%	HBV_B2	747.27	61	61	0	0	79.38%	79.35%	79.35%	79.31%	79.31%	79.31%	101	113	112	Y	N	N	N
T3	51	150;150	10600750	1060075000	96.66%	90.25%	49.72%	HBV_B2	135.26	61	61	0	0	79.38%	79.31%	76.33%	57.20%	43.79%	22.33%	53	35	26	Y	N	N	Y
T3	53	150;150	12042108	1204210800	96.30%	89.14%	52.87%	HBV_B2	5.83	24	24	0	0	75.65%	57.54%	9.18%	0.00%	0.00%	0.00%	3	2	2	N	N	N	N
T3	58	150;150	14661092	1466109200	96.63%	90.42%	49.12%	HBV_B2	6.28	24	24	0	0	79.25%	65.72%	9.70%	0.00%	0.00%	0.00%	4	4	1	N	N	N	N
T3	62	150;150	12141234	1214123400	95.96%	88.03%	56.22%	HBV_B2	5.61	19	19	0	0	79.32%	62.33%	8.27%	0.00%	0.00%	0.00%	5	3	2	N	N	N	N
T3	70	150;150	16024010	1602401000	97.29%	92.22%	43.26%	HBV_B2	138.96	61	61	0	0	79.38%	79.31%	79.31%	79.31%	77.85%	27.31%	52	28	44	N	N	N	N
T3	72	150;150	18915576	1891557600	96.92%	91.33%	45.94%	HBV_B2	176.14	61	61	0	0	79.38%	79.35%	79.31%	79.31%	79.31%	73.31%	70	57	36	N	N	N	N
T3	73	150;150	18138922	1813892200	97.35%	92.38%	42.57%	HBV_B2	5.68	19	19	0	0	76.21%	57.70%	8.52%	0.00%	0.00%	0.00%	3	4	1	N	N	N	N
T3	74	150;150	10493154	1049315400	97.38%	92.46%	42.72%	HBV_B2	8.72	32	32	0	0	79.32%	70.54%	30.61%	0.00%	0.00%	0.00%	5	6	1	N	N	N	N
T3	75	150;150	9408544	940854400	97.34%	92.41%	42.48%	HBV_B2	330.78	61	61	0	0	79.35%	79.35%	79.32%	79.32%	79.32%	79.32%	78	84	52	N	N	N	N
T3	76	150;150	11839144	1183914400	97.27%	92.36%	41.83%	HBV_B2	4.01	5	5	0	0	74.46%	39.69%	0.34%	0.00%	0.00%	0.00%	-	-	-	-	-	-	-
T3	77	150;150	12215792	1221579200	97.50%	92.74%	41.12%	HBV_B2	3.98	9	9	0	0	73.06%	37.14%	1.93%	0.00%	0.00%	0.00%	-	-	-	-	-	-	-
T3	78	150;150	11207464	1120746400	97.06%	91.90%	42.92%	HBV_B2	4.87	12	12	0	0	70.92%	50.39%	2.58%	0.00%	0.00%	0.00%	-	-	-	-	-	-	-

T3	81	150;150	12470088	1247008800	97.50%	92.73%	41.63%	HBV_B2	4.13	12	12	0	0	69.67%	36.15%	3.76%	0.00%	0.00%	0.00%	-	-	-	-	-	-	-
T3	82	150;150	12238492	1223849200	97.50%	92.77%	40.95%	HBV_B2	5.29	21	21	0	0	71.38%	52.00%	4.54%	0.00%	0.00%	0.00%	3	UC	1	N	N	N	N
T3	84	150;150	12154088	1215408800	97.44%	92.60%	41.58%	HBV_B2	4.55	14	14	0	0	74.68%	46.13%	2.08%	0.00%	0.00%	0.00%	-	-	-	-	-	-	-
T3	85	150;150	13198192	1319819200	97.45%	92.61%	41.42%	HBV_B2	4.83	9	9	0	0	77.82%	55.67%	5.10%	0.00%	0.00%	0.00%	-	-	-	-	-	-	-
T3	86	150;150	12728660	1272866000	97.37%	92.45%	41.98%	HBV_B2	4.15	12	12	0	0	77.67%	35.14%	4.51%	0.00%	0.00%	0.00%	-	-	-	-	-	-	-
T3	87	150;150	15484558	1548455800	97.51%	92.82%	40.32%	HBV_B2	4.72	14	14	0	0	76.98%	45.66%	5.13%	0.00%	0.00%	0.00%	-	-	-	-	-	-	-
T3	88	150;150	17646762	1764676200	97.13%	92.26%	41.11%	HBV_B2	6.93	23	23	0	0	79.32%	60.72%	19.13%	0.00%	0.00%	0.00%	7	2	1	N	N	N	N
T3	89	150;150	13713592	1371359200	97.50%	92.74%	41.00%	HBV_B2	4.07	7	7	0	0	78.38%	39.32%	1.31%	0.00%	0.00%	0.00%	-	-	-	-	-	-	-
T3	91	150;150	19062998	1906299800	97.41%	92.54%	41.58%	HBV_B2	37.81	61	61	0	0	79.32%	79.32%	74.12%	48.56%	11.67%	2.18%	22	5	16	N	N	N	N
T3	92	150;150	17856264	1785626400	97.42%	92.55%	41.19%	HBV_B2	5.06	16	16	0	0	77.42%	53.65%	5.57%	0.00%	0.00%	0.00%	6	1	1	N	N	N	N
T3	94	150;150	13619430	1361943000	97.52%	92.78%	40.74%	HBV_B2	4.57	11	11	0	0	73.69%	46.13%	1.43%	0.00%	0.00%	0.00%	-	-	-	-	-	-	-
T3	95	150;150	21274456	2127445600	97.57%	92.90%	40.81%	HBV_B2	22.87	60	60	0	0	79.32%	79.32%	73.16%	19.97%	0.00%	0.00%	14	10	3	N	N	N	N
T3	96	150;150	21340344	2134034400	97.38%	92.49%	40.45%	HBV_B2	16.7	50	50	0	0	79.32%	78.07%	66.01%	3.98%	0.00%	0.00%	11	3	6	N	N	N	N
T3	97	150;150	20530846	2053084600	97.43%	92.57%	42.19%	HBV_B2	164.04	61	61	0	0	79.32%	79.32%	79.32%	79.32%	79.32%	70.11%	68	57	44	N	N	N	N
T3	98	150;150	19656684	1965668400	97.46%	92.72%	40.78%	HBV_B2	27.37	60	60	0	0	79.32%	79.32%	77.70%	33.28%	1.55%	0.00%	16	8	4	N	N	N	N
T3	99	150;150	25069374	2506937400	97.42%	92.57%	41.43%	HBV_B2	24.4	59	59	0	0	79.32%	79.32%	74.96%	26.75%	3.36%	0.00%	11	6	3	N	N	N	N
T3	100	150;150	21901156	2190115600	97.33%	92.34%	42.30%	HBV_B2	23.68	61	61	0	0	79.32%	79.32%	75.55%	17.42%	1.86%	0.00%	16	10	5	N	N	N	N
T3	101	150;150	26708684	2670868400	97.32%	92.37%	41.87%	HBV_B2	33.67	61	61	0	0	79.32%	79.32%	79.32%	43.48%	8.53%	0.00%	20	20	8	N	N	N	N
T3	102	150;150	18571238	1857123800	97.41%	92.60%	40.98%	HBV_B2	40.68	61	61	0	0	79.38%	79.31%	79.31%	56.45%	22.15%	0.00%	20	16	9	N	N	N	N
T3	105	150;150	19009118	1900911800	97.41%	92.57%	41.16%	HBV_B2	20.58	55	55	0	0	79.32%	79.32%	71.01%	17.08%	0.00%	0.00%	9	6	5	N	N	N	N
T3	107	150;150	16751124	1675112400	97.40%	92.48%	42.50%	HBV_B2	19.24	60	60	0	0	79.32%	79.32%	76.11%	6.00%	0.00%	0.00%	18	6	6	N	N	N	N
T3	108	150;150	18008504	1800850400	97.46%	92.60%	42.07%	HBV_B2	27.24	61	61	0	0	79.35%	79.32%	76.67%	30.52%	3.76%	0.00%	22	5	5	N	N	N	N
T3	111	150;150	15532348	1553234800	97.47%	92.62%	42.54%	HBV_B2	24.23	59	59	0	0	79.32%	79.32%	78.07%	26.69%	0.62%	0.00%	12	3	3	N	N	N	N
T3	113	150;150	18191618	1819161800	97.42%	92.49%	42.32%	HBV_B2	59.02	61	61	0	0	79.32%	79.32%	79.32%	69.99%	44.01%	6.78%	35	27	8	N	N	N	N
T3	115	150;150	21062168	2106216800	97.26%	92.08%	42.96%	HBV_B2	26.85	58	58	0	0	79.32%	79.32%	76.24%	29.24%	3.30%	0.00%	15	7	6	N	N	N	N
T3	116	150;150	24814692	2481469200	97.36%	92.22%	43.95%	HBV_B2	7443.47	61	61	0	0	79.38%	79.38%	79.38%	79.38%	79.38%	79.31%	106	157	151	Y	N	N	Y
T3	117	150;150	17477894	1747789400	97.45%	92.54%	43.03%	HBV_B2	26.95	59	59	0	0	79.32%	79.32%	76.98%	33.38%	3.05%	0.00%	21	6	9	N	N	N	N
T3	120	150;150	35678718	3567871800	97.20%	92.01%	42.81%	HBV_B2	39.96	61	61	0	0	79.32%	79.32%	79.32%	52.72%	18.73%	0.00%	25	13	6	N	N	N	N
T4	1	150;150	20086808	2008680800	97.74%	93.30%	39.88%	-	-	-	-	-	-	-	-	-	-	-	-	-	-	-	-	-	-	-
T4	3	150;150	13896200	1389620000	97.48%	92.64%	42.66%	-	-	-	-	-	-	-	-	-	-	-	-	-	-	-	-	-	-	-
T4	6	150;150	13429690	1342969000	97.50%	92.79%	41.87%	-	-	-	-	-	-	-	-	-	-	-	-	-	-	-	-	-	-	-
T4	7	150;150	12142618	1214261800	97.70%	93.25%	40.28%	-	-	-	-	-	-	-	-	-	-	-	-	-	-	-	-	-	-	-
T4	9	150;150	10357592	1035759200	97.54%	92.88%	40.56%	-	-	-	-	-	-	-	-	-	-	-	-	-	-	-	-	-	-	-
T4	10	150;150	16558102	1655810200	97.54%	92.89%	41.06%	-	-	-	-	-	-	-	-	-	-	-	-	-	-	-	-	-	-	-
T4	12	150;150	11983130	1198313000	97.43%	92.59%	42.57%	-	-	-	-	-	-	-	-	-	-	-	-	-	-	-	-	-	-	-
T4	17	150;150	12206512	1220651200	97.67%	93.17%	39.97%	-	-	-	-	-	-	-	-	-	-	-	-	-	-	-	-	-	-	-
T4	18	150;150	13881056	1388105600	97.56%	92.94%	40.57%	-	-	-	-	-	-	-	-	-	-	-	-	-	-	-	-	-	-	-
T4	19	150;150	12724052	1272405200	97.46%	92.64%	42.22%	-	-	-	-	-	-	-	-	-	-	-	-	-	-	-	-	-	-	-
T4	23	150;150	13450244	1345024400	97.69%	93.21%	39.91%	-	-	-	-	-	-	-	-	-	-	-	-	-	-	-	-	-	-	-
T4	24	150;150	13663036	1366303600	97.54%	92.87%	40.50%	-	-	-	-	-	-	-	-	-	-	-	-	-	-	-	-	-	-	-
T4	25	150;150	21987370	2198737000	97.66%	93.20%	39.02%	-	-	-	-	-	-	-	-	-	-	-	-	-	-	-	-	-	-	-
T4	26	150;150	12762340	1276234000	97.70%	93.25%	38.93%	-	-	-	-	-	-	-	-	-	-	-	-	-	-	-	-	-	-	-
T4	27	150;150	9594910	959491000	97.57%	92.96%	40.26%	-	-	-	-	-	-	-	-	-	-	-	-	-	-	-	-	-	-	-
T4	29	150;150	12296432	1229643200	97.47%	92.69%	42.18%	-	-	-	-	-	-	-	-	-	-	-	-	-	-	-	-	-	-	-
T4	30	150;150	10895354	1089535400	97.53%	92.82%	40.63%	-	-	-	-	-	-	-	-	-	-	-	-	-	-	-	-	-	-	-
T4	32	150;150	11053156	1105315600	97.67%	93.17%	39.57%	-	-	-	-	-	-	-	-	-	-	-	-	-	-	-	-	-	-	-
T4	33	150;150	9343158	934315800	97.56%	92.93%	40.62%	***HBV_B9***	3.48	9	9	0	0	66.72%	18.97%	2.15%	1.21%	0.00%	0.00%	-	-	-	-	-	-	-
T4	34	150;150	10094650	1009465000	97.52%	92.84%	40.82%	-	-	-	-	-	-	-	-	-	-	-	-	-	-	-	-	-	-	-
T4	35	150;150	9737550	973755000	97.50%	92.79%	40.99%	-	-	-	-	-	-	-	-	-	-	-	-	-	-	-	-	-	-	-
T4	38	150;150	10325592	1032559200	97.60%	92.99%	40.81%	HBV_B2	7.24	21	21	0	0	68.71%	52.04%	11.63%	1.15%	0.93%	0.00%	7	UC	1	N	N	N	N
T4	39	150;150	12403974	1240397400	97.53%	92.87%	40.35%	-	-	-	-	-	-	-	-	-	-	-	-	-	-	-	-	-	-	-
T4	41	150;150	14375992	1437599200	97.49%	92.75%	41.21%	-	-	-	-	-	-	-	-	-	-	-	-	-	-	-	-	-	-	-
T4	42	150;150	19133716	1913371600	97.80%	93.47%	38.46%	-	-	-	-	-	-	-	-	-	-	-	-	-	-	-	-	-	-	-
T4	43	150;150	13619280	1361928000	97.70%	93.25%	39.56%	-	-	-	-	-	-	-	-	-	-	-	-	-	-	-	-	-	-	-
T4	44	150;150	12247968	1224796800	97.61%	93.03%	39.84%	-	-	-	-	-	-	-	-	-	-	-	-	-	-	-	-	-	-	-
T4	45	150;150	19738944	1973894400	97.59%	92.99%	39.67%	HBV_B2	5.87	18	18	0	0	48.80%	30.08%	10.48%	0.00%	0.00%	0.00%	4	1	3	N	N	N	N
T4	46	150;150	15965094	1596509400	97.63%	93.06%	39.06%	HBV_B2	5.38	20	20	0	0	43.55%	28.96%	3.92%	0.00%	0.00%	0.00%	2	UC	3	N	N	N	N
T4	48	150;150	16075308	1607530800	97.71%	93.26%	38.78%	HBV_B2	4	7	7	0	0	41.40%	19.16%	4.17%	0.00%	0.00%	0.00%	-	-	-	-	-	-	-
T4	49	150;150	18847990	1884799000	97.64%	93.13%	39.69%	HBV_B2	5.51	8	8	0	0	42.77%	28.68%	5.82%	0.00%	0.00%	0.00%	7	UC	3	N	N	N	N
T4	50	150;150	18233466	1823346600	97.60%	93.01%	38.99%	HBV_B2	6.24	18	18	0	0	40.47%	28.40%	7.96%	0.00%	0.00%	0.00%	3	UC	4	N	N	N	N
T4	51	150;150	17765028	1776502800	97.72%	93.34%	39.03%	HBV_B2	36.41	49	49	0	0	70.54%	68.52%	66.22%	39.50%	9.11%	2.71%	19	10	9	N	N	N	N
T4	52	150;150	23452480	2345248000	97.76%	93.46%	37.99%	HBV_B2	142.12	60	60	0	0	79.35%	79.32%	79.01%	68.21%	63.60%	49.70%	77	16	52	N	N	N	N
T4	53	150;150	23917688	2391768800	97.76%	93.44%	38.04%	HBV_B2	7.91	19	19	0	0	46.19%	36.52%	15.40%	0.00%	0.00%	0.00%	7	UC	2	N	N	N	N
T4	54	150;150	28937000	2893700000	97.65%	93.19%	38.63%	HBV_B2	19.41	32	32	0	0	66.44%	48.31%	39.23%	16.42%	6.10%	1.12%	9	1	12	N	N	N	N
T4	56	150;150	22488190	2248819000	97.79%	93.51%	37.14%	HBV_B2	1287.29	61	61	0	0	79.38%	79.38%	79.35%	79.31%	79.31%	74.00%	101	43	136	Y	Y	N	N
T4	57	150;150	17176960	1717696000	97.70%	93.31%	38.34%	HBV_B2	7.69	26	26	0	0	50.48%	35.58%	11.11%	0.53%	0.00%	0.00%	7	UC	5	N	N	N	N
T4	58	150;150	16457136	1645713600	97.55%	92.92%	40.05%	HBV_B2	4.62	20	20	0	0	57.51%	28.68%	5.66%	0.00%	0.00%	0.00%	-	-	-	-	-	-	-
T4	60	150;150	18282580	1828258000	97.58%	92.99%	40.13%	HBV_B2	24.89	40	40	0	0	77.51%	59.60%	51.75%	19.50%	4.42%	3.89%	26	UC	16	N	N	N	N
T4	64	150;150	17499890	1749989000	97.60%	93.05%	39.32%	HBV_B2	4.19	13	13	0	0	42.89%	25.44%	2.14%	0.00%	0.00%	0.00%	-	-	-	-	-	-	-
T4	65	150;150	18662814	1866281400	97.76%	93.45%	38.13%	HBV_B2	6.5	16	16	0	0	43.92%	30.01%	8.86%	0.00%	0.00%	0.00%	5	UC	6	N	N	N	N
T4	66	150;150	19682036	1968203600	97.68%	93.25%	38.40%	HBV_B2	6.26	17	17	0	0	51.35%	33.66%	7.81%	0.00%	0.00%	0.00%	5	UC	1	N	N	N	N
T4	71	150;150	23130434	2313043400	97.68%	93.22%	37.34%	HBV_B2	8.57	14	14	0	0	43.33%	34.93%	10.92%	0.00%	0.00%	0.00%	11	UC	6	N	N	N	N
T4	72	150;150	20478872	2047887200	97.70%	93.35%	37.64%	HBV_B2	6.48	22	22	0	0	62.33%	31.79%	15.08%	0.00%	0.00%	0.00%	7	UC	3	N	N	N	N
HVTT	1	150;150	13748416	1374841600	94.15%	85.30%	50.41%	***HBV_B1***	4.09	10	10	0	0	46.67%	26.01%	2.99%	0.00%	0.00%	0.00%	-	-	-	-	-	-	-
HVTT	2	150;150	15196008	1519600800	95.99%	89.28%	42.74%	HBV_B2	22.09	57	57	0	0	79.32%	79.32%	61.09%	16.61%	3.85%	2.20%	22	5	16	N	N	N	N
HVTT	4	150;150	18125218	1812521800	95.64%	88.49%	43.17%	HBV_B2	4.73	21	21	0	0	60.44%	35.92%	2.55%	0.00%	0.00%	0.00%	-	-	-	-	-	-	-
HVTT	5	150;150	15609848	1560984800	95.82%	89.01%	42.57%	HBV_B2	3.70	10	10	0	0	57.88%	27.90%	0.34%	0.00%	0.00%	0.00%	-	-	-	-	-	-	-
HVTT	6	150;150	23713362	2371336200	95.30%	87.89%	45.66%	HBV_B2	7.41	29	29	0	0	72.26%	51.60%	21.09%	0.00%	0.00%	0.00%	13	UC	5	N	N	N	N
HVTT	7	150;150	17071984	1707198400	95.88%	89.06%	43.20%	HBV_B2	3.41	7	7	0	0	60.25%	23.27%	0.00%	0.00%	0.00%	0.00%	-	-	-	-	-	-	-
HVTT	8	150;150	15108784	1510878400	95.54%	88.29%	44.49%	HBV_B2	1592.26	33	33	0	0	67.25%	42.24%	41.80%	41.68%	41.59%	41.55%	101	UC	129	N	Y	N	N
HVTT	9	150;150	11817672	1181767200	95.70%	88.69%	44.13%	HBV_B2	18.78	58	58	0	0	79.32%	78.78%	64.07%	11.38%	0.03%	0.00%	10	2	8	N	N	N	N
HVTT	10	150;150	12805732	1280573200	95.82%	88.91%	43.83%	HBV_B2	3.12	9	9	0	0	57.33%	18.94%	0.25%	0.00%	0.00%	0.00%	-	-	-	-	-	-	-
HVTT	11	150;150	14670404	1467040400	95.48%	88.18%	44.67%	HBV_B2	26.07	61	61	0	0	79.32%	79.32%	79.32%	17.98%	1.62%	1.25%	18	13	6	N	N	N	N
HVTT	12	150;150	14836288	1483628800	96.02%	89.34%	42.71%	HBV_B2	34.28	61	61	0	0	79.32%	79.32%	79.32%	46.91%	4.05%	1.05%	22	11	9	N	N	N	N
HVTT	16	150;150	15199592	1519959200	95.49%	88.23%	44.74%	HBV_B2	3.44	6	6	0	0	51.32%	21.00%	2.80%	0.00%	0.00%	0.00%	-	-	-	-	-	-	-
HVTT	17	150;150	16992516	1699251600	95.20%	87.58%	45.94%	HBV_B2	2.62	3	3	0	0	57.57%	16.79%	0.00%	0.00%	0.00%	0.00%	-	-	-	-	-	-	-
HVTT	19	150;150	12592288	1259228800	95.73%	88.81%	42.63%	HBV_B2	3.49	6	6	0	0	55.61%	31.73%	0.00%	0.00%	0.00%	0.00%	-	-	-	-	-	-	-
HVTT	22	150;150	16577758	1657775800	96.16%	89.58%	41.90%	HBV_B2	4.2	12	12	0	0	69.92%	37.51%	3.98%	0.00%	0.00%	0.00%	-	-	-	-	-	-	-
HVTT	23	150;150	12205552	1220555200	96.01%	89.33%	42.10%	HBV_B2	3.09	5	5	0	0	57.60%	23.33%	0.44%	0.00%	0.00%	0.00%	-	-	-	-	-	-	-
HVTT	25	150;150	14769226	1476922600	96.02%	89.34%	41.79%	HBV_B2	4.77	11	11	0	0	58.23%	34.28%	3.39%	0.00%	0.00%	0.00%	-	-	-	-	-	-	-
HVTT	28	150;150	15308246	1530824600	96.26%	89.84%	40.97%	HBV_B2	3.36	4	4	0	0	61.56%	22.02%	1.09%	0.00%	0.00%	0.00%	-	-	-	-	-	-	-
HVTT	30	150;150	12297668	1229766800	96.25%	89.81%	40.31%	HBV_B2	3.94	7	7	0	0	55.80%	27.03%	3.76%	0.00%	0.00%	0.00%	-	-	-	-	-	-	-
HVTT	33	150;150	14029344	1402934400	96.02%	89.37%	41.53%	HBV_B2	4.17	15	15	0	0	71.79%	37.85%	2.27%	0.00%	0.00%	0.00%	-	-	-	-	-	-	-
HVTT	35	150;150	17015520	1701552000	96.25%	89.85%	40.64%	HBV_B2	6.49	24	24	0	0	77.26%	48.09%	18.01%	0.00%	0.00%	0.00%	14	1	2	N	N	N	N
HVTT	36	150;150	16360162	1636016200	96.25%	89.82%	41.10%	HBV_B2	4.95	24	24	0	0	70.45%	40.75%	7.15%	0.00%	0.00%	0.00%	-	-	-	-	-	-	-
HVTT	37	150;150	17892340	1789234000	97.09%	91.28%	43.32%	***HBV_B9***	1.94	0	0	0	0	55.89%	4.70%	0.00%	0.00%	0.00%	0.00%	-	-	-	-	-	-	-
HVTT	38	150;150	26211660	2621166000	97.11%	91.34%	42.77%	HBV_B2	1.94	0	0	0	0	70.14%	3.79%	0.00%	0.00%	0.00%	0.00%	-	-	-	-	-	-	-
HVTT	39	150;150	23447512	2344751200	97.22%	91.61%	41.62%	HBV_B2	2.86	6	6	0	0	62.52%	18.47%	0.00%	0.00%	0.00%	0.00%	-	-	-	-	-	-	-
HVTT	40	150;150	30903812	3090381200	97.26%	91.66%	42.19%	HBV_B2	744.15	61	61	0	0	79.38%	79.35%	79.31%	79.31%	79.31%	79.31%	96	115	107	N	N	N	N
HVTT	41	150;150	23710680	2371068000	97.08%	91.27%	43.24%	HBV_B2	2.16	3	3	0	0	66.66%	9.33%	0.00%	0.00%	0.00%	0.00%	-	-	-	-	-	-	-
HVTT	43	150;150	25140542	2514054200	97.31%	91.85%	41.19%	HBV_B2	3.29	0	0	0	0	72.38%	25.29%	2.08%	0.00%	0.00%	0.00%	-	-	-	-	-	-	-
HVTT	50	150;150	2403848	240384800	96.99%	91.09%	42.89%	-	-	-	-	-	-	-	-	-	-	-	-	-	-	-	-	-	-	-
HVTT	51	150;150	23519928	2351992800	97.05%	91.19%	43.13%	HBV_B2	2.36	1	1	0	0	54.74%	12.35%	0.00%	0.00%	0.00%	0.00%	-	-	-	-	-	-	-
HVTT	52	150;150	27172124	2717212400	97.13%	91.34%	42.56%	HBV_B2	2.31	1	1	0	0	62.18%	10.89%	0.00%	0.00%	0.00%	0.00%	-	-	-	-	-	-	-
HVTT	53	150;150	17098498	1709849800	96.59%	90.05%	45.89%	***HBV_B4***	1.58	0	0	0	0	33.00%	1.12%	0.00%	0.00%	0.00%	0.00%	-	-	-	-	-	-	-
HVTT	57	150;150	22179466	2217946600	96.85%	90.65%	45.17%	HBV_B2	2.15	1	1	0	0	53.31%	9.70%	0.00%	0.00%	0.00%	0.00%	-	-	-	-	-	-	-
HVTT	62	150;150	21287952	2128795200	96.72%	90.35%	44.51%	HBV_B2	2.75	1	1	0	0	65.35%	19.94%	0.00%	0.00%	0.00%	0.00%	-	-	-	-	-	-	-
HVTT	64	150;150	21905018	2190501800	97.30%	91.82%	41.70%	HBV_B2	1.79	0	0	0	0	52.88%	4.20%	0.00%	0.00%	0.00%	0.00%	-	-	-	-	-	-	-
HVTT	68	150;150	22481340	2248134000	97.20%	91.65%	41.76%	HBV_B2	281.11	61	61	0	0	79.35%	79.35%	79.32%	79.32%	79.32%	79.19%	86	74	84	N	N	N	N
HVTT	69	150;150	22100992	2210099200	97.01%	91.14%	42.91%	HBV_B2	2.21	0	0	0	0	42.58%	5.35%	0.00%	0.00%	0.00%	0.00%	-	-	-	-	-	-	-
HVTT	70	150;150	20428086	2042808600	96.17%	88.71%	49.04%	HBV_B2	1.58	1	1	0	0	38.01%	3.11%	0.00%	0.00%	0.00%	0.00%	-	-	-	-	-	-	-
HVTT	71	150;150	22847662	2284766200	95.88%	87.94%	51.15%	HBV_B2	1.62	0	0	0	0	54.37%	0.78%	0.00%	0.00%	0.00%	0.00%	-	-	-	-	-	-	-
HVTT	72	150;150	26751500	2675150000	96.97%	90.94%	44.65%	***HBV_B1***	2.14	0	0	0	0	39.23%	8.37%	0.00%	0.00%	0.00%	0.00%	-	-	-	-	-	-	-
PVTT	2	150;150	14129204	1412920400	97.71%	92.98%	44.00%	HBV_B2	4.67	12	12	0	0	77.95%	48.27%	3.98%	0.00%	0.00%	0.00%	-	-	-	-	-	-	-

PVTT	3	150;150	15489812	1548981200	97.61%	92.76%	44.88%	***HBV_B4***	3.23	6	6	0	0	78.41%	28.12%	0.90%	0.00%	0.00%	0.00%	-	-	-	-	-	-	-
PVTT	4	150;150	14177732	1417773200	97.77%	93.15%	42.62%	HBV_B2	126.71	61	61	0	0	79.38%	79.31%	79.31%	79.31%	79.31%	63.76%	61	47	59	N	N	N	N
PVTT	5	150;150	13883896	1388389600	97.78%	93.16%	43.77%	HBV_B2	19.74	60	60	0	0	79.32%	79.32%	77.05%	5.07%	0.47%	0.00%	14	4	9	N	N	N	N
PVTT	7	150;150	10972672	1097267200	97.05%	91.26%	47.06%	HBV_B2	16.63	52	52	0	0	79.32%	77.76%	70.88%	4.82%	0.00%	0.00%	11	8	10	N	N	N	N
PVTT	8	150;150	11581378	1158137800	97.20%	91.52%	46.15%	HBV_B2	2.24	3	3	0	0	61.15%	11.36%	0.00%	0.00%	0.00%	0.00%	-	-	-	-	-	-	-
PVTT	10	150;150	12568740	1256874000	97.22%	91.63%	47.76%	HBV_B2	332.27	61	61	0	0	79.38%	79.35%	79.31%	79.31%	79.31%	79.25%	85	66	80	N	N	N	N
PVTT	11	150;150	10983660	1098366000	97.07%	91.07%	48.82%	HBV_B2	1.36	0	0	0	0	28.74%	0.22%	0.00%	0.00%	0.00%	0.00%	-	-	-	-	-	-	-
PVTT	12	150;150	8970880	897088000	97.17%	91.44%	47.97%	HBV_B2	30.54	61	61	0	0	79.32%	79.32%	78.41%	39.38%	9.08%	0.00%	13	8	6	N	N	N	N
PVTT	13	150;150	13251336	1325133600	96.92%	90.98%	48.98%	-	-	-	-	-	-	-	-	-	-	-	-	-	-	-	-	-	-	-
PVTT	19	150;150	13812898	1381289800	97.31%	91.92%	46.48%	HBV_B2	3.26	8	8	0	0	71.17%	29.18%	1.27%	0.00%	0.00%	0.00%	-	-	-	-	-	-	-
PVTT	20	150;150	12155370	1215537000	96.18%	88.89%	54.99%	-	-	-	-	-	-	-	-	-	-	-	-	-	-	-	-	-	-	-
PVTT	24	150;150	11636414	1163641400	97.31%	92.01%	47.25%	HBV_B2	1.57	0	0	0	0	49.98%	1.99%	0.00%	0.00%	0.00%	0.00%	-	-	-	-	-	-	-
PVTT	26	150;150	11934052	1193405200	97.39%	92.21%	45.43%	-	-	-	-	-	-	-	-	-	-	-	-	-	-	-	-	-	-	-
PVTT	29	150;150	15647430	1564743000	97.47%	92.41%	45.95%	HBV_B2	226.1	61	61	0	0	79.35%	79.32%	79.32%	79.32%	79.32%	79.07%	76	71	65	N	N	N	N
PVTT	30	150;150	13286660	1328666000	97.16%	91.47%	49.53%	-	-	-	-	-	-	-	-	-	-	-	-	-	-	-	-	-	-	-
PVTT	32	150;150	10608880	1060888000	97.37%	92.17%	46.74%	HBV_B2	1.84	0	0	0	0	37.23%	1.12%	1.06%	0.00%	0.00%	0.00%	-	-	-	-	-	-	-
PVTT	33	150;150	10057370	1005737000	97.55%	92.55%	45.52%	HBV_B2	124.27	61	61	0	0	79.35%	79.32%	79.32%	79.32%	78.76%	57.98%	56	27	35	N	N	N	N
PVTT	35	150;150	16744094	1674409400	97.52%	92.46%	45.23%	HBV_B2	1.59	0	0	0	0	30.36%	0.22%	0.00%	0.00%	0.00%	0.00%	-	-	-	-	-	-	-
PVTT	36	150;150	13006422	1300642200	97.44%	92.30%	45.05%	HBV_B2	3.11	4	4	0	0	57.98%	20.06%	0.00%	0.00%	0.00%	0.00%	-	-	-	-	-	-	-
PVTT	37	150;150	15046710	1504671000	97.28%	91.83%	47.27%	HBV_B2	1.65	0	0	0	0	31.88%	0.06%	0.00%	0.00%	0.00%	0.00%	-	-	-	-	-	-	-
PVTT	38	150;150	14513544	1451354400	96.40%	89.39%	53.85%	-	-	-	-	-	-	-	-	-	-	-	-	-	-	-	-	-	-	-
PVTT	39	150;150	19290618	1929061800	97.54%	92.56%	45.19%	HBV_B2	8.85	39	39	0	0	75.74%	66.91%	31.05%	0.00%	0.00%	0.00%	5	UC	6	N	N	N	N
PVTT	41	150;150	17169072	1716907200	97.36%	92.11%	45.44%	HBV_B2	334.14	61	61	0	0	79.35%	79.32%	79.32%	79.32%	79.32%	79.32%	86	80	81	N	N	N	N
PVTT	43	150;150	15102886	1510288600	97.34%	92.04%	47.21%	HBV_B2	95.58	60	60	0	0	79.35%	79.32%	79.32%	77.79%	66.60%	36.52%	49	7	45	N	N	N	N
PVTT	44	150;150	17420046	1742004600	97.58%	92.59%	45.61%	HBV_B2	1.53	0	0	0	0	36.83%	0.50%	0.00%	0.00%	0.00%	0.00%	-	-	-	-	-	-	-
PVTT	45	150;150	16179076	1617907600	97.57%	92.59%	45.08%	HBV_B2	25.68	61	61	0	0	79.32%	79.32%	79.32%	18.82%	3.48%	0.00%	22	13	9	N	N	N	N
PVTT	47	150;150	18218828	1821882800	97.47%	92.29%	43.98%	-	-	-	-	-	-	-	-	-	-	-	-	-	-	-	-	-	-	-
PVTT	48	150;150	18535972	1853597200	97.33%	91.95%	44.42%	HBV_B2	5.7	22	22	0	0	78.48%	56.95%	7.27%	0.00%	0.00%	0.00%	4	5	3	N	N	N	N
PVTT	49	150;150	19255210	1925521000	97.46%	92.35%	44.36%	HBV_B2	1.33	0	0	0	0	48.12%	0.13%	0.00%	0.00%	0.00%	0.00%	-	-	-	-	-	-	-
PVTT	50	150;150	16264936	1626493600	97.50%	92.37%	44.81%	-	-	-	-	-	-	-	-	-	-	-	-	-	-	-	-	-	-	-
PVTT	51	150;150	15688752	1568875200	97.30%	91.83%	44.86%	HBV_B2	1.17	0	0	0	0	27.12%	0.00%	0.00%	0.00%	0.00%	0.00%	-	-	-	-	-	-	-
PVTT	52	150;150	19086990	1908699000	97.63%	92.74%	43.28%	HBV_B2	1.74	1	1	0	0	38.94%	3.92%	0.00%	0.00%	0.00%	0.00%	-	-	-	-	-	-	-
PVTT	55	150;150	20067218	2006721800	97.24%	91.66%	46.69%	HBV_B2	1.58	0	0	0	0	36.14%	1.93%	0.00%	0.00%	0.00%	0.00%	-	-	-	-	-	-	-
PVTT	56	150;150	17863476	1786347600	97.44%	92.26%	44.71%	HBV_B2	1.48	0	0	0	0	47.15%	1.90%	0.00%	0.00%	0.00%	0.00%	-	-	-	-	-	-	-
PVTT	57	150;150	21365008	2136500800	97.20%	91.59%	46.85%	HBV_B2	1.64	0	0	0	0	53.09%	1.31%	0.00%	0.00%	0.00%	0.00%	-	-	-	-	-	-	-
PVTT	60	150;150	18364200	1836420000	97.47%	92.38%	44.31%	HBV_B2	1.95	0	0	0	0	50.64%	2.92%	0.00%	0.00%	0.00%	0.00%	-	-	-	-	-	-	-
PVTT	62	150;150	20921120	2092112000	96.77%	90.44%	49.96%	***HBV_B9***	1.75	0	0	0	0	52.10%	4.17%	0.00%	0.00%	0.00%	0.00%	-	-	-	-	-	-	-
PVTT	63	150;150	19104408	1910440800	97.17%	91.47%	47.63%	HBV_B2	1.75	1	1	0	0	44.82%	3.86%	0.00%	0.00%	0.00%	0.00%	-	-	-	-	-	-	-
PVTT	64	150;150	19430640	1943064000	96.60%	90.17%	51.58%	HBV_B2	320.9	61	61	0	0	79.38%	79.31%	79.31%	79.31%	79.31%	74.37%	83	56	67	N	N	N	N
PVTT	66	150;150	19388824	1938882400	97.13%	91.42%	47.27%	HBV_B2	2.4	1	1	0	0	38.57%	4.79%	0.00%	0.00%	0.00%	0.00%	-	-	-	-	-	-	-
PVTT	69	150;150	21676184	2167618400	96.89%	90.76%	49.54%	HBV_B2	1.61	1	1	0	0	36.05%	2.89%	0.00%	0.00%	0.00%	0.00%	-	-	-	-	-	-	-
PVTT	70	150;150	23988962	2398896200	97.30%	91.90%	46.28%	HBV_B2	256.53	61	61	0	0	79.38%	79.35%	79.31%	79.31%	79.31%	73.87%	78	84	49	N	N	N	N
PVTT	71	150;150	26934638	2693463800	96.63%	90.02%	51.45%	HBV_B2	2.08	0	0	0	0	50.73%	6.35%	0.00%	0.00%	0.00%	0.00%	-	-	-	-	-	-	-
PVTT	72	150;150	31297816	3129781600	97.14%	91.48%	47.57%	HBV_B2	2.48	1	1	0	0	60.90%	12.63%	0.00%	0.00%	0.00%	0.00%	-	-	-	-	-	-	-

Table S5. Homologous sequences at the junction sites of minor viral integration and HBV rearrangement cases.

1. All HBV sequences are from the constructed HBV genome.

2. Upstream partner is in blue, and downstream partner is in yellow.

3. Mutated position on HBV genome is marked by asterisk and frame.

4. Red cells harbouring nucleotides that align to both reference sequences are micro-homologies.

Human_breakpoint	chr1:34397255:+; as downsteam	
HBV_breakpoint	HBV_B2:3026:+; as upstream	
shared_Cells	T1:NO.29; T1:NO.30; T2:NO.98; T3:NO.51; T3:NO.116	
**NO.1**	*upstream (5')*	*downstream (3')*
HBV_B2	GACCTCAACCCGCACAAGGACAACTGGCCGGACGCCAACAAGGTGGGAGT	GGGAGCATTCGGACCAGGGTTCACCCCTCCCCATGGGGACTGTTGGGGT
Integration	GACCTCAACCCGCACAAGGACAACTGGCCGGACGCCAACAAGGTGGGAGT	GGGAGCATTCTCTGGTGTGGCTAAAGTGGAAGCTACTTAAAATAAGTCTA
Chr1	CTAGAAGCAGGATTTGACATCCTCAGCTGGCTTCCCTAGGGCGTTTGGAA	GGGAGCATTCTCTGGTGTGGCTAAAGTGGAAGCTACTTAAAATAAGTCTA
Human_breakpoint	chr1:34397645:+; as downstream	
HBV_breakpoint	HBV_B2:3112:+; as upstream	
shared_Cells	T1:NO.29; T1:NO.58	
**NO.2**	*upstream (5')*	*downstream (3')*
HBV_B2	GGGACTGTTGGGGTGGAGCCCTCAGGCTCAGGGCATACTCACAACTGTGC	CAGCAACTCCTCCTCCTGCCTCCACCAATCGGCAGTCAGGAAGGCAGCCT
Integration	GGGACTGTTGGGGTGGAGCCCTCAGGCTCAGGGCATACTCACAACTGTGC	CAGCAAGAAAAGCAAGAATGTAAGATGCCCTGAGGGGTTCAGCTTGGCCA
Chr1	AGGGGGTAGGCTGGTGCAGAGGGACATGGGGGCCGATGATGCGATGTGAA	CAGCAAGAAAAGCAAGAATGTAAGATGCCCTGAGGGGTTCAGCTTGGCCA
Human_breakpoint	chr1:34397754:+; as downstream	
HBV_breakpoint	HBV_B2:2885:+; as upstream	
shared_Cells	T2:NO.97; T3:NO.116	
**NO.3**	*upstream (5')*	*downstream (3')*
HBV_B2	AAGATCTACAGCATGGGAGGTTGGTCTTCCAAACCTCGAAAAGGCATGGG	GACAAATCTTTCTGTCCCCAATCCCCTGGGATTCTTCCCCGATCATCAGT
Integration	AAGATCTACAGCATGGGAGGTTGGTCTTCCAAACCTCGAAAAGGCATGGG	GAAAGCAGGAAGAGTGAGGTTTTCATGGGCCAAGTTTGAAACAAGCTGAG
Chr1	GGGACACTTTTCACTGAGCTGGGGAAGGCAACAAAAAGAGCAGGTTTGGG	GAAAGCAGGAAGAGTGAGGTTTTCATGGGCCAAGTTTGAAACAAGCTGAG
Human_breakpoint	chr1:34397852:+; as downstream	
HBV_breakpoint	HBV_B2:3096:+; as upstream	
shared_Cells	T3:NO.47; T3:NO.116	
**NO.4**	*upstream (5')*	*downstream (3')*
HBV_B2	TCACCCCTCCCCATGGGGACTGTTGGGGTGGAGCCCTCAGGCTCAGGGC	ATACTCACAACTGTGCCAGCAACTCCTCCTCCTGCCTCCACCAATCGGCA
Integration	TCACCCCTCCCCATGGGGACTGTTGGGGTGGAGCCCTCAGGCTCAGGGC	AGAGAATGGGGTTGGAGGTGGGGATTGGAGAGTTGCCCCCTAGTAACAAT
Chr1	AGGAGAACTTTAGCATAGTTCACTCTCCACAGGGGCCTGAAGCTCAGGAC	AGAGAATGGGGTTGGAGGTGGGGATTGGAGAGTTGCCCCCTAGTAACAAT
Human_breakpoint	chr1:34399597:+; as downstream	
HBV_breakpoint	HBV_B2:2629:+; as upstream	
shared_Cells	T2:NO.86; T2:NO.97; T3:NO.51; T3: NO.116; T4:NO.56	
**NO.5**	*upstream (5')*	*downstream (3')*
HBV_B2	TGTAAGCAATTTGTGGGGCCCCTTACAGTAAATGAAAACAGGAGACTAAA	ATTAATTATGCCTGCTAGGTTTTATCCCAATGTTACTAAATATTTGCCCT
Integration	TGTAAGCAATTTGTGGGGCCCCTTACAGTAAATGAAAACAGGAGACTAAA	ATTAATTATGTCTGCCATGGGATTGGAAGAAGGGGAATAGGTATACATG
Chr1	AGGGAATTCACAGCTGTGTCTGGACCCAAGCAGAAAGGGTGCAATGAACA	ATTAATTATGTCTGCCATGGGATTGGAAGAAGGGGAATAGGTATACATG
Human_breakpoint	chr1:34400010:+; as downstream	
HBV_breakpoint	HBV_B2:2417:+; as upstream	
shared_Cells	T1:NO.58; T3: NO.116	
**NO.6**	*upstream (5')*	*downstream (3')*
HBV_B2	TAGAAGAAGAACTCCCTCGCCTCGCAGACGAAGGTCTCAATCGCCGCGTC	GCAGAAGATCTCAATCTCGGGAATCTCAATGTTAGTATTCCTTGGACACA
Integration	TAGAAGAAGAACTCCCTCGCCTCGCAGACGAAGGTCTCAATCGCCGCGTC	GCAGAGGAGTAAATGAGAAGGGAAAAGGAGGGACAAGGCTGGATGGAGCT
Chr1	CCCATCTGATTTGGCAAAATGATGCAAATATTGAGGGCAAAGGCCAGTCT	GCAGAGGAGTAAATGAGAAGGGAAAAGGAGGGACAAGGCTGGATGGAGCT
Human_breakpoint	chr1:34400086:+; as downstream	
HBV_breakpoint	HBV_B2:2879:+; as upstream	
shared_Cells	T3: NO.116; T4:NO.56	
**NO.7**	*upstream (5')*	*downstream (3')*
HBV_B2	GGGAACAAGATCTACAGCATGGGAGGTTGGTCTTCCAAACCTCGAAAAGG	CATGGGGACAAATCTTTCTGTCCCCAATCCCCTGGGATTCTTCCCCGATC
Integration	GGGAACAAGATCTACAGCATGGGAGGTTGGTCTTCCAAACCTCGAAAAGG	CATGGGAAATCTTACAGTGTGATGTGATTGATTGTTTTTCAAAGGTGGTT
Chr1	GGAGGGACAAGGCTGGATGGAGCTTTCAAGATGCTGGGCTATGTTGATGG	CATGGGAAATCTTACAGTGTGATGTGATTGATTGTTTTTCAAAGGTGGTT
Human_breakpoint	chr1:34397304:+; as downstream	
HBV_breakpoint	HBV_B2:2352:+; as upstream	
shared_Cells	T3: NO.116; T4:NO.56	
**NO.8**	*upstream (5')*	*downstream (3')*
HBV_B2	CCAAATGCCCCTATCTTATCAACACTTCCGGAAACTACTGTTGTTAGACG	AAGAGGCAGGTCCCCTAGAAGAAGAACTCCCTCGCCTCGCAGACGAAGGT
Integration	CCAAATGCCCCTATCTTATCAACACTTCCGGAAACTACTGTTGTTAGACG	AAGAGACAGACTGGAAAGATTGGGGTGGGGCTCTTTCTGGAAGAGCCTTC

[illegible]	Chr1	AGGGAGCATTCTCTGGTGTGGCTAAAGTGGAAGCTACTTAAAATAAGTCTAAGAGACAGACTGGAAAGATTGGGGTGGGGCTCTTTCTGGAAGAGCCTTC
Human_breakpoint	chr8:118557874:-; as upstream	
HBV_breakpoint	HBV_B2:480:+; as downstream	
shared_Cells	T2: NO.102; T2:NO.110	
NO.9		upstream (5') … downstream (3')
	Chr8	ATTGCCCTCCACCATCTGAAAGAAAAAGACCCTTCTCCCACCGACTCCTCTCCCTCTCCCCCACAAATACAGAAACAGTTGTAAACAAGCAAGAAAGGAC
	Integration	ATTGCCCTCCACCATCTGAAAGAAAAAGACCCTTCTCCCACCGACTCCTCTCATTCCAGGATCATCAACAACCAGCACCGGACCATGCAAAACCTGCACA
	HBV_B2	CTTCTTGTTGGTTCTTCTGGACTATCAAGGTATGTTGCCCGTTTGTCCTCTCATTCCAGGATCATCAACAACCAGCACCGGACCATGCAAAACCTGCACA
Human_breakpoint	chr8:118557559:-; as upstream	
HBV_breakpoint	HBV_B2:969:+; as downstream	
shared_Cells	T2: NO.105; T4:NO.56; HVTT:NO.8	
NO.10		upstream (5') … downstream (3')
	Chr8	TTCAAATTCTGCCTCTGAGACTGTTTGATGCTTTAGTTATTTCTCACCTGGACTATTGTTGGTACCTTGGCAAGCCCTTTCTATTCATCTGTCATGCTTT
	Integration	TTCAAATTCTGCCTCTGAGACTGTTTGATGCTTTAGTTATTTCTCACCTGGCCTATTGATTGGAAAGTATGTCAACGCATTGTGGGTCTTTTGGGGTTTG
	HBV_B2	CATATTGTACAAAAAAATCAAAATGTGTTTTCGGAAACTTCCTGTAAACCGGCCTATTGATTGGAAAGTATGTCAACGCATTGTGGGTCTTTTGGGGTTTG
HBV_breakpoint	HBV_B2:1982:+; as upstream	
HBV_breakpoint	HBV_B2:2429:-; as downstream	
shared_Cells	T3: NO.51; T3:NO.116	
NO.11		upstream (5') … downstream (3')
	HBV_B2	TTCTGTGGAGTTACTCTCTTTTTTGCCTTCTGACTTCTTTCCTTCTATTCGAGATCTCCTCGACACCGCCTCTGCTCTGCATCGGGAGGCCTTAGAGTCT
	Integration	TTCTGTGGAGTTACTCTCTTTTTTGCCTTCTGACTTCTTTCCTTCTATTCGAGATCTTCTGCGACGCGGCGATTGAGACCTTCGTCTGCGAGGCGAGGGA
	HBV_B2	TTTCCCACCTTATGTGTCCAAGGAATACTAACATTGAGATTCCCGAGATTGAGATCTTCTGCGACGCGGCGATTGAGACCTTCGTCTGCGAGGCGAGGGA

Table S6. Copy number alterations of six tumor specimens.
1. The result is from PatchWork.
2. "CN" means the full copy number, "mCN" means the minor copy number.
3. Cells with "-" value mean relevant genomic region lacks CNA information in relevant sample.

	Start	End	CopyNumber (CN)						minor CN (mCN)						CN	CN	Z-Score (CN)					
Chr	Position	Position	T1	T2	T3	T4	HVTT	PVTT	T1	T2	T3	T4	HVTT	PVTT	Mean	SD	T1	T2	T3	T4	HV	PV
chr1	19801	159800	3	4	4	5	3	4	1	2	0	2	1	1	3.83	0.75	-1.11	0.22	0.22	1.55	-1.11	0.22
chr1	159801	169800	3	4	4	5	3	4	1	2	0	2	1	1	3.83	0.75	-1.11	0.22	0.22	1.55	-1.11	0.22
chr1	169801	230195	3	4	4	5	4	4	1	2	0	2	2	1	4	0.63	-1.58	0	0	1.58	0	0
chr1	230196	320590	3	4	5	5	4	4	1	2	1	2	2	1	4.17	0.75	-1.55	-0.22	1.11	1.11	-0.22	-0.22
chr1	320591	340390	3	4	5	5	4	5	1	2	1	2	2	2	4.33	0.82	-1.63	-0.41	0.82	0.82	-0.41	0.82
chr1	340391	630785	4	4	5	5	4	5	2	2	1	2	2	2	4.5	0.55	-0.91	-0.91	0.91	0.91	-0.91	0.91
chr1	630786	640785	4	4	5	5	4	5	2	2	1	2	2	2	4.5	0.55	-0.91	-0.91	0.91	0.91	-0.91	0.91
chr1	640786	660585	4	4	3	5	3	5	2	2	0	2	1	2	4	0.89	0	0	-1.12	1.12	-1.12	1.12
chr1	660586	670785	2	2	3	5	3	5	0	0	0	2	1	2	3.33	1.37	-0.98	-0.98	-0.24	1.22	-0.24	1.22
chr1	670786	730785	2	2	2	2	2	2	0	0	0	0	0	1	2	0	0	0	0	0	0	0
chr1	730786	840385	2	2	2	2	2	1	0	0	0	0	0	0	1.83	0.41	0.41	0.41	0.41	0.41	0.41	-2.04
chr1	840386	850385	2	2	2	2	2	1	0	0	0	0	0	0	1.83	0.41	0.41	0.41	0.41	0.41	0.41	-2.04
chr1	850386	2711185	2	2	2	2	2	2	0	0	0	0	0	0	2	0	0	0	0	0	0	0
chr1	2711186	2721185	2	2	2	2	2	2	0	0	0	0	0	0	2	0	0	0	0	0	0	0
chr1	2721186	5961380	2	2	2	2	2	2	0	0	0	0	0	0	2	0	0	0	0	0	0	0
chr1	5961381	5971380	2	2	2	2	2	2	0	0	0	0	0	0	2	0	0	0	0	0	0	0
chr1	5971381	13192975	2	2	2	2	2	2	0	0	0	0	0	0	2	0	0	0	0	0	0	0
chr1	13192976	13202975	2	2	2	2	2	2	0	0	0	0	0	0	2	0	0	0	0	0	0	0
chr1	13202976	13353170	2	1	2	2	1	1	0	0	0	0	0	0	1.5	0.55	0.91	-0.91	0.91	0.91	-0.91	-0.91
chr1	13353171	13363170	2	1	2	2	1	1	0	0	0	0	0	0	1.5	0.55	0.91	-0.91	0.91	0.91	-0.91	-0.91
chr1	13363171	13373970	2	2	2	2	2	1	0	0	0	0	0	0	1.83	0.41	0.41	0.41	0.41	0.41	0.41	-2.04
chr1	13373971	15214165	2	2	2	2	2	2	0	0	0	0	0	0	2	0	0	0	0	0	0	0
chr1	15214166	15224165	2	2	2	2	2	2	0	0	0	0	0	0	2	0	0	0	0	0	0	0
chr1	15224166	16914565	2	2	2	2	2	2	0	0	0	0	0	0	2	0	0	0	0	0	0	0
chr1	16914566	16924565	2	2	2	2	2	2	0	0	0	0	0	0	2	0	0	0	0	0	0	0
chr1	16924566	16944565	2	2	2	2	2	2	1	0	0	0	0	1	2	0	0	0	0	0	0	0
chr1	16944566	16954565	2	2	2	2	2	2	1	0	0	0	0	1	2	0	0	0	0	0	0	0
chr1	16954566	17125365	2	2	2	2	2	2	1	1	0	0	1	1	2	0	0	0	0	0	0	0
chr1	17125366	17184760	2	2	2	2	2	2	1	1	0	0	1	1	2	0	0	0	0	0	0	0
chr1	17184761	17194760	2	2	2	2	2	3	1	1	0	0	1	1	2.17	0.41	-0.41	-0.41	-0.41	-0.41	-0.41	2.04
chr1	17194761	17274760	3	3	2	2	3	3	1	1	0	0	1	1	2.67	0.52	0.65	0.65	-1.29	-1.29	0.65	0.65
chr1	17274761	17284760	3	3	2	2	3	3	1	1	0	0	1	1	2.67	0.52	0.65	0.65	-1.29	-1.29	0.65	0.65
chr1	17284761	24255760	2	2	2	2	2	2	0	0	0	0	0	0	2	0	0	0	0	0	0	0
chr1	24255761	24266160	2	2	2	2	2	2	1	0	0	0	0	0	2	0	0	0	0	0	0	0
chr1	24266161	24284960	2	2	2	2	2	2	1	0	0	0	0	0	2	0	0	0	0	0	0	0
chr1	24284961	24305360	2	2	2	2	2	2	1	0	0	0	1	0	2	0	0	0	0	0	0	0
chr1	24305361	24315360	2	2	2	2	2	2	1	0	0	0	1	0	2	0	0	0	0	0	0	0
chr1	24315361	24356160	2	2	2	2	2	2	0	0	0	0	1	0	2	0	0	0	0	0	0	0
chr1	24356161	24366160	2	2	2	2	2	2	0	0	0	0	1	0	2	0	0	0	0	0	0	0
chr1	24366161	34386755	2	2	2	2	2	2	0	0	0	0	0	0	2	0	0	0	0	0	0	0
chr1	34386756	34396955	2	2	4	2	2	2	0	0	2	0	0	0	2.33	0.82	-0.41	-0.41	2.04	-0.41	-0.41	-0.41
chr1	34396956	34406955	3	3	4	3	3	2	1	1	2	1	1	0	3	0.63	0	0	1.58	0	0	-1.58
chr1	34406956	35276355	3	3	4	3	3	3	1	1	2	1	1	1	3.17	0.41	-0.41	-0.41	2.04	-0.41	-0.41	-0.41
chr1	35276356	35286355	3	3	4	3	3	3	1	1	2	1	1	1	3.17	0.41	-0.41	-0.41	2.04	-0.41	-0.41	-0.41
chr1	35286356	36986555	3	3	4	3	3	3	1	1	2	1	1	1	3.17	0.41	-0.41	-0.41	2.04	-0.41	-0.41	-0.41
chr1	36986556	36996555	3	3	4	3	3	3	1	1	2	1	1	1	3.17	0.41	-0.41	-0.41	2.04	-0.41	-0.41	-0.41
chr1	36996556	37886555	3	3	4	3	3	3	1	1	2	1	1	1	3.17	0.41	-0.41	-0.41	2.04	-0.41	-0.41	-0.41
chr1	37886556	37896555	3	3	4	3	3	3	1	1	2	1	1	1	3.17	0.41	-0.41	-0.41	2.04	-0.41	-0.41	-0.41
chr1	37896556	48896555	3	3	4	3	3	3	1	1	2	1	1	1	3.17	0.41	-0.41	-0.41	2.04	-0.41	-0.41	-0.41
chr1	48896556	48906555	3	3	4	3	3	3	1	1	2	1	1	1	3.17	0.41	-0.41	-0.41	2.04	-0.41	-0.41	-0.41
chr1	48906556	50906555	3	3	4	3	3	3	1	1	2	1	1	1	3.17	0.41	-0.41	-0.41	2.04	-0.41	-0.41	-0.41
chr1	50906556	50916555	3	3	4	3	3	3	1	1	2	1	1	1	3.17	0.41	-0.41	-0.41	2.04	-0.41	-0.41	-0.41
chr1	50916556	53466555	3	3	4	3	3	3	1	1	2	1	1	1	3.17	0.41	-0.41	-0.41	2.04	-0.41	-0.41	-0.41
chr1	53466556	53476555	3	3	4	3	3	3	1	1	2	1	1	1	3.17	0.41	-0.41	-0.41	2.04	-0.41	-0.41	-0.41
chr1	53476556	59356955	1	1	2	1	1	1	0	0	0	0	0	0	1.17	0.41	-0.41	-0.41	2.04	-0.41	-0.41	-0.41
chr1	59356956	59816955	1	1	2	1	1	1	0	0	0	0	0	0	1.17	0.41	-0.41	-0.41	2.04	-0.41	-0.41	-0.41
chr1	59816956	59826955	1	1	2	1	1	1	0	0	0	0	0	0	1.17	0.41	-0.41	-0.41	2.04	-0.41	-0.41	-0.41
chr1	59826956	60506755	1	1	2	1	1	1	0	0	0	0	0	0	1.17	0.41	-0.41	-0.41	2.04	-0.41	-0.41	-0.41
chr1	60506756	60516755	1	1	2	1	1	1	0	0	0	0	0	0	1.17	0.41	-0.41	-0.41	2.04	-0.41	-0.41	-0.41
chr1	60516756	60596755	1	1	2	1	1	1	0	0	0	0	0	0	1.17	0.41	-0.41	-0.41	2.04	-0.41	-0.41	-0.41
chr1	60596756	60606755	1	1	2	1	1	1	0	0	0	0	0	0	1.17	0.41	-0.41	-0.41	2.04	-0.41	-0.41	-0.41
chr1	60606756	65207555	1	1	2	1	1	1	0	0	0	0	0	0	1.17	0.41	-0.41	-0.41	2.04	-0.41	-0.41	-0.41
chr1	65207556	65217555	1	1	2	1	1	1	0	0	0	0	0	0	1.17	0.41	-0.41	-0.41	2.04	-0.41	-0.41	-0.41
chr1	65217556	65747555	1	1	2	1	1	1	0	0	0	0	0	0	1.17	0.41	-0.41	-0.41	2.04	-0.41	-0.41	-0.41
chr1	65747556	65757555	1	1	2	1	1	1	0	0	0	0	0	0	1.17	0.41	-0.41	-0.41	2.04	-0.41	-0.41	-0.41
chr1	65757556	67068155	1	1	2	1	1	1	0	0	0	0	0	0	1.17	0.41	-0.41	-0.41	2.04	-0.41	-0.41	-0.41
chr1	67068156	67078155	1	1	2	1	1	1	0	0	0	0	0	0	1.17	0.41	-0.41	-0.41	2.04	-0.41	-0.41	-0.41
chr1	67078156	67118155	3	3	2	1	3	1	1	1	0	0	1	0	2.17	0.98	0.85	0.85	-0.17	-1.19	0.85	-1.19
chr1	67118156	67128155	3	3	2	1	3	1	1	1	0	0	1	0	2.17	0.98	0.85	0.85	-0.17	-1.19	0.85	-1.19
chr1	67128156	67267555	1	1	2	1	1	1	0	0	0	0	0	0	1.17	0.41	-0.41	-0.41	2.04	-0.41	-0.41	-0.41

chr1	67267556	67277555	1	1	2	1	1	1	0	0	0	0	0	0	1.17	0.41	-0.41	-0.41	2.04	-0.41	-0.41	-0.41
chr1	67277556	68938155	3	3	4	3	3	3	1	1	2	1	1	1	3.17	0.41	-0.41	-0.41	2.04	-0.41	-0.41	-0.41
chr1	68938156	68948155	3	3	4	3	3	3	1	1	2	1	1	1	3.17	0.41	-0.41	-0.41	2.04	-0.41	-0.41	-0.41
chr1	68948156	70558155	3	3	4	3	3	3	1	1	2	1	1	1	3.17	0.41	-0.41	-0.41	2.04	-0.41	-0.41	-0.41
chr1	70558156	70568155	3	3	4	3	3	3	1	1	2	1	1	1	3.17	0.41	-0.41	-0.41	2.04	-0.41	-0.41	-0.41
chr1	70568156	70908155	3	3	4	3	3	3	1	1	2	1	1	1	3.17	0.41	-0.41	-0.41	2.04	-0.41	-0.41	-0.41
chr1	70908156	70918155	3	3	4	3	3	3	1	1	2	1	1	1	3.17	0.41	-0.41	-0.41	2.04	-0.41	-0.41	-0.41
chr1	70918156	74341755	3	3	4	3	3	3	1	1	2	1	1	1	3.17	0.41	-0.41	-0.41	2.04	-0.41	-0.41	-0.41
chr1	74341756	85953155	3	3	3	3	3	3	1	1	1	1	1	1	3	0	0	0	0	0	0	0
chr1	85953156	85963155	3	3	3	3	3	3	1	1	1	1	1	1	3	0	0	0	0	0	0	0
chr1	85963156	85973355	3	3	3	6	3	3	1	1	1	3	1	1	3.5	1.22	-0.41	-0.41	-0.41	2.04	-0.41	-0.41
chr1	85973356	85983355	3	6	6	6	7	6	1	3	2	3	2	2	5.67	1.37	-1.95	0.24	0.24	0.24	0.98	0.24
chr1	85983356	86003155	5	6	6	6	7	6	2	3	2	3	2	2	6	0.63	-1.58	0	0	0	1.58	0
chr1	86003156	86013155	5	6	6	6	7	6	2	3	2	3	2	2	6	0.63	-1.58	0	0	0	1.58	0
chr1	86013156	86023355	5	6	6	3	7	6	2	3	2	1	2	2	5.5	1.38	-0.36	0.36	0.36	-1.81	1.09	0.36
chr1	86023356	86033355	5	3	3	3	3	3	2	1	1	1	1	1	3.33	0.82	2.04	-0.41	-0.41	-0.41	-0.41	-0.41
chr1	86033356	101712155	3	3	3	3	3	3	1	1	1	1	1	1	3	0	0	0	0	0	0	0
chr1	101712156	101722155	3	3	3	3	3	3	1	1	1	1	1	1	3	0	0	0	0	0	0	0
chr1	101722156	107919350	3	3	3	3	3	3	1	1	1	1	1	1	3	0	0	0	0	0	0	0
chr1	107919351	107929350	3	3	3	3	3	3	1	1	1	1	1	1	3	0	0	0	0	0	0	0
chr1	107929351	118574150	3	3	3	3	3	3	1	1	1	1	1	1	3	0	0	0	0	0	0	0
chr1	118574151	118584150	3	3	3	3	3	3	1	1	1	1	1	1	3	0	0	0	0	0	0	0
chr1	118584151	119064150	3	3	3	3	3	3	1	1	1	1	1	1	3	0	0	0	0	0	0	0
chr1	119064151	119074150	3	3	3	3	3	3	1	1	1	1	1	1	3	0	0	0	0	0	0	0
chr1	119074151	120523550	3	3	3	3	3	3	1	1	1	1	1	1	3	0	0	0	0	0	0	0
chr1	120523551	120533550	3	3	5	3	3	3	1	1	1	1	1	1	3.33	0.82	-0.41	-0.41	2.04	-0.41	-0.41	-0.41
chr1	120533551	121133945	4	4	5	5	4	4	2	2	1	1	1	2	4.33	0.52	-0.65	-0.65	1.29	1.29	-0.65	-0.65
chr1	121133946	121143945	4	4	5	5	4	4	2	2	1	1	1	2	4.33	0.52	-0.65	-0.65	1.29	1.29	-0.65	-0.65
chr1	121143946	121153945	3	4	5	5	3	3	1	2	1	1	1	1	3.83	0.98	-0.85	0.17	1.19	1.19	-0.85	-0.85
chr1	121153946	121383945	3	3	3	3	3	3	1	1	0	1	1	1	3	0	0	0	0	0	0	0
chr1	121383946	121393945	3	3	3	3	3	3	1	1	0	1	1	1	3	0	0	0	0	0	0	0
chr1	121393946	121465745	3	3	3	3	3	3	1	1	0	1	1	1	3	0	0	0	0	0	0	0
chr1	121465746	142545540	3	3	3	3	3	3	1	1	0	1	1	1	3	0	0	0	0	0	0	0
chr1	142545541	142645540	4	5	4	4	5	4	2	2	0	0	1	1	4.33	0.52	-0.65	1.29	-0.65	-0.65	1.29	-0.65
chr1	142645541	142655540	4	5	4	4	5	4	2	2	0	0	1	1	4.33	0.52	-0.65	1.29	-0.65	-0.65	1.29	-0.65
chr1	142655541	142905735	4	5	4	4	4	4	2	2	0	0	1	1	4.17	0.41	-0.41	2.04	-0.41	-0.41	-0.41	-0.41
chr1	142905736	142915735	4	5	4	4	4	4	2	2	0	0	1	1	4.17	0.41	-0.41	2.04	-0.41	-0.41	-0.41	-0.41
chr1	142915736	142965735	4	5	4	4	5	5	2	2	0	0	2	2	4.5	0.55	-0.91	0.91	-0.91	-0.91	0.91	0.91
chr1	142965736	143125930	4	5	4	4	5	5	2	2	0	0	2	2	4.5	0.55	-0.91	0.91	-0.91	-0.91	0.91	0.91
chr1	143125931	143285930	4	4	3	3	3	4	2	2	0	1	1	1	3.5	0.55	0.91	0.91	-0.91	-0.91	-0.91	0.91
chr1	143285931	143346125	4	4	3	3	3	4	2	2	0	1	1	1	3.5	0.55	0.91	0.91	-0.91	-0.91	-0.91	0.91
chr1	143346126	143536125	4	6	5	5	6	6	2	3	1	2	2	3	5.33	0.82	-1.63	0.82	-0.41	-0.41	0.82	0.82
chr1	143536126	143646325	4	6	5	5	6	6	2	3	1	2	2	3	5.33	0.82	-1.63	0.82	-0.41	-0.41	0.82	0.82
chr1	143646326	143966520	7	8	8	8	8	8	2	3	0	2	4	4	7.83	0.41	-2.04	0.41	0.41	0.41	0.41	0.41
chr1	143966521	143976520	7	8	8	8	8	8	2	3	0	2	4	4	7.83	0.41	-2.04	0.41	0.41	0.41	0.41	0.41
chr1	143976521	144006520	6	8	8	8	8	8	1	3	0	0	4	4	7.67	0.82	-2.04	0.41	0.41	0.41	0.41	0.41
chr1	144006521	144076520	6	8	8	8	8	7	1	4	0	0	4	3	7.5	0.84	-1.79	0.6	0.6	0.6	0.6	-0.6
chr1	144076521	144086520	6	8	8	8	8	7	1	4	0	0	4	3	7.5	0.84	-1.79	0.6	0.6	0.6	0.6	-0.6
chr1	144086521	144216715	6	8	8	8	8	8	3	4	0	0	4	4	7.67	0.82	-2.04	0.41	0.41	0.41	0.41	0.41
chr1	144216716	144276910	6	8	8	8	8	8	3	4	0	0	4	4	7.67	0.82	-2.04	0.41	0.41	0.41	0.41	0.41
chr1	144276911	144708900	4	5	5	5	5	4	2	2	1	1	2	1	4.67	0.52	-1.29	0.65	0.65	0.65	0.65	-1.29
chr1	144708901	144818295	4	5	5	-	5	4	2	2	1	-	2	1	-	-	-	-	-	-	-	-
chr1	144818296	145079095	5	6	7	6	6	6	2	2	0	1	1	2	6	0.63	-1.58	0	1.58	0	0	0
chr1	145079096	145089095	5	6	7	6	6	6	2	2	0	1	1	2	6	0.63	-1.58	0	1.58	0	0	0
chr1	145089096	145099095	5	6	7	6	8	8	2	2	0	1	2	2	6.67	1.21	-1.38	-0.55	0.28	-0.55	1.1	1.1
chr1	145099096	145109095	5	8	7	6	8	8	2	2	0	1	2	2	7	1.26	-1.58	0.79	0	-0.79	0.79	0.79
chr1	145109096	145119095	7	8	7	6	8	8	2	2	0	1	2	2	7.33	0.82	-0.41	0.82	-0.41	-1.63	0.82	0.82
chr1	145119096	145379295	7	8	8	8	8	8	2	2	0	1	2	2	7.83	0.41	-2.04	0.41	0.41	0.41	0.41	0.41
chr1	145379296	145389295	7	8	8	8	8	8	2	2	0	1	2	2	7.83	0.41	-2.04	0.41	0.41	0.41	0.41	0.41
chr1	145389296	146399885	7	8	8	8	8	8	2	2	0	1	2	2	7.83	0.41	-2.04	0.41	0.41	0.41	0.41	0.41
chr1	146399886	146409885	7	8	8	8	8	8	2	2	0	1	2	2	7.83	0.41	-2.04	0.41	0.41	0.41	0.41	0.41
chr1	146409886	146459885	7	8	8	8	8	7	2	2	0	1	2	3	7.67	0.52	-1.29	0.65	0.65	0.65	0.65	-1.29
chr1	146459886	146469885	7	8	8	8	8	7	2	2	0	1	2	3	7.67	0.52	-1.29	0.65	0.65	0.65	0.65	-1.29
chr1	146469886	147929085	7	8	8	8	8	8	2	2	0	1	2	2	7.83	0.41	-2.04	0.41	0.41	0.41	0.41	0.41
chr1	147929086	147939085	7	8	8	8	8	8	2	2	0	1	2	2	7.83	0.41	-2.04	0.41	0.41	0.41	0.41	0.41
chr1	147939086	147970085	7	5	5	5	8	6	2	1	0	0	2	1	6	1.26	0.79	-0.79	-0.79	-0.79	1.58	0
chr1	147970086	147980485	7	5	5	5	8	6	2	1	0	0	2	1	6	1.26	0.79	-0.79	-0.79	-0.79	1.58	0
chr1	147980486	148019885	4	5	5	5	5	6	2	1	0	0	2	1	5	0.63	-1.58	0	0	0	0	1.58
chr1	148019886	148180080	4	5	5	5	5	6	2	1	0	0	2	1	5	0.63	-1.58	0	0	0	0	1.58
chr1	148180081	149031070	6	8	8	8	8	8	2	3	1	1	3	2	7.67	0.82	-2.04	0.41	0.41	0.41	0.41	0.41
chr1	149031071	149041070	6	8	8	8	8	8	2	3	1	1	3	2	7.67	0.82	-2.04	0.41	0.41	0.41	0.41	0.41
chr1	149041071	149201670	8	8	8	8	8	8	3	2	0	1	0	4	8	0	0	0	0	0	0	0
chr1	149201671	149211670	8	8	8	8	8	8	3	2	0	1	0	4	8	0	0	0	0	0	0	0
chr1	149211671	149271070	8	8	8	8	8	8	3	2	0	1	0	1	8	0	0	0	0	0	0	0
chr1	149271071	149281070	8	8	8	8	8	8	3	2	0	1	0	1	8	0	0	0	0	0	0	0
chr1	149281071	149451070	7	8	8	8	8	8	2	1	0	1	1	1	7.83	0.41	-2.04	0.41	0.41	0.41	0.41	0.41

chr1	149451071	149511270	7	8	8	8	8	8	2	1	0	1	1	1	7.83	0.41	-2.04	0.41	0.41	0.41	0.41	0.41
chr1	149511271	149522070	7	6	8	6	6	8	2	2	0	1	1	1	6.83	0.98	0.17	-0.85	1.19	-0.85	-0.85	1.19
chr1	149522071	149532470	7	6	6	6	6	6	2	2	0	1	1	1	6.17	0.41	2.04	-0.41	-0.41	-0.41	-0.41	-0.41
chr1	149532471	149702870	5	6	6	6	6	6	2	2	0	1	1	1	5.83	0.41	-2.04	0.41	0.41	0.41	0.41	0.41
chr1	149702871	149712870	5	6	6	6	6	6	2	2	0	1	1	1	5.83	0.41	-2.04	0.41	0.41	0.41	0.41	0.41
chr1	149712871	149743670	6	8	8	8	8	7	2	2	0	1	3	3	7.5	0.84	-1.79	0.6	0.6	0.6	0.6	-0.6
chr1	149743671	149753670	6	8	8	8	8	7	2	2	0	1	3	3	7.5	0.84	-1.79	0.6	0.6	0.6	0.6	-0.6
chr1	149753671	149772870	7	8	8	8	8	8	2	2	0	1	3	2	7.83	0.41	-2.04	0.41	0.41	0.41	0.41	0.41
chr1	149772871	149782870	7	8	8	8	8	8	2	2	0	1	3	2	7.83	0.41	-2.04	0.41	0.41	0.41	0.41	0.41
chr1	149782871	152152870	7	8	8	8	8	8	2	2	0	1	2	2	7.83	0.41	-2.04	0.41	0.41	0.41	0.41	0.41
chr1	152152871	152162870	7	8	8	8	8	8	2	2	0	1	2	2	7.83	0.41	-2.04	0.41	0.41	0.41	0.41	0.41
chr1	152162871	153462870	7	8	8	8	8	8	2	2	0	1	2	2	7.83	0.41	-2.04	0.41	0.41	0.41	0.41	0.41
chr1	153462871	153472870	7	8	8	8	8	8	2	2	0	1	2	2	7.83	0.41	-2.04	0.41	0.41	0.41	0.41	0.41
chr1	153472871	156772870	7	8	8	8	8	8	2	2	0	1	2	2	7.83	0.41	-2.04	0.41	0.41	0.41	0.41	0.41
chr1	156772871	156782870	7	8	8	8	8	8	2	2	0	1	2	2	7.83	0.41	-2.04	0.41	0.41	0.41	0.41	0.41
chr1	156782871	158292870	7	8	8	8	8	8	2	2	0	1	1	2	7.83	0.41	-2.04	0.41	0.41	0.41	0.41	0.41
chr1	158292871	158302870	7	8	8	8	8	8	2	2	0	1	1	2	7.83	0.41	-2.04	0.41	0.41	0.41	0.41	0.41
chr1	158302871	158792870	7	8	8	8	8	8	2	2	0	1	1	2	7.83	0.41	-2.04	0.41	0.41	0.41	0.41	0.41
chr1	158792871	158802870	7	8	8	8	8	8	2	2	0	1	1	2	7.83	0.41	-2.04	0.41	0.41	0.41	0.41	0.41
chr1	158802871	160966070	7	8	8	8	8	8	2	2	0	1	2	2	7.83	0.41	-2.04	0.41	0.41	0.41	0.41	0.41
chr1	160966071	160976070	7	8	8	8	8	8	2	2	0	1	2	2	7.83	0.41	-2.04	0.41	0.41	0.41	0.41	0.41
chr1	160976071	161226270	7	8	8	8	8	8	2	2	0	1	2	2	7.83	0.41	-2.04	0.41	0.41	0.41	0.41	0.41
chr1	161226271	162136070	7	8	8	8	8	8	2	2	0	1	2	2	7.83	0.41	-2.04	0.41	0.41	0.41	0.41	0.41
chr1	162136071	162146070	7	8	8	8	8	8	2	2	0	1	2	2	7.83	0.41	-2.04	0.41	0.41	0.41	0.41	0.41
chr1	162146071	169215470	7	8	8	8	8	8	2	2	0	1	2	2	7.83	0.41	-2.04	0.41	0.41	0.41	0.41	0.41
chr1	169215471	169225470	7	8	8	8	8	8	2	2	0	1	2	2	7.83	0.41	-2.04	0.41	0.41	0.41	0.41	0.41
chr1	169225471	169265470	7	5	3	5	5	6	2	1	0	0	1	1	5.17	1.33	1.38	-0.13	-1.63	-0.13	-0.13	0.63
chr1	169265471	169275470	7	5	3	5	5	6	2	1	0	0	1	1	5.17	1.33	1.38	-0.13	-1.63	-0.13	-0.13	0.63
chr1	169275471	170066070	7	8	8	8	8	8	2	2	0	1	2	2	7.83	0.41	-2.04	0.41	0.41	0.41	0.41	0.41
chr1	170066071	170076070	7	8	8	8	8	8	2	2	0	1	2	2	7.83	0.41	-2.04	0.41	0.41	0.41	0.41	0.41
chr1	170076071	171036070	7	8	8	8	8	8	2	2	0	1	1	2	7.83	0.41	-2.04	0.41	0.41	0.41	0.41	0.41
chr1	171036071	171046070	7	8	8	8	8	8	2	2	0	1	1	2	7.83	0.41	-2.04	0.41	0.41	0.41	0.41	0.41
chr1	171046071	186672070	7	8	8	8	8	8	2	2	0	1	2	2	7.83	0.41	-2.04	0.41	0.41	0.41	0.41	0.41
chr1	186672071	186682070	7	8	8	8	8	8	2	2	0	1	2	2	7.83	0.41	-2.04	0.41	0.41	0.41	0.41	0.41
chr1	186682071	192413670	7	8	8	8	8	8	2	2	0	1	2	2	7.83	0.41	-2.04	0.41	0.41	0.41	0.41	0.41
chr1	192413671	192423670	7	8	8	8	8	8	2	2	0	1	2	2	7.83	0.41	-2.04	0.41	0.41	0.41	0.41	0.41
chr1	192423671	193683670	7	8	8	8	8	8	2	2	0	1	2	2	7.83	0.41	-2.04	0.41	0.41	0.41	0.41	0.41
chr1	193683671	193693670	7	8	8	8	8	8	2	2	0	1	2	2	7.83	0.41	-2.04	0.41	0.41	0.41	0.41	0.41
chr1	193693671	195933670	7	8	8	8	8	8	2	2	0	1	2	2	7.83	0.41	-2.04	0.41	0.41	0.41	0.41	0.41
chr1	195933671	195943670	7	8	8	8	8	8	2	2	0	1	2	2	7.83	0.41	-2.04	0.41	0.41	0.41	0.41	0.41
chr1	195943671	206326065	7	8	8	8	8	8	2	2	0	1	2	2	7.83	0.41	-2.04	0.41	0.41	0.41	0.41	0.41
chr1	206326066	206486260	7	8	8	8	8	8	2	2	0	1	2	2	7.83	0.41	-2.04	0.41	0.41	0.41	0.41	0.41
chr1	206486261	206536260	7	8	8	8	8	8	2	2	0	1	1	2	7.83	0.41	-2.04	0.41	0.41	0.41	0.41	0.41
chr1	206536261	206546260	7	8	8	8	8	8	2	2	0	1	1	2	7.83	0.41	-2.04	0.41	0.41	0.41	0.41	0.41
chr1	206546261	212087060	7	8	8	8	8	8	2	2	0	1	2	2	7.83	0.41	-2.04	0.41	0.41	0.41	0.41	0.41
chr1	212087061	212097060	7	8	8	8	8	8	2	2	0	1	2	2	7.83	0.41	-2.04	0.41	0.41	0.41	0.41	0.41
chr1	212097061	212627060	7	8	8	8	8	8	2	2	0	1	2	2	7.83	0.41	-2.04	0.41	0.41	0.41	0.41	0.41
chr1	212627061	212637060	7	8	8	8	8	8	2	2	0	1	2	2	7.83	0.41	-2.04	0.41	0.41	0.41	0.41	0.41
chr1	212637061	223717060	7	8	8	8	8	8	2	2	0	1	2	2	7.83	0.41	-2.04	0.41	0.41	0.41	0.41	0.41
chr1	223717061	223727060	7	8	8	8	8	8	2	2	0	1	2	2	7.83	0.41	-2.04	0.41	0.41	0.41	0.41	0.41
chr1	223727061	223817255	7	8	8	8	8	8	2	2	0	1	2	2	7.83	0.41	-2.04	0.41	0.41	0.41	0.41	0.41
chr1	223817256	223827255	7	8	8	8	8	8	2	2	0	1	2	2	7.83	0.41	-2.04	0.41	0.41	0.41	0.41	0.41
chr1	223827256	228727255	7	8	8	8	8	8	2	2	0	1	2	2	7.83	0.41	-2.04	0.41	0.41	0.41	0.41	0.41
chr1	228727256	228737255	7	8	8	8	8	8	2	2	0	1	2	2	7.83	0.41	-2.04	0.41	0.41	0.41	0.41	0.41
chr1	228737256	228777255	7	7	8	8	8	8	2	2	0	1	2	2	7.67	0.52	-1.29	-1.29	0.65	0.65	0.65	0.65
chr1	228777256	228787255	7	7	8	8	8	8	2	2	0	1	2	2	7.67	0.52	-1.29	-1.29	0.65	0.65	0.65	0.65
chr1	228787256	234489455	7	8	8	8	8	8	2	2	0	1	2	2	7.83	0.41	-2.04	0.41	0.41	0.41	0.41	0.41
chr1	234489456	234499455	7	8	8	8	8	8	2	2	0	1	2	2	7.83	0.41	-2.04	0.41	0.41	0.41	0.41	0.41
chr1	234499456	234900855	7	8	8	8	8	8	2	2	0	1	2	2	7.83	0.41	-2.04	0.41	0.41	0.41	0.41	0.41
chr1	234900856	234910855	7	8	8	8	8	8	2	2	0	1	2	2	7.83	0.41	-2.04	0.41	0.41	0.41	0.41	0.41
chr1	234910856	234950855	7	7	8	8	8	5	2	2	0	1	4	2	7.17	1.17	-0.14	-0.14	0.71	0.71	0.71	-1.85
chr1	234950856	234960855	7	7	8	8	8	5	2	2	0	1	4	2	7.17	1.17	-0.14	-0.14	0.71	0.71	0.71	-1.85
chr1	234960856	237080050	7	8	8	8	8	8	2	2	0	1	2	2	7.83	0.41	-2.04	0.41	0.41	0.41	0.41	0.41
chr1	237080051	237090050	7	8	8	8	8	8	2	2	0	1	2	2	7.83	0.41	-2.04	0.41	0.41	0.41	0.41	0.41
chr1	237090051	241640050	7	8	8	8	8	8	2	2	0	1	2	2	7.83	0.41	-2.04	0.41	0.41	0.41	0.41	0.41
chr1	241640051	241650050	7	8	8	8	8	8	2	2	0	1	2	2	7.83	0.41	-2.04	0.41	0.41	0.41	0.41	0.41
chr1	241650051	247570050	7	8	8	8	8	8	2	2	0	1	2	2	7.83	0.41	-2.04	0.41	0.41	0.41	0.41	0.41
chr1	247570051	247580050	7	8	8	8	8	8	2	2	0	1	2	2	7.83	0.41	-2.04	0.41	0.41	0.41	0.41	0.41
chr1	247580051	248300050	7	8	8	8	8	8	2	2	0	1	1	2	7.83	0.41	-2.04	0.41	0.41	0.41	0.41	0.41
chr1	248300051	248310050	7	8	8	8	8	8	2	2	0	1	1	2	7.83	0.41	-2.04	0.41	0.41	0.41	0.41	0.41
chr1	248310051	248400050	7	8	8	8	8	8	2	2	0	1	2	2	7.83	0.41	-2.04	0.41	0.41	0.41	0.41	0.41
chr1	248400051	248410050	7	8	8	8	8	8	2	2	0	1	2	2	7.83	0.41	-2.04	0.41	0.41	0.41	0.41	0.41
chr1	248410051	248730050	7	8	8	8	8	8	2	2	0	1	2	2	7.83	0.41	-2.04	0.41	0.41	0.41	0.41	0.41
chr1	248730051	248740050	7	8	8	8	8	8	2	2	0	1	2	2	7.83	0.41	-2.04	0.41	0.41	0.41	0.41	0.41
chr1	248740051	249220650	7	8	8	8	8	8	2	2	0	1	2	2	7.83	0.41	-2.04	0.41	0.41	0.41	0.41	0.41
chr2	19801	3189800	4	5	5	5	5	5	2	2	2	2	2	2	4.83	0.41	-2.04	0.41	0.41	0.41	0.41	0.41

chr2	3189801	3199800	4	5	5	5	5	5	2	2	2	2	2	2	4.83	0.41	-2.04	0.41	0.41	0.41	0.41	0.41
chr2	3199801	3309800	4	5	5	5	5	5	2	2	2	2	2	2	4.83	0.41	-2.04	0.41	0.41	0.41	0.41	0.41
chr2	3309801	3319800	4	5	5	5	5	5	2	2	2	2	2	2	4.83	0.41	-2.04	0.41	0.41	0.41	0.41	0.41
chr2	3319801	3619995	4	5	5	5	5	5	2	2	2	2	2	2	4.83	0.41	-2.04	0.41	0.41	0.41	0.41	0.41
chr2	3619996	3629995	4	5	5	5	5	5	2	2	2	2	2	2	4.83	0.41	-2.04	0.41	0.41	0.41	0.41	0.41
chr2	3629996	3639995	4	5	5	5	5	5	2	2	2	2	2	2	4.83	0.41	-2.04	0.41	0.41	0.41	0.41	0.41
chr2	3639996	3679995	4	5	5	5	5	5	2	2	2	2	2	2	4.83	0.41	-2.04	0.41	0.41	0.41	0.41	0.41
chr2	3679996	3689995	4	5	5	5	5	5	2	2	2	2	2	2	4.83	0.41	-2.04	0.41	0.41	0.41	0.41	0.41
chr2	3689996	6870190	4	5	5	5	5	5	2	2	2	2	2	2	4.83	0.41	-2.04	0.41	0.41	0.41	0.41	0.41
chr2	6870191	6880190	4	5	5	5	5	5	2	2	2	2	2	2	4.83	0.41	-2.04	0.41	0.41	0.41	0.41	0.41
chr2	6880191	11820190	4	5	5	5	5	5	2	2	2	2	2	2	4.83	0.41	-2.04	0.41	0.41	0.41	0.41	0.41
chr2	11820191	11830190	4	5	5	5	5	5	2	2	2	2	2	2	4.83	0.41	-2.04	0.41	0.41	0.41	0.41	0.41
chr2	11830191	13070190	4	4	4	4	4	4	2	2	2	2	2	2	4	0	0	0	0	0	0	0
chr2	13070191	13080190	4	4	4	4	4	4	2	2	2	2	2	2	4	0	0	0	0	0	0	0
chr2	13080191	15350190	4	5	5	5	5	5	2	2	2	2	2	2	4.83	0.41	-2.04	0.41	0.41	0.41	0.41	0.41
chr2	15350191	15360190	4	5	5	5	5	5	2	2	2	2	2	2	4.83	0.41	-2.04	0.41	0.41	0.41	0.41	0.41
chr2	15360191	27195580	4	5	5	5	5	5	2	2	2	2	2	2	4.83	0.41	-2.04	0.41	0.41	0.41	0.41	0.41
chr2	27195581	27205580	4	5	5	5	5	5	2	2	2	2	2	2	4.83	0.41	-2.04	0.41	0.41	0.41	0.41	0.41
chr2	27205581	27915580	4	5	5	5	5	5	2	2	2	2	2	2	4.83	0.41	-2.04	0.41	0.41	0.41	0.41	0.41
chr2	27915581	27925580	4	5	5	5	5	5	2	2	2	2	2	2	4.83	0.41	-2.04	0.41	0.41	0.41	0.41	0.41
chr2	27925581	29265580	4	5	5	5	5	5	2	2	2	2	2	2	4.83	0.41	-2.04	0.41	0.41	0.41	0.41	0.41
chr2	29265581	29275580	4	5	5	5	5	5	2	2	2	2	2	2	4.83	0.41	-2.04	0.41	0.41	0.41	0.41	0.41
chr2	29275581	29515780	4	5	5	5	5	5	2	2	2	2	2	2	4.83	0.41	-2.04	0.41	0.41	0.41	0.41	0.41
chr2	29515781	29535980	4	5	5	5	5	5	2	2	2	2	2	2	4.83	0.41	-2.04	0.41	0.41	0.41	0.41	0.41
chr2	29535981	29545980	4	5	5	5	5	5	2	2	2	2	2	2	4.83	0.41	-2.04	0.41	0.41	0.41	0.41	0.41
chr2	29545981	30155980	4	5	5	5	5	5	2	2	2	2	2	2	4.83	0.41	-2.04	0.41	0.41	0.41	0.41	0.41
chr2	30155981	30165980	4	5	5	5	5	5	2	2	2	2	2	2	4.83	0.41	-2.04	0.41	0.41	0.41	0.41	0.41
chr2	30165981	30245780	4	5	5	5	5	5	2	2	2	2	2	2	4.83	0.41	-2.04	0.41	0.41	0.41	0.41	0.41
chr2	30245781	30255780	4	5	5	5	5	5	2	2	2	2	2	2	4.83	0.41	-2.04	0.41	0.41	0.41	0.41	0.41
chr2	30255781	30275580	4	5	5	5	5	5	2	2	2	2	2	2	4.83	0.41	-2.04	0.41	0.41	0.41	0.41	0.41
chr2	30275581	30285580	4	5	5	5	5	5	2	2	2	2	2	2	4.83	0.41	-2.04	0.41	0.41	0.41	0.41	0.41
chr2	30285581	31105580	4	5	5	5	5	5	2	2	2	2	2	2	4.83	0.41	-2.04	0.41	0.41	0.41	0.41	0.41
chr2	31105581	31115580	4	5	5	5	5	5	2	2	2	2	2	2	4.83	0.41	-2.04	0.41	0.41	0.41	0.41	0.41
chr2	31115581	31565580	4	5	5	5	5	5	2	2	2	2	2	2	4.83	0.41	-2.04	0.41	0.41	0.41	0.41	0.41
chr2	31565581	31575580	4	5	5	5	5	5	2	2	2	2	2	2	4.83	0.41	-2.04	0.41	0.41	0.41	0.41	0.41
chr2	31575581	33870020	4	5	5	5	5	5	2	2	2	2	2	2	4.83	0.41	-2.04	0.41	0.41	0.41	0.41	0.41
chr2	33870021	33880020	4	5	5	5	5	5	2	2	2	2	2	2	4.83	0.41	-2.04	0.41	0.41	0.41	0.41	0.41
chr2	33880021	36580020	4	5	5	5	5	5	2	2	2	2	2	2	4.83	0.41	-2.04	0.41	0.41	0.41	0.41	0.41
chr2	36580021	36590020	4	5	5	5	5	5	2	2	2	2	2	2	4.83	0.41	-2.04	0.41	0.41	0.41	0.41	0.41
chr2	36590021	37950020	4	5	5	5	5	5	2	2	2	2	2	2	4.83	0.41	-2.04	0.41	0.41	0.41	0.41	0.41
chr2	37950021	37960020	4	5	5	5	5	5	2	2	2	2	2	2	4.83	0.41	-2.04	0.41	0.41	0.41	0.41	0.41
chr2	37960021	38000020	4	5	5	5	6	6	2	2	2	2	3	3	5.17	0.75	-1.55	-0.22	-0.22	-0.22	1.11	1.11
chr2	38000021	38010020	4	5	5	5	6	6	2	2	2	2	3	3	5.17	0.75	-1.55	-0.22	-0.22	-0.22	1.11	1.11
chr2	38010021	40080020	4	5	5	5	5	5	2	2	2	2	2	2	4.83	0.41	-2.04	0.41	0.41	0.41	0.41	0.41
chr2	40080021	40090020	4	5	5	5	5	5	2	2	2	2	2	2	4.83	0.41	-2.04	0.41	0.41	0.41	0.41	0.41
chr2	40090021	41640020	4	5	5	5	5	5	2	2	2	2	2	2	4.83	0.41	-2.04	0.41	0.41	0.41	0.41	0.41
chr2	41640021	41650020	4	5	5	5	5	5	2	2	2	2	2	2	4.83	0.41	-2.04	0.41	0.41	0.41	0.41	0.41
chr2	41650021	42300020	4	5	5	5	5	5	2	2	2	2	2	2	4.83	0.41	-2.04	0.41	0.41	0.41	0.41	0.41
chr2	42300021	42310020	4	5	5	5	5	5	2	2	2	2	2	2	4.83	0.41	-2.04	0.41	0.41	0.41	0.41	0.41
chr2	42310021	42530020	4	5	5	5	6	5	2	2	2	2	2	2	5	0.63	-1.58	0	0	0	1.58	0
chr2	42530021	42540020	4	5	5	5	6	5	2	2	2	2	2	2	5	0.63	-1.58	0	0	0	1.58	0
chr2	42540021	49161420	4	5	5	5	5	5	2	2	2	2	2	2	4.83	0.41	-2.04	0.41	0.41	0.41	0.41	0.41
chr2	49161421	49171420	4	5	5	5	5	5	2	2	2	2	2	2	4.83	0.41	-2.04	0.41	0.41	0.41	0.41	0.41
chr2	49171421	53891420	4	5	5	5	5	5	2	2	2	2	2	2	4.83	0.41	-2.04	0.41	0.41	0.41	0.41	0.41
chr2	53891421	53901420	4	5	5	5	5	5	2	2	2	2	2	2	4.83	0.41	-2.04	0.41	0.41	0.41	0.41	0.41
chr2	53901421	54581620	4	5	5	5	5	5	2	2	2	2	2	2	4.83	0.41	-2.04	0.41	0.41	0.41	0.41	0.41
chr2	54581621	54591620	4	5	5	5	5	5	2	2	2	2	2	2	4.83	0.41	-2.04	0.41	0.41	0.41	0.41	0.41
chr2	54591621	54602020	4	5	6	6	6	6	2	2	2	2	2	2	5.5	0.84	-1.79	-0.6	0.6	0.6	0.6	0.6
chr2	54602021	55121620	4	6	6	6	6	6	2	2	2	2	2	2	5.67	0.82	-2.04	0.41	0.41	0.41	0.41	0.41
chr2	55121621	55131620	4	6	6	6	6	6	2	2	2	2	2	2	5.67	0.82	-2.04	0.41	0.41	0.41	0.41	0.41
chr2	55131621	55151420	4	6	5	6	6	6	2	2	2	2	2	2	5.5	0.84	-1.79	0.6	-0.6	0.6	0.6	0.6
chr2	55151421	55161420	4	6	5	6	6	6	2	2	2	2	2	2	5.5	0.84	-1.79	0.6	-0.6	0.6	0.6	0.6
chr2	55161421	57411420	4	5	5	5	5	5	2	2	2	2	2	2	4.83	0.41	-2.04	0.41	0.41	0.41	0.41	0.41
chr2	57411421	57421420	4	5	5	5	5	5	2	2	2	2	2	2	4.83	0.41	-2.04	0.41	0.41	0.41	0.41	0.41
chr2	57421421	57461420	4	5	5	5	4	5	2	2	2	2	2	2	4.67	0.52	-1.29	0.65	0.65	0.65	-1.29	0.65
chr2	57461421	57471420	4	5	5	5	4	5	2	2	2	2	2	2	4.67	0.52	-1.29	0.65	0.65	0.65	-1.29	0.65
chr2	57471421	60481420	4	5	5	5	5	5	2	2	2	2	2	2	4.83	0.41	-2.04	0.41	0.41	0.41	0.41	0.41
chr2	60481421	60491420	4	5	5	5	5	5	2	2	2	2	2	2	4.83	0.41	-2.04	0.41	0.41	0.41	0.41	0.41
chr2	60491421	75973020	4	5	5	5	5	5	2	2	2	2	2	2	4.83	0.41	-2.04	0.41	0.41	0.41	0.41	0.41
chr2	75973021	75983020	4	5	5	5	5	5	2	2	2	2	2	2	4.83	0.41	-2.04	0.41	0.41	0.41	0.41	0.41
chr2	75983021	84475220	4	5	5	5	5	5	2	2	2	2	2	2	4.83	0.41	-2.04	0.41	0.41	0.41	0.41	0.41
chr2	84475221	84485220	4	5	5	5	5	5	2	2	2	2	2	2	4.83	0.41	-2.04	0.41	0.41	0.41	0.41	0.41
chr2	84485221	87565420	4	5	5	5	5	5	2	2	2	2	2	2	4.83	0.41	-2.04	0.41	0.41	0.41	0.41	0.41
chr2	87565421	87575420	4	5	5	5	5	5	2	2	2	2	2	2	4.83	0.41	-2.04	0.41	0.41	0.41	0.41	0.41
chr2	87575421	87625420	4	6	7	5	7	7	2	2	0	2	2	2	6	1.26	-1.58	0	0.79	-0.79	0.79	0.79
chr2	87625421	87635420	4	6	7	5	7	7	2	2	0	2	2	2	6	1.26	-1.58	0	0.79	-0.79	0.79	0.79

chr2	87635421	87726215	4	6	5	5	5	6	2	2	1	2	2	2	5.17	0.75	-1.55	1.11	-0.22	-0.22	-0.22	1.11
chr2	87726216	87736215	4	6	5	5	5	6	2	2	1	2	2	2	5.17	0.75	-1.55	1.11	-0.22	-0.22	-0.22	1.11
chr2	87736216	89475615	4	5	5	5	5	5	2	2	1	2	2	2	4.83	0.41	-2.04	0.41	0.41	0.41	0.41	0.41
chr2	89475616	89485615	4	5	5	5	5	5	2	2	1	2	2	2	4.83	0.41	-2.04	0.41	0.41	0.41	0.41	0.41
chr2	89485616	90275810	4	5	5	5	5	5	2	2	1	2	2	2	4.83	0.41	-2.04	0.41	0.41	0.41	0.41	0.41
chr2	90275811	90285810	4	5	5	5	5	5	2	2	1	2	2	2	4.83	0.41	-2.04	0.41	0.41	0.41	0.41	0.41
chr2	90285811	90486010	4	6	5	5	6	5	2	2	1	2	2	2	5.17	0.75	-1.55	1.11	-0.22	-0.22	1.11	-0.22
chr2	90486011	96809800	4	6	5	5	6	5	2	2	1	2	2	2	5.17	0.75	-1.55	1.11	-0.22	-0.22	1.11	-0.22
chr2	96809801	102841400	4	5	5	5	5	5	2	2	2	2	2	2	4.83	0.41	-2.04	0.41	0.41	0.41	0.41	0.41
chr2	102841401	102851400	4	5	5	5	5	5	2	2	2	2	2	2	4.83	0.41	-2.04	0.41	0.41	0.41	0.41	0.41
chr2	102851401	103991400	4	5	5	5	5	5	2	2	2	2	2	2	4.83	0.41	-2.04	0.41	0.41	0.41	0.41	0.41
chr2	103991401	104001400	4	5	5	5	5	5	2	2	2	2	2	2	4.83	0.41	-2.04	0.41	0.41	0.41	0.41	0.41
chr2	104001401	104061400	4	5	5	5	4	5	2	2	2	2	2	2	4.67	0.52	-1.29	0.65	0.65	0.65	-1.29	0.65
chr2	104061401	104071400	4	5	5	5	4	5	2	2	2	2	2	2	4.67	0.52	-1.29	0.65	0.65	0.65	-1.29	0.65
chr2	104071401	108801400	4	5	5	5	5	5	2	2	2	2	2	2	4.83	0.41	-2.04	0.41	0.41	0.41	0.41	0.41
chr2	108801401	108811400	4	5	5	5	5	5	2	2	2	2	2	2	4.83	0.41	-2.04	0.41	0.41	0.41	0.41	0.41
chr2	108811401	109051200	4	5	5	5	5	5	2	2	2	2	2	2	4.83	0.41	-2.04	0.41	0.41	0.41	0.41	0.41
chr2	109051201	109461400	4	5	5	5	5	5	2	2	2	2	2	2	4.83	0.41	-2.04	0.41	0.41	0.41	0.41	0.41
chr2	109461401	109471400	4	5	5	5	5	5	2	2	2	2	2	2	4.83	0.41	-2.04	0.41	0.41	0.41	0.41	0.41
chr2	109471401	114764995	4	5	5	5	5	5	2	2	2	2	2	2	4.83	0.41	-2.04	0.41	0.41	0.41	0.41	0.41
chr2	114764996	114774995	4	5	5	5	5	5	2	2	2	2	2	2	4.83	0.41	-2.04	0.41	0.41	0.41	0.41	0.41
chr2	114774996	127776595	4	5	5	5	5	5	2	2	2	2	2	2	4.83	0.41	-2.04	0.41	0.41	0.41	0.41	0.41
chr2	127776596	127786595	4	5	5	5	5	5	2	2	2	2	2	2	4.83	0.41	-2.04	0.41	0.41	0.41	0.41	0.41
chr2	127786596	128946595	4	5	5	5	5	5	2	2	2	2	2	2	4.83	0.41	-2.04	0.41	0.41	0.41	0.41	0.41
chr2	128946596	128956595	4	5	5	5	5	5	2	2	2	2	2	2	4.83	0.41	-2.04	0.41	0.41	0.41	0.41	0.41
chr2	128956596	131196395	4	5	5	5	5	5	2	2	2	2	2	2	4.83	0.41	-2.04	0.41	0.41	0.41	0.41	0.41
chr2	131196396	131206395	4	5	5	5	5	5	2	2	2	2	2	2	4.83	0.41	-2.04	0.41	0.41	0.41	0.41	0.41
chr2	131206396	131276595	4	5	5	5	5	4	2	2	2	2	2	1	4.67	0.52	-1.29	0.65	0.65	0.65	0.65	-1.29
chr2	131276596	131286595	4	5	5	5	5	4	2	2	2	2	2	1	4.67	0.52	-1.29	0.65	0.65	0.65	0.65	-1.29
chr2	131286596	145577995	4	5	5	5	5	5	2	2	2	2	2	2	4.83	0.41	-2.04	0.41	0.41	0.41	0.41	0.41
chr2	145577996	145587995	4	5	5	5	5	5	2	2	2	2	2	2	4.83	0.41	-2.04	0.41	0.41	0.41	0.41	0.41
chr2	145587996	153968590	4	5	5	5	5	5	2	2	2	2	2	2	4.83	0.41	-2.04	0.41	0.41	0.41	0.41	0.41
chr2	153968591	153978590	4	5	5	5	5	5	2	2	2	2	2	2	4.83	0.41	-2.04	0.41	0.41	0.41	0.41	0.41
chr2	153978591	194970190	4	5	5	5	5	5	2	2	2	2	2	2	4.83	0.41	-2.04	0.41	0.41	0.41	0.41	0.41
chr2	194970191	194980190	4	5	5	5	5	5	2	2	2	2	2	2	4.83	0.41	-2.04	0.41	0.41	0.41	0.41	0.41
chr2	194980191	196060390	4	5	5	5	5	5	2	2	2	2	2	2	4.83	0.41	-2.04	0.41	0.41	0.41	0.41	0.41
chr2	196060391	196070390	4	5	5	5	5	5	2	2	2	2	2	2	4.83	0.41	-2.04	0.41	0.41	0.41	0.41	0.41
chr2	196070391	209250790	4	5	5	5	5	5	2	2	2	2	2	2	4.83	0.41	-2.04	0.41	0.41	0.41	0.41	0.41
chr2	209250791	209260790	4	5	5	5	5	5	2	2	2	2	2	2	4.83	0.41	-2.04	0.41	0.41	0.41	0.41	0.41
chr2	209260791	210830790	4	5	5	5	5	5	2	2	2	2	2	2	4.83	0.41	-2.04	0.41	0.41	0.41	0.41	0.41
chr2	210830791	210840790	4	5	5	5	5	5	2	2	2	2	2	2	4.83	0.41	-2.04	0.41	0.41	0.41	0.41	0.41
chr2	210840791	211590790	4	5	5	5	5	5	2	2	2	2	2	2	4.83	0.41	-2.04	0.41	0.41	0.41	0.41	0.41
chr2	211590791	211600790	4	5	5	5	5	5	2	2	2	2	2	2	4.83	0.41	-2.04	0.41	0.41	0.41	0.41	0.41
chr2	211600791	214960790	4	5	5	5	5	5	2	2	2	2	2	2	4.83	0.41	-2.04	0.41	0.41	0.41	0.41	0.41
chr2	214960791	214970790	4	5	5	5	5	5	2	2	2	2	2	2	4.83	0.41	-2.04	0.41	0.41	0.41	0.41	0.41
chr2	214970791	220620790	4	5	5	5	5	5	2	2	2	2	2	2	4.83	0.41	-2.04	0.41	0.41	0.41	0.41	0.41
chr2	220620791	220630790	4	5	5	5	5	5	2	2	2	2	2	2	4.83	0.41	-2.04	0.41	0.41	0.41	0.41	0.41
chr2	220630791	222870790	4	5	5	5	5	5	2	2	2	2	2	2	4.83	0.41	-2.04	0.41	0.41	0.41	0.41	0.41
chr2	222870791	222880790	4	5	5	5	5	5	2	2	2	2	2	2	4.83	0.41	-2.04	0.41	0.41	0.41	0.41	0.41
chr2	222880791	223700790	4	5	5	5	5	5	2	2	2	2	2	2	4.83	0.41	-2.04	0.41	0.41	0.41	0.41	0.41
chr2	223700791	223710790	4	5	5	5	5	5	2	2	2	2	2	2	4.83	0.41	-2.04	0.41	0.41	0.41	0.41	0.41
chr2	223710791	223821190	4	5	5	5	5	5	2	2	2	2	2	2	4.83	0.41	-2.04	0.41	0.41	0.41	0.41	0.41
chr2	223821191	223831190	4	5	5	5	5	5	2	2	2	2	2	2	4.83	0.41	-2.04	0.41	0.41	0.41	0.41	0.41
chr2	223831191	224601190	4	5	5	5	5	5	2	2	2	2	2	2	4.83	0.41	-2.04	0.41	0.41	0.41	0.41	0.41
chr2	224601191	224611190	4	5	5	5	5	5	2	2	2	2	2	2	4.83	0.41	-2.04	0.41	0.41	0.41	0.41	0.41
chr2	224611191	225501190	4	5	5	5	5	5	2	2	2	2	2	2	4.83	0.41	-2.04	0.41	0.41	0.41	0.41	0.41
chr2	225501191	225511190	4	5	5	5	5	5	2	2	2	2	2	2	4.83	0.41	-2.04	0.41	0.41	0.41	0.41	0.41
chr2	225511191	226261790	4	5	5	5	5	5	2	2	2	2	2	2	4.83	0.41	-2.04	0.41	0.41	0.41	0.41	0.41
chr2	226261791	226791790	4	5	5	4	5	5	2	2	2	2	2	2	4.67	0.52	-1.29	0.65	0.65	-1.29	0.65	0.65
chr2	226791791	226801790	4	5	5	4	5	5	2	2	2	2	2	2	4.67	0.52	-1.29	0.65	0.65	-1.29	0.65	0.65
chr2	226801791	226891190	4	5	5	5	5	5	2	2	2	2	2	2	4.83	0.41	-2.04	0.41	0.41	0.41	0.41	0.41
chr2	226891191	226901190	4	5	5	5	5	5	2	2	2	2	2	2	4.83	0.41	-2.04	0.41	0.41	0.41	0.41	0.41
chr2	226901191	243087375	4	5	5	5	5	5	2	2	2	2	2	2	4.83	0.41	-2.04	0.41	0.41	0.41	0.41	0.41
chr2	243087376	243097375	4	5	5	5	5	5	2	2	2	2	2	2	4.83	0.41	-2.04	0.41	0.41	0.41	0.41	0.41
chr2	243097376	243177570	4	5	5	5	5	5	2	2	2	2	2	2	4.83	0.41	-2.04	0.41	0.41	0.41	0.41	0.41
chr3	69801	4295000	3	4	4	4	4	4	1	2	2	2	2	2	3.83	0.41	-2.04	0.41	0.41	0.41	0.41	0.41
chr3	4295001	4305000	3	4	4	4	4	4	1	2	2	2	2	2	3.83	0.41	-2.04	0.41	0.41	0.41	0.41	0.41
chr3	4305001	5415000	3	4	4	4	4	4	1	2	2	2	2	2	3.83	0.41	-2.04	0.41	0.41	0.41	0.41	0.41
chr3	5415001	5425000	3	4	4	4	4	4	1	2	2	2	2	2	3.83	0.41	-2.04	0.41	0.41	0.41	0.41	0.41
chr3	5425001	8345000	3	4	4	4	4	4	1	2	2	2	2	2	3.83	0.41	-2.04	0.41	0.41	0.41	0.41	0.41
chr3	8345001	8355000	3	4	4	4	4	4	1	2	2	2	2	2	3.83	0.41	-2.04	0.41	0.41	0.41	0.41	0.41
chr3	8355001	16038000	3	4	4	4	4	4	1	2	2	2	2	2	3.83	0.41	-2.04	0.41	0.41	0.41	0.41	0.41
chr3	16038001	16048000	3	4	4	4	4	4	1	2	2	2	2	2	3.83	0.41	-2.04	0.41	0.41	0.41	0.41	0.41
chr3	16048001	16268000	3	4	4	4	4	4	1	2	2	2	2	2	3.83	0.41	-2.04	0.41	0.41	0.41	0.41	0.41
chr3	16268001	16278000	3	4	4	4	4	4	1	2	2	2	2	2	3.83	0.41	-2.04	0.41	0.41	0.41	0.41	0.41
chr3	16278001	17858000	3	4	4	4	4	4	1	2	2	2	2	2	3.83	0.41	-2.04	0.41	0.41	0.41	0.41	0.41

chr3	17858001	17868000	3	4	4	4	4	4	1	2	2	2	2	2	3.83	0.41	-2.04	0.41	0.41	0.41	0.41	0.41
chr3	17868001	18768000	3	4	4	4	4	4	1	2	2	2	2	2	3.83	0.41	-2.04	0.41	0.41	0.41	0.41	0.41
chr3	18768001	18778000	3	4	4	4	4	4	1	2	2	2	2	2	3.83	0.41	-2.04	0.41	0.41	0.41	0.41	0.41
chr3	18778001	18938000	3	4	4	4	4	4	1	2	2	2	2	2	3.83	0.41	-2.04	0.41	0.41	0.41	0.41	0.41
chr3	18938001	18948000	3	4	4	4	4	4	1	2	2	2	2	2	3.83	0.41	-2.04	0.41	0.41	0.41	0.41	0.41
chr3	18948001	75674400	3	4	4	4	4	4	1	2	2	2	2	2	3.83	0.41	-2.04	0.41	0.41	0.41	0.41	0.41
chr3	75674401	75684400	3	4	4	4	4	4	1	2	2	2	2	2	3.83	0.41	-2.04	0.41	0.41	0.41	0.41	0.41
chr3	75684401	75784400	4	5	5	5	5	5	2	2	0	0	1	1	4.83	0.41	-2.04	0.41	0.41	0.41	0.41	0.41
chr3	75784401	75794400	4	5	4	5	5	5	2	2	2	0	1	1	4.67	0.52	-1.29	0.65	-1.29	0.65	0.65	0.65
chr3	75794401	75815400	4	4	4	5	4	5	2	2	2	0	2	1	4.33	0.52	-0.65	-0.65	-0.65	1.29	-0.65	1.29
chr3	75815401	75825400	4	4	4	5	4	5	2	2	2	0	2	1	4.33	0.52	-0.65	-0.65	-0.65	1.29	-0.65	1.29
chr3	75825401	75846200	4	4	4	4	4	4	2	2	2	2	2	2	4	0	0	0	0	0	0	0
chr3	75846201	75856200	4	4	4	4	4	4	2	2	2	2	2	2	4	0	0	0	0	0	0	0
chr3	75856201	86386400	3	4	4	4	4	4	1	2	2	2	2	2	3.83	0.41	-2.04	0.41	0.41	0.41	0.41	0.41
chr3	86386401	86396400	3	4	4	4	4	4	1	2	2	2	2	2	3.83	0.41	-2.04	0.41	0.41	0.41	0.41	0.41
chr3	86396401	86436400	3	4	4	4	3	3	1	2	2	2	1	1	3.5	0.55	-0.91	0.91	0.91	0.91	-0.91	-0.91
chr3	86436401	86446400	3	4	4	4	3	3	1	2	2	2	1	1	3.5	0.55	-0.91	0.91	0.91	0.91	-0.91	-0.91
chr3	86446401	87886400	3	4	4	4	4	4	1	2	2	2	2	2	3.83	0.41	-2.04	0.41	0.41	0.41	0.41	0.41
chr3	87886401	93909995	3	4	4	4	4	4	1	2	2	2	2	2	3.83	0.41	-2.04	0.41	0.41	0.41	0.41	0.41
chr3	93909996	97899995	3	4	4	4	4	4	1	2	2	2	2	2	3.83	0.41	-2.04	0.41	0.41	0.41	0.41	0.41
chr3	97899996	98039995	3	4	4	3	4	4	1	2	2	1	2	2	3.67	0.52	-1.29	0.65	0.65	-1.29	0.65	0.65
chr3	98039996	98049995	3	4	4	3	4	4	1	2	2	1	2	2	3.67	0.52	-1.29	0.65	0.65	-1.29	0.65	0.65
chr3	98049996	169638995	3	4	4	4	4	4	1	2	2	2	2	2	3.83	0.41	-2.04	0.41	0.41	0.41	0.41	0.41
chr3	169638996	169648995	3	4	4	4	4	4	1	2	2	2	2	2	3.83	0.41	-2.04	0.41	0.41	0.41	0.41	0.41
chr3	169648996	172628995	3	4	4	4	4	4	1	2	2	2	2	2	3.83	0.41	-2.04	0.41	0.41	0.41	0.41	0.41
chr3	172628996	172638995	3	4	4	4	4	4	1	2	2	2	2	2	3.83	0.41	-2.04	0.41	0.41	0.41	0.41	0.41
chr3	172638996	179248995	3	4	4	4	4	4	1	2	2	2	2	2	3.83	0.41	-2.04	0.41	0.41	0.41	0.41	0.41
chr3	179248996	179258995	3	4	4	4	4	4	1	2	2	2	2	2	3.83	0.41	-2.04	0.41	0.41	0.41	0.41	0.41
chr3	179258996	179428995	3	4	4	4	4	4	1	2	2	2	2	2	3.83	0.41	-2.04	0.41	0.41	0.41	0.41	0.41
chr3	179428996	179438995	3	4	4	4	4	4	1	2	2	2	2	2	3.83	0.41	-2.04	0.41	0.41	0.41	0.41	0.41
chr3	179438996	182478995	3	4	4	4	4	4	1	2	2	2	2	2	3.83	0.41	-2.04	0.41	0.41	0.41	0.41	0.41
chr3	182478996	182488995	3	4	4	4	4	4	1	2	2	2	2	2	3.83	0.41	-2.04	0.41	0.41	0.41	0.41	0.41
chr3	182488996	188297795	3	4	4	4	4	4	1	2	2	2	2	2	3.83	0.41	-2.04	0.41	0.41	0.41	0.41	0.41
chr3	188297796	188407795	3	4	3	4	4	4	1	2	1	2	2	2	3.67	0.52	-1.29	0.65	-1.29	0.65	0.65	0.65
chr3	188407796	188417795	3	4	3	4	4	4	1	2	1	2	2	2	3.67	0.52	-1.29	0.65	-1.29	0.65	0.65	0.65
chr3	188417796	188948995	3	4	4	4	4	4	1	2	2	2	2	2	3.83	0.41	-2.04	0.41	0.41	0.41	0.41	0.41
chr3	188948996	188958995	3	4	4	4	4	4	1	2	2	2	2	2	3.83	0.41	-2.04	0.41	0.41	0.41	0.41	0.41
chr3	188958996	190128995	3	4	4	4	4	4	1	2	2	2	2	2	3.83	0.41	-2.04	0.41	0.41	0.41	0.41	0.41
chr3	190128996	190138995	3	4	4	4	4	4	1	2	2	2	2	2	3.83	0.41	-2.04	0.41	0.41	0.41	0.41	0.41
chr3	190138996	190388995	3	4	4	4	4	4	1	2	2	2	2	2	3.83	0.41	-2.04	0.41	0.41	0.41	0.41	0.41
chr3	190388996	190398995	3	4	4	4	4	4	1	2	2	2	2	2	3.83	0.41	-2.04	0.41	0.41	0.41	0.41	0.41
chr3	190398996	193328595	3	4	4	4	4	4	1	2	2	2	2	2	3.83	0.41	-2.04	0.41	0.41	0.41	0.41	0.41
chr3	193328596	193338595	3	4	4	4	4	4	1	2	2	2	2	2	3.83	0.41	-2.04	0.41	0.41	0.41	0.41	0.41
chr3	193338596	195375280	3	4	4	4	4	4	1	2	2	2	2	2	3.83	0.41	-2.04	0.41	0.41	0.41	0.41	0.41
chr3	195375281	195445280	4	4	4	4	4	4	2	2	2	2	2	2	4	0	0	0	0	0	0	0
chr3	195445281	195455280	4	4	4	4	4	4	2	2	2	2	2	2	4	0	0	0	0	0	0	0
chr3	195455281	197945480	3	4	4	4	4	4	1	2	2	2	2	2	3.83	0.41	-2.04	0.41	0.41	0.41	0.41	0.41
chr4	19801	4886095	4	4	4	4	4	4	2	2	2	2	2	2	4	0	0	0	0	0	0	0
chr4	4886096	4896095	4	4	4	4	4	4	2	2	2	2	2	2	4	0	0	0	0	0	0	0
chr4	4896096	6576095	4	4	4	4	4	4	2	2	2	2	2	2	4	0	0	0	0	0	0	0
chr4	6576096	6586095	4	4	4	4	4	4	2	2	2	2	2	2	4	0	0	0	0	0	0	0
chr4	6586096	7126095	4	4	4	4	4	4	2	2	2	2	2	2	4	0	0	0	0	0	0	0
chr4	7126096	7136095	4	4	4	4	4	4	2	2	2	2	2	2	4	0	0	0	0	0	0	0
chr4	7136096	9154890	4	4	4	4	4	4	2	2	2	2	2	2	4	0	0	0	0	0	0	0
chr4	9154891	9175690	4	4	4	4	4	4	2	2	2	2	2	2	4	0	0	0	0	0	0	0
chr4	9175691	9232490	3	4	2	3	3	2	1	2	0	1	0	0	2.83	0.75	0.22	1.55	-1.11	0.22	0.22	-1.11
chr4	9232491	9242490	3	4	2	3	3	2	1	2	0	1	0	0	2.83	0.75	0.22	1.55	-1.11	0.22	0.22	-1.11
chr4	9242491	9253690	4	4	4	3	4	4	2	2	2	1	2	2	3.83	0.41	0.41	0.41	0.41	-2.04	0.41	0.41
chr4	9253691	9915085	4	4	4	4	4	4	2	2	2	2	2	2	4	0	0	0	0	0	0	0
chr4	9915086	9925085	4	4	4	4	4	4	2	2	2	2	2	2	4	0	0	0	0	0	0	0
chr4	9925086	10155085	4	4	4	4	4	4	2	2	2	2	2	2	4	0	0	0	0	0	0	0
chr4	10155086	10165085	4	4	4	4	4	4	2	2	2	2	2	2	4	0	0	0	0	0	0	0
chr4	10165086	37218375	4	4	4	4	4	4	2	2	2	2	2	2	4	0	0	0	0	0	0	0
chr4	37218376	37228375	4	4	4	4	4	4	2	2	2	2	2	2	4	0	0	0	0	0	0	0
chr4	37228376	37548575	4	4	4	4	4	4	2	2	2	2	2	2	4	0	0	0	0	0	0	0
chr4	37548576	37558575	4	4	4	4	4	4	2	2	2	2	2	2	4	0	0	0	0	0	0	0
chr4	37558576	37607575	4	4	4	4	4	4	2	2	2	2	2	2	4	0	0	0	0	0	0	0
chr4	37607576	37617575	4	4	4	4	4	4	2	2	2	2	2	2	4	0	0	0	0	0	0	0
chr4	37617576	41198470	4	4	4	4	4	4	2	2	2	2	2	2	4	0	0	0	0	0	0	0
chr4	41198471	41208470	4	4	4	4	4	4	2	2	2	2	2	2	4	0	0	0	0	0	0	0
chr4	41208471	41499470	4	4	4	4	4	4	2	2	2	2	2	2	4	0	0	0	0	0	0	0
chr4	41499471	41509470	4	4	4	4	4	4	2	2	2	2	2	2	4	0	0	0	0	0	0	0
chr4	41509471	42679270	4	4	4	4	4	4	2	2	2	2	2	2	4	0	0	0	0	0	0	0
chr4	42679271	42689270	4	4	4	4	4	4	2	2	2	2	2	2	4	0	0	0	0	0	0	0
chr4	42689271	47429270	4	4	4	4	4	4	2	2	2	2	2	2	4	0	0	0	0	0	0	0
chr4	47429271	47439270	4	4	4	4	4	4	2	2	2	2	2	2	4	0	0	0	0	0	0	0

chr4	47439271	48188470	4	4	4	4	4	4	2	2	2	2	2	2	4	0	0	0	0	0	0	0
chr4	48188471	52709860	4	4	4	4	4	4	2	2	2	2	2	2	4	0	0	0	0	0	0	0
chr4	52709861	69170055	4	4	4	4	4	4	2	2	2	2	2	2	4	0	0	0	0	0	0	0
chr4	69170056	69240055	4	4	4	5	4	4	2	2	2	2	2	2	4.17	0.41	-0.41	-0.41	-0.41	2.04	-0.41	-0.41
chr4	69240056	69250055	4	4	4	5	4	4	2	2	2	2	2	2	4.17	0.41	-0.41	-0.41	-0.41	2.04	-0.41	-0.41
chr4	69250056	73840055	4	4	4	4	4	4	2	2	2	2	2	2	4	0	0	0	0	0	0	0
chr4	73840056	73850055	4	4	4	4	4	4	2	2	2	2	2	2	4	0	0	0	0	0	0	0
chr4	73850056	74240055	4	5	5	5	5	5	2	2	2	2	2	2	4.83	0.41	-2.04	0.41	0.41	0.41	0.41	0.41
chr4	74240056	74250055	4	5	5	5	5	5	2	2	2	2	2	2	4.83	0.41	-2.04	0.41	0.41	0.41	0.41	0.41
chr4	74250056	74260055	4	5	5	5	5	4	2	2	2	2	2	2	4.67	0.52	-1.29	0.65	0.65	0.65	0.65	-1.29
chr4	74260056	74270055	4	4	4	5	4	4	2	2	2	2	2	2	4.17	0.41	-0.41	-0.41	-0.41	2.04	-0.41	-0.41
chr4	74270056	80025350	4	4	4	4	4	4	2	2	2	2	2	2	4	0	0	0	0	0	0	0
chr4	80025351	80035350	4	4	4	4	4	4	2	2	2	2	2	2	4	0	0	0	0	0	0	0
chr4	80035351	80715350	4	4	4	4	4	4	2	2	2	2	2	2	4	0	0	0	0	0	0	0
chr4	80715351	80725350	4	4	4	4	4	4	2	2	2	2	2	2	4	0	0	0	0	0	0	0
chr4	80725351	81085350	4	4	4	4	4	4	2	2	2	2	2	2	4	0	0	0	0	0	0	0
chr4	81085351	81095350	4	4	4	4	4	4	2	2	2	2	2	2	4	0	0	0	0	0	0	0
chr4	81095351	82315350	4	4	4	4	4	4	2	2	2	2	2	2	4	0	0	0	0	0	0	0
chr4	82315351	82325350	4	4	4	4	4	4	2	2	2	2	2	2	4	0	0	0	0	0	0	0
chr4	82325351	82545350	4	4	4	4	4	4	2	2	2	2	2	2	4	0	0	0	0	0	0	0
chr4	82545351	82555350	4	4	4	4	4	4	2	2	2	2	2	2	4	0	0	0	0	0	0	0
chr4	82555351	87795550	4	4	4	4	4	4	2	2	2	2	2	2	4	0	0	0	0	0	0	0
chr4	87795551	88045550	4	4	4	4	4	4	2	2	2	2	2	2	4	0	0	0	0	0	0	0
chr4	88045551	88055550	4	4	4	4	4	4	2	2	2	2	2	2	4	0	0	0	0	0	0	0
chr4	88055551	95985150	4	4	4	4	4	4	2	2	2	2	2	2	4	0	0	0	0	0	0	0
chr4	95985151	95995150	4	4	4	4	4	4	2	2	2	2	2	2	4	0	0	0	0	0	0	0
chr4	95995151	106615750	2	2	2	2	2	2	0	0	0	0	0	0	2	0	0	0	0	0	0	0
chr4	106615751	106625750	2	2	2	2	2	2	0	0	0	0	0	0	2	0	0	0	0	0	0	0
chr4	106625751	106695750	0	0	0	0	0	0	0	0	0	0	0	0	0	0	0	0	0	0	0	0
chr4	106695751	106705750	0	0	0	0	0	0	0	0	0	0	0	0	0	0	0	0	0	0	0	0
chr4	106705751	114066950	2	2	2	2	2	2	0	0	0	0	0	0	2	0	0	0	0	0	0	0
chr4	114066951	114076950	2	2	2	2	2	2	0	0	0	0	0	0	2	0	0	0	0	0	0	0
chr4	114076951	114726950	2	2	2	2	2	2	0	0	0	0	0	0	2	0	0	0	0	0	0	0
chr4	114726951	114736950	2	2	2	2	2	2	0	0	0	0	0	0	2	0	0	0	0	0	0	0
chr4	114736951	119456950	2	2	2	2	2	2	0	0	0	0	0	0	2	0	0	0	0	0	0	0
chr4	119456951	119586950	2	2	2	2	2	2	0	0	0	0	0	1	2	0	0	0	0	0	0	0
chr4	119586951	119596950	2	2	2	2	2	2	0	0	0	0	0	1	2	0	0	0	0	0	0	0
chr4	119596951	132637150	2	2	2	2	2	2	0	0	0	0	0	0	2	0	0	0	0	0	0	0
chr4	132637151	132647150	2	2	2	2	2	2	0	0	0	0	0	0	2	0	0	0	0	0	0	0
chr4	132647151	132657350	2	1	2	0	1	1	0	0	0	0	0	0	1.17	0.75	1.11	-0.22	1.11	-1.55	-0.22	-0.22
chr4	132657351	132687150	1	1	1	0	1	1	0	0	0	0	0	0	0.83	0.41	0.41	0.41	0.41	-2.04	0.41	0.41
chr4	132687151	132697150	1	1	1	0	1	1	0	0	0	0	0	0	0.83	0.41	0.41	0.41	0.41	-2.04	0.41	0.41
chr4	132697151	132707350	1	2	1	2	2	2	0	0	0	0	0	0	1.67	0.52	-1.29	0.65	-1.29	0.65	0.65	0.65
chr4	132707351	165981550	2	2	2	2	2	2	0	0	0	0	0	0	2	0	0	0	0	0	0	0
chr4	165981551	165991550	2	2	2	2	2	2	0	0	0	0	0	0	2	0	0	0	0	0	0	0
chr4	165991551	166203350	2	2	2	3	2	2	0	0	0	0	0	0	2.17	0.41	-0.41	-0.41	-0.41	2.04	-0.41	-0.41
chr4	166203351	166283550	2	2	2	2	2	2	0	0	0	0	0	0	2	0	0	0	0	0	0	0
chr4	166283551	166293550	2	2	2	2	2	2	0	0	0	0	0	0	2	0	0	0	0	0	0	0
chr4	166293551	166362950	2	2	2	2	2	2	0	0	0	0	0	0	2	0	0	0	0	0	0	0
chr4	166362951	166372950	2	2	2	2	2	2	0	0	0	0	0	0	2	0	0	0	0	0	0	0
chr4	166372951	179425950	2	2	2	2	2	2	0	0	0	0	0	0	2	0	0	0	0	0	0	0
chr4	179425951	179436350	2	1	2	2	2	2	0	0	0	0	0	0	1.83	0.41	0.41	-2.04	0.41	0.41	0.41	0.41
chr4	179436351	182156350	2	1	1	2	1	2	0	0	0	0	0	0	1.5	0.55	0.91	-0.91	-0.91	0.91	-0.91	0.91
chr4	182156351	182166350	2	1	1	2	1	2	0	0	0	0	0	0	1.5	0.55	0.91	-0.91	-0.91	0.91	-0.91	0.91
chr4	182166351	182176750	2	2	1	2	2	2	0	0	0	0	0	0	1.83	0.41	0.41	0.41	-2.04	0.41	0.41	0.41
chr4	182176751	183626950	2	2	2	2	2	2	0	0	0	0	0	0	2	0	0	0	0	0	0	0
chr4	183626951	183636950	2	2	2	2	2	2	0	0	0	0	0	0	2	0	0	0	0	0	0	0
chr4	183636951	187799150	2	2	2	2	2	2	0	0	0	0	0	0	2	0	0	0	0	0	0	0
chr4	187799151	187809150	2	2	2	2	2	2	0	0	0	0	0	0	2	0	0	0	0	0	0	0
chr4	187809151	190530150	2	2	2	2	2	2	0	0	0	0	0	0	2	0	0	0	0	0	0	0
chr4	190530151	190540150	2	2	2	2	2	2	0	0	0	0	0	0	2	0	0	0	0	0	0	0
chr4	190540151	190679150	3	4	4	3	4	4	1	2	0	0	1	1	3.67	0.52	-1.29	0.65	0.65	-1.29	0.65	0.65
chr4	190679151	190689150	3	4	4	3	4	4	1	2	0	0	1	1	3.67	0.52	-1.29	0.65	0.65	-1.29	0.65	0.65
chr4	190689151	190929150	2	2	2	2	2	2	1	1	0	1	1	1	2	0	0	0	0	0	0	0
chr4	190929151	190939150	2	2	2	2	2	2	1	1	0	1	1	1	2	0	0	0	0	0	0	0
chr4	190939151	190949550	2	4	2	2	3	3	1	2	0	1	1	1	2.67	0.82	-0.82	1.63	-0.82	-0.82	0.41	0.41
chr4	190949551	190959550	2	4	2	4	3	3	1	2	0	1	1	1	3	0.89	-1.12	1.12	-1.12	1.12	0	0
chr4	190959551	191029150	2	4	3	4	3	3	1	2	1	1	1	1	3.17	0.75	-1.55	1.11	-0.22	1.11	-0.22	-0.22
chr5	19801	759800	4	5	5	4	5	4	2	2	2	1	2	1	4.5	0.55	-0.91	0.91	0.91	-0.91	0.91	-0.91
chr5	759801	849800	4	5	5	4	5	4	2	2	2	1	2	1	4.5	0.55	-0.91	0.91	0.91	-0.91	0.91	-0.91
chr5	849801	859800	4	5	5	4	5	4	2	2	2	1	2	1	4.5	0.55	-0.91	0.91	0.91	-0.91	0.91	-0.91
chr5	859801	1859800	4	5	5	4	5	4	2	2	2	1	2	1	4.5	0.55	-0.91	0.91	0.91	-0.91	0.91	-0.91
chr5	1859801	1869800	4	5	5	4	5	4	2	2	2	1	2	1	4.5	0.55	-0.91	0.91	0.91	-0.91	0.91	-0.91
chr5	1869801	7230000	4	5	5	4	5	4	2	2	2	1	2	1	4.5	0.55	-0.91	0.91	0.91	-0.91	0.91	-0.91
chr5	7230001	7240000	4	5	5	5	5	4	2	2	2	2	2	1	4.67	0.52	-1.29	0.65	0.65	0.65	0.65	-1.29
chr5	7240001	32091795	4	5	5	5	5	5	2	2	2	2	2	2	4.83	0.41	-2.04	0.41	0.41	0.41	0.41	0.41

chr5	32091796	32101795	4	5	5	5	5	5	2	2	2	2	2	2	4.83	0.41	-2.04	0.41	0.41	0.41	0.41	0.41
chr5	32101796	32441795	4	5	5	5	5	5	2	2	2	2	2	2	4.83	0.41	-2.04	0.41	0.41	0.41	0.41	0.41
chr5	32441796	32451795	4	5	5	5	5	5	2	2	2	2	2	2	4.83	0.41	-2.04	0.41	0.41	0.41	0.41	0.41
chr5	32451796	37112395	4	5	5	5	5	5	2	2	2	2	2	2	4.83	0.41	-2.04	0.41	0.41	0.41	0.41	0.41
chr5	37112396	37692395	4	5	5	5	5	5	2	2	2	2	2	2	4.83	0.41	-2.04	0.41	0.41	0.41	0.41	0.41
chr5	37692396	37702395	4	5	5	5	5	5	2	2	2	2	2	2	4.83	0.41	-2.04	0.41	0.41	0.41	0.41	0.41
chr5	37702396	42631995	4	5	5	5	5	5	2	2	2	2	2	2	4.83	0.41	-2.04	0.41	0.41	0.41	0.41	0.41
chr5	42631996	42641995	4	5	5	5	5	5	2	2	2	2	2	2	4.83	0.41	-2.04	0.41	0.41	0.41	0.41	0.41
chr5	42641996	42652195	4	5	5	5	5	5	2	2	2	2	2	2	4.83	0.41	-2.04	0.41	0.41	0.41	0.41	0.41
chr5	42652196	43671995	4	5	5	5	5	5	2	2	2	2	2	2	4.83	0.41	-2.04	0.41	0.41	0.41	0.41	0.41
chr5	43671996	43681995	4	5	5	5	5	5	2	2	2	2	2	2	4.83	0.41	-2.04	0.41	0.41	0.41	0.41	0.41
chr5	43681996	43702195	4	5	5	5	5	5	2	2	2	2	2	2	4.83	0.41	-2.04	0.41	0.41	0.41	0.41	0.41
chr5	43702196	43712195	4	5	5	5	5	5	2	2	2	2	2	2	4.83	0.41	-2.04	0.41	0.41	0.41	0.41	0.41
chr5	43712196	46082195	4	5	5	5	5	5	2	2	2	2	2	2	4.83	0.41	-2.04	0.41	0.41	0.41	0.41	0.41
chr5	46082196	50709990	4	5	5	5	5	5	2	2	2	2	2	2	4.83	0.41	-2.04	0.41	0.41	0.41	0.41	0.41
chr5	50709991	68311990	4	5	5	5	5	5	2	2	2	2	2	2	4.83	0.41	-2.04	0.41	0.41	0.41	0.41	0.41
chr5	68311991	68361990	4	5	5	6	5	5	2	2	2	2	2	2	5	0.63	-1.58	0	0	1.58	0	0
chr5	68361991	68801990	4	5	5	6	5	5	2	2	2	2	2	2	5	0.63	-1.58	0	0	1.58	0	0
chr5	68801991	68811990	4	5	5	6	5	5	2	2	2	2	2	2	5	0.63	-1.58	0	0	1.58	0	0
chr5	68811991	68852190	4	5	5	6	5	5	2	2	2	2	2	2	5	0.63	-1.58	0	0	1.58	0	0
chr5	68852191	70294390	4	5	5	6	5	5	2	2	2	2	2	2	5	0.63	-1.58	0	0	1.58	0	0
chr5	70294391	70304390	4	5	5	6	5	5	2	2	2	2	2	2	5	0.63	-1.58	0	0	1.58	0	0
chr5	70304391	70545990	4	5	5	6	5	5	2	2	2	2	2	2	5	0.63	-1.58	0	0	1.58	0	0
chr5	70545991	70555990	4	5	5	6	5	5	2	2	2	2	2	2	5	0.63	-1.58	0	0	1.58	0	0
chr5	70555991	70566390	4	5	5	6	5	5	2	2	2	2	2	2	5	0.63	-1.58	0	0	1.58	0	0
chr5	70566391	70636390	4	5	5	5	5	5	2	2	2	2	2	2	4.83	0.41	-2.04	0.41	0.41	0.41	0.41	0.41
chr5	70636391	70646390	4	5	5	5	5	5	2	2	2	2	2	2	4.83	0.41	-2.04	0.41	0.41	0.41	0.41	0.41
chr5	70646391	74554990	4	5	5	5	5	5	2	2	2	2	2	2	4.83	0.41	-2.04	0.41	0.41	0.41	0.41	0.41
chr5	74554991	74564990	4	5	5	5	5	5	2	2	2	2	2	2	4.83	0.41	-2.04	0.41	0.41	0.41	0.41	0.41
chr5	74564991	74796190	4	5	5	5	5	5	2	2	2	2	2	2	4.83	0.41	-2.04	0.41	0.41	0.41	0.41	0.41
chr5	74796191	74806190	4	5	5	5	5	5	2	2	2	2	2	2	4.83	0.41	-2.04	0.41	0.41	0.41	0.41	0.41
chr5	74806191	75024990	4	5	5	5	5	5	2	2	2	2	2	2	4.83	0.41	-2.04	0.41	0.41	0.41	0.41	0.41
chr5	75024991	75034990	4	5	5	5	5	5	2	2	2	2	2	2	4.83	0.41	-2.04	0.41	0.41	0.41	0.41	0.41
chr5	75034991	82494990	4	5	5	5	5	5	2	2	2	2	2	2	4.83	0.41	-2.04	0.41	0.41	0.41	0.41	0.41
chr5	82494991	82504990	4	5	5	5	5	5	2	2	2	2	2	2	4.83	0.41	-2.04	0.41	0.41	0.41	0.41	0.41
chr5	82504991	85121190	4	5	5	5	5	5	2	2	2	2	2	2	4.83	0.41	-2.04	0.41	0.41	0.41	0.41	0.41
chr5	85121191	85131190	4	5	5	5	5	5	2	2	2	2	2	2	4.83	0.41	-2.04	0.41	0.41	0.41	0.41	0.41
chr5	85131191	85781190	4	5	5	5	4	5	2	2	2	2	2	2	4.67	0.52	-1.29	0.65	0.65	0.65	-1.29	0.65
chr5	85781191	85791190	4	5	5	5	4	5	2	2	2	2	2	2	4.67	0.52	-1.29	0.65	0.65	0.65	-1.29	0.65
chr5	85791191	94823585	4	5	5	5	5	5	2	2	2	2	2	2	4.83	0.41	-2.04	0.41	0.41	0.41	0.41	0.41
chr5	94823586	94833585	4	5	5	5	5	5	2	2	2	2	2	2	4.83	0.41	-2.04	0.41	0.41	0.41	0.41	0.41
chr5	94833586	95074985	4	5	5	5	5	5	2	2	2	2	2	2	4.83	0.41	-2.04	0.41	0.41	0.41	0.41	0.41
chr5	95074986	95084985	4	5	5	5	5	5	2	2	2	2	2	2	4.83	0.41	-2.04	0.41	0.41	0.41	0.41	0.41
chr5	95084986	95314985	4	5	5	6	5	5	2	2	2	3	2	2	5	0.63	-1.58	0	0	1.58	0	0
chr5	95314986	95324985	4	5	5	6	5	5	2	2	2	3	2	2	5	0.63	-1.58	0	0	1.58	0	0
chr5	95324986	95673585	4	5	5	5	5	5	2	2	2	2	2	2	4.83	0.41	-2.04	0.41	0.41	0.41	0.41	0.41
chr5	95673586	95683585	4	5	5	5	5	5	2	2	2	2	2	2	4.83	0.41	-2.04	0.41	0.41	0.41	0.41	0.41
chr5	95683586	98033585	4	5	5	5	5	5	2	2	2	2	2	2	4.83	0.41	-2.04	0.41	0.41	0.41	0.41	0.41
chr5	98033586	98043585	4	5	5	5	5	5	2	2	2	2	2	2	4.83	0.41	-2.04	0.41	0.41	0.41	0.41	0.41
chr5	98043586	98405785	4	5	5	5	5	5	2	2	2	2	2	2	4.83	0.41	-2.04	0.41	0.41	0.41	0.41	0.41
chr5	98405786	98415785	4	5	5	5	5	5	2	2	2	2	2	2	4.83	0.41	-2.04	0.41	0.41	0.41	0.41	0.41
chr5	98415786	117826585	4	5	5	5	5	5	2	2	2	2	2	2	4.83	0.41	-2.04	0.41	0.41	0.41	0.41	0.41
chr5	117826586	117836585	4	5	5	5	5	5	2	2	2	2	2	2	4.83	0.41	-2.04	0.41	0.41	0.41	0.41	0.41
chr5	117836586	117976585	4	5	5	5	4	5	2	2	2	2	2	2	4.67	0.52	-1.29	0.65	0.65	0.65	-1.29	0.65
chr5	117976586	117986585	4	5	5	5	4	5	2	2	2	2	2	2	4.67	0.52	-1.29	0.65	0.65	0.65	-1.29	0.65
chr5	117986586	120099585	4	5	5	5	5	5	2	2	2	2	2	2	4.83	0.41	-2.04	0.41	0.41	0.41	0.41	0.41
chr5	120099586	120169585	3	5	5	5	5	5	1	2	2	2	2	2	4.67	0.82	-2.04	0.41	0.41	0.41	0.41	0.41
chr5	120169586	120179585	3	5	5	5	5	5	1	2	2	2	2	2	4.67	0.82	-2.04	0.41	0.41	0.41	0.41	0.41
chr5	120179586	124889585	4	5	5	5	5	5	2	2	2	2	2	2	4.83	0.41	-2.04	0.41	0.41	0.41	0.41	0.41
chr5	124889586	124899585	4	5	5	5	5	5	2	2	2	2	2	2	4.83	0.41	-2.04	0.41	0.41	0.41	0.41	0.41
chr5	124899586	125029585	4	5	5	5	4	5	2	2	2	2	2	2	4.67	0.52	-1.29	0.65	0.65	0.65	-1.29	0.65
chr5	125029586	125039585	4	5	5	5	4	5	2	2	2	2	2	2	4.67	0.52	-1.29	0.65	0.65	0.65	-1.29	0.65
chr5	125039586	127501385	4	5	5	5	5	5	2	2	2	2	2	2	4.83	0.41	-2.04	0.41	0.41	0.41	0.41	0.41
chr5	127501386	127511385	4	5	5	5	5	5	2	2	2	2	2	2	4.83	0.41	-2.04	0.41	0.41	0.41	0.41	0.41
chr5	127511386	130411385	4	5	5	5	5	5	2	2	2	2	2	2	4.83	0.41	-2.04	0.41	0.41	0.41	0.41	0.41
chr5	130411386	130421385	4	5	5	5	5	5	2	2	2	2	2	2	4.83	0.41	-2.04	0.41	0.41	0.41	0.41	0.41
chr5	130421386	148992980	4	5	5	5	5	5	2	2	2	2	2	2	4.83	0.41	-2.04	0.41	0.41	0.41	0.41	0.41
chr5	148992981	149002980	4	5	5	5	5	5	2	2	2	2	2	2	4.83	0.41	-2.04	0.41	0.41	0.41	0.41	0.41
chr5	149002981	149062980	4	5	5	5	4	5	2	2	2	2	2	2	4.67	0.52	-1.29	0.65	0.65	0.65	-1.29	0.65
chr5	149062981	149072980	4	5	5	5	4	5	2	2	2	2	2	2	4.67	0.52	-1.29	0.65	0.65	0.65	-1.29	0.65
chr5	149072981	170797975	4	5	5	5	5	5	2	2	2	2	2	2	4.83	0.41	-2.04	0.41	0.41	0.41	0.41	0.41
chr5	170797976	170807975	4	5	5	5	5	5	2	2	2	2	2	2	4.83	0.41	-2.04	0.41	0.41	0.41	0.41	0.41
chr5	170807976	170847975	5	5	5	5	5	5	2	2	2	2	2	2	5	0	0	0	0	0	0	0
chr5	170847976	170857975	5	5	5	5	5	5	2	2	2	2	2	2	5	0	0	0	0	0	0	0
chr5	170857976	176636575	4	5	5	5	5	5	2	2	2	2	2	2	4.83	0.41	-2.04	0.41	0.41	0.41	0.41	0.41
chr5	176636576	176646575	4	5	5	5	5	5	2	2	2	2	2	2	4.83	0.41	-2.04	0.41	0.41	0.41	0.41	0.41

chr5	176646576	176856575	4	5	5	5	6	5	2	2	2	2	2	2	5	0.63	-1.58	0	0	0	1.58	0
chr5	176856576	176866575	4	5	5	5	6	5	2	2	2	2	2	2	5	0.63	-1.58	0	0	0	1.58	0
chr5	176866576	178928375	4	5	5	5	5	5	2	2	2	2	2	2	4.83	0.41	-2.04	0.41	0.41	0.41	0.41	0.41
chr5	178928376	178938375	4	5	5	5	5	5	2	2	2	2	2	2	4.83	0.41	-2.04	0.41	0.41	0.41	0.41	0.41
chr5	178938376	179418375	4	5	5	5	5	5	2	2	2	2	2	2	4.83	0.41	-2.04	0.41	0.41	0.41	0.41	0.41
chr5	179418376	179428375	4	5	5	5	5	5	2	2	2	2	2	2	4.83	0.41	-2.04	0.41	0.41	0.41	0.41	0.41
chr5	179428376	180718375	4	5	5	5	5	5	2	2	2	2	2	2	4.83	0.41	-2.04	0.41	0.41	0.41	0.41	0.41
chr5	180718376	180728375	4	5	5	5	5	5	2	2	2	2	2	2	4.83	0.41	-2.04	0.41	0.41	0.41	0.41	0.41
chr5	180728376	180888375	4	4	5	5	5	4	2	2	2	2	2	2	4.5	0.55	-0.91	-0.91	0.91	0.91	0.91	-0.91
chr6	69801	301200	4	4	4	4	4	4	2	2	2	2	2	2	4	0	0	0	0	0	0	0
chr6	301201	371200	4	4	4	4	4	4	2	2	2	2	2	1	4	0	0	0	0	0	0	0
chr6	371201	381200	4	4	4	4	4	4	2	2	2	2	2	1	4	0	0	0	0	0	0	0
chr6	381201	2820800	4	4	4	4	4	4	2	2	2	2	2	2	4	0	0	0	0	0	0	0
chr6	2820801	2830800	4	4	4	4	4	4	2	2	2	2	2	2	4	0	0	0	0	0	0	0
chr6	2830801	3251200	4	5	5	5	5	5	2	2	2	2	2	2	4.83	0.41	-2.04	0.41	0.41	0.41	0.41	0.41
chr6	3251201	3261200	4	5	4	4	5	5	2	2	2	2	2	2	4.5	0.55	-0.91	0.91	-0.91	-0.91	0.91	0.91
chr6	3261201	3271200	4	5	4	4	5	5	2	2	2	2	2	2	4.5	0.55	-0.91	0.91	-0.91	-0.91	0.91	0.91
chr6	3271201	11090800	4	4	4	4	4	4	2	2	2	2	2	2	4	0	0	0	0	0	0	0
chr6	11090801	11100800	4	4	4	4	4	4	2	2	2	2	2	2	4	0	0	0	0	0	0	0
chr6	11100801	11560800	4	4	4	4	4	4	2	2	2	2	2	2	4	0	0	0	0	0	0	0
chr6	11560801	11570800	4	4	4	4	4	4	2	2	2	2	2	2	4	0	0	0	0	0	0	0
chr6	11570801	29622800	4	4	4	4	4	4	2	2	2	2	2	2	4	0	0	0	0	0	0	0
chr6	29622801	29642000	4	3	4	4	4	4	2	1	2	2	2	2	3.83	0.41	0.41	-2.04	0.41	0.41	0.41	0.41
chr6	29642001	29653000	4	3	4	4	4	4	2	1	2	2	2	2	3.83	0.41	0.41	-2.04	0.41	0.41	0.41	0.41
chr6	29653001	29695400	4	3	4	4	2	4	2	1	2	2	1	2	3.5	0.84	0.6	-0.6	0.6	0.6	-1.79	0.6
chr6	29695401	29708400	4	3	4	4	2	4	2	1	2	2	1	2	3.5	0.84	0.6	-0.6	0.6	0.6	-1.79	0.6
chr6	29708401	29736400	4	3	4	4	4	4	2	1	2	2	2	2	3.83	0.41	0.41	-2.04	0.41	0.41	0.41	0.41
chr6	29736401	29747000	4	3	4	4	4	4	2	1	2	2	2	2	3.83	0.41	0.41	-2.04	0.41	0.41	0.41	0.41
chr6	29747001	29817200	4	4	4	4	4	4	2	2	2	2	2	2	4	0	0	0	0	0	0	0
chr6	29817201	29926200	2	4	4	4	4	4	1	2	2	2	2	2	3.67	0.82	-2.04	0.41	0.41	0.41	0.41	0.41
chr6	29926201	29945000	2	4	4	4	4	4	1	2	2	2	2	2	3.67	0.82	-2.04	0.41	0.41	0.41	0.41	0.41
chr6	29945001	31002600	4	4	4	4	4	4	2	2	2	2	2	2	4	0	0	0	0	0	0	0
chr6	31002601	31012600	4	4	4	4	4	4	2	2	2	2	2	2	4	0	0	0	0	0	0	0
chr6	31012601	31035600	4	2	4	4	4	4	2	1	2	2	2	2	3.67	0.82	0.41	-2.04	0.41	0.41	0.41	0.41
chr6	31035601	31063400	4	2	4	4	4	4	2	1	2	2	1	2	3.67	0.82	0.41	-2.04	0.41	0.41	0.41	0.41
chr6	31063401	31075000	4	2	4	4	4	4	2	1	2	2	1	2	3.67	0.82	0.41	-2.04	0.41	0.41	0.41	0.41
chr6	31075001	31426000	4	4	4	4	4	4	2	2	2	2	1	2	4	0	0	0	0	0	0	0
chr6	31426001	31436000	4	4	4	4	4	4	2	2	2	2	1	2	4	0	0	0	0	0	0	0
chr6	31436001	31489600	4	4	0	4	4	4	2	2	0	2	1	2	3.33	1.63	0.41	0.41	-2.04	0.41	0.41	0.41
chr6	31489601	31501000	4	4	0	4	4	4	2	2	0	2	1	2	3.33	1.63	0.41	0.41	-2.04	0.41	0.41	0.41
chr6	31501001	47690600	4	4	4	4	4	4	2	2	2	2	2	2	4	0	0	0	0	0	0	0
chr6	47690601	47700600	4	4	4	4	4	4	2	2	2	2	2	2	4	0	0	0	0	0	0	0
chr6	47700601	48780600	4	4	4	4	4	4	2	2	2	2	2	2	4	0	0	0	0	0	0	0
chr6	48780601	48790600	4	4	4	4	4	4	2	2	2	2	2	2	4	0	0	0	0	0	0	0
chr6	48790601	48860600	4	4	4	4	3	4	2	2	2	2	1	2	3.83	0.41	0.41	0.41	0.41	0.41	-2.04	0.41
chr6	48860601	48870600	4	4	4	4	3	4	2	2	2	2	1	2	3.83	0.41	0.41	0.41	0.41	0.41	-2.04	0.41
chr6	48870601	58681995	4	4	4	4	4	4	2	2	2	2	2	2	4	0	0	0	0	0	0	0
chr6	58681996	63309985	4	4	4	4	4	4	2	2	2	2	2	2	4	0	0	0	0	0	0	0
chr6	63309986	63999985	4	4	4	4	4	4	2	2	2	2	2	2	4	0	0	0	0	0	0	0
chr6	63999986	64009985	4	4	4	4	4	4	2	2	2	2	2	2	4	0	0	0	0	0	0	0
chr6	64009986	65229985	4	4	4	4	4	4	2	2	2	2	2	2	4	0	0	0	0	0	0	0
chr6	65229986	65239985	4	4	4	4	4	4	2	2	2	2	2	2	4	0	0	0	0	0	0	0
chr6	65239986	73609985	4	4	4	4	4	4	2	2	2	2	2	2	4	0	0	0	0	0	0	0
chr6	73609986	73619985	4	4	4	4	4	4	2	2	2	2	2	2	4	0	0	0	0	0	0	0
chr6	73619986	74839385	4	4	4	4	4	4	2	2	2	2	2	2	4	0	0	0	0	0	0	0
chr6	74839386	74849385	4	4	4	4	4	4	2	2	2	2	2	2	4	0	0	0	0	0	0	0
chr6	74849386	79619385	4	4	4	4	4	4	2	2	2	2	2	2	4	0	0	0	0	0	0	0
chr6	79619386	79629385	4	4	4	4	4	4	2	2	2	2	2	2	4	0	0	0	0	0	0	0
chr6	79629386	79819385	4	4	4	4	4	4	2	2	2	2	2	2	4	0	0	0	0	0	0	0
chr6	79819386	79829385	4	4	4	4	4	4	2	2	2	2	2	2	4	0	0	0	0	0	0	0
chr6	79829386	86069585	4	4	4	4	4	4	2	2	2	2	2	2	4	0	0	0	0	0	0	0
chr6	86069586	86079585	4	4	4	4	4	4	2	2	2	2	2	2	4	0	0	0	0	0	0	0
chr6	86079586	86409585	4	4	4	4	4	4	2	2	2	2	2	2	4	0	0	0	0	0	0	0
chr6	86409586	86419585	4	4	4	4	4	4	2	2	2	2	2	2	4	0	0	0	0	0	0	0
chr6	86419586	86839585	4	4	4	4	4	4	2	2	2	2	2	2	4	0	0	0	0	0	0	0
chr6	86839586	86849585	4	4	4	4	4	4	2	2	2	2	2	2	4	0	0	0	0	0	0	0
chr6	86849586	86919585	4	4	4	4	4	4	2	2	2	2	2	2	4	0	0	0	0	0	0	0
chr6	86919586	86929585	4	4	4	4	4	4	2	2	2	2	2	2	4	0	0	0	0	0	0	0
chr6	86929586	170932165	4	4	4	4	4	4	2	2	2	2	2	2	4	0	0	0	0	0	0	0
chr6	170932166	171041165	4	4	4	4	4	4	2	2	2	2	2	1	4	0	0	0	0	0	0	0
chr7	19801	2909395	4	5	5	5	5	5	2	2	2	2	2	2	4.83	0.41	-2.04	0.41	0.41	0.41	0.41	0.41
chr7	2909396	2919395	4	5	5	5	5	5	2	2	2	2	2	2	4.83	0.41	-2.04	0.41	0.41	0.41	0.41	0.41
chr7	2919396	5029395	4	5	5	5	5	5	2	2	2	2	2	2	4.83	0.41	-2.04	0.41	0.41	0.41	0.41	0.41
chr7	5029396	5039395	4	5	5	5	5	5	2	2	2	2	2	2	4.83	0.41	-2.04	0.41	0.41	0.41	0.41	0.41
chr7	5039396	6759595	4	5	5	5	5	5	2	2	2	2	2	2	4.83	0.41	-2.04	0.41	0.41	0.41	0.41	0.41
chr7	6759596	6769595	4	5	5	5	5	5	2	2	2	2	2	2	4.83	0.41	-2.04	0.41	0.41	0.41	0.41	0.41

chr7	6769596	7969595	4	5	5	5	5	5	2	2	2	2	2	2	4.83	0.41	-2.04	0.41	0.41	0.41	0.41	0.41
chr7	7969596	7979595	4	5	5	5	5	5	2	2	2	2	2	2	4.83	0.41	-2.04	0.41	0.41	0.41	0.41	0.41
chr7	7979596	8249595	4	5	5	5	5	5	2	2	2	2	2	2	4.83	0.41	-2.04	0.41	0.41	0.41	0.41	0.41
chr7	8249596	8259595	4	5	5	5	5	5	2	2	2	2	2	2	4.83	0.41	-2.04	0.41	0.41	0.41	0.41	0.41
chr7	8259596	35669995	4	5	5	5	5	5	2	2	2	2	2	2	4.83	0.41	-2.04	0.41	0.41	0.41	0.41	0.41
chr7	35669996	35679995	4	5	5	5	5	5	2	2	2	2	2	2	4.83	0.41	-2.04	0.41	0.41	0.41	0.41	0.41
chr7	35679996	37530395	4	5	5	5	5	5	2	2	2	2	2	2	4.83	0.41	-2.04	0.41	0.41	0.41	0.41	0.41
chr7	37530396	37540395	4	5	5	5	5	5	2	2	2	2	2	2	4.83	0.41	-2.04	0.41	0.41	0.41	0.41	0.41
chr7	37540396	38290395	4	5	5	5	5	5	2	2	2	2	2	2	4.83	0.41	-2.04	0.41	0.41	0.41	0.41	0.41
chr7	38290396	38300395	5	5	5	5	5	5	2	2	2	2	2	2	5	0	0	0	0	0	0	0
chr7	38300396	38310795	5	5	7	5	6	5	2	2	3	2	3	2	5.5	0.84	-0.6	-0.6	1.79	-0.6	0.6	-0.6
chr7	38310796	38389995	5	6	7	5	6	6	2	3	3	2	3	2	5.83	0.75	-1.11	0.22	1.55	-1.11	0.22	0.22
chr7	38389996	38399995	5	6	7	5	6	6	2	3	3	2	3	2	5.83	0.75	-1.11	0.22	1.55	-1.11	0.22	0.22
chr7	38399996	57984990	4	5	5	5	5	5	2	2	2	2	2	2	4.83	0.41	-2.04	0.41	0.41	0.41	0.41	0.41
chr7	57984991	61737180	4	5	5	5	5	5	2	2	2	2	2	2	4.83	0.41	-2.04	0.41	0.41	0.41	0.41	0.41
chr7	61737181	62677375	4	5	5	5	5	5	2	2	2	2	2	2	4.83	0.41	-2.04	0.41	0.41	0.41	0.41	0.41
chr7	62677376	62687375	4	5	5	5	5	5	2	2	2	2	2	2	4.83	0.41	-2.04	0.41	0.41	0.41	0.41	0.41
chr7	62687376	62797375	4	5	5	5	4	5	2	2	2	2	2	2	4.67	0.52	-1.29	0.65	0.65	0.65	-1.29	0.65
chr7	62797376	62807375	4	5	5	5	4	5	2	2	2	2	2	2	4.67	0.52	-1.29	0.65	0.65	0.65	-1.29	0.65
chr7	62807376	63767375	4	5	5	5	5	5	2	2	2	2	2	2	4.83	0.41	-2.04	0.41	0.41	0.41	0.41	0.41
chr7	63767376	63777375	4	5	5	5	5	5	2	2	2	2	2	2	4.83	0.41	-2.04	0.41	0.41	0.41	0.41	0.41
chr7	63777376	67687375	4	5	5	5	5	5	2	2	2	2	2	2	4.83	0.41	-2.04	0.41	0.41	0.41	0.41	0.41
chr7	67687376	67697375	4	5	5	5	5	5	2	2	2	2	2	2	4.83	0.41	-2.04	0.41	0.41	0.41	0.41	0.41
chr7	67697376	72002175	4	5	5	5	5	5	2	2	2	2	2	2	4.83	0.41	-2.04	0.41	0.41	0.41	0.41	0.41
chr7	72002176	72012175	4	5	5	5	5	5	2	2	2	2	2	2	4.83	0.41	-2.04	0.41	0.41	0.41	0.41	0.41
chr7	72012176	77597570	4	5	5	5	5	5	2	2	2	2	2	2	4.83	0.41	-2.04	0.41	0.41	0.41	0.41	0.41
chr7	77597571	77607570	4	5	5	5	5	5	2	2	2	2	2	2	4.83	0.41	-2.04	0.41	0.41	0.41	0.41	0.41
chr7	77607571	82618170	4	5	5	5	5	5	2	2	2	2	2	2	4.83	0.41	-2.04	0.41	0.41	0.41	0.41	0.41
chr7	82618171	82628170	4	5	5	5	5	5	2	2	2	2	2	2	4.83	0.41	-2.04	0.41	0.41	0.41	0.41	0.41
chr7	82628171	82728170	4	5	5	5	4	5	2	2	2	2	2	2	4.67	0.52	-1.29	0.65	0.65	0.65	-1.29	0.65
chr7	82728171	82738170	4	5	5	5	4	5	2	2	2	2	2	2	4.67	0.52	-1.29	0.65	0.65	0.65	-1.29	0.65
chr7	82738171	114571365	4	5	5	5	5	5	2	2	2	2	2	2	4.83	0.41	-2.04	0.41	0.41	0.41	0.41	0.41
chr7	114571366	114671365	4	5	5	5	5	5	2	2	2	2	2	2	4.83	0.41	-2.04	0.41	0.41	0.41	0.41	0.41
chr7	114671366	114681365	4	5	5	5	5	5	2	2	2	2	2	2	4.83	0.41	-2.04	0.41	0.41	0.41	0.41	0.41
chr7	114681366	142337950	4	5	5	5	5	5	2	2	2	2	2	2	4.83	0.41	-2.04	0.41	0.41	0.41	0.41	0.41
chr7	142337951	142347950	4	5	5	5	5	5	2	2	2	2	2	2	4.83	0.41	-2.04	0.41	0.41	0.41	0.41	0.41
chr7	142347951	142447950	4	5	6	5	5	6	2	2	1	2	2	2	5.17	0.75	-1.55	-0.22	1.11	-0.22	-0.22	1.11
chr7	142447951	142457950	4	5	6	5	5	6	2	2	1	2	2	2	5.17	0.75	-1.55	-0.22	1.11	-0.22	-0.22	1.11
chr7	142457951	142489550	4	5	6	5	5	5	2	2	1	2	2	2	5	0.63	-1.58	0	1.58	0	0	0
chr7	142489551	142499550	4	5	6	5	5	5	2	2	1	2	2	2	5	0.63	-1.58	0	1.58	0	0	0
chr7	142499551	153153545	4	5	5	5	5	5	2	2	2	2	2	2	4.83	0.41	-2.04	0.41	0.41	0.41	0.41	0.41
chr7	153153546	153163545	4	5	5	5	5	5	2	2	2	2	2	2	4.83	0.41	-2.04	0.41	0.41	0.41	0.41	0.41
chr7	153163546	153233545	4	5	5	5	4	5	2	2	2	2	2	2	4.67	0.52	-1.29	0.65	0.65	0.65	-1.29	0.65
chr7	153233546	153243545	4	5	5	5	4	5	2	2	2	2	2	2	4.67	0.52	-1.29	0.65	0.65	0.65	-1.29	0.65
chr7	153243546	159110740	4	5	5	5	5	5	2	2	2	2	2	2	4.83	0.41	-2.04	0.41	0.41	0.41	0.41	0.41
chr8	19801	121200	4	5	5	5	4	5	2	2	2	0	2	2	4.67	0.52	-1.29	0.65	0.65	0.65	-1.29	0.65
chr8	121201	132000	4	5	3	5	4	3	2	2	0	0	2	0	4	0.89	0	1.12	-1.12	1.12	0	-1.12
chr8	132001	163800	4	3	3	3	3	3	2	1	0	0	0	0	3.17	0.41	2.04	-0.41	-0.41	-0.41	-0.41	-0.41
chr8	163801	734200	2	3	3	3	3	3	0	1	0	0	0	0	2.83	0.41	-2.04	0.41	0.41	0.41	0.41	0.41
chr8	734201	744200	2	3	3	3	3	3	0	1	0	0	0	0	2.83	0.41	-2.04	0.41	0.41	0.41	0.41	0.41
chr8	744201	5217600	2	3	3	3	3	3	0	1	0	0	0	0	2.83	0.41	-2.04	0.41	0.41	0.41	0.41	0.41
chr8	5217601	5227600	2	3	3	3	3	3	0	1	0	0	0	0	2.83	0.41	-2.04	0.41	0.41	0.41	0.41	0.41
chr8	5227601	5367600	4	5	5	5	5	5	2	2	2	2	2	2	4.83	0.41	-2.04	0.41	0.41	0.41	0.41	0.41
chr8	5367601	5377600	4	5	5	5	5	5	2	2	2	2	2	2	4.83	0.41	-2.04	0.41	0.41	0.41	0.41	0.41
chr8	5377601	5387800	2	3	5	3	3	3	0	1	2	0	0	0	3.17	0.98	-1.19	-0.17	1.86	-0.17	-0.17	-0.17
chr8	5387801	8030195	2	3	3	3	3	3	0	1	0	0	0	0	2.83	0.41	-2.04	0.41	0.41	0.41	0.41	0.41
chr8	8030196	8040195	2	3	3	3	3	3	0	1	0	0	0	0	2.83	0.41	-2.04	0.41	0.41	0.41	0.41	0.41
chr8	8040196	8080195	2	3	3	3	2	2	0	1	0	0	1	1	2.5	0.55	-0.91	0.91	0.91	0.91	-0.91	-0.91
chr8	8080196	8090195	2	3	3	3	2	2	0	1	0	0	1	1	2.5	0.55	-0.91	0.91	0.91	0.91	-0.91	-0.91
chr8	8090196	11540195	2	3	3	3	3	3	0	1	0	0	0	0	2.83	0.41	-2.04	0.41	0.41	0.41	0.41	0.41
chr8	11540196	11550195	2	3	3	3	3	3	0	1	0	0	0	0	2.83	0.41	-2.04	0.41	0.41	0.41	0.41	0.41
chr8	11550196	11690195	2	3	3	3	3	3	0	1	0	0	0	0	2.83	0.41	-2.04	0.41	0.41	0.41	0.41	0.41
chr8	11690196	11700195	2	3	3	3	3	3	0	1	0	0	0	0	2.83	0.41	-2.04	0.41	0.41	0.41	0.41	0.41
chr8	11700196	11920795	2	3	3	3	3	3	0	1	0	0	0	0	2.83	0.41	-2.04	0.41	0.41	0.41	0.41	0.41
chr8	11920796	11930795	2	3	3	3	3	3	0	1	0	0	0	0	2.83	0.41	-2.04	0.41	0.41	0.41	0.41	0.41
chr8	11930796	11942795	3	3	3	3	-	3	1	1	0	0	-	0	-	-	-	-	-	-	-	-
chr8	11942796	11962995	3	3	3	3	4	3	1	1	0	0	2	0	3.17	0.41	-0.41	-0.41	-0.41	-0.41	2.04	-0.41
chr8	11962996	11972995	3	4	3	3	4	3	1	2	0	0	2	0	3.33	0.52	-0.65	1.29	-0.65	-0.65	1.29	-0.65
chr8	11972996	12313390	3	4	4	4	4	4	1	2	1	1	2	2	3.83	0.41	-2.04	0.41	0.41	0.41	0.41	0.41
chr8	12313391	12323390	3	4	4	4	4	4	1	2	1	1	2	2	3.83	0.41	-2.04	0.41	0.41	0.41	0.41	0.41
chr8	12323391	12354990	3	4	3	3	4	4	1	2	0	0	2	2	3.5	0.55	-0.91	0.91	-0.91	-0.91	0.91	0.91
chr8	12354991	12364990	3	4	3	3	4	4	1	2	0	0	2	2	3.5	0.55	-0.91	0.91	-0.91	-0.91	0.91	0.91
chr8	12364991	12384390	3	3	3	3	4	3	1	1	0	0	2	1	3.17	0.41	-0.41	-0.41	-0.41	-0.41	2.04	-0.41
chr8	12384391	12403390	2	3	3	3	4	3	0	1	0	0	2	1	3	0.63	-1.58	0	0	0	1.58	0
chr8	12403391	12486190	2	3	3	3	3	3	0	1	0	0	1	1	2.83	0.41	-2.04	0.41	0.41	0.41	0.41	0.41
chr8	12486191	12496190	2	3	3	3	3	3	0	1	0	0	1	1	2.83	0.41	-2.04	0.41	0.41	0.41	0.41	0.41

chr8	12496191	12844590	2	3	3	3	3	3	0	1	0	0	0	0	2.83	0.41	-2.04	0.41	0.41	0.41	0.41	0.41
chr8	12844591	12854590	2	3	3	3	3	3	0	1	0	0	0	0	2.83	0.41	-2.04	0.41	0.41	0.41	0.41	0.41
chr8	12854591	13324590	2	3	3	3	3	3	0	1	0	0	0	0	2.83	0.41	-2.04	0.41	0.41	0.41	0.41	0.41
chr8	13324591	13334590	2	3	3	3	3	3	0	1	0	0	0	0	2.83	0.41	-2.04	0.41	0.41	0.41	0.41	0.41
chr8	13334591	16934590	2	3	3	3	3	3	0	1	0	0	0	0	2.83	0.41	-2.04	0.41	0.41	0.41	0.41	0.41
chr8	16934591	16944590	2	3	3	3	3	3	0	1	0	0	0	0	2.83	0.41	-2.04	0.41	0.41	0.41	0.41	0.41
chr8	16944591	18054590	2	3	3	3	3	3	0	1	0	0	0	0	2.83	0.41	-2.04	0.41	0.41	0.41	0.41	0.41
chr8	18054591	18064590	2	3	3	3	3	3	0	1	0	0	0	0	2.83	0.41	-2.04	0.41	0.41	0.41	0.41	0.41
chr8	18064591	36159190	2	3	3	3	3	3	0	1	0	0	0	0	2.83	0.41	-2.04	0.41	0.41	0.41	0.41	0.41
chr8	36159191	36169190	2	3	3	3	3	3	0	1	0	0	0	0	2.83	0.41	-2.04	0.41	0.41	0.41	0.41	0.41
chr8	36169191	43082390	3	3	3	3	3	3	1	1	0	0	0	0	3	0	0	0	0	0	0	0
chr8	43082391	48109985	3	3	3	3	3	3	1	1	0	0	0	0	3	0	0	0	0	0	0	0
chr8	48109986	49045480	3	3	3	3	3	3	1	1	0	0	0	0	3	0	0	0	0	0	0	0
chr8	49045481	49155280	3	3	3	3	3	3	1	1	0	0	0	0	3	0	0	0	0	0	0	0
chr8	49155281	49475480	3	3	3	3	3	3	1	1	0	0	0	0	3	0	0	0	0	0	0	0
chr8	49475481	49485480	3	3	3	3	3	3	1	1	0	0	0	0	3	0	0	0	0	0	0	0
chr8	49485481	74145480	3	3	3	3	3	3	1	1	0	0	0	0	3	0	0	0	0	0	0	0
chr8	74145481	74155480	3	3	3	3	3	3	1	1	0	0	0	0	3	0	0	0	0	0	0	0
chr8	74155481	74905480	3	3	3	3	3	3	1	1	0	0	0	0	3	0	0	0	0	0	0	0
chr8	74905481	74915480	3	3	3	3	3	3	1	1	0	0	0	0	3	0	0	0	0	0	0	0
chr8	74915481	86005480	3	3	3	3	3	3	1	1	0	0	0	0	3	0	0	0	0	0	0	0
chr8	86005481	86015480	3	3	3	3	3	3	1	1	0	0	0	0	3	0	0	0	0	0	0	0
chr8	86015481	86175480	3	3	3	3	3	3	1	1	0	0	0	1	3	0	0	0	0	0	0	0
chr8	86175481	86185480	3	3	3	3	3	3	1	1	0	0	0	1	3	0	0	0	0	0	0	0
chr8	86185481	87236075	3	3	3	3	3	3	1	1	0	0	0	0	3	0	0	0	0	0	0	0
chr8	87236076	87246075	3	3	3	3	3	3	1	1	0	0	0	0	3	0	0	0	0	0	0	0
chr8	87246076	87596075	3	3	3	3	3	3	1	1	0	0	0	0	3	0	0	0	0	0	0	0
chr8	87596076	87606075	3	3	3	3	3	3	1	1	0	0	0	0	3	0	0	0	0	0	0	0
chr8	87606076	118541075	3	3	3	3	3	3	1	1	0	0	0	0	3	0	0	0	0	0	0	0
chr8	118541076	118551075	3	3	3	4	3	3	1	1	0	0	0	0	3.17	0.41	-0.41	-0.41	-0.41	2.04	-0.41	-0.41
chr8	118551076	118561075	4	3	5	4	3	4	2	1	0	0	0	0	3.83	0.75	0.22	-1.11	1.55	0.22	-1.11	0.22
chr8	118561076	146282270	4	4	5	4	4	4	2	1	0	0	0	0	4.17	0.41	-0.41	-0.41	2.04	-0.41	-0.41	-0.41
chr9	19801	39800	3	4	5	5	4	5	1	2	2	2	2	2	4.33	0.82	-1.63	-0.41	0.82	0.82	-0.41	0.82
chr9	39801	49800	3	4	5	5	4	5	1	2	2	2	2	2	4.33	0.82	-1.63	-0.41	0.82	0.82	-0.41	0.82
chr9	49801	59800	3	4	5	5	5	5	1	2	2	2	2	2	4.5	0.84	-1.79	-0.6	0.6	0.6	0.6	0.6
chr9	59801	519800	3	5	5	5	5	5	1	2	2	2	2	2	4.67	0.82	-2.04	0.41	0.41	0.41	0.41	0.41
chr9	519801	1109800	3	5	5	5	5	5	1	2	2	2	2	2	4.67	0.82	-2.04	0.41	0.41	0.41	0.41	0.41
chr9	1109801	1119800	3	5	5	5	5	5	1	2	2	2	2	2	4.67	0.82	-2.04	0.41	0.41	0.41	0.41	0.41
chr9	1119801	1749800	3	5	5	5	5	5	1	2	2	2	2	2	4.67	0.82	-2.04	0.41	0.41	0.41	0.41	0.41
chr9	1749801	1759800	3	5	5	5	5	5	1	2	2	2	2	2	4.67	0.82	-2.04	0.41	0.41	0.41	0.41	0.41
chr9	1759801	1969800	3	5	5	5	5	5	1	2	2	2	2	2	4.67	0.82	-2.04	0.41	0.41	0.41	0.41	0.41
chr9	1969801	1979800	3	5	5	5	5	5	1	2	2	2	2	2	4.67	0.82	-2.04	0.41	0.41	0.41	0.41	0.41
chr9	1979801	19026200	3	5	5	5	5	5	1	2	2	2	2	2	4.67	0.82	-2.04	0.41	0.41	0.41	0.41	0.41
chr9	19026201	19036200	3	5	5	5	5	5	1	2	2	2	2	2	4.67	0.82	-2.04	0.41	0.41	0.41	0.41	0.41
chr9	19036201	19056400	3	5	5	5	5	5	1	2	2	2	2	2	4.67	0.82	-2.04	0.41	0.41	0.41	0.41	0.41
chr9	19056401	19066400	3	5	5	5	5	5	1	2	2	2	2	2	4.67	0.82	-2.04	0.41	0.41	0.41	0.41	0.41
chr9	19066401	19356200	3	5	5	5	5	5	1	2	2	2	2	2	4.67	0.82	-2.04	0.41	0.41	0.41	0.41	0.41
chr9	19356201	19366200	3	5	5	5	5	5	1	2	2	2	2	2	4.67	0.82	-2.04	0.41	0.41	0.41	0.41	0.41
chr9	19366201	19466400	3	5	5	5	5	5	1	2	2	2	2	2	4.67	0.82	-2.04	0.41	0.41	0.41	0.41	0.41
chr9	19466401	19476400	3	5	5	5	5	5	1	2	2	2	2	2	4.67	0.82	-2.04	0.41	0.41	0.41	0.41	0.41
chr9	19476401	25006200	3	5	5	5	5	5	1	2	2	2	2	2	4.67	0.82	-2.04	0.41	0.41	0.41	0.41	0.41
chr9	25006201	25016200	3	5	5	5	5	5	1	2	2	2	2	2	4.67	0.82	-2.04	0.41	0.41	0.41	0.41	0.41
chr9	25016201	25176200	3	5	5	5	4	5	1	2	2	2	1	2	4.5	0.84	-1.79	0.6	0.6	0.6	-0.6	0.6
chr9	25176201	25186200	3	5	5	5	4	5	1	2	2	2	1	2	4.5	0.84	-1.79	0.6	0.6	0.6	-0.6	0.6
chr9	25186201	28649800	3	5	5	5	5	5	1	2	2	2	2	2	4.67	0.82	-2.04	0.41	0.41	0.41	0.41	0.41
chr9	28649801	28659800	3	5	4	5	5	5	1	2	2	2	2	2	4.5	0.84	-1.79	0.6	-0.6	0.6	0.6	0.6
chr9	28659801	28670000	3	5	4	5	4	5	1	2	2	2	2	2	4.33	0.82	-1.63	0.82	-0.41	0.82	-0.41	0.82
chr9	28670001	28680000	2	5	4	5	4	5	1	2	2	2	2	2	4.17	1.17	-1.85	0.71	-0.14	0.71	-0.14	0.71
chr9	28680001	28709400	2	3	4	5	4	4	1	1	2	2	2	2	3.67	1.03	-1.61	-0.65	0.32	1.29	0.32	0.32
chr9	28709401	28719400	2	3	4	5	4	4	1	1	2	2	2	2	3.67	1.03	-1.61	-0.65	0.32	1.29	0.32	0.32
chr9	28719401	28729600	2	3	5	5	5	4	1	1	2	2	2	2	4	1.26	-1.58	-0.79	0.79	0.79	0.79	0
chr9	28729601	32349600	3	5	5	5	5	5	1	2	2	2	2	2	4.67	0.82	-2.04	0.41	0.41	0.41	0.41	0.41
chr9	32349601	32359600	3	5	5	5	5	5	1	2	2	2	2	2	4.67	0.82	-2.04	0.41	0.41	0.41	0.41	0.41
chr9	32359601	32389800	3	5	5	5	5	5	1	2	2	2	2	2	4.67	0.82	-2.04	0.41	0.41	0.41	0.41	0.41
chr9	32389801	32399800	3	5	5	5	5	5	1	2	2	2	2	2	4.67	0.82	-2.04	0.41	0.41	0.41	0.41	0.41
chr9	32399801	38480800	3	5	5	5	5	5	1	2	2	2	2	2	4.67	0.82	-2.04	0.41	0.41	0.41	0.41	0.41
chr9	38480801	38490800	3	5	5	5	5	5	1	2	2	2	2	2	4.67	0.82	-2.04	0.41	0.41	0.41	0.41	0.41
chr9	38490801	40062590	3	5	5	5	5	5	1	2	2	2	2	2	4.67	0.82	-2.04	0.41	0.41	0.41	0.41	0.41
chr9	40062591	40072790	3	5	5	5	5	5	1	2	2	2	2	2	4.67	0.82	-2.04	0.41	0.41	0.41	0.41	0.41
chr9	40072791	40353585	3	5	5	5	5	5	1	2	2	2	2	2	4.67	0.82	-2.04	0.41	0.41	0.41	0.41	0.41
chr9	40353586	40363585	3	5	5	5	5	5	1	2	2	2	2	2	4.67	0.82	-2.04	0.41	0.41	0.41	0.41	0.41
chr9	40363586	40483780	2	5	5	5	5	5	1	2	2	2	2	2	4.5	1.22	-2.04	0.41	0.41	0.41	0.41	0.41
chr9	40483781	40493780	2	5	5	5	5	5	1	2	2	2	2	2	4.5	1.22	-2.04	0.41	0.41	0.41	0.41	0.41
chr9	40493781	41335770	3	5	5	5	5	5	1	2	2	2	2	2	4.67	0.82	-2.04	0.41	0.41	0.41	0.41	0.41
chr9	41335771	41345770	3	5	5	5	5	5	1	2	2	2	2	2	4.67	0.82	-2.04	0.41	0.41	0.41	0.41	0.41
chr9	41345771	41456165	4	5	5	5	5	5	2	2	2	2	2	2	4.83	0.41	-2.04	0.41	0.41	0.41	0.41	0.41

chr9	41456166	41466365	4	5	5	5	5	5	2	2	2	2	2	2	4.83	0.41	-2.04	0.41	0.41	0.41	0.41	0.41
chr9	41466366	43130765	3	5	5	5	5	5	1	2	2	2	2	2	4.67	0.82	-2.04	0.41	0.41	0.41	0.41	0.41
chr9	43130766	43140765	3	5	5	5	5	5	1	2	2	2	2	2	4.67	0.82	-2.04	0.41	0.41	0.41	0.41	0.41
chr9	43140766	43150765	3	5	5	5	5	5	1	2	2	2	2	2	4.67	0.82	-2.04	0.41	0.41	0.41	0.41	0.41
chr9	43150766	43160765	3	5	5	5	5	5	1	2	2	2	2	2	4.67	0.82	-2.04	0.41	0.41	0.41	0.41	0.41
chr9	43160766	43202765	3	5	5	5	5	5	1	2	2	2	2	2	4.67	0.82	-2.04	0.41	0.41	0.41	0.41	0.41
chr9	43202766	43212765	3	5	5	5	5	5	1	2	2	2	2	2	4.67	0.82	-2.04	0.41	0.41	0.41	0.41	0.41
chr9	43212766	44294155	3	5	5	5	5	5	1	2	2	2	2	2	4.67	0.82	-2.04	0.41	0.41	0.41	0.41	0.41
chr9	44294156	44304155	3	5	5	5	5	5	1	2	2	2	2	2	4.67	0.82	-2.04	0.41	0.41	0.41	0.41	0.41
chr9	44304156	44585355	3	5	5	5	5	5	1	2	2	2	2	2	4.67	0.82	-2.04	0.41	0.41	0.41	0.41	0.41
chr9	44585356	44595355	3	5	5	5	5	5	1	2	2	2	2	2	4.67	0.82	-2.04	0.41	0.41	0.41	0.41	0.41
chr9	44595356	44674155	3	5	5	5	5	6	1	2	2	2	2	2	4.83	0.98	-1.86	0.17	0.17	0.17	0.17	1.19
chr9	44674156	44734350	3	5	5	4	5	6	1	2	2	1	2	2	4.67	1.03	-1.61	0.32	0.32	-0.65	0.32	1.29
chr9	44734351	44844350	2	3	5	4	4	4	1	1	2	1	2	2	3.67	1.03	-1.61	-0.65	1.29	0.32	0.32	0.32
chr9	44844351	44854350	2	3	5	4	4	4	1	1	2	1	2	2	3.67	1.03	-1.61	-0.65	1.29	0.32	0.32	0.32
chr9	44854351	44865550	3	5	5	4	5	4	1	2	2	1	2	2	4.33	0.82	-1.63	0.82	0.82	-0.41	0.82	-0.41
chr9	44865551	45026145	3	5	5	4	5	5	1	2	2	1	2	2	4.5	0.84	-1.79	0.6	0.6	-0.6	0.6	0.6
chr9	45026146	45036145	3	5	5	4	5	5	1	2	2	1	2	2	4.5	0.84	-1.79	0.6	0.6	-0.6	0.6	0.6
chr9	45036146	45165745	3	5	5	5	5	5	1	2	2	1	2	2	4.67	0.82	-2.04	0.41	0.41	0.41	0.41	0.41
chr9	45165746	45175745	3	5	5	5	5	5	1	2	2	1	2	2	4.67	0.82	-2.04	0.41	0.41	0.41	0.41	0.41
chr9	45175746	47286525	3	5	5	5	5	5	1	2	2	1	2	2	4.67	0.82	-2.04	0.41	0.41	0.41	0.41	0.41
chr9	47286526	65478320	3	5	5	5	5	5	1	2	2	1	2	2	4.67	0.82	-2.04	0.41	0.41	0.41	0.41	0.41
chr9	65478321	65618920	3	6	5	5	5	5	1	3	2	2	1	1	4.83	0.98	-1.86	1.19	0.17	0.17	0.17	0.17
chr9	65618921	65628920	3	6	5	5	5	5	1	3	2	2	1	1	4.83	0.98	-1.86	1.19	0.17	0.17	0.17	0.17
chr9	65628921	65649120	3	5	5	5	5	5	1	2	2	2	1	1	4.67	0.82	-2.04	0.41	0.41	0.41	0.41	0.41
chr9	65649121	65659120	3	5	5	5	5	5	1	2	2	2	1	1	4.67	0.82	-2.04	0.41	0.41	0.41	0.41	0.41
chr9	65659121	86177670	3	5	5	5	5	5	1	2	2	2	2	2	4.67	0.82	-2.04	0.41	0.41	0.41	0.41	0.41
chr9	86177671	86187670	3	5	5	5	5	5	1	2	2	2	2	2	4.67	0.82	-2.04	0.41	0.41	0.41	0.41	0.41
chr9	86187671	86617670	3	5	5	5	5	5	1	2	2	2	2	2	4.67	0.82	-2.04	0.41	0.41	0.41	0.41	0.41
chr9	86617671	86627670	3	5	5	5	5	5	1	2	2	2	2	2	4.67	0.82	-2.04	0.41	0.41	0.41	0.41	0.41
chr9	86627671	91818870	3	5	5	5	5	5	1	2	2	2	2	2	4.67	0.82	-2.04	0.41	0.41	0.41	0.41	0.41
chr9	91818871	91828870	3	5	5	5	5	5	1	2	2	2	2	2	4.67	0.82	-2.04	0.41	0.41	0.41	0.41	0.41
chr9	91828871	92959260	3	5	5	5	5	5	1	2	2	2	2	2	4.67	0.82	-2.04	0.41	0.41	0.41	0.41	0.41
chr9	92959261	92969260	3	5	5	5	5	5	1	2	2	2	2	2	4.67	0.82	-2.04	0.41	0.41	0.41	0.41	0.41
chr9	92969261	93529260	3	5	5	5	5	5	1	2	2	2	2	2	4.67	0.82	-2.04	0.41	0.41	0.41	0.41	0.41
chr9	93529261	93539260	3	5	5	5	5	5	1	2	2	2	2	2	4.67	0.82	-2.04	0.41	0.41	0.41	0.41	0.41
chr9	93539261	97009260	3	5	5	5	5	5	1	2	2	2	2	2	4.67	0.82	-2.04	0.41	0.41	0.41	0.41	0.41
chr9	97009261	97019260	3	5	5	5	5	5	1	2	2	2	2	2	4.67	0.82	-2.04	0.41	0.41	0.41	0.41	0.41
chr9	97019261	97109260	3	5	5	5	4	5	1	2	2	2	2	2	4.5	0.84	-1.79	0.6	0.6	0.6	-0.6	0.6
chr9	97109261	97119260	3	5	5	5	4	5	1	2	2	2	2	2	4.5	0.84	-1.79	0.6	0.6	0.6	-0.6	0.6
chr9	97119261	104079260	3	5	5	5	5	5	1	2	2	2	2	2	4.67	0.82	-2.04	0.41	0.41	0.41	0.41	0.41
chr9	104079261	104089260	3	5	5	5	5	5	1	2	2	2	2	2	4.67	0.82	-2.04	0.41	0.41	0.41	0.41	0.41
chr9	104089261	104199260	3	5	5	5	5	5	1	2	2	2	2	2	4.67	0.82	-2.04	0.41	0.41	0.41	0.41	0.41
chr9	104199261	104209260	3	5	5	5	5	5	1	2	2	2	2	2	4.67	0.82	-2.04	0.41	0.41	0.41	0.41	0.41
chr9	104209261	107949260	3	5	5	5	5	5	1	2	2	2	2	2	4.67	0.82	-2.04	0.41	0.41	0.41	0.41	0.41
chr9	107949261	107959260	3	5	5	5	5	5	1	2	2	2	2	2	4.67	0.82	-2.04	0.41	0.41	0.41	0.41	0.41
chr9	107959261	108529260	3	5	5	5	5	5	1	2	2	2	2	2	4.67	0.82	-2.04	0.41	0.41	0.41	0.41	0.41
chr9	108529261	108539260	3	5	5	5	5	5	1	2	2	2	2	2	4.67	0.82	-2.04	0.41	0.41	0.41	0.41	0.41
chr9	108539261	111508860	3	5	5	5	5	5	1	2	2	2	2	2	4.67	0.82	-2.04	0.41	0.41	0.41	0.41	0.41
chr9	111508861	111518860	3	5	5	5	5	5	1	2	2	2	2	2	4.67	0.82	-2.04	0.41	0.41	0.41	0.41	0.41
chr9	111518861	111529260	3	6	5	6	5	6	1	3	2	3	2	3	5.17	1.17	-1.85	0.71	-0.14	0.71	-0.14	0.71
chr9	111529261	112139660	3	6	6	6	6	6	1	3	3	3	3	3	5.5	1.22	-2.04	0.41	0.41	0.41	0.41	0.41
chr9	112139661	112149660	3	6	5	6	6	6	1	3	2	3	3	3	5.33	1.21	-1.93	0.55	-0.28	0.55	0.55	0.55
chr9	112149661	112159860	3	5	5	6	5	5	1	2	2	3	2	2	4.83	0.98	-1.86	0.17	0.17	1.19	0.17	0.17
chr9	112159861	113419260	3	5	5	5	5	5	1	2	2	2	2	2	4.67	0.82	-2.04	0.41	0.41	0.41	0.41	0.41
chr9	113419261	113429260	3	5	5	5	5	5	1	2	2	2	2	2	4.67	0.82	-2.04	0.41	0.41	0.41	0.41	0.41
chr9	113429261	113469260	3	5	5	5	4	5	1	2	2	2	2	2	4.5	0.84	-1.79	0.6	0.6	0.6	-0.6	0.6
chr9	113469261	113479260	3	5	5	5	4	5	1	2	2	2	2	2	4.5	0.84	-1.79	0.6	0.6	0.6	-0.6	0.6
chr9	113479261	114079260	3	5	5	5	5	5	1	2	2	2	2	2	4.67	0.82	-2.04	0.41	0.41	0.41	0.41	0.41
chr9	114079261	114089260	3	5	5	5	5	5	1	2	2	2	2	2	4.67	0.82	-2.04	0.41	0.41	0.41	0.41	0.41
chr9	114089261	117669460	3	5	5	5	5	5	1	2	2	2	2	2	4.67	0.82	-2.04	0.41	0.41	0.41	0.41	0.41
chr9	117669461	117679460	3	5	5	5	5	5	1	2	2	2	2	2	4.67	0.82	-2.04	0.41	0.41	0.41	0.41	0.41
chr9	117679461	120109460	3	5	5	5	5	5	1	2	2	2	2	2	4.67	0.82	-2.04	0.41	0.41	0.41	0.41	0.41
chr9	120109461	120119460	3	5	5	5	5	5	1	2	2	2	2	2	4.67	0.82	-2.04	0.41	0.41	0.41	0.41	0.41
chr9	120119461	120459460	3	5	5	5	5	5	1	2	2	2	2	2	4.67	0.82	-2.04	0.41	0.41	0.41	0.41	0.41
chr9	120459461	120469460	3	5	5	5	5	5	1	2	2	2	2	2	4.67	0.82	-2.04	0.41	0.41	0.41	0.41	0.41
chr9	120469461	123149460	3	5	5	5	5	5	1	2	2	2	2	2	4.67	0.82	-2.04	0.41	0.41	0.41	0.41	0.41
chr9	123149461	123159460	3	5	5	5	5	5	1	2	2	2	2	2	4.67	0.82	-2.04	0.41	0.41	0.41	0.41	0.41
chr9	123159461	141134850	3	5	5	5	5	5	1	2	2	2	2	2	4.67	0.82	-2.04	0.41	0.41	0.41	0.41	0.41
chr10	69801	850000	3	5	5	5	5	5	1	2	2	2	2	2	4.67	0.82	-2.04	0.41	0.41	0.41	0.41	0.41
chr10	850001	860000	3	5	5	5	5	5	1	2	2	2	2	2	4.67	0.82	-2.04	0.41	0.41	0.41	0.41	0.41
chr10	860001	1210000	3	5	5	5	6	5	1	2	2	2	2	2	4.83	0.98	-1.86	0.17	0.17	0.17	1.19	0.17
chr10	1210001	1220000	3	5	5	5	6	5	1	2	2	2	2	2	4.83	0.98	-1.86	0.17	0.17	0.17	1.19	0.17
chr10	1220001	5163600	3	5	5	5	5	5	1	2	2	2	2	2	4.67	0.82	-2.04	0.41	0.41	0.41	0.41	0.41
chr10	5163601	5173600	3	5	5	5	5	5	1	2	2	2	2	2	4.67	0.82	-2.04	0.41	0.41	0.41	0.41	0.41
chr10	5173601	6343600	3	5	5	5	5	5	1	2	2	2	2	2	4.67	0.82	-2.04	0.41	0.41	0.41	0.41	0.41

chr10	6343601	6353600	3	5	5	5	5	5	1	2	2	2	2	2	4.67	0.82	-2.04	0.41	0.41	0.41	0.41	0.41
chr10	6353601	7593600	3	5	5	5	5	5	1	2	2	2	2	2	4.67	0.82	-2.04	0.41	0.41	0.41	0.41	0.41
chr10	7593601	7603600	3	5	5	5	5	5	1	2	2	2	2	2	4.67	0.82	-2.04	0.41	0.41	0.41	0.41	0.41
chr10	7603601	8023600	3	5	5	5	5	5	1	2	2	2	2	2	4.67	0.82	-2.04	0.41	0.41	0.41	0.41	0.41
chr10	8023601	8033600	3	5	5	5	5	5	1	2	2	2	2	2	4.67	0.82	-2.04	0.41	0.41	0.41	0.41	0.41
chr10	8033601	11433600	3	5	5	5	5	5	1	2	2	2	2	2	4.67	0.82	-2.04	0.41	0.41	0.41	0.41	0.41
chr10	11433601	11443600	3	5	5	5	5	5	1	2	2	2	2	2	4.67	0.82	-2.04	0.41	0.41	0.41	0.41	0.41
chr10	11443601	12253600	3	5	5	5	5	5	1	2	2	2	2	2	4.67	0.82	-2.04	0.41	0.41	0.41	0.41	0.41
chr10	12253601	12263600	3	5	5	5	5	5	1	2	2	2	2	2	4.67	0.82	-2.04	0.41	0.41	0.41	0.41	0.41
chr10	12263601	19344000	3	5	5	5	5	5	1	2	2	2	2	2	4.67	0.82	-2.04	0.41	0.41	0.41	0.41	0.41
chr10	19344001	19354000	3	5	5	5	5	5	1	2	2	2	2	2	4.67	0.82	-2.04	0.41	0.41	0.41	0.41	0.41
chr10	19354001	20044000	3	5	5	5	5	5	1	2	2	2	2	2	4.67	0.82	-2.04	0.41	0.41	0.41	0.41	0.41
chr10	20044001	20054000	3	5	5	5	5	5	1	2	2	2	2	2	4.67	0.82	-2.04	0.41	0.41	0.41	0.41	0.41
chr10	20054001	37744200	3	5	5	5	5	5	1	2	2	2	2	2	4.67	0.82	-2.04	0.41	0.41	0.41	0.41	0.41
chr10	37744201	37754200	3	5	5	5	5	5	1	2	2	2	2	2	4.67	0.82	-2.04	0.41	0.41	0.41	0.41	0.41
chr10	37754201	37804200	3	5	5	5	4	5	1	2	2	2	2	2	4.5	0.84	-1.79	0.6	0.6	0.6	-0.6	0.6
chr10	37804201	37814200	3	5	5	5	4	5	1	2	2	2	2	2	4.5	0.84	-1.79	0.6	0.6	0.6	-0.6	0.6
chr10	37814201	37984200	3	5	5	5	5	5	1	2	2	2	2	2	4.67	0.82	-2.04	0.41	0.41	0.41	0.41	0.41
chr10	37984201	42365000	3	5	5	5	5	5	1	2	2	2	2	2	4.67	0.82	-2.04	0.41	0.41	0.41	0.41	0.41
chr10	42365001	46836590	3	5	5	5	5	5	1	2	2	2	2	2	4.67	0.82	-2.04	0.41	0.41	0.41	0.41	0.41
chr10	46836591	46890590	3	4	5	5	5	5	1	2	2	2	2	2	4.5	0.84	-1.79	-0.6	0.6	0.6	0.6	0.6
chr10	46890591	46900590	3	4	5	5	5	5	1	2	2	2	2	2	4.5	0.84	-1.79	-0.6	0.6	0.6	0.6	0.6
chr10	46900591	47189790	3	5	5	5	5	5	1	2	2	2	2	2	4.67	0.82	-2.04	0.41	0.41	0.41	0.41	0.41
chr10	47189791	47199790	3	5	5	5	5	6	1	2	2	2	2	2	4.83	0.98	-1.86	0.17	0.17	0.17	0.17	1.19
chr10	47199791	47240790	3	5	5	5	6	6	1	2	2	2	2	2	5	1.1	-1.83	0	0	0	0.91	0.91
chr10	47240791	47250790	3	5	5	5	6	6	1	2	2	2	2	2	5	1.1	-1.83	0	0	0	0.91	0.91
chr10	47250791	47329590	3	6	5	5	6	6	1	2	2	2	2	2	5.17	1.17	-1.85	0.71	-0.14	-0.14	0.71	0.71
chr10	47329591	47339590	3	6	5	5	6	6	1	2	2	2	2	2	5.17	1.17	-1.85	0.71	-0.14	-0.14	0.71	0.71
chr10	47339591	72751770	3	5	5	5	5	5	1	2	2	2	2	2	4.67	0.82	-2.04	0.41	0.41	0.41	0.41	0.41
chr10	72751771	72761770	3	5	5	5	5	5	1	2	2	2	2	2	4.67	0.82	-2.04	0.41	0.41	0.41	0.41	0.41
chr10	72761771	72841770	3	5	5	5	5	5	1	2	2	2	2	2	4.67	0.82	-2.04	0.41	0.41	0.41	0.41	0.41
chr10	72841771	72851770	3	5	5	5	5	5	1	2	2	2	2	2	4.67	0.82	-2.04	0.41	0.41	0.41	0.41	0.41
chr10	72851771	78321770	3	5	5	5	5	5	1	2	2	2	2	2	4.67	0.82	-2.04	0.41	0.41	0.41	0.41	0.41
chr10	78321771	78331770	3	5	5	5	5	5	1	2	2	2	2	2	4.67	0.82	-2.04	0.41	0.41	0.41	0.41	0.41
chr10	78331771	78701770	3	5	5	5	5	5	1	2	2	2	2	2	4.67	0.82	-2.04	0.41	0.41	0.41	0.41	0.41
chr10	78701771	78711770	3	5	5	5	5	5	1	2	2	2	2	2	4.67	0.82	-2.04	0.41	0.41	0.41	0.41	0.41
chr10	78711771	82631770	3	5	5	5	5	5	1	2	2	2	2	2	4.67	0.82	-2.04	0.41	0.41	0.41	0.41	0.41
chr10	82631771	82641770	3	5	5	5	5	5	1	2	2	2	2	2	4.67	0.82	-2.04	0.41	0.41	0.41	0.41	0.41
chr10	82641771	87821770	3	5	5	5	5	5	1	2	2	2	2	2	4.67	0.82	-2.04	0.41	0.41	0.41	0.41	0.41
chr10	87821771	87831770	3	5	5	5	5	5	1	2	2	2	2	2	4.67	0.82	-2.04	0.41	0.41	0.41	0.41	0.41
chr10	87831771	101746770	3	5	5	5	5	5	1	2	2	2	2	2	4.67	0.82	-2.04	0.41	0.41	0.41	0.41	0.41
chr10	101746771	101756770	3	5	5	5	5	5	1	2	2	2	2	2	4.67	0.82	-2.04	0.41	0.41	0.41	0.41	0.41
chr10	101756771	102196770	3	5	5	5	5	5	1	2	2	2	2	2	4.67	0.82	-2.04	0.41	0.41	0.41	0.41	0.41
chr10	102196771	102206770	3	5	5	5	5	5	1	2	2	2	2	2	4.67	0.82	-2.04	0.41	0.41	0.41	0.41	0.41
chr10	102206771	106214170	3	5	5	5	5	5	1	2	2	2	2	2	4.67	0.82	-2.04	0.41	0.41	0.41	0.41	0.41
chr10	106214171	106224170	3	5	5	5	5	5	1	2	2	2	2	2	4.67	0.82	-2.04	0.41	0.41	0.41	0.41	0.41
chr10	106224171	109786570	3	5	5	5	5	5	1	2	2	2	2	2	4.67	0.82	-2.04	0.41	0.41	0.41	0.41	0.41
chr10	109786571	109796570	3	5	5	5	5	5	1	2	2	2	2	2	4.67	0.82	-2.04	0.41	0.41	0.41	0.41	0.41
chr10	109796571	109886570	3	5	5	5	4	5	1	2	2	2	2	2	4.5	0.84	-1.79	0.6	0.6	0.6	-0.6	0.6
chr10	109886571	109896570	3	5	5	5	4	5	1	2	2	2	2	2	4.5	0.84	-1.79	0.6	0.6	0.6	-0.6	0.6
chr10	109896571	126338965	3	5	5	5	5	5	1	2	2	2	2	2	4.67	0.82	-2.04	0.41	0.41	0.41	0.41	0.41
chr10	126338966	126348965	3	5	5	5	5	5	1	2	2	2	2	2	4.67	0.82	-2.04	0.41	0.41	0.41	0.41	0.41
chr10	126348966	126678965	3	5	5	5	5	5	1	2	2	2	2	2	4.67	0.82	-2.04	0.41	0.41	0.41	0.41	0.41
chr10	126678966	126688965	3	5	5	5	5	5	1	2	2	2	2	2	4.67	0.82	-2.04	0.41	0.41	0.41	0.41	0.41
chr10	126688966	132119160	3	5	5	5	5	5	1	2	2	2	2	2	4.67	0.82	-2.04	0.41	0.41	0.41	0.41	0.41
chr10	132119161	132129160	3	5	5	5	5	5	1	2	2	2	2	2	4.67	0.82	-2.04	0.41	0.41	0.41	0.41	0.41
chr10	132129161	132949160	3	5	5	5	5	5	1	2	2	2	2	2	4.67	0.82	-2.04	0.41	0.41	0.41	0.41	0.41
chr10	132949161	132959160	3	5	5	5	5	5	1	2	2	2	2	2	4.67	0.82	-2.04	0.41	0.41	0.41	0.41	0.41
chr10	132959161	135510550	3	5	5	5	5	5	1	2	2	2	2	2	4.67	0.82	-2.04	0.41	0.41	0.41	0.41	0.41
chr11	69801	139800	4	6	5	5	5	5	2	2	1	2	1	2	5	0.63	-1.58	1.58	0	0	0	0
chr11	139801	149800	4	6	5	5	5	5	2	2	1	2	1	2	5	0.63	-1.58	1.58	0	0	0	0
chr11	149801	169800	4	6	5	6	6	5	2	2	1	2	2	2	5.33	0.82	-1.63	0.82	-0.41	0.82	0.82	-0.41
chr11	169801	199800	4	6	6	6	6	6	2	2	1	2	2	2	5.67	0.82	-2.04	0.41	0.41	0.41	0.41	0.41
chr11	199801	1720595	3	6	6	6	6	6	1	2	1	2	2	2	5.5	1.22	-2.04	0.41	0.41	0.41	0.41	0.41
chr11	1720596	1730595	3	7	6	6	6	6	1	3	1	2	2	2	5.67	1.37	-1.95	0.98	0.24	0.24	0.24	0.24
chr11	1730596	1740795	3	7	8	6	8	8	1	3	3	2	3	3	6.67	1.97	-1.86	0.17	0.68	-0.34	0.68	0.68
chr11	1740796	2170395	3	7	8	8	8	8	1	3	3	3	3	3	7	2	-2	0	0.5	0.5	0.5	0.5
chr11	2170396	2180395	3	7	8	8	8	8	1	3	3	3	3	3	7	2	-2	0	0.5	0.5	0.5	0.5
chr11	2180396	2190595	3	6	8	6	8	6	1	2	3	2	3	2	6.17	1.83	-1.73	-0.09	1	-0.09	1	-0.09
chr11	2190596	4231395	3	6	6	6	6	6	1	2	2	2	2	2	5.5	1.22	-2.04	0.41	0.41	0.41	0.41	0.41
chr11	4231396	4241395	3	6	6	6	6	6	1	2	2	2	2	2	5.5	1.22	-2.04	0.41	0.41	0.41	0.41	0.41
chr11	4241396	4262195	1	4	6	4	4	4	0	1	2	0	1	1	3.83	1.6	-1.77	0.1	1.35	0.1	0.1	0.1
chr11	4262196	4273395	1	4	6	4	4	4	0	1	2	0	1	1	3.83	1.6	-1.77	0.1	1.35	0.1	0.1	0.1
chr11	4273396	4314795	1	4	4	4	4	4	0	1	0	0	1	1	3.5	1.22	-2.04	0.41	0.41	0.41	0.41	0.41
chr11	4314796	4324795	1	4	4	4	4	4	0	1	0	0	1	1	3.5	1.22	-2.04	0.41	0.41	0.41	0.41	0.41
chr11	4324796	4335795	1	4	6	4	4	6	0	1	2	0	1	2	4.17	1.83	-1.73	-0.09	1	-0.09	-0.09	1

chr11	4335796	6466995	3	5	6	6	6	6	0	2	2	2	2	2	5.33	1.21	-1.93	-0.28	0.55	0.55	0.55	0.55
chr11	6466996	6476995	3	5	6	6	6	6	0	2	2	2	2	2	5.33	1.21	-1.93	-0.28	0.55	0.55	0.55	0.55
chr11	6476996	6716995	3	5	6	6	6	6	0	2	2	2	2	2	5.33	1.21	-1.93	-0.28	0.55	0.55	0.55	0.55
chr11	6716996	6726995	3	5	6	6	6	6	0	2	2	2	2	2	5.33	1.21	-1.93	-0.28	0.55	0.55	0.55	0.55
chr11	6726996	18748995	3	5	6	6	6	6	0	2	2	2	2	2	5.33	1.21	-1.93	-0.28	0.55	0.55	0.55	0.55
chr11	18748996	18758995	3	5	6	6	6	6	0	2	2	2	2	2	5.33	1.21	-1.93	-0.28	0.55	0.55	0.55	0.55
chr11	18758996	31797595	2	5	6	6	6	6	0	2	2	2	2	2	5.17	1.6	-1.98	-0.1	0.52	0.52	0.52	0.52
chr11	31797596	31807595	2	5	6	6	6	6	0	2	2	2	2	2	5.17	1.6	-1.98	-0.1	0.52	0.52	0.52	0.52
chr11	31807596	45777195	3	5	6	6	6	6	1	2	2	2	2	2	5.33	1.21	-1.93	-0.28	0.55	0.55	0.55	0.55
chr11	45777196	45787195	3	5	6	6	6	6	1	2	2	2	2	2	5.33	1.21	-1.93	-0.28	0.55	0.55	0.55	0.55
chr11	45787196	48190595	3	5	6	6	6	6	1	2	2	2	2	2	5.33	1.21	-1.93	-0.28	0.55	0.55	0.55	0.55
chr11	48190596	48200595	3	5	6	6	6	6	1	2	2	2	2	2	5.33	1.21	-1.93	-0.28	0.55	0.55	0.55	0.55
chr11	48200596	48791995	3	5	6	6	6	6	1	2	2	1	2	2	5.33	1.21	-1.93	-0.28	0.55	0.55	0.55	0.55
chr11	48791996	48801995	3	5	6	6	6	6	1	2	2	1	2	2	5.33	1.21	-1.93	-0.28	0.55	0.55	0.55	0.55
chr11	48801996	48961995	3	5	6	6	6	5	1	2	2	1	2	1	5.17	1.17	-1.85	-0.14	0.71	0.71	0.71	-0.14
chr11	48961996	48971995	3	5	6	6	6	5	1	2	2	1	2	1	5.17	1.17	-1.85	-0.14	0.71	0.71	0.71	-0.14
chr11	48971996	50633995	3	5	6	6	6	6	1	2	2	1	2	2	5.33	1.21	-1.93	-0.28	0.55	0.55	0.55	0.55
chr11	50633996	50644395	3	5	6	6	6	6	1	2	2	1	2	2	5.33	1.21	-1.93	-0.28	0.55	0.55	0.55	0.55
chr11	50644396	50672395	3	5	6	5	6	6	1	2	2	0	2	2	5.17	1.17	-1.85	-0.14	0.71	-0.14	0.71	0.71
chr11	50672396	50682395	3	5	6	5	6	6	1	2	2	0	2	2	5.17	1.17	-1.85	-0.14	0.71	-0.14	0.71	0.71
chr11	50682396	50772395	3	5	6	5	6	5	1	2	2	0	2	1	5	1.1	-1.83	0	0.91	0	0.91	0
chr11	50772396	50782395	3	5	6	5	6	5	1	2	2	0	2	1	5	1.1	-1.83	0	0.91	0	0.91	0
chr11	50782396	51091590	3	5	6	5	6	6	1	2	2	0	2	2	5.17	1.17	-1.85	-0.14	0.71	-0.14	0.71	0.71
chr11	51091591	51101590	3	5	6	5	6	6	1	2	2	0	2	2	5.17	1.17	-1.85	-0.14	0.71	-0.14	0.71	0.71
chr11	51101591	51578790	3	5	6	6	6	6	1	2	2	2	2	2	5.33	1.21	-1.93	-0.28	0.55	0.55	0.55	0.55
chr11	51578791	55709990	3	5	6	6	6	6	1	2	2	2	2	2	5.33	1.21	-1.93	-0.28	0.55	0.55	0.55	0.55
chr11	55709991	56879990	3	6	6	6	6	6	1	2	2	2	2	2	5.5	1.22	-2.04	0.41	0.41	0.41	0.41	0.41
chr11	56879991	56889990	3	6	6	6	6	6	1	2	2	2	2	2	5.5	1.22	-2.04	0.41	0.41	0.41	0.41	0.41
chr11	56889991	57599990	3	6	6	6	6	6	1	2	2	2	2	2	5.5	1.22	-2.04	0.41	0.41	0.41	0.41	0.41
chr11	57599991	57609990	3	6	6	6	6	6	1	2	2	2	2	2	5.5	1.22	-2.04	0.41	0.41	0.41	0.41	0.41
chr11	57609991	61129990	3	6	6	6	6	6	1	2	2	2	2	2	5.5	1.22	-2.04	0.41	0.41	0.41	0.41	0.41
chr11	61129991	61139990	3	6	6	6	6	6	1	2	2	2	2	2	5.5	1.22	-2.04	0.41	0.41	0.41	0.41	0.41
chr11	61139991	67420990	3	6	6	6	6	6	1	2	2	2	2	2	5.5	1.22	-2.04	0.41	0.41	0.41	0.41	0.41
chr11	67420991	67430990	3	6	6	6	6	6	1	2	2	2	2	2	5.5	1.22	-2.04	0.41	0.41	0.41	0.41	0.41
chr11	67430991	67580990	3	6	6	6	6	6	1	2	2	2	2	2	5.5	1.22	-2.04	0.41	0.41	0.41	0.41	0.41
chr11	67580991	67590990	3	6	6	6	6	6	1	2	2	2	2	2	5.5	1.22	-2.04	0.41	0.41	0.41	0.41	0.41
chr11	67590991	68901190	3	6	6	6	6	6	1	2	2	2	2	2	5.5	1.22	-2.04	0.41	0.41	0.41	0.41	0.41
chr11	68901191	68911190	3	6	6	6	6	6	1	2	2	2	2	2	5.5	1.22	-2.04	0.41	0.41	0.41	0.41	0.41
chr11	68911191	68921390	3	6	6	6	7	6	1	2	2	2	3	2	5.67	1.37	-1.95	0.24	0.24	0.24	0.98	0.24
chr11	68921391	68931390	3	6	7	6	7	6	1	3	3	2	3	2	5.83	1.47	-1.92	0.11	0.79	0.11	0.79	0.11
chr11	68931391	68941590	3	6	7	6	7	7	1	3	3	2	3	3	6	1.55	-1.94	0	0.65	0	0.65	0.65
chr11	68941591	69491785	3	6	7	7	7	7	1	3	3	3	3	3	6.17	1.6	-1.98	-0.1	0.52	0.52	0.52	0.52
chr11	69491786	69501785	3	6	7	7	7	7	1	3	3	3	3	3	6.17	1.6	-1.98	-0.1	0.52	0.52	0.52	0.52
chr11	69501786	69521385	3	6	6	6	7	7	1	3	2	2	3	3	5.83	1.47	-1.92	0.11	0.11	0.11	0.79	0.79
chr11	69521386	69531385	3	6	6	6	7	7	1	3	2	2	3	3	5.83	1.47	-1.92	0.11	0.11	0.11	0.79	0.79
chr11	69531386	72921585	3	6	6	6	6	6	1	2	2	2	2	2	5.5	1.22	-2.04	0.41	0.41	0.41	0.41	0.41
chr11	72921586	72931585	3	6	6	6	6	6	1	2	2	2	2	2	5.5	1.22	-2.04	0.41	0.41	0.41	0.41	0.41
chr11	72931586	72971585	3	6	6	6	5	6	1	2	2	2	2	2	5.33	1.21	-1.93	0.55	0.55	0.55	-0.28	0.55
chr11	72971586	72981585	3	6	6	6	5	6	1	2	2	2	2	2	5.33	1.21	-1.93	0.55	0.55	0.55	-0.28	0.55
chr11	72981586	73951585	3	6	6	6	6	6	1	2	2	2	2	2	5.5	1.22	-2.04	0.41	0.41	0.41	0.41	0.41
chr11	73951586	73961585	3	6	6	6	6	6	1	2	2	2	2	2	5.5	1.22	-2.04	0.41	0.41	0.41	0.41	0.41
chr11	73961586	74191585	3	6	6	6	6	6	1	2	2	2	2	2	5.5	1.22	-2.04	0.41	0.41	0.41	0.41	0.41
chr11	74191586	74201585	3	6	6	6	6	6	1	2	2	2	2	2	5.5	1.22	-2.04	0.41	0.41	0.41	0.41	0.41
chr11	74201586	78361585	3	6	6	6	6	6	1	2	2	2	2	2	5.5	1.22	-2.04	0.41	0.41	0.41	0.41	0.41
chr11	78361586	78371585	3	6	6	6	6	6	1	2	2	2	2	2	5.5	1.22	-2.04	0.41	0.41	0.41	0.41	0.41
chr11	78371586	82601585	3	6	6	6	6	6	1	2	2	2	2	2	5.5	1.22	-2.04	0.41	0.41	0.41	0.41	0.41
chr11	82601586	82611585	3	6	6	6	6	6	1	2	2	2	2	2	5.5	1.22	-2.04	0.41	0.41	0.41	0.41	0.41
chr11	82611586	83011585	3	6	6	6	6	6	1	2	2	2	2	2	5.5	1.22	-2.04	0.41	0.41	0.41	0.41	0.41
chr11	83011586	83021585	3	6	6	6	6	6	1	2	2	2	2	2	5.5	1.22	-2.04	0.41	0.41	0.41	0.41	0.41
chr11	83021586	89541380	3	6	6	6	6	6	1	2	2	2	2	2	5.5	1.22	-2.04	0.41	0.41	0.41	0.41	0.41
chr11	89541381	89551380	3	6	6	6	6	6	1	2	2	2	2	2	5.5	1.22	-2.04	0.41	0.41	0.41	0.41	0.41
chr11	89551381	89561780	3	5	5	6	6	6	1	2	2	2	2	2	5.17	1.17	-1.85	-0.14	-0.14	0.71	0.71	0.71
chr11	89561781	89651780	3	5	5	6	5	6	1	2	2	2	2	2	5	1.1	-1.83	0	0	0.91	0	0.91
chr11	89651781	89661780	3	5	5	6	5	6	1	2	2	2	2	2	5	1.1	-1.83	0	0	0.91	0	0.91
chr11	89661781	89754380	3	5	6	6	5	6	1	2	2	2	2	2	5.17	1.17	-1.85	-0.14	0.71	0.71	-0.14	0.71
chr11	89754381	89764380	3	5	6	6	5	6	1	2	2	2	2	2	5.17	1.17	-1.85	-0.14	0.71	0.71	-0.14	0.71
chr11	89764381	89823980	3	5	6	6	5	5	1	2	2	2	2	2	5	1.1	-1.83	0	0.91	0.91	0	0
chr11	89823981	89833980	3	5	6	6	5	5	1	2	2	2	2	2	5	1.1	-1.83	0	0.91	0.91	0	0
chr11	89833981	89902980	3	6	6	6	5	6	1	2	2	2	2	2	5.33	1.21	-1.93	0.55	0.55	0.55	-0.28	0.55
chr11	89902981	89912980	3	6	6	6	5	6	1	2	2	2	2	2	5.33	1.21	-1.93	0.55	0.55	0.55	-0.28	0.55
chr11	89912981	101695575	3	6	6	6	6	6	1	2	2	2	2	2	5.5	1.22	-2.04	0.41	0.41	0.41	0.41	0.41
chr11	101695576	101705575	3	6	6	6	6	6	1	2	2	2	2	2	5.5	1.22	-2.04	0.41	0.41	0.41	0.41	0.41
chr11	101705576	102445575	3	6	6	6	6	6	1	2	2	2	2	2	5.5	1.22	-2.04	0.41	0.41	0.41	0.41	0.41
chr11	102445576	102455575	3	6	6	6	6	6	1	2	2	2	2	2	5.5	1.22	-2.04	0.41	0.41	0.41	0.41	0.41
chr11	102455576	103885575	3	6	6	6	6	6	1	2	2	2	2	2	5.5	1.22	-2.04	0.41	0.41	0.41	0.41	0.41
chr11	103885576	103895575	3	6	6	6	6	6	1	2	2	2	2	2	5.5	1.22	-2.04	0.41	0.41	0.41	0.41	0.41

chr11	103895576	104025575	3	6	6	6	5	6	1	2	2	2	1	2	5.33	1.21	-1.93	0.55	0.55	0.55	-0.28	0.55
chr11	104025576	104035575	3	6	6	6	5	6	1	2	2	2	1	2	5.33	1.21	-1.93	0.55	0.55	0.55	-0.28	0.55
chr11	104035576	107635575	3	6	6	6	6	6	1	2	2	2	2	2	5.5	1.22	-2.04	0.41	0.41	0.41	0.41	0.41
chr11	107635576	107645575	3	6	6	6	6	6	1	2	2	2	2	2	5.5	1.22	-2.04	0.41	0.41	0.41	0.41	0.41
chr11	107645576	108815575	3	6	6	6	6	6	1	2	2	2	2	2	5.5	1.22	-2.04	0.41	0.41	0.41	0.41	0.41
chr11	108815576	108825575	3	6	6	6	6	6	1	2	2	2	2	2	5.5	1.22	-2.04	0.41	0.41	0.41	0.41	0.41
chr11	108825576	109955575	3	6	6	6	6	6	1	2	2	2	2	2	5.5	1.22	-2.04	0.41	0.41	0.41	0.41	0.41
chr11	109955576	109965575	3	6	6	6	6	6	1	2	2	2	2	2	5.5	1.22	-2.04	0.41	0.41	0.41	0.41	0.41
chr11	109965576	111475975	3	6	6	6	6	6	1	2	2	2	2	2	5.5	1.22	-2.04	0.41	0.41	0.41	0.41	0.41
chr11	111475976	111485975	3	6	6	6	6	6	1	2	2	2	2	2	5.5	1.22	-2.04	0.41	0.41	0.41	0.41	0.41
chr11	111485976	111496975	3	6	7	6	6	6	1	2	2	2	2	2	5.67	1.37	-1.95	0.24	0.98	0.24	0.24	0.24
chr11	111496976	111985975	3	6	7	6	6	6	1	2	2	2	2	2	5.67	1.37	-1.95	0.24	0.98	0.24	0.24	0.24
chr11	111985976	111995975	3	6	7	6	6	6	1	2	2	2	2	2	5.67	1.37	-1.95	0.24	0.98	0.24	0.24	0.24
chr11	111995976	112056975	3	6	6	6	6	6	1	2	2	2	2	2	5.5	1.22	-2.04	0.41	0.41	0.41	0.41	0.41
chr11	112056976	112066975	3	6	6	6	6	6	1	2	2	2	2	2	5.5	1.22	-2.04	0.41	0.41	0.41	0.41	0.41
chr11	112066976	112086775	3	6	6	6	6	6	1	2	2	2	2	2	5.5	1.22	-2.04	0.41	0.41	0.41	0.41	0.41
chr11	112086776	112096775	3	6	6	6	6	6	1	2	2	2	2	2	5.5	1.22	-2.04	0.41	0.41	0.41	0.41	0.41
chr11	112096776	112375575	3	6	6	6	6	6	1	2	2	2	2	2	5.5	1.22	-2.04	0.41	0.41	0.41	0.41	0.41
chr11	112375576	112385575	3	6	6	6	6	6	1	2	2	2	2	2	5.5	1.22	-2.04	0.41	0.41	0.41	0.41	0.41
chr11	112385576	113605575	3	6	6	6	6	6	1	2	2	2	2	2	5.5	1.22	-2.04	0.41	0.41	0.41	0.41	0.41
chr11	113605576	113615575	3	6	6	6	6	6	1	2	2	2	2	2	5.5	1.22	-2.04	0.41	0.41	0.41	0.41	0.41
chr11	113615576	114305575	3	6	6	6	6	6	1	2	2	2	2	2	5.5	1.22	-2.04	0.41	0.41	0.41	0.41	0.41
chr11	114305576	114315575	3	6	6	6	6	6	1	2	2	2	2	2	5.5	1.22	-2.04	0.41	0.41	0.41	0.41	0.41
chr11	114315576	114435575	3	6	6	6	6	6	1	2	2	2	2	2	5.5	1.22	-2.04	0.41	0.41	0.41	0.41	0.41
chr11	114435576	114445575	3	6	6	6	6	6	1	2	2	2	2	2	5.5	1.22	-2.04	0.41	0.41	0.41	0.41	0.41
chr11	114445576	114565575	3	6	6	6	5	6	1	2	2	2	2	2	5.33	1.21	-1.93	0.55	0.55	0.55	-0.28	0.55
chr11	114565576	114575575	3	6	6	6	5	6	1	2	2	2	2	2	5.33	1.21	-1.93	0.55	0.55	0.55	-0.28	0.55
chr11	114575576	129695775	3	6	6	6	6	6	1	2	2	2	2	2	5.5	1.22	-2.04	0.41	0.41	0.41	0.41	0.41
chr11	129695776	129705775	3	6	6	6	6	6	1	2	2	2	2	2	5.5	1.22	-2.04	0.41	0.41	0.41	0.41	0.41
chr11	129705776	130235775	3	6	6	6	6	6	1	2	2	2	2	2	5.5	1.22	-2.04	0.41	0.41	0.41	0.41	0.41
chr11	130235776	130245775	3	6	6	6	6	6	1	2	2	2	2	2	5.5	1.22	-2.04	0.41	0.41	0.41	0.41	0.41
chr11	130245776	133995775	3	6	6	6	6	6	1	2	2	2	2	2	5.5	1.22	-2.04	0.41	0.41	0.41	0.41	0.41
chr11	133995776	134005775	3	6	6	6	6	6	1	2	2	2	2	2	5.5	1.22	-2.04	0.41	0.41	0.41	0.41	0.41
chr11	134005776	134317175	3	6	6	6	6	6	1	2	2	2	2	2	5.5	1.22	-2.04	0.41	0.41	0.41	0.41	0.41
chr11	134317176	134327175	3	6	6	6	6	6	1	2	2	2	2	2	5.5	1.22	-2.04	0.41	0.41	0.41	0.41	0.41
chr11	134327176	134337175	3	6	6	7	6	6	1	2	2	1	2	2	5.67	1.37	-1.95	0.24	0.24	0.98	0.24	0.24
chr11	134337176	134347175	5	7	8	7	8	6	1	2	0	1	2	2	6.83	1.17	-1.57	0.14	1	0.14	1	-0.71
chr11	134347176	134926775	5	7	8	7	8	8	1	2	0	1	2	2	7.17	1.17	-1.85	-0.14	0.71	-0.14	0.71	0.71
chr12	69801	1891395	4	4	4	4	4	4	2	2	2	2	2	2	4	0	0	0	0	0	0	0
chr12	1891396	2871395	4	4	4	4	4	4	2	2	2	2	2	2	4	0	0	0	0	0	0	0
chr12	2871396	2881395	4	4	4	4	4	4	2	2	2	2	2	2	4	0	0	0	0	0	0	0
chr12	2881396	3081395	4	5	4	4	4	4	2	2	2	2	2	2	4.17	0.41	-0.41	2.04	-0.41	-0.41	-0.41	-0.41
chr12	3081396	3091395	4	5	4	4	4	4	2	2	2	2	2	2	4.17	0.41	-0.41	2.04	-0.41	-0.41	-0.41	-0.41
chr12	3091396	6091195	4	4	4	4	4	4	2	2	2	2	2	2	4	0	0	0	0	0	0	0
chr12	6091196	6101195	4	4	4	4	4	4	2	2	2	2	2	2	4	0	0	0	0	0	0	0
chr12	6101196	6370995	4	4	4	4	4	4	2	2	2	2	2	2	4	0	0	0	0	0	0	0
chr12	6370996	7281390	4	4	4	4	4	4	2	2	2	2	2	2	4	0	0	0	0	0	0	0
chr12	7281391	7291390	4	4	4	4	4	4	2	2	2	2	2	2	4	0	0	0	0	0	0	0
chr12	7291391	14514790	4	4	4	4	4	4	2	2	2	2	2	2	4	0	0	0	0	0	0	0
chr12	14514791	14524790	4	4	4	4	4	4	2	2	2	2	2	2	4	0	0	0	0	0	0	0
chr12	14524791	14634790	4	4	4	4	4	4	2	2	2	2	2	2	4	0	0	0	0	0	0	0
chr12	14634791	14644790	4	4	4	4	4	4	2	2	2	2	2	2	4	0	0	0	0	0	0	0
chr12	14644791	31655390	4	4	4	4	4	4	2	2	2	2	2	2	4	0	0	0	0	0	0	0
chr12	31655391	31665390	4	4	4	4	4	4	2	2	2	2	2	2	4	0	0	0	0	0	0	0
chr12	31665391	32165390	4	4	4	4	4	4	2	2	2	2	2	2	4	0	0	0	0	0	0	0
chr12	32165391	32175390	4	4	4	4	4	4	2	2	2	2	2	2	4	0	0	0	0	0	0	0
chr12	32175391	33285190	4	4	4	4	4	4	2	2	2	2	2	2	4	0	0	0	0	0	0	0
chr12	33285191	38209985	4	4	-	4	4	4	2	2	-	2	2	2	-	-	-	-	-	-	-	-
chr12	38209986	46060185	4	4	4	4	4	4	2	2	2	2	2	2	4	0	0	0	0	0	0	0
chr12	46060186	46070185	4	4	4	4	4	4	2	2	2	2	2	2	4	0	0	0	0	0	0	0
chr12	46070186	55110985	4	4	4	4	4	4	2	2	2	2	2	2	4	0	0	0	0	0	0	0
chr12	55110986	55120985	4	4	4	4	4	4	2	2	2	2	2	2	4	0	0	0	0	0	0	0
chr12	55120986	55741385	4	4	4	4	4	4	2	2	2	2	2	2	4	0	0	0	0	0	0	0
chr12	55741386	55751385	4	5	4	4	4	4	2	2	2	2	2	2	4.17	0.41	-0.41	2.04	-0.41	-0.41	-0.41	-0.41
chr12	55751386	56451385	4	5	5	5	5	5	2	2	2	2	2	2	4.83	0.41	-2.04	0.41	0.41	0.41	0.41	0.41
chr12	56451386	56461385	4	5	5	5	5	5	2	2	2	2	2	2	4.83	0.41	-2.04	0.41	0.41	0.41	0.41	0.41
chr12	56461386	56471385	4	5	5	4	4	5	2	2	2	2	2	2	4.5	0.55	-0.91	0.91	0.91	-0.91	-0.91	0.91
chr12	56471386	58471385	4	4	4	4	4	4	2	2	2	2	2	2	4	0	0	0	0	0	0	0
chr12	58471386	58481385	4	4	4	4	4	4	2	2	2	2	2	2	4	0	0	0	0	0	0	0
chr12	58481386	72011385	4	4	4	4	4	4	2	2	2	2	2	2	4	0	0	0	0	0	0	0
chr12	72011386	72291385	4	4	4	4	4	4	2	2	2	2	2	2	4	0	0	0	0	0	0	0
chr12	72291386	72301385	4	4	4	4	4	4	2	2	2	2	2	2	4	0	0	0	0	0	0	0
chr12	72301386	86782585	4	4	4	4	4	4	2	2	2	2	2	2	4	0	0	0	0	0	0	0
chr12	86782586	86792585	4	4	4	4	4	4	2	2	2	2	2	2	4	0	0	0	0	0	0	0
chr12	86792586	86992585	4	4	4	4	4	4	2	2	2	2	2	2	4	0	0	0	0	0	0	0
chr12	86992586	87002585	4	4	4	4	4	4	2	2	2	2	2	2	4	0	0	0	0	0	0	0

chr12	87002586	101452585	4	4	4	4	4	4	2	2	2	2	2	2	4	0	0	0	0	0	0	0
chr12	101452586	101462585	4	4	4	4	4	4	2	2	2	2	2	2	4	0	0	0	0	0	0	0
chr12	101462586	101632585	4	4	4	4	4	4	2	2	2	2	2	2	4	0	0	0	0	0	0	0
chr12	101632586	101642585	4	4	4	4	4	4	2	2	2	2	2	2	4	0	0	0	0	0	0	0
chr12	101642586	105992585	4	4	4	4	4	4	2	2	2	2	2	2	4	0	0	0	0	0	0	0
chr12	105992586	106002585	4	4	4	4	4	4	2	2	2	2	2	2	4	0	0	0	0	0	0	0
chr12	106002586	106172585	4	4	4	4	4	4	2	2	2	2	2	2	4	0	0	0	0	0	0	0
chr12	106172586	106182585	4	4	4	4	4	4	2	2	2	2	2	2	4	0	0	0	0	0	0	0
chr12	106182586	110592385	4	4	4	4	4	4	2	2	2	2	2	2	4	0	0	0	0	0	0	0
chr12	110592386	110602385	4	4	4	4	4	4	2	2	2	2	2	2	4	0	0	0	0	0	0	0
chr12	110602386	111082385	4	5	4	4	4	4	2	2	2	2	2	2	4.17	0.41	-0.41	2.04	-0.41	-0.41	-0.41	-0.41
chr12	111082386	111092385	4	5	4	4	4	4	2	2	2	2	2	2	4.17	0.41	-0.41	2.04	-0.41	-0.41	-0.41	-0.41
chr12	111092386	126043870	4	4	4	4	4	4	2	2	2	2	2	2	4	0	0	0	0	0	0	0
chr12	126043871	126053870	4	4	5	4	4	4	2	2	2	2	2	2	4.17	0.41	-0.41	-0.41	2.04	-0.41	-0.41	-0.41
chr12	126053871	126064270	5	4	5	4	4	4	2	2	2	2	2	2	4.33	0.52	1.29	-0.65	1.29	-0.65	-0.65	-0.65
chr12	126064271	126083870	5	4	5	4	4	6	2	2	2	2	2	2	4.67	0.82	0.41	-0.82	0.41	-0.82	-0.82	1.63
chr12	126083871	126093870	5	4	5	4	4	6	2	2	2	2	2	2	4.67	0.82	0.41	-0.82	0.41	-0.82	-0.82	1.63
chr12	126093871	126103870	5	4	4	4	4	6	2	2	2	2	2	2	4.5	0.84	0.6	-0.6	-0.6	-0.6	-0.6	1.79
chr12	126103871	128534070	4	4	4	4	4	4	2	2	2	2	2	2	4	0	0	0	0	0	0	0
chr12	128534071	128544070	4	4	4	4	4	4	2	2	2	2	2	2	4	0	0	0	0	0	0	0
chr12	128544071	128604070	4	4	4	4	3	4	2	2	2	2	1	2	3.83	0.41	0.41	0.41	0.41	0.41	-2.04	0.41
chr12	128604071	128614070	4	4	4	4	3	4	2	2	2	2	1	2	3.83	0.41	0.41	0.41	0.41	0.41	-2.04	0.41
chr12	128614071	131203870	4	4	4	4	4	4	2	2	2	2	2	2	4	0	0	0	0	0	0	0
chr12	131203871	131213870	4	4	4	4	4	4	2	2	2	2	2	2	4	0	0	0	0	0	0	0
chr12	131213871	131264070	4	4	4	4	4	4	2	2	2	2	2	2	4	0	0	0	0	0	0	0
chr12	131264071	131274070	4	4	4	4	4	4	2	2	2	2	2	2	4	0	0	0	0	0	0	0
chr12	131274071	131423870	4	4	4	4	4	4	2	2	2	2	2	2	4	0	0	0	0	0	0	0
chr12	131423871	131433870	4	4	4	4	4	4	2	2	2	2	2	2	4	0	0	0	0	0	0	0
chr12	131433871	131544070	4	4	4	4	4	4	2	2	2	2	2	2	4	0	0	0	0	0	0	0
chr12	131544071	131554070	4	4	4	4	4	4	2	2	2	2	2	2	4	0	0	0	0	0	0	0
chr12	131554071	132174670	4	4	4	4	4	4	2	2	2	2	2	2	4	0	0	0	0	0	0	0
chr12	132174671	132184670	4	4	4	4	4	4	2	2	2	2	2	2	4	0	0	0	0	0	0	0
chr12	132184671	133124665	4	4	4	4	4	4	2	2	2	2	2	2	4	0	0	0	0	0	0	0
chr12	133124666	133134665	4	4	4	4	4	4	2	2	2	2	2	2	4	0	0	0	0	0	0	0
chr12	133134666	133144665	4	4	4	4	4	4	2	2	2	2	2	2	4	0	0	0	0	0	0	0
chr12	133144666	133154665	4	4	4	4	4	4	2	2	2	2	2	2	4	0	0	0	0	0	0	0
chr12	133154666	133704665	4	4	4	4	4	4	2	2	2	2	2	2	4	0	0	0	0	0	0	0
chr12	133704666	133714665	4	4	4	4	4	4	2	2	2	2	2	2	4	0	0	0	0	0	0	0
chr12	133714666	133824665	4	4	4	4	4	4	2	2	2	2	2	2	4	0	0	0	0	0	0	0
chr13	19509801	22180800	3	3	3	3	3	3	1	1	0	0	0	0	3	0	0	0	0	0	0	0
chr13	22180801	32710800	2	3	3	3	3	3	0	1	0	0	0	0	2.83	0.41	-2.04	0.41	0.41	0.41	0.41	0.41
chr13	32710801	33160800	2	3	3	3	3	3	0	1	0	0	0	0	2.83	0.41	-2.04	0.41	0.41	0.41	0.41	0.41
chr13	33160801	33170800	2	3	3	3	3	3	0	1	0	0	0	0	2.83	0.41	-2.04	0.41	0.41	0.41	0.41	0.41
chr13	33170801	39501000	2	3	3	3	3	3	0	1	0	0	0	0	2.83	0.41	-2.04	0.41	0.41	0.41	0.41	0.41
chr13	39501001	39511000	2	3	3	3	3	3	0	1	0	0	0	0	2.83	0.41	-2.04	0.41	0.41	0.41	0.41	0.41
chr13	39511001	39571000	2	3	3	3	2	3	0	1	0	0	0	0	2.67	0.52	-1.29	0.65	0.65	0.65	-1.29	0.65
chr13	39571001	39581000	2	3	3	3	2	3	0	1	0	0	0	0	2.67	0.52	-1.29	0.65	0.65	0.65	-1.29	0.65
chr13	39581001	53347800	2	3	3	3	3	3	0	1	0	0	0	0	2.83	0.41	-2.04	0.41	0.41	0.41	0.41	0.41
chr13	53347801	53357800	2	3	3	3	3	3	0	1	0	0	0	0	2.83	0.41	-2.04	0.41	0.41	0.41	0.41	0.41
chr13	53357801	57488000	2	3	3	3	3	3	0	1	0	0	0	0	2.83	0.41	-2.04	0.41	0.41	0.41	0.41	0.41
chr13	57488001	57498000	2	3	3	3	3	3	0	1	0	0	0	0	2.83	0.41	-2.04	0.41	0.41	0.41	0.41	0.41
chr13	57498001	57698000	2	3	3	3	3	3	0	1	0	0	0	0	2.83	0.41	-2.04	0.41	0.41	0.41	0.41	0.41
chr13	57698001	57708000	2	3	3	3	3	3	0	1	0	0	0	0	2.83	0.41	-2.04	0.41	0.41	0.41	0.41	0.41
chr13	57708001	75831000	2	3	3	3	3	3	0	1	0	0	0	0	2.83	0.41	-2.04	0.41	0.41	0.41	0.41	0.41
chr13	75831001	75841000	2	3	3	3	3	3	0	1	0	0	0	0	2.83	0.41	-2.04	0.41	0.41	0.41	0.41	0.41
chr13	75841001	76171000	2	3	3	3	3	3	0	1	0	0	0	0	2.83	0.41	-2.04	0.41	0.41	0.41	0.41	0.41
chr13	76171001	76181000	2	3	3	3	3	3	0	1	0	0	0	0	2.83	0.41	-2.04	0.41	0.41	0.41	0.41	0.41
chr13	76181001	86751000	2	3	3	3	3	3	0	1	0	0	0	0	2.83	0.41	-2.04	0.41	0.41	0.41	0.41	0.41
chr13	86751001	86911195	2	3	3	3	3	3	0	1	0	0	0	0	2.83	0.41	-2.04	0.41	0.41	0.41	0.41	0.41
chr13	86911196	86951195	2	3	3	3	2	3	0	1	0	0	0	0	2.67	0.52	-1.29	0.65	0.65	0.65	-1.29	0.65
chr13	86951196	86961195	2	3	3	3	2	3	0	1	0	0	0	0	2.67	0.52	-1.29	0.65	0.65	0.65	-1.29	0.65
chr13	86961196	97912795	2	3	3	3	3	3	0	1	0	0	0	0	2.83	0.41	-2.04	0.41	0.41	0.41	0.41	0.41
chr13	97912796	97922795	2	3	3	3	3	3	0	1	0	0	0	0	2.83	0.41	-2.04	0.41	0.41	0.41	0.41	0.41
chr13	97922796	101442795	2	3	3	3	3	3	0	1	0	0	0	0	2.83	0.41	-2.04	0.41	0.41	0.41	0.41	0.41
chr13	101442796	101452795	2	3	3	3	3	3	0	1	0	0	0	0	2.83	0.41	-2.04	0.41	0.41	0.41	0.41	0.41
chr13	101452796	115093380	2	3	3	3	3	3	0	1	0	0	0	0	2.83	0.41	-2.04	0.41	0.41	0.41	0.41	0.41
chr14	19109801	19179800	4	6	6	6	6	6	2	3	2	2	3	3	5.67	0.82	-2.04	0.41	0.41	0.41	0.41	0.41
chr14	19179801	20650000	3	6	6	6	6	6	1	3	2	2	3	3	5.5	1.22	-2.04	0.41	0.41	0.41	0.41	0.41
chr14	20650001	20669800	3	6	6	6	6	6	1	3	3	2	3	3	5.5	1.22	-2.04	0.41	0.41	0.41	0.41	0.41
chr14	20669801	20790000	3	6	6	6	6	6	1	3	3	3	3	3	5.5	1.22	-2.04	0.41	0.41	0.41	0.41	0.41
chr14	20790001	20800000	3	6	6	6	6	6	1	3	3	3	3	3	5.5	1.22	-2.04	0.41	0.41	0.41	0.41	0.41
chr14	20800001	22330000	3	6	6	6	6	6	1	3	3	3	3	3	5.5	1.22	-2.04	0.41	0.41	0.41	0.41	0.41
chr14	22330001	22340000	3	6	6	6	6	6	1	3	3	3	3	3	5.5	1.22	-2.04	0.41	0.41	0.41	0.41	0.41
chr14	22340001	22400200	3	6	7	6	6	6	1	3	3	3	3	3	5.67	1.37	-1.95	0.24	0.98	0.24	0.24	0.24
chr14	22400201	22500000	3	6	7	6	6	7	1	3	3	3	3	3	5.83	1.47	-1.92	0.11	0.79	0.11	0.11	0.79
chr14	22500001	22550200	3	7	7	6	6	7	1	3	3	3	3	3	6	1.55	-1.94	0.65	0.65	0	0	0.65

chr14	22550201	22560200	3	7	7	6	6	7	1	3	3	3	3	3	6	1.55	-1.94	0.65	0.65	0	0	0.65
chr14	22560201	22599800	4	7	7	6	6	7	2	3	3	3	3	3	6.17	1.17	-1.85	0.71	0.71	-0.14	-0.14	0.71
chr14	22599801	22609800	4	7	7	6	6	7	2	3	3	3	3	3	6.17	1.17	-1.85	0.71	0.71	-0.14	-0.14	0.71
chr14	22609801	22660000	4	7	7	7	6	7	2	3	3	3	3	3	6.33	1.21	-1.93	0.55	0.55	0.55	-0.28	0.55
chr14	22660001	22670000	4	7	7	7	6	7	2	3	3	3	3	3	6.33	1.21	-1.93	0.55	0.55	0.55	-0.28	0.55
chr14	22670001	22700200	4	7	7	7	8	7	2	3	3	3	4	3	6.67	1.37	-1.95	0.24	0.24	0.24	0.98	0.24
chr14	22700201	22710200	4	7	7	7	8	7	2	3	3	3	4	3	6.67	1.37	-1.95	0.24	0.24	0.24	0.98	0.24
chr14	22710201	22940600	4	7	7	7	8	7	2	3	3	3	4	3	6.67	1.37	-1.95	0.24	0.24	0.24	0.98	0.24
chr14	22940601	22950600	4	7	7	7	8	7	2	3	3	3	4	3	6.67	1.37	-1.95	0.24	0.24	0.24	0.98	0.24
chr14	22950601	22960600	4	7	7	7	8	7	2	3	3	3	4	3	6.67	1.37	-1.95	0.24	0.24	0.24	0.98	0.24
chr14	22960601	22970600	4	7	7	6	8	7	2	3	3	3	4	3	6.5	1.38	-1.81	0.36	0.36	-0.36	1.09	0.36
chr14	22970601	22980600	3	6	7	6	8	7	1	3	3	3	4	3	6.17	1.72	-1.84	-0.1	0.48	-0.1	1.06	0.48
chr14	22980601	22990600	3	6	7	6	6	7	1	3	3	3	3	3	5.83	1.47	-1.92	0.11	0.79	0.11	0.11	0.79
chr14	22990601	25560200	3	6	6	6	6	6	1	3	3	3	3	3	5.5	1.22	-2.04	0.41	0.41	0.41	0.41	0.41
chr14	25560201	25570200	3	6	6	6	6	6	1	3	3	3	3	3	5.5	1.22	-2.04	0.41	0.41	0.41	0.41	0.41
chr14	25570201	31000400	3	6	6	6	6	6	1	3	3	3	3	3	5.5	1.22	-2.04	0.41	0.41	0.41	0.41	0.41
chr14	31000401	31010400	3	6	6	6	6	6	1	3	3	3	3	3	5.5	1.22	-2.04	0.41	0.41	0.41	0.41	0.41
chr14	31010401	31100200	3	6	6	6	6	6	1	3	3	3	3	3	5.5	1.22	-2.04	0.41	0.41	0.41	0.41	0.41
chr14	31100201	31110200	3	6	6	6	6	6	1	3	3	3	3	3	5.5	1.22	-2.04	0.41	0.41	0.41	0.41	0.41
chr14	31110201	31840200	3	6	6	7	6	6	1	3	3	3	3	3	5.67	1.37	-1.95	0.24	0.24	0.98	0.24	0.24
chr14	31840201	31850200	3	6	6	7	6	6	1	3	3	3	3	3	5.67	1.37	-1.95	0.24	0.24	0.98	0.24	0.24
chr14	31850201	32610400	3	6	6	6	6	6	1	3	3	3	3	3	5.5	1.22	-2.04	0.41	0.41	0.41	0.41	0.41
chr14	32610401	32620400	3	6	6	6	6	6	1	3	3	3	3	3	5.5	1.22	-2.04	0.41	0.41	0.41	0.41	0.41
chr14	32620401	33911400	3	6	6	6	6	6	1	3	3	3	3	3	5.5	1.22	-2.04	0.41	0.41	0.41	0.41	0.41
chr14	33911401	33921400	3	6	6	6	6	6	1	3	3	3	3	3	5.5	1.22	-2.04	0.41	0.41	0.41	0.41	0.41
chr14	33921401	34131400	3	6	6	6	5	6	1	3	3	3	2	3	5.33	1.21	-1.93	0.55	0.55	0.55	-0.28	0.55
chr14	34131401	34141400	3	6	6	6	5	6	1	3	3	3	2	3	5.33	1.21	-1.93	0.55	0.55	0.55	-0.28	0.55
chr14	34141401	39272600	3	6	6	6	6	6	1	3	3	3	3	3	5.5	1.22	-2.04	0.41	0.41	0.41	0.41	0.41
chr14	39272601	39282600	3	6	6	6	6	6	1	3	3	3	3	3	5.5	1.22	-2.04	0.41	0.41	0.41	0.41	0.41
chr14	39282601	39292800	3	7	6	6	6	7	1	3	3	3	3	3	5.83	1.47	-1.92	0.79	0.11	0.11	0.11	0.79
chr14	39292801	39312400	3	7	7	6	7	7	1	3	3	3	3	3	6.17	1.6	-1.98	0.52	0.52	-0.1	0.52	0.52
chr14	39312401	39603000	3	7	7	7	7	7	1	3	3	3	3	3	6.33	1.63	-2.04	0.41	0.41	0.41	0.41	0.41
chr14	39603001	39613000	3	7	7	7	7	7	1	3	3	3	3	3	6.33	1.63	-2.04	0.41	0.41	0.41	0.41	0.41
chr14	39613001	39742400	3	6	7	7	6	6	1	3	3	3	3	3	5.83	1.47	-1.92	0.11	0.79	0.79	0.11	0.11
chr14	39742401	39752400	3	6	7	7	6	6	1	3	3	3	3	3	5.83	1.47	-1.92	0.11	0.79	0.79	0.11	0.11
chr14	39752401	39912800	3	6	6	6	6	6	1	3	3	3	3	3	5.5	1.22	-2.04	0.41	0.41	0.41	0.41	0.41
chr14	39912801	39922800	3	6	6	6	6	6	1	3	3	3	3	3	5.5	1.22	-2.04	0.41	0.41	0.41	0.41	0.41
chr14	39922801	45352800	3	6	6	6	6	6	1	3	3	3	3	3	5.5	1.22	-2.04	0.41	0.41	0.41	0.41	0.41
chr14	45352801	45362800	3	6	6	6	6	6	1	3	3	3	3	3	5.5	1.22	-2.04	0.41	0.41	0.41	0.41	0.41
chr14	45362801	45822800	3	6	6	6	6	6	1	3	3	3	3	3	5.5	1.22	-2.04	0.41	0.41	0.41	0.41	0.41
chr14	45822801	45832800	3	6	6	6	6	6	1	3	3	3	3	3	5.5	1.22	-2.04	0.41	0.41	0.41	0.41	0.41
chr14	45832801	49522800	3	6	6	6	6	6	1	3	3	3	3	3	5.5	1.22	-2.04	0.41	0.41	0.41	0.41	0.41
chr14	49522801	49532800	3	6	6	6	6	6	1	3	3	3	3	3	5.5	1.22	-2.04	0.41	0.41	0.41	0.41	0.41
chr14	49532801	58633600	3	6	6	6	6	6	1	3	3	3	3	3	5.5	1.22	-2.04	0.41	0.41	0.41	0.41	0.41
chr14	58633601	58643600	3	6	6	6	6	6	1	3	3	3	3	3	5.5	1.22	-2.04	0.41	0.41	0.41	0.41	0.41
chr14	58643601	58653800	3	6	6	6	6	6	1	3	3	3	3	3	5.5	1.22	-2.04	0.41	0.41	0.41	0.41	0.41
chr14	58653801	58663800	3	6	6	6	6	6	1	3	3	3	3	3	5.5	1.22	-2.04	0.41	0.41	0.41	0.41	0.41
chr14	58663801	58963600	3	6	6	6	6	6	1	3	3	3	3	3	5.5	1.22	-2.04	0.41	0.41	0.41	0.41	0.41
chr14	58963601	58973600	3	6	6	6	6	6	1	3	3	3	3	3	5.5	1.22	-2.04	0.41	0.41	0.41	0.41	0.41
chr14	58973601	62394200	3	6	6	6	6	6	1	3	3	3	3	3	5.5	1.22	-2.04	0.41	0.41	0.41	0.41	0.41
chr14	62394201	62404200	3	6	6	6	6	6	1	3	3	3	3	3	5.5	1.22	-2.04	0.41	0.41	0.41	0.41	0.41
chr14	62404201	63414000	3	6	6	6	6	6	1	3	3	3	3	3	5.5	1.22	-2.04	0.41	0.41	0.41	0.41	0.41
chr14	63414001	63424000	3	6	6	6	6	6	1	3	3	3	3	3	5.5	1.22	-2.04	0.41	0.41	0.41	0.41	0.41
chr14	63424001	73974800	4	4	4	4	4	4	2	2	1	1	1	1	4	0	0	0	0	0	0	0
chr14	73974801	73984800	4	4	4	4	4	4	2	2	1	1	1	1	4	0	0	0	0	0	0	0
chr14	73984801	74024800	4	4	4	4	4	6	2	2	1	1	1	1	4.33	0.82	-0.41	-0.41	-0.41	-0.41	-0.41	2.04
chr14	74024801	74034800	4	4	4	4	4	6	2	2	1	1	1	1	4.33	0.82	-0.41	-0.41	-0.41	-0.41	-0.41	2.04
chr14	74034801	77274600	4	4	4	4	4	4	2	2	1	1	1	1	4	0	0	0	0	0	0	0
chr14	77274601	77284600	4	4	4	4	4	4	2	2	1	1	1	1	4	0	0	0	0	0	0	0
chr14	77284601	77294800	4	4	4	3	4	4	2	2	1	0	1	1	3.83	0.41	0.41	0.41	0.41	-2.04	0.41	0.41
chr14	77294801	78314800	4	4	4	3	4	3	2	2	1	0	1	0	3.67	0.52	0.65	0.65	0.65	-1.29	0.65	-1.29
chr14	78314801	78324800	4	4	4	3	4	3	2	2	1	0	1	0	3.67	0.52	0.65	0.65	0.65	-1.29	0.65	-1.29
chr14	78324801	88129800	4	4	4	3	4	3	2	2	1	0	1	0	3.67	0.52	0.65	0.65	0.65	-1.29	0.65	-1.29
chr14	88129801	88139800	4	4	4	3	4	3	2	2	1	0	1	0	3.67	0.52	0.65	0.65	0.65	-1.29	0.65	-1.29
chr14	88139801	97059800	4	4	4	3	4	3	2	2	1	0	1	0	3.67	0.52	0.65	0.65	0.65	-1.29	0.65	-1.29
chr14	97059801	97069800	4	4	4	3	4	3	2	2	1	0	1	0	3.67	0.52	0.65	0.65	0.65	-1.29	0.65	-1.29
chr14	97069801	99739800	4	4	4	3	4	3	2	2	1	0	1	0	3.67	0.52	0.65	0.65	0.65	-1.29	0.65	-1.29
chr14	99739801	99749800	4	4	4	3	4	3	2	2	1	0	1	0	3.67	0.52	0.65	0.65	0.65	-1.29	0.65	-1.29
chr14	99749801	107271000	4	4	4	3	4	3	2	2	1	0	1	0	3.67	0.52	0.65	0.65	0.65	-1.29	0.65	-1.29
chr15	20709801	22067420	4	4	4	4	4	4	2	2	2	2	1	2	4	0	0	0	0	0	0	0
chr15	22067421	22077420	4	4	4	4	4	4	2	2	2	2	1	2	4	0	0	0	0	0	0	0
chr15	22077421	22438015	4	4	4	4	4	4	2	2	2	2	1	2	4	0	0	0	0	0	0	0
chr15	22438016	22448015	4	4	4	4	4	4	2	2	2	2	1	2	4	0	0	0	0	0	0	0
chr15	22448016	73589200	4	4	4	4	4	4	2	2	2	2	2	2	4	0	0	0	0	0	0	0
chr15	73589201	73599200	4	4	4	4	4	4	2	2	2	2	2	2	4	0	0	0	0	0	0	0
chr15	73599201	73769200	4	4	4	4	4	4	2	2	2	2	2	2	4	0	0	0	0	0	0	0

chr15	73769201	73779200	4	4	4	4	4	4	2	2	2	2	2	2	4	0	0	0	0	0	0	0
chr15	73779201	85061795	4	4	4	4	4	4	2	2	2	2	2	2	4	0	0	0	0	0	0	0
chr15	85061796	85071795	4	4	4	4	4	4	2	2	2	2	2	2	4	0	0	0	0	0	0	0
chr15	85071796	85651795	4	4	4	4	4	4	2	2	2	2	2	2	4	0	0	0	0	0	0	0
chr15	85651796	85661795	4	4	4	4	4	4	2	2	2	2	2	2	4	0	0	0	0	0	0	0
chr15	85661796	86831995	4	4	4	4	4	4	2	2	2	2	2	2	4	0	0	0	0	0	0	0
chr15	86831996	86841995	4	4	4	4	4	4	2	2	2	2	2	2	4	0	0	0	0	0	0	0
chr15	86841996	87852195	4	4	4	4	4	4	2	2	2	2	2	2	4	0	0	0	0	0	0	0
chr15	87852196	88631995	4	4	4	5	4	5	2	2	2	2	2	2	4.33	0.52	-0.65	-0.65	-0.65	1.29	-0.65	1.29
chr15	88631996	88641995	4	4	4	5	4	5	2	2	2	2	2	2	4.33	0.52	-0.65	-0.65	-0.65	1.29	-0.65	1.29
chr15	88641996	93341995	4	4	4	5	4	5	2	2	2	2	2	2	4.33	0.52	-0.65	-0.65	-0.65	1.29	-0.65	1.29
chr15	93341996	93351995	4	4	4	5	4	5	2	2	2	2	2	2	4.33	0.52	-0.65	-0.65	-0.65	1.29	-0.65	1.29
chr15	93351996	93381195	4	4	4	5	4	5	2	2	2	2	2	2	4.33	0.52	-0.65	-0.65	-0.65	1.29	-0.65	1.29
chr15	93381196	93551195	4	5	4	5	4	5	2	2	2	2	2	2	4.5	0.55	-0.91	0.91	-0.91	0.91	-0.91	0.91
chr15	93551196	93561195	4	5	4	5	4	5	2	2	2	2	2	2	4.5	0.55	-0.91	0.91	-0.91	0.91	-0.91	0.91
chr15	93561196	93571995	4	4	4	5	4	5	2	2	2	2	2	2	4.33	0.52	-0.65	-0.65	-0.65	1.29	-0.65	1.29
chr15	93571996	102433795	4	4	4	5	4	5	2	2	2	2	2	2	4.33	0.52	-0.65	-0.65	-0.65	1.29	-0.65	1.29
chr15	102433796	102443795	4	4	4	5	4	5	2	2	2	2	2	2	4.33	0.52	-0.65	-0.65	-0.65	1.29	-0.65	1.29
chr15	102443796	102503795	4	5	4	5	5	5	2	2	2	2	2	2	4.67	0.52	-1.29	0.65	-1.29	0.65	0.65	0.65
chr16	69801	5121400	3	3	3	3	3	3	1	1	0	0	0	0	3	0	0	0	0	0	0	0
chr16	5121401	5131400	3	3	3	3	3	3	1	1	0	0	0	0	3	0	0	0	0	0	0	0
chr16	5131401	8451400	3	3	3	3	3	3	1	1	0	0	0	0	3	0	0	0	0	0	0	0
chr16	8451401	8461400	3	3	3	3	3	3	1	1	0	0	0	0	3	0	0	0	0	0	0	0
chr16	8461401	16352195	3	3	3	3	3	3	1	1	0	0	0	0	3	0	0	0	0	0	0	0
chr16	16352196	16362195	3	3	3	3	3	3	1	1	0	0	0	0	3	0	0	0	0	0	0	0
chr16	16362196	16542195	3	3	3	3	3	3	1	1	0	0	1	1	3	0	0	0	0	0	0	0
chr16	16542196	16552195	3	3	3	3	3	3	1	1	0	0	1	1	3	0	0	0	0	0	0	0
chr16	16552196	16722195	2	2	2	2	2	2	0	0	0	0	0	0	2	0	0	0	0	0	0	0
chr16	16722196	16732195	2	2	2	2	2	2	0	0	0	0	0	0	2	0	0	0	0	0	0	0
chr16	16732196	16742395	2	3	2	2	2	2	0	1	0	0	0	0	2.17	0.41	-0.41	2.04	-0.41	-0.41	-0.41	-0.41
chr16	16742396	16812595	2	3	2	2	3	2	0	1	0	0	1	0	2.33	0.52	-0.65	1.29	-0.65	-0.65	1.29	-0.65
chr16	16812596	16822595	2	3	2	3	3	2	0	1	0	0	1	0	2.5	0.55	-0.91	0.91	-0.91	0.91	0.91	-0.91
chr16	16822596	16832595	2	3	3	3	3	2	0	1	0	0	1	0	2.67	0.52	-1.29	0.65	0.65	0.65	0.65	-1.29
chr16	16832596	16902395	3	3	3	3	3	3	0	1	0	0	1	0	3	0	0	0	0	0	0	0
chr16	16902396	16912395	3	3	3	3	3	3	0	1	0	0	1	0	3	0	0	0	0	0	0	0
chr16	16912396	18282595	3	3	3	3	3	3	0	1	0	0	0	0	3	0	0	0	0	0	0	0
chr16	18282596	18302595	2	3	3	3	3	3	0	1	0	0	0	0	2.83	0.41	-2.04	0.41	0.41	0.41	0.41	0.41
chr16	18302596	18312595	2	3	3	3	3	3	0	1	0	0	0	0	2.83	0.41	-2.04	0.41	0.41	0.41	0.41	0.41
chr16	18312596	18342195	2	3	2	2	2	3	0	1	0	0	1	0	2.33	0.52	-0.65	1.29	-0.65	-0.65	-0.65	1.29
chr16	18342196	18352195	2	3	2	2	2	3	0	1	0	0	1	0	2.33	0.52	-0.65	1.29	-0.65	-0.65	-0.65	1.29
chr16	18352196	18712195	2	2	2	2	2	2	0	0	0	0	1	1	2	0	0	0	0	0	0	0
chr16	18712196	18722595	2	2	2	2	2	2	0	0	0	0	1	1	2	0	0	0	0	0	0	0
chr16	18722596	18732595	2	2	3	3	2	2	0	0	0	0	1	1	2.33	0.52	-0.65	-0.65	1.29	1.29	-0.65	-0.65
chr16	18732596	19073595	3	3	3	3	3	3	1	1	0	0	0	0	3	0	0	0	0	0	0	0
chr16	19073596	19083595	3	3	3	3	3	3	1	1	0	0	0	0	3	0	0	0	0	0	0	0
chr16	19083596	19153595	3	3	4	3	3	3	1	1	0	0	0	0	3.17	0.41	-0.41	-0.41	2.04	-0.41	-0.41	-0.41
chr16	19153596	19163595	3	3	4	3	3	3	1	1	0	0	0	0	3.17	0.41	-0.41	-0.41	2.04	-0.41	-0.41	-0.41
chr16	19163596	21473195	3	3	3	3	3	3	1	1	0	0	0	0	3	0	0	0	0	0	0	0
chr16	21473196	21483195	3	3	3	3	3	3	1	1	0	0	0	0	3	0	0	0	0	0	0	0
chr16	21483196	21493395	3	2	3	3	3	3	1	0	0	0	0	0	2.83	0.41	0.41	-2.04	0.41	0.41	0.41	0.41
chr16	21493396	21593195	2	2	2	2	2	2	0	0	1	1	0	0	2	0	0	0	0	0	0	0
chr16	21593196	21603195	2	2	2	2	2	2	0	0	1	1	0	0	2	0	0	0	0	0	0	0
chr16	21603196	22433395	3	3	3	3	3	3	1	1	0	0	0	0	3	0	0	0	0	0	0	0
chr16	22433396	22443395	3	3	3	3	3	3	1	1	0	0	0	0	3	0	0	0	0	0	0	0
chr16	22443396	22453995	3	3	3	2	3	3	1	1	0	0	0	0	2.83	0.41	0.41	0.41	0.41	-2.04	0.41	0.41
chr16	22453996	22533995	3	3	3	2	3	3	1	1	0	0	0	0	2.83	0.41	0.41	0.41	0.41	-2.04	0.41	0.41
chr16	22533996	22543995	3	3	3	2	3	3	1	1	0	0	0	0	2.83	0.41	0.41	0.41	0.41	-2.04	0.41	0.41
chr16	22543996	22703195	2	2	2	2	2	2	0	0	0	0	0	0	2	0	0	0	0	0	0	0
chr16	22703196	22713195	2	2	2	2	2	2	0	0	0	0	0	0	2	0	0	0	0	0	0	0
chr16	22713196	22723595	2	3	2	2	3	3	0	1	0	0	0	0	2.5	0.55	-0.91	0.91	-0.91	-0.91	0.91	0.91
chr16	22723596	23083795	3	3	3	3	3	3	1	1	0	0	0	0	3	0	0	0	0	0	0	0
chr16	23083796	23093795	3	3	3	3	3	3	1	1	0	0	0	0	3	0	0	0	0	0	0	0
chr16	23093796	23343395	3	3	3	3	3	3	1	1	0	0	0	0	3	0	0	0	0	0	0	0
chr16	23343396	23353395	3	3	3	3	3	3	1	1	0	0	0	0	3	0	0	0	0	0	0	0
chr16	23353396	23683395	3	3	3	3	3	3	1	1	0	0	0	0	3	0	0	0	0	0	0	0
chr16	23683396	23693395	3	3	3	3	3	3	1	1	0	0	0	0	3	0	0	0	0	0	0	0
chr16	23693396	27134995	3	3	3	3	3	3	1	1	0	0	0	0	3	0	0	0	0	0	0	0
chr16	27134996	27144995	3	3	3	3	3	3	1	1	0	0	0	0	3	0	0	0	0	0	0	0
chr16	27144996	28335795	3	3	3	3	3	3	1	1	0	0	0	0	3	0	0	0	0	0	0	0
chr16	28335796	28345795	3	3	3	3	3	3	1	1	0	0	0	0	3	0	0	0	0	0	0	0
chr16	28345796	28385795	3	3	3	3	3	3	1	1	0	0	0	0	3	0	0	0	0	0	0	0
chr16	28385796	28395795	3	3	3	3	3	3	1	1	0	0	0	0	3	0	0	0	0	0	0	0
chr16	28395796	32035395	3	3	3	3	3	3	1	1	0	0	0	0	3	0	0	0	0	0	0	0
chr16	32035396	32045395	3	3	3	3	3	3	1	1	0	0	0	0	3	0	0	0	0	0	0	0
chr16	32045396	32245995	3	4	4	3	4	4	1	2	1	0	2	2	3.67	0.52	-1.29	0.65	0.65	-1.29	0.65	0.65
chr16	32245996	32255995	3	4	4	3	4	4	1	2	1	0	2	2	3.67	0.52	-1.29	0.65	0.65	-1.29	0.65	0.65

chr16	32255996	32276395	4	4	4	3	4	4	2	2	1	0	2	2	3.83	0.41	0.41	0.41	0.41	-2.04	0.41	0.41
chr16	32276396	32286395	4	4	4	3	4	4	2	2	1	0	2	2	3.83	0.41	0.41	0.41	0.41	-2.04	0.41	0.41
chr16	32286396	32296395	4	4	4	4	4	4	2	2	1	1	2	2	4	0	0	0	0	0	0	0
chr16	32296396	32306395	4	4	4	4	4	4	2	2	1	1	2	2	4	0	0	0	0	0	0	0
chr16	32306396	32646395	4	4	4	4	4	4	2	2	1	1	2	2	4	0	0	0	0	0	0	0
chr16	32646396	32656395	4	4	4	4	4	4	2	2	1	1	2	2	4	0	0	0	0	0	0	0
chr16	32656396	32666395	3	4	3	3	3	4	1	2	1	1	1	2	3.33	0.52	-0.65	1.29	-0.65	-0.65	-0.65	1.29
chr16	32666396	32855395	3	4	3	3	3	3	1	2	1	1	1	1	3.17	0.41	-0.41	2.04	-0.41	-0.41	-0.41	-0.41
chr16	32855396	32865395	3	4	3	3	3	3	1	2	1	1	1	1	3.17	0.41	-0.41	2.04	-0.41	-0.41	-0.41	-0.41
chr16	32865396	32885595	3	3	3	3	3	3	1	1	1	1	1	1	3	0	0	0	0	0	0	0
chr16	32885596	32895595	3	3	3	3	3	3	1	1	1	1	1	1	3	0	0	0	0	0	0	0
chr16	32895596	32935595	3	3	3	3	2	2	1	1	1	1	1	1	2.67	0.52	0.65	0.65	0.65	0.65	-1.29	-1.29
chr16	32935596	32945595	3	3	3	3	2	2	1	1	1	1	1	1	2.67	0.52	0.65	0.65	0.65	0.65	-1.29	-1.29
chr16	32945596	33275395	3	3	3	3	3	3	1	1	1	1	1	1	3	0	0	0	0	0	0	0
chr16	33275396	33285395	3	3	3	3	3	3	1	1	1	1	1	1	3	0	0	0	0	0	0	0
chr16	33285396	33415595	3	4	3	3	3	3	1	2	1	1	1	1	3.17	0.41	-0.41	2.04	-0.41	-0.41	-0.41	-0.41
chr16	33415596	33425595	3	4	3	3	3	3	1	2	1	1	1	1	3.17	0.41	-0.41	2.04	-0.41	-0.41	-0.41	-0.41
chr16	33425596	33585595	4	4	4	3	4	4	2	2	1	1	2	2	3.83	0.41	0.41	0.41	0.41	-2.04	0.41	0.41
chr16	33585596	33595595	4	4	4	3	4	4	2	2	1	1	2	2	3.83	0.41	0.41	0.41	0.41	-2.04	0.41	0.41
chr16	33595596	33625995	4	4	4	3	3	4	2	2	1	1	1	2	3.67	0.52	0.65	0.65	0.65	-1.29	-1.29	0.65
chr16	33625996	33636395	4	4	3	3	3	4	2	2	0	1	1	2	3.5	0.55	0.91	0.91	-0.91	-0.91	-0.91	0.91
chr16	33636396	33725395	4	4	3	3	3	3	2	2	0	1	1	1	3.33	0.52	1.29	1.29	-0.65	-0.65	-0.65	-0.65
chr16	33725396	33735395	4	4	3	3	3	3	2	2	0	1	1	1	3.33	0.52	1.29	1.29	-0.65	-0.65	-0.65	-0.65
chr16	33735396	33785395	4	5	5	4	6	5	2	2	2	1	3	2	4.83	0.75	-1.11	0.22	0.22	-1.11	1.55	0.22
chr16	33785396	33795395	4	5	5	4	6	5	2	2	2	1	3	2	4.83	0.75	-1.11	0.22	0.22	-1.11	1.55	0.22
chr16	33795396	34186195	4	4	3	4	4	4	2	2	0	1	2	2	3.83	0.41	0.41	0.41	-2.04	0.41	0.41	0.41
chr16	34186196	34196195	4	4	3	4	4	4	2	2	0	1	2	2	3.83	0.41	0.41	0.41	-2.04	0.41	0.41	0.41
chr16	34196196	34585595	5	4	3	4	4	4	2	2	0	1	2	2	4	0.63	1.58	0	-1.58	0	0	0
chr16	34585596	46395990	5	4	3	4	4	4	2	2	0	1	2	2	4	0.63	1.58	0	-1.58	0	0	0
chr16	46395991	46455990	5	5	4	5	5	5	2	2	1	1	2	2	4.83	0.41	0.41	0.41	-2.04	0.41	0.41	0.41
chr16	46455991	46465990	5	3	3	3	5	5	2	1	0	0	2	2	4	1.1	0.91	-0.91	-0.91	-0.91	0.91	0.91
chr16	46465991	49377790	3	3	3	3	3	3	1	1	0	0	0	0	3	0	0	0	0	0	0	0
chr16	49377791	49387790	3	3	3	3	3	3	1	1	0	0	0	0	3	0	0	0	0	0	0	0
chr16	49387791	49398190	4	4	3	3	4	4	1	1	0	0	1	1	3.67	0.52	0.65	0.65	-1.29	-1.29	0.65	0.65
chr16	49398191	49877790	4	4	3	4	4	4	1	1	0	1	1	1	3.83	0.41	0.41	0.41	-2.04	0.41	0.41	0.41
chr16	49877791	49887790	4	4	3	4	4	4	1	1	0	1	1	1	3.83	0.41	0.41	0.41	-2.04	0.41	0.41	0.41
chr16	49887791	49898190	3	3	3	4	3	3	0	1	0	1	0	0	3.17	0.41	-0.41	-0.41	-0.41	2.04	-0.41	-0.41
chr16	49898191	59530590	3	3	3	3	3	3	0	1	0	0	0	0	3	0	0	0	0	0	0	0
chr16	59530591	59540590	3	3	3	3	3	3	0	1	0	0	0	0	3	0	0	0	0	0	0	0
chr16	59540591	59790590	3	3	3	3	2	3	0	1	0	0	0	0	2.83	0.41	0.41	0.41	0.41	0.41	-2.04	0.41
chr16	59790591	59800590	3	3	3	3	2	3	0	1	0	0	0	0	2.83	0.41	0.41	0.41	0.41	0.41	-2.04	0.41
chr16	59800591	66360590	3	3	3	3	3	3	0	1	0	0	0	0	3	0	0	0	0	0	0	0
chr16	66360591	66370590	3	3	3	3	3	3	0	1	0	0	0	0	3	0	0	0	0	0	0	0
chr16	66370591	70150790	3	3	3	3	3	3	0	1	0	0	0	0	3	0	0	0	0	0	0	0
chr16	70150791	70160790	3	3	3	3	3	3	0	1	0	0	0	0	3	0	0	0	0	0	0	0
chr16	70160791	70200790	4	3	3	3	4	4	2	1	0	0	2	2	3.5	0.55	0.91	-0.91	-0.91	-0.91	0.91	0.91
chr16	70200791	70210790	4	3	3	3	4	4	2	1	0	0	2	2	3.5	0.55	0.91	-0.91	-0.91	-0.91	0.91	0.91
chr16	70210791	70840790	3	3	3	3	3	3	1	1	0	0	0	0	3	0	0	0	0	0	0	0
chr16	70840791	70850790	3	3	3	3	3	3	1	1	0	0	0	0	3	0	0	0	0	0	0	0
chr16	70850791	70861590	3	3	3	3	4	4	1	1	0	0	1	1	3.33	0.52	-0.65	-0.65	-0.65	-0.65	1.29	1.29
chr16	70861591	70881190	3	4	3	3	4	4	1	1	0	0	1	1	3.5	0.55	-0.91	0.91	-0.91	-0.91	0.91	0.91
chr16	70881191	70891190	3	4	3	3	4	4	1	1	0	0	1	1	3.5	0.55	-0.91	0.91	-0.91	-0.91	0.91	0.91
chr16	70891191	71191190	3	4	4	3	4	4	1	1	0	0	1	1	3.67	0.52	-1.29	0.65	0.65	-1.29	0.65	0.65
chr16	71191191	71201190	3	4	3	3	4	4	1	1	0	0	1	1	3.5	0.55	-0.91	0.91	-0.91	-0.91	0.91	0.91
chr16	71201191	71211190	3	3	3	3	3	3	1	1	0	0	0	0	3	0	0	0	0	0	0	0
chr16	71211191	73330790	3	3	3	3	3	3	0	1	0	0	0	0	3	0	0	0	0	0	0	0
chr16	73330791	73340790	3	3	3	3	3	3	0	1	0	0	0	0	3	0	0	0	0	0	0	0
chr16	73340791	74180790	3	3	3	3	3	3	0	1	0	0	0	0	3	0	0	0	0	0	0	0
chr16	74180791	74190790	3	3	3	3	3	3	0	1	0	0	0	0	3	0	0	0	0	0	0	0
chr16	74190791	74351790	3	3	3	3	3	3	0	1	0	0	0	0	3	0	0	0	0	0	0	0
chr16	74351791	74361790	3	3	3	3	3	3	0	1	0	0	0	0	3	0	0	0	0	0	0	0
chr16	74361791	74381190	3	4	4	3	3	3	0	2	0	0	0	0	3.33	0.52	-0.65	1.29	1.29	-0.65	-0.65	-0.65
chr16	74381191	74391190	3	4	4	3	3	3	0	2	0	0	0	0	3.33	0.52	-0.65	1.29	1.29	-0.65	-0.65	-0.65
chr16	74391191	74460790	4	4	4	3	4	4	2	2	0	0	1	2	3.83	0.41	0.41	0.41	0.41	-2.04	0.41	0.41
chr16	74460791	74470790	4	4	4	3	4	4	2	2	0	0	1	2	3.83	0.41	0.41	0.41	0.41	-2.04	0.41	0.41
chr16	74470791	88624185	3	3	3	3	3	3	0	1	0	0	0	0	3	0	0	0	0	0	0	0
chr16	88624186	88634185	3	3	3	3	3	3	0	1	0	0	0	0	3	0	0	0	0	0	0	0
chr16	88634186	88934185	3	4	5	4	4	4	0	1	0	0	0	0	4	0.63	-1.58	0	1.58	0	0	0
chr16	88934186	88944185	3	4	5	4	4	4	0	1	0	0	0	0	4	0.63	-1.58	0	1.58	0	0	0
chr16	88944186	88954585	3	4	5	4	3	3	0	1	0	0	0	0	3.67	0.82	-0.82	0.41	1.63	0.41	-0.82	-0.82
chr16	88954586	88964585	3	3	5	4	3	3	0	1	0	0	0	0	3.5	0.84	-0.6	-0.6	1.79	0.6	-0.6	-0.6
chr16	88964586	90154585	3	3	3	3	3	3	0	1	0	0	0	0	3	0	0	0	0	0	0	0
chr16	90154586	90164585	3	3	3	3	3	3	0	1	0	0	0	0	3	0	0	0	0	0	0	0
chr16	90164586	90174785	3	3	3	4	3	4	0	1	0	0	0	1	3.33	0.52	-0.65	-0.65	-0.65	1.29	-0.65	1.29
chr16	90174786	90184785	3	3	3	4	4	4	0	1	0	0	1	1	3.5	0.55	-0.91	-0.91	-0.91	0.91	0.91	0.91
chr16	90184786	90274585	3	5	4	4	4	4	0	2	0	0	1	1	4	0.63	-1.58	1.58	0	0	0	0

chr17	5451996	5471795	4	5	5	5	5	5	2	2	2	2	2	2	4.83	0.41	-2.04	0.41	0.41	0.41	0.41	0.41
chr17	5471796	5481795	4	5	5	5	5	5	2	2	2	2	2	2	4.83	0.41	-2.04	0.41	0.41	0.41	0.41	0.41
chr17	5481796	6301795	4	5	5	5	5	5	2	2	2	2	2	2	4.83	0.41	-2.04	0.41	0.41	0.41	0.41	0.41
chr17	6301796	6311795	4	5	5	5	5	5	2	2	2	2	2	2	4.83	0.41	-2.04	0.41	0.41	0.41	0.41	0.41
chr17	6311796	6481995	4	5	5	5	5	5	2	2	2	2	2	2	4.83	0.41	-2.04	0.41	0.41	0.41	0.41	0.41
chr17	6481996	6491995	4	5	5	5	5	5	2	2	2	2	2	2	4.83	0.41	-2.04	0.41	0.41	0.41	0.41	0.41
chr17	6491996	7221795	4	5	5	5	5	5	2	2	2	2	2	2	4.83	0.41	-2.04	0.41	0.41	0.41	0.41	0.41
chr17	7221796	7231795	4	5	5	5	5	5	2	2	2	2	2	2	4.83	0.41	-2.04	0.41	0.41	0.41	0.41	0.41
chr17	7231796	7241995	4	5	5	7	7	5	2	2	2	2	2	2	5.5	1.22	-1.22	-0.41	-0.41	1.22	1.22	-0.41
chr17	7241996	7681795	4	6	7	7	7	6	2	2	2	2	2	2	6.17	1.17	-1.85	-0.14	0.71	0.71	0.71	-0.14
chr17	7681796	7691795	4	6	7	7	7	6	2	2	2	2	2	2	6.17	1.17	-1.85	-0.14	0.71	0.71	0.71	-0.14
chr17	7691796	7701995	4	6	7	7	5	5	2	2	2	2	2	2	5.67	1.21	-1.38	0.28	1.1	1.1	-0.55	-0.55
chr17	7701996	9112195	4	5	5	5	5	5	2	2	2	2	2	2	4.83	0.41	-2.04	0.41	0.41	0.41	0.41	0.41
chr17	9112196	9122195	4	5	5	5	5	5	2	2	2	2	2	2	4.83	0.41	-2.04	0.41	0.41	0.41	0.41	0.41
chr17	9122196	10562195	4	5	5	5	5	5	2	2	2	2	2	2	4.83	0.41	-2.04	0.41	0.41	0.41	0.41	0.41
chr17	10562196	10572195	4	5	5	5	5	5	2	2	2	2	2	2	4.83	0.41	-2.04	0.41	0.41	0.41	0.41	0.41
chr17	10572196	10662195	4	5	5	5	6	5	2	2	2	2	3	2	5	0.63	-1.58	0	0	0	1.58	0
chr17	10662196	10672195	4	5	5	5	6	5	2	2	2	2	3	2	5	0.63	-1.58	0	0	0	1.58	0
chr17	10672196	15342195	4	5	5	5	5	5	2	2	2	2	2	2	4.83	0.41	-2.04	0.41	0.41	0.41	0.41	0.41
chr17	15342196	15352195	4	5	5	5	5	5	2	2	2	2	2	2	4.83	0.41	-2.04	0.41	0.41	0.41	0.41	0.41
chr17	15352196	19836595	4	5	5	5	5	5	2	2	2	2	2	2	4.83	0.41	-2.04	0.41	0.41	0.41	0.41	0.41
chr17	19836596	19846595	4	5	5	5	5	5	2	2	2	2	2	2	4.83	0.41	-2.04	0.41	0.41	0.41	0.41	0.41
chr17	19846596	21256595	4	5	5	5	5	5	2	2	2	2	2	2	4.83	0.41	-2.04	0.41	0.41	0.41	0.41	0.41
chr17	21256596	21276995	4	5	5	5	5	5	2	2	2	2	2	2	4.83	0.41	-2.04	0.41	0.41	0.41	0.41	0.41
chr17	21276996	21286995	4	5	5	5	5	5	2	2	2	2	2	2	4.83	0.41	-2.04	0.41	0.41	0.41	0.41	0.41
chr17	21286996	21386595	4	5	5	5	5	5	2	2	2	2	2	2	4.83	0.41	-2.04	0.41	0.41	0.41	0.41	0.41
chr17	21386596	21396595	4	5	5	5	5	5	2	2	2	2	2	2	4.83	0.41	-2.04	0.41	0.41	0.41	0.41	0.41
chr17	21396596	21516995	4	5	5	5	5	5	2	2	2	2	2	2	4.83	0.41	-2.04	0.41	0.41	0.41	0.41	0.41
chr17	21516996	21777190	4	5	5	5	5	5	2	2	2	2	2	2	4.83	0.41	-2.04	0.41	0.41	0.41	0.41	0.41
chr17	21777191	21787190	4	5	5	5	5	5	2	2	2	2	2	2	4.83	0.41	-2.04	0.41	0.41	0.41	0.41	0.41
chr17	21787191	22186590	4	5	5	5	5	5	2	2	2	2	2	2	4.83	0.41	-2.04	0.41	0.41	0.41	0.41	0.41
chr17	22186591	25809985	4	5	5	5	5	5	2	2	2	2	2	2	4.83	0.41	-2.04	0.41	0.41	0.41	0.41	0.41
chr17	25809986	31289985	4	5	5	5	5	5	2	2	2	2	2	2	4.83	0.41	-2.04	0.41	0.41	0.41	0.41	0.41
chr17	31289986	33219985	4	5	5	5	5	5	2	2	2	2	2	2	4.83	0.41	-2.04	0.41	0.41	0.41	0.41	0.41
chr17	33219986	33229985	4	5	5	5	5	5	2	2	2	2	2	2	4.83	0.41	-2.04	0.41	0.41	0.41	0.41	0.41
chr17	33229986	34459985	4	5	5	5	5	5	2	2	2	2	2	2	4.83	0.41	-2.04	0.41	0.41	0.41	0.41	0.41
chr17	34459986	34469985	4	5	5	5	5	5	2	2	2	2	2	2	4.83	0.41	-2.04	0.41	0.41	0.41	0.41	0.41
chr17	34469986	34479985	4	4	5	5	5	5	2	2	2	2	2	2	4.67	0.52	-1.29	-1.29	0.65	0.65	0.65	0.65
chr17	34479986	34489985	4	4	5	5	5	5	2	2	2	2	2	2	4.67	0.52	-1.29	-1.29	0.65	0.65	0.65	0.65
chr17	34489986	34499985	4	4	5	4	4	5	2	2	2	1	2	2	4.33	0.52	-0.65	-0.65	1.29	-0.65	-0.65	1.29
chr17	34499986	34760180	4	4	4	4	4	4	2	2	2	1	2	2	4	0	0	0	0	0	0	0
chr17	34760181	34770180	4	4	4	4	4	4	2	2	2	1	2	2	4	0	0	0	0	0	0	0
chr17	34770181	34820180	4	4	4	4	4	5	2	2	2	1	2	2	4.17	0.41	-0.41	-0.41	-0.41	-0.41	-0.41	2.04
chr17	34820181	34830180	4	4	4	4	4	5	2	2	2	1	2	2	4.17	0.41	-0.41	-0.41	-0.41	-0.41	-0.41	2.04
chr17	34830181	34840180	4	4	4	4	5	5	2	2	2	1	2	2	4.33	0.52	-0.65	-0.65	-0.65	-0.65	1.29	1.29
chr17	34840181	34860180	4	5	4	5	5	5	2	2	2	2	2	2	4.67	0.52	-1.29	0.65	-1.29	0.65	0.65	0.65
chr17	34860181	36270180	4	5	5	5	5	5	2	2	2	2	2	2	4.83	0.41	-2.04	0.41	0.41	0.41	0.41	0.41
chr17	36270181	36280180	4	5	5	5	5	5	2	2	2	2	2	2	4.83	0.41	-2.04	0.41	0.41	0.41	0.41	0.41
chr17	36280181	36290180	4	5	5	5	5	5	2	2	2	2	2	2	4.83	0.41	-2.04	0.41	0.41	0.41	0.41	0.41
chr17	36290181	36400180	4	5	5	5	5	5	2	2	2	2	2	1	4.83	0.41	-2.04	0.41	0.41	0.41	0.41	0.41
chr17	36400181	36410180	4	5	5	5	5	5	2	2	2	2	2	1	4.83	0.41	-2.04	0.41	0.41	0.41	0.41	0.41
chr17	36410181	39120180	4	5	5	5	5	5	2	2	2	2	2	2	4.83	0.41	-2.04	0.41	0.41	0.41	0.41	0.41
chr17	39120181	39130180	4	5	5	5	5	5	2	2	2	2	2	2	4.83	0.41	-2.04	0.41	0.41	0.41	0.41	0.41
chr17	39130181	39980180	4	5	5	5	5	5	2	2	2	2	2	2	4.83	0.41	-2.04	0.41	0.41	0.41	0.41	0.41
chr17	39980181	39990180	4	5	5	5	5	5	2	2	2	2	2	2	4.83	0.41	-2.04	0.41	0.41	0.41	0.41	0.41
chr17	39990181	49781380	4	5	5	5	5	5	2	2	2	2	2	2	4.83	0.41	-2.04	0.41	0.41	0.41	0.41	0.41
chr17	49781381	49791380	4	5	5	5	5	5	2	2	2	2	2	2	4.83	0.41	-2.04	0.41	0.41	0.41	0.41	0.41
chr17	49791381	52941380	4	5	5	5	5	5	2	2	2	2	2	2	4.83	0.41	-2.04	0.41	0.41	0.41	0.41	0.41
chr17	52941381	52951380	4	5	5	5	5	5	2	2	2	2	2	2	4.83	0.41	-2.04	0.41	0.41	0.41	0.41	0.41
chr17	52951381	67643580	4	5	5	5	5	5	2	2	2	2	2	2	4.83	0.41	-2.04	0.41	0.41	0.41	0.41	0.41
chr17	67643581	67653580	4	5	5	5	5	5	2	2	2	2	2	2	4.83	0.41	-2.04	0.41	0.41	0.41	0.41	0.41
chr17	67653581	70653580	4	5	5	5	5	5	2	2	2	2	2	2	4.83	0.41	-2.04	0.41	0.41	0.41	0.41	0.41
chr17	70653581	70663580	4	5	5	5	5	5	2	2	2	2	2	2	4.83	0.41	-2.04	0.41	0.41	0.41	0.41	0.41
chr17	70663581	77073580	4	5	5	5	5	5	2	2	2	2	2	2	4.83	0.41	-2.04	0.41	0.41	0.41	0.41	0.41
chr17	77073581	77083580	4	5	5	5	5	5	2	2	2	2	2	2	4.83	0.41	-2.04	0.41	0.41	0.41	0.41	0.41
chr17	77083581	77683575	4	5	5	5	5	5	2	2	2	2	2	2	4.83	0.41	-2.04	0.41	0.41	0.41	0.41	0.41
chr17	77683576	77693575	4	5	5	5	5	5	2	2	2	2	2	2	4.83	0.41	-2.04	0.41	0.41	0.41	0.41	0.41
chr17	77693576	77733775	4	5	5	5	5	5	2	2	2	2	2	2	4.83	0.41	-2.04	0.41	0.41	0.41	0.41	0.41
chr17	77733776	77743775	4	5	5	5	5	5	2	2	2	2	2	2	4.83	0.41	-2.04	0.41	0.41	0.41	0.41	0.41
chr17	77743776	79514175	4	5	5	5	5	5	2	2	2	2	2	2	4.83	0.41	-2.04	0.41	0.41	0.41	0.41	0.41
chr17	79514176	79524175	4	5	5	5	5	5	2	2	2	2	2	2	4.83	0.41	-2.04	0.41	0.41	0.41	0.41	0.41
chr17	79524176	79534175	4	5	6	5	5	5	2	2	3	2	2	2	5	0.63	-1.58	0	1.58	0	0	0
chr17	79534176	79583975	4	5	6	6	5	5	2	2	3	3	2	2	5.17	0.75	-1.55	-0.22	1.11	1.11	-0.22	-0.22
chr17	79583976	79593975	4	5	6	6	5	5	2	2	3	3	2	2	5.17	0.75	-1.55	-0.22	1.11	1.11	-0.22	-0.22
chr17	79593976	80024370	4	6	6	6	7	6	2	3	3	3	3	3	5.83	0.98	-1.86	0.17	0.17	0.17	1.19	0.17
chr17	80024371	80034370	4	5	6	6	7	6	2	2	3	3	3	3	5.67	1.03	-1.61	-0.65	0.32	0.32	1.29	0.32

chr17	80034371	81184170	4	5	5	5	5	5	2	2	2	2	2	2	4.83	0.41	-2.04	0.41	0.41	0.41	0.41	0.41
chr18	19801	15381400	4	3	3	3	3	3	2	1	1	1	1	1	3.17	0.41	2.04	-0.41	-0.41	-0.41	-0.41	-0.41
chr18	15381401	19009995	4	3	3	3	3	3	2	1	1	1	1	1	3.17	0.41	2.04	-0.41	-0.41	-0.41	-0.41	-0.41
chr18	19009996	27010995	4	3	3	3	3	3	2	1	1	1	1	1	3.17	0.41	2.04	-0.41	-0.41	-0.41	-0.41	-0.41
chr18	27010996	27020995	4	3	3	3	3	3	2	1	1	1	1	1	3.17	0.41	2.04	-0.41	-0.41	-0.41	-0.41	-0.41
chr18	27020996	27191195	4	3	3	3	3	3	2	1	1	1	1	1	3.17	0.41	2.04	-0.41	-0.41	-0.41	-0.41	-0.41
chr18	27191196	27201195	4	3	3	3	3	3	2	1	1	1	1	1	3.17	0.41	2.04	-0.41	-0.41	-0.41	-0.41	-0.41
chr18	27201196	44624595	4	3	3	3	3	3	2	1	1	1	1	1	3.17	0.41	2.04	-0.41	-0.41	-0.41	-0.41	-0.41
chr18	44624596	44634595	4	3	3	3	3	3	2	1	1	1	1	1	3.17	0.41	2.04	-0.41	-0.41	-0.41	-0.41	-0.41
chr18	44634596	44744595	4	3	3	3	3	3	2	1	1	1	1	1	3.17	0.41	2.04	-0.41	-0.41	-0.41	-0.41	-0.41
chr18	44744596	44754595	4	3	3	3	3	3	2	1	1	1	1	1	3.17	0.41	2.04	-0.41	-0.41	-0.41	-0.41	-0.41
chr18	44754596	56276790	4	3	3	3	3	3	2	1	1	1	1	1	3.17	0.41	2.04	-0.41	-0.41	-0.41	-0.41	-0.41
chr18	56276791	56696790	4	3	3	3	3	3	2	1	1	1	1	1	3.17	0.41	2.04	-0.41	-0.41	-0.41	-0.41	-0.41
chr18	56696791	56706790	4	3	3	3	3	3	2	1	1	1	1	1	3.17	0.41	2.04	-0.41	-0.41	-0.41	-0.41	-0.41
chr18	56706791	67815390	4	3	3	3	3	3	2	1	1	1	1	1	3.17	0.41	2.04	-0.41	-0.41	-0.41	-0.41	-0.41
chr18	67815391	67825390	4	3	3	3	3	3	2	1	1	1	1	1	3.17	0.41	2.04	-0.41	-0.41	-0.41	-0.41	-0.41
chr18	67825391	68135390	4	3	3	3	3	3	2	1	1	1	1	1	3.17	0.41	2.04	-0.41	-0.41	-0.41	-0.41	-0.41
chr18	68135391	68145390	4	3	3	3	3	3	2	1	1	1	1	1	3.17	0.41	2.04	-0.41	-0.41	-0.41	-0.41	-0.41
chr18	68145391	68825390	4	3	3	3	3	3	2	1	1	1	1	1	3.17	0.41	2.04	-0.41	-0.41	-0.41	-0.41	-0.41
chr18	68825391	68835390	4	3	3	3	3	3	2	1	1	1	1	1	3.17	0.41	2.04	-0.41	-0.41	-0.41	-0.41	-0.41
chr18	68835391	69285390	4	3	3	3	3	3	2	1	1	1	1	1	3.17	0.41	2.04	-0.41	-0.41	-0.41	-0.41	-0.41
chr18	69285391	69295390	4	3	3	3	3	3	2	1	1	1	1	1	3.17	0.41	2.04	-0.41	-0.41	-0.41	-0.41	-0.41
chr18	69295391	74535585	4	3	3	3	3	3	2	1	1	1	1	1	3.17	0.41	2.04	-0.41	-0.41	-0.41	-0.41	-0.41
chr18	74535586	74545585	4	3	3	3	3	3	2	1	1	1	1	1	3.17	0.41	2.04	-0.41	-0.41	-0.41	-0.41	-0.41
chr18	74545586	74855785	4	3	3	3	3	3	2	1	1	1	1	1	3.17	0.41	2.04	-0.41	-0.41	-0.41	-0.41	-0.41
chr18	74855786	74865785	4	3	3	3	3	3	2	1	1	1	1	1	3.17	0.41	2.04	-0.41	-0.41	-0.41	-0.41	-0.41
chr18	74865786	75065785	4	3	3	3	3	3	2	1	1	1	1	1	3.17	0.41	2.04	-0.41	-0.41	-0.41	-0.41	-0.41
chr18	75065786	75075785	4	3	3	3	3	3	2	1	1	1	1	1	3.17	0.41	2.04	-0.41	-0.41	-0.41	-0.41	-0.41
chr18	75075786	76786785	4	3	3	3	3	3	2	1	1	1	1	1	3.17	0.41	2.04	-0.41	-0.41	-0.41	-0.41	-0.41
chr18	76786786	76796785	4	3	3	3	3	3	2	1	1	1	1	1	3.17	0.41	2.04	-0.41	-0.41	-0.41	-0.41	-0.41
chr18	76796786	78006785	4	3	3	3	3	3	2	1	1	1	1	1	3.17	0.41	2.04	-0.41	-0.41	-0.41	-0.41	-0.41
chr19	69801	190000	3	4	3	4	4	4	1	2	0	2	2	2	3.67	0.52	-1.29	0.65	-1.29	0.65	0.65	0.65
chr19	190001	200000	3	4	3	4	4	4	1	2	0	2	2	2	3.67	0.52	-1.29	0.65	-1.29	0.65	0.65	0.65
chr19	200001	219800	3	4	3	4	3	4	1	2	0	2	0	2	3.5	0.55	-0.91	0.91	-0.91	0.91	-0.91	0.91
chr19	219801	439800	3	3	3	3	3	3	1	1	0	0	0	0	3	0	0	0	0	0	0	0
chr19	439801	449800	3	3	3	3	3	3	1	1	0	0	0	0	3	0	0	0	0	0	0	0
chr19	449801	470000	3	4	4	3	5	4	1	1	0	0	0	0	3.83	0.75	-1.11	0.22	0.22	-1.11	1.55	0.22
chr19	470001	480000	3	4	4	3	5	4	1	1	0	0	0	0	3.83	0.75	-1.11	0.22	0.22	-1.11	1.55	0.22
chr19	480001	870000	3	4	4	4	5	4	1	1	0	0	0	0	4	0.63	-1.58	0	0	0	1.58	0
chr19	870001	880000	3	4	4	4	5	4	1	1	0	0	0	0	4	0.63	-1.58	0	0	0	1.58	0
chr19	880001	6981400	3	3	3	3	3	3	1	1	0	0	0	0	3	0	0	0	0	0	0	0
chr19	6981401	6991400	3	3	3	3	3	3	1	1	0	0	0	0	3	0	0	0	0	0	0	0
chr19	6991401	7071400	3	3	3	3	3	3	1	1	0	0	0	0	3	0	0	0	0	0	0	0
chr19	7071401	7081400	3	3	3	3	3	3	1	1	0	0	0	0	3	0	0	0	0	0	0	0
chr19	7081401	7290800	3	3	3	3	3	3	1	1	0	0	0	0	3	0	0	0	0	0	0	0
chr19	7290801	7300800	3	3	3	3	3	3	1	1	0	0	0	0	3	0	0	0	0	0	0	0
chr19	7300801	7311000	3	3	5	3	5	5	1	1	1	0	1	1	4	1.1	-0.91	-0.91	0.91	-0.91	0.91	0.91
chr19	7311001	7340800	4	5	5	6	5	5	2	2	1	3	1	1	5	0.63	-1.58	0	0	1.58	0	0
chr19	7340801	7401195	4	5	5	6	5	5	2	2	1	3	1	1	5	0.63	-1.58	0	0	1.58	0	0
chr19	7401196	7411395	4	5	3	6	3	3	2	2	0	3	0	0	4	1.26	0	0.79	-0.79	1.58	-0.79	-0.79
chr19	7411396	8841390	3	3	3	3	3	3	1	1	0	0	0	0	3	0	0	0	0	0	0	0
chr19	8841391	8851390	3	3	3	3	3	3	1	1	0	0	0	0	3	0	0	0	0	0	0	0
chr19	8851391	8861990	2	2	2	2	2	3	1	0	0	0	1	0	2.17	0.41	-0.41	-0.41	-0.41	-0.41	-0.41	2.04
chr19	8861991	8891990	2	2	2	2	2	2	1	0	0	0	1	0	2	0	0	0	0	0	0	0
chr19	8891991	8901990	2	2	2	2	2	2	1	0	0	0	1	0	2	0	0	0	0	0	0	0
chr19	8901991	8911990	3	3	3	3	3	2	0	1	0	0	0	0	2.83	0.41	0.41	0.41	0.41	0.41	0.41	-2.04
chr19	8911991	9391990	3	3	3	3	3	3	0	1	0	0	0	0	3	0	0	0	0	0	0	0
chr19	9391991	9401990	3	3	3	3	3	3	0	1	0	0	0	0	3	0	0	0	0	0	0	0
chr19	9401991	17664790	3	3	3	3	3	3	0	1	0	0	0	0	3	0	0	0	0	0	0	0
chr19	17664791	17674790	3	3	3	3	3	3	0	1	0	0	0	0	3	0	0	0	0	0	0	0
chr19	17674791	17864390	3	3	3	3	3	3	0	1	0	0	0	0	3	0	0	0	0	0	0	0
chr19	17864391	17874390	3	3	3	3	3	3	0	1	0	0	0	0	3	0	0	0	0	0	0	0
chr19	17874391	18054790	4	4	3	3	4	4	1	1	0	0	1	1	3.67	0.52	0.65	0.65	-1.29	-1.29	0.65	0.65
chr19	18054791	18064790	4	4	3	4	4	4	1	1	0	1	1	1	3.83	0.41	0.41	0.41	-2.04	0.41	0.41	0.41
chr19	18064791	18654390	4	4	3	4	4	4	1	1	0	1	1	1	3.83	0.41	0.41	0.41	-2.04	0.41	0.41	0.41
chr19	18654391	18664390	4	4	3	4	4	4	1	1	0	1	1	1	3.83	0.41	0.41	0.41	-2.04	0.41	0.41	0.41
chr19	18664391	19684390	4	4	3	5	4	5	1	1	0	2	1	2	4.17	0.75	-0.22	-0.22	-1.55	1.11	-0.22	1.11
chr19	19684391	19694390	4	4	3	5	4	5	1	1	0	2	1	2	4.17	0.75	-0.22	-0.22	-1.55	1.11	-0.22	1.11
chr19	19694391	20195190	4	4	3	3	4	3	1	1	0	0	1	0	3.5	0.55	0.91	0.91	-0.91	-0.91	0.91	-0.91
chr19	20195191	20205190	4	4	3	3	4	3	1	1	0	0	1	0	3.5	0.55	0.91	0.91	-0.91	-0.91	0.91	-0.91
chr19	20205191	20384990	4	4	3	3	4	3	1	1	0	0	1	0	3.5	0.55	0.91	0.91	-0.91	-0.91	0.91	-0.91
chr19	20384991	20394990	4	4	3	3	4	3	1	1	0	0	1	0	3.5	0.55	0.91	0.91	-0.91	-0.91	0.91	-0.91
chr19	20394991	24384790	4	4	3	4	4	4	1	1	0	1	1	1	3.83	0.41	0.41	0.41	-2.04	0.41	0.41	0.41
chr19	24384791	28609985	4	4	3	4	4	4	1	1	0	1	1	1	3.83	0.41	0.41	0.41	-2.04	0.41	0.41	0.41
chr19	28609986	32649985	5	4	5	4	5	5	2	2	2	2	2	2	4.67	0.52	0.65	-1.29	0.65	-1.29	0.65	0.65
chr19	32649986	32770185	5	4	5	5	5	5	2	2	2	2	2	2	4.83	0.41	0.41	-2.04	0.41	0.41	0.41	0.41

chr19	32770186	33060185	5	4	5	5	5	5	2	2	2	2	2	2	4.83	0.41	0.41	-2.04	0.41	0.41	0.41	0.41
chr19	33060186	33070185	5	4	5	5	5	5	2	2	2	2	2	2	4.83	0.41	0.41	-2.04	0.41	0.41	0.41	0.41
chr19	33070186	34020185	5	4	5	5	5	5	2	2	2	2	2	2	4.83	0.41	0.41	-2.04	0.41	0.41	0.41	0.41
chr19	34020186	34030185	5	4	5	5	5	5	2	2	2	2	2	2	4.83	0.41	0.41	-2.04	0.41	0.41	0.41	0.41
chr19	34030186	34450185	5	4	5	5	5	5	2	2	2	2	2	2	4.83	0.41	0.41	-2.04	0.41	0.41	0.41	0.41
chr19	34450186	34460185	5	4	5	5	5	5	2	2	2	2	2	2	4.83	0.41	0.41	-2.04	0.41	0.41	0.41	0.41
chr19	34460186	34980185	5	4	5	5	5	5	2	2	2	2	2	2	4.83	0.41	0.41	-2.04	0.41	0.41	0.41	0.41
chr19	34980186	34990185	5	4	5	5	5	5	2	2	2	2	2	2	4.83	0.41	0.41	-2.04	0.41	0.41	0.41	0.41
chr19	34990186	35050185	5	4	5	5	5	5	2	2	2	2	2	2	4.83	0.41	0.41	-2.04	0.41	0.41	0.41	0.41
chr19	35050186	35060185	5	4	5	5	5	5	2	2	2	2	2	2	4.83	0.41	0.41	-2.04	0.41	0.41	0.41	0.41
chr19	35060186	36939985	5	4	5	5	5	5	2	2	2	2	2	2	4.83	0.41	0.41	-2.04	0.41	0.41	0.41	0.41
chr19	36939986	37339985	5	5	5	5	5	5	2	2	2	2	2	2	5	0	0	0	0	0	0	0
chr19	37339986	37349985	5	5	5	5	5	5	2	2	2	2	2	2	5	0	0	0	0	0	0	0
chr19	37349986	38310185	5	5	5	5	5	5	2	2	2	2	2	2	5	0	0	0	0	0	0	0
chr19	38310186	38320185	5	5	5	5	5	5	2	2	2	2	2	2	5	0	0	0	0	0	0	0
chr19	38320186	38380185	5	5	5	5	5	5	2	2	2	2	2	2	5	0	0	0	0	0	0	0
chr19	38380186	38390185	5	5	5	5	5	5	2	2	2	2	2	2	5	0	0	0	0	0	0	0
chr19	38390186	41930185	5	5	5	5	5	5	2	2	2	2	2	2	5	0	0	0	0	0	0	0
chr19	41930186	41940185	5	5	5	5	5	5	2	2	2	2	2	2	5	0	0	0	0	0	0	0
chr19	41940186	42320185	5	5	5	5	5	5	2	2	2	2	2	2	5	0	0	0	0	0	0	0
chr19	42320186	42330185	5	5	5	5	5	5	2	2	2	2	2	2	5	0	0	0	0	0	0	0
chr19	42330186	43050185	5	5	5	5	5	5	2	2	2	2	2	2	5	0	0	0	0	0	0	0
chr19	43050186	43060185	5	5	5	5	5	5	2	2	2	2	2	2	5	0	0	0	0	0	0	0
chr19	43060186	43220385	5	5	5	5	5	5	2	2	2	2	2	2	5	0	0	0	0	0	0	0
chr19	43220386	43410185	5	5	5	5	5	5	2	2	2	2	2	2	5	0	0	0	0	0	0	0
chr19	43410186	43420185	5	5	5	5	5	5	2	2	2	2	2	2	5	0	0	0	0	0	0	0
chr19	43420186	43449985	5	5	5	5	5	5	2	2	2	2	2	2	5	0	0	0	0	0	0	0
chr19	43449986	43459985	5	5	5	5	5	5	2	2	2	2	2	2	5	0	0	0	0	0	0	0
chr19	43459986	43629985	5	4	4	4	5	5	2	2	1	1	2	2	4.5	0.55	0.91	-0.91	-0.91	-0.91	0.91	0.91
chr19	43629986	43639985	5	4	4	4	5	5	2	2	1	1	2	2	4.5	0.55	0.91	-0.91	-0.91	-0.91	0.91	0.91
chr19	43639986	43690385	5	5	4	5	5	5	2	2	1	2	2	2	4.83	0.41	0.41	0.41	-2.04	0.41	0.41	0.41
chr19	43690386	43700385	5	5	4	5	5	5	2	2	1	2	2	2	4.83	0.41	0.41	0.41	-2.04	0.41	0.41	0.41
chr19	43700386	43720185	6	5	6	5	5	5	1	2	0	2	2	2	5.33	0.52	1.29	-0.65	1.29	-0.65	-0.65	-0.65
chr19	43720186	43760185	6	5	6	5	5	6	1	2	0	2	2	1	5.5	0.55	0.91	-0.91	0.91	-0.91	-0.91	0.91
chr19	43760186	43770185	6	5	6	4	5	6	1	2	0	1	2	1	5.33	0.82	0.82	-0.41	0.82	-1.63	-0.41	0.82
chr19	43770186	43929985	5	5	5	4	5	5	2	2	2	1	2	2	4.83	0.41	0.41	0.41	0.41	-2.04	0.41	0.41
chr19	43929986	43939985	5	5	5	4	5	5	2	2	2	1	2	2	4.83	0.41	0.41	0.41	0.41	-2.04	0.41	0.41
chr19	43939986	59101185	5	5	5	5	5	5	2	2	2	2	2	2	5	0	0	0	0	0	0	0
chr20	69801	1541200	4	5	5	5	5	5	2	2	2	2	2	2	4.83	0.41	-2.04	0.41	0.41	0.41	0.41	0.41
chr20	1541201	1551200	4	5	5	5	5	5	2	2	2	2	2	2	4.83	0.41	-2.04	0.41	0.41	0.41	0.41	0.41
chr20	1551201	1591200	4	4	5	5	4	5	2	2	2	2	2	2	4.5	0.55	-0.91	-0.91	0.91	0.91	-0.91	0.91
chr20	1591201	1601200	4	4	5	5	4	5	2	2	2	2	2	2	4.5	0.55	-0.91	-0.91	0.91	0.91	-0.91	0.91
chr20	1601201	17491400	4	5	5	5	5	5	2	2	2	2	2	2	4.83	0.41	-2.04	0.41	0.41	0.41	0.41	0.41
chr20	17491401	17501400	4	5	5	5	5	5	2	2	2	2	2	2	4.83	0.41	-2.04	0.41	0.41	0.41	0.41	0.41
chr20	17501401	17981400	4	5	5	5	5	5	2	2	2	2	2	2	4.83	0.41	-2.04	0.41	0.41	0.41	0.41	0.41
chr20	17981401	17991400	4	5	5	5	5	5	2	2	2	2	2	2	4.83	0.41	-2.04	0.41	0.41	0.41	0.41	0.41
chr20	17991401	22433800	4	5	5	5	5	5	2	2	2	2	2	2	4.83	0.41	-2.04	0.41	0.41	0.41	0.41	0.41
chr20	22433801	22443800	4	5	5	5	5	5	2	2	2	2	2	2	4.83	0.41	-2.04	0.41	0.41	0.41	0.41	0.41
chr20	22443801	22533800	4	5	5	5	6	5	2	2	2	2	2	2	5	0.63	-1.58	0	0	0	1.58	0
chr20	22533801	22543800	4	5	5	5	6	5	2	2	2	2	2	2	5	0.63	-1.58	0	0	0	1.58	0
chr20	22543801	25584400	4	5	5	5	5	5	2	2	2	2	2	2	4.83	0.41	-2.04	0.41	0.41	0.41	0.41	0.41
chr20	25584401	29429595	-	-	5		5	5	-	-	2	-	2	2	-	-	-	-	-	-	-	-
chr20	29429596	29649595	4	5	5	4	5	5	2	2	2	1	2	2	4.67	0.52	-1.29	0.65	0.65	-1.29	0.65	0.65
chr20	29649596	57402390	4	5	5	5	5	5	2	2	2	2	2	2	4.83	0.41	-2.04	0.41	0.41	0.41	0.41	0.41
chr20	57402391	57652390	4	5	5	5	5	5	2	2	2	2	2	2	4.83	0.41	-2.04	0.41	0.41	0.41	0.41	0.41
chr20	57652391	57662390	4	5	5	5	5	5	2	2	2	2	2	2	4.83	0.41	-2.04	0.41	0.41	0.41	0.41	0.41
chr20	57662391	62952980	4	5	5	5	5	5	2	2	2	2	2	2	4.83	0.41	-2.04	0.41	0.41	0.41	0.41	0.41
chr21	9420991	9841380	4	5	5	5	5	5	2	2	1	1	2	1	4.83	0.41	-2.04	0.41	0.41	0.41	0.41	0.41
chr21	9841381	9851380	4	5	5	5	5	5	2	2	1	1	2	1	4.83	0.41	-2.04	0.41	0.41	0.41	0.41	0.41
chr21	9851381	9991380	4	5	5	5	4	5	2	2	1	1	1	2	4.67	0.52	-1.29	0.65	0.65	0.65	-1.29	0.65
chr21	9991381	10371775	3	4	4	4	4	5	1	2	0	1	1	2	4	0.63	-1.58	0	0	0	0	1.58
chr21	10371776	10881970	3	4	4	4	4	4	1	2	0	1	1	1	3.83	0.41	-2.04	0.41	0.41	0.41	0.41	0.41
chr21	10881971	14348165	3	4	4	4	4	4	1	2	0	1	1	1	3.83	0.41	-2.04	0.41	0.41	0.41	0.41	0.41
chr21	14348166	15031165	4	4	4	4	4	4	2	2	2	2	2	2	4	0	0	0	0	0	0	0
chr21	15031166	15072565	5	4	4	4	4	4	2	2	2	2	2	2	4.17	0.41	2.04	-0.41	-0.41	-0.41	-0.41	-0.41
chr21	15072566	15082565	5	4	4	4	4	4	2	2	2	2	2	2	4.17	0.41	2.04	-0.41	-0.41	-0.41	-0.41	-0.41
chr21	15082566	30254765	4	4	4	4	4	4	2	2	2	2	2	2	4	0	0	0	0	0	0	0
chr21	30254766	30314765	4	4	4	4	4	4	2	2	2	2	2	2	4	0	0	0	0	0	0	0
chr21	30314766	30324765	4	4	5	4	4	4	2	2	2	2	2	2	4.17	0.41	-0.41	-0.41	2.04	-0.41	-0.41	-0.41
chr21	30324766	30594365	4	4	5	4	4	4	2	2	2	2	2	2	4.17	0.41	-0.41	-0.41	2.04	-0.41	-0.41	-0.41
chr21	30594366	30604365	4	4	5	4	4	4	2	2	2	2	2	2	4.17	0.41	-0.41	-0.41	2.04	-0.41	-0.41	-0.41
chr21	30604366	30644765	4	4	4	4	4	4	2	2	2	2	2	2	4	0	0	0	0	0	0	0
chr21	30644766	30654765	4	4	4	4	4	4	2	2	2	2	2	2	4	0	0	0	0	0	0	0
chr21	30654766	30864765	4	4	4	4	4	4	2	2	2	2	2	2	4	0	0	0	0	0	0	0
chr21	30864766	30874765	4	4	4	4	4	4	2	2	2	2	2	2	4	0	0	0	0	0	0	0
chr21	30874766	32844765	4	4	4	4	4	4	2	2	2	2	2	2	4	0	0	0	0	0	0	0

chr21	32844766	32854765	4	4	4	4	4	4	2	2	2	2	2	2	4	0	0	0	0	0	0	0
chr21	32854766	48102590	4	4	4	4	4	4	2	2	2	2	2	2	4	0	0	0	0	0	0	0
chr22	17909801	18649800	5	7	7	7	8	7	2	3	3	3	3	3	6.83	0.98	-1.86	0.17	0.17	0.17	1.19	0.17
chr22	18649801	18659800	5	7	7	7	8	7	2	3	3	3	3	3	6.83	0.98	-1.86	0.17	0.17	0.17	1.19	0.17
chr22	18659801	18689800	5	7	7	7	7	7	2	3	3	3	2	3	6.67	0.82	-2.04	0.41	0.41	0.41	0.41	0.41
chr22	18689801	18709800	5	7	7	7	7	7	2	3	3	3	2	2	6.67	0.82	-2.04	0.41	0.41	0.41	0.41	0.41
chr22	18709801	18899800	5	7	7	7	7	7	2	3	3	3	2	2	6.67	0.82	-2.04	0.41	0.41	0.41	0.41	0.41
chr22	18899801	18909800	5	7	7	7	7	7	2	3	3	3	2	2	6.67	0.82	-2.04	0.41	0.41	0.41	0.41	0.41
chr22	18909801	18979800	5	7	7	7	7	7	2	3	3	3	3	2	6.67	0.82	-2.04	0.41	0.41	0.41	0.41	0.41
chr22	18979801	18989800	5	7	7	7	7	7	2	3	3	3	3	2	6.67	0.82	-2.04	0.41	0.41	0.41	0.41	0.41
chr22	18989801	20482400	5	7	7	7	7	7	2	3	3	3	3	3	6.67	0.82	-2.04	0.41	0.41	0.41	0.41	0.41
chr22	20482401	20492400	5	7	7	7	7	7	2	3	3	3	3	3	6.67	0.82	-2.04	0.41	0.41	0.41	0.41	0.41
chr22	20492401	20712595	5	7	7	7	6	7	2	3	3	3	2	3	6.5	0.84	-1.79	0.6	0.6	0.6	-0.6	0.6
chr22	20712596	20722595	5	7	7	7	6	7	2	3	3	3	2	3	6.5	0.84	-1.79	0.6	0.6	0.6	-0.6	0.6
chr22	20722596	21463395	5	7	7	7	8	7	2	3	3	3	3	3	6.83	0.98	-1.86	0.17	0.17	0.17	1.19	0.17
chr22	21463396	21473395	5	7	7	7	8	7	2	3	3	3	3	3	6.83	0.98	-1.86	0.17	0.17	0.17	1.19	0.17
chr22	21473396	21502595	5	7	7	7	8	7	2	3	3	3	3	3	6.83	0.98	-1.86	0.17	0.17	0.17	1.19	0.17
chr22	21502596	21512595	5	7	7	7	8	7	2	3	3	3	3	3	6.83	0.98	-1.86	0.17	0.17	0.17	1.19	0.17
chr22	21512596	21732595	5	7	7	7	7	7	2	3	3	3	2	3	6.67	0.82	-2.04	0.41	0.41	0.41	0.41	0.41
chr22	21732596	21742595	5	7	7	7	7	7	2	3	3	3	2	3	6.67	0.82	-2.04	0.41	0.41	0.41	0.41	0.41
chr22	21742596	21753395	5	7	7	7	7	7	2	3	3	3	3	3	6.67	0.82	-2.04	0.41	0.41	0.41	0.41	0.41
chr22	21753396	29233595	5	7	7	7	7	7	2	3	3	3	3	3	6.67	0.82	-2.04	0.41	0.41	0.41	0.41	0.41
chr22	29233596	29243595	5	7	7	7	7	7	2	3	3	3	3	3	6.67	0.82	-2.04	0.41	0.41	0.41	0.41	0.41
chr22	29243596	29523595	5	8	8	8	8	8	2	4	4	4	4	4	7.5	1.22	-2.04	0.41	0.41	0.41	0.41	0.41
chr22	29523596	29533595	5	8	8	8	8	8	2	4	4	4	4	4	7.5	1.22	-2.04	0.41	0.41	0.41	0.41	0.41
chr22	29533596	29544195	5	7	8	7	7	7	2	3	4	3	3	3	6.83	0.98	-1.86	0.17	1.19	0.17	0.17	0.17
chr22	29544196	29554195	5	7	8	7	7	7	2	3	4	3	3	3	6.83	0.98	-1.86	0.17	1.19	0.17	0.17	0.17
chr22	29554196	31553595	5	7	7	7	7	7	2	3	3	3	3	3	6.67	0.82	-2.04	0.41	0.41	0.41	0.41	0.41
chr22	31553596	31563595	5	7	7	7	7	7	2	3	3	3	3	3	6.67	0.82	-2.04	0.41	0.41	0.41	0.41	0.41
chr22	31563596	31933595	5	7	7	7	8	7	2	3	3	3	4	3	6.83	0.98	-1.86	0.17	0.17	0.17	1.19	0.17
chr22	31933596	31943595	5	7	7	7	8	7	2	3	3	3	4	3	6.83	0.98	-1.86	0.17	0.17	0.17	1.19	0.17
chr22	31943596	32483595	5	7	7	7	7	7	2	3	3	3	3	3	6.67	0.82	-2.04	0.41	0.41	0.41	0.41	0.41
chr22	32483596	32493595	5	7	7	7	7	7	2	3	3	3	3	3	6.67	0.82	-2.04	0.41	0.41	0.41	0.41	0.41
chr22	32493596	32753595	5	7	7	7	7	7	2	3	3	3	3	3	6.67	0.82	-2.04	0.41	0.41	0.41	0.41	0.41
chr22	32753596	32763595	5	7	7	7	7	7	2	3	3	3	3	3	6.67	0.82	-2.04	0.41	0.41	0.41	0.41	0.41
chr22	32763596	34543595	5	7	7	7	7	7	2	3	3	3	3	3	6.67	0.82	-2.04	0.41	0.41	0.41	0.41	0.41
chr22	34543596	34553595	5	7	7	7	7	7	2	3	3	3	3	3	6.67	0.82	-2.04	0.41	0.41	0.41	0.41	0.41
chr22	34553596	35503595	5	7	7	7	7	7	2	3	3	3	3	3	6.67	0.82	-2.04	0.41	0.41	0.41	0.41	0.41
chr22	35503596	35513595	5	7	7	7	7	7	2	3	3	3	3	3	6.67	0.82	-2.04	0.41	0.41	0.41	0.41	0.41
chr22	35513596	47497395	5	7	7	7	7	7	2	3	3	3	3	3	6.67	0.82	-2.04	0.41	0.41	0.41	0.41	0.41
chr22	47497396	47507395	5	7	7	7	7	7	2	3	3	3	3	3	6.67	0.82	-2.04	0.41	0.41	0.41	0.41	0.41
chr22	47507396	49877395	5	7	7	7	7	7	2	3	3	3	3	3	6.67	0.82	-2.04	0.41	0.41	0.41	0.41	0.41
chr22	49877396	49887395	5	7	7	7	7	7	2	3	3	3	3	3	6.67	0.82	-2.04	0.41	0.41	0.41	0.41	0.41
chr22	49887396	51227590	5	7	7	7	8	7	2	3	3	3	4	3	6.83	0.98	-1.86	0.17	0.17	0.17	1.19	0.17
chrX	69801	161195	3	3	4	4	3	4	1	1	2	2	1	2	3.5	0.55	-0.91	-0.91	0.91	0.91	-0.91	0.91
chrX	161196	1364775	3	3	3	4	3	4	1	1	1	2	1	2	3.33	0.52	-0.65	-0.65	-0.65	1.29	-0.65	1.29
chrX	1364776	1374775	3	3	3	4	3	4	1	1	1	2	1	2	3.33	0.52	-0.65	-0.65	-0.65	1.29	-0.65	1.29
chrX	1374776	2687570	3	3	3	4	3	4	1	1	1	2	1	2	3.33	0.52	-0.65	-0.65	-0.65	1.29	-0.65	1.29
chrX	2687571	2697570	3	3	3	4	3	6	1	1	1	2	1	1	3.67	1.21	-0.55	-0.55	-0.55	0.28	-0.55	1.93
chrX	2697571	2709170	3	4	4	6	4	6	1	2	1	0	1	1	4.5	1.22	-1.22	-0.41	-0.41	1.22	-0.41	1.22
chrX	2709171	30644960	2	4	4	6	4	6	1	2	1	0	1	1	4.33	1.51	-1.55	-0.22	-0.22	1.11	-0.22	1.11
chrX	30644961	30654960	2	4	4	6	4	6	1	2	1	0	1	1	4.33	1.51	-1.55	-0.22	-0.22	1.11	-0.22	1.11
chrX	30654961	48351355	2	4	4	4	4	4	1	2	1	1	1	2	3.67	0.82	-2.04	0.41	0.41	0.41	0.41	0.41
chrX	48351356	48361355	2	4	4	4	4	4	1	2	1	1	1	2	3.67	0.82	-2.04	0.41	0.41	0.41	0.41	0.41
chrX	48361356	49212155	2	4	4	4	4	4	1	2	1	1	2	2	3.67	0.82	-2.04	0.41	0.41	0.41	0.41	0.41
chrX	49212156	49222155	2	4	4	4	4	4	1	2	1	1	2	2	3.67	0.82	-2.04	0.41	0.41	0.41	0.41	0.41
chrX	49222156	53124140	2	4	4	4	4	4	1	2	1	1	2	2	3.67	0.82	-2.04	0.41	0.41	0.41	0.41	0.41
chrX	53124141	53134140	2	4	4	4	4	4	1	2	1	1	2	2	3.67	0.82	-2.04	0.41	0.41	0.41	0.41	0.41
chrX	53134141	53474140	2	4	4	4	4	4	1	2	1	1	0	2	3.67	0.82	-2.04	0.41	0.41	0.41	0.41	0.41
chrX	53474141	53484140	2	4	4	4	4	4	1	2	1	1	0	2	3.67	0.82	-2.04	0.41	0.41	0.41	0.41	0.41
chrX	53484141	58081540	2	4	4	4	4	4	1	2	1	1	1	2	3.67	0.82	-2.04	0.41	0.41	0.41	0.41	0.41
chrX	58081541	63009935	2	4	4	4	4	4	1	2	1	1	1	2	3.67	0.82	-2.04	0.41	0.41	0.41	0.41	0.41
chrX	63009936	70350935	2	4	4	4	4	4	1	1	0	0	1	1	3.67	0.82	-2.04	0.41	0.41	0.41	0.41	0.41
chrX	70350936	70360935	2	4	4	4	4	4	1	1	0	0	1	1	3.67	0.82	-2.04	0.41	0.41	0.41	0.41	0.41
chrX	70360936	70870935	2	4	4	4	4	4	1	1	0	0	1	1	3.67	0.82	-2.04	0.41	0.41	0.41	0.41	0.41
chrX	70870936	70880935	2	4	4	4	4	4	1	1	0	0	1	1	3.67	0.82	-2.04	0.41	0.41	0.41	0.41	0.41
chrX	70880936	143161725	2	4	4	4	4	4	1	1	0	0	1	1	3.67	0.82	-2.04	0.41	0.41	0.41	0.41	0.41
chrX	143161726	143331925	3	4	4	4	4	4	1	1	0	0	1	1	3.83	0.41	-2.04	0.41	0.41	0.41	0.41	0.41
chrX	143331926	143341925	3	4	4	4	4	4	1	1	0	0	1	1	3.83	0.41	-2.04	0.41	0.41	0.41	0.41	0.41
chrX	143341926	154903905	2	4	4	4	4	4	1	1	0	0	1	1	3.67	0.82	-2.04	0.41	0.41	0.41	0.41	0.41
chrX	154903906	154923505	2	5	4	4	4	4	1	2	0	0	1	1	3.83	0.98	-1.86	1.19	0.17	0.17	0.17	0.17
chrX	154923506	154933505	2	5	4	4	4	4	1	2	0	0	1	1	3.83	0.98	-1.86	1.19	0.17	0.17	0.17	0.17
chrX	154933506	155243505	2	5	4	4	5	5	1	2	0	0	2	2	4.17	1.17	-1.85	0.71	-0.14	-0.14	0.71	0.71
chrY	19801	381190	3	3	3	4	3	4	1	1	1	2	1	2	3.33	0.52	-0.65	-0.65	-0.65	1.29	-0.65	1.29
chrY	381191	391190	3	3	3	4	3	4	1	1	1	2	1	2	3.33	0.52	-0.65	-0.65	-0.65	1.29	-0.65	1.29
chrY	391191	1284375	3	3	3	4	3	4	1	1	1	2	1	2	3.33	0.52	-0.65	-0.65	-0.65	1.29	-0.65	1.29

chrY	1284376	1294375	3	3	3	4	3	4	1	1	1	2	1	2	3.33	0.52	-0.65	-0.65	-0.65	1.29	-0.65	1.29
chrY	1294376	2646970	3	3	3	4	3	4	1	1	1	2	1	2	3.33	0.52	-0.65	-0.65	-0.65	1.29	-0.65	1.29
chrY	2646971	2656970	3	3	3	4	3	4	1	1	1	2	1	2	3.33	0.52	-0.65	-0.65	-0.65	1.29	-0.65	1.29
chrY	2656971	2667970	4	2	2	4	2	2	2	1	1	2	1	1	2.67	1.03	1.29	-0.65	-0.65	1.29	-0.65	-0.65
chrY	2667971	7458570	4	2	2	2	2	2	2	1	1	1	1	1	2.33	0.82	2.04	-0.41	-0.41	-0.41	-0.41	-0.41
chrY	7458571	7468570	4	2	2	2	2	2	2	1	1	1	1	1	2.33	0.82	2.04	-0.41	-0.41	-0.41	-0.41	-0.41
chrY	7468571	7508570	4	2	2	2	2	4	2	1	1	1	1	2	2.67	1.03	1.29	-0.65	-0.65	-0.65	-0.65	1.29
chrY	7508571	7518570	4	2	2	2	2	4	2	1	1	1	1	2	2.67	1.03	1.29	-0.65	-0.65	-0.65	-0.65	1.29
chrY	7518571	9898965	4	2	2	2	2	2	2	1	1	1	1	1	2.33	0.82	2.04	-0.41	-0.41	-0.41	-0.41	-0.41
chrY	9898966	9908965	4	2	2	2	2	2	2	1	1	1	1	1	2.33	0.82	2.04	-0.41	-0.41	-0.41	-0.41	-0.41
chrY	9908966	9918965	4	4	3	2	4	2	2	1	0	1	1	1	3.17	0.98	0.85	0.85	-0.17	-1.19	0.85	-1.19
chrY	9918966	9929765	4	4	3	2	4	4	2	1	0	1	1	1	3.5	0.84	0.6	0.6	-0.6	-1.79	0.6	0.6
chrY	9929766	9939765	4	4	3	2	4	4	2	1	0	1	1	1	3.5	0.84	0.6	0.6	-0.6	-1.79	0.6	0.6
chrY	9939766	10089165	4	4	3	3	4	4	2	1	0	0	1	1	3.67	0.52	0.65	0.65	-1.29	-1.29	0.65	0.65
chrY	10089166	13409955	4	4	3	3	4	4	2	1	0	0	1	1	3.67	0.52	0.65	0.65	-1.29	-1.29	0.65	0.65
chrY	13409956	13479955	4	4	4	4	4	4	2	2	1	1	1	1	4	0	0	0	0	0	0	0
chrY	13479956	13519955	4	4	3	3	4	4	2	2	0	0	1	1	3.67	0.52	0.65	0.65	-1.29	-1.29	0.65	0.65
chrY	13519956	13529955	4	4	3	3	4	4	2	2	0	0	1	1	3.67	0.52	0.65	0.65	-1.29	-1.29	0.65	0.65
chrY	13529956	13870150	4	3	3	3	3	3	2	1	0	0	1	1	3.17	0.41	2.04	-0.41	-0.41	-0.41	-0.41	-0.41
chrY	13870151	13880150	4	3	3	3	3	3	2	1	0	0	1	1	3.17	0.41	2.04	-0.41	-0.41	-0.41	-0.41	-0.41
chrY	13880151	13890150	4	2	3	2	2	2	2	1	0	1	1	1	2.5	0.84	1.79	-0.6	0.6	-0.6	-0.6	-0.6
chrY	13890151	13900150	4	2	3	2	2	2	2	1	0	1	1	1	2.5	0.84	1.79	-0.6	0.6	-0.6	-0.6	-0.6
chrY	13900151	18590150	4	2	2	2	2	2	2	1	1	1	1	1	2.33	0.82	2.04	-0.41	-0.41	-0.41	-0.41	-0.41
chrY	18590151	18600150	4	2	2	2	2	2	2	1	1	1	1	1	2.33	0.82	2.04	-0.41	-0.41	-0.41	-0.41	-0.41
chrY	18600151	22107950	2	6	6	6	6	6	0	2	1	2	2	2	5.33	1.63	-2.04	0.41	0.41	0.41	0.41	0.41
chrY	22107951	22187550	2	6	6	6	6	6	1	2	1	2	2	2	5.33	1.63	-2.04	0.41	0.41	0.41	0.41	0.41
chrY	22187551	22197550	2	6	4	6	6	6	1	2	1	2	2	2	5	1.67	-1.79	0.6	-0.6	0.6	0.6	0.6
chrY	22197551	22227550	2	6	4	6	5	6	1	2	1	2	2	2	4.83	1.6	-1.77	0.73	-0.52	0.73	0.1	0.73
chrY	22227551	22488145	2	5	4	5	5	5	1	2	1	1	2	2	4.33	1.21	-1.93	0.55	-0.28	0.55	0.55	0.55
chrY	22488146	22498145	2	5	4	5	5	5	1	2	1	1	2	2	4.33	1.21	-1.93	0.55	-0.28	0.55	0.55	0.55
chrY	22498146	22508545	2	5	6	5	5	5	1	2	1	1	2	2	4.67	1.37	-1.95	0.24	0.98	0.24	0.24	0.24
chrY	22508546	22518545	2	6	6	5	5	5	1	2	1	1	2	2	4.83	1.47	-1.92	0.79	0.79	0.11	0.11	0.11
chrY	22518546	22599345	2	6	6	6	6	6	1	2	1	2	2	2	5.33	1.63	-2.04	0.41	0.41	0.41	0.41	0.41
chrY	22599346	22609345	2	6	6	6	6	6	1	2	1	2	2	2	5.33	1.63	-2.04	0.41	0.41	0.41	0.41	0.41
chrY	22609346	58908935	2	6	6	6	6	6	1	2	1	2	2	2	5.33	1.63	-2.04	0.41	0.41	0.41	0.41	0.41
chrY	58908936	58969130	2	6	6	6	6	6	1	2	1	2	2	2	5.33	1.63	-2.04	0.41	0.41	0.41	0.41	0.41
chrY	58969131	58979130	4	5	6	5	5	6	2	2	1	1	1	2	5.17	0.75	-1.55	-0.22	1.11	-0.22	-0.22	1.11
chrY	58979131	59029930	4	5	6	5	5	5	2	2	1	1	1	2	5	0.63	-1.58	0	1.58	0	0	0
chrY	59029931	59039930	4	5	6	5	5	5	2	2	1	1	1	2	5	0.63	-1.58	0	1.58	0	0	0
chrY	59039931	59348730	2	5	6	5	5	5	1	2	1	1	1	2	4.67	1.37	-1.95	0.24	0.98	0.24	0.24	0.24

Table S7. Somatic structure variations of six tumor specimens.

1. The result is from Meerkat.

2. The gene annotation is from wANNOVAR.

	upstream (5')			downstream (3')			SharingOfSixTissue						sv type		sv	breakpoint of upstream (5')					breakpoint of downstream (3')				
NO.	segment	bkpos	strand	segment	bkpos	strand	T1	T2	T3	T4	HVTT	PVTT	type	stream	mechanism	Func	gene_symbol	gene_strand	gene_type	distance	Func	gene_symbol	gene_strand	gene_type	distance
1	chr1	756268	+	chr1	758749	+	N	N	N	Y	N	N	del	na	alt-EJ	intergenic	FAM87B,LINC00115	+,-	lincRNA,linc	1054;5318	intergenic	FAM87B,LINC00115	+,-	lincRNA,linc	3535;2837
2	chr1	869885	+	chr1	870348	+	N	N	N	Y	N	N	del	na	alt-EJ	intronic	SAMD11	+	protein_cod	0	intronic	SAMD11	+	protein_cod	0
3	chr1	1142719	+	chr1	1143141	+	Y	Y	Y	N	Y	Y	del	na	NHEJ	upstream	TNFRSF18	-	protein_cod	0	intergenic	TNFRSF18,TNFRSF4	-,-	protein_cod	1070;3565
4	chr1	1232663	+	chr1	1233036	+	N	N	Y	N	N	N	del	na	alt-EJ	intronic	ACAP3	-	protein_cod	0	intronic	ACAP3	-	protein_cod	0
5	chr1	2585567	+	chr1	2620930	+	N	N	N	Y	N	N	del	na	alt-EJ	intronic	TTC34	-	protein_cod	0	intronic	TTC34	-	protein_cod	0
6	chr1	2617080	+	chr1	2623571	+	N	N	N	Y	N	N	del	na	alt-EJ	intronic	TTC34	-	protein_cod	0	intronic	TTC34	-	protein_cod	0
7	chr1	2618918	+	chr1	2626177	+	Y	N	N	N	N	N	del	na	alt-EJ	intronic	TTC34	-	protein_cod	0	intronic	TTC34	-	protein_cod	0
8	chr1	2621300	+	chr1	2628882	+	N	N	N	Y	N	N	del	na	alt-EJ	intronic	TTC34	-	protein_cod	0	intronic	TTC34	-	protein_cod	0
9	chr1	7570071	+	chr1	7571527	+	Y	N	N	N	N	N	del	na	alt-EJ	intronic;intr	CAMTA1	+	protein_cod	0	intronic;intr	CAMTA1	+	protein_cod	0
10	chr1	14437073	+	chr1	14438941	+	Y	N	N	N	N	N	del	na	alt-EJ	intergenic	PRDM2,RNU6-1265P	+,-	protein_cod	285499;13655	intergenic	PRDM2,RNU6-1265P	+,-	protein_cod	287367;11787
11	chr1	16376519	+	chr1	16386257	+	N	N	Y	N	N	N	del	na	alt-EJ	intronic	CLCNKB	+	protein_cod	0	intronic	FAM131C	-	protein_cod	0
12	chr1	17009563	+	chr1	17012755	+	N	Y	Y	N	N	N	del	na	alt-EJ	intergenic	AL021920.1,EIF1AXP	+,-	miRNA,pseu	1740;2553	upstream	EIF1AXP1	-	pseudogene	0
13	chr1	17676167	+	chr1	17677661	+	Y	N	Y	Y	N	N	del	na	alt-EJ	intronic	PADI4	+	protein_cod	0	intronic	PADI4	+	protein_cod	0
14	chr1	17676184	+	chr1	17677519	+	N	N	N	N	N	Y	del	na	NHEJ	intronic	PADI4	+	protein_cod	0	intronic	PADI4	+	protein_cod	0
15	chr1	20497191	+	chr1	20497740	+	Y	Y	Y	Y	Y	Y	del	na	alt-EJ	intronic	PLA2G2C	-	protein_cod	0	intronic	PLA2G2C	-	protein_cod	0
16	chr1	21786419	+	chr1	21786692	+	N	N	Y	N	N	N	del	na	alt-EJ	ncRNA_exo	PFN1P10	-	pseudogene	0	intronic	NBPF3	+	protein_cod	0
17	chr1	24994644	+	chr1	24997171	+	Y	N	N	N	N	N	del	na	alt-EJ	intronic;intr	SRRM1	+	protein_cod	0	intronic;intr	SRRM1	+	protein_cod	0
18	chr1	37423392	+	chr1	37423505	+	N	N	N	N	N	Y	del	na	alt-EJ	intronic	GRIK3	-	protein_cod	0	intronic	GRIK3	-	protein_cod	0
19	chr1	53468857	+	chr1	67082731	+	Y	Y	Y	Y	Y	Y	del	na	NHEJ	intronic	SCP2	+	protein_cod	0	intronic	SGIP1	+	protein_cod	0
20	chr1	60656266	+	chr1	235798859	+	N	N	N	N	Y	N	del	na	alt-EJ	intergenic	RP11-575B7.3,RP11-7	+,-	lincRNA,linc	40915;349655	intronic	GNG4	-	protein_cod	0
21	chr1	62082860	+	chr1	62083705	+	Y	Y	Y	Y	Y	Y	del	na	alt-EJ	intergenic	NFIA,AC099791.1	+,+	protein_cod	154395;11843	intergenic	NFIA,AC099791.1	+,+	protein_cod	155240;10998
22	chr1	67123907	+	chr1	67277113	+	Y	Y	Y	Y	Y	Y	del	na	NHEJ	intronic	SGIP1	+	protein_cod	0	intergenic	INSL5,WDR78	-,-	protein_cod	10174;1455
23	chr1	68008004	+	chr1	68008862	+	N	N	N	N	N	Y	del	na	alt-EJ	intergenic	RNU6-1031P,RP11-39	+,+	snRNA,pseu	1088;18752	intergenic	RNU6-1031P,RP11-393N	+,+	snRNA,pseu	1946;17894
24	chr1	72766323	+	chr1	72811840	+	Y	Y	Y	Y	Y	N	del	na	NHEJ	upstream	RPL31P12	+	pseudogene	0	intergenic	RPL31P12,RNU6-1246P	+,-	pseudogene	44328;371506
25	chr1	86400851	+	chr1	86404539	+	N	N	Y	N	N	N	del	na	NHEJ	intronic;intr	COL24A1	-	protein_cod	0	intronic;intr	COL24A1	-	protein_cod	0
26	chr1	87081599	+	chr1	87101802	+	Y	Y	Y	Y	Y	Y	del	na	alt-EJ	ncRNA_intr	RP4-651E10.4	-	antisense	0	ncRNA_intr	CLCA3P,RP4-651E10.4	+,-	pseudogene	0
27	chr1	94288373	+	chr1	94291258	+	Y	Y	Y	Y	Y	Y	del	na	alt-EJ	intronic	BCAR3	-	protein_cod	0	intronic	BCAR3	-	protein_cod	0
28	chr1	103124309	+	chr1	103124729	+	N	N	N	N	Y	N	del	na	alt-EJ	intergenic	RP11-202K23.1,RP5-9	-,-	lincRNA,linc	269123;104569	intergenic	RP11-202K23.1,RP5-936	-,-	lincRNA,linc	269543;104149
29	chr1	113799629	+	chr1	113800092	+	N	Y	N	Y	N	Y	del	na	alt-EJ	intergenic	RP11-389O22.5,MAGI	+,+	pseudogene	57749;133742	intergenic	RP11-389O22.5,MAGI3	+,+	pseudogene	58212;133279
30	chr1	142535592	+	chr1	142537369	+	N	N	N	N	N	Y	del	na	alt-EJ	intergenic	NONE,RP11-417J8.1	NONE,+	NONE,lincR	NONE;17701	intergenic	NONE,RP11-417J8.1	NONE,+	NONE,lincR	NONE;15924
31	chr1	142535917	+	chr1	142537362	+	N	N	Y	Y	N	N	del	na	alt-EJ	intergenic	NONE,RP11-417J8.1	NONE,+	NONE,lincR	NONE;17376	intergenic	NONE,RP11-417J8.1	NONE,+	NONE,lincR	NONE;15931
32	chr1	142536075	+	chr1	142536899	+	N	N	N	N	Y	N	del	na	alt-EJ	intergenic	NONE,RP11-417J8.1	NONE,+	NONE,lincR	NONE;17218	intergenic	NONE,RP11-417J8.1	NONE,+	NONE,lincR	NONE;16394
33	chr1	144901180	+	chr1	144906810	+	Y	Y	Y	Y	Y	Y	del	na	NHEJ	UTR3	PDE4DIP	-	protein_cod	0	intronic	PDE4DIP	-	protein_cod	0
34	chr1	145092947	+	chr1	145097083	+	Y	Y	Y	Y	Y	Y	del	na	alt-EJ	intergenic	RP4-725K1.1,SEC22B	-,+	pseudogene	4380;3273	ncRNA_intr	SEC22B	+	processed_	0
35	chr1	158867531	+	chr1	158869983	+	Y	Y	Y	Y	Y	Y	del	na	alt-EJ	intergenic	RP11-393K10.1,PYHIN	+,+	pseudogene	14241;33055	intergenic	RP11-393K10.1,PYHIN1	+,+	pseudogene	16693;30603
36	chr1	159648707	+	chr1	159649658	+	Y	Y	Y	Y	Y	Y	del	na	NHEJ	intergenic	OR10J6P,CRPP1	+,-	pseudogene	79690;26066	intergenic	OR10J6P,CRPP1	+,-	pseudogene	80641;25115
37	chr1	162230748	+	chr1	162231278	+	N	Y	N	Y	Y	Y	del	na	NHEJ	intronic	NOS1AP	+	protein_cod	0	intronic	NOS1AP	+	protein_cod	0
38	chr1	165731227	+	chr1	165731508	+	Y	Y	Y	N	Y	Y	del	na	alt-EJ	intronic	TMCO1	-	protein_cod	0	intronic	TMCO1	-	protein_cod	0
39	chr1	168427011	+	chr1	168428843	+	Y	Y	Y	Y	Y	Y	del	na	alt-EJ	intergenic	QRSL1P1,RP5-968D2	-,-	pseudogene	6562;6441	intergenic	QRSL1P1,RP5-968D22.	-,-	pseudogene	8394;4609
40	chr1	174796558	+	chr1	174801847	+	Y	Y	Y	Y	Y	Y	del	na	alt-EJ	intronic	RABGAP1L	+	protein_cod	0	intronic	RABGAP1L	+	protein_cod	0
41	chr1	185630047	+	chr1	185632327	+	Y	Y	Y	Y	Y	Y	del	na	alt-EJ	intergenic	GS1-204I12.4,HMCN1	+,+	lincRNA,pro	3747;73636	intergenic	GS1-204I12.4,HMCN1	+,+	lincRNA,pro	6027;71356
42	chr1	188539455	+	chr1	188540231	+	Y	Y	Y	Y	Y	Y	del	na	alt-EJ	intergenic	RP11-669M2.1,RP11-6	+,+	lincRNA,pse	33859;101029	intergenic	RP11-669M2.1,RP11-669	+,+	lincRNA,pse	34635;100253
43	chr1	194450987	+	chr1	194454313	+	N	N	N	Y	N	N	del	na	alt-EJ	intergenic	RP11-476H20.1,RNU6	+,-	lincRNA,sn	129431;6246	intergenic;i	RP11-476H20.1,RNU6-9	+,-	lincRNA,sn	132757;2920
44	chr1	194451058	+	chr1	194454313	+	N	Y	Y	N	N	Y	del	na	NHEJ	intergenic	RP11-476H20.1,RNU6	+,-	lincRNA,sn	129502;6175	intergenic;i	RP11-476H20.1,RNU6-9	+,-	lincRNA,sn	132757;2920
45	chr1	194451099	+	chr1	194454313	+	N	N	N	N	Y	N	del	na	alt-EJ	intergenic	RP11-476H20.1,RNU6	+,-	lincRNA,sn	129543;6134	intergenic;i	RP11-476H20.1,RNU6-9	+,-	lincRNA,sn	132757;2920
46	chr1	194517609	+	chr1	194517868	+	N	N	N	N	Y	N	del	na	NHEJ	intergenic	RNU6-983P,RP11-489	-,+	snRNA,pseu	60274;170316	intergenic	RNU6-983P,RP11-489C1	-,+	snRNA,pseu	60533;170057
47	chr1	198773749	+	chr1	198775415	+	N	Y	Y	Y	N	Y	del	na	NHEJ	intergenic	PTPRC,MIR181A1HG	+,-	protein_cod	47204;2873	intergenic	PTPRC,MIR181A1HG	+,-	protein_cod	48870;1207
48	chr1	206814684	+	chr1	206815662	+	Y	Y	Y	N	Y	Y	del	na	alt-EJ	intronic	DYRK3	+	protein_cod	0	intronic	DYRK3	+	protein_cod	0
49	chr1	209627163	+	chr1	213087909	+	N	N	Y	N	N	N	del	na	alt-EJ	intergenic	MIR205HG,RP1-272L1	+,-	lincRNA,linc	20980;74637	intergenic	FLVCR1,VASH2	+,+	protein_cod	15204;35953
50	chr1	213083569	+	chr1	213087762	+	N	N	N	N	N	Y	del	na	TEI_complex	intergenic	FLVCR1,VASH2	+,+	protein_cod	10864;40293	intergenic	FLVCR1,VASH2	+,+	protein_cod	15057;36100
51	chr1	213714501	+	chr1	225312465	+	N	Y	N	Y	N	N	del	na	alt-EJ	ncRNA_intr	RP11-554K11.2	+	lincRNA	0	intronic	DNAH14	+	protein_cod	0
52	chr1	220502944	+	chr1	220503584	+	Y	Y	Y	Y	Y	Y	del	na	alt-EJ	intergenic	RP11-302I18.1,RP11-3	-,-	pseudogene	12003;30129	intergenic	RP11-302I18.1,RP11-302	-,-	pseudogene	12643;29489
53	chr1	224247109	+	chr1	224247586	+	Y	Y	Y	Y	Y	Y	del	na	alt-EJ	intergenic	RP11-504P24.5,RN7S	-,-	pseudogene	24351;47875	intergenic	RP11-504P24.5,RN7SK	-,-	pseudogene	24828;47398
54	chr1	226002815	+	chr1	226003143	+	Y	Y	Y	Y	Y	Y	del	na	alt-EJ	intronic	EPHX1	+	protein_cod	0	intronic	EPHX1	+	protein_cod	0
55	chr1	229373388	+	chr1	229373763	+	N	N	N	N	N	Y	del	na	NHEJ	intergenic	RP5-1061H20.5,TMEM	-,+	antisense,p	10079;11995	intergenic	RP5-1061H20.5,TMEM7	-,+	antisense,p	10454;11620
56	chr1	229812535	+	chr1	229820839	+	Y	Y	Y	Y	Y	Y	del	na	alt-EJ	intergenic	URB2,RP11-108F13.2	+,-	protein_cod	16589;12211	intergenic	URB2,RP11-108F13.2	+,-	protein_cod	24893;3907
57	chr1	233962210	+	chr1	233963554	+	N	Y	N	Y	Y	Y	del	na	alt-EJ	intergenic	KCNK1,RP11-301G21.	+,-	protein_cod	153952;9616	intergenic	KCNK1,RP11-301G21.1	+,-	protein_cod	155296;8272
58	chr1	234318645	+	chr1	234319749	+	Y	Y	Y	Y	Y	Y	del	na	alt-EJ	intronic	SLC35F3	+	protein_cod	0	intronic	SLC35F3	+	protein_cod	0
59	chr10	703123	+	chr10	703820	+	Y	N	N	N	N	N	del	na	alt-EJ	intronic	DIP2C,PRR26	-,+	protein_cod	0	intronic	DIP2C,PRR26	-,+	protein_cod	0
60	chr10	3095720	+	chr10	3095955	+	N	N	N	N	Y	N	del	na	alt-EJ	intergenic	RP11-118K6.2,RP11-1	+,-	lincRNA,linc	40417;11896	intergenic	RP11-118K6.2,RP11-118	+,-	lincRNA,linc	40652;11661
61	chr10	4290062	+	chr10	4291683	+	Y	Y	Y	Y	Y	Y	del	na	NHEJ	intergenic	LINC00702,LINC0070	-,+	lincRNA,linc	3958;136376	intergenic	LINC00702,LINC00703	-,+	lincRNA,linc	5579;134755
62	chr10	12596195	+	chr10	12596578	+	Y	N	Y	Y	N	Y	del	na	alt-EJ	intronic	CAMK1D	+	protein_cod	0	intronic	CAMK1D	+	protein_cod	0
63	chr10	18503075	+	chr10	18503590	+	N	Y	Y	Y	N	Y	del	na	NHEJ	intronic	CACNB2	+	protein_cod	0	intronic	CACNB2	+	protein_cod	0
64	chr10	18850033	+	chr10	18850182	+	N	Y	N	N	N	N	del	na	VNTR	intronic	NSUN6	-	protein_cod	0	intronic	NSUN6	-	protein_cod	0
65	chr10	18850129	+	chr10	18850402	+	N	N	N	N	N	Y	del	na	alt-EJ	intronic	NSUN6	-	protein_cod	0	intronic	NSUN6	-	protein_cod	0
66	chr10	38807684	+	chr10	38868787	+	N	N	Y	N	N	N	del	na	alt-EJ	intergenic	RP11-291L22.4,ABCD	+,+	lincRNA,pse	52580;86865	intergenic	RP11-291L22.4,ABCD1P	+,+	lincRNA,pse	113683;25762
67	chr10	38816527	+	chr10	38818875	+	N	Y	N	N	N	N	del	na	alt-EJ	intergenic;i	RP11-291L22.4,ABCD	+,+	lincRNA,pse	61423;78022	intergenic;i	RP11-291L22.4,ABCD1P	+,+	lincRNA,pse	63771;75674
68	chr10	42355729	+	chr10	42376881	+	N	N	N	N	Y	N	del	na	alt-EJ	intergenic	NONE,KSR1P1	NONE,-	NONE,pseu	NONE;289029	intergenic	NONE,KSR1P1	NONE,-	NONE,pseu	NONE;267877
69	chr10	42365926	+	chr10	42376685	+	N	N	N	N	N	Y	del	na	alt-EJ	intergenic	NONE,KSR1P1	NONE,-	NONE,pseu	NONE;278832	intergenic	NONE,KSR1P1	NONE,-	NONE,pseu	NONE;268073
70	chr10	45158151	+	chr10	45158501	+	N	N	N	Y	Y	N	del	na	alt-EJ	intergenic	RP11-733D4.1,EIF2A	-,+	lincRNA,pse	60599;143740	intergenic	RP11-733D4.1,EIF2AP4	-,+	lincRNA,pse	60949;143390
71	chr10	45158417	+	chr10	45158526	+	N	N	Y	N	N	N	del	na	alt-EJ	intergenic	RP11-733D4.1,EIF2A	-,+	lincRNA,pse	60865;143474	intergenic	RP11-733D4.1,EIF2AP4	-,+	lincRNA,pse	60974;143365
72	chr10	53984360	+	chr10	54034068	+	N	N	N	N	Y	N	del	na	alt-EJ	intronic	PRKG1	+	protein_cod	0	ncRNA_intr	PRKG1-AS1	-	antisense	0
73	chr10	53984584	+	chr10	54034054	+	N	N	N	N	N	Y	del	na	alt-EJ	intronic	PRKG1	+	protein_cod	0	ncRNA_intr	PRKG1-AS1	-	antisense	0
74	chr10	56682779	+	chr10	56684360	+	Y	Y	Y	N	Y	Y	del	na	alt-EJ	intronic	PCDH15	-	protein_cod	0	intronic	PCDH15	-	protein_cod	0
75	chr10	67306933	+	chr10	67315296	+	Y	Y	Y	Y	Y	Y	del	na	alt-EJ	intergenic	RP11-428G2.1,RP11-2	+,+	lincRNA,linc	232205;23163	intergenic	RP11-428G2.1,RP11-222	+,+	lincRNA,linc	240568;14800
76	chr10	70068226	+	chr10	70070646	+	Y	Y	Y	Y	Y	Y	del	na	alt-EJ	intronic	PBLD	-	protein_cod	0	intronic	PBLD	-	protein_cod	0
77	chr10	77623964	+	chr10	77625205	+	Y	Y	Y	Y	Y	Y	del	na	alt-EJ	intronic	C10orf11	+	protein_cod	0	intronic	C10orf11	+	protein_cod	0
78	chr10	90940514	+	chr10	90948052	+	Y	Y	Y	Y	Y	Y	del	na	alt-EJ	intergenic	RP11-341B24.3,CH25	+,-	pseudogene	41299;25180	intergenic	RP11-341B24.3,CH25H	+,-	pseudogene	48837;17642

79	chr10	91998538	+	chr10	92002031	+	Y	Y	Y	Y	Y	Y	del	na	alt-EJ	intergenic	RN7SKP143,RP11-15	-,-	misc_RNA,l	74821;163740	intergenic	RN7SKP143,RP11-15K3	-,-	misc_RNA,l	78314;160247
80	chr10	93633336	+	chr10	93634564	+	Y	Y	Y	N	Y	Y	del	na	alt-EJ	intergenic	TNKS2,FGFBP3	+,-	protein_cod	8303;33010	intergenic	TNKS2,FGFBP3	+,-	protein_cod	9531;31782
81	chr10	100496980	+	chr10	100497289	+	N	Y	N	Y	Y	Y	del	na	alt-EJ	intronic	HPSE2	-	protein_cod	0	intronic	HPSE2	-	protein_cod	0
82	chr10	107253000	+	chr10	107253354	+	Y	Y	Y	Y	Y	Y	del	na	alt-EJ	intergenic	SORCS3,RNU6-463P	+,+	protein_cod	228007;41417	intergenic	SORCS3,RNU6-463P	+,+	protein_cod	228361;41063
83	chr10	107950683	+	chr10	107951597	+	Y	Y	Y	Y	Y	Y	del	na	alt-EJ	intergenic	RP11-298H24.1,RP11-	+,+	lincRNA,pse	2203;359181	intergenic	RP11-298H24.1,RP11-44	+,+	lincRNA,pse	3117;358267
84	chr10	108030319	+	chr10	108032544	+	Y	N	Y	Y	Y	Y	del	na	alt-EJ	intergenic	RP11-298H24.1,RP11-	+,+	lincRNA,pse	81839;279545	intergenic	RP11-298H24.1,RP11-44	+,+	lincRNA,pse	84064;277320
85	chr10	114112182	+	chr10	114116660	+	Y	Y	Y	Y	Y	Y	del	na	alt-EJ	ncRNA_intr	GUCY2GP	-	pseudogene	0	upstream	GUCY2GP	-	pseudogene	0
86	chr10	117711616	+	chr10	117712003	+	Y	Y	Y	Y	Y	N	del	na	NHEJ	intergenic	ATRNL1,GFRA1	+,-	protein_cod	3113;104828	intergenic	ATRNL1,GFRA1	+,-	protein_cod	3500;104441
87	chr10	127191656	+	chr10	127197236	+	Y	Y	Y	N	Y	N	del	na	alt-EJ	intergenic	RPS27P18,TEX36-AS	-,+	pseudogene	29155;71284	intergenic	RPS27P18,TEX36-AS1	-,+	pseudogene	34735;65704
88	chr10	128582125	+	chr10	128770974	+	N	N	Y	N	N	N	del	na	alt-EJ	intergenic	SNORD60,DOCK1	-,+	snoRNA,pro	113782;11853	intronic	DOCK1	+	protein_cod	0
89	chr10	131595178	+	chr10	131595475	+	Y	N	Y	N	Y	Y	del	na	alt-EJ	intergenic	RP11-109A6.3,EBF3	+,-	lincRNA,pro	27479;38369	intergenic	RP11-109A6.3,EBF3	+,-	lincRNA,pro	27776;38072
90	chr10	134831025	+	chr10	134831245	+	Y	N	Y	N	N	Y	del	na	alt-EJ	intergenic	LINC01168,GPR123	+,+	lincRNA,pro	41167;53408	intergenic	LINC01168,GPR123	+,+	lincRNA,pro	41387;53188
91	chr10	134831028	+	chr10	134831667	+	N	Y	N	N	N	N	del	na	alt-EJ	intergenic	LINC01168,GPR123	+,+	lincRNA,pro	41170;53405	intergenic	LINC01168,GPR123	+,+	lincRNA,pro	41809;52766
92	chr10	135104756	+	chr10	135105968	+	Y	Y	Y	Y	Y	Y	del	na	alt-EJ	intronic	TUBGCP2	-	protein_cod	0	intronic	TUBGCP2	-	protein_cod	0
93	chr11	1016980	+	chr11	1017598	+	N	N	N	Y	N	N	del	na	alt-EJ	exonic	MUC6	-	protein_cod	0	exonic	MUC6	-	protein_cod	0
94	chr11	2215323	+	chr11	2215578	+	N	Y	Y	Y	Y	Y	del	na	alt-EJ	intergenic	MIR4686,ASCL2	+,-	miRNA,prot	20955;74402	intergenic	MIR4686,ASCL2	+,-	miRNA,prot	21210;74147
95	chr11	3463795	+	chr11	67592760	+	N	N	Y	N	N	N	del	na	alt-EJ	intergenic	FAM86GP,RP13-726E	-,+	pseudogene	20069;26754	intergenic	FAM86C2P,RP11-119D9.	-,+	pseudogene	19953;15653
96	chr11	5521631	+	chr11	5523173	+	Y	Y	Y	Y	N	Y	del	na	alt-EJ	ncRNA_intr	AC104389.28	-	processed_	0	ncRNA_intr	AC104389.28	-	processed_	0
97	chr11	5784574	+	chr11	5809284	+	Y	Y	Y	Y	Y	Y	del	na	alt-EJ	intronic	TRIM5	-	protein_cod	0	exonic	OR52N1	-	protein_cod	0
98	chr11	9644770	+	chr11	10154921	+	Y	N	N	N	N	N	del	na	alt-EJ	intergenic;ir	RP11-16F15.1,RP11-1	+,-	pseudogene	16614;7785	intronic;intro	SBF2	-	protein_cod	0
99	chr11	10292737	+	chr11	10293844	+	Y	Y	Y	Y	Y	Y	del	na	alt-EJ	intronic	SBF2	-	protein_cod	0	intronic	SBF2	-	protein_cod	0
100	chr11	11822987	+	chr11	11824624	+	Y	Y	Y	Y	Y	Y	del	na	NHEJ	intergenic	RP11-756D7.1,USP47	-,+	pseudogene	42007;39983	intergenic	RP11-756D7.1,USP47	-,+	pseudogene	43644;38346
101	chr11	15529789	+	chr11	15536549	+	Y	Y	Y	Y	Y	Y	del	na	alt-EJ	intergenic	snoMBII-202,RP11-53	-,+	snoRNA,linc	26350;44612	intergenic	snoMBII-202,RP11-531H	-,+	snoRNA,linc	33110;37852
102	chr11	16996173	+	chr11	16996598	+	Y	Y	Y	Y	Y	Y	del	na	alt-EJ	intronic	PLEKHA7	-	protein_cod	0	intronic	PLEKHA7	-	protein_cod	0
103	chr11	29967578	+	chr11	29968467	+	Y	Y	N	Y	Y	Y	del	na	NHEJ	intergenic	CTD-3138F19.1,RP11-	+,-	lincRNA,linc	64317;34082	intergenic	CTD-3138F19.1,RP11-21	+,-	lincRNA,linc	65206;33193
104	chr11	31393774	+	chr11	31397648	+	N	Y	N	N	Y	N	del	na	alt-EJ	intronic	DNAJC24	+	protein_cod	0	intronic	DNAJC24	+	protein_cod	0
105	chr11	39795370	+	chr11	39796866	+	N	N	Y	N	N	N	del	na	NHEJ	intergenic;ir	AC027806.1,AC02174	+,-	miRNA,miR	42917;87686	intergenic;ir	AC027806.1,AC021749.1	+,-	miRNA,miR	44413;86190
106	chr11	40699730	+	chr11	40700469	+	Y	Y	Y	N	Y	Y	del	na	alt-EJ	intronic	LRRC4C	-	protein_cod	0	intronic	LRRC4C	-	protein_cod	0
107	chr11	47660603	+	chr11	47663931	+	N	N	N	N	Y	N	del	na	alt-EJ	exonic	MTCH2	-	protein_cod	0	exonic	MTCH2	-	protein_cod	0
108	chr11	48600857	+	chr11	48604284	+	Y	Y	Y	Y	Y	Y	del	na	alt-EJ	upstream	OR4A45P	+	pseudogene	0	intergenic	OR4A45P,OR4A41P	+,+	pseudogene	2396;7028
109	chr11	58166990	+	chr11	58168159	+	Y	Y	Y	Y	Y	Y	del	na	alt-EJ	intergenic	OR5B15P,OR5B3	-,-	pseudogene	11031;2947	intergenic	OR5B15P,OR5B3	-,-	pseudogene	12200;1778
110	chr11	58631773	+	chr11	58636555	+	Y	Y	Y	Y	Y	Y	del	na	alt-EJ	intergenic	GLYATL2,GLYATL1P2	-,+	protein_cod	19516;14002	intergenic	GLYATL2,GLYATL1P2	-,+	protein_cod	24298;9220
111	chr11	60372231	+	chr11	60372359	+	Y	N	N	N	N	N	del	na	VNTR	intergenic	MS4A13,LINC00301	+,+	protein_cod	62037;10978	intergenic	MS4A13,LINC00301	+,+	protein_cod	62165;10850
112	chr11	62512344	+	chr11	62513155	+	N	N	N	Y	N	N	del	na	alt-EJ	intergenic	TTC9C,ZBTB3	+,-	protein_cod	4588;3447	intergenic	TTC9C,ZBTB3	+,-	protein_cod	5399;2636
113	chr11	62512527	+	chr11	62513227	+	N	N	N	N	Y	N	del	na	alt-EJ	intergenic	TTC9C,ZBTB3	+,-	protein_cod	4771;3264	intergenic	TTC9C,ZBTB3	+,-	protein_cod	5471;2564
114	chr11	68183114	+	chr11	68183425	+	N	Y	Y	Y	Y	Y	del	na	alt-EJ	intronic	LRP5	+	protein_cod	0	intronic	LRP5	+	protein_cod	0
115	chr11	79948611	+	chr11	79949691	+	N	Y	Y	N	Y	Y	del	na	NHEJ	intergenic	RP11-885L14.1,CTD-2	+,-	pseudogene	247938;84053	intergenic	RP11-885L14.1,CTD-255	+,-	pseudogene	249018;82973
116	chr11	83143381	+	chr11	83143662	+	N	Y	Y	N	Y	N	del	na	alt-EJ	intergenic	RP11-727A23.10,DLG	+,-	antisense,p	8822;22674	intergenic	RP11-727A23.10,DLG2	+,-	antisense,p	9103;22393
117	chr11	98248206	+	chr11	98248601	+	Y	Y	Y	Y	Y	Y	del	na	alt-EJ	intergenic	RP11-684B20.1,CTD-2	-,-	pseudogene	246280;187598	intergenic	RP11-684B20.1,CTD-234	-,-	pseudogene	246675;187203
118	chr11	106878596	+	chr11	106878979	+	N	Y	Y	Y	N	Y	del	na	alt-EJ	intronic	GUCY1A2	-	protein_cod	0	intronic	GUCY1A2	-	protein_cod	0
119	chr11	114424910	+	chr11	114431927	+	Y	Y	Y	Y	Y	Y	del	na	alt-EJ	UTR5	NXPE1	-	protein_cod	0	intergenic	NXPE1,NXPE4	-,-	protein_cod	1310;9386
120	chr11	134605144	+	chr11	134605829	+	Y	Y	Y	Y	Y	Y	del	na	alt-EJ	upstream	RP11-469N6.1	+	lincRNA	0	ncRNA_exo	RP11-469N6.1	+	lincRNA	0
121	chr11	134732899	+	chr11	134734114	+	Y	Y	Y	Y	Y	Y	del	na	alt-EJ	intergenic	RP11-469N6.1,RP11-5	+,+	lincRNA,linc	99195;87703	intergenic	RP11-469N6.1,RP11-555	+,+	lincRNA,linc	100410;86488
122	chr12	1028821	+	chr12	1029628	+	N	Y	Y	N	N	N	del	na	alt-EJ	intronic	RAD52	-	protein_cod	0	intronic	RAD52	-	protein_cod	0
123	chr12	1029131	+	chr12	1029297	+	N	N	N	Y	N	N	del	na	alt-EJ	intronic	RAD52	-	protein_cod	0	intronic	RAD52	-	protein_cod	0
124	chr12	1437991	+	chr12	1438612	+	Y	Y	Y	Y	Y	Y	del	na	alt-EJ	intronic	ERC1	+	protein_cod	0	intronic	ERC1	+	protein_cod	0
125	chr12	1864013	+	chr12	1864329	+	Y	Y	N	Y	Y	Y	del	na	alt-EJ	intronic	ADIPOR2	+	protein_cod	0	intronic	ADIPOR2	+	protein_cod	0
126	chr12	3591313	+	chr12	3595629	+	Y	Y	Y	Y	Y	Y	del	na	alt-EJ	intronic	PRMT8	+	protein_cod	0	intronic	PRMT8	+	protein_cod	0
127	chr12	8558485	+	chr12	8568227	+	Y	Y	Y	N	Y	Y	del	na	NHEJ	downstream	RP11-113C12.8	-	pseudogene	0	ncRNA_exo	OR7E140P	-	pseudogene	0
128	chr12	9557740	+	chr12	9558492	+	N	N	N	Y	N	N	del	na	alt-EJ	ncRNA_intr	RP11-599J14.2	-	pseudogene	0	ncRNA_intr	RP11-599J14.2	-	pseudogene	0
129	chr12	9557777	+	chr12	9558815	+	N	N	N	N	N	Y	del	na	alt-EJ	ncRNA_intr	RP11-599J14.2	-	pseudogene	0	ncRNA_intr	RP11-599J14.2	-	pseudogene	0
130	chr12	9558121	+	chr12	9558535	+	Y	N	N	N	N	N	del	na	alt-EJ	ncRNA_intr	RP11-599J14.2	-	pseudogene	0	ncRNA_intr	RP11-599J14.2	-	pseudogene	0
131	chr12	11166792	+	chr12	11221980	+	N	N	Y	Y	N	N	del	na	alt-EJ	intergenic	TAS2R20,TAS2R19	-,-	protein_cod	16318;7426	intergenic	TAS2R46,TAS2R64P	-,-	protein_cod	7087;7388
132	chr12	11166902	+	chr12	11222092	+	N	N	N	N	N	Y	del	na	alt-EJ	intergenic	TAS2R20,TAS2R19	-,-	protein_cod	16428;7316	intergenic	TAS2R46,TAS2R64P	-,-	protein_cod	7199;7276
133	chr12	11178538	+	chr12	11205497	+	Y	N	Y	N	N	Y	del	na	alt-EJ	intergenic;ir	TAS2R19,TAS2R31	-,-	protein_cod	3319;4448	intergenic;ir	TAS2R63P,TAS2R46	-,-	pseudogene	3642;8467
134	chr12	11341899	+	chr12	11342214	+	Y	Y	N	N	Y	Y	del	na	alt-EJ	intergenic	TAS2R42,RP11-144O2	-,-	protein_cod	2356;7088	intergenic	TAS2R42,RP11-144O23.	-,-	protein_cod	2671;6773
135	chr12	12026415	+	chr12	12027154	+	Y	Y	Y	Y	Y	Y	del	na	NHEJ	intronic	ETV6	+	protein_cod	0	intronic	ETV6	+	protein_cod	0
136	chr12	26937704	+	chr12	26937938	+	N	N	N	N	Y	N	del	na	alt-EJ	intronic	ITPR2	-	protein_cod	0	intronic	ITPR2	-	protein_cod	0
137	chr12	30371317	+	chr12	30371743	+	Y	Y	Y	N	Y	Y	del	na	NHEJ	downstream	RP11-776A13.1	+	lincRNA	0	downstream	RP11-776A13.1	+	lincRNA	0
138	chr12	30508189	+	chr12	30508637	+	Y	Y	Y	Y	Y	Y	del	na	alt-EJ	intergenic	RP11-155I9.1,RP11-77	+,+	lincRNA,pse	33639;99663	intergenic	RP11-155I9.1,RP11-776	+,+	lincRNA,pse	34087;99215
139	chr12	31271947	+	chr12	31273232	+	N	Y	N	N	N	N	del	na	alt-EJ	ncRNA_exo	RP11-551L14.1	-	pseudogene	0	ncRNA_intr	RP11-551L14.1	-	pseudogene	0
140	chr12	33296556	+	chr12	33307370	+	Y	Y	Y	Y	Y	Y	del	na	alt-EJ	intergenic	AC026357.1,SNORD1	-,-	miRNA,snol	101801;218962	intergenic	AC026357.1,SNORD112	-,-	miRNA,snol	112615;208148
141	chr12	34226277	+	chr12	34236250	+	N	Y	Y	Y	Y	Y	del	na	alt-EJ	intergenic	RP11-847H18.3,RP11-	+,+	lincRNA,linc	16602;76497	intergenic	RP11-847H18.3,RP11-31	+,+	lincRNA,linc	26575;66524
142	chr12	41183195	+	chr12	41183502	+	Y	Y	Y	Y	Y	Y	del	na	NHEJ	intronic	CNTN1	+	protein_cod	0	intronic	CNTN1	+	protein_cod	0
143	chr12	42299572	+	chr12	42302112	+	N	Y	Y	N	Y	N	del	na	NHEJ	ncRNA_intr	RP11-630C16.2	-	lincRNA	0	ncRNA_intr	RP11-630C16.2	-	lincRNA	0
144	chr12	49470058	+	chr12	49470532	+	N	N	Y	Y	Y	Y	del	na	alt-EJ	intergenic	RHEBL1,DHH	-,-	protein_cod	6250;13146	intergenic	RHEBL1,DHH	-,-	protein_cod	6724;12672
145	chr12	60521842	+	chr12	60525039	+	Y	Y	Y	Y	Y	Y	del	na	alt-EJ	intergenic	RP11-316A16.1,RP11-	+,+	lincRNA,pse	32210;87933	intergenic	RP11-316A16.1,RP11-33	+,+	lincRNA,pse	35407;84736
146	chr12	61332513	+	chr12	61333366	+	Y	N	Y	Y	Y	Y	del	na	NHEJ	intergenic	RP11-154D9.1,RP11-4	+,-	pseudogene	519439;292427	intergenic	RP11-154D9.1,RP11-471	+,-	pseudogene	520292;291574
147	chr12	63946730	+	chr12	63946958	+	N	N	N	N	Y	N	del	na	alt-EJ	intergenic	RP11-1022B3.1,DPY1	-,-	lincRNA,pro	192913;5963	intergenic	RP11-1022B3.1,DPY19L	-,-	lincRNA,pro	193141;5735
148	chr12	72189158	+	chr12	72190574	+	Y	Y	Y	Y	Y	Y	del	na	NHEJ	ncRNA_intr	RP11-2H8.2	-	lincRNA	0	ncRNA_intr	RP11-2H8.2	-	lincRNA	0
149	chr12	74644577	+	chr12	74648056	+	Y	Y	Y	Y	Y	Y	del	na	NHEJ	ncRNA_intr	RP11-81H3.2,RP11-27	-,-	lincRNA,linc	0	ncRNA_intr	RP11-81H3.2,RP11-274	-,-	lincRNA,linc	0
150	chr12	83909386	+	chr12	83918580	+	N	N	N	Y	N	Y	del	na	alt-EJ	intergenic	RP11-87P13.2,RP11-3	+,-	lincRNA,linc	342867;145402	intergenic	RP11-87P13.2,RP11-384	+,-	lincRNA,linc	352061;136208
151	chr12	95244025	+	chr12	95244449	+	Y	Y	Y	Y	N	Y	del	na	alt-EJ	intergenic	KRT19P2,NDUFA12	+,-	pseudogene	15091;46806	intergenic	KRT19P2,NDUFA12	+,-	pseudogene	15515;46382
152	chr12	96010454	+	chr12	96012416	+	Y	Y	Y	N	Y	Y	del	na	alt-EJ	intergenic	USP44,Y_RNA	-,+	protein_cod	65188;1011	downstream	Y_RNA	+	misc_RNA	0
153	chr12	101756303	+	chr12	101756958	+	N	N	Y	N	Y	Y	del	na	NHEJ	intronic	UTP20	+	protein_cod	0	intronic	UTP20	+	protein_cod	0
154	chr12	102102408	+	chr12	102107089	+	Y	Y	Y	Y	Y	Y	del	na	alt-EJ	intronic	CHPT1	+	protein_cod	0	intronic	CHPT1	+	protein_cod	0
155	chr12	102275968	+	chr12	102277175	+	N	Y	Y	Y	N	N	del	na	alt-EJ	intronic	DRAM1	+	protein_cod	0	intronic	DRAM1	+	protein_cod	0
156	chr12	129192676	+	chr12	129193326	+	N	N	Y	N	N	N	del	na	alt-EJ	downstream	TMEM132C	+	protein_cod	0	downstream	TMEM132C	+	protein_cod	0
157	chr12	129192745	+	chr12	129193257	+	N	N	N	N	Y	N	del	na	alt-EJ	downstream	TMEM132C	+	protein_cod	0	downstream	TMEM132C	+	protein_cod	0
158	chr12	129572487	+	chr12	129574127	+	Y	N	N	N	Y	N	del	na	alt-EJ	intronic	TMEM132D	-	protein_cod	0	intronic;intro	TMEM132D	-	protein_cod	0
159	chr12	129572714	+	chr12	129574127	+	N	N	N	N	N	Y	del	na	alt-EJ	intronic	TMEM132D	-	protein_cod	0	intronic;intro	TMEM132D	-	protein_cod	0
160	chr12	129573286	+	chr12	129574187	+	N	N	Y	N	N	N	del	na	alt-EJ	intronic	TMEM132D	-	protein_cod	0	intronic;intro	TMEM132D	-	protein_cod	0
161	chr12	129573437	+	chr12	129574187	+	N	N	N	Y	N	N	del	na	alt-EJ	intronic	TMEM132D	-	protein_cod	0	intronic;intro	TMEM132D	-	protein_cod	0
162	chr13	20201135	+	chr13	23580876	+	N	N	N	N	N	Y	del	na	alt-EJ	intergenic	LINC00350,MPHOSPH	+,+	lincRNA,pro	38645;6653	intergenic	AL157931.1,HMGA1P6	-,+	miRNA,pse	28740;127437

163	chr13	20825483	+	chr13	20828795	+	Y	Y	Y	Y	Y	Y	del	na	alt-EJ	intergenic	GJB6,CRYL1	-,-	protein_cod	18949;152323	intergenic	GJB6,CRYL1	-,-	protein_cod	22261;149011
164	chr13	21729289	+	chr13	21729832	+	Y	Y	Y	Y	Y	Y	del	na	NHEJ	exonic	SKA3	-	protein_cod	0	exonic	SKA3	-	protein_cod	0
165	chr13	21729951	+	chr13	21732062	+	N	N	N	Y	N	N	del	na	alt-EJ	splicing	SKA3	-	protein_cod	0	exonic	SKA3	-	protein_cod	0
166	chr13	21732263	+	chr13	21734040	+	N	Y	Y	Y	Y	Y	del	na	alt-EJ	exonic	SKA3	-	protein_cod	0	splicing	SKA3	-	protein_cod	0
167	chr13	21736015	+	chr13	21742128	+	Y	Y	Y	Y	Y	Y	del	na	alt-EJ	splicing	SKA3	-	protein_cod	0	exonic	SKA3	-	protein_cod	0
168	chr13	21742539	+	chr13	21746479	+	Y	Y	Y	Y	Y	Y	del	na	alt-EJ	splicing	SKA3	-	protein_cod	0	exonic	SKA3	-	protein_cod	0
169	chr13	21746643	+	chr13	21750514	+	Y	Y	Y	Y	Y	Y	del	na	alt-EJ	exonic	SKA3	-	protein_cod	0	exonic	SKA3	-	protein_cod	0
170	chr13	36532283	+	chr13	36532967	+	N	N	Y	N	N	N	del	na	alt-EJ	intronic	DCLK1	-	protein_cod	0	intronic	DCLK1	-	protein_cod	0
171	chr13	37414466	+	chr13	37414787	+	Y	Y	N	Y	N	Y	del	na	alt-EJ	intergenic	RFXAP,SMAD9	+,-	protein_cod	11225;4502	intergenic	RFXAP,SMAD9	+,-	protein_cod	11546;4181
172	chr13	38071941	+	chr13	38085579	+	Y	Y	Y	Y	Y	Y	del	na	alt-EJ	intergenic	LINC01048,POSTN	-,-	lincRNA,pro	13035;64779	intergenic	LINC01048,POSTN	-,-	lincRNA,pro	26673;51141
173	chr13	39934227	+	chr13	39935525	+	N	Y	N	N	N	N	del	na	alt-EJ	intronic	LHFP	-	protein_cod	0	intronic	LHFP	-	protein_cod	0
174	chr13	46005328	+	chr13	46005665	+	N	Y	Y	Y	N	N	del	na	alt-EJ	intergenic	SLC25A30-AS1,PPIAF	+,+	lincRNA,pse	10822;4928	intergenic	SLC25A30-AS1,PPIAP25	+,+	lincRNA,pse	11159;4591
175	chr13	46005419	+	chr13	46005757	+	N	N	N	N	Y	N	del	na	alt-EJ	intergenic	SLC25A30-AS1,PPIAF	+,+	lincRNA,pse	10913;4837	intergenic	SLC25A30-AS1,PPIAP25	+,+	lincRNA,pse	11251;4499
176	chr13	51069348	+	chr13	51075081	+	Y	Y	Y	Y	Y	Y	del	na	alt-EJ	intergenic	AL138696.1,RP11-175	-,-	miRNA,lincF	131975;25721	intergenic	AL138696.1,RP11-175B1	-,-	miRNA,lincF	137708;19988
177	chr13	55941180	+	chr13	55941471	+	N	N	Y	Y	Y	N	del	na	alt-EJ	intergenic	MIR5007,HNF4GP1	+,-	miRNA,pseu	192497;632154	intergenic	MIR5007,HNF4GP1	+,-	miRNA,pseu	192788;631863
178	chr13	57787109	+	chr13	57788381	+	Y	Y	Y	Y	Y	Y	del	na	alt-EJ	intergenic	MTCO2P3,SLC25A5P4	+,+	pseudogene	8176;101764	intergenic	MTCO2P3,SLC25A5P4	+,+	pseudogene	9448;100492
179	chr13	63569057	+	chr13	63570110	+	N	Y	N	N	Y	Y	del	na	alt-EJ	intergenic	LINC00448,LINC0037€	-,-	lincRNA,linc	262565;188177	intergenic	LINC00448,LINC00376	-,-	lincRNA,linc	263618;187124
180	chr13	65637808	+	chr13	65638211	+	Y	Y	Y	Y	Y	Y	del	na	NHEJ	intergenic	LGMNP1,STARP1	-,-	pseudogene	104280;246179	intergenic	LGMNP1,STARP1	-,-	pseudogene	104683;245776
181	chr13	71197946	+	chr13	71199993	+	Y	Y	Y	Y	Y	Y	del	na	NHEJ	intergenic	RNU6-54P,SOGA2P1	+,+	snRNA,pseu	164244;299850	intergenic	RNU6-54P,SOGA2P1	+,+	snRNA,pseu	166291;297803
182	chr13	73721884	+	chr13	73724479	+	Y	Y	Y	Y	N	Y	del	na	alt-EJ	intergenic	AL354720.1,RNU4-10F	-,+	miRNA,snR	22937;43781	intergenic	AL354720.1,RNU4-10P	-,+	miRNA,snR	25532;41186
183	chr13	78997630	+	chr13	78998741	+	N	Y	Y	N	N	N	del	na	alt-EJ	ncRNA_intr	RNF219-AS1	+	antisense	0	ncRNA_intr	RNF219-AS1	+	antisense	0
184	chr13	90862853	+	chr13	90864809	+	Y	Y	Y	Y	Y	Y	del	na	alt-EJ	intergenic	FAR1P1,KRT18P27	+,+	pseudogene	38800;19785	intergenic	FAR1P1,KRT18P27	+,+	pseudogene	40756;17829
185	chr13	94040793	+	chr13	94045539	+	Y	Y	Y	Y	Y	N	del	na	NHEJ	intronic	GPC6	+	protein_cod	0	intronic	GPC6	+	protein_cod	0
186	chr13	101894144	+	chr13	101896426	+	Y	Y	Y	Y	Y	Y	del	na	alt-EJ	intronic	NALCN	-	protein_cod	0	intronic	NALCN	-	protein_cod	0
187	chr13	104091764	+	chr13	104092247	+	Y	Y	Y	Y	Y	Y	del	na	NHEJ	intergenic	RP11-123H22.1,ATP6V	+,-	lincRNA,pse	11729;5206	intergenic	RP11-123H22.1,ATP6V1	+,-	lincRNA,pse	12212;4723
188	chr13	112932527	+	chr13	112943006	+	N	Y	N	N	N	N	del	na	alt-EJ	intergenic	LINC01070,LINC0104	+,+	lincRNA,linc	77213;35254	intergenic	LINC01070,LINC01043	+,+	lincRNA,linc	87692;24775
189	chr13	112932721	+	chr13	112942987	+	N	N	N	Y	N	N	del	na	alt-EJ	intergenic	LINC01070,LINC0104	+,+	lincRNA,linc	77407;35060	intergenic	LINC01070,LINC01043	+,+	lincRNA,linc	87673;24794
190	chr13	114219610	+	chr13	114220063	+	N	N	N	N	N	Y	del	na	alt-EJ	intergenic	TMCO3,TFDP1	+,+	protein_cod	15068;19403	intergenic	TMCO3,TFDP1	+,+	protein_cod	15521;18950
191	chr14	22881666	+	chr14	22882260	+	Y	Y	Y	Y	Y	Y	del	na	NHEJ	ncRNA_intr	AE000661.37	-	processed_	0	ncRNA_intr	AE000661.37	-	processed_	0
192	chr14	23922248	+	chr14	23922649	+	N	Y	Y	Y	Y	Y	del	na	alt-EJ	intergenic	MYH7,NGDN	-,+	protein_cod	17321;16649	intergenic	MYH7,NGDN	-,+	protein_cod	17722;16248
193	chr14	25262335	+	chr14	25263126	+	Y	Y	Y	Y	Y	Y	del	na	alt-EJ	intergenic	RP11-104E19.1,STXBI	+,-	antisense,pı	135355;16527	intergenic	RP11-104E19.1,STXBP6	+,-	antisense,pı	136146;15736
194	chr14	28869844	+	chr14	28872162	+	Y	Y	Y	Y	Y	Y	del	na	alt-EJ	ncRNA_intr	CTD-2591A6.2	+	lincRNA	0	ncRNA_intr	CTD-2591A6.2	+	lincRNA	0
195	chr14	31285720	+	chr14	31298988	+	Y	N	N	N	N	N	del	na	alt-EJ	ncRNA_intr	RP11-159L20.2	-	antisense	0	intergenic	RP11-159L20.2,COCH	-,+	antisense,pı	7080;44761
196	chr14	37631608	+	chr14	37771229	+	N	N	N	N	Y	N	del	na	alt-EJ	intronic	SLC25A21	-	protein_cod	0	intronic	MIPOL1	+	protein_cod	0
197	chr14	39004518	+	chr14	39005028	+	Y	Y	Y	Y	Y	N	del	na	NHEJ	intergenic	RP11-96D24.1,RP11-1	+,+	lincRNA,pse	223384;69941	intergenic	RP11-96D24.1,RP11-14N	+,+	lincRNA,pse	223894;69431
198	chr14	45053196	+	chr14	45053821	+	N	N	N	N	N	Y	del	na	alt-EJ	intergenic	snoU13,RP11-398E10.	-,-	snoRNA,linc	16987;179164	intergenic	snoU13,RP11-398E10.1	-,-	snoRNA,linc	17612;178539
199	chr14	50540127	+	chr14	50541667	+	Y	Y	Y	Y	Y	Y	del	na	alt-EJ	ncRNA_intr	RP11-58E21.5,RP11-5	-,-	lincRNA,linc	0	ncRNA_intr	RP11-58E21.5,RP11-58E	-,-	lincRNA,linc	0
200	chr14	51408901	+	chr14	51409546	+	Y	Y	Y	Y	Y	Y	del	na	alt-EJ	intronic	PYGL	-	protein_cod	0	intronic	PYGL	-	protein_cod	0
201	chr14	53252466	+	chr14	53255080	+	N	N	Y	Y	Y	Y	del	na	NHEJ	ncRNA_exo	RN7SL588P	+	misc_RNA	0	intronic	GNPNAT1	-	protein_cod	0
202	chr14	53252709	+	chr14	53254971	+	Y	N	N	N	N	N	del	na	alt-EJ	intronic	GNPNAT1	-	protein_cod	0	intronic	GNPNAT1	-	protein_cod	0
203	chr14	54710347	+	chr14	54713687	+	Y	Y	Y	Y	Y	Y	del	na	alt-EJ	intergenic	ATP5C1P1,CDKN3	-,+	pseudogene	251634;153326	intergenic	ATP5C1P1,CDKN3	-,+	pseudogene	254974;149986
204	chr14	59883478	+	chr14	59884079	+	Y	Y	Y	Y	Y	Y	del	na	NHEJ	intergenic	AL159140.1,GPR135	+,-	miRNA,prot	16609;12262	intergenic	AL159140.1,GPR135	+,-	miRNA,prot	17210;11661
205	chr14	63621574	+	chr14	63621869	+	N	Y	N	N	Y	N	del	na	alt-EJ	intergenic	RP11-1112J20.2,RHOJ	-,+	lincRNA,pro	26642;49258	intergenic	RP11-1112J20.2,RHOJ	-,+	lincRNA,pro	26937;48963
206	chr14	73953750	+	chr14	73954051	+	N	N	Y	N	N	N	del	na	alt-EJ	intronic	HEATR4	-	protein_cod	0	intronic	HEATR4	-	protein_cod	0
207	chr14	83203928	+	chr14	83206864	+	N	Y	Y	Y	Y	Y	del	na	NHEJ	intergenic	RP11-406A9.2,RP11-1	+,-	lincRNA,linc	30759;50894	intergenic	RP11-406A9.2,RP11-11K	+,-	lincRNA,linc	33695;47958
208	chr14	83852040	+	chr14	83852364	+	N	Y	Y	Y	Y	Y	del	na	NHEJ	intergenic	RNU7-51P,RNU6ATAC	+,+	snRNA,snR	337056;364688	intergenic	RNU7-51P,RNU6ATAC28	+,+	snRNA,snR	337380;364364
209	chr14	85297112	+	chr14	85302160	+	Y	Y	Y	Y	Y	Y	del	na	alt-EJ	intergenic	RNU6-976P,RP11-688I	+,+	snRNA,pseu	90716;120506	intergenic	RNU6-976P,RP11-688D1	+,+	snRNA,pseu	95764;115458
210	chr14	93651375	+	chr14	93652603	+	N	Y	N	N	N	Y	del	na	alt-EJ	UTR5	TMEM251,RP11-371E8	+,+	protein_cod	0	splicing	TMEM251,RP11-371E8.4	+,+	protein_cod	0
211	chr14	98783991	+	chr14	98785099	+	Y	Y	Y	Y	Y	Y	del	na	alt-EJ	intergenic	RP11-61O1.1,AL1637€	-,-	lincRNA,miF	112511;13978	intergenic	RP11-61O1.1,AL163760.	-,-	lincRNA,miF	113619;12870
212	chr14	103575504	+	chr14	103575865	+	N	N	N	N	N	Y	del	na	alt-EJ	intronic	EXOC3L4	+	protein_cod	0	intronic	EXOC3L4	+	protein_cod	0
213	chr14	103575598	+	chr14	103576055	+	Y	N	N	N	N	N	del	na	alt-EJ	intronic	EXOC3L4	+	protein_cod	0	intronic	EXOC3L4	+	protein_cod	0
214	chr14	106082667	+	chr14	106227334	+	N	N	Y	Y	N	N	del	na	alt-EJ	intergenic	RP11-731F5.1,IGHG4	-,-	lincRNA,IG_	10973;8020	intergenic	IGHG1,IGHG3	-,-	IG_C_gene,	17926;8105
215	chr14	106449027	+	chr14	106450849	+	Y	Y	Y	Y	Y	Y	del	na	NHEJ	intergenic	AB019441.29,IGHV1-2	-,-	pseudogene	3794;3644	intergenic	AB019441.29,IGHV1-2	-,-	pseudogene	5616;1822
216	chr14	106932638	+	chr14	107174929	+	N	N	N	Y	N	Y	del	na	alt-EJ	intergenic	IGHVII-43-1,IGHVIII-44	-,-	IG_V_pseu	3750;1588	intergenic	IGHV1-69,IGHV2-70	-,-	IG_V_gene,	4501;3891
217	chr15	22373078	+	chr15	22383653	+	Y	Y	Y	Y	Y	Y	del	na	NHEJ	ncRNA_intr	RP11-69H14.6	+	sense_overl	0	ncRNA_exo	RP11-69H14.6	+	sense_over	0
218	chr15	23671483	+	chr15	23675291	+	N	N	N	Y	Y	N	del	na	alt-EJ	intergenic	GOLGA8S,RP11-529J	+,+	protein_cod	61202;4503	upstream	RP11-529J17.3	+	pseudogene	0
219	chr15	25931396	+	chr15	25932381	+	Y	Y	Y	Y	Y	Y	del	na	NHEJ	intronic	ATP10A	-	protein_cod	0	intronic	ATP10A	-	protein_cod	0
220	chr15	29411478	+	chr15	29411901	+	Y	Y	Y	Y	Y	Y	del	na	alt-EJ	downstream	APBA2,FAM189A1	+,-	protein_cod	0	downstream	FAM189A1	-	protein_cod	0
221	chr15	39744386	+	chr15	39744856	+	Y	Y	Y	Y	Y	Y	del	na	alt-EJ	intergenic	RP11-624L4.1,RP11-6	-,+	antisense,lir	24990;20518	intergenic	RP11-624L4.1,RP11-624	-,+	antisense,lir	25460;20048
222	chr15	41851540	+	chr15	41853322	+	N	Y	N	Y	Y	N	del	na	alt-EJ	exonic;exon	TYRO3	+	protein_cod	0	intronic;intrc	TYRO3	+	protein_cod	0
223	chr15	41864762	+	chr15	41865200	+	N	N	Y	N	N	N	del	na	NHEJ	exonic;exon	TYRO3	+	protein_cod	0	exonic	TYRO3	+	protein_cod	0
224	chr15	41865309	+	chr15	41865506	+	N	N	N	Y	N	N	del	na	NHEJ	exonic	TYRO3	+	protein_cod	0	exonic	TYRO3	+	protein_cod	0
225	chr15	41866014	+	chr15	41870085	+	Y	N	Y	Y	Y	N	del	na	alt-EJ	splicing	TYRO3	+	protein_cod	0	exonic	TYRO3	+	protein_cod	0
226	chr15	42782361	+	chr15	42782759	+	Y	Y	Y	Y	Y	Y	del	na	alt-EJ	intronic	ZNF106	-	protein_cod	0	intronic	ZNF106	-	protein_cod	0
227	chr15	48396500	+	chr15	48400278	+	Y	Y	Y	Y	Y	Y	del	na	alt-EJ	intergenic	RP11-208K4.1,SLC24/	+,+	lincRNA,pro	55191;16669	intergenic	RP11-208K4.1,SLC24A5	+,+	lincRNA,pro	58969;12891
228	chr15	55655533	+	chr15	55657059	+	Y	Y	Y	Y	Y	Y	del	na	alt-EJ	ncRNA_intr	DYX1C1-CCPG1	-	processed_	0	ncRNA_intr	DYX1C1-CCPG1	-	processed_	0
229	chr15	56789862	+	chr15	56801002	+	Y	Y	N	Y	Y	Y	del	na	alt-EJ	intergenic	MNS1,RP11-1129I3.1	-,-	protein_cod	32527;45288	intergenic	MNS1,RP11-1129I3.1	-,-	protein_cod	43667;34148
230	chr15	62706093	+	chr15	62707794	+	Y	Y	Y	Y	Y	Y	del	na	alt-EJ	intergenic	HMGN1P26,TLN2	-,+	pseudogene	51563;147471	intergenic	HMGN1P26,TLN2	-,+	pseudogene	53264;145770
231	chr15	79220652	+	chr15	79233878	+	Y	Y	Y	N	Y	Y	del	na	alt-EJ	intronic;intrc	CTSH	-	protein_cod	0	intronic;intrc	CTSH	-	protein_cod	0
232	chr15	86396674	+	chr15	86397216	+	N	Y	N	Y	Y	Y	del	na	alt-EJ	intergenic	MIR548AP,RP11-23A2	+,-	miRNA,pseu	27713;105274	intergenic	MIR548AP,RP11-23A22.1	+,-	miRNA,pseu	28255;104732
233	chr15	100686977	+	chr15	100693183	+	Y	Y	N	Y	Y	Y	del	na	alt-EJ	intronic	ADAMTS17	-	protein_cod	0	intronic	ADAMTS17	-	protein_cod	0
234	chr15	101341488	+	chr15	101342372	+	Y	Y	Y	Y	Y	Y	del	na	alt-EJ	ncRNA_intr	RP11-66B24.5	+	lincRNA	0	ncRNA_intr	RP11-66B24.5	+	lincRNA	0
235	chr16	3918273	+	chr16	3918502	+	N	N	Y	N	N	N	del	na	NHEJ	intronic	CREBBP	-	protein_cod	0	intronic	CREBBP	-	protein_cod	0
236	chr16	9770901	+	chr16	59556686	+	Y	N	N	N	N	N	del	na	alt-EJ	upstream	RP11-297M9.1	-	protein_cod	0	intergenic	AC040163.1,RNU4-58P	+,+	miRNA,snR	107937;17344
237	chr16	22019728	+	chr16	22086808	+	Y	Y	Y	Y	Y	Y	del	na	alt-EJ	exonic	C16orf52	+	protein_cod	0	exonic	C16orf52	+	protein_cod	0
238	chr16	22087020	+	chr16	22092017	+	Y	Y	Y	Y	Y	Y	del	na	NHEJ	exonic	C16orf52	+	protein_cod	0	exonic	C16orf52	+	protein_cod	0
239	chr16	31185430	+	chr16	31186568	+	N	N	Y	N	Y	Y	del	na	alt-EJ	upstream	NDUFA3P6	+	pseudogene	0	downstream	NDUFA3P6	+	pseudogene	0
240	chr16	32371596	+	chr16	32371748	+	N	N	N	Y	N	N	del	na	alt-EJ	ncRNA_intr	RP11-17M15.4	-	pseudogene	0	ncRNA_intr	RP11-17M15.4	-	pseudogene	0
241	chr16	46440091	+	chr16	46445608	+	N	N	N	Y	N	N	del	na	alt-EJ	intergenic	NONE,ANKRD26P1	NONE,-	NONE,pseu	NONE;63162	intergenic	NONE,ANKRD26P1	NONE,-	NONE,pseu	NONE;57645
242	chr16	46447345	+	chr16	49377700	+	N	N	N	Y	N	N	del	na	NHEJ	intergenic	NONE,ANKRD26P1	NONE,-	NONE,pseu	NONE;55908	intergenic	RP11-491F9.5,C16orf78	+,+	lincRNA,pro	4796;30034
243	chr16	56617026	+	chr16	56617361	+	Y	Y	Y	N	Y	Y	del	na	alt-EJ	intergenic	MT4,MT3	+,+	protein_cod	14157;5960	intergenic	MT4,MT3	+,+	protein_cod	14492;5625
244	chr16	57366311	+	chr16	57367119	+	N	N	N	N	Y	N	del	na	NHEJ	ncRNA_exo	CTC-661I16.2	-	pseudogene	0	upstream;up	CTC-661I16.2	-	pseudogene	0
245	chr16	57725395	+	chr16	57727847	+	Y	Y	Y	Y	Y	Y	del	na	NHEJ	ncRNA_intr	RP11-405F3.4	-	antisense	0	ncRNA_intr	RP11-405F3.4	-	antisense	0
246	chr16	59335844	+	chr16	59380553	+	N	N	N	N	Y	N	del	na	NHEJ	intergenic	RP11-102D18.1,AC04(	-,+	pseudogene	134788;112833	intergenic	RP11-102D18.1,AC0401(	-,+	pseudogene	179497;68124

247	chr16	59536169	+	chr16	59793000	+	N	N	N	N	Y	N	del	na	alt-EJ	intergenic	AC040163.1,RNU4-58	+,+	miRNA,snR	87420;37861	intergenic	APOOP5,RP11-430C1.1	-,+	pseudogene	4070;96257
248	chr16	85149504	+	chr16	85150093	+	N	N	Y	Y	Y	Y	del	na	alt-EJ	intergenic	FAM92B,CTC-786C10	-,+	protein_cod	3390;55378	intergenic	FAM92B,CTC-786C10.1	-,+	protein_cod	3979;54789
249	chr16	88311034	+	chr16	88312838	+	N	N	Y	N	N	N	del	na	alt-EJ	ncRNA_intr	LA16c-444G7.1	+	lincRNA	0	ncRNA_intr	LA16c-444G7.1	+	lincRNA	0
250	chr16	89967838	+	chr16	89969150	+	Y	N	Y	Y	Y	Y	del	na	alt-EJ	intronic	TCF25	+	protein_cod	0	intronic	TCF25	+	protein_cod	0
251	chr17	1913188	+	chr17	1913663	+	N	N	N	N	Y	N	del	na	alt-EJ	intronic	RTN4RL1	-	protein_cod	0	intronic	RTN4RL1	-	protein_cod	0
252	chr17	4697074	+	chr17	4701535	+	N	Y	N	N	Y	N	del	na	alt-EJ	intergenic	GLTPD2,PSMB6	+,+	protein_cod	3389;2365	intronic	PSMB6	+	protein_cod	0
253	chr17	7524422	+	chr17	7524931	+	Y	Y	Y	N	Y	N	del	na	alt-EJ	intronic	SHBG	+	protein_cod	0	intronic	SHBG	+	protein_cod	0
254	chr17	8246703	+	chr17	8247925	+	Y	N	N	N	N	N	del	na	alt-EJ	intronic	ODF4	+	protein_cod	0	intronic	ODF4	+	protein_cod	0
255	chr17	8711646	+	chr17	8713994	+	Y	Y	Y	Y	Y	N	del	na	alt-EJ	intronic	PIK3R6	-	protein_cod	0	intronic	PIK3R6	-	protein_cod	0
256	chr17	11411667	+	chr17	11412338	+	N	N	N	Y	N	N	del	na	NHEJ	intronic;intro	SHISA6	+	protein_cod	0	intronic;intro	SHISA6	+	protein_cod	0
257	chr17	15183463	+	chr17	15185720	+	Y	N	N	N	N	N	del	na	alt-EJ	upstream;up	AC005703.2	-	lincRNA	0	intergenic;in	AC005703.2,TEKT3	-,-	lincRNA,pro	2926;21408
258	chr17	15723934	+	chr17	20541155	+	Y	N	N	N	N	N	del	na	alt-EJ	intergenic	AC015922.6,AC01592	-,+	lincRNA,pse	2878;8749	intergenic	RP11-218E15.1,AC0874	-,+	pseudogene	4583;9445
259	chr17	15771553	+	chr17	15772071	+	Y	N	N	N	N	N	del	na	alt-EJ	intergenic	AC015922.5,ADORA2	+,+	pseudogene	4086;76678	intergenic	AC015922.5,ADORA2B	+,+	pseudogene	4604;76160
260	chr17	15789557	+	chr17	15793785	+	Y	Y	Y	Y	Y	Y	del	na	NHEJ	intergenic	AC015922.5,ADORA2	+,+	pseudogene	22090;58674	intergenic	AC015922.5,ADORA2B	+,+	pseudogene	26318;54446
261	chr17	17230369	+	chr17	17231246	+	Y	Y	Y	Y	Y	Y	del	na	alt-EJ	intronic	NT5M	+	protein_cod	0	intronic	NT5M	+	protein_cod	0
262	chr17	21212906	+	chr17	21221183	+	Y	Y	Y	Y	Y	Y	del	na	NHEJ	intronic	MAP2K3	+	protein_cod	0	intergenic	MAP2K3,KCNJ12	+,+	protein_cod	2631;58326
263	chr17	21321277	+	chr17	21322822	+	N	Y	N	N	N	N	del	na	alt-EJ	UTR3;UTR3	KCNJ12	+	protein_cod	0	UTR3;UTR3	KCNJ12	+	protein_cod	0
264	chr17	21334291	+	chr17	21341613	+	Y	N	N	N	N	N	del	na	alt-EJ	intergenic	KCNJ12,PDLIM1P2	+,-	protein_cod	11112;13854	intergenic	KCNJ12,PDLIM1P2	+,-	protein_cod	18434;6532
265	chr17	21556718	+	chr17	21558070	+	N	Y	Y	N	N	N	del	na	alt-EJ	intergenic	AC144838.2,AC14483	+,-	pseudogene	6739;3830	intergenic	AC144838.2,AC144838.3	+,-	pseudogene	8091;2478
266	chr17	25536546	+	chr17	25540389	+	Y	Y	Y	Y	Y	Y	del	na	alt-EJ	intergenic	TUFMP1,RP11-663N2	-,-	pseudogene	125484;28339	intergenic	TUFMP1,RP11-663N22.1	-,-	pseudogene	129327;24496
267	chr17	35778946	+	chr17	35781770	+	N	Y	N	N	N	N	del	na	alt-EJ	intronic	TADA2A	+	protein_cod	0	intronic	TADA2A	+	protein_cod	0
268	chr17	38377919	+	chr17	38378393	+	Y	Y	Y	Y	Y	Y	del	na	alt-EJ	intronic	WIPF2	+	protein_cod	0	intronic	WIPF2	+	protein_cod	0
269	chr17	43258203	+	chr17	43258445	+	N	N	N	N	Y	N	del	na	alt-EJ	intergenic	RP13-890H12.2,CTD-2	-,-	antisense,a	9252;10095	intergenic	RP13-890H12.2,CTD-20	-,-	antisense,a	9494;9853
270	chr17	47351758	+	chr17	47353062	+	Y	Y	Y	Y	N	Y	del	na	alt-EJ	intergenic	RP1-62O9.3,ZNF652	+,-	processed_	15734;14812	intergenic	RP1-62O9.3,ZNF652	+,-	processed_	17038;13508
271	chr17	48427504	+	chr17	48427889	+	Y	Y	N	N	Y	Y	del	na	alt-EJ	intronic	XYLT2	+	protein_cod	0	intronic	XYLT2	+	protein_cod	0
272	chr17	51442981	+	chr17	51447478	+	Y	Y	Y	Y	Y	Y	del	na	alt-EJ	intergenic	RP11-146P2.1,CTD-25	-,+	pseudogene	11486;68511	intergenic	RP11-146P2.1,CTD-2535	-,+	pseudogene	15983;64014
273	chr17	55687810	+	chr17	55689916	+	Y	Y	Y	Y	Y	Y	del	na	alt-EJ	intronic	MSI2	+	protein_cod	0	intronic	MSI2	+	protein_cod	0
274	chr17	57363927	+	chr17	57365718	+	N	N	N	N	N	Y	del	na	alt-EJ	intergenic	SNRPGP17,YPEL2	+,+	pseudogene	5223;45136	intergenic	SNRPGP17,YPEL2	+,+	pseudogene	7014;43345
275	chr17	57363961	+	chr17	57365746	+	Y	N	Y	N	N	N	del	na	alt-EJ	intergenic	SNRPGP17,YPEL2	+,+	pseudogene	5257;45102	intergenic	SNRPGP17,YPEL2	+,+	pseudogene	7042;43317
276	chr17	57870215	+	chr17	57871185	+	Y	Y	N	N	N	N	del	na	NHEJ	intronic	VMP1	+	protein_cod	0	intronic;intro	VMP1	+	protein_cod	0
277	chr17	57870847	+	chr17	57871185	+	N	N	N	N	N	Y	del	na	alt-EJ	intronic	VMP1	+	protein_cod	0	intronic;intro	VMP1	+	protein_cod	0
278	chr17	72729270	+	chr17	72729683	+	N	N	N	N	Y	N	del	na	alt-EJ	intronic	RAB37	+	protein_cod	0	intronic	RAB37	+	protein_cod	0
279	chr17	80929429	+	chr17	80930070	+	N	N	N	N	N	Y	del	na	alt-EJ	intronic	B3GNTL1	-	protein_cod	0	intronic	B3GNTL1	-	protein_cod	0
280	chr18	10222279	+	chr18	10223606	+	N	Y	N	N	Y	Y	del	na	alt-EJ	intergenic	RP11-419J16.1,RP11-	+,-	lincRNA,linc	77876;100850	intergenic	RP11-419J16.1,RP11-13	+,-	lincRNA,linc	79203;99523
281	chr18	14450641	+	chr18	14460493	+	N	N	Y	N	N	N	del	na	NHEJ	ncRNA_exo	LONRF2P1	-	pseudogene	0	intergenic	LONRF2P1,CXADRP3	-,-	pseudogene	9611;17461
282	chr18	21903367	+	chr18	21904599	+	Y	N	Y	Y	Y	Y	del	na	alt-EJ	intronic	OSBPL1A	-	protein_cod	0	intronic	OSBPL1A	-	protein_cod	0
283	chr18	24571620	+	chr18	24572342	+	Y	Y	Y	Y	Y	Y	del	na	NHEJ	ncRNA_intr	AQP4-AS1	+	antisense	0	ncRNA_intr	AQP4-AS1	+	antisense	0
284	chr18	24791587	+	chr18	24791872	+	Y	N	N	N	Y	Y	del	na	alt-EJ	intergenic	AQP4-AS1,RP11-57J1	+,+	antisense,li	20925;14119	intergenic	AQP4-AS1,RP11-57J16.	+,+	antisense,li	21210;13834
285	chr18	27094934	+	chr18	27102699	+	Y	Y	Y	N	N	Y	del	na	alt-EJ	intergenic	AC091321.1,RP11-784	+,+	miRNA,linc	31546;3290	ncRNA_intr	RP11-784B15.1	+	lincRNA	0
286	chr18	36716289	+	chr18	36716723	+	N	Y	N	N	Y	N	del	na	alt-EJ	intergenic	RNU6-706P,LINC0066	+,-	snRNA,linc	102215;70599	intergenic	RNU6-706P,LINC00669	+,-	snRNA,linc	102649;70165
287	chr18	38006375	+	chr18	38007002	+	Y	Y	Y	Y	Y	Y	del	na	alt-EJ	intergenic	RPL17P45,RNU7-145	-,+	pseudogene	281907;46410	intergenic	RPL17P45,RNU7-145P	-,+	pseudogene	282534;45783
288	chr18	38259892	+	chr18	38266750	+	Y	Y	Y	Y	Y	Y	del	na	NHEJ	intergenic	RNU7-145P,RP11-142	+,-	snRNA,linc	207046;787503	intergenic	RNU7-145P,RP11-142I2	+,-	snRNA,linc	213904;780645
289	chr18	49196757	+	chr18	49198894	+	Y	N	Y	N	N	N	del	na	alt-EJ	intergenic	RSL24D1P9,RP11-44	-,-	pseudogene	57277;17138	intergenic	RSL24D1P9,RP11-440L1	-,-	pseudogene	59414;15001
290	chr18	50462566	+	chr18	50463032	+	Y	Y	Y	Y	Y	Y	del	na	alt-EJ	intronic	DCC	+	protein_cod	0	intronic	DCC	+	protein_cod	0
291	chr18	54946737	+	chr18	54948718	+	Y	Y	Y	Y	Y	Y	del	na	NHEJ	intergenic	CTD-2526M8.2,RNU6-	-,-	lincRNA,sn	108312;5096	intergenic	CTD-2526M8.2,RNU6-73	-,-	lincRNA,sn	110293;3115
292	chr18	63723838	+	chr18	63732369	+	Y	Y	Y	Y	Y	Y	del	na	alt-EJ	intergenic	RP11-389J22.3,PRPF	-,-	lincRNA,pse	135660;12148	intergenic	RP11-389J22.3,PRPF19	-,-	lincRNA,pse	144191;3617
293	chr18	64006979	+	chr18	64008004	+	Y	Y	Y	Y	Y	Y	del	na	alt-EJ	intergenic	PRPF19P1,CDH19	-,-	pseudogene	270096;161341	intergenic	PRPF19P1,CDH19	-,-	pseudogene	271121;160316
294	chr18	64219705	+	chr18	64221256	+	Y	Y	Y	Y	Y	Y	del	na	alt-EJ	intronic	CDH19	-	protein_cod	0	intronic	CDH19	-	protein_cod	0
295	chr18	64786415	+	chr18	64786994	+	N	N	Y	Y	Y	Y	del	na	alt-EJ	intergenic	MIR5011,RPL31P9	+,-	miRNA,pseu	37492;18433	intergenic	MIR5011,RPL31P9	+,-	miRNA,pseu	38071;17854
296	chr18	66228957	+	chr18	66229336	+	Y	Y	Y	Y	Y	Y	del	na	NHEJ	intergenic	RP11-585K6.2,TMX3	+,-	pseudogene	33597;111968	intergenic	RP11-585K6.2,TMX3	+,-	pseudogene	33976;111589
297	chr18	66745596	+	chr18	66757002	+	Y	Y	Y	Y	Y	Y	del	na	alt-EJ	intergenic	CCDC102B,RP11-674	+,+	protein_cod	23170;133918	intergenic	CCDC102B,RP11-674P1	+,+	protein_cod	34576;122512
298	chr18	75266997	+	chr18	75268160	+	Y	Y	Y	Y	Y	Y	del	na	NHEJ	ncRNA_intr	RP11-176N18.2	+	lincRNA	0	ncRNA_intr	RP11-176N18.2	+	lincRNA	0
299	chr18	77309944	+	chr18	77312085	+	N	Y	N	N	Y	Y	del	na	alt-EJ	intergenic	NFATC1,RP11-567M1	+,+	protein_cod	20619;26516	intergenic	NFATC1,RP11-567M16.1	+,+	protein_cod	22760;24375
300	chr19	605299	+	chr19	608434	+	Y	Y	N	N	Y	N	del	na	alt-EJ	intronic;intro	HCN2	+	protein_cod	0	intronic;intro	HCN2	+	protein_cod	0
301	chr19	3173295	+	chr19	3175832	+	N	N	Y	N	N	N	del	na	alt-EJ	intergenic	GNA15,S1PR4	+,+	protein_cod	9528;5441	intergenic	GNA15,S1PR4	+,+	protein_cod	12065;2904
302	chr19	4511351	+	chr19	4511550	+	N	N	Y	N	N	N	del	na	alt-EJ	exonic	PLIN4	-	protein_cod	0	exonic	PLIN4	-	protein_cod	0
303	chr19	8243000	+	chr19	8244947	+	Y	Y	Y	Y	Y	Y	del	na	alt-EJ	intergenic	CTD-3020H12.3,CERS	+,+	pseudogene	2549;28620	intergenic	CTD-3020H12.3,CERS4	+,+	pseudogene	4496;26673
304	chr19	9222947	+	chr19	9225125	+	N	N	N	Y	N	N	del	na	alt-EJ	intergenic	OR7G2,OR7G1	-,-	protein_cod	8965;2327	downstream	OR7G1	-	protein_cod	0
305	chr19	14732337	+	chr19	14734135	+	Y	Y	Y	Y	Y	N	del	na	alt-EJ	intronic	EMR3	-	protein_cod	0	intronic	EMR3	-	protein_cod	0
306	chr19	15046391	+	chr19	15049478	+	Y	Y	Y	Y	N	Y	del	na	NHEJ	intergenic	OR7A15P,OR7C2	+,+	pseudogene	7555;5910	intergenic	OR7A15P,OR7C2	+,+	pseudogene	10642;2823
307	chr19	19834444	+	chr19	19839664	+	N	Y	Y	Y	Y	N	del	na	alt-EJ	intronic	ZNF14	-	protein_cod	0	intronic	ZNF14	-	protein_cod	0
308	chr19	21268282	+	chr19	21327851	+	Y	Y	N	N	N	Y	del	na	alt-EJ	intronic	ZNF714	+	protein_cod	0	intronic	ZNF431	+	protein_cod	0
309	chr19	21268356	+	chr19	21327845	+	N	N	N	Y	N	N	del	na	alt-EJ	intronic	ZNF714	+	protein_cod	0	intronic	ZNF431	+	protein_cod	0
310	chr19	35661080	+	chr19	35665799	+	N	Y	Y	Y	Y	Y	del	na	alt-EJ	downstream	FXYD5	+	protein_cod	0	intergenic	FXYD5,FAM187B	+,-	protein_cod	5013;49904
311	chr19	36662658	+	chr19	36662955	+	N	Y	N	Y	Y	Y	del	na	alt-EJ	intergenic	COX7A1,AC002984.2	-,-	protein_cod	18887;1650	intergenic	COX7A1,AC002984.2	-,-	protein_cod	19184;1353
312	chr19	39512015	+	chr19	39535548	+	N	N	Y	N	N	N	del	na	alt-EJ	intronic	FBXO27	-	protein_cod	0	intergenic	FBXO27,snoU13	-,-	protein_cod	12123;4997
313	chr19	46278649	+	chr19	46279626	+	Y	N	Y	N	Y	N	del	na	alt-EJ	intronic	DMPK	-	protein_cod	0	intronic	DMPK	-	protein_cod	0
314	chr19	46278656	+	chr19	46279312	+	N	N	N	N	N	Y	del	na	alt-EJ	intronic	DMPK	-	protein_cod	0	intronic	DMPK	-	protein_cod	0
315	chr19	46278994	+	chr19	46279308	+	N	N	N	Y	N	N	del	na	alt-EJ	intronic	DMPK	-	protein_cod	0	intronic	DMPK	-	protein_cod	0
316	chr19	53936267	+	chr19	53973204	+	N	N	N	Y	N	N	del	na	alt-EJ	ncRNA_intr	ZNF761,TPM3P9	+,+	processed_	0	intronic;intro	ZNF813	+	protein_cod	0
317	chr19	53936696	+	chr19	53973485	+	Y	Y	N	N	Y	Y	del	na	alt-EJ	ncRNA_intr	ZNF761,TPM3P9	+,+	processed_	0	intronic	ZNF813	+	protein_cod	0
318	chr19	55604622	+	chr19	55605716	+	Y	N	N	N	N	N	del	na	VNTR	intronic	PPP1R12C	-	protein_cod	0	exonic	PPP1R12C	-	protein_cod	0
319	chr19	56041837	+	chr19	56042174	+	N	Y	Y	N	Y	Y	del	na	alt-EJ	intronic	SBK2	-	protein_cod	0	intronic	SBK2	-	protein_cod	0
320	chr19	57474906	+	chr19	57475611	+	Y	N	Y	Y	N	Y	del	na	alt-EJ	intergenic	CTC-258N23.3,RPL7A	+,+	pseudogene	41325;101796	intergenic	CTC-258N23.3,RPL7AP	+,+	pseudogene	42030;101091
321	chr2	1052363	+	chr2	1052548	+	N	N	N	Y	N	N	del	na	alt-EJ	intronic	SNTG2	+	protein_cod	0	intronic	SNTG2	+	protein_cod	0
322	chr2	1358027	+	chr2	1358472	+	Y	Y	Y	Y	Y	N	del	na	alt-EJ	intronic	SNTG2	+	protein_cod	0	intronic	SNTG2	+	protein_cod	0
323	chr2	3184199	+	chr2	3184587	+	N	Y	N	N	N	N	del	na	alt-EJ	intergenic	AC019118.4,TSSC1	+,-	lincRNA,pro	22631;8497	intergenic	AC019118.4,TSSC1	+,-	lincRNA,pro	23019;8109
324	chr2	3628784	+	chr2	3632152	+	N	Y	Y	Y	Y	Y	del	na	alt-EJ	downstream	RPS7,SNORA73	+,+	protein_cod	0	intergenic	RPS7,COLEC11	+,+	protein_cod	3643;10393
325	chr2	11823439	+	chr2	13075603	+	N	Y	Y	Y	Y	Y	del	na	alt-EJ	ncRNA_exo	AC106875.1	+	sense_intro	0	intergenic	TRIB2,AC064875.2	+,-	protein_cod	192743;31307
326	chr2	17754092	+	chr2	17754707	+	Y	N	N	N	N	N	del	na	alt-EJ	intronic	VSNL1	+	protein_cod	0	intronic	VSNL1	+	protein_cod	0
327	chr2	27616427	+	chr2	181738277	+	Y	N	Y	N	Y	N	del	na	alt-EJ	intronic	PPM1G	-	protein_cod	0	upstream;up	FTH1P20	-	pseudogene	0
328	chr2	33224462	+	chr2	33227293	+	Y	Y	Y	Y	Y	Y	del	na	NHEJ	intronic	LTBP1	+	protein_cod	0	intronic	LTBP1	+	protein_cod	0
329	chr2	34523655	+	chr2	34525721	+	Y	Y	Y	Y	Y	Y	del	na	alt-EJ	downstream	AC009499.1	+	lincRNA	0	intergenic	AC009499.1,AC073218.1	+,+	lincRNA,linc	2901;83134
330	chr2	44467108	+	chr2	44468833	+	N	Y	Y	Y	N	Y	del	na	alt-EJ	upstream	snoU13	-	snoRNA	0	intergenic	snoU13,AC013717.3	-,+	snoRNA,pse	2033;28927

331	chr2	51224547	+	chr2	51225543	+	Y	Y	Y	Y	Y	Y	del	na	NHEJ	intronic	NRXN1	-	protein_cod	0	intronic	NRXN1	-	protein_cod	0
332	chr2	53770842	+	chr2	53771858	+	N	N	N	N	Y	N	del	na	NHEJ	intronic	GPR75-ASB3	-	protein_cod	0	intronic	GPR75-ASB3	-	protein_cod	0
333	chr2	59623256	+	chr2	59631049	+	Y	Y	Y	Y	Y	Y	del	na	NHEJ	ncRNA_intr	AC007131.2,RP11-444	-,-	lincRNA,linc	0	ncRNA_intr	AC007131.2,RP11-444A	-,-	lincRNA,linc	0
334	chr2	66505591	+	chr2	66507556	+	Y	Y	Y	Y	Y	Y	del	na	alt-EJ	intergenic	AC118345.2,AC09266	-,+	miRNA,lincF	38805;48890	intergenic	AC118345.2,AC092669.2	-,+	miRNA,lincF	40770;46925
335	chr2	76557056	+	chr2	76557291	+	N	N	N	N	N	Y	del	na	NHEJ	intergenic	RP11-335E8.1,RN7SK	-,-	pseudogene	68269;115149	intergenic	RP11-335E8.1,RN7SKP2	-,-	pseudogene	68504;114914
336	chr2	76773553	+	chr2	76775453	+	Y	Y	N	N	Y	Y	del	na	alt-EJ	intergenic;ir	RN7SKP203,RN7SKP	-,-	misc_RNA,r	101017;48986	intergenic;ir	RN7SKP203,RN7SKP16	-,-	misc_RNA,r	102917;47086
337	chr2	78117890	+	chr2	78118358	+	Y	Y	Y	Y	Y	Y	del	na	NHEJ	intergenic	AC105399.2,AC07362	+,+	pseudogene	97677;25170	intergenic	AC105399.2,AC073628.1	+,+	pseudogene	98145;24702
338	chr2	90413527	+	chr2	91634219	+	N	N	Y	N	N	N	del	na	alt-EJ	intergenic	AC113612.1,CH17-132	+,+	pseudogene	113125;44674	intergenic	RP11-685N3.1,AC01869	-,+	protein_cod	1095822;1712
339	chr2	90447804	+	chr2	90450048	+	N	Y	N	N	N	N	del	na	alt-EJ	intergenic;ir	AC113612.1,CH17-132	+,+	pseudogene	147402;10397	intergenic	AC113612.1,CH17-132F	+,+	pseudogene	149646;8153
340	chr2	90447804	+	chr2	90450238	+	N	N	Y	N	N	N	del	na	alt-EJ	intergenic;ir	AC113612.1,CH17-132	+,+	pseudogene	147402;10397	intergenic	AC113612.1,CH17-132F	+,+	pseudogene	149836;7963
341	chr2	90449641	+	chr2	91670390	+	N	N	Y	N	N	N	del	na	alt-EJ	intergenic	AC113612.1,CH17-132	+,+	pseudogene	149239;8560	intergenic	AC018696.1,IGKV1OR2-	+,+	pseudogene	34241;8697
342	chr2	91670558	+	chr2	91671000	+	N	Y	N	N	N	N	del	na	alt-EJ	intergenic	AC018696.1,IGKV1OF	+,+	pseudogene	34409;8529	intergenic	AC018696.1,IGKV1OR2-	+,+	pseudogene	34851;8087
343	chr2	91671059	+	chr2	91671211	+	N	N	Y	N	N	N	del	na	alt-EJ	intergenic	AC018696.1,IGKV1OF	+,+	pseudogene	34910;8028	intergenic	AC018696.1,IGKV1OR2-	+,+	pseudogene	35062;7876
344	chr2	91751061	+	chr2	91759768	+	Y	Y	Y	Y	Y	Y	del	na	alt-EJ	intergenic	AC018696.7,AC01869	+,+	pseudogene	10268;15476	intergenic	AC018696.7,AC018696.4	+,+	pseudogene	18975;6769
345	chr2	98385314	+	chr2	98385711	+	Y	Y	Y	Y	Y	Y	del	na	alt-EJ	intronic	TMEM131	-	protein_cod	0	intronic	TMEM131	-	protein_cod	0
346	chr2	109635158	+	chr2	109636127	+	Y	Y	Y	Y	Y	Y	del	na	NHEJ	intergenic	EDAR,SH3RF3-AS1	-,-	protein_cod	29330;108625	intergenic	EDAR,SH3RF3-AS1	-,-	protein_cod	30299;107656
347	chr2	115628105	+	chr2	115628845	+	Y	Y	Y	Y	Y	Y	del	na	alt-EJ	intronic	DPP10	+	protein_cod	0	intronic	DPP10	+	protein_cod	0
348	chr2	116974633	+	chr2	116978304	+	Y	Y	Y	Y	Y	Y	del	na	NHEJ	intergenic	DPP10,RP11-338I24.1	+,-	protein_cod	371305;470161	intergenic	DPP10,RP11-338I24.1	+,-	protein_cod	374976;466490
349	chr2	121797807	+	chr2	121798402	+	Y	Y	N	N	Y	Y	del	na	alt-EJ	downstream	Y_RNA	-	misc_RNA	0	upstream	Y_RNA	-	misc_RNA	0
350	chr2	124170256	+	chr2	124170587	+	Y	N	N	N	N	N	del	na	alt-EJ	intergenic;ir	AC062020.1,AC07340	-,-	lincRNA,linc	339199;30586	intergenic;ir	AC062020.1,AC073409.1	-,-	lincRNA,linc	339530;30255
351	chr2	127674757	+	chr2	127677274	+	Y	Y	Y	Y	Y	Y	del	na	alt-EJ	intergenic	AC114783.1,RNU7-18	+,+	protein_cod	15084;100240	intergenic	AC114783.1,RNU7-182P	+,+	protein_cod	17601;97723
352	chr2	129097893	+	chr2	129098612	+	N	N	N	N	N	Y	del	na	alt-EJ	intergenic;ir	HS6ST1,Y_RNA	-,-	protein_cod	21742;10510	intergenic	HS6ST1,Y_RNA	-,-	protein_cod	22461;9791
353	chr2	129097893	+	chr2	129098899	+	N	N	Y	N	N	N	del	na	alt-EJ	intergenic;ir	HS6ST1,Y_RNA	-,-	protein_cod	21742;10510	intergenic	HS6ST1,Y_RNA	-,-	protein_cod	22748;9504
354	chr2	129097972	+	chr2	129098916	+	N	Y	N	N	N	N	del	na	VNTR	intergenic	HS6ST1,Y_RNA	-,-	protein_cod	21821;10431	intergenic	HS6ST1,Y_RNA	-,-	protein_cod	22765;9487
355	chr2	130824120	+	chr2	130824673	+	Y	N	N	N	N	N	del	na	alt-EJ	intergenic	FAR2P1,POTEF	-,-	pseudogene	15416;6988	intergenic	FAR2P1,POTEF	-,-	pseudogene	15969;6435
356	chr2	130954926	+	chr2	130957401	+	Y	Y	N	Y	Y	Y	del	na	alt-EJ	intronic	TUBA3E	-	protein_cod	0	intergenic	TUBA3E,AC018804.6	-,+	protein_cod	1367;2484
357	chr2	132534465	+	chr2	132534928	+	Y	Y	Y	Y	Y	Y	del	na	alt-EJ	ncRNA_exo	AC103564.9	+	pseudogene	0	downstream	AC103564.9	+	pseudogene	0
358	chr2	145566463	+	chr2	153910989	+	N	N	N	N	N	Y	del	na	NHEJ	ncRNA_intr	TEX41	+	lincRNA	0	intergenic	UBQLN4P2,ATP5F1P4	+,+	pseudogene	176708;104463
359	chr2	146862620	+	chr2	146876864	+	Y	Y	Y	Y	Y	Y	del	na	alt-EJ	intergenic	AC062031.1,RNU7-2P	+,+	lincRNA,snF	173906;40104	intergenic	AC062031.1,RNU7-2P	+,+	lincRNA,snF	188150;25860
360	chr2	162332269	+	chr2	162334044	+	Y	Y	N	Y	Y	Y	del	na	alt-EJ	intergenic	AC009487.5,AHCTF1F	+,-	3prime_ove	46984;25127	intergenic	AC009487.5,AHCTF1P1	+,-	3prime_ove	48759;23352
361	chr2	166014694	+	chr2	166016746	+	Y	Y	N	Y	Y	Y	del	na	alt-EJ	intronic	SCN3A	-	protein_cod	0	intronic	SCN3A	-	protein_cod	0
362	chr2	173004267	+	chr2	173007500	+	Y	Y	Y	Y	Y	Y	del	na	alt-EJ	intergenic	AC104801.1,snoU13	+,-	lincRNA,snc	29557;16490	intergenic	AC104801.1,snoU13	+,-	lincRNA,snc	32790;13257
363	chr2	176709476	+	chr2	176709732	+	N	N	N	N	N	Y	del	na	alt-EJ	intergenic	EXTL2P1,AC016751.3	+,+	pseudogene	1446;52588	intergenic	EXTL2P1,AC016751.3	+,+	pseudogene	1702;52332
364	chr2	188421854	+	chr2	188422031	+	N	N	N	Y	N	N	del	na	alt-EJ	intronic	TFPI	-	protein_cod	0	intronic	TFPI	-	protein_cod	0
365	chr2	194689501	+	chr2	194698766	+	N	Y	Y	N	Y	N	del	na	NHEJ	intergenic	AC074290.1,RP11-764	-,+	pseudogene	94088;73590	intergenic	AC074290.1,RP11-764E	-,+	pseudogene	103353;64325
366	chr2	196214815	+	chr2	196215228	+	Y	Y	N	Y	Y	Y	del	na	alt-EJ	intergenic	AC010983.1,AC06483	-,+	lincRNA,linc	323721;98441	intergenic	AC010983.1,AC064834.1	-,+	lincRNA,linc	324134;98028
367	chr2	225292981	+	chr2	225293952	+	Y	N	N	N	N	N	del	na	NHEJ	intergenic;ir	FAM124B,CUL3	-,-	protein_cod	26179;41886	intergenic;ir	FAM124B,CUL3	-,-	protein_cod	27150;40915
368	chr2	227165425	+	chr2	227171171	+	Y	Y	N	Y	Y	Y	del	na	alt-EJ	intergenic	AC068138.1,MIR5702	-,-	lincRNA,miF	115338;358001	intergenic	AC068138.1,MIR5702	-,-	lincRNA,miF	121084;352255
369	chr2	234305842	+	chr2	234306174	+	Y	N	N	Y	N	N	del	na	alt-EJ	intronic	DGKD	+	protein_cod	0	intronic	DGKD	+	protein_cod	0
370	chr2	238453404	+	chr2	238453926	+	N	N	N	Y	N	N	del	na	alt-EJ	intronic	MLPH	+	protein_cod	0	intronic	MLPH	+	protein_cod	0
371	chr2	239500396	+	chr2	239501734	+	Y	Y	Y	Y	Y	Y	del	na	alt-EJ	intergenic	LINC01107,AC113618.	-,+	lincRNA,linc	36701;128569	intergenic	LINC01107,AC113618.1	-,+	lincRNA,linc	38039;127231
372	chr2	239779733	+	chr2	239779903	+	N	Y	N	N	N	N	del	na	VNTR	intronic	TWIST2	+	protein_cod	0	intronic	TWIST2	+	protein_cod	0
373	chr2	239779735	+	chr2	239779924	+	N	N	N	N	Y	N	del	na	VNTR	intronic	TWIST2	+	protein_cod	0	intronic	TWIST2	+	protein_cod	0
374	chr2	240564317	+	chr2	240565123	+	N	N	N	N	Y	N	del	na	alt-EJ	intergenic	AC079612.2,RP11-315	+,+	lincRNA,linc	11784;92333	intergenic	AC079612.2,RP11-315F	+,+	lincRNA,linc	12590;91527
375	chr2	241782021	+	chr2	241782917	+	N	N	N	N	N	Y	del	na	alt-EJ	intergenic	KIF1A,AGXT	-,+	protein_cod	22296;25875	intergenic	KIF1A,AGXT	-,+	protein_cod	23192;24979
376	chr2	242506103	+	chr2	242507335	+	N	N	Y	N	N	N	del	na	alt-EJ	intronic;intrc	BOK	+	protein_cod	0	intronic;intrc	BOK	+	protein_cod	0
377	chr2	242614134	+	chr2	242615736	+	Y	Y	Y	Y	Y	N	del	na	alt-EJ	downstream	ATG4B	+	protein_cod	0	intronic	DTYMK	-	protein_cod	0
378	chr20	1389144	+	chr20	1390816	+	Y	Y	Y	Y	Y	N	del	na	alt-EJ	intergenic	FKBP1A,NSFL1C	-,-	protein_cod	15338;33663	intergenic	FKBP1A,NSFL1C	-,-	protein_cod	17010;31991
379	chr20	5534546	+	chr20	5534923	+	Y	Y	Y	N	Y	Y	del	na	alt-EJ	intronic	GPCPD1	-	protein_cod	0	intronic	GPCPD1	-	protein_cod	0
380	chr20	15301732	+	chr20	15303770	+	Y	Y	Y	Y	Y	Y	del	na	alt-EJ	intronic	MACROD2	+	protein_cod	0	intronic	MACROD2	+	protein_cod	0
381	chr20	17927392	+	chr20	17979413	+	N	N	Y	N	N	N	del	na	alt-EJ	intronic	SNX5	-	protein_cod	0	intergenic	MGME1,PTMAP3	+,+	protein_cod	7648;13186
382	chr20	21286040	+	chr20	21288841	+	Y	N	N	N	Y	N	del	na	alt-EJ	intronic	XRN2	+	protein_cod	0	intronic	XRN2	+	protein_cod	0
383	chr20	21286106	+	chr20	21288788	+	N	N	Y	Y	N	Y	del	na	alt-EJ	intronic	XRN2	+	protein_cod	0	intronic	XRN2	+	protein_cod	0
384	chr20	23103195	+	chr20	23103634	+	N	Y	Y	N	N	N	del	na	alt-EJ	intergenic	CD93,LINC00656	-,-	protein_cod	36218;2510	intergenic	CD93,LINC00656	-,-	protein_cod	36657;2071
385	chr20	23103301	+	chr20	23103635	+	N	N	N	Y	Y	Y	del	na	alt-EJ	intergenic	CD93,LINC00656	-,-	protein_cod	36324;2404	intergenic	CD93,LINC00656	-,-	protein_cod	36658;2070
386	chr20	23683546	+	chr20	23746262	+	N	N	Y	N	N	N	del	na	alt-EJ	intergenic	CST4,CST2P1	-,-	protein_cod	13869;8411	intergenic	CST1,XXyac-YX60D10.1	-,+	protein_cod	14357;32516
387	chr20	23683639	+	chr20	23746169	+	Y	Y	N	Y	Y	Y	del	na	alt-EJ	intergenic	CST4,CST2P1	-,-	protein_cod	13962;8318	intergenic	CST1,XXyac-YX60D10.1	-,+	protein_cod	14264;32609
388	chr20	23699497	+	chr20	23774962	+	N	N	Y	N	N	N	del	na	alt-EJ	intergenic	CST2P1,CST1	-,-	pseudogene	7454;28693	intergenic	CST1,XXyac-YX60D10.1	-,+	protein_cod	43057;3816
389	chr20	24903698	+	chr20	24904417	+	Y	Y	N	Y	Y	Y	del	na	alt-EJ	intergenic	RP5-860P4.2,CST7	+,+	lincRNA,pro	242071;26168	intergenic	RP5-860P4.2,CST7	+,+	lincRNA,pro	242790;25449
390	chr20	26092751	+	chr20	26094316	+	Y	Y	Y	Y	Y	Y	del	na	NHEJ	ncRNA_intr	NCOR1P1	-	pseudogene	0	ncRNA_intr	NCOR1P1	-	pseudogene	0
391	chr20	26102844	+	chr20	26103502	+	N	Y	N	N	N	Y	del	na	NHEJ	intergenic;ir	NCOR1P1,RP13-329D	-,-	pseudogene	8180;10679	intergenic;ir	NCOR1P1,RP13-329D4.	-,-	pseudogene	8838;10021
392	chr20	26204613	+	chr20	26205372	+	N	Y	Y	N	Y	N	del	na	NHEJ	ncRNA_intr	MIR663A	-	processed_	0	ncRNA_intr	MIR663A	-	processed_	0
393	chr20	49248623	+	chr20	49249348	+	Y	Y	Y	Y	Y	Y	del	na	NHEJ	intronic	FAM65C	-	protein_cod	0	intronic	FAM65C	-	protein_cod	0
394	chr20	52575764	+	chr20	52576530	+	N	Y	Y	Y	Y	Y	del	na	alt-EJ	intronic	BCAS1	-	protein_cod	0	intronic	BCAS1	-	protein_cod	0
395	chr20	61372624	+	chr20	61374773	+	Y	Y	Y	Y	Y	Y	del	na	alt-EJ	intronic	NTSR1	+	protein_cod	0	intronic	NTSR1	+	protein_cod	0
396	chr21	9851249	+	chr21	9854010	+	Y	Y	Y	Y	Y	Y	del	na	alt-EJ	intergenic	MIR3687,TEKT4P2	+,-	miRNA,pseu	24986;55941	intergenic	MIR3687,TEKT4P2	+,-	miRNA,pseu	27747;53180
397	chr21	10703500	+	chr21	10703877	+	N	Y	N	N	N	N	del	na	alt-EJ	intergenic	bP-21201H5.1,IGHV1C	-,+	pseudogene	227439;159122	intergenic	bP-21201H5.1,IGHV1OR	-,+	pseudogene	227816;158745
398	chr21	10742376	+	chr21	10743290	+	N	N	Y	N	N	N	del	na	alt-EJ	intergenic	bP-21201H5.1,IGHV1C	-,+	pseudogene	266315;120246	intergenic	bP-21201H5.1,IGHV1OR	-,+	pseudogene	267229;119332
399	chr21	10783846	+	chr21	10789785	+	N	N	Y	N	N	N	del	na	alt-EJ	intergenic	bP-21201H5.1,IGHV1C	-,+	pseudogene	307785;78776	intergenic	bP-21201H5.1,IGHV1OR	-,+	pseudogene	313724;72837
400	chr21	10783848	+	chr21	10790171	+	Y	N	N	N	N	N	del	na	alt-EJ	intergenic	bP-21201H5.1,IGHV1C	-,+	pseudogene	307787;78774	intergenic	bP-21201H5.1,IGHV1OR	-,+	pseudogene	314110;72451
401	chr21	10799942	+	chr21	10807837	+	N	Y	N	N	N	N	del	na	alt-EJ	intergenic	bP-21201H5.1,IGHV1C	-,+	pseudogene	323881;62680	intergenic	bP-21201H5.1,IGHV1OR	-,+	pseudogene	331776;54785
402	chr21	10847856	+	chr21	10848024	+	N	N	N	Y	N	N	del	na	VNTR	intergenic	bP-21201H5.1,IGHV1C	-,+	pseudogene	371795;14766	intergenic	bP-21201H5.1,IGHV1OR	-,+	pseudogene	371963;14598
403	chr21	11036169	+	chr21	11036737	+	Y	Y	Y	Y	Y	Y	del	na	NHEJ	ncRNA_intr	BAGE2	-	pseudogene	0	ncRNA_intr	BAGE2	-	pseudogene	0
404	chr21	11089827	+	chr21	11092581	+	N	N	Y	N	Y	N	del	na	alt-EJ	ncRNA_intr	BAGE2	-	pseudogene	0	ncRNA_intr	BAGE2	-	pseudogene	0
405	chr21	16094579	+	chr21	16098842	+	N	N	Y	N	Y	Y	del	na	alt-EJ	intergenic	AF165138.7,AF12793	-,-	protein_cod	63437;24504	intergenic	AF165138.7,AF127936.3	-,-	protein_cod	67700;20241
406	chr21	16588379	+	chr21	16591454	+	Y	Y	Y	Y	Y	Y	del	na	alt-EJ	intergenic	AF127577.12,AJ0069	+,-	lincRNA,linc	148221;154462	intergenic	AF127577.12,AJ006998.	+,-	lincRNA,linc	151296;151387
407	chr21	18612330	+	chr21	18613635	+	Y	Y	Y	Y	Y	Y	del	na	alt-EJ	intergenic	NEK4P1,RNU6-113P	-,+	pseudogene	28665;191535	intergenic	NEK4P1,RNU6-113P	-,+	pseudogene	29970;190230
408	chr21	20494862	+	chr21	20505146	+	Y	Y	Y	Y	Y	Y	del	na	NHEJ	intergenic	AP000431.1,AP00043	-,+	lincRNA,linc	74859;7979	ncRNA_intr	AP000431.2	+	lincRNA	0
409	chr21	29713036	+	chr21	29714126	+	Y	Y	Y	Y	Y	Y	del	na	alt-EJ	intergenic	AL035610.2,AF131217	-,-	lincRNA,linc	112050;98631	intergenic	AL035610.2,AF131217.1	-,-	lincRNA,linc	113140;97541
410	chr21	30263651	+	chr21	30264210	+	N	N	N	N	N	Y	del	na	alt-EJ	intergenic	HSPD1P7,THUMPD1F	-,-	pseudogene	2057;10450	intergenic	HSPD1P7,THUMPD1P1	-,-	pseudogene	2616;9891
411	chr21	30264006	+	chr21	30264297	+	N	Y	N	N	N	N	del	na	alt-EJ	intergenic	HSPD1P7,THUMPD1F	-,-	pseudogene	2412;10095	intergenic	HSPD1P7,THUMPD1P1	-,-	pseudogene	2703;9804
412	chr21	30382883	+	chr21	37890853	+	Y	N	N	Y	N	N	del	na	alt-EJ	intronic	RWDD2B	-	protein_cod	0	ncRNA_intr	AP000695.4	+	antisense	0
413	chr21	33156996	+	chr21	33157326	+	N	Y	N	N	N	N	del	na	alt-EJ	intergenic	AP000255.6,TPT1P1	+,+	lincRNA,pse	48276;55657	intergenic	AP000255.6,TPT1P1	+,+	lincRNA,pse	48606;55327
414	chr21	43037464	+	chr21	43039080	+	Y	Y	Y	Y	Y	Y	del	na	NHEJ	intergenic	snoU13,LINC00111	-,+	snoRNA,linc	12621;61877	intergenic	snoU13,LINC00111	-,+	snoRNA,linc	14237;60261

415	chr21	44504856	+	chr21	44508509	+	N	N	Y	Y	Y	Y	del	na	alt-EJ	intergenic	CBS,U2AF1	-,-	protein_cod	7803;8210	intergenic	CBS,U2AF1	-,-	protein_cod	11456;4557
416	chr21	44970366	+	chr21	44973303	+	Y	Y	Y	Y	Y	Y	del	na	alt-EJ	intronic	HSF2BP	-	protein_cod	0	intronic	HSF2BP	-	protein_cod	0
417	chr21	47801910	+	chr21	47804106	+	N	N	Y	N	N	N	del	na	alt-EJ	intronic	PCNT	+	protein_cod	0	intronic	PCNT	+	protein_cod	0
418	chr21	47801914	+	chr21	47804583	+	Y	N	N	N	N	Y	del	na	alt-EJ	intronic	PCNT	+	protein_cod	0	intronic	PCNT	+	protein_cod	0
419	chr21	47802471	+	chr21	47804700	+	N	N	N	Y	N	N	del	na	alt-EJ	intronic	PCNT	+	protein_cod	0	intronic	PCNT	+	protein_cod	0
420	chr22	18025623	+	chr22	18026958	+	N	Y	Y	Y	Y	Y	del	na	alt-EJ	intronic	CECR2	+	protein_cod	0	intronic	CECR2	+	protein_cod	0
421	chr22	22520386	+	chr22	22752586	+	N	N	N	N	N	Y	del	na	alt-EJ	intergenic	IGLV4-60,LL22NC03-3	+,+	IG_V_gene,	3312;2296	upstream;do	IGLVVII-41-1,IGLVI-42	+,+	IG_V_pseu	0
422	chr22	22520494	+	chr22	22752604	+	N	Y	Y	Y	Y	N	del	na	alt-EJ	intergenic	IGLV4-60,LL22NC03-3	+,+	IG_V_gene,	3420;2188	upstream;do	IGLVVII-41-1,IGLVI-42	+,+	IG_V_pseu	0
423	chr22	24274143	+	chr22	24311299	+	Y	Y	Y	Y	Y	Y	del	na	alt-EJ	intergenic	AP000350.6,AP00035(	+,+	pseudogene	2382;8042	intronic	DDTL	+	protein_cod	0
424	chr22	25692203	+	chr22	25988820	+	N	N	N	N	Y	N	del	na	alt-EJ	intergenic	RP3-462D8.2,IGLL3P	-,+	lincRNA,pse	13562;22020	intronic	ADRBK2	+	protein_cod	0
425	chr22	25692330	+	chr22	25988857	+	Y	N	Y	Y	N	N	del	na	alt-EJ	intergenic	RP3-462D8.2,IGLL3P	-,+	lincRNA,pse	13689;21893	intronic	ADRBK2	+	protein_cod	0
426	chr22	37143149	+	chr22	37147876	+	Y	Y	Y	Y	N	Y	del	na	alt-EJ	intergenic	RP1-293L6.1,IFT27	+,-	antisense,pi	25632;11097	intergenic	RP1-293L6.1,IFT27	+,-	antisense,pi	30359;6370
427	chr22	37905658	+	chr22	37906196	+	Y	N	N	N	N	N	del	na	alt-EJ	intronic	CARD10	-	protein_cod	0	intronic	CARD10	-	protein_cod	0
428	chr22	37905692	+	chr22	37906171	+	N	Y	Y	Y	N	N	del	na	alt-EJ	intronic	CARD10	-	protein_cod	0	intronic	CARD10	-	protein_cod	0
429	chr22	39294106	+	chr22	39298687	+	Y	Y	Y	Y	Y	Y	del	na	alt-EJ	intergenic	CBX6,CTA-150C2.13	-,+	protein_cod	25787;23126	intergenic	CBX6,CTA-150C2.13	-,+	protein_cod	30368;18545
430	chr22	39469325	+	chr22	39469638	+	N	N	Y	N	N	N	del	na	alt-EJ	intronic	APOBEC3G	+	protein_cod	0	intronic	APOBEC3G	+	protein_cod	0
431	chr22	43676811	+	chr22	43677047	+	Y	N	N	N	N	N	del	na	alt-EJ	ncRNA_intr	Z99756.1	+	antisense	0	ncRNA_intr	Z99756.1	+	antisense	0
432	chr22	44508567	+	chr22	44511652	+	N	N	N	N	N	Y	del	na	alt-EJ	intronic	PARVB	+	protein_cod	0	intronic	PARVB	+	protein_cod	0
433	chr22	44509011	+	chr22	44510114	+	Y	N	N	N	N	N	del	na	alt-EJ	intronic	PARVB	+	protein_cod	0	intronic	PARVB	+	protein_cod	0
434	chr22	45554719	+	chr22	45557125	+	N	Y	N	Y	N	Y	del	na	alt-EJ	ncRNA_intr	CTA-217C2.1	-	lincRNA	0	ncRNA_intr	CTA-217C2.1	-	lincRNA	0
435	chr22	45723922	+	chr22	45724251	+	Y	Y	Y	Y	Y	Y	del	na	alt-EJ	exonic	FAM118A	+	protein_cod	0	intronic	FAM118A	+	protein_cod	0
436	chr22	49034177	+	chr22	49034450	+	N	N	N	N	N	Y	del	na	alt-EJ	intronic	FAM19A5	+	protein_cod	0	intronic;intro	FAM19A5	+	protein_cod	0
437	chr22	50466975	+	chr22	50467434	+	N	Y	N	N	N	N	del	na	alt-EJ	intronic	TTLL8	-	protein_cod	0	intronic	TTLL8	-	protein_cod	0
438	chr3	3481046	+	chr3	3483342	+	Y	Y	Y	Y	Y	Y	del	na	alt-EJ	ncRNA_intr	AC026188.1	-	lincRNA	0	ncRNA_intr	AC026188.1	-	lincRNA	0
439	chr3	5155855	+	chr3	5284212	+	Y	N	N	N	N	N	del	na	alt-EJ	intergenic	AC090955.5,ARL8B	+,+	pseudogene	14309;8050	intergenic	EDEM1,MIR4790	+,-	protein_cod	22570;7650
440	chr3	6169030	+	chr3	6187098	+	N	N	N	N	Y	N	del	na	alt-EJ	intergenic;ir	AC027119.1,AC02616	+,+	lincRNA,linc	3223;21141	intergenic;ir	AC027119.1,AC026167.1	+,+	lincRNA,linc	21291;3073
441	chr3	8325638	+	chr3	8326812	+	Y	Y	Y	Y	Y	Y	del	na	NHEJ	ncRNA_intr	LMCD1-AS1	-	antisense	0	ncRNA_intr	LMCD1-AS1	-	antisense	0
442	chr3	8720104	+	chr3	8721110	+	N	N	Y	N	N	N	del	na	alt-EJ	intronic	SSUH2	-	protein_cod	0	intronic	SSUH2	-	protein_cod	0
443	chr3	8720256	+	chr3	8720542	+	N	Y	N	N	Y	N	del	na	alt-EJ	intronic	SSUH2	-	protein_cod	0	intronic	SSUH2	-	protein_cod	0
444	chr3	8720287	+	chr3	8720447	+	Y	N	N	N	N	N	del	na	VNTR	intronic	SSUH2	-	protein_cod	0	intronic	SSUH2	-	protein_cod	0
445	chr3	13171015	+	chr3	124808643	+	N	N	N	N	Y	N	del	na	alt-EJ	intergenic	IQSEC1,NUP210	-,-	protein_cod	56398;186722	intronic	SLC12A8	-	protein_cod	0
446	chr3	20653871	+	chr3	20654261	+	N	N	Y	N	N	N	del	na	NHEJ	intergenic;ir	RNU6-815P,AC104441	-,-	snRNA,miR	104623;525884	intergenic;ir	RNU6-815P,AC104441.1	-,-	snRNA,miR	105013;525494
447	chr3	21723705	+	chr3	21725967	+	N	Y	Y	N	N	N	del	na	NHEJ	intronic;intro	ZNF385D	-	protein_cod	0	intronic;intro	ZNF385D	-	protein_cod	0
448	chr3	26450969	+	chr3	26452299	+	Y	Y	Y	Y	Y	Y	del	na	NHEJ	intergenic	VENTXP4,AC099754.1	-,-	pseudogene	61840;209852	intergenic	VENTXP4,AC099754.1	-,-	pseudogene	63170;208522
449	chr3	32102051	+	chr3	32107885	+	Y	Y	Y	Y	Y	Y	del	na	alt-EJ	intergenic	Y_RNA,NIFKP7	+,-	misc_RNA,p	14580;3415	ncRNA_intr	NIFKP7	-	pseudogene	0
450	chr3	32201700	+	chr3	32202164	+	N	Y	N	N	N	N	del	na	VNTR	intronic	GPD1L	+	protein_cod	0	intronic	GPD1L	+	protein_cod	0
451	chr3	45609876	+	chr3	45610277	+	N	N	Y	N	N	N	del	na	alt-EJ	intergenic	LARS2,AC099539.1	+,+	protein_cod	18963;23871	intergenic	LARS2,AC099539.1	+,+	protein_cod	19364;23470
452	chr3	47490668	+	chr3	47493442	+	Y	Y	Y	Y	Y	Y	del	na	alt-EJ	intronic	SCAP	-	protein_cod	0	intronic	SCAP	-	protein_cod	0
453	chr3	48957790	+	chr3	48958731	+	N	N	N	Y	N	N	del	na	alt-EJ	intronic	ARIH2	+	protein_cod	0	intronic	ARIH2	+	protein_cod	0
454	chr3	54241168	+	chr3	54241557	+	Y	Y	Y	Y	Y	Y	del	na	NHEJ	intronic	CACNA2D3	+	protein_cod	0	intronic	CACNA2D3	+	protein_cod	0
455	chr3	56604699	+	chr3	56604956	+	N	N	N	N	N	Y	del	na	NHEJ	UTR3	CCDC66	+	protein_cod	0	intronic	CCDC66	+	protein_cod	0
456	chr3	67493701	+	chr3	67496806	+	Y	Y	Y	Y	Y	Y	del	na	alt-EJ	intronic	SUCLG2	-	protein_cod	0	intronic	SUCLG2	-	protein_cod	0
457	chr3	68634771	+	chr3	68640255	+	N	Y	Y	Y	Y	Y	del	na	alt-EJ	intergenic	FAM19A1,RP11-436J2	+,-	protein_cod	39995;48086	intergenic	FAM19A1,RP11-436J20.	+,-	protein_cod	45479;42602
458	chr3	68739682	+	chr3	68747859	+	Y	Y	Y	Y	Y	Y	del	na	NHEJ	intergenic	PSMC1P1,FAM19A4	+,-	pseudogene	53507;41235	intergenic	PSMC1P1,FAM19A4	+,-	pseudogene	61684;33058
459	chr3	69179673	+	chr3	69181542	+	N	Y	Y	Y	Y	Y	del	na	NHEJ	intergenic	LMOD3,FRMD4B	-,-	protein_cod	7490;39468	intergenic	LMOD3,FRMD4B	-,-	protein_cod	9359;37599
460	chr3	75276885	+	chr3	75278914	+	Y	Y	Y	Y	Y	Y	del	na	alt-EJ	intergenic	HNRNPA3P6,MYLKP1	+,-	pseudogene	12098;100815	intergenic	HNRNPA3P6,MYLKP1	+,-	pseudogene	14127;98786
461	chr3	80062738	+	chr3	80063977	+	Y	N	N	N	N	N	del	na	alt-EJ	intergenic	HMGB1P38,OSBPL9P	-,+	pseudogene	55446;149739	intergenic	HMGB1P38,OSBPL9P1	-,+	pseudogene	56685;148500
462	chr3	80062780	+	chr3	80064368	+	N	N	N	Y	N	N	del	na	alt-EJ	intergenic	HMGB1P38,OSBPL9P	-,+	pseudogene	55488;149697	intergenic	HMGB1P38,OSBPL9P1	-,+	pseudogene	57076;148109
463	chr3	80062834	+	chr3	80063968	+	N	Y	N	N	N	Y	del	na	alt-EJ	intergenic;ir	HMGB1P38,OSBPL9P	-,+	pseudogene	55542;149643	intergenic	HMGB1P38,OSBPL9P1	-,+	pseudogene	56676;148509
464	chr3	80062834	+	chr3	80064088	+	N	N	Y	N	N	N	del	na	alt-EJ	intergenic;ir	HMGB1P38,OSBPL9P	-,+	pseudogene	55542;149643	intergenic	HMGB1P38,OSBPL9P1	-,+	pseudogene	56796;148389
465	chr3	84104942	+	chr3	84107385	+	Y	Y	Y	Y	Y	Y	del	na	NHEJ	intergenic	SRRM1P2,AC108696.	+,-	pseudogene	117555;234321	intergenic	SRRM1P2,AC108696.1	+,-	pseudogene	119998;231878
466	chr3	86394262	+	chr3	86430244	+	N	Y	Y	N	Y	Y	del	na	alt-EJ	intergenic	RP11-789F5.1,RN7SK	-,+	lincRNA,mis	77751;17020	intergenic	RN7SKP284,RP11-331K	+,+	misc_RNA,l	18620;100849
467	chr3	98136828	+	chr3	98138387	+	N	N	Y	N	Y	Y	del	na	NHEJ	ncRNA_intr	RP11-325B23.2	+	lincRNA	0	ncRNA_intr	RP11-325B23.2	+	lincRNA	0
468	chr3	98899061	+	chr3	98902391	+	Y	Y	Y	Y	Y	Y	del	na	alt-EJ	intergenic	CTD-2021J15.1,ACTG	+,-	antisense,p	161655;35007	intergenic	CTD-2021J15.1,ACTG1F	+,-	antisense,p	164985;31677
469	chr3	99628782	+	chr3	99629632	+	N	N	Y	N	N	N	del	na	alt-EJ	intronic;intro	FILIP1L,CMSS1	-,+	protein_cod	0	intronic;intro	FILIP1L,CMSS1	-,+	protein_cod	0
470	chr3	100521629	+	chr3	100522508	+	Y	Y	Y	Y	Y	Y	del	na	NHEJ	intronic	ABI3BP	-	protein_cod	0	intronic	ABI3BP	-	protein_cod	0
471	chr3	107037955	+	chr3	107040334	+	Y	Y	Y	Y	Y	Y	del	na	alt-EJ	ncRNA_intr	RP11-446H18.5,LINC0	-,+	lincRNA,linc	0	ncRNA_intr	RP11-446H18.5,LINC008	-,+	lincRNA,linc	0
472	chr3	109103301	+	chr3	109104948	+	Y	Y	Y	Y	Y	Y	del	na	alt-EJ	intergenic	RP11-397K18.1,RP11-	-,+	pseudogene	20220;12162	intergenic	RP11-397K18.1,RP11-70	-,+	pseudogene	21867;10515
473	chr3	118155992	+	chr3	118156541	+	Y	Y	Y	Y	Y	Y	del	na	alt-EJ	ncRNA_intr	RP11-384F7.1	-	lincRNA	0	ncRNA_intr	RP11-384F7.1	-	lincRNA	0
474	chr3	119893984	+	chr3	119894445	+	Y	Y	Y	Y	Y	Y	del	na	alt-EJ	intronic	GPR156	-	protein_cod	0	intronic	GPR156	-	protein_cod	0
475	chr3	124936295	+	chr3	124937164	+	Y	Y	Y	Y	Y	Y	del	na	NHEJ	intronic	SLC12A8	-	protein_cod	0	intronic	SLC12A8	-	protein_cod	0
476	chr3	129763381	+	chr3	129806747	+	Y	N	N	Y	Y	Y	del	na	alt-EJ	intergenic	OR7E21P,RP11-77P16	-,-	pseudogene	8999;11186	ncRNA_intr	ALG1L2	+	pseudogene	0
477	chr3	129763552	+	chr3	129806252	+	N	N	N	N	N	N	del	na	alt-EJ	intergenic	OR7E21P,RP11-77P16	-,-	pseudogene	9170;11015	ncRNA_intr	ALG1L2	+	pseudogene	0
478	chr3	131708252	+	chr3	131713386	+	N	Y	N	N	N	N	del	na	alt-EJ	intronic;intro	CPNE4	-	protein_cod	0	intronic;intro	CPNE4	-	protein_cod	0
479	chr3	131708253	+	chr3	131713136	+	N	N	Y	N	Y	N	del	na	alt-EJ	intronic;intro	CPNE4	-	protein_cod	0	intronic	CPNE4	-	protein_cod	0
480	chr3	131708253	+	chr3	131713385	+	Y	N	N	Y	N	N	del	na	alt-EJ	intronic;intro	CPNE4	-	protein_cod	0	intronic	CPNE4	-	protein_cod	0
481	chr3	131708358	+	chr3	131713386	+	N	N	N	N	N	Y	del	na	alt-EJ	intronic	CPNE4	-	protein_cod	0	intronic;intro	CPNE4	-	protein_cod	0
482	chr3	140544405	+	chr3	140547045	+	Y	Y	Y	Y	Y	Y	del	na	alt-EJ	intergenic	TRIM42,RP11-691G17	+,-	protein_cod	124413;39512	intergenic	TRIM42,RP11-691G17.1	+,-	protein_cod	127053;36872
483	chr3	147606663	+	chr3	147607241	+	Y	Y	N	Y	N	Y	del	na	NHEJ	intergenic	RP11-78O22.1,AC092	-,-	lincRNA,miF	42219;22530	intergenic	RP11-78O22.1,AC0929	-,-	lincRNA,miF	42797;21952
484	chr3	162512133	+	chr3	162626335	+	Y	Y	Y	Y	Y	Y	del	na	NHEJ	intergenic	RP13-526J3.1,RP11-1	+,+	pseudogene	192553;232051	intergenic	RP13-526J3.1,RP11-10C	+,+	pseudogene	306755;117849
485	chr3	162764901	+	chr3	162769085	+	Y	Y	Y	Y	Y	Y	del	na	NHEJ	ncRNA_intr	RP11-10O22.1	+	lincRNA	0	ncRNA_intr	RP11-10O22.1	+	lincRNA	0
486	chr3	168738455	+	chr3	168738837	+	Y	Y	Y	Y	Y	Y	del	na	alt-EJ	intergenic	RP11-152C17.1,MECO	+,-	lincRNA,pro	15201;62832	intergenic	RP11-152C17.1,MECOM	+,-	lincRNA,pro	15583;62450
487	chr3	186581024	+	chr3	186585294	+	Y	Y	Y	Y	Y	Y	del	na	alt-EJ	intergenic	ADIPOQ,RPS20P14	+,+	protein_cod	4772;36963	intergenic	ADIPOQ,RPS20P14	+,+	protein_cod	9042;32693
488	chr3	189363421	+	chr3	189370901	+	Y	Y	Y	Y	Y	Y	del	na	alt-EJ	intronic	TP63	+	protein_cod	0	intronic	TP63	+	protein_cod	0
489	chr3	192744354	+	chr3	192744895	+	Y	Y	Y	Y	Y	Y	del	na	alt-EJ	intergenic	MB21D2,RP11-143P4.	-,-	protein_cod	108404;117899	intergenic	MB21D2,RP11-143P4.2	-,-	protein_cod	108945;117358
490	chr3	192875330	+	chr3	192885407	+	Y	Y	Y	Y	Y	Y	del	na	alt-EJ	ncRNA_intr	RP11-143P4.2	-	lincRNA	0	ncRNA_intr	RP11-143P4.2	-	lincRNA	0
491	chr3	194384122	+	chr3	194384515	+	Y	Y	Y	Y	Y	Y	del	na	alt-EJ	intronic	LSG1	-	protein_cod	0	intronic	LSG1	-	protein_cod	0
492	chr3	195225020	+	chr3	195230886	+	N	Y	N	N	N	N	del	na	alt-EJ	intergenic	ACAP2,PPP1R2	-,-	protein_cod	61213;16201	intergenic	ACAP2,PPP1R2	-,-	protein_cod	67079;10335
493	chr3	195569216	+	chr3	195569933	+	Y	Y	Y	Y	Y	N	del	na	NHEJ	ncRNA_intr	AC124944.5	-	lincRNA	0	ncRNA_intr	AC124944.5	-	lincRNA	0
494	chr3	197111517	+	chr3	197118216	+	N	N	N	Y	N	N	del	na	alt-EJ	intergenic	snoU13,AC128709.3	-,-	snoRNA,linc	45095;60415	intergenic	snoU13,AC128709.3	-,-	snoRNA,linc	51794;53716
495	chr3	197111717	+	chr3	197112139	+	N	N	N	N	N	Y	del	na	alt-EJ	intergenic	snoU13,AC128709.3	-,-	snoRNA,linc	45295;60215	intergenic	snoU13,AC128709.3	-,-	snoRNA,linc	45717;59793
496	chr3	197111928	+	chr3	197112115	+	N	N	Y	N	N	N	del	na	alt-EJ	intergenic	snoU13,AC128709.3	-,-	snoRNA,linc	45506;60004	intergenic	snoU13,AC128709.3	-,-	snoRNA,linc	45693;59817
497	chr4	43804	+	chr4	49019	+	Y	Y	Y	Y	Y	Y	del	na	alt-EJ	intergenic	NONE,Z95704.4	NONE,-	NONE,pseu	NONE;5187	ncRNA_exo	Z95704.4	-	pseudogene	0
498	chr4	85992	+	chr4	367158	+	N	Y	N	N	N	N	del	na	alt-EJ	ncRNA_exo	Z95704.2	+	pseudogene	0	exonic	ZNF141	+	protein_cod	0

499	chr4	86050	+	chr4	367236	+	N	N	N	N	N	Y	del	na	alt-EJ	ncRNA_exo	Z95704.2	+	pseudogene	0	exonic	ZNF141	+	protein_cod	0
500	chr4	86250	+	chr4	367298	+	N	N	Y	N	N	N	del	na	alt-EJ	ncRNA_exo	Z95704.2	+	pseudogene	0	exonic	ZNF141	+	protein_cod	0
501	chr4	3613374	+	chr4	3618620	+	Y	Y	Y	Y	Y	Y	del	na	alt-EJ	intergenic	LINC00955,RP3-368B	+,+	protein_cod	21643;21382	intergenic	LINC00955,RP3-368B9.2	+,+	protein_cod	26889;16136
502	chr4	6682394	+	chr4	6685863	+	Y	Y	Y	Y	Y	Y	del	na	alt-EJ	intergenic	AC093323.3,RP11-539	+,-	lincRNA,linc	4620;6781	intergenic	AC093323.3,RP11-539L1	+,-	lincRNA,linc	8089;3312
503	chr4	7635511	+	chr4	7635853	+	N	N	Y	Y	Y	Y	del	na	NHEJ	intronic	SORCS2	+	protein_cod	0	intronic	SORCS2	+	protein_cod	0
504	chr4	9492589	+	chr4	9495006	+	N	N	Y	N	N	N	del	na	alt-EJ	intergenic	OR7E85P,UNC93B7	+,+	pseudogene	6240;2791	upstream	UNC93B7	+	pseudogene	0
505	chr4	9576977	+	chr4	9577449	+	Y	Y	Y	Y	Y	Y	del	na	NHEJ	intergenic	RP11-1396O13.2,RP1	+,-	pseudogene	11407;8589	intergenic	RP11-1396O13.2,RP11-2	+,-	pseudogene	11879;8117
506	chr4	10211263	+	chr4	10234571	+	Y	Y	Y	Y	Y	Y	del	na	alt-EJ	intergenic	AC006499.6,AC00649	-,-	pseudogene	8967;28574	intergenic	AC006499.6,AC006499.	-,-	pseudogene	32275;5266
507	chr4	19057471	+	chr4	19057970	+	N	N	N	N	N	Y	del	na	alt-EJ	intergenic;ir	RP11-608B3.1,RP11-3	-,-	lincRNA,linc	566340;116487	intergenic;ir	RP11-608B3.1,RP11-3J1	-,-	lincRNA,linc	566839;115988
508	chr4	28421590	+	chr4	28421941	+	Y	Y	Y	Y	N	Y	del	na	NHEJ	intergenic	RP11-180C1.1,RP11-1	-,+	protein_cod	17104;15481	intergenic	RP11-180C1.1,RP11-123	-,+	protein_cod	17455;15130
509	chr4	32071041	+	chr4	32074611	+	N	N	N	Y	N	N	del	na	TEI_complex	ncRNA_intr	RP11-734I18.1	+	lincRNA	0	ncRNA_intr	RP11-734I18.1	+	lincRNA	0
510	chr4	40296719	+	chr4	40297192	+	N	N	N	N	Y	N	del	na	alt-EJ	intergenic	RP11-395I6.2,AC1954	+,+	lincRNA,linc	28642;21783	intergenic	RP11-395I6.2,AC195454	+,+	lincRNA,linc	29115;21310
511	chr4	42905165	+	chr4	42905854	+	Y	Y	Y	Y	Y	Y	del	na	NHEJ	intronic	GRXCR1	+	protein_cod	0	intronic	GRXCR1	+	protein_cod	0
512	chr4	43931495	+	chr4	43931837	+	N	Y	Y	N	Y	Y	del	na	NHEJ	intergenic	RP11-663P9.2,RP11-6	+,-	pseudogene	30127;50528	intergenic	RP11-663P9.2,RP11-663	+,-	pseudogene	30469;50186
513	chr4	48661407	+	chr4	48661615	+	N	N	Y	N	N	N	del	na	alt-EJ	intronic	FRYL	-	protein_cod	0	intronic	FRYL	-	protein_cod	0
514	chr4	48661490	+	chr4	48661962	+	N	Y	N	Y	N	N	del	na	alt-EJ	intronic	FRYL	-	protein_cod	0	intronic	FRYL	-	protein_cod	0
515	chr4	49639241	+	chr4	49648129	+	N	N	Y	N	N	N	del	na	alt-EJ	intergenic	AC119751.4,NONE	-,NONE	miRNA,NON	37715;NONE	intergenic	AC119751.4,NONE	-,NONE	miRNA,NON	46603;NONE
516	chr4	58252073	+	chr4	58262328	+	Y	N	N	N	Y	N	del	na	alt-EJ	intergenic	RPS26P24,RP11-319E	-,+	pseudogene	32578;39965	intergenic	RPS26P24,RP11-319E12	-,+	pseudogene	42833;29710
517	chr4	65097099	+	chr4	65097834	+	Y	N	Y	Y	Y	Y	del	na	NHEJ	intergenic	RP11-12K22.1,TECRL	-,-	lincRNA,pro	737103;43876	intergenic	RP11-12K22.1,TECRL	-,-	lincRNA,pro	737838;43141
518	chr4	73831248	+	chr4	73831821	+	Y	Y	Y	Y	Y	Y	del	na	NHEJ	downstream	RNU4ATAC9P	+	snRNA	0	downstream	RNU4ATAC9P	+	snRNA	0
519	chr4	79480007	+	chr4	79481319	+	Y	Y	Y	Y	Y	Y	del	na	alt-EJ	intronic	ANXA3	+	protein_cod	0	intronic	ANXA3	+	protein_cod	0
520	chr4	91931556	+	chr4	91935837	+	Y	Y	Y	Y	Y	Y	del	na	NHEJ	intronic	CCSER1	+	protein_cod	0	intronic	CCSER1	+	protein_cod	0
521	chr4	106623778	+	chr4	106699929	+	Y	Y	Y	Y	Y	Y	del	na	NHEJ	intronic	INTS12	-	protein_cod	0	ncRNA_intr	RP11-45L9.1	-	antisense	0
522	chr4	106940468	+	chr4	106943267	+	Y	Y	Y	Y	Y	Y	del	na	alt-EJ	ncRNA_intr	RP11-710F7.3	-	antisense	0	ncRNA_exo	RP11-710F7.3	-	antisense	0
523	chr4	113985910	+	chr4	113986373	+	Y	Y	Y	Y	Y	Y	del	na	alt-EJ	ncRNA_intr	RP11-650J17.1	-	antisense	0	ncRNA_intr	RP11-650J17.1	-	antisense	0
524	chr4	114135100	+	chr4	114135586	+	Y	Y	Y	Y	Y	Y	del	na	alt-EJ	intronic	ANK2	+	protein_cod	0	ncRNA_spli	AC004057.1	-	pseudogene	0
525	chr4	115175126	+	chr4	115183837	+	Y	Y	Y	N	N	N	del	na	alt-EJ	intergenic	RP11-362M19.1,UGT8	+,+	pseudogene	149745;344485	intergenic	RP11-362M19.1,UGT8	+,+	pseudogene	158456;335774
526	chr4	122282250	+	chr4	122289953	+	Y	Y	Y	N	Y	Y	del	na	alt-EJ	intronic;intro	QRFPR	-	protein_cod	0	intronic	QRFPR	-	protein_cod	0
527	chr4	122282250	+	chr4	122290051	+	N	N	N	Y	N	N	del	na	alt-EJ	intronic;intro	QRFPR	-	protein_cod	0	intronic	QRFPR	-	protein_cod	0
528	chr4	128752673	+	chr4	128753245	+	Y	Y	Y	Y	Y	N	del	na	alt-EJ	intronic	HSPA4L	+	protein_cod	0	intronic	HSPA4L	+	protein_cod	0
529	chr4	134132685	+	chr4	134132823	+	N	N	Y	N	N	N	del	na	alt-EJ	intergenic	PCDH10,AC105252.1	+,-	protein_cod	16920;349326	intergenic	PCDH10,AC105252.1	+,-	protein_cod	17058;349188
530	chr4	134132690	+	chr4	134133130	+	N	N	N	Y	N	N	del	na	alt-EJ	intergenic	PCDH10,AC105252.1	+,-	protein_cod	16925;349321	intergenic	PCDH10,AC105252.1	+,-	protein_cod	17365;348881
531	chr4	138667026	+	chr4	138667828	+	Y	Y	Y	Y	Y	Y	del	na	NHEJ	intergenic	RP13-884E18.4,RP11-	-,+	lincRNA,linc	10895;61834	intergenic	RP13-884E18.4,RP11-79	-,+	lincRNA,linc	11697;61032
532	chr4	145316446	+	chr4	145319907	+	Y	Y	Y	Y	Y	N	del	na	alt-EJ	intergenic	GYPA,RP11-361D14.2	-,+	protein_cod	254542;110606	intergenic	GYPA,RP11-361D14.2	-,+	protein_cod	258003;107145
533	chr4	149990580	+	chr4	149992580	+	Y	Y	Y	Y	N	N	del	na	alt-EJ	intergenic	RP11-563M4.1,RP11-3	-,-	lincRNA,linc	6906;78374	intergenic	RP11-563M4.1,RP11-342	-,-	lincRNA,linc	8906;76374
534	chr4	169619457	+	chr4	169621230	+	N	Y	Y	Y	Y	Y	del	na	alt-EJ	intronic	PALLD	+	protein_cod	0	exonic	PALLD	+	protein_cod	0
535	chr4	170278462	+	chr4	170278884	+	Y	N	Y	Y	N	N	del	na	alt-EJ	intergenic	SH3RF1,NEK1	-,-	protein_cod	86206;35964	intergenic	SH3RF1,NEK1	-,-	protein_cod	86628;35542
536	chr4	172374411	+	chr4	172379426	+	Y	Y	Y	Y	Y	Y	del	na	NHEJ	intergenic	RP11-717H13.1,RP11-	-,-	lincRNA,linc	154993;99740	intergenic	RP11-717H13.1,RP11-97	-,-	lincRNA,linc	160008;94725
537	chr4	175625167	+	chr4	175626972	+	Y	Y	Y	Y	Y	Y	del	na	alt-EJ	intronic	GLRA3	-	protein_cod	0	intronic	GLRA3	-	protein_cod	0
538	chr4	176689450	+	chr4	176689972	+	N	N	N	Y	N	Y	del	na	alt-EJ	intronic	GPM6A	-	protein_cod	0	intronic	GPM6A	-	protein_cod	0
539	chr4	180063818	+	chr4	180064162	+	Y	Y	Y	N	N	N	del	na	alt-EJ	intergenic	RP11-296L20.1,RP11-	+,-	pseudogene	73070;246470	intergenic	RP11-296L20.1,RP11-40	+,-	pseudogene	73414;246126
540	chr4	180097876	+	chr4	180098508	+	Y	Y	Y	Y	N	Y	del	na	NHEJ	intergenic	RP11-296L20.1,RP11-	+,-	pseudogene	107128;212412	intergenic	RP11-296L20.1,RP11-40	+,-	pseudogene	107760;211780
541	chr4	182056560	+	chr4	182057131	+	Y	Y	Y	Y	Y	Y	del	na	alt-EJ	ncRNA_intr	LINC00290	-	lincRNA	0	ncRNA_intr	LINC00290	-	lincRNA	0
542	chr4	186441636	+	chr4	186444075	+	Y	Y	Y	Y	Y	Y	del	na	alt-EJ	intronic	PDLIM3	-	protein_cod	0	intronic	PDLIM3	-	protein_cod	0
543	chr4	189137172	+	chr4	189137433	+	N	Y	N	N	N	N	del	na	VNTR	intergenic	RP11-366H4.1,RP11-2	+,-	lincRNA,pse	54879;133358	intergenic	RP11-366H4.1,RP11-219	+,-	lincRNA,pse	55140;133097
544	chr4	190191601	+	chr4	190200464	+	Y	Y	Y	Y	Y	N	del	na	alt-EJ	intergenic	RP11-706F1.1,RP11-7	+,-	lincRNA,linc	65570;18230	intergenic	RP11-706F1.1,RP11-706	+,-	lincRNA,linc	74433;9367
545	chr4	190572699	+	chr4	190573534	+	N	N	Y	N	N	N	del	na	alt-EJ	intergenic	HSP90AA4P,RP11-462	+,+	pseudogene	176353;8060	intergenic	HSP90AA4P,RP11-462G	+,+	pseudogene	177188;7225
546	chr4	190573132	+	chr4	190573426	+	N	N	N	N	N	Y	del	na	alt-EJ	intergenic	HSP90AA4P,RP11-462	+,+	pseudogene	176786;7627	intergenic	HSP90AA4P,RP11-462G	+,+	pseudogene	177080;7333
547	chr4	190606114	+	chr4	190610285	+	N	N	N	N	N	Y	del	na	alt-EJ	intergenic	RP11-462G22.1,RP11-	+,-	lincRNA,linc	23474;18921	intergenic	RP11-462G22.1,RP11-46	+,-	lincRNA,linc	27645;14750
548	chr4	190606817	+	chr4	190610094	+	N	N	N	N	Y	N	del	na	alt-EJ	intergenic	RP11-462G22.1,RP11-	+,-	lincRNA,linc	24177;18218	intergenic	RP11-462G22.1,RP11-46	+,-	lincRNA,linc	27454;14941
549	chr4	190657074	+	chr4	190657554	+	N	N	N	N	Y	N	del	na	alt-EJ	intergenic	RNU1-51P,AF146191.	-,-	snRNA,lincR	26206;44416	intergenic	RNU1-51P,AF146191.4	-,-	snRNA,lincR	26686;43936
550	chr4	190662931	+	chr4	190663727	+	Y	Y	Y	Y	Y	Y	del	na	NHEJ	intergenic	RNU1-51P,AF146191.	-,-	snRNA,lincR	32063;38559	intergenic	RNU1-51P,AF146191.4	-,-	snRNA,lincR	32859;37763
551	chr5	282677	+	chr5	282820	+	N	N	Y	N	N	N	del	na	alt-EJ	intronic	PDCD6	+	protein_cod	0	intronic	PDCD6	+	protein_cod	0
552	chr5	650483	+	chr5	650865	+	N	N	N	N	Y	N	del	na	alt-EJ	intronic	CEP72	+	protein_cod	0	intronic	CEP72	+	protein_cod	0
553	chr5	712047	+	chr5	796171	+	N	N	N	Y	N	N	del	na	alt-EJ	UTR3	ZDHHC11B	-	protein_cod	0	UTR3	ZDHHC11	-	protein_cod	0
554	chr5	1178293	+	chr5	1180733	+	Y	Y	Y	Y	Y	Y	del	na	NHEJ	ncRNA_intr	CTD-3080P12.3	-	antisense	0	intergenic	CTD-3080P12.3,SLC6A1	-,+	antisense,pr	2013;20977
555	chr5	1924590	+	chr5	1925203	+	Y	Y	Y	Y	Y	Y	del	na	NHEJ	intergenic	CTD-2194D22.4,RP11	+,+	antisense,lir	23983;9387	intergenic	CTD-2194D22.4,RP11-2	+,+	antisense,lir	24596;8774
556	chr5	10756181	+	chr5	10756517	+	N	N	Y	Y	Y	Y	del	na	alt-EJ	intronic	DAP	-	protein_cod	0	intronic	DAP	-	protein_cod	0
557	chr5	11817047	+	chr5	11817600	+	Y	N	N	N	N	N	del	na	alt-EJ	intronic	CTNND2	-	protein_cod	0	intronic	CTNND2	-	protein_cod	0
558	chr5	16869734	+	chr5	16870763	+	N	Y	Y	N	Y	Y	del	na	alt-EJ	intronic	MYO10	-	protein_cod	0	intronic	MYO10	-	protein_cod	0
559	chr5	18873312	+	chr5	18874848	+	Y	Y	Y	Y	Y	Y	del	na	NHEJ	intergenic	snoU13,UBE2V1P12	+,-	snoRNA,pse	29203;13419	intergenic	snoU13,UBE2V1P12	+,-	snoRNA,pse	30739;11883
560	chr5	19078554	+	chr5	19079038	+	N	N	N	N	Y	Y	del	na	alt-EJ	ncRNA_intr	RP11-124N3.3	-	lincRNA	0	ncRNA_intr	RP11-124N3.3	-	lincRNA	0
561	chr5	25885860	+	chr5	25886533	+	N	Y	N	Y	Y	Y	del	na	alt-EJ	intergenic	RNU6-374P,MSNP1	-,+	snRNA,pseu	184427;23752	intergenic	RNU6-374P,MSNP1	-,+	snRNA,pseu	185100;23079
562	chr5	26119158	+	chr5	26119396	+	N	N	N	N	Y	N	del	na	alt-EJ	intergenic	RNU4-43P,RP11-351N	+,-	snRNA,lincR	106024;263470	intergenic	RNU4-43P,RP11-351N6.	+,-	snRNA,lincR	106262;263232
563	chr5	29434219	+	chr5	69664776	+	N	N	Y	N	N	N	del	na	alt-EJ	intergenic	CTD-2010I22.2,UBL5F	-,+	lincRNA,pse	38131;166564	intergenic	RP11-1415C14.3,GTF2H	-,+	pseudogene	84090;46403
564	chr5	41226816	+	chr5	41243965	+	Y	Y	Y	Y	Y	Y	del	na	alt-EJ	intronic	C6	-	protein_cod	0	intronic	C6	-	protein_cod	0
565	chr5	44794994	+	chr5	44795572	+	Y	Y	Y	Y	Y	Y	del	na	alt-EJ	ncRNA_intr	RP11-53O19.1	-	antisense	0	ncRNA_intr	RP11-53O19.1	-	antisense	0
566	chr5	45527131	+	chr5	45527287	+	Y	N	N	N	N	N	del	na	alt-EJ	intronic	HCN1	-	protein_cod	0	intronic	HCN1	-	protein_cod	0
567	chr5	57323477	+	chr5	57333780	+	Y	Y	Y	Y	Y	Y	del	na	alt-EJ	intergenic	RP11-478P10.1,snoU1	-,+	lincRNA,snc	128488;42538	intergenic	RP11-478P10.1,snoU13	-,+	lincRNA,snc	138791;32235
568	chr5	60001706	+	chr5	60003668	+	Y	N	Y	Y	Y	Y	del	na	alt-EJ	intergenic	DEPDC1B,KRT8P31	-,-	protein_cod	5689;37684	intergenic	DEPDC1B,KRT8P31	-,-	protein_cod	7651;35722
569	chr5	65028091	+	chr5	65030389	+	Y	Y	Y	Y	Y	Y	del	na	alt-EJ	intronic	NLN	+	protein_cod	0	intronic	NLN	+	protein_cod	0
570	chr5	68724040	+	chr5	68724493	+	Y	N	Y	Y	Y	Y	del	na	alt-EJ	intronic	MARVELD2	+	protein_cod	0	intronic	MARVELD2	+	protein_cod	0
571	chr5	90499773	+	chr5	90502097	+	Y	Y	Y	Y	Y	Y	del	na	alt-EJ	intergenic	GPR98,RP11-414H23.	+,+	protein_cod	39735;19463	intergenic	GPR98,RP11-414H23.2	+,+	protein_cod	42059;17139
572	chr5	95966536	+	chr5	95967363	+	Y	Y	Y	Y	Y	Y	del	na	alt-EJ	ncRNA_intr	CTD-2337A12.1	+	antisense	0	intronic	CAST	+	protein_cod	0
573	chr5	99400880	+	chr5	99715014	+	Y	N	Y	N	N	N	del	na	alt-EJ	intergenic	RP11-475J5.5,RNU6-1	-,+	pseudogene	10131;88496	intergenic;ir	RNU6-1119P,RP11-346J	+,+	snRNA,lincR	225531;22131
574	chr5	99401013	+	chr5	99715014	+	N	N	N	N	Y	N	del	na	alt-EJ	intergenic	RP11-475J5.5,RNU6-1	-,+	pseudogene	10264;88363	intergenic;ir	RNU6-1119P,RP11-346J	+,+	snRNA,lincR	225531;22131
575	chr5	106019446	+	chr5	106239810	+	N	N	N	N	Y	N	del	na	alt-EJ	intergenic	SNORA31,CTC-254B4	+,-	snoRNA,linc	136942;131452	ncRNA_intr	CTC-254B4.1	-	lincRNA	0
576	chr5	114756506	+	chr5	114757898	+	Y	Y	Y	Y	Y	Y	del	na	alt-EJ	intergenic	AK3P4,CTB-161J7.1	+,+	pseudogene	17847;68825	intergenic	AK3P4,CTB-161J7.1	+,+	pseudogene	19239;67433
577	chr5	115130462	+	chr5	115130812	+	Y	Y	Y	Y	Y	Y	del	na	alt-EJ	intergenic	RNU2-49P,CDO1	-,-	snRNA,prot	20263;9968	intergenic	RNU2-49P,CDO1	-,-	snRNA,prot	20613;9618
578	chr5	120099747	+	chr5	120174317	+	Y	N	N	N	N	N	del	na	NHEJ	intergenic	RNU4-69P,CTD-2334D	-,+	snRNA,lincR	53212;17166	intergenic	CTD-2334D19.1,AC0085	+,+	lincRNA,pse	47844;201468
579	chr5	120103495	+	chr5	120126635	+	N	N	N	Y	N	Y	del	na	alt-EJ	intergenic	RNU4-69P,CTD-2334D	-,+	snRNA,lincR	56960;13418	downstream	CTD-2334D19.1	+	lincRNA	0
580	chr5	121059231	+	chr5	121060011	+	Y	N	N	Y	N	N	del	na	NHEJ	intergenic	RP11-510I6.3,FTMT	-,+	pseudogene	51664;128419	intergenic	RP11-510I6.3,FTMT	-,+	pseudogene	52444;127639
581	chr5	126458377	+	chr5	126458816	+	Y	Y	Y	Y	Y	Y	del	na	NHEJ	intergenic	C5orf63,MRPS5P3	-,-	protein_cod	49193;20397	intergenic	C5orf63,MRPS5P3	-,-	protein_cod	49632;19958
582	chr5	127172331	+	chr5	127173282	+	N	N	N	Y	N	N	del	na	alt-EJ	ncRNA_intr	CTC-228N24.1	+	lincRNA	0	ncRNA_intr	CTC-228N24.1	+	lincRNA	0

583	chr5	127336000	+	chr5	127336932	+	Y	Y	Y	N	Y	Y	del	na	alt-EJ	ncRNA_intr	CTC-228N24.3	-	lincRNA	0	ncRNA_intr	CTC-228N24.3	-	lincRNA	0
584	chr5	133134678	+	chr5	133136489	+	N	Y	Y	N	N	N	del	na	alt-EJ	intergenic;ir	AC005178.1,snoU13	-,-	pseudogene	78053;51870	intergenic;ir	AC005178.1,snoU13	-,-	pseudogene	79864;50059
585	chr5	136854681	+	chr5	136856398	+	Y	Y	Y	Y	Y	Y	del	na	alt-EJ	intergenic	SPOCK1,AC106775.1	-,+	protein_cod	19644;14462	intergenic	SPOCK1,AC106775.1	-,+	protein_cod	21361;12745
586	chr5	138734512	+	chr5	138734762	+	Y	N	N	N	N	N	del	na	alt-EJ	intronic	SPATA24	-	protein_cod	0	intronic	SPATA24	-	protein_cod	0
587	chr5	141459200	+	chr5	141459506	+	Y	Y	N	N	N	Y	del	na	NHEJ	intergenic	AC005740.4,NDFIP1	-,+	pseudogene	42510;28870	intergenic	AC005740.4,NDFIP1	-,+	pseudogene	42816;28564
588	chr5	150177661	+	chr5	150181600	+	Y	Y	Y	Y	Y	Y	del	na	NHEJ	intergenic	AC010441.1,IRGM	+,+	protein_cod	1364;48424	intergenic	AC010441.1,IRGM	+,+	protein_cod	5303;44485
589	chr5	152684103	+	chr5	152692358	+	N	N	N	Y	N	N	del	na	alt-EJ	intergenic	AC091969.1,GRIA1	-,+	lincRNA,pro	81000;186003	intergenic	AC091969.1,GRIA1	-,+	lincRNA,pro	89255;177748
590	chr5	155311786	+	chr5	155312578	+	Y	Y	Y	Y	Y	Y	del	na	alt-EJ	intronic	SGCD	+	protein_cod	0	intronic	SGCD	+	protein_cod	0
591	chr5	162462399	+	chr5	162462755	+	N	N	N	N	Y	N	del	na	NHEJ	ncRNA_intr	RP11-541P9.3	-	antisense	0	ncRNA_intr	RP11-541P9.3	-	antisense	0
592	chr6	857169	+	chr6	857436	+	Y	N	N	N	N	Y	del	na	NHEJ	intergenic	RP11-532F6.5,RP5-10	+,-	lincRNA,linc	76521;48293	intergenic	RP11-532F6.5,RP5-1077	+,-	lincRNA,linc	76788;48026
593	chr6	1270713	+	chr6	1270999	+	N	N	Y	N	N	N	del	na	NHEJ	intergenic	snoU13,FOXQ1	+,+	snoRNA,pro	83858;41962	intergenic	snoU13,FOXQ1	+,+	snoRNA,pro	84144;41676
594	chr6	8666373	+	chr6	8672863	+	N	N	N	N	N	Y	del	na	NHEJ	intergenic	HULC,RP11-314C16.1	+,+	lincRNA,linc	12294;118038	intergenic	HULC,RP11-314C16.1	+,+	lincRNA,linc	18784;111548
595	chr6	18402088	+	chr6	18402869	+	Y	Y	Y	Y	Y	Y	del	na	alt-EJ	intronic	RNF144B	+	protein_cod	0	intronic	RNF144B	+	protein_cod	0
596	chr6	23743259	+	chr6	23745946	+	Y	Y	Y	Y	Y	Y	del	na	NHEJ	intergenic	RP4-810F7.1,SPTLC1	+,-	pseudogene	93257;113667	intergenic	RP4-810F7.1,SPTLC1P2	+,-	pseudogene	95944;110980
597	chr6	24325363	+	chr6	24327810	+	Y	Y	Y	Y	Y	Y	del	na	alt-EJ	intronic	DCDC2	-	protein_cod	0	intronic	DCDC2	-	protein_cod	0
598	chr6	34699920	+	chr6	34701120	+	Y	N	Y	N	N	Y	del	na	alt-EJ	intergenic	RP11-140K17.2,RP3-3	+,+	pseudogene	16203;12033	intergenic	RP11-140K17.2,RP3-375	+,+	pseudogene	17403;10833
599	chr6	34700241	+	chr6	34701125	+	N	Y	N	Y	Y	N	del	na	alt-EJ	intergenic	RP11-140K17.2,RP3-3	+,+	pseudogene	16524;11712	intergenic	RP11-140K17.2,RP3-375	+,+	pseudogene	17408;10828
600	chr6	48930945	+	chr6	48938534	+	Y	Y	Y	Y	Y	Y	del	na	NHEJ	intergenic	RP1-57E3.1,RP4-724F	-,-	pseudogene	10527;114404	intergenic	RP1-57E3.1,RP4-724P1	-,-	pseudogene	18116;106815
601	chr6	51736110	+	chr6	51736806	+	Y	Y	Y	Y	Y	Y	del	na	alt-EJ	intronic	PKHD1	-	protein_cod	0	intronic	PKHD1	-	protein_cod	0
602	chr6	52651128	+	chr6	52652647	+	Y	Y	N	N	N	Y	del	na	l_LTR_LTR1	intergenic	RP1-152L7.1,GSTA1	-,-	pseudogene	7983;5334	intergenic	RP1-152L7.1,GSTA1	-,-	pseudogene	9502;3815
603	chr6	57297190	+	chr6	57301247	+	Y	Y	Y	Y	Y	Y	del	na	NHEJ	intronic	PRIM2	+	protein_cod	0	intronic	PRIM2	+	protein_cod	0
604	chr6	57533116	+	chr6	57534168	+	N	N	Y	N	N	N	del	na	VNTR	intergenic	PRIM2,GAPDHP41	+,-	protein_cod	19741;153853	intergenic	PRIM2,GAPDHP41	+,-	protein_cod	20793;152801
605	chr6	66111311	+	chr6	66111704	+	N	Y	N	N	Y	N	del	na	alt-EJ	intronic	EYS	-	protein_cod	0	intronic	EYS	-	protein_cod	0
606	chr6	68101532	+	chr6	68112168	+	Y	Y	Y	Y	Y	Y	del	na	alt-EJ	intergenic	SNORD65,RP11-409K	-,-	snoRNA,ps	181159;64707	intergenic	SNORD65,RP11-409K15	-,-	snoRNA,ps	191795;54071
607	chr6	74592061	+	chr6	74602441	+	Y	Y	Y	Y	Y	Y	del	na	alt-EJ	intergenic	CD109,TXNP7	+,-	protein_cod	54021;122175	intergenic	CD109,TXNP7	+,-	protein_cod	64401;111795
608	chr6	77097494	+	chr6	77102644	+	Y	Y	Y	Y	Y	Y	del	na	alt-EJ	intergenic	RNU6-248P,RNU6-261	+,-	snRNA,snR	294837;59211	intergenic	RNU6-248P,RNU6-261P	+,-	snRNA,snR	299987;54061
609	chr6	81283718	+	chr6	81293578	+	Y	Y	Y	Y	Y	Y	del	na	NHEJ	intergenic	RP11-486E2.1,FAM46	-,-	pseudogene	16812;917438	intergenic	RP11-486E2.1,FAM46A	-,-	pseudogene	26672;907578
610	chr6	92807965	+	chr6	92808366	+	Y	N	N	N	N	N	del	na	alt-EJ	intergenic	RPL5P19,U3	+,+	pseudogene	83513;21275	intergenic	RPL5P19,U3	+,+	pseudogene	83914;20874
611	chr6	93706456	+	chr6	93743265	+	N	N	N	N	N	Y	del	na	alt-EJ	intergenic	ATF1P1,RP1-23E21.2	-,+	pseudogene	108157;73649	intergenic	ATF1P1,RP1-23E21.2	-,+	pseudogene	144966;36840
612	chr6	93706892	+	chr6	93743870	+	N	N	N	Y	N	N	del	na	alt-EJ	intergenic	ATF1P1,RP1-23E21.2	-,+	pseudogene	108593;73213	intergenic	ATF1P1,RP1-23E21.2	-,+	pseudogene	145571;36235
613	chr6	98595583	+	chr6	98598034	+	Y	N	Y	Y	Y	Y	del	na	NHEJ	ncRNA_intr	RP11-436D23.1	+	lincRNA	0	ncRNA_intr	RP11-436D23.1	+	lincRNA	0
614	chr6	111071016	+	chr6	111071570	+	Y	N	Y	Y	Y	Y	del	na	NHEJ	intronic	CDK19	-	protein_cod	0	intronic	CDK19	-	protein_cod	0
615	chr6	121797444	+	chr6	121798056	+	Y	Y	Y	Y	Y	Y	del	na	alt-EJ	intergenic	RNU4-35P,RP1-172I2	-,-	snRNA,pseu	22177;1975	intergenic	RNU4-35P,RP1-172I22.1	-,-	snRNA,pseu	22789;1363
616	chr6	136582615	+	chr6	136589300	+	N	N	Y	N	N	N	del	na	NHEJ	exonic;exon	BCLAF1	-	protein_cod	0	exonic	BCLAF1	-	protein_cod	0
617	chr6	136594326	+	chr6	136596671	+	N	N	N	N	Y	N	del	na	alt-EJ	splicing	BCLAF1	-	protein_cod	0	exonic	BCLAF1	-	protein_cod	0
618	chr6	136851675	+	chr6	136852924	+	Y	Y	Y	Y	Y	Y	del	na	NHEJ	intronic	MAP7	-	protein_cod	0	intronic	MAP7	-	protein_cod	0
619	chr6	137313693	+	chr6	137314187	+	Y	Y	Y	Y	Y	Y	del	na	NHEJ	ncRNA_intr	RP11-55K22.5	-	lincRNA	0	ncRNA_intr	RP11-55K22.5	-	lincRNA	0
620	chr6	148683250	+	chr6	148684464	+	N	Y	N	Y	Y	Y	del	na	alt-EJ	intronic	SASH1	+	protein_cod	0	intronic	SASH1	+	protein_cod	0
621	chr6	152389991	+	chr6	152392236	+	Y	Y	Y	Y	Y	Y	del	na	alt-EJ	intronic	ESR1	+	protein_cod	0	intronic	ESR1	+	protein_cod	0
622	chr6	153747364	+	chr6	153748554	+	Y	Y	N	Y	N	N	del	na	alt-EJ	intergenic;ir	RNA5SP225,RP11-33	+,+	rRNA,pseu	5657;239478	intergenic;ir	RNA5SP225,RP11-331O	+,+	rRNA,pseu	6847;238288
623	chr6	154619997	+	chr6	154621513	+	Y	Y	Y	Y	Y	Y	del	na	alt-EJ	intronic	IPCEF1	-	protein_cod	0	intronic	IPCEF1	-	protein_cod	0
624	chr6	159126111	+	chr6	159126432	+	N	N	N	N	N	Y	del	na	alt-EJ	intronic	SYTL3	+	protein_cod	0	intronic	SYTL3	+	protein_cod	0
625	chr6	159313925	+	chr6	159318652	+	Y	Y	Y	Y	Y	Y	del	na	alt-EJ	intronic	C6orf99	+	protein_cod	0	intronic	C6orf99	+	protein_cod	0
626	chr6	160877814	+	chr6	160956374	+	Y	N	N	N	N	Y	del	na	alt-EJ	ncRNA_intr	LPAL2	-	pseudogene	0	intronic	LPA	-	protein_cod	0
627	chr6	160877897	+	chr6	160956009	+	N	Y	N	N	N	N	del	na	alt-EJ	ncRNA_intr	LPAL2	-	pseudogene	0	intronic	LPA	-	protein_cod	0
628	chr6	161263311	+	chr6	161278498	+	N	N	N	N	N	Y	del	na	alt-EJ	intergenic	PLG,RP11-235G24.1	+,-	protein_cod	88973;30609	intergenic	PLG,RP11-235G24.1	+,-	protein_cod	104160;15422
629	chr6	165724667	+	chr6	165732134	+	Y	N	N	N	Y	Y	del	na	alt-EJ	intergenic	C6orf118,PDE10A	-,-	protein_cod	1571;16109	intergenic	C6orf118,PDE10A	-,-	protein_cod	9038;8642
630	chr6	167488070	+	chr6	167489577	+	Y	N	N	N	N	N	del	na	NHEJ	ncRNA_intr	RP11-517H2.6	+	processed_	0	ncRNA_intr	RP11-517H2.6	+	processed_	0
631	chr6	170206122	+	chr6	170206406	+	N	Y	N	N	N	N	del	na	alt-EJ	intergenic	LINC00574,RP1-182D	+,-	lincRNA,linc	3153;3292	intergenic	LINC00574,RP1-182D15	+,-	lincRNA,linc	3437;3008
632	chr6	170228977	+	chr6	170229426	+	N	Y	N	Y	Y	Y	del	na	alt-EJ	intergenic	RP1-182D15.2,RP11-3	-,+	lincRNA,ant	18779;248764	intergenic	RP1-182D15.2,RP11-302	-,+	lincRNA,ant	19228;248315
633	chr7	224494	+	chr7	225995	+	Y	N	N	N	N	N	del	na	alt-EJ	intronic	FAM20C	+	protein_cod	0	intronic	FAM20C	+	protein_cod	0
634	chr7	1944499	+	chr7	1944983	+	N	Y	N	N	N	N	del	na	alt-EJ	intronic	MAD1L1	-	protein_cod	0	intronic	MAD1L1	-	protein_cod	0
635	chr7	5842872	+	chr7	5843118	+	N	N	N	N	N	Y	del	na	alt-EJ	intergenic	RNF216,ZNF815P	-,+	protein_cod	21502;19919	intergenic	RNF216,ZNF815P	-,+	protein_cod	21748;19673
636	chr7	9730726	+	chr7	9743956	+	N	N	N	Y	Y	Y	del	na	NHEJ	intergenic	AC096553.5,AC06083	+,+	pseudogene	55393;35144	intergenic	AC096553.5,AC060834.2	+,+	pseudogene	68623;21914
637	chr7	16171773	+	chr7	16174073	+	Y	Y	Y	Y	Y	Y	del	na	NHEJ	intronic	ISPD	-	protein_cod	0	intronic	ISPD	-	protein_cod	0
638	chr7	22434801	+	chr7	22436762	+	Y	Y	Y	Y	Y	Y	del	na	alt-EJ	intergenic	AC099759.1,STEAP1E	+,-	miRNA,prot	15081;24262	intergenic	AC099759.1,STEAP1B	+,-	miRNA,prot	17042;22301
639	chr7	24038163	+	chr7	24040073	+	Y	Y	Y	Y	Y	Y	del	na	alt-EJ	intergenic	AC009508.1,RNA5SP2	-,+	lincRNA,rRN	101209;134161	intergenic	AC009508.1,RNA5SP22	-,+	lincRNA,rRN	103119;132251
640	chr7	46355983	+	chr7	46362390	+	Y	Y	Y	Y	Y	Y	del	na	alt-EJ	ncRNA_intr	AC092657.2	+	lincRNA	0	ncRNA_intr	AC092657.2	+	lincRNA	0
641	chr7	52171075	+	chr7	52397319	+	N	Y	N	N	N	N	del	na	alt-EJ	intergenic	RN7SL292P,RP11-153	+,-	misc_RNA,l	386460;61856	intergenic	snoU13,RP11-398K14.1	-,-	snoRNA,ps	5734;562211
642	chr7	61532034	+	chr7	61536426	+	N	N	N	Y	N	N	del	na	alt-EJ	intergenic	NONE,RP11-715L17.1	NONE,+	NONE,pseu	NONE;289835	intergenic	NONE,RP11-715L17.1	NONE,+	NONE,pseu	NONE;285443
643	chr7	61749551	+	chr7	61804093	+	N	Y	N	Y	N	N	del	na	NHEJ	intergenic	NONE,RP11-715L17.1	NONE,+	NONE,pseu	NONE;72318	intergenic	NONE,RP11-715L17.1	NONE,+	NONE,pseu	NONE;17776
644	chr7	61789192	+	chr7	61789337	+	N	N	N	N	N	Y	del	na	VNTR	intergenic	NONE,RP11-715L17.1	NONE,+	NONE,pseu	NONE;32677	intergenic	NONE,RP11-715L17.1	NONE,+	NONE,pseu	NONE;32532
645	chr7	63296652	+	chr7	67427956	+	N	N	N	N	Y	N	del	na	alt-EJ	intergenic	CTD-2526L21.3,RP11-	+,+	pseudogene	46176;7040	intergenic	RP11-421N10.1,RP11-35	-,+	pseudogene	192501;57284
646	chr7	63655297	+	chr7	63655806	+	N	N	N	N	Y	N	del	na	alt-EJ	intergenic	RP11-165H4.3,RP11-4	+,+	pseudogene	12026;12284	intergenic	RP11-165H4.3,RP11-468	+,+	pseudogene	12535;11775
647	chr7	71211905	+	chr7	71212963	+	Y	Y	Y	Y	Y	Y	del	na	alt-EJ	intergenic	WBSCR17,CALN1	+,-	protein_cod	33321;32571	intergenic	WBSCR17,CALN1	+,-	protein_cod	34379;31513
648	chr7	76870542	+	chr7	76870797	+	N	N	Y	N	Y	N	del	na	NHEJ	intronic	CCDC146	+	protein_cod	0	intronic	CCDC146	+	protein_cod	0
649	chr7	81358337	+	chr7	81358612	+	Y	N	N	Y	N	Y	del	na	NHEJ	intronic	HGF	-	protein_cod	0	intronic	HGF	-	protein_cod	0
650	chr7	97655762	+	chr7	97657560	+	N	N	N	Y	Y	N	del	na	alt-EJ	intergenic	RN7SL13P,LMTK2	+,+	misc_RNA,p	2474;80435	intergenic	RN7SL13P,LMTK2	+,+	misc_RNA,p	4272;78637
651	chr7	100550638	+	chr7	100551251	+	Y	N	N	N	N	N	del	na	alt-EJ	upstream;d	MUC3A,MUC3A	+,+	protein_cod	0	exonic	MUC3A	+	protein_cod	0
652	chr7	100556007	+	chr7	100607228	+	N	N	Y	N	Y	N	del	na	alt-EJ	intronic	MUC3A	+	protein_cod	0	ncRNA_exo	RP11-395B7.2	-	processed_	0
653	chr7	105733991	+	chr7	105734330	+	N	Y	Y	N	N	Y	del	na	alt-EJ	intronic	SYPL1	-	protein_cod	0	intronic	SYPL1	-	protein_cod	0
654	chr7	115583565	+	chr7	115587801	+	Y	Y	Y	Y	Y	Y	del	na	alt-EJ	intronic	TFEC	-	protein_cod	0	intronic	TFEC	-	protein_cod	0
655	chr7	119428936	+	chr7	119429429	+	Y	Y	Y	Y	Y	Y	del	na	alt-EJ	ncRNA_intr	RP11-328J2.1	-	lincRNA	0	ncRNA_intr	RP11-328J2.1	-	lincRNA	0
656	chr7	126045887	+	chr7	126051449	+	Y	Y	Y	Y	Y	Y	del	na	alt-EJ	ncRNA_intr	AC000370.2	+	antisense	0	ncRNA_intr	AC000370.2	+	antisense	0
657	chr7	132669983	+	chr7	132670208	+	N	N	N	Y	N	N	del	na	alt-EJ	intronic	CHCHD3	-	protein_cod	0	intronic	CHCHD3	-	protein_cod	0
658	chr7	132670315	+	chr7	132670493	+	N	N	N	N	N	Y	del	na	alt-EJ	intronic	CHCHD3	-	protein_cod	0	intronic	CHCHD3	-	protein_cod	0
659	chr7	133785001	+	chr7	133798331	+	Y	Y	Y	Y	Y	Y	del	na	alt-EJ	intergenic	EXOC4,LRGUK	+,+	protein_cod	34489;27051	intergenic	EXOC4,LRGUK	+,+	protein_cod	47819;13721
660	chr7	140003972	+	chr7	140009832	+	N	N	Y	Y	N	Y	del	na	alt-EJ	intronic	SLC37A3	-	protein_cod	0	intronic	SLC37A3	-	protein_cod	0
661	chr7	142036585	+	chr7	142041966	+	Y	Y	Y	Y	Y	Y	del	na	alt-EJ	intergenic	TRBV7-1,TRBV4-2	+,+	TR_V_gene	4058;8667	intergenic	TRBV7-1,TRBV4-2	+,+	TR_V_gene	9439;3286
662	chr7	142099580	+	chr7	142247245	+	Y	Y	N	N	N	Y	del	na	alt-EJ	exonic;exon	TRBV7-8	-	TR_V_gene	0	exonic;exon	TRBV7-3	-	TR_V_gene	0
663	chr7	142105725	+	chr7	142125928	+	N	N	Y	N	N	N	del	na	alt-EJ	intergenic	TRBV6-9,TRBV5-7	-,-	TR_V_gene	1172;5668	intergenic	TRBV6-8,TRBV5-6	-,-	TR_V_gene	1363;5484
664	chr7	142467772	+	chr7	142478350	+	N	N	Y	N	N	N	del	na	alt-EJ	upstream	PRSS3P1	+	pseudogene	0	ncRNA_intr	PRSS3P2	+	pseudogene	0
665	chr7	148063141	+	chr7	148063290	+	N	N	N	N	N	Y	del	na	alt-EJ	intronic	CNTNAP2	+	protein_cod	0	intronic	CNTNAP2	+	protein_cod	0
666	chr7	148072864	+	chr7	148076325	+	Y	Y	Y	Y	Y	Y	del	na	alt-EJ	intronic	CNTNAP2	+	protein_cod	0	intronic	CNTNAP2	+	protein_cod	0

667	chr7	152111273	+	chr7	152113612	+	N	Y	Y	N	N	Y	del	na	alt-EJ	intronic	KMT2C	-	protein_cod	0	intronic	KMT2C	-	protein_cod	0
668	chr7	155726669	+	chr7	155726917	+	Y	N	N	N	N	N	del	na	alt-EJ	intergenic	AC104133.1,AC02121	+,+	miRNA,prot	61968;28657	intergenic	AC104133.1,AC021218.2	+,+	miRNA,prot	62216;28409
669	chr7	155726947	+	chr7	155727203	+	N	N	N	N	N	Y	del	na	alt-EJ	intergenic	AC104133.1,AC02121	+,+	miRNA,prot	62246;28379	intergenic	AC104133.1,AC021218.2	+,+	miRNA,prot	62502;28123
670	chr7	155726969	+	chr7	155727455	+	N	Y	N	N	N	N	del	na	alt-EJ	intergenic	AC104133.1,AC02121	+,+	miRNA,prot	62268;28357	intergenic	AC104133.1,AC021218.2	+,+	miRNA,prot	62754;27871
671	chr7	157733334	+	chr7	157733532	+	N	N	N	N	N	Y	del	na	alt-EJ	intronic	PTPRN2	-	protein_cod	0	intronic	PTPRN2	-	protein_cod	0
672	chr8	1837320	+	chr8	1838045	+	N	N	N	Y	N	N	del	na	alt-EJ	intronic	ARHGEF10	+	protein_cod	0	intronic	ARHGEF10	+	protein_cod	0
673	chr8	2122083	+	chr8	2122348	+	N	N	Y	N	Y	N	del	na	alt-EJ	intergenic	MYOM2,AC133633.2	+,-	protein_cod	28703;228909	intergenic	MYOM2,AC133633.2	+,-	protein_cod	28968;228644
674	chr8	2215337	+	chr8	2216121	+	N	N	Y	Y	N	Y	del	na	alt-EJ	intergenic	NONE,AC133633.2	NONE,-	NONE,lincR	NONE;135655	intergenic	NONE,AC133633.2	NONE,-	NONE,lincR	NONE;134871
675	chr8	3994880	+	chr8	3995338	+	N	Y	Y	N	N	N	del	na	NHEJ	intronic	CSMD1	-	protein_cod	0	intronic	CSMD1	-	protein_cod	0
676	chr8	6991665	+	chr8	6995406	+	Y	Y	Y	Y	Y	Y	del	na	alt-EJ	intergenic	RPS3AP30,AF228730.	+,-	pseudogene	10439;9489	intergenic	RPS3AP30,AF228730.12	+,-	pseudogene	14180;5748
677	chr8	9497866	+	chr8	9501000	+	N	Y	Y	Y	N	N	del	na	alt-EJ	intronic	TNKS	+	protein_cod	0	intronic	TNKS	+	protein_cod	0
678	chr8	12427293	+	chr8	12485593	+	N	Y	N	N	N	N	del	na	alt-EJ	downstream	RPS3AP34	-	pseudogene	0	downstream	RPS3AP35	-	pseudogene	0
679	chr8	12427987	+	chr8	12485566	+	N	N	Y	N	N	N	del	na	alt-EJ	ncRNA_exo	RPS3AP34	-	pseudogene	0	downstream	RPS3AP35	-	pseudogene	0
680	chr8	20943403	+	chr8	20943807	+	N	N	Y	Y	N	N	del	na	NHEJ	intergenic	RP11-421P23.2,AC02	+,+	lincRNA,mi	56834;70239	intergenic	RP11-421P23.2,AC0216	+,+	lincRNA,mi	57238;69835
681	chr8	25066681	+	chr8	25070656	+	Y	Y	Y	Y	Y	Y	del	na	alt-EJ	intronic	DOCK5	+	protein_cod	0	intronic	DOCK5	+	protein_cod	0
682	chr8	32152230	+	chr8	32154994	+	Y	Y	Y	Y	Y	N	del	na	alt-EJ	intronic	NRG1	+	protein_cod	0	intronic	NRG1	+	protein_cod	0
683	chr8	32680007	+	chr8	32691264	+	Y	Y	Y	Y	Y	Y	del	na	alt-EJ	intergenic	RP11-1002K11.1,RNU	+,-	lincRNA,sn	54530;89004	intergenic	RP11-1002K11.1,RNU6-6	+,-	lincRNA,sn	65787;77747
684	chr8	51224983	+	chr8	51228316	+	Y	Y	Y	Y	Y	Y	del	na	alt-EJ	intronic	SNTG1	+	protein_cod	0	intronic	SNTG1	+	protein_cod	0
685	chr8	54122552	+	chr8	54123649	+	Y	Y	Y	Y	Y	Y	del	na	alt-EJ	ncRNA_intr	RP11-162D9.3	+	antisense	0	ncRNA_intr	RP11-162D9.3	+	antisense	0
686	chr8	58116933	+	chr8	58118309	+	Y	N	N	N	N	N	del	na	alt-EJ	ncRNA_intr	RP11-513O17.2	+	lincRNA	0	ncRNA_intr	RP11-513O17.2	+	lincRNA	0
687	chr8	80029401	+	chr8	80137177	+	N	N	N	Y	N	N	del	na	alt-EJ	intergenic	AC009941.1,RP11-111	+,+	miRNA,linc	19713;180551	intergenic	AC009941.1,RP11-1114I	+,+	miRNA,linc	127489;72775
688	chr8	108380699	+	chr8	108381100	+	N	Y	N	N	N	N	del	na	alt-EJ	intronic	ANGPT1	-	protein_cod	0	intronic	ANGPT1	-	protein_cod	0
689	chr8	110234719	+	chr8	110235053	+	Y	Y	Y	Y	Y	Y	del	na	NHEJ	intergenic	TRHR,NUDCD1	+,-	protein_cod	102906;18429	intergenic	TRHR,NUDCD1	+,-	protein_cod	103240;18095
690	chr8	112294051	+	chr8	112297140	+	Y	Y	Y	Y	Y	N	del	na	NHEJ	intergenic	RP11-946L20.4,RP11-	-,+	lincRNA,linc	45619;94817	intergenic	RP11-946L20.4,RP11-11	-,+	lincRNA,linc	48708;91728
691	chr8	138742966	+	chr8	138743863	+	Y	Y	Y	Y	Y	Y	del	na	alt-EJ	intergenic	CTD-3145H4.1,RP11-2	+,-	pseudogene	32256;78721	intergenic	CTD-3145H4.1,RP11-23	+,-	pseudogene	33153;77824
692	chr8	143337715	+	chr8	144016829	+	Y	N	N	N	N	N	del	na	alt-EJ	intronic	TSNARE1	-	protein_cod	0	intergenic	CYP11B2,RP11-273G15.	-,-	protein_cod	17570;46326
693	chr8	143337969	+	chr8	144016830	+	N	Y	Y	Y	Y	Y	del	na	alt-EJ	intronic	TSNARE1	-	protein_cod	0	intergenic	CYP11B2,RP11-273G15.	-,-	protein_cod	17571;46325
694	chr8	143397458	+	chr8	143398513	+	N	N	N	Y	N	N	del	na	NHEJ	intronic;intr	TSNARE1	-	protein_cod	0	intronic	TSNARE1	-	protein_cod	0
695	chr8	143397458	+	chr8	143398621	+	Y	N	Y	N	N	N	del	na	NHEJ	intronic;intr	TSNARE1	-	protein_cod	0	intronic	TSNARE1	-	protein_cod	0
696	chr8	143397458	+	chr8	143398657	+	N	Y	N	N	Y	N	del	na	NHEJ	intronic;intr	TSNARE1	-	protein_cod	0	intronic	TSNARE1	-	protein_cod	0
697	chr8	144634065	+	chr8	144636241	+	Y	Y	Y	Y	Y	N	del	na	alt-EJ	intergenic	RP11-661A12.5,GSDM	-,+	antisense,p	2166;1312	intronic	GSDMD	+	protein_cod	0
698	chr8	144748661	+	chr8	144749103	+	N	N	N	N	N	Y	del	na	alt-EJ	intergenic	ZNF623,ZNF707	+,+	protein_cod	12761;17961	intergenic	ZNF623,ZNF707	+,+	protein_cod	13203;17519
699	chr8	146057038	+	chr8	146057850	+	N	Y	Y	N	N	N	del	na	alt-EJ	intronic	ZNF7	+	protein_cod	0	intronic	ZNF7	+	protein_cod	0
700	chr9	3107534	+	chr9	4190314	+	N	Y	N	N	N	N	del	na	alt-EJ	intergenic	CARM1P1,RP11-32F1	-,+	pseudogene	54126;74055	intronic	GLIS3	-	protein_cod	0
701	chr9	3107563	+	chr9	4190437	+	N	N	N	N	Y	N	del	na	alt-EJ	intergenic	CARM1P1,RP11-32F1	-,+	pseudogene	54155;74026	intronic	GLIS3	-	protein_cod	0
702	chr9	6702466	+	chr9	6705127	+	N	Y	N	N	N	N	del	na	alt-EJ	intergenic	RP11-390F4.2,RP11-3	+,+	pseudogene	26852;2005	ncRNA_intr	RP11-390F4.10	+	lincRNA	0
703	chr9	10404562	+	chr9	10405093	+	Y	N	Y	Y	Y	Y	del	na	alt-EJ	intronic	PTPRD	-	protein_cod	0	intronic	PTPRD	-	protein_cod	0
704	chr9	11217954	+	chr9	11220098	+	N	N	Y	N	N	N	del	na	alt-EJ	intergenic	RP11-109M17.1,RP11-	+,-	pseudogene	204858;57360	intergenic	RP11-109M17.1,RP11-23	+,-	pseudogene	207002;55216
705	chr9	15815307	+	chr9	15821942	+	Y	Y	Y	Y	Y	Y	del	na	alt-EJ	intronic	CCDC171	+	protein_cod	0	intronic	CCDC171	+	protein_cod	0
706	chr9	23115020	+	chr9	23115322	+	N	Y	N	N	N	N	del	na	alt-EJ	intergenic	AL391117.1,SUMO2P2	-,+	miRNA,pseu	161918;517084	intergenic	AL391117.1,SUMO2P2	-,+	miRNA,pseu	162220;516782
707	chr9	24456327	+	chr9	24457598	+	N	Y	N	N	N	N	del	na	alt-EJ	intergenic	RP11-321L2.2,IZUMO	-,-	lincRNA,pro	558275;86623	intergenic	RP11-321L2.2,IZUMO3	-,-	lincRNA,pro	559546;85352
708	chr9	24456331	+	chr9	24457710	+	Y	N	Y	Y	Y	Y	del	na	alt-EJ	intergenic	RP11-321L2.2,IZUMO	-,-	lincRNA,pro	558279;86619	intergenic	RP11-321L2.2,IZUMO3	-,-	lincRNA,pro	559658;85240
709	chr9	28047359	+	chr9	28051256	+	N	Y	N	N	N	N	del	na	alt-EJ	intronic;intr	LINGO2	-	protein_cod	0	intronic;intr	LINGO2	-	protein_cod	0
710	chr9	28676975	+	chr9	28710755	+	Y	Y	Y	Y	Y	N	del	na	alt-EJ	intergenic	LINGO2,MIR876	-,-	protein_cod	6692;186649	intergenic	LINGO2,MIR876	-,-	protein_cod	40472;152869
711	chr9	29092575	+	chr9	29098016	+	Y	Y	Y	Y	Y	Y	del	na	alt-EJ	intergenic	MIR873,RP11-373A6.1	-,-	miRNA,pseu	203622;161498	intergenic	MIR873,RP11-373A6.1	-,-	miRNA,pseu	209063;156057
712	chr9	31291355	+	chr9	31292673	+	Y	Y	Y	Y	Y	Y	del	na	NHEJ	intergenic	SLC25A6P2,RP11-271	+,-	pseudogene	36580;80254	intergenic	SLC25A6P2,RP11-271O	+,-	pseudogene	37898;78936
713	chr9	33573402	+	chr9	33574215	+	Y	N	Y	N	Y	Y	del	na	alt-EJ	downstream	RP11-255A11.4,ANKR	-,+	pseudogene	0	downstream	RP11-255A11.4	-	pseudogene	0
714	chr9	36362899	+	chr9	36364098	+	N	Y	N	N	N	N	del	na	alt-EJ	intronic	RNF38	-	protein_cod	0	intronic	RNF38	-	protein_cod	0
715	chr9	69786722	+	chr9	69787295	+	N	N	Y	N	N	N	del	na	alt-EJ	intergenic	IGKV1OR-2,AL359955	-,-	IG_V_pseu	8769;35038	intergenic	IGKV1OR-2,AL359955.1	-,-	IG_V_pseu	9342;34465
716	chr9	69787281	+	chr9	69787508	+	Y	N	N	N	N	N	del	na	alt-EJ	intergenic	IGKV1OR-2,AL359955	-,-	IG_V_pseu	9328;34479	intergenic	IGKV1OR-2,AL359955.1	-,-	IG_V_pseu	9555;34252
717	chr9	71738121	+	chr9	71743356	+	Y	Y	Y	Y	Y	Y	del	na	alt-EJ	intronic	TJP2	+	protein_cod	0	intronic	TJP2	+	protein_cod	0
718	chr9	82028161	+	chr9	82033450	+	Y	Y	Y	Y	Y	Y	del	na	alt-EJ	intergenic	CHCHD2P9,RP11-375	+,-	pseudogene	21487;92558	intergenic	CHCHD2P9,RP11-375O	+,-	pseudogene	26776;87269
719	chr9	82774772	+	chr9	82776690	+	Y	Y	Y	Y	Y	Y	del	na	alt-EJ	intergenic	RP11-394O9.1,NPAP1	+,-	lincRNA,pse	125302;197172	intergenic	RP11-394O9.1,NPAP1P4	+,-	lincRNA,pse	127220;195254
720	chr9	92176046	+	chr9	92177976	+	Y	Y	Y	Y	Y	Y	del	na	NHEJ	intergenic	SEMA4D,GADD45G	-,+	protein_cod	63001;43882	intergenic	SEMA4D,GADD45G	-,+	protein_cod	64931;41952
721	chr9	96470354	+	chr9	96471465	+	Y	Y	Y	Y	Y	Y	del	na	NHEJ	intergenic	PHF2,MIR4291	+,+	protein_cod	28485;111285	intergenic	PHF2,MIR4291	+,+	protein_cod	29596;110174
722	chr9	108476303	+	chr9	108478943	+	N	N	Y	N	N	N	del	na	NHEJ	intronic	TMEM38B	+	protein_cod	0	intronic	TMEM38B	+	protein_cod	0
723	chr9	124411498	+	chr9	124411801	+	N	N	Y	Y	Y	N	del	na	alt-EJ	intronic	DAB2IP	+	protein_cod	0	intronic	DAB2IP	+	protein_cod	0
724	chr9	130901473	+	chr9	130901989	+	N	Y	N	N	N	N	del	na	alt-EJ	intergenic	AL590708.2,LCN2	+,+	protein_cod	9992;9877	intergenic	AL590708.2,LCN2	+,+	protein_cod	10508;9361
725	chr9	130901854	+	chr9	130901980	+	N	N	Y	N	N	N	del	na	alt-EJ	intergenic	AL590708.2,LCN2	+,+	protein_cod	10373;9496	intergenic	AL590708.2,LCN2	+,+	protein_cod	10499;9370
726	chr9	132191076	+	chr9	132191523	+	Y	Y	Y	Y	Y	Y	del	na	alt-EJ	intergenic	RP11-65J3.6,RP11-65	-,-	pseudogene	48327;1855	intergenic	RP11-65J3.6,RP11-65J3.	-,-	pseudogene	48774;1408
727	chr9	138479101	+	chr9	138480201	+	Y	Y	Y	Y	Y	Y	del	na	alt-EJ	upstream;d	RP11-98L5.4,RP11-98	+,+	pseudogene	0	ncRNA_intr	RP11-98L5.4	+	pseudogene	0
728	chr9	140772668	+	chr9	140773505	+	Y	Y	Y	Y	N	Y	del	na	NHEJ	exonic	CACNA1B	+	protein_cod	0	splicing	CACNA1B	+	protein_cod	0
729	chr9	140773611	+	chr9	140777196	+	N	N	N	N	Y	N	del	na	alt-EJ	exonic	CACNA1B	+	protein_cod	0	exonic	CACNA1B	+	protein_cod	0
730	chrX	3606230	+	chrX	3606648	+	N	N	N	Y	Y	Y	del	na	alt-EJ	intronic	PRKX	-	protein_cod	0	intronic	PRKX	-	protein_cod	0
731	chrX	5055345	+	chrX	5057502	+	Y	Y	Y	Y	Y	Y	del	na	alt-EJ	intergenic	AC074035.1,MTND6P	-,-	miRNA,pseu	418390;31614	intergenic	AC074035.1,MTND6P12	-,-	miRNA,pseu	420547;29457
732	chrX	7623882	+	chrX	7673883	+	Y	Y	Y	Y	Y	Y	del	na	NHEJ	intergenic	STS,VCX	+,+	protein_cod	351031;186421	intergenic	STS,VCX	+,+	protein_cod	401032;136420
733	chrX	10738634	+	chrX	10788714	+	Y	Y	Y	Y	Y	Y	del	na	alt-EJ	intronic	MID1	-	protein_cod	0	intronic	MID1	-	protein_cod	0
734	chrX	38584364	+	chrX	38588495	+	Y	Y	Y	Y	Y	N	del	na	alt-EJ	intergenic	TSPAN7,RP4-646N3.1	+,+	protein_cod	36197;45220	intergenic	TSPAN7,RP4-646N3.1	+,+	protein_cod	40328;41089
735	chrX	48092291	+	chrX	48093129	+	Y	N	N	N	N	N	del	na	alt-EJ	intergenic	RP11-552E4.4,RP11-5	-,+	pseudogene	3897;12038	intergenic	RP11-552E4.4,RP11-552	-,+	pseudogene	4735;11200
736	chrX	65977314	+	chrX	65977589	+	N	N	Y	N	N	N	del	na	alt-EJ	intergenic	RNU6-394P,RP6-22P1	-,+	snRNA,pseu	81108;616101	intergenic	RNU6-394P,RP6-22P16.	-,+	snRNA,pseu	81383;615826
737	chrX	70122992	+	chrX	70123932	+	Y	Y	Y	Y	Y	Y	del	na	alt-EJ	intronic	TEX11	-	protein_cod	0	intronic	TEX11	-	protein_cod	0
738	chrX	117454143	+	chrX	117454970	+	Y	Y	Y	Y	Y	Y	del	na	alt-EJ	intergenic	RP11-197B12.1,WDR4	+,+	pseudogene	36813;25893	intergenic	RP11-197B12.1,WDR44	+,+	pseudogene	37640;25066
739	chrX	124115712	+	chrX	124116142	+	Y	Y	Y	Y	Y	Y	del	na	alt-EJ	intergenic	TENM1,RP1-161N10.1	-,-	protein_cod	18046;221942	intergenic	TENM1,RP1-161N10.1	-,-	protein_cod	18476;221512
740	chrX	144422075	+	chrX	144424507	+	Y	Y	Y	Y	Y	Y	del	na	alt-EJ	intergenic	SPANXN1,RP13-150K	+,+	protein_cod	84443;289661	intergenic	SPANXN1,RP13-150K15	+,+	protein_cod	86875;287229
741	chrY	13448587	+	chrY	13450635	+	N	N	N	N	Y	N	del	na	alt-EJ	intergenic	AC134878.1,DUX4L16	-,+	miRNA,pseu	107954;14007	intergenic	AC134878.1,DUX4L16	-,+	miRNA,pseu	110002;11959
742	chrY	13693810	+	chrY	13705622	+	Y	N	N	N	N	N	del	na	alt-EJ	intergenic	RP11-295P22.2,RCC2	-,-	pseudogene	63897;207948	intergenic	RP11-295P22.2,RCC2P1	-,-	pseudogene	75709;196136
743	chrY	13802756	+	chrY	13803135	+	Y	N	N	N	N	N	del	na	VNTR	intergenic	RP11-295P22.2,RCC2	-,-	pseudogene	172843;99002	intergenic	RP11-295P22.2,RCC2P1	-,-	pseudogene	173222;98623
744	chrY	13839107	+	chrY	13843821	+	N	N	N	Y	N	N	del	na	alt-EJ	intergenic	RP11-295P22.2,RCC2	-,-	pseudogene	209194;62651	intergenic	RP11-295P22.2,RCC2P1	-,-	pseudogene	213908;57937
745	chrY	13851703	+	chrY	13866102	+	N	N	N	N	N	Y	del	na	alt-EJ	intergenic	RP11-295P22.2,RCC2	-,-	pseudogene	221790;50055	intergenic	RP11-295P22.2,RCC2P1	-,-	pseudogene	236189;35656
746	chrY	19316319	+	chrY	19324149	+	Y	Y	Y	Y	Y	Y	del	na	alt-EJ	intergenic	SURF6P1,TCEB1P35	+,-	pseudogene	10787;260440	intergenic	SURF6P1,TCEB1P35	+,-	pseudogene	18617;252610
747	chrY	58976017	+	chrY	58979478	+	N	Y	N	N	N	N	del	na	alt-EJ	intergenic	NONE,CTBP2P1	NONE,+	NONE,pseu	NONE;25374	intergenic	NONE,CTBP2P1	NONE,+	NONE,pseu	NONE;21913
748	chrY	58978129	+	chrY	58982423	+	N	N	N	Y	N	N	del	na	alt-EJ	intergenic	NONE,CTBP2P1	NONE,+	NONE,pseu	NONE;23262	intergenic	NONE,CTBP2P1	NONE,+	NONE,pseu	NONE;18968
749	chrY	58978358	+	chrY	58982796	+	N	Y	N	N	N	N	del	na	alt-EJ	intergenic	NONE,CTBP2P1	NONE,+	NONE,pseu	NONE;23033	intergenic	NONE,CTBP2P1	NONE,+	NONE,pseu	NONE;18595
750	chr1	913106	+	chr1	913397	+	N	N	Y	N	N	N	del_ins_uksr	na	NHEJ	intronic	C1orf170	-	protein_cod	0	intronic	C1orf170	-	protein_cod	0

751	chr1	7570071	+	chr1	7571527	+	N	Y	Y	Y	N	N	del_ins_uksr	na	FoSTeS	intronic;intrc	CAMTA1	+	protein_cod	0	intronic;intrc	CAMTA1	+	protein_cod	0
752	chr1	9595665	+	chr1	9597125	+	N	N	Y	N	N	N	del_ins_uksr	na	FoSTeS	intergenic	RP13-392I16.1,SLC25	-,+	lincRNA,pro	32135;3876	intergenic	RP13-392I16.1,SLC25A3	-,+	lincRNA,pro	33595;2416
753	chr1	17229801	+	chr1	145382197	+	N	N	N	N	Y	N	del_ins_uksr	na	FoSTeS	intergenic	RNU1-2,RP11-108M9.(	+,-	snRNA,lincF	7163;1033	ncRNA_exo	RP11-458D21.1	-	lincRNA	0
754	chr1	24520347	+	chr1	24523675	+	Y	Y	Y	Y	N	Y	del_ins_uksr	na	FoSTeS	intergenic	IFNLR1,RP11-10N16.2	-,+	protein_cod	5898;6383	intergenic	IFNLR1,RP11-10N16.2	-,+	protein_cod	9226;3055
755	chr1	38132311	+	chr1	38132982	+	N	Y	Y	Y	Y	Y	del_ins_uksr	na	FoSTeS	intergenic	RSPO1,C1orf109	-,-	protein_cod	31716;14931	intergenic	RSPO1,C1orf109	-,-	protein_cod	32387;14260
756	chr1	72449620	+	chr1	72450033	+	Y	Y	Y	Y	N	Y	del_ins_uksr	na	NHEJ	intronic	NEGR1	-	protein_cod	0	intronic	NEGR1	-	protein_cod	0
757	chr1	76111576	+	chr1	79480752	+	N	N	N	N	Y	N	del_ins_uksr	na	FoSTeS	intergenic	RP4-595K12.2,RP4-68	-,-	pseudogene	3804;64112	intergenic	ELTD1,PSAT1P3	-,-	protein_cod	8349;39878
758	chr1	86400851	+	chr1	86404539	+	Y	Y	N	Y	Y	Y	del_ins_uksr	na	NHEJ	intronic;intrc	COL24A1	-	protein_cod	0	intronic;intrc	COL24A1	-	protein_cod	0
759	chr1	106015814	+	chr1	106023398	+	Y	Y	Y	Y	Y	Y	del_ins_uksr	na	NHEJ	intergenic	RP11-414B7.1,RP11-2	+,-	pseudogene	38371;114383	intergenic	RP11-414B7.1,RP11-251	+,-	pseudogene	45955;106799
760	chr1	108733324	+	chr1	108737252	+	Y	Y	Y	Y	Y	Y	del_ins_uksr	na	FoSTeS	intronic	SLC25A24	-	protein_cod	0	intronic	SLC25A24	-	protein_cod	0
761	chr1	120393298	+	chr1	147389638	+	N	Y	Y	Y	Y	Y	del_ins_uksr	na	FoSTeS	intergenic;ir	NBPF7,PFN1P9	-,+	pseudogene	5519;2641	intergenic;ir	GJA8,GPR89B	+,+	protein_cod	8245;10868
762	chr1	144896297	+	chr1	144901047	+	Y	Y	Y	Y	Y	Y	del_ins_uksr	na	NHEJ	intronic	PDE4DIP	-	protein_cod	0	UTR3	PDE4DIP	-	protein_cod	0
763	chr1	157173864	+	chr1	157175397	+	Y	Y	Y	Y	Y	Y	del_ins_uksr	na	FoSTeS	intergenic	RP11-71G12.1,RP11-8	+,-	lincRNA,linc	16669;28157	intergenic	RP11-71G12.1,RP11-85(	+,-	lincRNA,linc	18202;26624
764	chr1	179607288	+	chr1	179608060	+	Y	Y	Y	Y	Y	N	del_ins_uksr	na	NHEJ	intronic	TDRD5	+	protein_cod	0	intronic	TDRD5	+	protein_cod	0
765	chr1	194451064	+	chr1	194454313	+	Y	N	N	N	N	N	del_ins_uksr	na	NHEJ	intergenic	RP11-476H20.1,RNU6	+,-	lincRNA,snF	129508;6169	intergenic;ir	RP11-476H20.1,RNU6-9	+,-	lincRNA,snF	132757;2920
766	chr1	207292347	+	chr1	207293196	+	N	N	N	N	N	Y	del_ins_uksr	na	NHEJ	intronic	C4BPA	+	protein_cod	0	intronic	C4BPA	+	protein_cod	0
767	chr1	207292362	+	chr1	207292854	+	Y	Y	Y	Y	Y	N	del_ins_uksr	na	NHEJ	intronic	C4BPA	+	protein_cod	0	intronic	C4BPA	+	protein_cod	0
768	chr1	222558223	+	chr1	222561017	+	N	Y	Y	Y	Y	Y	del_ins_uksr	na	NHEJ	ncRNA_intr	RP11-400N13.1	-	lincRNA	0	upstream	RP11-400N13.1	-	lincRNA	0
769	chr1	225132981	+	chr1	225248843	+	Y	N	N	N	N	N	del_ins_uksr	na	NHEJ	intronic	DNAH14	+	protein_cod	0	intronic	DNAH14	+	protein_cod	0
770	chr1	240392999	+	chr1	240394783	+	N	Y	N	N	Y	Y	del_ins_uksr	na	NHEJ	intronic	FMN2	+	protein_cod	0	intronic	FMN2	+	protein_cod	0
771	chr10	4708518	+	chr10	4710523	+	Y	Y	Y	Y	Y	Y	del_ins_uksr	na	NHEJ	ncRNA_intr	LINC00704	-	lincRNA	0	ncRNA_intr	LINC00704	-	lincRNA	0
772	chr10	25492525	+	chr10	25492952	+	N	Y	Y	Y	Y	N	del_ins_uksr	na	NHEJ	intronic	GPR158	+	protein_cod	0	intronic	GPR158	+	protein_cod	0
773	chr10	27638254	+	chr10	27642097	+	Y	Y	Y	Y	Y	Y	del_ins_uksr	na	NHEJ	downstream	FAM210CP	-	pseudogene	0	intergenic	FAM210CP,TRIAP1P1	-,-	pseudogene	2878;24970
774	chr10	28663803	+	chr10	28665096	+	Y	Y	Y	Y	Y	Y	del_ins_uksr	na	FoSTeS	intergenic	ZNF101P1,U3	-,-	pseudogene	33932;33166	intergenic	ZNF101P1,U3	-,-	pseudogene	35225;31873
775	chr10	42673128	+	chr10	42673432	+	N	N	N	Y	N	N	del_ins_uksr	na	FoSTeS	intergenic	KSR1P1,IGKV1OR10-	-,+	pseudogene	28131;7659	intergenic;ir	KSR1P1,IGKV1OR10-1	-,+	pseudogene	28435;7355
776	chr10	42673287	+	chr10	42673432	+	N	N	N	N	N	Y	del_ins_uksr	na	FoSTeS	intergenic	KSR1P1,IGKV1OR10-	-,+	pseudogene	28290;7500	intergenic;ir	KSR1P1,IGKV1OR10-1	-,+	pseudogene	28435;7355
777	chr10	55893665	+	chr10	55894133	+	Y	Y	Y	Y	Y	Y	del_ins_uksr	na	NHEJ	intronic	PCDH15	-	protein_cod	0	intronic	PCDH15	-	protein_cod	0
778	chr10	67032386	+	chr10	67032802	+	Y	Y	Y	Y	Y	Y	del_ins_uksr	na	FoSTeS	intergenic	RP11-252C24.2,RP11-	-,+	pseudogene	23836;41157	intergenic	RP11-252C24.2,RP11-42	-,+	pseudogene	24252;40741
779	chr10	81092182	+	chr10	81092781	+	Y	Y	Y	Y	Y	Y	del_ins_uksr	na	FoSTeS	intergenic	ZMIZ1,PPIF	+,+	protein_cod	15906;15043	intergenic	ZMIZ1,PPIF	+,+	protein_cod	16505;14444
780	chr10	92461854	+	chr10	92465630	+	N	Y	Y	Y	Y	Y	del_ins_uksr	na	NHEJ	intergenic	RP11-15K3.1,HTR7	-,-	lincRNA,pro	161292;38724	intergenic	RP11-15K3.1,HTR7	-,-	lincRNA,pro	165068;34948
781	chr10	94134599	+	chr10	94137654	+	Y	Y	Y	Y	Y	Y	del_ins_uksr	na	FoSTeS	intergenic	MARCH5,MARK2P9	+,+	protein_cod	20878;43825	intergenic	MARCH5,MARK2P9	+,+	protein_cod	23933;40770
782	chr10	95545477	+	chr10	95546496	+	Y	Y	Y	Y	Y	Y	del_ins_uksr	na	FoSTeS	intronic	LGI1	+	protein_cod	0	intronic	LGI1	+	protein_cod	0
783	chr10	124216820	+	chr10	124217264	+	Y	Y	Y	Y	Y	Y	del_ins_uksr	na	FoSTeS	UTR3	ARMS2	+	protein_cod	0	downstream	ARMS2	+	protein_cod	0
784	chr11	5760087	+	chr11	5762366	+	Y	Y	Y	Y	Y	N	del_ins_uksr	na	NHEJ	intronic	TRIM5	-	protein_cod	0	intronic	TRIM5	-	protein_cod	0
785	chr11	6881365	+	chr11	6881965	+	Y	Y	Y	Y	Y	Y	del_ins_uksr	na	FoSTeS	intergenic	OR10A5,OR10A2	+,+	protein_cod	13429;9559	intergenic	OR10A5,OR10A2	+,+	protein_cod	14029;8959
786	chr11	29007202	+	chr11	29012889	+	Y	Y	Y	Y	Y	Y	del_ins_uksr	na	FoSTeS	ncRNA_intr	RP11-115J23.1	+	lincRNA	0	ncRNA_intr	RP11-115J23.1	+	lincRNA	0
787	chr11	39795370	+	chr11	39796866	+	Y	N	N	Y	Y	Y	del_ins_uksr	na	NHEJ	intergenic;ir	AC027806.1,AC02174	+,-	miRNA,miR	42917;87686	intergenic;ir	AC027806.1,AC021749.1	+,-	miRNA,miR	44413;86190
788	chr11	47057588	+	chr11	47063707	+	Y	Y	Y	Y	Y	Y	del_ins_uksr	na	FoSTeS	intronic	C11orf49	+	protein_cod	0	intronic	C11orf49	+	protein_cod	0
789	chr11	47660351	+	chr11	47663929	+	Y	N	N	N	N	N	del_ins_uksr	na	FoSTeS	exonic	MTCH2	-	protein_cod	0	splicing	MTCH2	-	protein_cod	0
790	chr11	63698350	+	chr11	63702022	+	Y	N	N	N	N	N	del_ins_uksr	na	FoSTeS	intergenic	RCOR2,NAA40	-,+	protein_cod	14034;8118	intergenic	RCOR2,NAA40	-,+	protein_cod	17706;4446
791	chr11	63698907	+	chr11	63701944	+	N	N	Y	N	N	N	del_ins_uksr	na	FoSTeS	intergenic	RCOR2,NAA40	-,+	protein_cod	14591;7561	intergenic	RCOR2,NAA40	-,+	protein_cod	17628;4524
792	chr11	128682716	+	chr11	128683411	+	Y	Y	Y	N	Y	Y	del_ins_uksr	na	NHEJ	UTR3	FLI1	+	protein_cod	0	downstream	FLI1	+	protein_cod	0
793	chr12	7018316	+	chr12	7018676	+	N	N	N	N	Y	N	del_ins_uksr	na	VNTR	intronic	LRRC23	+	protein_cod	0	intronic	LRRC23	+	protein_cod	0
794	chr12	11173687	+	chr12	11200350	+	N	N	Y	Y	Y	Y	del_ins_uksr	na	FoSTeS	downstream	TAS2R19	-	protein_cod	0	downstream	TAS2R63P	-	pseudogene	0
795	chr12	11211280	+	chr12	11211924	+	N	N	N	Y	N	N	del_ins_uksr	na	NHEJ	intergenic;ir	TAS2R63P,TAS2R46	-,-	pseudogene	9425;2684	intergenic	TAS2R63P,TAS2R46	-,-	pseudogene	10069;2040
796	chr12	11211280	+	chr12	11242125	+	N	N	Y	N	N	N	del_ins_uksr	na	NHEJ	intergenic;ir	TAS2R63P,TAS2R46	-,-	pseudogene	9425;2684	intergenic	TAS2R64P,TAS2R43	-,-	pseudogene	10355;1761
797	chr12	11211280	+	chr12	11283949	+	N	N	N	N	N	Y	del_ins_uksr	na	NHEJ	intergenic;ir	TAS2R63P,TAS2R46	-,-	pseudogene	9425;2684	intergenic	RP11-673D15.7,TAS2R3	-,-	pseudogene	34076;1608
798	chr12	11237390	+	chr12	11279318	+	N	N	N	N	N	Y	del_ins_uksr	na	FoSTeS	intergenic	TAS2R64P,TAS2R43	-,-	pseudogene	5620;6496	intergenic	RP11-673D15.7,TAS2R3	-,-	pseudogene	29445;6239
799	chr12	16420123	+	chr12	16421284	+	Y	Y	Y	Y	Y	Y	del_ins_uksr	na	NHEJ	intronic	SLC15A5	-	protein_cod	0	intronic	SLC15A5	-	protein_cod	0
800	chr12	42512966	+	chr12	42538226	+	Y	N	N	N	N	N	del_ins_uksr	na	NHEJ	exonic	GXYLT1	-	protein_cod	0	splicing	GXYLT1	-	protein_cod	0
801	chr12	45967248	+	chr12	45967630	+	N	Y	Y	N	Y	Y	del_ins_uksr	na	FoSTeS	ncRNA_intr	RP11-352M15.1	-	lincRNA	0	ncRNA_intr	RP11-352M15.1	-	lincRNA	0
802	chr12	54840542	+	chr12	54841195	+	Y	Y	Y	Y	Y	Y	del_ins_uksr	na	NHEJ	ncRNA_intr	RP11-753H16.5,RP11-	+,+	antisense,ar	0	ncRNA_intr	RP11-753H16.5,RP11-75	+,+	antisense,ar	0
803	chr12	96010456	+	chr12	96012329	+	N	N	N	Y	N	N	del_ins_uksr	na	FoSTeS	intergenic	USP44,Y_RNA	-,+	protein_cod	65190;1009	downstream	Y_RNA	+	misc_RNA	0
804	chr13	21732265	+	chr13	21735929	+	Y	N	Y	Y	N	Y	del_ins_uksr	na	FoSTeS	splicing	SKA3	-	protein_cod	0	exonic	SKA3	-	protein_cod	0
805	chr13	21734127	+	chr13	21742127	+	N	Y	Y	Y	Y	Y	del_ins_uksr	na	FoSTeS	splicing	SKA3	-	protein_cod	0	exonic	SKA3	-	protein_cod	0
806	chr13	65133014	+	chr13	65133674	+	Y	Y	Y	Y	Y	Y	del_ins_uksr	na	NHEJ	intergenic	LINC00355,LGMNP1	-,-	lincRNA,pse	482870;399215	intergenic	LINC00355,LGMNP1	-,-	lincRNA,pse	483530;398555
807	chr13	68936888	+	chr13	68937267	+	Y	Y	Y	Y	Y	Y	del_ins_uksr	na	NHEJ	intergenic	RPL37P21,RPS3AP52	-,+	pseudogene	31143;271434	intergenic	RPL37P21,RPS3AP52	-,+	pseudogene	31522;271055
808	chr13	72843037	+	chr13	72846925	+	Y	Y	Y	Y	Y	Y	del_ins_uksr	na	NHEJ	intergenic	RNA5SP32,SNORD37	+,-	rRNA,snoRI	290693;185003	intergenic	RNA5SP32,SNORD37	+,-	rRNA,snoRI	294581;181115
809	chr13	73299597	+	chr13	73300306	+	Y	Y	Y	Y	Y	Y	del_ins_uksr	na	NHEJ	intronic	MZT1	-	protein_cod	0	intronic	MZT1	-	protein_cod	0
810	chr13	93056847	+	chr13	93057423	+	Y	Y	Y	Y	Y	Y	del_ins_uksr	na	NHEJ	intronic	GPC5	+	protein_cod	0	intronic	GPC5	+	protein_cod	0
811	chr14	21377300	+	chr14	21379532	+	N	N	Y	N	Y	Y	del_ins_uksr	na	FoSTeS	ncRNA_intr	RP11-219E7.4	-	lincRNA	0	ncRNA_intr	RP11-219E7.4	-	lincRNA	0
812	chr14	21952624	+	chr14	21954663	+	Y	Y	Y	Y	Y	Y	del_ins_uksr	na	NHEJ	intronic	TOX4	+	protein_cod	0	intronic	TOX4	+	protein_cod	0
813	chr14	50288026	+	chr14	50288354	+	N	N	Y	N	N	N	del_ins_uksr	na	FoSTeS	intronic	NEMF	-	protein_cod	0	intronic	NEMF	-	protein_cod	0
814	chr14	53252467	+	chr14	53254935	+	N	Y	N	N	N	N	del_ins_uksr	na	NHEJ	ncRNA_exo	RN7SL588P	+	misc_RNA	0	intronic	GNPNAT1	-	protein_cod	0
815	chr14	77687154	+	chr14	77687596	+	N	Y	N	N	N	N	del_ins_uksr	na	FoSTeS	intronic	TMEM63C,RP11-463C	+,+	protein_cod	0	intronic	TMEM63C,RP11-463C8.	+,+	protein_cod	0
816	chr14	77687238	+	chr14	77687450	+	N	N	N	Y	N	N	del_ins_uksr	na	VNTR	intronic	TMEM63C,RP11-463C	+,+	protein_cod	0	intronic	TMEM63C,RP11-463C8.	+,+	protein_cod	0
817	chr14	81334185	+	chr14	81340109	+	N	Y	Y	Y	Y	Y	del_ins_uksr	na	NHEJ	intronic	CEP128	-	protein_cod	0	intronic	CEP128	-	protein_cod	0
818	chr14	81878211	+	chr14	81880613	+	Y	Y	Y	Y	Y	Y	del_ins_uksr	na	FoSTeS	intronic	STON2	-	protein_cod	0	intronic	STON2	-	protein_cod	0
819	chr14	82499110	+	chr14	82503331	+	Y	Y	Y	Y	Y	Y	del_ins_uksr	na	NHEJ	intergenic	RP11-666E17.1,snoU1	+,+	lincRNA,snc	2417;429322	intergenic	RP11-666E17.1,snoU13	+,+	lincRNA,snc	6638;425101
820	chr14	106471857	+	chr14	106774647	+	N	Y	Y	Y	N	Y	del_ins_uksr	na	FoSTeS	upstream	IGHV1-3	-	IG_V_gene	0	upstream	IGHV7-27	-	IG_V_pseu	0
821	chr15	22336853	+	chr15	22344094	+	Y	Y	Y	Y	Y	Y	del_ins_uksr	na	NHEJ	ncRNA_intr	RP11-69H14.6	+	sense_over	0	ncRNA_intr	RP11-69H14.6	+	sense_over	0
822	chr15	34200447	+	chr15	34200715	+	N	N	Y	N	N	N	del_ins_uksr	na	FoSTeS	intronic	AVEN	-	protein_cod	0	intronic	AVEN	-	protein_cod	0
823	chr15	41851540	+	chr15	41853322	+	N	N	Y	N	N	Y	del_ins_uksr	na	FoSTeS	exonic;exon	TYRO3	+	protein_cod	0	intronic;intrc	TYRO3	+	protein_cod	0
824	chr15	71881569	+	chr15	71882967	+	Y	Y	Y	Y	Y	Y	del_ins_uksr	na	NHEJ	intronic	THSD4	+	protein_cod	0	intronic	THSD4	+	protein_cod	0
825	chr15	84541434	+	chr15	84543849	+	Y	Y	Y	Y	Y	Y	del_ins_uksr	na	NHEJ	intronic	ADAMTSL3	+	protein_cod	0	intronic	ADAMTSL3	+	protein_cod	0
826	chr16	1290146	+	chr16	1295289	+	Y	Y	Y	Y	Y	Y	del_ins_uksr	na	NHEJ	upstream	TPSAB1	+	protein_cod	0	intergenic	TPSAB1,TPSD1	+,+	protein_cod	2734;10771
827	chr16	8614383	+	chr16	8614542	+	N	N	N	N	N	Y	del_ins_uksr	na	VNTR	intergenic	RP11-483K5.3,TMEM1	-,-	lincRNA,pro	32368;5119	intergenic	RP11-483K5.3,TMEM114	-,-	lincRNA,pro	32527;4960
828	chr16	31185721	+	chr16	31185920	+	N	Y	N	N	N	N	del_ins_uksr	na	FoSTeS	upstream	NDUFA3P6	+	pseudogene	0	ncRNA_exo	NDUFA3P6	+	pseudogene	0
829	chr16	32371608	+	chr16	32371789	+	N	Y	N	N	N	N	del_ins_uksr	na	FoSTeS	ncRNA_intr	RP11-17M15.4	-	pseudogene	0	ncRNA_intr	RP11-17M15.4	-	pseudogene	0
830	chr16	33374049	+	chr16	33379406	+	N	Y	Y	Y	N	N	del_ins_uksr	na	NHEJ	upstream	RP11-23E10.4	-	pseudogene	0	upstream	RP11-293B20.3	+	pseudogene	0
831	chr16	57366311	+	chr16	57367119	+	Y	Y	N	Y	N	Y	del_ins_uksr	na	NHEJ	ncRNA_exo	CTC-661I16.2	-	pseudogene	0	upstream;up	CTC-661I16.2	-	pseudogene	0
832	chr16	76539133	+	chr16	76544026	+	Y	Y	Y	Y	N	N	del_ins_uksr	na	NHEJ	intronic	CNTNAP4	+	protein_cod	0	intronic	CNTNAP4	+	protein_cod	0
833	chr16	78876652	+	chr16	78877578	+	Y	Y	N	Y	N	N	del_ins_uksr	na	NHEJ	intronic	WWOX	+	protein_cod	0	intronic	WWOX	+	protein_cod	0
834	chr16	88240948	+	chr16	88241057	+	N	Y	N	N	N	N	del_ins_uksr	na	FoSTeS	intergenic	LA16c-444G7.2,LA16c	+,+	lincRNA,linc	12125;27443	intergenic	LA16c-444G7.2,LA16c-4	+,+	lincRNA,linc	12234;27334

835	chr16	88862632	+	chr16	88863602	+	Y	Y	Y	Y	Y	Y	del_ins_uksr	na	FoSTeS	intergenic	PIEZO1,CDT1	-,+	protein_cod	11013;6989	intergenic	PIEZO1,CDT1	-,+	protein_cod	11983;6019
836	chr17	1654669	+	chr17	1655401	+	N	N	Y	N	Y	N	del_ins_uksr	na	NHEJ	intronic	SERPINF2	+	protein_cod	0	intronic	SERPINF2	+	protein_cod	0
837	chr17	11411667	+	chr17	11412338	+	N	N	N	N	Y	Y	del_ins_uksr	na	FoSTeS	intronic;intr	SHISA6	+	protein_cod	0	intronic;intr	SHISA6	+	protein_cod	0
838	chr17	15183463	+	chr17	15185720	+	N	Y	Y	Y	Y	Y	del_ins_uksr	na	NHEJ	upstream;u	AC005703.2	-	lincRNA	0	intergenic;i	AC005703.2,TEKT3	-,-	lincRNA,pro	2926;21408
839	chr17	15882895	+	chr17	15883151	+	N	N	N	Y	N	N	del_ins_uksr	na	FoSTeS	intronic	ZSWIM7	-	protein_cod	0	intronic	ZSWIM7	-	protein_cod	0
840	chr17	16505449	+	chr17	16506299	+	Y	N	N	Y	N	Y	del_ins_uksr	na	NHEJ	intergenic	ZNF287,AC005822.1	-,-	protein_cod	32929;14713	intergenic	ZNF287,AC005822.1	-,-	protein_cod	33779;13863
841	chr17	21321277	+	chr17	21322822	+	Y	N	Y	Y	Y	Y	del_ins_uksr	na	NHEJ	UTR3;UTR	KCNJ12	+	protein_cod	0	UTR3;UTR	KCNJ12	+	protein_cod	0
842	chr17	21556719	+	chr17	21557917	+	Y	N	N	N	Y	N	del_ins_uksr	na	NHEJ	intergenic	AC144838.2,AC14483	+,-	pseudogene	6740;3829	intergenic	AC144838.2,AC144838.	+,-	pseudogene	7938;2631
843	chr17	35250486	+	chr17	35250929	+	Y	Y	Y	Y	Y	Y	del_ins_uksr	na	NHEJ	ncRNA_intr	RP11-445F12.1	-	antisense	0	ncRNA_intr	RP11-445F12.1	-	antisense	0
844	chr17	41383489	+	chr17	41466011	+	Y	Y	Y	Y	Y	Y	del_ins_uksr	na	FoSTeS	upstream	LINC00854	-	processed_	0	ncRNA_exo	LINC00910	-	lincRNA	0
845	chr17	41466841	+	chr17	41467031	+	N	N	N	N	Y	N	del_ins_uksr	na	FoSTeS	upstream	LINC00910	-	lincRNA	0	upstream	LINC00910	-	lincRNA	0
846	chr17	47352338	+	chr17	47353126	+	N	N	N	N	Y	N	del_ins_uksr	na	FoSTeS	intergenic	RP1-62O9.3,ZNF652	+,-	processed_	16314;14232	intergenic	RP1-62O9.3,ZNF652	+,-	processed_	17102;13444
847	chr17	51694036	+	chr17	51728488	+	N	Y	N	N	N	N	del_ins_uksr	na	NHEJ	intergenic	AC090079.1,RP11-312	+,-	miRNA,pse	79071;64955	intergenic	AC090079.1,RP11-312B	+,-	miRNA,pse	113523;30503
848	chr17	54949929	+	chr17	54952295	+	Y	Y	Y	N	N	Y	del_ins_uksr	na	FoSTeS	intergenic	RNU6-1158P,RP11-67	+,-	snRNA,pse	1292;12071	intergenic	RNU6-1158P,RP11-670E	+,-	snRNA,pse	3658;9705
849	chr17	57364158	+	chr17	57365707	+	N	Y	N	N	N	N	del_ins_uksr	na	FoSTeS	intergenic	SNRPGP17,YPEL2	+,+	pseudogene	5454;44905	intergenic	SNRPGP17,YPEL2	+,+	pseudogene	7003;43356
850	chr17	57870400	+	chr17	57871185	+	N	N	N	N	Y	N	del_ins_uksr	na	FoSTeS	intronic	VMP1	+	protein_cod	0	intronic;intr	VMP1	+	protein_cod	0
851	chr17	62986279	+	chr17	62988337	+	Y	Y	Y	Y	Y	Y	del_ins_uksr	na	FoSTeS	intergenic	AMZ2P1,GNA13	-,-	pseudogene	14585;20554	intergenic	AMZ2P1,GNA13	-,-	pseudogene	16643;18496
852	chr18	4955592	+	chr18	4955990	+	Y	Y	Y	Y	Y	Y	del_ins_uksr	na	NHEJ	ncRNA_intr	RP11-172F10.1	-	lincRNA	0	ncRNA_intr	RP11-172F10.1	-	lincRNA	0
853	chr18	5209648	+	chr18	5210659	+	Y	Y	Y	Y	Y	Y	del_ins_uksr	na	NHEJ	intergenic	C18orf42,LINC00667	-,+	protein_cod	12146;23227	intergenic	C18orf42,LINC00667	-,+	protein_cod	13157;22216
854	chr18	32530959	+	chr18	32531237	+	N	N	Y	N	N	N	del_ins_uksr	na	NHEJ	intergenic	DTNA,MAPRE2	+,+	protein_cod	59151;25933	intergenic	DTNA,MAPRE2	+,+	protein_cod	59429;25655
855	chr18	35306060	+	chr18	35306633	+	Y	Y	Y	Y	Y	Y	del_ins_uksr	na	NHEJ	intergenic	RP11-19F9.1,RP11-68	+,-	pseudogene	52921;929113	intergenic	RP11-19F9.1,RP11-687D	+,-	pseudogene	53494;928540
856	chr18	38864784	+	chr18	38868344	+	N	N	Y	Y	N	N	del_ins_uksr	na	NHEJ	intergenic	RNU7-145P,RP11-142	+,-	snRNA,linc	811938;182611	intergenic	RNU7-145P,RP11-142I2	+,-	snRNA,linc	815498;179051
857	chr18	49196970	+	chr18	49198874	+	N	N	N	Y	N	N	del_ins_uksr	na	FoSTeS	intergenic	RSL24D1P9,RP11-44	-,-	pseudogene	57490;16925	intergenic	RSL24D1P9,RP11-440L1	-,-	pseudogene	59394;15021
858	chr18	55345792	+	chr18	55346136	+	Y	N	N	N	N	N	del_ins_uksr	na	FoSTeS	ncRNA_intr	RP11-35G9.3,RP11-35	+,+	antisense,a	0	ncRNA_intr	RP11-35G9.3,RP11-35G	+,+	antisense,a	0
859	chr18	73901668	+	chr18	73902041	+	Y	Y	Y	Y	Y	Y	del_ins_uksr	na	NHEJ	intergenic	RP11-94B19.6,RP11-9	-,+	lincRNA,linc	11975;19797	intergenic	RP11-94B19.6,RP11-94B	-,+	lincRNA,linc	12348;19424
860	chr18	74347196	+	chr18	74347998	+	Y	Y	Y	Y	Y	Y	del_ins_uksr	na	FoSTeS	ncRNA_intr	RP11-111H3.3	-	lincRNA	0	ncRNA_intr	RP11-111H3.3	-	lincRNA	0
861	chr19	4885732	+	chr19	4885945	+	N	N	Y	N	N	N	del_ins_uksr	na	FoSTeS	intergenic	AC027319.2,AC02731	+,-	miRNA,miR	8922;3483	intergenic	AC027319.2,AC027319.	+,-	miRNA,miR	9135;3270
862	chr19	15046544	+	chr19	15049144	+	N	N	N	N	Y	N	del_ins_uksr	na	FoSTeS	intergenic	OR7A15P,OR7C2	+,+	pseudogene	7708;5757	intergenic	OR7A15P,OR7C2	+,+	pseudogene	10308;3157
863	chr19	23140801	+	chr19	23142920	+	Y	Y	Y	Y	Y	Y	del_ins_uksr	na	FoSTeS	intergenic	CTD-2579N5.4,ZNF72	+,-	pseudogene	32452;17469	intergenic	CTD-2579N5.4,ZNF728	+,-	pseudogene	34571;15350
864	chr19	23506167	+	chr19	23648639	+	Y	Y	N	N	N	N	del_ins_uksr	na	FoSTeS	upstream	CTB-176F20.3	+	antisense	0	intergenic	VN1R92P,ZNF725P	+,-	pseudogene	20648;26591
865	chr2	442188	+	chr2	442361	+	N	N	N	N	N	Y	del_ins_uksr	na	FoSTeS	intergenic	AC105393.2,AC09332	+,-	lincRNA,linc	19885;48756	intergenic	AC105393.2,AC093326.	+,-	lincRNA,linc	20058;48583
866	chr2	1723768	+	chr2	1724276	+	N	N	N	N	N	Y	del_ins_uksr	na	FoSTeS	intronic	PXDN	-	protein_cod	0	intronic	PXDN	-	protein_cod	0
867	chr2	14531291	+	chr2	14531619	+	N	Y	N	Y	Y	Y	del_ins_uksr	na	NHEJ	ncRNA_intr	LINC00276	-	lincRNA	0	ncRNA_intr	LINC00276	-	lincRNA	0
868	chr2	16406393	+	chr2	16407988	+	Y	Y	Y	Y	Y	Y	del_ins_uksr	na	NHEJ	ncRNA_intr	AC010745.2	+	lincRNA	0	ncRNA_intr	AC010745.2	+	lincRNA	0
869	chr2	19002874	+	chr2	19003392	+	Y	Y	Y	Y	N	Y	del_ins_uksr	na	FoSTeS	intergenic	AC106053.1,AC09259	+,-	lincRNA,linc	33856;164855	intergenic	AC106053.1,AC092594.	+,-	lincRNA,linc	34374;164337
870	chr2	31725168	+	chr2	31727398	+	N	N	Y	N	N	Y	del_ins_uksr	na	FoSTeS	intergenic	XDH,SRD5A2	-,-	protein_cod	87587;22382	intergenic	XDH,SRD5A2	-,-	protein_cod	89817;20152
871	chr2	51973823	+	chr2	51974241	+	Y	Y	Y	Y	Y	Y	del_ins_uksr	na	NHEJ	ncRNA_intr	AC007682.1	+	lincRNA	0	ncRNA_intr	AC007682.1	+	lincRNA	0
872	chr2	52749686	+	chr2	52785271	+	Y	Y	Y	Y	Y	Y	del_ins_uksr	na	NHEJ	intergenic	AC139712.4,AC13971	-,+	lincRNA,pse	21796;48100	intergenic	AC139712.4,AC139712.	-,+	lincRNA,pse	57381;12515
873	chr2	54565493	+	chr2	54567474	+	Y	Y	Y	Y	Y	Y	del_ins_uksr	na	NHEJ	intronic	C2orf73	+	protein_cod	0	intronic	C2orf73	+	protein_cod	0
874	chr2	56652302	+	chr2	56655883	+	Y	Y	Y	Y	Y	Y	del_ins_uksr	na	NHEJ	intergenic	CCDC85A,AC008173.	+,+	protein_cod	38994;325133	intergenic	CCDC85A,AC008173.1	+,+	protein_cod	42575;321552
875	chr2	60302516	+	chr2	233285640	+	N	N	N	N	N	Y	del_ins_uksr	na	NHEJ	ncRNA_intr	RP11-444A22.1	-	lincRNA	0	ncRNA_exo	AC068134.6	-	lincRNA	0
876	chr2	76773553	+	chr2	76775453	+	N	N	Y	Y	N	N	del_ins_uksr	na	NHEJ	intergenic;i	RN7SKP203,RN7SKP	-,-	misc_RNA,	101017;48986	intergenic;i	RN7SKP203,RN7SKP16	-,-	misc_RNA,	102917;47086
877	chr2	100103713	+	chr2	100105032	+	Y	Y	Y	Y	Y	Y	del_ins_uksr	na	FoSTeS	intronic	REV1	-	protein_cod	0	intronic	REV1	-	protein_cod	0
878	chr2	106879557	+	chr2	106885916	+	Y	Y	Y	Y	Y	Y	del_ins_uksr	na	NHEJ	intergenic	AC092106.1,AC11475	-,-	pseudogene	9384;25702	intergenic	AC092106.1,AC114755.	-,-	pseudogene	15743;19343
879	chr2	124170256	+	chr2	124170587	+	N	Y	Y	Y	Y	Y	del_ins_uksr	na	NHEJ	intergenic;i	AC062020.1,AC07340	-,-	lincRNA,linc	339199;30586	intergenic;i	AC062020.1,AC073409.	-,-	lincRNA,linc	339530;30255
880	chr2	130824430	+	chr2	130824860	+	N	N	N	N	Y	N	del_ins_uksr	na	FoSTeS	intergenic	FAR2P1,POTEF	-,-	pseudogene	15726;6678	intergenic	FAR2P1,POTEF	-,-	pseudogene	16156;6248
881	chr2	189119619	+	chr2	189120108	+	N	Y	N	N	N	N	del_ins_uksr	na	FoSTeS	ncRNA_intr	LINC01090	-	lincRNA	0	ncRNA_intr	LINC01090	-	lincRNA	0
882	chr2	189119619	+	chr2	189120983	+	Y	N	Y	Y	Y	Y	del_ins_uksr	na	FoSTeS	ncRNA_intr	LINC01090	-	lincRNA	0	ncRNA_intr	LINC01090	-	lincRNA	0
883	chr2	241504580	+	chr2	241505855	+	Y	Y	Y	Y	Y	Y	del_ins_uksr	na	NHEJ	intronic	ANKMY1	-	protein_cod	0	intronic	RNPEPL1,ANKMY1	+,-	protein_cod	0
884	chr2	241781957	+	chr2	241782217	+	N	N	Y	N	N	N	del_ins_uksr	na	FoSTeS	intergenic	KIF1A,AGXT	-,+	protein_cod	22232;25939	intergenic	KIF1A,AGXT	-,+	protein_cod	22492;25679
885	chr2	242506103	+	chr2	242507335	+	Y	Y	N	Y	Y	Y	del_ins_uksr	na	NHEJ	intronic;intr	BOK	+	protein_cod	0	intronic;intr	BOK	+	protein_cod	0
886	chr20	18190831	+	chr20	18191025	+	N	N	N	Y	N	N	del_ins_uksr	na	FoSTeS	intergenic	CSRP2BP,RP4-717M2	+,+	protein_cod	21800;9926	intergenic	CSRP2BP,RP4-717M23.	+,+	protein_cod	21994;9732
887	chr20	26102844	+	chr20	26103502	+	Y	N	N	Y	N	N	del_ins_uksr	na	NHEJ	intergenic;i	NCOR1P1,RP13-329D	-,-	pseudogene	8180;10679	intergenic;i	NCOR1P1,RP13-329D4.	-,-	pseudogene	8838;10021
888	chr20	26257678	+	chr20	26258265	+	Y	Y	Y	Y	Y	Y	del_ins_uksr	na	NHEJ	intergenic	MIR663A,NONE	-,NONE	processed_	25516;NONE	intergenic	MIR663A,NONE	-,NONE	processed_	26103;NONE
889	chr20	37545036	+	chr20	37545305	+	Y	Y	Y	Y	Y	N	del_ins_uksr	na	FoSTeS	intronic	PPP1R16B	+	protein_cod	0	intronic	PPP1R16B	+	protein_cod	0
890	chr20	61724698	+	chr20	61725598	+	Y	Y	Y	Y	Y	Y	del_ins_uksr	na	NHEJ	intergenic	RP11-305P22.9,HAR1	+,-	lincRNA,linc	8275;2147	intergenic	RP11-305P22.9,HAR1B	+,-	lincRNA,linc	9175;1247
891	chr21	10945339	+	chr21	11177588	+	N	N	N	N	Y	N	del_ins_uksr	na	FoSTeS	intronic	TPTE	-	protein_cod	0	ncRNA_intr	AP003900.6	+	lincRNA	0
892	chr21	15460789	+	chr21	15461865	+	Y	Y	Y	N	Y	Y	del_ins_uksr	na	NHEJ	ncRNA_intr	AP001347.6	+	antisense	0	ncRNA_intr	AP001347.6	+	antisense	0
893	chr21	23654900	+	chr21	23666004	+	Y	Y	Y	Y	Y	Y	del_ins_uksr	na	NHEJ	ncRNA_intr	AP000705.7	+	lincRNA	0	ncRNA_intr	AP000705.7	+	lincRNA	0
894	chr21	24374796	+	chr21	24375421	+	Y	Y	Y	Y	Y	Y	del_ins_uksr	na	NHEJ	intergenic	AP000949.1,AP00125	+,+	pseudogene	118475;63017	intergenic	AP000949.1,AP001255.	+,+	pseudogene	119100;62392
895	chr21	30264168	+	chr21	30264286	+	N	N	N	N	Y	N	del_ins_uksr	na	FoSTeS	intergenic	HSPD1P7,THUMPD1P	-,-	pseudogene	2574;9933	intergenic	HSPD1P7,THUMPD1P1	-,-	pseudogene	2692;9815
896	chr21	30382818	+	chr21	37890832	+	N	Y	Y	N	N	Y	del_ins_uksr	na	FoSTeS	intronic	RWDD2B	-	protein_cod	0	ncRNA_intr	AP000695.4	+	antisense	0
897	chr21	37821541	+	chr21	37825965	+	Y	N	Y	N	N	Y	del_ins_uksr	na	NHEJ	ncRNA_intr	AP000695.6,AP00069	+,+	antisense,a	0	ncRNA_intr	AP000695.6,AP000695.4	+,+	antisense,a	0
898	chr22	35459001	+	chr22	35459422	+	Y	Y	Y	Y	Y	Y	del_ins_uksr	na	NHEJ	ncRNA_intr	RP1-272J12.1	-	antisense	0	ncRNA_intr	RP1-272J12.1	-	antisense	0
899	chr22	45300384	+	chr22	45300746	+	N	Y	Y	Y	Y	N	del_ins_uksr	na	FoSTeS	intronic	PHF21B	-	protein_cod	0	intronic	PHF21B	-	protein_cod	0
900	chr22	47781989	+	chr22	47782205	+	N	N	N	N	Y	N	del_ins_uksr	na	NHEJ	intergenic	CITF22-49D8.1,LL22N	+,-	lincRNA,pro	12698;75059	intergenic	CITF22-49D8.1,LL22NC	+,-	lincRNA,pro	12914;74843
901	chr22	49034331	+	chr22	49034450	+	N	N	N	Y	N	N	del_ins_uksr	na	FoSTeS	intronic	FAM19A5	+	protein_cod	0	intronic;intr	FAM19A5	+	protein_cod	0
902	chr3	6169030	+	chr3	6187098	+	Y	Y	Y	Y	N	Y	del_ins_uksr	na	NHEJ	intergenic;i	AC027119.1,AC02616	+,+	lincRNA,linc	3223;21141	intergenic;i	AC027119.1,AC026167.1	+,+	lincRNA,linc	21291;3073
903	chr3	10060296	+	chr3	10060731	+	N	Y	Y	Y	Y	Y	del_ins_uksr	na	NHEJ	ncRNA_intr	CIDECP	-	pseudogene	0	ncRNA_intr	CIDECP	-	pseudogene	0
904	chr3	11645733	+	chr3	11646551	+	N	Y	N	N	Y	N	del_ins_uksr	na	NHEJ	intronic;intr	VGLL4	-	protein_cod	0	intronic	VGLL4	-	protein_cod	0
905	chr3	11645733	+	chr3	11646673	+	Y	N	Y	Y	N	Y	del_ins_uksr	na	NHEJ	intronic;intr	VGLL4	-	protein_cod	0	intronic	VGLL4	-	protein_cod	0
906	chr3	18982827	+	chr3	18983318	+	Y	N	N	N	N	N	del_ins_uksr	na	NHEJ	intergenic	AC144521.1,RNU6-13	+,-	processed_	23643;13608	intergenic	AC144521.1,RNU6-138P	+,-	processed_	24134;13117
907	chr3	20653871	+	chr3	20654261	+	Y	Y	N	Y	Y	Y	del_ins_uksr	na	NHEJ	intergenic;i	RNU6-815P,AC104441	-,-	snRNA,miR	104623;525884	intergenic;i	RNU6-815P,AC104441.1	-,-	snRNA,miR	105013;525494
908	chr3	21723705	+	chr3	21725967	+	Y	N	N	Y	Y	Y	del_ins_uksr	na	FoSTeS	intronic;intr	ZNF385D	-	protein_cod	0	intronic;intr	ZNF385D	-	protein_cod	0
909	chr3	23685755	+	chr3	23686207	+	Y	Y	Y	Y	Y	Y	del_ins_uksr	na	NHEJ	intergenic	RNU6-788P,AC020626	-,+	snRNA,miR	2672;123347	intergenic	RNU6-788P,AC020626.1	-,+	snRNA,miR	3124;122895
910	chr3	25030887	+	chr3	25032250	+	Y	Y	Y	Y	Y	Y	del_ins_uksr	na	NHEJ	ncRNA_intr	AC133680.1	+	lincRNA	0	ncRNA_intr	AC133680.1	+	lincRNA	0
911	chr3	37978417	+	chr3	37986928	+	Y	Y	Y	Y	Y	Y	del_ins_uksr	na	NHEJ	intronic	CTDSPL	+	protein_cod	0	intronic	CTDSPL	+	protein_cod	0
912	chr3	57522710	+	chr3	57524052	+	N	Y	Y	N	N	N	del_ins_uksr	na	FoSTeS	intronic;intr	DNAH12	-	protein_cod	0	intronic;intr	DNAH12	-	protein_cod	0
913	chr3	67953750	+	chr3	68125694	+	N	N	N	N	N	Y	del_ins_uksr	na	FoSTeS	ncRNA_intr	RP11-81N13.1	+	lincRNA	0	intronic	FAM19A1	+	protein_cod	0
914	chr3	67954132	+	chr3	68125675	+	Y	N	N	N	N	N	del_ins_uksr	na	FoSTeS	ncRNA_intr	RP11-81N13.1	+	lincRNA	0	intronic	FAM19A1	+	protein_cod	0
915	chr3	79960808	+	chr3	79961263	+	N	Y	N	Y	N	Y	del_ins_uksr	na	FoSTeS	intergenic	ROBO1,HMGB1P38	-,-	protein_cod	143843;45908	intergenic	ROBO1,HMGB1P38	-,-	protein_cod	144298;45453
916	chr3	99628782	+	chr3	99629632	+	Y	Y	N	Y	Y	Y	del_ins_uksr	na	NHEJ	intronic;intr	FILIP1L,CMSS1	-,+	protein_cod	0	intronic;intr	FILIP1L,CMSS1	-,+	protein_cod	0
917	chr3	113405393	+	chr3	113406350	+	N	N	Y	N	N	N	del_ins_uksr	na	NHEJ	intronic	KIAA2018	-	protein_cod	0	intronic	KIAA2018	-	protein_cod	0
918	chr3	116204834	+	chr3	116205179	+	N	N	Y	N	N	N	del_ins_uksr	na	NHEJ	intronic	LSAMP	-	protein_cod	0	intronic	LSAMP	-	protein_cod	0

919	chr3	129657308	+	chr3	129657873	+	Y	Y	Y	Y	Y	Y	del_ins_uksr	na	FoSTeS	intergenic	TMCC1-AS1,RP11-93K	+,-	antisense,a	19890;15640	intergenic	TMCC1-AS1,RP11-93K2	+,-	antisense,a	20455;15075
920	chr3	145644968	+	chr3	145649191	+	Y	Y	Y	Y	Y	Y	del_ins_uksr	na	NHEJ	intergenic	RPL21P39,RP11-88H1	-,+	pseudogene	102442;12731	intergenic	RPL21P39,RP11-88H10.	-,+	pseudogene	106665;8508
921	chr3	159257021	+	chr3	159257691	+	Y	Y	Y	Y	Y	Y	del_ins_uksr	na	NHEJ	intronic	IQCJ-SCHIP1	+	protein_cod	0	intronic	IQCJ-SCHIP1	+	protein_cod	0
922	chr3	178878613	+	chr3	178883884	+	Y	Y	Y	Y	Y	Y	del_ins_uksr	na	NHEJ	intronic	PIK3CA	+	protein_cod	0	intronic	PIK3CA	+	protein_cod	0
923	chr4	19057471	+	chr4	19057970	+	Y	Y	Y	Y	Y	N	del_ins_uksr	na	FoSTeS	intergenic;ir	RP11-608B3.1,RP11-3	-,-	lincRNA,linc	566340;116487	intergenic;ir	RP11-608B3.1,RP11-3J1	-,-	lincRNA,linc	566839;115988
924	chr4	61330106	+	chr4	61333187	+	Y	Y	Y	Y	Y	N	del_ins_uksr	na	NHEJ	intergenic	RNU6-1325P,AC09506	+,-	snRNA,miR	630221;199561	intergenic	RNU6-1325P,AC095061.	+,-	snRNA,miR	633302;196480
925	chr4	65719085	+	chr4	65719550	+	N	Y	Y	Y	Y	N	del_ins_uksr	na	FoSTeS	intergenic	RP11-158O16.1,RP11-	-,-	pseudogene	85470;60914	intergenic	RP11-158O16.1,RP11-70	-,-	pseudogene	85935;60449
926	chr4	67122068	+	chr4	67123492	+	Y	Y	N	Y	N	Y	del_ins_uksr	na	NHEJ	intergenic	RP11-25H12.1,MIR126	+,+	lincRNA,miF	106338;20474	intergenic	RP11-25H12.1,MIR1269/	+,+	lincRNA,miF	107762;19050
927	chr4	104894046	+	chr4	104894681	+	Y	Y	Y	Y	Y	Y	del_ins_uksr	na	NHEJ	ncRNA_intr	RP11-703G6.1	+	lincRNA	0	ncRNA_intr	RP11-703G6.1	+	lincRNA	0
928	chr4	107438051	+	chr4	107438479	+	N	N	Y	N	N	N	del_ins_uksr	na	FoSTeS	intergenic	RP13-612N21.1,ACTR	+,-	lincRNA,pse	63446;319604	intergenic	RP13-612N21.1,ACTR6F	+,-	lincRNA,pse	63874;319176
929	chr4	107438215	+	chr4	107438626	+	Y	N	N	N	N	N	del_ins_uksr	na	FoSTeS	intergenic	RP13-612N21.1,ACTR	+,-	lincRNA,pse	63610;319440	intergenic	RP13-612N21.1,ACTR6F	+,-	lincRNA,pse	64021;319029
930	chr4	120600265	+	chr4	120606568	+	N	Y	N	Y	Y	Y	del_ins_uksr	na	NHEJ	intergenic	PDE5A,RP11-236P13.	-,-	protein_cod	50119;119979	intergenic	PDE5A,RP11-236P13.1	-,-	protein_cod	56422;113676
931	chr4	134132542	+	chr4	134132765	+	N	N	N	N	Y	N	del_ins_uksr	na	FoSTeS	intergenic	PCDH10,AC105252.1	+,-	protein_cod	16777;349469	intergenic	PCDH10,AC105252.1	+,-	protein_cod	17000;349246
932	chr4	135433002	+	chr4	135435702	+	Y	Y	Y	Y	Y	N	del_ins_uksr	na	NHEJ	intergenic	RP11-400D2.3,CTD-20	+,+	lincRNA,linc	53995;247552	intergenic	RP11-400D2.3,CTD-2012	+,+	lincRNA,linc	56695;244852
933	chr4	152252541	+	chr4	152254130	+	N	Y	Y	Y	N	N	del_ins_uksr	na	NHEJ	intergenic	RNU6-1282P,RP11-73	+,-	snRNA,lincF	30776;2386	downstream	RP11-731D1.4	-	lincRNA	0
934	chr4	172988639	+	chr4	172992932	+	Y	Y	Y	Y	N	N	del_ins_uksr	na	NHEJ	intronic	GALNTL6	+	protein_cod	0	intronic	GALNTL6	+	protein_cod	0
935	chr4	173425032	+	chr4	173433521	+	Y	N	N	Y	Y	Y	del_ins_uksr	na	NHEJ	intronic	GALNTL6	+	protein_cod	0	intronic	GALNTL6	+	protein_cod	0
936	chr4	189445668	+	chr4	189447690	+	Y	Y	Y	N	Y	Y	del_ins_uksr	na	NHEJ	ncRNA_intr	LINC01060	+	lincRNA	0	ncRNA_intr	LINC01060	+	lincRNA	0
937	chr5	1061179	+	chr5	1061639	+	N	N	Y	N	N	N	del_ins_uksr	na	FoSTeS	intronic	SLC12A7	-	protein_cod	0	intronic	SLC12A7	-	protein_cod	0
938	chr5	2226443	+	chr5	2226966	+	Y	N	N	N	N	N	del_ins_uksr	na	NHEJ	intergenic	Y_RNA,RP11-129I19.2	+,-	misc_RNA,l	41509;510333	intergenic	Y_RNA,RP11-129I19.2	+,-	misc_RNA,l	42032;509810
939	chr5	6468227	+	chr5	6477094	+	Y	Y	Y	Y	Y	Y	del_ins_uksr	na	NHEJ	intronic	UBE2QL1	+	protein_cod	0	intronic	UBE2QL1	+	protein_cod	0
940	chr5	8694866	+	chr5	8696718	+	N	N	N	Y	N	Y	del_ins_uksr	na	NHEJ	intergenic	RP11-417J1.3,RP11-3	+,-	pseudogene	74444;90288	intergenic	RP11-417J1.3,RP11-315	+,-	pseudogene	76296;88436
941	chr5	16870015	+	chr5	16870736	+	Y	N	N	N	N	N	del_ins_uksr	na	FoSTeS	intronic	MYO10	-	protein_cod	0	intronic	MYO10	-	protein_cod	0
942	chr5	45527116	+	chr5	45527606	+	N	N	N	N	Y	N	del_ins_uksr	na	FoSTeS	intronic	HCN1	-	protein_cod	0	intronic	HCN1	-	protein_cod	0
943	chr5	99400983	+	chr5	99714162	+	N	Y	N	N	N	N	del_ins_uksr	na	FoSTeS	intergenic	RP11-475J5.5,RNU6-1	-,+	pseudogene	10234;88393	intergenic	RNU6-1119P,RP11-346J	+,+	snRNA,lincF	224679;22983
944	chr5	106324751	+	chr5	106326444	+	Y	Y	Y	Y	Y	Y	del_ins_uksr	na	FoSTeS	ncRNA_intr	CTC-254B4.1	-	lincRNA	0	ncRNA_intr	CTC-254B4.1	-	lincRNA	0
945	chr5	119380146	+	chr5	119382681	+	Y	Y	Y	Y	Y	Y	del_ins_uksr	na	NHEJ	intergenic	CTC-507E12.1,CTC-5	+,-	pseudogene	363371;200997	intergenic	CTC-507E12.1,CTC-552	+,-	pseudogene	365906;198462
946	chr5	127336000	+	chr5	127336932	+	N	N	N	Y	N	N	del_ins_uksr	na	NHEJ	ncRNA_intr	CTC-228N24.3	-	lincRNA	0	ncRNA_intr	CTC-228N24.3	-	lincRNA	0
947	chr5	133134678	+	chr5	133136489	+	Y	N	N	N	Y	Y	del_ins_uksr	na	NHEJ	intergenic;ir	AC005178.1,snoU13	-,-	pseudogene	78053;51870	intergenic;ir	AC005178.1,snoU13	-,-	pseudogene	79864;50059
948	chr5	135115881	+	chr5	135120406	+	N	N	N	N	Y	N	del_ins_uksr	na	NHEJ	intergenic	AC011431.1,AC01143	-,-	pseudogene	33836;21037	intergenic	AC011431.1,AC011431.2	-,-	pseudogene	38361;16512
949	chr5	150203160	+	chr5	150223264	+	Y	Y	Y	Y	N	Y	del_ins_uksr	na	NHEJ	intergenic	AC010441.1,IRGM	+,+	protein_cod	26863;22925	intergenic	AC010441.1,IRGM	+,+	protein_cod	46967;2821
950	chr5	156848607	+	chr5	156850882	+	N	Y	Y	N	Y	Y	del_ins_uksr	na	FoSTeS	ncRNA_intr	CTB-109A12.1	-	sense_over	0	ncRNA_intr	CTB-109A12.1	-	sense_over	0
951	chr5	162861773	+	chr5	162863292	+	Y	Y	Y	Y	Y	Y	del_ins_uksr	na	FoSTeS	ncRNA_intr	RP11-541P9.3	-	antisense	0	ncRNA_intr	RP11-541P9.3	-	antisense	0
952	chr6	14745281	+	chr6	14745698	+	Y	Y	Y	Y	Y	Y	del_ins_uksr	na	FoSTeS	intergenic	RNU6-793P,RP11-146	+,-	snRNA,lincF	98412;231738	intergenic	RNU6-793P,RP11-146I2.	+,-	snRNA,lincF	98829;231321
953	chr6	47091639	+	chr6	47091992	+	N	N	N	Y	N	N	del_ins_uksr	na	FoSTeS	intergenic	GPR110,TNFRSF21	-,-	protein_cod	81540;107629	intergenic	GPR110,TNFRSF21	-,-	protein_cod	81893;107276
954	chr6	51847696	+	chr6	101194032	+	Y	Y	N	Y	Y	N	del_ins_uksr	na	NHEJ	intronic	PKHD1	-	protein_cod	0	intronic	ASCC3	-	protein_cod	0
955	chr6	57284911	+	chr6	57289357	+	Y	Y	Y	Y	Y	Y	del_ins_uksr	na	NHEJ	intronic	PRIM2	+	protein_cod	0	intronic	PRIM2	+	protein_cod	0
956	chr6	57533072	+	chr6	57533837	+	N	N	N	N	N	Y	del_ins_uksr	na	VNTR	intergenic	PRIM2,GAPDHP41	+,-	protein_cod	19697;153897	intergenic	PRIM2,GAPDHP41	+,-	protein_cod	20462;153132
957	chr6	63597634	+	chr6	63598986	+	Y	Y	Y	Y	Y	Y	del_ins_uksr	na	NHEJ	intergenic	AL121931.1,AL590558	-,-	miRNA,miR	160805;95355	intergenic	AL121931.1,AL590558.1	-,-	miRNA,miR	162157;94003
958	chr6	67040800	+	chr6	67043190	+	Y	Y	Y	Y	Y	Y	del_ins_uksr	na	FoSTeS	intergenic	NUFIP1P,AC002485.1	-,+	pseudogene	235998;28518	intergenic	NUFIP1P,AC002485.1	-,+	pseudogene	238388;26128
959	chr6	73221940	+	chr6	73222766	+	N	Y	Y	N	Y	Y	del_ins_uksr	na	FoSTeS	intergenic	RIMS1,RP11-135M8__	+,+	protein_cod	109095;10403	intergenic	RIMS1,RP11-135M8__A.	+,+	protein_cod	109921;9577
960	chr6	74932722	+	chr6	74933074	+	N	Y	Y	N	Y	N	del_ins_uksr	na	NHEJ	ncRNA_intr	RP11-554D15.1	+	lincRNA	0	ncRNA_intr	RP11-554D15.1	+	lincRNA	0
961	chr6	86623833	+	chr6	86625833	+	Y	Y	Y	Y	Y	Y	del_ins_uksr	na	NHEJ	intergenic	RP11-207F8.1,RNU4-1	+,-	pseudogene	44651;78906	intergenic	RP11-207F8.1,RNU4-12F	+,-	pseudogene	46651;76906
962	chr6	96853032	+	chr6	96853180	+	N	N	N	N	N	Y	del_ins_uksr	na	FoSTeS	ncRNA_intr	UFL1-AS1	-	antisense	0	ncRNA_intr	UFL1-AS1	-	antisense	0
963	chr6	98416730	+	chr6	98418538	+	Y	Y	Y	Y	Y	Y	del_ins_uksr	na	NHEJ	ncRNA_intr	RP11-436D23.1	+	lincRNA	0	ncRNA_intr	RP11-436D23.1	+	lincRNA	0
964	chr6	100034501	+	chr6	100035322	+	Y	Y	Y	Y	Y	Y	del_ins_uksr	na	NHEJ	intergenic	RP1-199J3.5,PRDM13	+,+	pseudogene	10169;20105	intergenic	RP1-199J3.5,PRDM13	+,+	pseudogene	10990;19284
965	chr6	103737464	+	chr6	103762890	+	N	Y	N	Y	Y	Y	del_ins_uksr	na	NHEJ	intergenic	RP1-76C18.1,SNORA3	+,+	pseudogene	285692;293546	intergenic	RP1-76C18.1,SNORA33	+,+	pseudogene	311118;268120
966	chr6	108031342	+	chr6	108032403	+	Y	Y	Y	Y	Y	Y	del_ins_uksr	na	FoSTeS	intronic	SCML4	-	protein_cod	0	intronic	SCML4	-	protein_cod	0
967	chr6	153747364	+	chr6	153748554	+	N	N	Y	N	Y	Y	del_ins_uksr	na	NHEJ	intergenic;ir	RNA5SP225,RP11-33	+,+	rRNA,pseuc	5657;239478	intergenic;ir	RNA5SP225,RP11-331O	+,+	rRNA,pseuc	6847;238288
968	chr6	160877913	+	chr6	160956049	+	N	N	N	N	Y	N	del_ins_uksr	na	FoSTeS	ncRNA_intr	LPAL2	-	pseudogene	0	intronic	LPA	-	protein_cod	0
969	chr6	167488070	+	chr6	167489577	+	N	Y	Y	Y	Y	Y	del_ins_uksr	na	NHEJ	ncRNA_intr	RP11-517H2.6	+	processed_	0	ncRNA_intr	RP11-517H2.6	+	processed_	0
970	chr7	151134	+	chr7	160558	+	Y	Y	Y	Y	N	Y	del_ins_uksr	na	NHEJ	ncRNA_exo	AC093627.10	+	lincRNA	0	intergenic	AC093627.10,RP11-90P	+,-	lincRNA,linc	5093;1207
971	chr7	151958	+	chr7	160572	+	N	N	N	N	Y	N	del_ins_uksr	na	FoSTeS	ncRNA_exo	AC093627.10	+	lincRNA	0	intergenic	AC093627.10,RP11-90P	+,-	lincRNA,linc	5107;1193
972	chr7	605922	+	chr7	606305	+	N	N	N	N	N	Y	del_ins_uksr	na	FoSTeS	intronic	PRKAR1B	-	protein_cod	0	intronic	PRKAR1B	-	protein_cod	0
973	chr7	929280	+	chr7	929611	+	N	N	N	N	Y	N	del_ins_uksr	na	FoSTeS	intronic	SUN1,GET4	+,+	protein_cod	0	intronic	SUN1,GET4	+,+	protein_cod	0
974	chr7	24258087	+	chr7	24258484	+	Y	Y	Y	N	Y	Y	del_ins_uksr	na	NHEJ	ncRNA_intr	AC004485.3	-	antisense	0	ncRNA_intr	AC004485.3	-	antisense	0
975	chr7	35984737	+	chr7	62472988	+	N	Y	Y	N	N	N	del_ins_uksr	na	NHEJ	intergenic	RNU6-1085P,SEPT7P	-,+	snRNA,pseu	14863;1167	intergenic	RNU6-417P,RP11-196D1	-,-	snRNA,lincF	1041;32217
976	chr7	51594312	+	chr7	51598690	+	Y	Y	Y	Y	Y	Y	del_ins_uksr	na	NHEJ	intergenic	CTD-2021A8.3,ROBO	-,-	pseudogene	137501;73670	intergenic	CTD-2021A8.3,ROBO2P	-,-	pseudogene	141879;69292
977	chr7	52171023	+	chr7	52397345	+	Y	N	N	N	N	N	del_ins_uksr	na	FoSTeS	intergenic	RN7SL292P,RP11-153	+,-	misc_RNA,l	386408;61908	intergenic	snoU13,RP11-398K14.1	-,-	snoRNA,ps	5760;562185
978	chr7	52171297	+	chr7	52397627	+	N	N	N	N	Y	N	del_ins_uksr	na	FoSTeS	intergenic	RN7SL292P,RP11-153	+,-	misc_RNA,l	386682;61634	intergenic	snoU13,RP11-398K14.1	-,-	snoRNA,ps	6042;561903
979	chr7	53267356	+	chr7	53267717	+	Y	N	Y	Y	N	N	del_ins_uksr	na	NHEJ	intergenic	HAUS6P1,RP11-476O	+,+	pseudogene	10725;116486	intergenic	HAUS6P1,RP11-476O21	+,+	pseudogene	11086;116125
980	chr7	56403796	+	chr7	100908125	+	Y	N	N	N	N	N	del_ins_uksr	na	FoSTeS	intergenic	RP11-814E24.1,RNU6	+,-	pseudogene	12502;4128	intergenic	FIS1,RNU6-1104P	-,-	protein_cod	12528;5094
981	chr7	61525507	+	chr7	61526375	+	Y	Y	N	Y	Y	N	del_ins_uksr	na	NHEJ	intergenic	NONE,RP11-715L17.1	NONE,+	NONE,pseu	NONE;296362	intergenic	NONE,RP11-715L17.1	NONE,+	NONE,pseu	NONE;295494
982	chr7	62366049	+	chr7	62369710	+	Y	N	N	Y	Y	Y	del_ins_uksr	na	VNTR	intergenic	RP11-715L17.1,RNU6-	+,-	pseudogene	543863;105792	intergenic	RP11-715L17.1,RNU6-41	+,-	pseudogene	547524;102131
983	chr7	67131795	+	chr7	67132146	+	N	Y	Y	Y	Y	Y	del_ins_uksr	na	FoSTeS	intergenic	RP4-736H5.2,RP4-736	+,-	pseudogene	38459;24250	intergenic	RP4-736H5.2,RP4-736H	+,-	pseudogene	38810;23899
984	chr7	91031083	+	chr7	91042585	+	Y	N	N	Y	N	Y	del_ins_uksr	na	NHEJ	ncRNA_intr	RP11-142A5.1,RP11-1	-,+	lincRNA,linc	0	ncRNA_intr	RP11-142A5.1,RP11-115	-,+	lincRNA,linc	0
985	chr7	93541816	+	chr7	93542540	+	Y	Y	Y	Y	Y	Y	del_ins_uksr	na	NHEJ	intergenic	GNGT1,GNG11	+,+	protein_cod	1239;9195	intergenic	GNGT1,GNG11	+,+	protein_cod	1963;8471
986	chr7	97496333	+	chr7	97496666	+	N	Y	Y	Y	Y	Y	del_ins_uksr	na	NHEJ	intronic	ASNS	-	protein_cod	0	intronic	ASNS	-	protein_cod	0
987	chr7	100550585	+	chr7	100551240	+	N	N	N	N	Y	N	del_ins_uksr	na	FoSTeS	upstream;d	MUC3A,MUC3A	+,+	protein_cod	0	upstream;d	MUC3A,MUC3A	+,+	protein_cod	0
988	chr7	100551112	+	chr7	100551336	+	N	N	N	Y	N	N	del_ins_uksr	na	FoSTeS	upstream;d	MUC3A,MUC3A	+,+	protein_cod	0	exonic	MUC3A	+	protein_cod	0
989	chr7	118831248	+	chr7	118834755	+	Y	Y	Y	Y	Y	Y	del_ins_uksr	na	FoSTeS	intergenic	RP11-500M10.1,AC09	-,-	pseudogene	12045;293131	intergenic	RP11-500M10.1,AC0913	-,-	pseudogene	15552;289624
990	chr7	137995515	+	chr7	137996419	+	Y	Y	Y	Y	Y	Y	del_ins_uksr	na	NHEJ	intergenic	AC008155.2,AC00815	-,-	pseudogene	12286;93425	intergenic	AC008155.2,AC008155.1	-,-	pseudogene	13190;92521
991	chr7	142041709	+	chr7	142045948	+	Y	Y	Y	Y	N	Y	del_ins_uksr	na	FoSTeS	intergenic	TRBV7-1,TRBV4-2	+,+	TR_V_gene	9182;3543	downstream	TRBV4-2	+	TR_V_gene	0
992	chr7	142099580	+	chr7	142247245	+	N	N	N	Y	Y	N	del_ins_uksr	na	NHEJ	exonic;exon	TRBV7-8	-	TR_V_gene	0	exonic;exon	TRBV7-3	-	TR_V_gene	0
993	chr7	142360546	+	chr7	142362014	+	Y	Y	Y	Y	Y	Y	del_ins_uksr	na	FoSTeS	intergenic	TRBV23-1,TRBV24-1	+,+	TR_V_gene	6582;3652	intergenic	TRBV23-1,TRBV24-1	+,+	TR_V_gene	8050;2184
994	chr7	149736246	+	chr7	149739272	+	N	Y	N	N	N	N	del_ins_uksr	na	FoSTeS	intergenic	RP11-728K20.2,RP11-	+,+	lincRNA,linc	34033;1364	ncRNA_intr	RP11-545G3.1	+	lincRNA	0
995	chr8	594397	+	chr8	599415	+	Y	Y	Y	Y	Y	Y	del_ins_uksr	na	NHEJ	intronic	ERICH1	-	protein_cod	0	intronic	ERICH1	-	protein_cod	0
996	chr8	1837298	+	chr8	1837427	+	Y	N	N	N	N	N	del_ins_uksr	na	FoSTeS	intronic	ARHGEF10	+	protein_cod	0	intronic	ARHGEF10	+	protein_cod	0
997	chr8	2251669	+	chr8	2267087	+	N	N	N	Y	N	N	del_ins_uksr	na	FoSTeS	intergenic	NONE,AC133633.2	NONE,-	NONE,lincR	NONE;99323	intergenic	NONE,AC133633.2	NONE,-	NONE,lincR	NONE;83905
998	chr8	12427810	+	chr8	12432836	+	N	Y	N	N	Y	N	del_ins_uksr	na	FoSTeS	downstream	RPS3AP34	-	pseudogene	0	intergenic	RPS3AP34,AC068587.2	-,+	pseudogene	4197;2678
999	chr8	16201310	+	chr8	16207534	+	Y	Y	Y	Y	N	Y	del_ins_uksr	na	NHEJ	intronic	MSR1	-	protein_cod	0	intronic	MSR1	-	protein_cod	0
1000	chr8	60248648	+	chr8	60249098	+	Y	N	N	N	N	N	del_ins_uksr	na	NHEJ	intergenic	SNORA51,RNA5SP26	-,+	snoRNA,rRI	198587;119796	intergenic	SNORA51,RNA5SP267	-,+	snoRNA,rRI	199037;119346
1001	chr8	75362871	+	chr8	75366963	+	Y	N	Y	Y	N	N	del_ins_uksr	na	NHEJ	intergenic;ir	Y_RNA,MIR5681A	+,+	misc_RNA,r	73843;97907	intergenic	Y_RNA,MIR5681A	+,+	misc_RNA,r	77935;93815
1002	chr8	75362871	+	chr8	75367000	+	N	Y	N	N	Y	Y	del_ins_uksr	na	NHEJ	intergenic;ir	Y_RNA,MIR5681A	+,+	misc_RNA,r	73843;97907	intergenic	Y_RNA,MIR5681A	+,+	misc_RNA,r	77972;93778

1003	chr8	83005233	+	chr8	83005683	+	Y	Y	Y	Y	Y	Y	del_ins_uksr	na	NHEJ	intergenic	RP11-354A14.1,RP11-	+,-	lincRNA,linc	169805;5453	intergenic	RP11-354A14.1,RP11-99	+,-	lincRNA,linc	170255;5003
1004	chr8	127192205	+	chr8	127194572	+	Y	Y	Y	Y	Y	N	del_ins_uksr	na	NHEJ	intergenic	RFPL4AP5,RP11-65D1	-,+	pseudogene	4947;145535	intergenic	RFPL4AP5,RP11-65D17.	-,+	pseudogene	7314;143168
1005	chr8	131850749	+	chr8	131852739	+	Y	Y	Y	Y	Y	Y	del_ins_uksr	na	NHEJ	intronic	ADCY8	-	protein_cod	0	intronic	ADCY8	-	protein_cod	0
1006	chr8	146057716	+	chr8	146057957	+	N	N	N	N	N	Y	del_ins_uksr	na	NHEJ	intronic	ZNF7	+	protein_cod	0	intronic	ZNF7	+	protein_cod	0
1007	chr8	146057826	+	chr8	146057959	+	N	N	N	Y	N	N	del_ins_uksr	na	NHEJ	intronic	ZNF7	+	protein_cod	0	intronic	ZNF7	+	protein_cod	0
1008	chr9	12345338	+	chr9	12346062	+	Y	Y	Y	Y	N	Y	del_ins_uksr	na	NHEJ	intergenic	RNU2-47P,TYRP1	-,+	snRNA,prot	44887;340101	intergenic	RNU2-47P,TYRP1	-,+	snRNA,prot	45611;339377
1009	chr9	23362801	+	chr9	23377686	+	Y	Y	Y	Y	Y	Y	del_ins_uksr	na	NHEJ	intergenic	AL391117.1,SUMO2P2	-,+	miRNA,pseu	409699;269303	intergenic	AL391117.1,SUMO2P2	-,+	miRNA,pseu	424584;254418
1010	chr9	28047359	+	chr9	28051256	+	Y	N	Y	Y	Y	Y	del_ins_uksr	na	FoSTeS	intronic;intro	LINGO2	-	protein_cod	0	intronic;intro	LINGO2	-	protein_cod	0
1011	chr9	66532439	+	chr9	66533325	+	Y	Y	Y	Y	Y	Y	del_ins_uksr	na	FoSTeS	ncRNA_intr	RP11-262H14.3	-	lincRNA	0	ncRNA_intr	RP11-262H14.3	-	lincRNA	0
1012	chr9	69786670	+	chr9	69787039	+	N	Y	N	N	N	N	del_ins_uksr	na	FoSTeS	intergenic	IGKV1OR-2,AL359955	-,-	IG_V_pseu	8717;35090	intergenic	IGKV1OR-2,AL359955.1	-,-	IG_V_pseu	9086;34721
1013	chr9	114333291	+	chr9	114333884	+	Y	Y	Y	Y	N	Y	del_ins_uksr	na	FoSTeS	intronic	PTGR1,ZNF483	-,+	protein_cod	0	intronic	PTGR1,ZNF483	-,+	protein_cod	0
1014	chr9	136625194	+	chr9	136626268	+	Y	Y	Y	Y	Y	Y	del_ins_uksr	na	NHEJ	intergenic	SARDH,VAV2	-,-	protein_cod	20117;1822	downstream	VAV2,AL590710.1	-,-	protein_cod	0
1015	chr9	139427524	+	chr9	139427796	+	Y	Y	Y	N	Y	Y	del_ins_uksr	na	NHEJ	intronic	NOTCH1	-	protein_cod	0	intronic	NOTCH1	-	protein_cod	0
1016	chrX	38386504	+	chrX	38387332	+	Y	Y	Y	Y	Y	Y	del_ins_uksr	na	NHEJ	intronic	TM4SF2	+	protein_cod	0	intronic	TM4SF2	+	protein_cod	0
1017	chrX	122972807	+	chrX	122973266	+	N	N	Y	N	N	N	del_ins_uksr	na	FoSTeS	intergenic	CHCHD2P1,Y_RNA	+,-	pseudogene	53506;18105	intergenic	CHCHD2P1,Y_RNA	+,-	pseudogene	53965;17646
1018	chrY	13674175	+	chrY	13798170	+	N	N	Y	Y	N	N	del_ins_uksr	na	FoSTeS	intergenic	RP11-295P22.2,RCC2	-,-	pseudogene	44262;227583	intergenic	RP11-295P22.2,RCC2P1	-,-	pseudogene	168257;103588
1019	chrY	28804466	+	chrY	28812570	+	N	Y	N	N	N	N	del_ins_uksr	na	FoSTeS	intergenic	PARP4P1,NONE	-,NONE	pseudogene	23667;NONE	intergenic	PARP4P1,NONE	-,NONE	pseudogene	31771;NONE
1020	chr1	226415473	-	chr11	59283672	+	N	N	N	Y	N	N	del_inso	d	FoSTeS	downstream	MIXL1	+	protein_cod	0	downstream	OR4D9	+	protein_cod	0
1021	chr1	3754993	+	chr11	119124249	-	Y	N	N	N	N	N	del_inso	u	FoSTeS	intronic;intro	CEP104	-	protein_cod	0	intronic;intro	CBL	+	protein_cod	0
1022	chr1	247685228	+	chr11	68243429	-	N	N	Y	N	N	N	del_inso	u	FoSTeS	intronic	GCSAML	+	protein_cod	0	intronic	PPP6R3	+	protein_cod	0
1023	chr1	46219074	-	chr12	11236776	+	N	N	N	N	Y	N	del_inso	d	l_LTR_LTR1(	intergenic	IPP,RP11-630I5.1	-,+	protein_cod	2752;27875	intergenic	TAS2R64P,TAS2R43	-,-	pseudogene	5006;7110
1024	chr1	46219185	-	chr12	11236655	+	Y	N	Y	Y	N	N	del_inso	d	FoSTeS	intergenic	IPP,RP11-630I5.1	-,+	protein_cod	2863;27764	intergenic	TAS2R64P,TAS2R43	-,-	pseudogene	4885;7231
1025	chr1	46219230	-	chr12	11236779	+	N	Y	N	N	N	N	del_inso	d	FoSTeS	intergenic	IPP,RP11-630I5.1	-,+	protein_cod	2908;27719	intergenic	TAS2R64P,TAS2R43	-,-	pseudogene	5009;7107
1026	chr1	12909078	-	chr15	83428548	+	N	N	Y	N	N	N	del_inso	d	FoSTeS	upstream	HNRNPCL1	-	protein_cod	0	intronic	FSD2	-	protein_cod	0
1027	chr1	17230525	+	chr15	75867613	-	Y	N	N	Y	Y	Y	del_inso	u	FoSTeS	downstream	RP11-108M9.6	-	lincRNA	0	ncRNA_intr	CTD-2323K18.1	-	processed_	0
1028	chr1	53890873	-	chr17	74607011	+	N	N	N	N	Y	N	del_inso	d	FoSTeS	ncRNA_intr	SLC25A3P1	-	pseudogene	0	intergenic	RP11-666A8.11,ST6GAL	-,-	pseudogene	15157;13832
1029	chr1	185025679	-	chr19	45828385	+	N	Y	N	Y	N	N	del_inso	d	FoSTeS	intronic;intro	RNF2	+	protein_cod	0	intergenic;ir	CKM,RPS16P9	-,-	protein_cod	2150;7571
1030	chr1	144042276	-	chr4	142757071	+	N	N	N	N	Y	N	del_inso	d	FoSTeS	ncRNA_intr	SRGAP2B	+	pseudogene	0	intergenic	IL15,INPP4B	+,-	protein_cod	101931;187242
1031	chr1	121214742	+	chr5	49771514	-	N	N	Y	N	N	N	del_inso	u	FoSTeS	intergenic	AL592494.5,RP11-344	+,-	lincRNA,linc	9759;21435	intergenic	EMB,RP11-269M20.2	-,+	protein_cod	34313;127549
1032	chr1	121214967	+	chr5	49771194	-	N	N	N	Y	N	N	del_inso	u	FoSTeS	intergenic;ir	AL592494.5,RP11-344	+,-	lincRNA,linc	9984;21210	intergenic;ir	EMB,RP11-269M20.2	-,+	protein_cod	33993;127869
1033	chr1	4827759	+	chr8	142837414	-	N	N	N	N	N	Y	del_inso	u	FoSTeS	intronic;intro	AJAP1	+	protein_cod	0	intergenic;ir	AC138647.1,AC104417.1	+,+	protein_cod	308577;189446
1034	chr1	142542550	-	chrY	13447038	+	N	N	N	N	N	Y	del_inso	d	FoSTeS	intergenic	NONE,RP11-417J8.1	NONE,+	NONE,lincR	NONE;10743	intergenic	AC134878.1,DUX4L16	-,+	miRNA,pseu	106405;15556
1035	chr10	84855768	+	chr2	241602862	-	N	N	N	N	N	Y	del_inso	u	EA_LTR_LTI	intergenic;ir	RNU6-478P,MARK2P1	-,-	snRNA,pseu	27941;215616	intergenic;ir	GPR35,AQP12B	+,-	protein_cod	32186;12973
1036	chr10	85222525	-	chr4	4350755	+	Y	N	N	N	N	N	del_inso	d	FoSTeS	intergenic;ir	AL356140.1,RP11-344	-,-	miRNA,lincF	25640;209637	intronic;intro	NSG1	+	protein_cod	0
1037	chr11	68243301	-	chr1	247685665	+	N	N	Y	N	N	N	del_inso	d	FoSTeS	intronic	PPP6R3	+	protein_cod	0	intronic	GCSAML	+	protein_cod	0
1038	chr11	20255079	+	chr1	207991569	-	N	N	N	N	Y	N	del_inso	u	FoSTeS	intergenic	DBX1,HTATIP2	-,+	protein_cod	72920;130152	ncRNA_intr	C1orf132	-	processed_	0
1039	chr11	83126620	+	chr13	91319636	-	N	N	N	N	Y	N	del_inso	u	FoSTeS	ncRNA_intr	RP11-727A23.10	+	antisense	0	intergenic	LINC01049,RNU6-75P	+,-	lincRNA,snF	132302;114384
1040	chr11	83126767	+	chr13	91319608	-	N	N	Y	N	N	N	del_inso	u	FoSTeS	ncRNA_intr	RP11-727A23.10	+	antisense	0	intergenic	LINC01049,RNU6-75P	+,-	lincRNA,snF	132274;114412
1041	chr11	14863243	+	chr14	56319805	-	N	N	Y	Y	N	N	del_inso	u	FoSTeS	intronic;intro	PDE3B	+	protein_cod	0	intergenic;ir	LINC00520,RP11-1012E1	-,+	processed_	56399;62640
1042	chr12	11236550	+	chr1	46219466	-	N	N	N	Y	N	N	del_inso	u	FoSTeS	intergenic	TAS2R64P,TAS2R43	-,-	pseudogene	4780;7336	intergenic	IPP,RP11-630I5.1	-,+	protein_cod	3144;27483
1043	chr12	11236583	+	chr1	46219433	-	Y	Y	Y	N	Y	N	del_inso	u	l_LTR_LTR1(	intergenic	TAS2R64P,TAS2R43	-,-	pseudogene	4813;7303	intergenic	IPP,RP11-630I5.1	-,+	protein_cod	3111;27516
1044	chr12	38592828	-	chr4	190908641	+	Y	N	N	N	N	N	del_inso	d	FoSTeS	intergenic	RNA5SP359,TUBB8P5	-,+	rRNA,pseuc	35511;2540	intergenic	TUBB7P,RNA5SP174	-,+	pseudogene	2615;27652
1045	chr12	19940304	+	chr6	131013597	-	N	N	N	N	N	Y	del_inso	u	FoSTeS	ncRNA_intr	RP11-405A12.2	+	lincRNA	0	intergenic;ir	Y_RNA,RP11-102N11.1	-,+	misc_RNA,l	118340;4860
1046	chr12	43292131	-	chr7	64883534	+	N	N	N	Y	N	N	del_inso	d	FoSTeS	intergenic	RP11-25I15.3,RP11-51	+,-	lincRNA,pse	181478;31520	intergenic	ZNF92,RP11-667F9.2	+,+	protein_cod	17496;44450
1047	chr13	91319330	-	chr11	83126980	+	N	N	Y	N	N	N	del_inso	d	FoSTeS	intergenic	LINC01049,RNU6-75P	+,-	lincRNA,snF	131996;114690	ncRNA_intr	RP11-727A23.10	+	antisense	0
1048	chr13	91319395	-	chr11	83126884	+	N	N	N	N	Y	N	del_inso	d	FoSTeS	intergenic	LINC01049,RNU6-75P	+,-	lincRNA,snF	132061;114625	ncRNA_intr	RP11-727A23.10	+	antisense	0
1049	chr13	60825361	+	chr2	90418228	-	N	Y	N	N	N	N	del_inso	u	FoSTeS	intergenic	LINC00434,TARDBPP	-,+	lincRNA,pse	36281;23549	intergenic	AC113612.1,CH17-132F2	+,+	pseudogene	117826;39973
1050	chr13	67020063	-	chr5	135829403	+	N	Y	N	N	N	N	del_inso	d	FoSTeS	intronic	PCDH9	-	protein_cod	0	ncRNA_intr	RP11-114H21.2	+	lincRNA	0
1051	chr14	49640022	-	chr11	25244525	+	N	N	Y	Y	N	N	del_inso	d	FoSTeS	intergenic	RP11-816J8.1,RP11-3	-,+	lincRNA,pse	95799;200662	intergenic	RP11-54J7.2,AC015820.	-,+	pseudogene	81956;314299
1052	chr14	37769602	+	chr16	61079285	-	Y	N	N	N	N	N	del_inso	u	FoSTeS	intronic;intro	MIPOL1	+	protein_cod	0	intergenic;ir	RP11-90K18.1,RP11-51C	-,-	pseudogene	116840;10018
1053	chr15	75867352	-	chr1	17230809	+	Y	N	N	Y	N	Y	del_inso	d	EA_SINE_SIN	ncRNA_intr	CTD-2323K18.1	-	processed_	0	downstream	RP11-108M9.6	-	lincRNA	0
1054	chr15	75867613	+	chr1	17230525	-	N	N	Y	N	N	N	del_inso	u	FoSTeS	ncRNA_intr	CTD-2323K18.1	-	processed_	0	downstream	RP11-108M9.6	-	lincRNA	0
1055	chr15	20452654	-	chr16	33406197	+	N	Y	N	N	N	N	del_inso	d	FoSTeS	ncRNA_intr	RP11-492D6.3	-	pseudogene	0	ncRNA_intr	RP11-293B20.2	+	pseudogene	0
1056	chr15	100587454	+	chr6	10927915	-	N	N	Y	N	Y	N	del_inso	u	FoSTeS	intronic	ADAMTS17	-	protein_cod	0	intronic	SYCP2L,RP11-637O19.3	+,+	protein_cod	0
1057	chr15	36380904	-	chr9	99002215	+	N	N	N	N	N	Y	del_inso	d	FoSTeS	ncRNA_intr	RP11-184D12.1	-	lincRNA	0	intronic;intro	HSD17B3	-	protein_cod	0
1058	chr16	34978716	-	chr14	93227423	+	Y	N	N	N	N	N	del_inso	d	FoSTeS	ncRNA_intr	5S_rRNA	+	lincRNA	0	intergenic	LGMN,GOLGA5	-,+	protein_cod	12376;33153
1059	chr16	61079285	+	chr14	37769602	-	N	N	N	N	N	Y	del_inso	u	FoSTeS	intergenic	RP11-90K18.1,RP11-5	-,-	pseudogene	116840;10018	intronic	MIPOL1	+	protein_cod	0
1060	chr16	21835151	+	chr15	22412531	-	N	Y	N	N	N	N	del_inso	u	FoSTeS	intergenic	RRN3P1,NPIPB4	-,-	pseudogene	3420;10739	ncRNA_intr	RP11-69H14.6	+	sense_over	0
1061	chr16	33853339	+	chr19	33444599	-	N	N	N	N	N	Y	del_inso	u	FoSTeS	ncRNA_intr	RP11-598D12.2	+	pseudogene	0	exonic;exon	CEP89	-	protein_cod	0
1062	chr16	33962482	-	chr2	133013782	+	N	Y	N	N	N	N	del_inso	d	FoSTeS	ncRNA_exo	LINC00273	-	lincRNA	0	downstream	MIR663B	-	miRNA	0
1063	chr16	46460360	-	chr2	92263985	+	N	Y	N	N	N	N	del_inso	d	FoSTeS	intergenic	NONE,ANKRD26P1	NONE,-	NONE,pseu	NONE;42893	intergenic	IGKV1OR2-2,NONE	-,NONE	pseudogene	40959;NONE
1064	chr16	33953561	+	chr2	133029808	-	Y	N	N	N	N	N	del_inso	u	FoSTeS	intergenic	AC136932.2,LINC0027	-,-	miRNA,lincF	7540;7491	intergenic	CDC27P1,AC097532.2	+,-	pseudogene	9193;13560
1065	chr17	74606989	+	chr1	53891025	-	N	N	N	N	Y	N	del_inso	u	FoSTeS	intergenic	RP11-666A8.11,ST6G/	-,-	pseudogene	15135;13854	ncRNA_intr	SLC25A3P1	-	pseudogene	0
1066	chr17	19156814	+	chr14	74928715	-	N	N	Y	N	N	N	del_inso	u	FoSTeS	intronic	EPN2	+	protein_cod	0	intergenic	SYNDIG1L,RP3-449M8.9	-,-	protein_cod	35910;9918
1067	chr17	19995620	+	chr2	209033721	-	N	N	N	N	N	Y	del_inso	u	FoSTeS	intronic;intro	SPECC1	+	protein_cod	0	intronic;intro	C2orf80	-	protein_cod	0
1068	chr17	41465773	-	chr3	73159850	+	N	N	N	N	Y	N	del_inso	d	FoSTeS	ncRNA_exo	LINC00910	-	lincRNA	0	upstream;up	RNU2-64P	+	snRNA	0
1069	chr18	55381573	-	chr5	175733409	+	N	N	N	N	Y	N	del_inso	d	FoSTeS	ncRNA_intr	RP11-35G9.3,RP11-35	+,+	antisense,ai	0	intronic;intro	SIMC1	+	protein_cod	0
1070	chr18	11959639	+	chr7	105052259	-	N	Y	N	N	N	N	del_inso	u	FoSTeS	intergenic	RP11-820I16.2,IMPA2	+,+	pseudogene	30164;21397	intergenic	SRPK2,PUS7	-,-	protein_cod	22421;27849
1071	chr18	22222371	-	chr8	11098987	+	N	Y	N	N	N	N	del_inso	d	FoSTeS	ncRNA_intr	RP11-449D8.1	+	lincRNA	0	intergenic;ir	AF131215.8,LINC00529	+,-	lincRNA,linc	37807;6148
1072	chr18	49574	+	chr9	141069873	-	N	Y	N	N	N	N	del_inso	u	FoSTeS	intronic	RP11-683L23.1	-	protein_cod	0	ncRNA_exo	TUBBP5	+	pseudogene	0
1073	chr19	45732602	+	chr1	210891700	-	N	Y	N	N	N	N	del_inso	u	FoSTeS	intronic	MARK4,EXOC3L2	+,-	protein_cod	0	intronic	KCNH1	-	protein_cod	0
1074	chr19	45732707	+	chr1	210892310	-	N	N	N	Y	N	N	del_inso	u	FoSTeS	intronic	MARK4,EXOC3L2	+,-	protein_cod	0	intronic	KCNH1	-	protein_cod	0
1075	chr19	52428125	-	chrY	9962795	+	Y	N	N	N	N	N	del_inso	d	FoSTeS	intergenic	ZNF649,ZNF613	-,+	protein_cod	19832;2348	intergenic	RNA5SP519,AC006987.(	-,-	rRNA,pseuc	32193;44602
1076	chr2	133014882	-	chr16	33961395	+	Y	N	N	N	N	N	del_inso	d	FoSTeS	upstream	MIR663B	-	miRNA	0	ncRNA_exo	LINC00273	-	lincRNA	0
1077	chr2	92261554	+	chr16	46462333	-	N	Y	N	N	N	N	del_inso	u	FoSTeS	intergenic	IGKV1OR2-2,NONE	-,NONE	pseudogene	38528;NONE	intergenic	NONE,ANKRD26P1	NONE,-	NONE,pseu	NONE;40920
1078	chr2	133011830	+	chr16	33964365	-	N	Y	N	N	N	N	del_inso	u	FoSTeS	upstream	RNA5-8SP5	-	rRNA	0	intergenic	LINC00273,RNA5-8SP2	-,+	lincRNA,rRI	1862;1061
1079	chr2	157225132	-	chr17	80759978	+	N	N	N	N	N	Y	del_inso	d	FoSTeS	intergenic	NR4A2,snoU13	-,-	protein_cod	26272;41524	intronic	TBCD	+	protein_cod	0
1080	chr2	237615486	-	chr21	39080027	+	N	N	N	N	Y	N	del_inso	d	FoSTeS	intergenic	ACKR3,AC011286.1	+,+	protein_cod	124485;26539	intronic	KCNJ6	-	protein_cod	0
1081	chr2	14414570	-	chr6	130218879	+	N	N	N	N	Y	N	del_inso	d	FoSTeS	ncRNA_intr	LINC00276	-	lincRNA	0	intergenic;ir	TMEM244,L3MBTL3	-,+	protein_cod	36187;115965
1082	chr2	47319280	+	chrX	153684967	-	Y	N	N	N	N	N	del_inso	u	EI_SINE_Alu	intronic;intro	C2orf61,RP11-761B3.1	-,-	protein_cod	0	intergenic;ir	FAM50A,PLXNA3	+,+	protein_cod	5965;1654
1083	chr2	133015072	-	chrY	10033584	+	N	N	N	N	N	Y	del_inso	d	FoSTeS	upstream	MIR663B	-	miRNA	0	upstream	AC010970.1	+	miRNA	0
1084	chr20	41587368	-	chr10	116764328	+	N	N	N	N	Y	N	del_inso	d	FoSTeS	intronic	PTPRT	-	protein_cod	0	intergenic	RP11-383C6.2,ATRNL1	+,+	lincRNA,pro	7976;88802
1085	chr20	59958290	-	chr10	54593888	+	N	N	N	Y	N	N	del_inso	d	FoSTeS	intronic	CDH4	+	protein_cod	0	intergenic	MBL2,Y_RNA	-,+	protein_cod	62428;18479
1086	chr20	26210879	-	chr12	85010620	+	Y	N	N	N	N	N	del_inso	d	FoSTeS	ncRNA_intr	MIR663A	-	processed_	0	intergenic	RP11-228G3.1,RP11-701	-,+	pseudogene	166963;54757

1087	chr21	39080014	+	chr2	237615782	-	N	N	N	N	Y	N	del_inso	u	FoSTeS	intronic	KCNJ6	-	protein_cod	0	intergenic	ACKR3,AC011286.1	+,+	protein_cod	124781;26243
1088	chr21	11022625	+	chr22	45991810	-	N	N	Y	N	Y	Y	del_inso	u	FoSTeS	ncRNA_intr	BAGE2	-	pseudogene	0	intronic	FBLN1	+	protein_cod	0
1089	chr21	18553575	-	chr8	64238060	+	N	N	N	N	Y	N	del_inso	d	FoSTeS	intergenic;ir	AF212831.2,NEK4P1	-,-	lincRNA,pse	307564;29212	intergenic;ir	RN7SL135P,CTD-3046C	-,-	misc_RNA,l	90591;59337
1090	chr22	28121813	-	chr16	86452837	+	N	N	Y	N	N	N	del_inso	d	FoSTeS	intergenic	RP11-375H17.1,MN1	+,-	antisense,pr	4148;22452	intergenic	LINC00917,RP11-158I3.1	-,-	lincRNA,pse	69548;15599
1091	chr22	45991517	-	chr21	11022748	+	N	Y	Y	N	Y	Y	del_inso	d	FoSTeS	intronic	FBLN1	+	protein_cod	0	ncRNA_intr	BAGE2	-	pseudogene	0
1092	chr22	23852560	-	chr9	132186744	+	N	N	N	N	N	Y	del_inso	d	FoSTeS	intergenic;ir	AP000345.1,AP00034	-,-	lincRNA,linc	23393;2121	intergenic;ir	RP11-65J3.6,RP11-65J3.	-,-	pseudogene	43995;6187
1093	chr3	73160133	+	chr11	62609281	-	Y	N	N	N	N	N	del_inso	u	FoSTeS	upstream	RNU2-64P	+	snRNA	0	ncRNA_UTI	WDR74,RNU2-2P	-,-	protein_cod	0
1094	chr3	46877491	+	chr17	41467211	-	N	N	N	N	Y	N	del_inso	u	FoSTeS	intergenic	PRSS42,MYL3	-,-	protein_cod	1906;21871	upstream	LINC00910	-	lincRNA	0
1095	chr3	26470316	-	chr6	77722512	+	Y	N	N	N	N	N	del_inso	d	FoSTeS	intergenic;ir	VENTXP4,AC099754.1	-,-	pseudogene	81187;190505	intergenic;ir	RP11-354K4.2,HTR1B	+,-	lincRNA,pro	236935;449436
1096	chr4	4350423	+	chr10	85222943	-	Y	N	N	N	N	N	del_inso	u	FoSTeS	intronic	NSG1	+	protein_cod	0	intergenic	AL356140.1,RP11-344L1	-,-	miRNA,lincF	26058;209219
1097	chr4	3636135	+	chr12	54478896	-	Y	N	N	N	N	N	del_inso	u	FoSTeS	downstream	RP3-368B9.2	+	lincRNA	0	ncRNA_intr	RP11-834C11.3,RP11-83	+,+	lincRNA,linc	0
1098	chr4	68150351	-	chr6	160656505	+	N	N	N	N	Y	N	del_inso	d	FoSTeS	intergenic	RNU6-699P,RP11-584I	+,-	snRNA,lincF	387262;132672	intronic	SLC22A2	-	protein_cod	0
1099	chr4	49268316	-	chrY	13267910	+	Y	N	N	Y	N	N	del_inso	d	FoSTeS	intergenic	RP11-1281K21.7,RP11	+,-	pseudogene	19384;220627	intergenic	RP1-85D24.1,AC134878	-,-	pseudogene	4347;72449
1100	chr5	49770934	-	chr1	121215151	+	N	N	N	Y	N	N	del_inso	d	FoSTeS	intergenic	EMB,RP11-269M20.2	-,+	protein_cod	33733;128129	intergenic	AL592494.5,RP11-344P1	+,-	lincRNA,linc	10168;21026
1101	chr5	49770982	-	chr1	121215571	+	N	N	Y	N	N	N	del_inso	d	FoSTeS	intergenic	EMB,RP11-269M20.2	-,+	protein_cod	33781;128081	intergenic	AL592494.5,RP11-344P1	+,-	lincRNA,linc	10588;20606
1102	chr5	35486157	+	chr13	114639942	-	N	Y	N	N	N	N	del_inso	u	FoSTeS	intergenic	U3,SPEF2	+,+	snoRNA,pro	56779;131789	intergenic	LINC00565,RASA3	-,-	lincRNA,pro	8125;107252
1103	chr5	175733389	+	chr18	55381820	-	N	N	N	N	Y	N	del_inso	u	FoSTeS	intronic	SIMC1	+	protein_cod	0	ncRNA_intr	RP11-35G9.3,RP11-35G	+,+	antisense,a	0
1104	chr5	79074614	+	chr8	92534263	-	N	Y	N	N	N	N	del_inso	u	FoSTeS	ncRNA_intr	CTC-431G16.2	-	antisense	0	intergenic	snoU13,RP11-122C21.1	-,-	snoRNA,linc	57896;20889
1105	chr6	10927634	-	chr15	100587624	+	N	N	N	N	Y	N	del_inso	d	FoSTeS	intronic	SYCP2L,RP11-637O1	+,+	protein_cod	0	intronic	ADAMTS17	-	protein_cod	0
1106	chr6	160656412	+	chr4	68150856	-	N	N	N	N	Y	N	del_inso	u	FoSTeS	intronic	SLC22A2	-	protein_cod	0	intergenic	RNU6-699P,RP11-584P2	+,-	snRNA,lincF	387767;132167
1107	chr6	144120386	-	chr7	153002453	+	N	Y	N	N	N	N	del_inso	d	FoSTeS	intronic	PHACTR2	+	protein_cod	0	intergenic	AC079809.2,AC073236.3	-,-	pseudogene	160780;94552
1108	chr6	63719714	-	chrX	102685593	+	N	N	Y	N	N	N	del_inso	d	FoSTeS	intergenic	AL590558.1,RP11-184	-,-	miRNA,pseu	26639;183263	intergenic	NGFRAP1,LL0XNC01-25	+,+	protein_cod	52588;66858
1109	chr6	63719768	-	chrX	102685505	+	N	N	N	Y	N	N	del_inso	d	FoSTeS	intergenic	AL590558.1,RP11-184	-,-	miRNA,pseu	26693;183209	intergenic	NGFRAP1,LL0XNC01-25	+,+	protein_cod	52500;66946
1110	chr7	64883044	+	chr12	43292316	-	N	N	N	Y	N	N	del_inso	u	FoSTeS	intergenic	ZNF92,RP11-667F9.2	+,+	protein_cod	17006;44940	intergenic	RP11-25I15.3,RP11-510F	+,-	lincRNA,pse	181663;31335
1111	chr7	63971332	-	chr18	77780755	+	N	Y	N	N	N	N	del_inso	d	FoSTeS	intergenic	HNRNPCP7,ZNF680	+,-	pseudogene	9225;8930	intronic	TXNL4A	-	protein_cod	0
1112	chr7	64289907	-	chr19	22314335	+	N	N	Y	N	N	N	del_inso	d	FoSTeS	intronic	ZNF138	+	protein_cod	0	ncRNA_intr	ZNF92P2	-	pseudogene	0
1113	chr7	153002421	+	chr6	144120580	-	N	Y	N	N	N	N	del_inso	u	FoSTeS	intergenic	AC079809.2,AC07323	-,-	pseudogene	160748;94584	intronic	PHACTR2	+	protein_cod	0
1114	chr8	11098805	+	chr18	22222917	-	N	Y	N	N	N	N	del_inso	u	FoSTeS	intergenic	AF131215.8,LINC0052	+,-	lincRNA,linc	37625;6330	ncRNA_intr	RP11-449D8.1	+	lincRNA	0
1115	chr8	21625055	+	chr21	47056645	-	N	N	N	N	Y	N	del_inso	u	FoSTeS	intronic	GFRA2	-	protein_cod	0	intergenic	AL133493.2,PCBP3	+,+	lincRNA,pro	39640;6963
1116	chr8	52070925	-	chr5	149050933	+	N	Y	N	N	N	N	del_inso	d	FoSTeS	intergenic	SNORA7,RP11-401H2	+,+	snoRNA,ant	143200;99323	intergenic	ARHGEF37,U3	+,-	protein_cod	36402;24379
1117	chr9	78523813	+	chr10	30078318	-	N	Y	N	N	N	N	del_inso	u	FoSTeS	intronic	PCSK5	+	protein_cod	0	intergenic	SVIL,RP11-224P11.1	-,-	protein_cod	53585;101643
1118	chr9	5904463	+	chr15	60537867	-	N	N	N	N	N	Y	del_inso	u	FoSTeS	intronic	MLANA,KIAA2026	+,-	protein_cod	0	intergenic	RP11-82L7.4,ANXA2	-,-	lincRNA,pro	127348;101466
1119	chr9	68492016	+	chr4	190819465	-	N	N	N	Y	N	N	del_inso	u	FoSTeS	intergenic	RP11-764K9.4,CR786	-,+	pseudogene	36641;20330	ncRNA_intr	AF146191.4	-	lincRNA	0
1120	chr9	88668615	+	chr8	83029872	-	N	N	N	N	Y	N	del_inso	u	FoSTeS	intronic	GOLM1	-	protein_cod	0	ncRNA_intr	RP11-99H20.1	-	lincRNA	0
1121	chr9	88805946	+	chrX	74928658	-	N	N	N	Y	Y	N	del_inso	u	FoSTeS	intergenic	RP11-379P1.4,C9orf15	+,-	pseudogene	58180;29234	intergenic	BRAFP1,TTC3P1	+,-	pseudogene	123317;31883
1122	chr9	88806122	+	chrX	74928698	-	N	N	Y	N	N	N	del_inso	u	FoSTeS	intergenic	RP11-379P1.4,C9orf15	+,-	pseudogene	58356;29058	intergenic	BRAFP1,TTC3P1	+,-	pseudogene	123357;31843
1123	chr9	68393475	-	chrY	9945989	+	Y	N	N	N	N	N	del_inso	d	FoSTeS	intergenic	PTGER4P3,RP11-764I	-,-	pseudogene	21598;4403	intergenic	RNA5SP519,AC006987.6	-,-	rRNA,pseuc	15387;61408
1124	chr9	68404572	-	chrY	9934963	+	N	Y	N	N	N	N	del_inso	d	complex_cor	ncRNA_intr	RP11-764K9.1	-	lincRNA	0	intergenic	RNA5SP519,AC006987.6	-,-	rRNA,pseuc	4361;72434
1125	chrX	53604462	-	chr19	24027922	+	N	Y	N	N	N	N	del_inso	d	FoSTeS	intronic	HUWE1	-	protein_cod	0	ncRNA_intr	RP11-255H23.4	-	lincRNA	0
1126	chrX	81651235	-	chr20	11281584	+	Y	N	N	Y	Y	Y	del_inso	d	EA_SINE_LIN	intergenic	RNU6-974P,RP4-581F	-,-	snRNA,pseu	394459;101883	ncRNA_intr	RP4-734C18.1	-	lincRNA	0
1127	chrX	102685356	+	chr6	63720560	-	N	N	Y	Y	N	N	del_inso	u	FoSTeS	intergenic	NGFRAP1,LL0XNC01-	+,+	protein_cod	52351;67095	intergenic	AL590558.1,RP11-184C2	-,-	miRNA,pseu	27485;182417
1128	chrX	65296781	-	chr9	130644826	+	N	N	N	Y	N	N	del_inso	d	FoSTeS	upstream	EIF4BP9	-	pseudogene	0	ncRNA_intr	RP11-203J24.9	-	processed_	0
1129	chrX	3351397	+	chrY	7431244	-	N	Y	Y	Y	Y	Y	del_inso	u	FoSTeS	intergenic	ASS1P4,SNORA48	+,-	pseudogene	64974;98761	intergenic	RNU6-521P,RBMY2HP	-,-	snRNA,pseu	140045;108540
1130	chrY	13447010	+	chr1	142543016	-	N	N	N	N	N	Y	del_inso	u	FoSTeS	intergenic	AC134878.1,DUX4L16	-,+	miRNA,pseu	106377;15584	intergenic	NONE,RP11-417J8.1	NONE,+	NONE,lincR	NONE;10277
1131	chrY	9962124	+	chr19	58322839	-	Y	N	N	N	N	N	del_inso	u	FoSTeS	intergenic	RNA5SP519,AC00698	-,-	rRNA,pseuc	31522;45273	intronic	ZNF586,ZNF552	+,-	protein_cod	0
1132	chrY	10033577	-	chr2	133015079	+	N	N	N	Y	N	N	del_inso	d	FoSTeS	upstream	AC010970.1	+	miRNA	0	upstream	MIR663B	-	miRNA	0
1133	chrY	10033277	+	chr2	133015917	-	N	N	N	N	N	Y	del_inso	u	FoSTeS	upstream	AC010970.1	+	miRNA	0	intergenic	MIR663B,CDC27P1	-,+	miRNA,pseu	1264;3984
1134	chrY	13267574	+	chr4	49268622	-	Y	N	N	N	N	N	del_inso	u	FoSTeS	intergenic	RP1-85D24.1,AC1348	-,-	pseudogene	4011;72785	intergenic	RP11-1281K21.7,RP11-2	+,-	pseudogene	19690;220321
1135	chrY	9929993	+	chr9	68409382	-	Y	N	N	N	N	N	del_inso	u	FoSTeS	downstream	RNA5SP519	-	rRNA	0	ncRNA_intr	RP11-764K9.1	-	lincRNA	0
1136	chrY	9930452	+	chr9	68409117	-	N	Y	N	N	N	N	del_inso	u	complex_cor	downstream	RNA5SP519	-	rRNA	0	ncRNA_intr	RP11-764K9.1	-	lincRNA	0
1137	chrY	7430757	-	chrX	3351778	+	N	N	N	N	Y	N	del_inso	d	FoSTeS	intergenic	RNU6-521P,RBMY2HF	-,-	snRNA,pseu	139558;109027	intergenic	ASS1P4,SNORA48	+,-	pseudogene	65355;98380
1138	chrY	7430761	-	chrX	3351822	+	N	N	Y	N	N	N	del_inso	d	FoSTeS	intergenic	RNU6-521P,RBMY2HF	-,-	snRNA,pseu	139562;109023	intergenic	ASS1P4,SNORA48	+,-	pseudogene	65399;98336
1139	chrY	7430767	-	chrX	3351738	+	N	N	N	Y	N	N	del_inso	d	FoSTeS	intergenic	RNU6-521P,RBMY2HF	-,-	snRNA,pseu	139568;109017	intergenic	ASS1P4,SNORA48	+,-	pseudogene	65315;98420
1140	chr1	167175202	-	chr1	113206276	+	N	N	Y	N	N	N	del_insod	d	FoSTeS	intergenic	RP11-277B15.2,RP11-	-,-	antisense,a	10173;13866	intronic	CAPZA1	+	protein_cod	0
1141	chr1	181044092	-	chr1	181043901	+	N	N	Y	N	N	N	del_insod	d	FoSTeS	intergenic;ir	MR1,IER5	+,+	protein_cod	13018;13546	intergenic;ir	MR1,IER5	+,+	protein_cod	12827;13737
1142	chr1	187466731	-	chr1	187466474	+	N	Y	N	Y	N	N	del_insod	d	FoSTeS	intergenic;ir	RP11-445P19.3,RP11-	+,-	lincRNA,pse	16985;9239	intergenic;ir	RP11-445P19.3,RP11-44	+,-	lincRNA,pse	16728;9496
1143	chr1	111106753	+	chr1	181869288	-	N	N	Y	N	N	N	del_insod	u	FoSTeS	intergenic	KCNA10,KCNA2	-,-	protein_cod	44956;29449	intergenic	RN7SKP229,ZNF648	-,-	misc_RNA,p	60379;154417
1144	chr1	181043278	+	chr1	181044439	-	N	N	Y	N	N	N	del_insod	u	FoSTeS	intergenic	MR1,IER5	+,+	protein_cod	12204;14360	intergenic	MR1,IER5	+,+	protein_cod	13365;13199
1145	chr10	54712086	-	chr10	38228943	+	Y	N	N	N	N	Y	del_insod	d	FoSTeS	intergenic;ir	Y_RNA,RP11-319F12.	+,-	misc_RNA,l	99623;21162	intergenic;ir	ZNF33AP1,ZNF25	+,-	pseudogene	43349;9557
1146	chr11	24287579	-	chr11	24285122	+	Y	N	Y	N	N	N	del_insod	d	FoSTeS	intergenic;ir	RP11-2F20.1,Y_RNA	-,+	lincRNA,mis	3815;189878	intergenic;ir	RP11-2F20.1,Y_RNA	-,+	lincRNA,mis	1358;192335
1147	chr11	31666769	-	chr11	18751611	+	Y	N	N	N	N	N	del_insod	d	FoSTeS	ncRNA_intr	Z83001.1	-	antisense	0	ncRNA_intr	RP11-1081L13.4	+	antisense	0
1148	chr11	55431551	-	chr11	55364889	+	N	N	N	Y	N	N	del_insod	d	FoSTeS	intergenic	OR4S2,OR4C6	+,+	protein_cod	12236;1063	intergenic	OR4C16,OR4C11	+,-	protein_cod	24353;5941
1149	chr11	15708282	+	chr11	47914868	-	N	N	Y	N	N	N	del_insod	u	FoSTeS	ncRNA_intr	RP11-396O20.1	+	lincRNA	0	intergenic	NUP160,RP11-793I11.1	-,-	protein_cod	44761;12007
1150	chr11	18749909	+	chr11	31803615	-	Y	N	N	N	N	N	del_insod	u	FoSTeS	ncRNA_intr	RP11-1081L13.4	+	antisense	0	intronic	ELP4	+	protein_cod	0
1151	chr11	24284731	+	chr11	24287914	-	Y	N	N	N	N	N	del_insod	u	FoSTeS	upstream	RP11-2F20.1	-	lincRNA	0	intergenic	RP11-2F20.1,Y_RNA	-,+	lincRNA,mis	4150;189543
1152	chr11	55364519	+	chr11	55434496	-	N	N	N	Y	N	N	del_insod	u	FoSTeS	intergenic;ir	OR4C16,OR4C11	+,-	protein_cod	23983;6311	downstream	OR4C6	+	protein_cod	0
1153	chr12	78387842	-	chr12	78383887	+	Y	Y	Y	Y	Y	Y	del_insod	d	FoSTeS	intronic	NAV3	+	protein_cod	0	intronic	NAV3	+	protein_cod	0
1154	chr12	78383875	+	chr12	78388274	-	Y	Y	Y	Y	Y	Y	del_insod	u	FoSTeS	intronic	NAV3	+	protein_cod	0	intronic	NAV3	+	protein_cod	0
1155	chr13	89112881	-	chr13	89110970	+	N	N	N	Y	N	N	del_insod	d	FoSTeS	intergenic;ir	RPL29P29,LINC00433	-,+	pseudogene	223657;80238	intergenic;ir	RPL29P29,LINC00433	-,+	pseudogene	221746;82149
1156	chr15	76552165	-	chr15	41864762	+	N	Y	N	N	N	Y	del_insod	d	FoSTeS	ncRNA_exo	TYRO3P	-	pseudogene	0	exonic	TYRO3	+	protein_cod	0
1157	chr15	76552197	-	chr15	41864730	+	N	N	N	Y	N	N	del_insod	d	FoSTeS	ncRNA_exo	TYRO3P	-	pseudogene	0	exonic	TYRO3	+	protein_cod	0
1158	chr15	41853488	+	chr15	76553757	-	N	N	N	Y	N	N	del_insod	u	FoSTeS	exonic;exon	TYRO3	+	protein_cod	0	ncRNA_exo	TYRO3P	-	pseudogene	0
1159	chr15	41853699	+	chr15	76553658	-	N	Y	N	N	N	Y	del_insod	u	FoSTeS	intronic	TYRO3	+	protein_cod	0	ncRNA_exo	TYRO3P	-	pseudogene	0
1160	chr17	36401098	-	chr17	36399834	+	N	N	N	N	Y	N	del_insod	d	FoSTeS	intronic	RP11-1407O15.2	-	protein_cod	0	intronic	RP11-1407O15.2	-	protein_cod	0
1161	chr17	36350387	+	chr17	36406169	-	N	N	N	N	Y	N	del_insod	u	FoSTeS	intronic;intro	RP11-1407O15.2,TBC	-,-	protein_cod	0	intronic;intro	RP11-1407O15.2	-	protein_cod	0
1162	chr19	16014859	-	chr19	15823087	+	N	N	N	Y	N	N	del_insod	d	FoSTeS	upstream	AC005336.5	-	pseudogene	0	intergenic	CYP4F12,OR10H2	+,+	protein_cod	15103;15747
1163	chr19	15822375	+	chr19	16015182	-	N	N	N	Y	N	N	del_insod	u	FoSTeS	intergenic;ir	CYP4F12,OR10H2	+,+	protein_cod	14391;16459	intergenic;ir	AC005336.5,AC005336.4	-,-	pseudogene	1134;6210
1164	chr2	184571027	-	chr2	184569190	+	Y	Y	Y	Y	Y	Y	del_insod	d	FoSTeS	intergenic	CACYBPP2,AC093639	-,+	pseudogene	98180;198229	intergenic	CACYBPP2,AC093639.1	-,+	pseudogene	96343;200066
1165	chr2	228075808	-	chr2	228075151	+	N	N	N	Y	N	N	del_insod	d	FoSTeS	intronic	COL4A3	+	protein_cod	0	intronic	COL4A3	+	protein_cod	0
1166	chr2	184569177	+	chr2	184572010	-	Y	Y	Y	Y	Y	Y	del_insod	u	FoSTeS	intergenic	CACYBPP2,AC093639	-,+	pseudogene	96330;200079	intergenic	CACYBPP2,AC093639.1	-,+	pseudogene	99163;197246
1167	chr2	228075134	+	chr2	228075906	-	N	N	N	Y	N	N	del_insod	u	FoSTeS	intronic;intro	COL4A3	+	protein_cod	0	intronic	COL4A3	+	protein_cod	0
1168	chr20	18595822	-	chr20	18591558	+	Y	Y	Y	N	Y	Y	del_insod	d	FoSTeS	intronic;intro	DTD1	+	protein_cod	0	intronic;intro	DTD1	+	protein_cod	0
1169	chr20	18591541	+	chr20	18595879	-	Y	Y	Y	N	Y	Y	del_insod	u	FoSTeS	intronic	DTD1	+	protein_cod	0	intronic	DTD1	+	protein_cod	0
1170	chr21	28021726	-	chr21	28020682	+	N	Y	N	Y	N	N	del_insod	d	FoSTeS	intergenic	CYYR1,ADAMTS1	-,-	protein_cod	76123;186340	intergenic	CYYR1,ADAMTS1	-,-	protein_cod	75079;187384

1171	chr21	23626205	+	chr21	29293556	-	N	Y	N	Y	N	N	del_insod	u	FoSTeS	ncRNA_intr	AP000705.7	+	lincRNA	0	intergenic	LINC00113,AJ006995.3	+,+	lincRNA,linc	170004;33684
1172	chr4	163578994	-	chr4	163578943	+	N	N	N	N	Y	N	del_insod	d	FoSTeS	intergenic	TOMM22P4,AC00515(	+,+	pseudogene	135801;82826	intergenic	TOMM22P4,AC005150.1	+,+	pseudogene	135750;82877
1173	chr4	187245816	-	chr4	185911516	+	N	N	Y	N	N	N	del_insod	d	FoSTeS	ncRNA_intr	F11-AS1	-	antisense	0	ncRNA_intr	RP11-386B13.4	+	lincRNA	0
1174	chr4	78905965	+	chr4	190684480	-	N	N	Y	N	N	N	del_insod	u	FoSTeS	intergenic;ir	HMGB1P44,HNRNPA1	+,-	pseudogene	20300;3049	intergenic;ir	RNU1-51P,AF146191.4	-,-	snRNA,lincF	53612;17010
1175	chr4	163578583	+	chr4	163579619	-	N	N	N	N	Y	N	del_insod	u	FoSTeS	intergenic;ir	TOMM22P4,AC00515(	+,+	pseudogene	135390;83237	intergenic;ir	TOMM22P4,AC005150.1	+,+	pseudogene	136426;82201
1176	chr5	28934874	-	chr5	28932852	+	Y	Y	Y	Y	Y	Y	del_insod	d	FoSTeS	intergenic	LSP1P3,SUCLG2P4	+,+	pseudogene	7178;66993	intergenic	LSP1P3,SUCLG2P4	+,+	pseudogene	5156;69015
1177	chr5	28932650	+	chr5	28935058	-	Y	Y	Y	Y	Y	Y	del_insod	u	FoSTeS	intergenic	LSP1P3,SUCLG2P4	+,+	pseudogene	4954;69217	intergenic	LSP1P3,SUCLG2P4	+,+	pseudogene	7362;66809
1178	chr6	131884928	-	chr6	131884765	+	N	N	N	N	Y	N	del_insod	d	FoSTeS	intergenic	RPL21P67,ARG1	+,+	pseudogene	94252;9356	intergenic	RPL21P67,ARG1	+,+	pseudogene	94089;9519
1179	chr6	161206729	-	chr6	153749313	+	Y	Y	Y	Y	Y	Y	del_insod	d	FoSTeS	intergenic	PLG,RP11-235G24.1	+,-	protein_cod	32391;87191	intergenic	RNA5SP225,RP11-331O	+,+	rRNA,pseuc	7606;237529
1180	chr6	131884281	+	chr6	131885007	-	N	N	N	N	Y	N	del_insod	u	FoSTeS	intergenic	RPL21P67,ARG1	+,+	pseudogene	93605;10003	intergenic	RPL21P67,ARG1	+,+	pseudogene	94331;9277
1181	chr6	153749250	+	chr6	161206937	-	Y	Y	Y	Y	Y	Y	del_insod	u	FoSTeS	intergenic	RNA5SP225,RP11-33	+,+	rRNA,pseuc	7543;237592	intergenic	PLG,RP11-235G24.1	+,-	protein_cod	32599;86983
1182	chr7	142183747	-	chr7	142045948	+	N	Y	Y	N	N	Y	del_insod	d	FoSTeS	intergenic;ir	TRBV6-5,TRBV12-2	-,-	TR_V_gene	2731;6891	downstream	TRBV4-2	+	TR_V_gene	0
1183	chr7	142183781	-	chr7	142045948	+	Y	N	N	Y	N	N	del_insod	d	FoSTeS	intergenic	TRBV6-5,TRBV12-2	-,-	TR_V_gene	2765;6857	downstream	TRBV4-2	+	TR_V_gene	0
1184	chr7	147571592	-	chr7	124662610	+	N	N	N	N	Y	N	del_insod	d	FoSTeS	intronic;intrc	CNTNAP2	+	protein_cod	0	ncRNA_intr	POT1-AS1	+	antisense	0
1185	chr7	124662180	+	chr7	147571853	-	N	N	N	N	Y	N	del_insod	u	FoSTeS	ncRNA_intr	POT1-AS1	+	antisense	0	intronic	CNTNAP2	+	protein_cod	0
1186	chr7	134506328	+	chr7	134520884	-	Y	N	N	N	Y	N	del_insod	u	FoSTeS	intronic	CALD1	+	protein_cod	0	intronic	CALD1	+	protein_cod	0
1187	chr7	142031064	+	chr7	142247782	-	N	N	N	Y	N	Y	del_insod	u	FoSTeS	upstream	TRBV7-1	+	TR_V_gene	0	upstream	TRBV7-3	-	TR_V_gene	0
1188	chr7	142031256	+	chr7	142247774	-	Y	N	Y	N	N	N	del_insod	u	FoSTeS	upstream	TRBV7-1	+	TR_V_gene	0	upstream	TRBV7-3	-	TR_V_gene	0
1189	chr7	142031825	+	chr7	142247724	-	N	Y	N	N	N	N	del_insod	u	FoSTeS	upstream	TRBV7-1	+	TR_V_gene	0	upstream	TRBV7-3	-	TR_V_gene	0
1190	chr8	71767847	-	chr8	68667795	+	N	Y	N	N	N	N	del_insod	d	FoSTeS	intergenic	Y_RNA,RP11-326E22.	-,+	misc_RNA,a	74604;299845	intergenic	CPA6,NDUFS5P6	-,+	protein_cod	9175;93484
1191	chr8	68667234	+	chr8	71768278	-	N	Y	N	N	N	N	del_insod	u	FoSTeS	intergenic	CPA6,NDUFS5P6	-,+	protein_cod	8614;94045	intergenic	Y_RNA,RP11-326E22.1	-,+	misc_RNA,a	75035;299414
1192	chrX	51965760	-	chrX	51279906	+	N	N	N	Y	N	Y	del_insod	d	FoSTeS	intergenic	RP11-363G10.2,XAGE	+,+	protein_cod	19402;146413	intergenic	RP11-56H2.2,CENPVP3	-,-	lincRNA,pse	26049;81055
1193	chrX	51279613	+	chrX	51966001	-	N	N	N	Y	N	N	del_insod	u	FoSTeS	intergenic	RP11-56H2.2,CENPVF	-,-	lincRNA,pse	25756;81348	intergenic	RP11-363G10.2,XAGE2E	+,+	protein_cod	19643;146172
1194	chrX	51279828	+	chrX	51965915	-	N	N	N	N	N	Y	del_insod	u	FoSTeS	intergenic	RP11-56H2.2,CENPVF	-,-	lincRNA,pse	25971;81133	intergenic	RP11-363G10.2,XAGE2E	+,+	protein_cod	19557;146258
1195	chr1	6507257	-	chr1	17027918	+	N	N	N	N	N	Y	del_insou	d	FoSTeS	intronic	ESPN	+	protein_cod	0	ncRNA_intr	ESPNP	-	pseudogene	0
1196	chr1	105412504	-	chr1	219161902	+	N	N	N	N	N	Y	del_insou	d	FoSTeS	intergenic	FTLP17,RP11-414B7.1	-,+	pseudogene	716052;564112	intergenic	RP11-412H9.2,RP11-135	-,-	lincRNA,ant	72376;98042
1197	chr1	113206277	-	chr1	167175201	+	Y	Y	N	N	N	N	del_insou	d	FoSTeS	intronic	CAPZA1	+	protein_cod	0	intergenic	RP11-277B15.2,RP11-27	-,-	antisense,a	10172;13867
1198	chr1	114645953	-	chr1	114654366	+	Y	Y	Y	Y	Y	Y	del_insou	d	FoSTeS	intronic	SYT6	-	protein_cod	0	intronic	SYT6	-	protein_cod	0
1199	chr1	12950025	+	chr1	12909375	-	N	N	N	N	N	Y	del_insou	u	FoSTeS	intergenic	PRAMEF4,PRAMEF1(	-,-	protein_cod	4000;2702	upstream	HNRNPCL1	-	protein_cod	0
1200	chr1	114654350	+	chr1	114646029	-	Y	Y	Y	Y	Y	Y	del_insou	u	FoSTeS	intronic	SYT6	-	protein_cod	0	intronic	SYT6	-	protein_cod	0
1201	chr1	147394544	+	chr1	145833078	-	N	N	N	N	Y	N	del_insou	u	FoSTeS	intergenic	GJA8,GPR89B	+,+	protein_cod	13151;5962	ncRNA_exo	WI2-925H4.1	+	antisense	0
1202	chr1	187106943	+	chr1	185963183	-	N	N	N	N	N	Y	del_insou	u	FoSTeS	ncRNA_intr	LINC01036	+	lincRNA	0	intronic	HMCN1	+	protein_cod	0
1203	chr10	47022999	-	chr10	47059938	+	N	N	N	N	N	Y	del_insou	d	FoSTeS	ncRNA_intr	LINC00842	-	lincRNA	0	ncRNA_intr	LINC00842	-	lincRNA	0
1204	chr10	127190418	-	chr10	127201102	+	Y	N	Y	Y	Y	Y	del_insou	d	FoSTeS	intergenic	RPS27P18,TEX36-AS	-,+	pseudogene	27917;72522	intergenic	RPS27P18,TEX36-AS1	-,+	pseudogene	38601;61838
1205	chr10	47059608	+	chr10	47023090	-	N	N	N	N	N	Y	del_insou	u	FoSTeS	ncRNA_intr	LINC00842	-	lincRNA	0	ncRNA_intr	LINC00842	-	lincRNA	0
1206	chr10	127197223	+	chr10	127190677	-	Y	N	Y	Y	Y	Y	del_insou	u	FoSTeS	intergenic	RPS27P18,TEX36-AS	-,+	pseudogene	34722;65717	intergenic	RPS27P18,TEX36-AS1	-,+	pseudogene	28176;72263
1207	chr11	1722794	-	chr11	68924896	+	N	N	N	N	N	Y	del_insou	d	FoSTeS	intergenic	KRTAP5-6,IFITM10	+,-	protein_cod	3809;30846	ncRNA_intr	RP11-554A11.9,RP11-55	+,-	antisense,li	0
1208	chr11	90421439	-	chr11	91809130	+	N	N	N	Y	N	N	del_insou	d	FoSTeS	ncRNA_intr	DISC1FP1	+	processed_	0	intergenic;ir	TUBB4BP4,RPL7AP57	-,+	pseudogene	125930;85142
1209	chr11	91808579	+	chr11	90422128	-	N	N	N	Y	N	N	del_insou	u	FoSTeS	intergenic	TUBB4BP4,RPL7AP57	-,+	pseudogene	125379;85693	ncRNA_intr	DISC1FP1	+	processed_	0
1210	chr11	99691307	+	chr11	99690483	-	Y	N	Y	N	N	N	del_insou	u	FoSTeS	intronic	CNTN5	+	protein_cod	0	exonic	CNTN5	+	protein_cod	0
1211	chr11	100349339	+	chr11	98146224	-	N	N	N	N	Y	N	del_insou	u	FoSTeS	intergenic	CNTN5,RN7SL222P	+,-	protein_cod	119723;163370	intergenic	RP11-684B20.1,CTD-234	-,-	pseudogene	144298;289580
1212	chr12	9722827	-	chr12	31271917	+	N	N	N	N	N	Y	del_insou	d	FoSTeS	ncRNA_intr	RP11-726G1.1	+	pseudogene	0	ncRNA_exo	RP11-551L14.1	-	pseudogene	0
1213	chr12	25956127	-	chr12	125801148	+	N	Y	N	N	N	N	del_insou	d	FoSTeS	ncRNA_intr	RP11-443N24.2	-	lincRNA	0	intergenic;ir	NONE,TMEM132B	NONE,+	NONE,prote	NONE;10014
1214	chr12	30501742	+	chr12	30404007	-	N	N	N	N	N	Y	del_insou	u	FoSTeS	intergenic	RP11-155I9.1,RP11-77	+,+	lincRNA,pse	27192;106110	ncRNA_intr	RP11-776A13.3	-	lincRNA	0
1215	chr14	21331497	-	chr14	22226998	+	N	N	Y	N	N	N	del_insou	d	FoSTeS	intergenic	RNASE1,RP11-219E7.	-,-	protein_cod	60060;6940	intergenic	RPL4P1,TRAV6	+,+	pseudogene	7168;9731
1216	chr15	41864762	-	chr15	76552165	+	Y	N	N	N	N	N	del_insou	d	FoSTeS	exonic;exon	TYRO3	+	protein_cod	0	ncRNA_exo	TYRO3P	-	pseudogene	0
1217	chr15	52264998	+	chr15	52264395	-	Y	N	N	N	N	N	del_insou	u	FoSTeS	upstream	LEO1	-	protein_cod	0	upstream	LEO1	-	protein_cod	0
1218	chr16	85189757	+	chr16	85188795	-	N	N	N	Y	N	N	del_insou	u	FoSTeS	intergenic	FAM92B,CTC-786C10	-,+	protein_cod	43643;15125	intergenic	FAM92B,CTC-786C10.1	-,+	protein_cod	42681;16087
1219	chr19	16015207	+	chr19	15822401	-	N	N	N	N	N	Y	del_insou	u	A_LINE_com	intergenic	AC005336.5,AC00533(	-,-	pseudogene	1159;6185	intergenic	CYP4F12,OR10H2	+,+	protein_cod	14417;16433
1220	chr4	3931305	-	chr4	4005427	+	N	N	Y	N	N	N	del_insou	d	FoSTeS	ncRNA_intr	AC226119.5	-	processed_	0	intergenic	ENPP7P9,AC116562.2	-,+	pseudogene	36040;33384
1221	chr4	4005243	+	chr4	3931747	-	N	N	Y	N	N	N	del_insou	u	FoSTeS	intergenic	ENPP7P9,AC116562.2	-,+	pseudogene	35856;33568	ncRNA_intr	AC226119.5	-	processed_	0
1222	chr4	28960170	+	chr4	28857521	-	Y	N	N	N	N	N	del_insou	u	FoSTeS	intergenic	MESTP3,RP11-292B1.	-,+	pseudogene	134337;37976	intergenic	MESTP3,RP11-292B1.2	-,+	pseudogene	31688;140625
1223	chr4	28960351	+	chr4	28857310	-	N	N	N	N	N	Y	del_insou	u	FoSTeS	intergenic	MESTP3,RP11-292B1.	-,+	pseudogene	134518;37795	intergenic	MESTP3,RP11-292B1.2	-,+	pseudogene	31477;140836
1224	chr4	70249433	+	chr4	70086206	-	N	N	Y	N	N	N	del_insou	u	FoSTeS	intergenic	RP11-618I10.2,RP11-6	+,+	pseudogene	25538;3865	intergenic	RP11-704M14.1,RP11-61	+,+	antisense,p	3722;22140
1225	chr5	99317522	-	chr5	104095000	+	N	N	N	N	Y	N	del_insou	d	FoSTeS	intergenic	CTD-2160D9.1,EEF1A	-,+	pseudogene	32235;14729	ncRNA_intr	RP11-6N13.1	-	lincRNA	0
1226	chr6	131884334	-	chr6	131885333	+	N	N	N	N	N	Y	del_insou	d	FoSTeS	intergenic	RPL21P67,ARG1	+,+	pseudogene	93658;9950	intergenic	RPL21P67,ARG1	+,+	pseudogene	94657;8951
1227	chr6	131884935	+	chr6	131884409	-	N	N	N	N	N	Y	del_insou	u	FoSTeS	intergenic	RPL21P67,ARG1	+,+	pseudogene	94259;9349	intergenic	RPL21P67,ARG1	+,+	pseudogene	93733;9875
1228	chr7	31586772	-	chr7	31591996	+	Y	Y	Y	Y	Y	Y	del_insou	d	FoSTeS	intronic	CCDC129	+	protein_cod	0	intronic	CCDC129	+	protein_cod	0
1229	chr7	91110408	-	chr7	91137764	+	N	N	Y	N	N	N	del_insou	d	FoSTeS	ncRNA_intr	RP11-142A5.1,RP11-1	-,+	lincRNA,linc	0	ncRNA_intr	RP11-142A5.1,RP11-115	-,+	lincRNA,linc	0
1230	chr7	91110500	-	chr7	91137515	+	Y	Y	N	N	Y	N	del_insou	d	FoSTeS	ncRNA_intr	RP11-142A5.1,RP11-1	-,+	lincRNA,linc	0	ncRNA_intr	RP11-142A5.1,RP11-115	-,+	lincRNA,linc	0
1231	chr7	124662610	-	chr7	147571592	+	N	N	N	N	N	Y	del_insou	d	FoSTeS	ncRNA_intr	POT1-AS1	+	antisense	0	intronic;intrc	CNTNAP2	+	protein_cod	0
1232	chr7	31590271	+	chr7	31587029	-	Y	Y	Y	Y	Y	Y	del_insou	u	FoSTeS	intronic	CCDC129	+	protein_cod	0	intronic	CCDC129	+	protein_cod	0
1233	chr7	91137134	+	chr7	91110992	-	Y	N	N	N	Y	N	del_insou	u	FoSTeS	ncRNA_intr	RP11-142A5.1,RP11-1	-,+	lincRNA,linc	0	ncRNA_intr	RP11-142A5.1,RP11-115	-,+	lincRNA,linc	0
1234	chr7	91137187	+	chr7	91110951	-	N	Y	N	N	N	N	del_insou	u	FoSTeS	ncRNA_intr	RP11-142A5.1,RP11-1	-,+	lincRNA,linc	0	ncRNA_intr	RP11-142A5.1,RP11-115	-,+	lincRNA,linc	0
1235	chr7	91137354	+	chr7	91110913	-	N	N	Y	N	N	N	del_insou	u	FoSTeS	ncRNA_intr	RP11-142A5.1,RP11-1	-,+	lincRNA,linc	0	ncRNA_intr	RP11-142A5.1,RP11-115	-,+	lincRNA,linc	0
1236	chr7	142251707	+	chr7	142048165	-	N	N	N	N	N	Y	del_insou	u	FoSTeS	upstream	TRBV6-4	-	TR_V_gene	0	intergenic	TRBV4-2,TRBV7-8	+,-	TR_V_gene	2349;51290
1237	chr9	2278924	-	chr9	3813494	+	N	N	N	Y	N	N	del_insou	d	FoSTeS	intergenic	RN7SL592P,RP11-125	-,-	misc_RNA,a	49683;143778	intergenic	RP11-509J21.3,GLIS3	+,-	lincRNA,prc	121680;10633
1238	chr9	2279032	-	chr9	3813387	+	N	N	N	N	Y	N	del_insou	d	FoSTeS	intergenic	RN7SL592P,RP11-125	-,-	misc_RNA,a	49791;143670	intergenic	RP11-509J21.3,GLIS3	+,-	lincRNA,prc	121573;10740
1239	chr9	2279100	-	chr9	3813359	+	Y	N	Y	N	N	N	del_insou	d	FoSTeS	intergenic	RN7SL592P,RP11-125	-,-	misc_RNA,a	49859;143602	intergenic	RP11-509J21.3,GLIS3	+,-	lincRNA,prc	121545;10768
1240	chr9	66454799	-	chr9	68412307	+	N	N	N	N	Y	N	del_insou	d	FoSTeS	intergenic	RP11-459O16.7,RP11-	+,+	pseudogene	57686;2486	intergenic	RP11-764K9.1,LINC0053	-,-	lincRNA,pse	2414;1175
1241	chr9	66455074	-	chr9	68412065	+	N	Y	Y	Y	N	N	del_insou	d	FoSTeS	intergenic	RP11-459O16.7,RP11-	+,+	pseudogene	57961;2211	intergenic	RP11-764K9.1,LINC0053	-,-	lincRNA,pse	2172;1417
1242	chr9	3812868	+	chr9	2279611	-	Y	N	Y	N	Y	N	del_insou	u	FoSTeS	intergenic	RP11-509J21.3,GLIS3	+,-	lincRNA,prc	121054;11259	intergenic	RN7SL592P,RP11-125B2	-,-	misc_RNA,a	50370;143091
1243	chr9	66474386	+	chr9	66462153	-	N	N	Y	Y	N	N	del_insou	u	FoSTeS	downstream	AL512625.1	+	pseudogene	0	ncRNA_exo	RP11-262H14.1	+	lincRNA	0
1244	chr9	68377525	+	chr9	66491185	-	N	Y	N	N	Y	N	del_insou	u	FoSTeS	intergenic	PTGER4P3,RP11-764I	-,-	pseudogene	5648;20353	intergenic	AL512625.1,RP11-262H1	+,-	pseudogene	17512;2387
1245	chr9	68429244	+	chr9	68421834	-	Y	N	N	N	N	N	del_insou	u	FoSTeS	ncRNA_intr	RP11-764K9.4	-	pseudogene	0	intergenic	MIR4477A,RP11-764K9.4	+,-	miRNA,pse	6446;6027
1246	chrX	30662412	-	chrX	147567865	+	N	N	N	N	Y	N	del_insou	d	FoSTeS	intergenic	FTLP2,GK	+,+	pseudogene	13470;9064	intergenic	RPL7L1P11,AC002368.4	+,-	pseudogene	20917;11768
1247	chr1	102114765	+	chr13	64826021	+	Y	Y	N	N	N	N	del_inss	d	FoSTeS	intergenic;ir	RP11-157N3.1,RP11-4	+,+	lincRNA,linc	258906;74533	intergenic;ir	LINC00355,LGMNP1	-,-	lincRNA,pse	175877;706208
1248	chr1	98978471	+	chr16	28677231	+	N	Y	N	N	N	N	del_inss	u	FoSTeS	intergenic	AL160056.1,SNX7	+,+	miRNA,prot	139416;148765	ncRNA_intr	RP11-57A19.4	-	pseudogene	0
1249	chr1	104480855	+	chr3	113965502	+	N	N	N	Y	Y	N	del_inss	u	FoSTeS	intergenic;ir	RP11-97C18.1,RP11-3	+,+	pseudogene	10427;134777	intergenic;ir	ZNF80,TIGIT	-,+	protein_cod	9077;30258
1250	chr1	147392538	+	chr3	48706151	+	N	N	N	Y	N	N	del_inss	u	FoSTeS	intergenic;ir	GJA8,GPR89B	+,+	protein_cod	11145;7968	ncRNA_intr	RP11-148G20.1	+	antisense	0
1251	chr1	85953831	+	chr7	942060	+	N	N	Y	N	N	N	del_inss	u	FoSTeS	intronic	DDAH1	-	protein_cod	0	intronic	ADAP1,COX19	-,-	protein_cod	0
1252	chr1	149118006	+	chr8	26413671	+	N	Y	N	N	N	N	del_inss	u	FoSTeS	ncRNA_intr	NBPF23	-	pseudogene	0	intronic	DPYSL2	+	protein_cod	0
1253	chr1	164989676	+	chrX	133182265	+	N	Y	N	N	N	N	del_inss	u	FoSTeS	intergenic	AL390119.1,SNORD11	-,-	miRNA,snol	36447;52034	intergenic	AF003529.2,Y_RNA	+,-	pseudogene	58152;93993
1254	chr10	47059672	+	chr11	89183630	+	N	N	N	Y	N	N	del_inss	u	FoSTeS	ncRNA_intr	LINC00842	-	lincRNA	0	intronic	NOX4	-	protein_cod	0

1255	chr10	1394181	+	chr14	59913999	+	N	N	Y	N	N	N	del_inss	u	FoSTeS	intronic	ADARB2	-	protein_cod	0	intronic	GPR135	-	protein_cod	0
1256	chr10	50462280	+	chr8	144263158	+	N	N	N	Y	N	N	del_inss	u	FoSTeS	intergenic;ir	C10orf128,C10orf71-A	-,-	protein_cod	65844;41877	intergenic	LY6H,GPIHBP1	-,+	protein_cod	21030;31910
1257	chr11	134221128	+	chr10	133431405	+	N	Y	N	Y	N	N	del_inss	d	FoSTeS	intronic	GLB1L2	+	protein_cod	0	intergenic	TCERG1L,AL450307.2	-,+	protein_cod	321421;58110
1258	chr11	110267640	+	chr13	23580846	+	N	N	N	N	N	Y	del_inss	d	FoSTeS	ncRNA_intr	RP11-347E10.1	+	lincRNA	0	intergenic	AL157931.1,HMGA1P6	-,+	miRNA,pseu	28710;127467
1259	chr11	4420710	+	chr6	133938136	+	N	N	N	N	N	Y	del_inss	u	FoSTeS	intergenic	TRIM21,OR51R1P	-,+	protein_cod	5784;18580	ncRNA_intr	RP3-323P13.2	-	antisense	0
1260	chr11	56474682	+	chr6	139666544	+	N	N	N	Y	N	N	del_inss	u	FoSTeS	intergenic	OR9G1,RP11-100N3.2	+,-	protein_cod	5901;29110	intergenic	RP11-12A2.1,CITED2	+,-	pseudogene	5827;26849
1261	chr11	70835253	+	chrY	13204033	+	Y	N	N	N	N	N	del_inss	u	FoSTeS	intronic	SHANK2	-	protein_cod	0	intergenic	NONE,RP1-85D24.3	NONE,-	NONE,pseu	NONE;58708
1262	chr12	33456332	+	chr1	103913907	+	N	N	N	N	N	Y	del_inss	d	FoSTeS	intergenic	AC026357.1,SNORD1	-,-	miRNA,snol	261577;59186	intergenic	RP11-347K2.2,RP11-153	-,+	lincRNA,linc	77921;43594
1263	chr12	51222886	+	chr2	214171082	+	N	Y	N	Y	Y	N	del_inss	d	FoSTeS	intergenic	ATF1,RN7SL519P	+,-	protein_cod	7981;12397	intronic	SPAG16	+	protein_cod	0
1264	chr12	121490598	+	chr3	183529734	+	N	N	N	N	Y	N	del_inss	d	FoSTeS	intergenic	OASL,RP11-216P16.4	-,+	protein_cod	13553;27047	UTR3	YEATS2	+	protein_cod	0
1265	chr13	21750661	+	chr11	108585748	+	N	Y	N	N	N	Y	del_inss	u	FoSTeS	UTR5	SKA3	-	protein_cod	0	intronic	DDX10	+	protein_cod	0
1266	chr13	97722135	+	chr16	69225316	+	N	N	N	N	N	Y	del_inss	d	FoSTeS	intergenic;ir	snoU13,LINC00456	+,-	snoRNA,linc	12401;102588	intronic;intrc	SNTB2	+	protein_cod	0
1267	chr13	63637902	+	chr7	57606290	+	Y	Y	N	N	Y	Y	del_inss	u	FoSTeS	intergenic;ir	LINC00448,LINC0037	-,-	lincRNA,linc	331410;119332	intergenic;ir	RP11-1324A7.2,NCOR1F	+,+	pseudogene	60689;53210
1268	chr14	77278458	+	chr15	87836923	+	N	N	N	Y	N	N	del_inss	d	FoSTeS	intronic;intrc	ANGEL1	-	protein_cod	0	intergenic;ir	RP11-138H10.2,RP11-64	+,-	lincRNA,linc	246531;138366
1269	chr14	77736165	+	chr15	98346707	+	N	N	N	N	N	Y	del_inss	d	FoSTeS	intronic	NGB	-	protein_cod	0	intronic	LINC00923	-	protein_cod	0
1270	chr14	100993716	+	chr17	76474620	+	N	N	Y	N	N	N	del_inss	u	FoSTeS	intronic	WDR25	+	protein_cod	0	intronic	DNAH17	-	protein_cod	0
1271	chr14	20079324	+	chr19	19632141	+	N	N	N	N	N	Y	del_inss	u	FoSTeS	ncRNA_intr	RP11-597A11.1	+	pseudogene	0	intronic;intrc	NDUFA13,YJEFN3,CTC-	+,+,+	protein_cod	0
1272	chr14	55267157	+	chr2	202453343	+	N	N	N	N	Y	N	del_inss	u	FoSTeS	intergenic	SAMD4A,GCH1	+,-	protein_cod	7124;41569	intronic	ALS2CR11	-	protein_cod	0
1273	chr14	55267238	+	chr2	202453988	+	N	N	N	N	N	Y	del_inss	u	FoSTeS	intergenic	SAMD4A,GCH1	+,-	protein_cod	7205;41488	intronic	ALS2CR11	-	protein_cod	0
1274	chr14	55267240	+	chr2	202454031	+	Y	N	N	N	N	N	del_inss	u	FoSTeS	intergenic	SAMD4A,GCH1	+,-	protein_cod	7207;41486	intronic	ALS2CR11	-	protein_cod	0
1275	chr14	55267453	+	chr2	202454315	+	N	Y	Y	Y	N	N	del_inss	u	FoSTeS	intergenic	SAMD4A,GCH1	+,-	protein_cod	7420;41273	intronic	ALS2CR11	-	protein_cod	0
1276	chr15	29850882	+	chr14	72263512	+	N	N	Y	Y	N	N	del_inss	u	FoSTeS	intronic	FAM189A1	-	protein_cod	0	intergenic	SIPA1L1,RP6-114E22.1	+,+	protein_cod	55566;51811
1277	chr15	84363716	+	chr22	50775669	+	Y	N	N	N	N	N	del_inss	d	FoSTeS	intronic	ADAMTSL3	+	protein_cod	0	intergenic	DENND6B,PPP6R2	-,+	protein_cod	10180;6064
1278	chr15	91112793	+	chr3	142283122	+	N	N	N	Y	N	N	del_inss	d	FoSTeS	intronic;intrc	CRTC3	+	protein_cod	0	intronic;intrc	ATR	-	protein_cod	0
1279	chr15	91112793	+	chr3	142283122	+	N	Y	N	N	N	N	del_inss	u	FoSTeS	intronic;intrc	CRTC3	+	protein_cod	0	intronic;intrc	ATR	-	protein_cod	0
1280	chr16	32127861	+	chr1	121137800	+	N	Y	N	N	N	N	del_inss	d	FoSTeS	ncRNA_intr	HERC2P4	-	pseudogene	0	ncRNA_intr	RP11-343N15.1	-	lincRNA	0
1281	chr16	33041231	+	chr10	42647088	+	N	N	Y	N	N	N	del_inss	u	FoSTeS	intergenic	RP11-19N8.2,RP11-19	-,-	pseudogene	1873;13699	intergenic	KSR1P1,IGKV1OR10-1	-,+	pseudogene	2091;33699
1282	chr16	33521338	+	chr7	57918040	+	N	N	N	N	N	Y	del_inss	u	FoSTeS	intergenic;ir	BMS1P8,ENPP7P13	-,+	pseudogene	20367;50550	intergenic;ir	RP11-548K12.12,NONE	+,NONE	pseudogene	21288;NONE
1283	chr16	33955750	+	chrY	10021293	+	N	N	N	N	N	Y	del_inss	d	FoSTeS	intergenic	AC136932.2,LINC0027	-,-	miRNA,lincF	9729;5302	intergenic	PCMTD1P1,CDC27P2	+,-	pseudogene	9477;6693
1284	chr17	41464006	+	chr12	96071648	+	N	Y	N	N	N	N	del_inss	u	FoSTeS	ncRNA_exo	LINC00910	-	lincRNA	0	intronic	NTN4	-	protein_cod	0
1285	chr17	25274659	+	chr7	61666149	+	N	N	Y	N	N	N	del_inss	u	FoSTeS	intergenic;ir	NONE,RP11-260A9.1	NONE,+	NONE,pseu	NONE;34061	intergenic;ir	NONE,RP11-715L17.1	NONE,+	NONE,pseu	NONE;155720
1286	chr17	25290065	+	chr7	65112570	+	N	N	N	N	N	Y	del_inss	u	FoSTeS	intergenic	NONE,RP11-260A9.1	NONE,+	NONE,pseu	NONE;18655	exonic	AC104057.1	-	protein_cod	0
1287	chr17	45209279	+	chr9	37062206	+	N	Y	N	N	N	N	del_inss	d	FoSTeS	intronic	CDC27	-	protein_cod	0	intergenic	RP11-297B17.2,RP11-46	+,-	pseudogene	14972;11322
1288	chr18	46604182	+	chr10	28938628	+	N	Y	N	N	N	N	del_inss	u	FoSTeS	intronic	DYM	-	protein_cod	0	intergenic	WAC,RNU4ATAC6P	+,+	protein_cod	26587;2931
1289	chr18	77301709	+	chr5	177696382	+	N	N	Y	N	N	N	del_inss	d	FoSTeS	intergenic	NFATC1,RP11-567M16	+,+	protein_cod	12384;34751	intronic	COL23A1	-	protein_cod	0
1290	chr19	11422016	+	chr1	38241383	+	N	Y	N	N	N	N	del_inss	u	FoSTeS	ncRNA_intr	CTC-510F12.4	-	3prime_ove	0	upstream	ACTN4P2	+	pseudogene	0
1291	chr19	31150120	+	chr14	34516674	+	N	N	N	N	N	Y	del_inss	d	FoSTeS	intronic	ZNF536	+	protein_cod	0	ncRNA_intr	EGLN3-AS1	+	antisense	0
1292	chr2	194083178	+	chr12	28226413	+	N	Y	N	Y	Y	N	del_inss	u	FoSTeS	intergenic	PCGEM1,AC092638.1	+,+	lincRNA,pse	441553;38030	intergenic	PTHLH,CCDC91	-,+	protein_cod	100775;59769
1293	chr2	202454315	+	chr14	55267453	+	Y	N	N	N	Y	N	del_inss	d	FoSTeS	intronic	ALS2CR11	-	protein_cod	0	intergenic	SAMD4A,GCH1	+,-	protein_cod	7420;41273
1294	chr2	202454409	+	chr14	55267514	+	N	Y	Y	Y	N	Y	del_inss	d	FoSTeS	intronic	ALS2CR11	-	protein_cod	0	intergenic	SAMD4A,GCH1	+,-	protein_cod	7481;41212
1295	chr2	190801557	+	chr15	78892087	+	N	N	Y	Y	N	N	del_inss	u	I_SINE_AluS	intergenic	AC019178.2,MSTN	-,-	pseudogene	5821;118866	intronic	CHRNA3	-	protein_cod	0
1296	chr2	219874443	+	chr20	47078889	+	N	N	N	Y	N	N	del_inss	d	FoSTeS	ncRNA_intr	AC097468.4	+	antisense	0	intergenic	RP1-66N13.1,RNU7-144	-,-	lincRNA,snF	65251;43591
1297	chr2	104694441	+	chr7	135542293	+	N	N	N	N	Y	N	del_inss	u	FoSTeS	downstream	RP11-76I14.1	+	lincRNA	0	intergenic	AC091736.1,AC015987.1	-,+	miRNA,antis	59436;68910
1298	chr20	34272355	+	chr10	34808792	+	N	N	N	N	N	Y	del_inss	d	FoSTeS	intronic	NFS1	-	protein_cod	0	intronic	PARD3	-	protein_cod	0
1299	chr20	26189652	+	chr2	133015264	+	N	N	N	Y	N	N	del_inss	u	FoSTeS	ncRNA_exo	MIR663A	-	processed_	0	upstream	MIR663B	-	miRNA	0
1300	chr20	13914969	+	chr5	132961586	+	N	N	N	Y	N	N	del_inss	u	FoSTeS	intronic	SEL1L2	-	protein_cod	0	ncRNA_exo	AC005195.1	-	miRNA	0
1301	chr21	11097576	+	chr7	152099091	+	N	N	N	N	Y	Y	del_inss	d	FoSTeS	ncRNA_exo	BAGE2	-	pseudogene	0	intronic	KMT2C	-	protein_cod	0
1302	chr21	44757304	+	chr7	44294745	+	N	Y	N	Y	N	N	del_inss	d	FoSTeS	intergenic;ir	LINC00322,AP001046	-,-	lincRNA,linc	5385;20723	intronic	CAMK2B	-	protein_cod	0
1303	chr21	44757304	+	chr7	44294747	+	N	N	Y	N	N	N	del_inss	d	FoSTeS	intergenic;ir	LINC00322,AP001046	-,-	lincRNA,linc	5385;20723	intronic	CAMK2B	-	protein_cod	0
1304	chr21	14603018	+	chr9	34904841	+	N	Y	N	N	N	N	del_inss	d	FoSTeS	intergenic	AJ239321.3,FGF7P2	+,+	pseudogene	109890;118568	intergenic	FAM205CP,GLULP4	-,+	pseudogene	9066;12331
1305	chr22	38506071	+	chr4	2245662	+	N	N	Y	N	N	N	del_inss	u	FoSTeS	intronic	BAIAP2L2	-	protein_cod	0	intergenic	HAUS3,MXD4	-,-	protein_cod	1771;3497
1306	chr22	48664958	+	chr5	102351002	+	N	N	N	N	Y	N	del_inss	d	FoSTeS	intergenic	LL22NC03-121E8.3,MI	+,+	lincRNA,miF	125912;5218	intronic	PAM	+	protein_cod	0
1307	chr3	84433394	+	chr15	71021994	+	N	N	N	Y	N	N	del_inss	u	FoSTeS	intergenic	AC108696.1,AC10702	-,+	miRNA,miR	93990;112333	intronic	UACA	-	protein_cod	0
1308	chr3	25939094	+	chr16	19332992	+	N	N	Y	N	N	N	del_inss	u	FoSTeS	intergenic	LINC00692,RPEP2	-,+	protein_cod	23905;118066	intronic	CLEC19A	+	protein_cod	0
1309	chr3	23128954	+	chr2	69921696	+	N	N	N	N	N	Y	del_inss	u	FoSTeS	intergenic	SALL4P5,RPL24P7	+,+	pseudogene	95881;46909	intergenic	snoU13,AC092431.3	+,-	snoRNA,linc	27103;5628
1310	chr3	117895756	+	chr4	188614276	+	N	N	Y	N	N	N	del_inss	d	FoSTeS	ncRNA_intr	RP11-384F7.1	-	lincRNA	0	intergenic;ir	RP11-565A3.2,ADAM20F	-,-	lincRNA,pse	20481;52972
1311	chr3	2747350	+	chr7	113682819	+	N	N	N	N	N	Y	del_inss	u	FoSTeS	intronic	CNTN4	+	protein_cod	0	intronic	PPP1R3A	-	protein_cod	0
1312	chr3	16409885	+	chrY	7588542	+	N	Y	N	N	N	N	del_inss	d	FoSTeS	intronic;intrc	RFTN1	-	protein_cod	0	ncRNA_intr	RFTN1P1	-	pseudogene	0
1313	chr4	20887010	+	chr17	33007470	+	Y	N	N	Y	N	N	del_inss	u	FoSTeS	intronic	KCNIP4	-	protein_cod	0	intergenic	TMEM132E,RP11-212E8	+,-	protein_cod	41133;45058
1314	chr4	95990463	+	chr8	5224791	+	N	N	Y	N	N	N	del_inss	u	FoSTeS	intronic;intrc	BMPR1B	+	protein_cod	0	intergenic;ir	RN7SL318P,RP11-745K9	-,-	misc_RNA,p	224562;87949
1315	chr4	191027638	+	chr8	146253372	+	N	N	Y	N	N	N	del_inss	u	FoSTeS	intergenic	DUX4L2,NONE	+,NONE	protein_cod	13919;NONE	intergenic	ZNF252P-AS1,AC13910	+,+	antisense,ps	21940;19874
1316	chr4	191027811	+	chr8	146253477	+	N	N	N	Y	N	N	del_inss	u	FoSTeS	intergenic;ir	DUX4L2,NONE	+,NONE	protein_cod	14092;NONE	intergenic;ir	ZNF252P-AS1,AC13910	+,+	antisense,ps	22045;19769
1317	chr4	171599205	+	chr9	109550983	+	N	N	Y	N	N	N	del_inss	d	FoSTeS	intergenic	HSP90AA6P,RP11-322	-,-	pseudogene	72604;64415	intergenic	RP11-308N19.5,RNA5SF	+,-	pseudogene	65772;55047
1318	chr5	102350936	+	chr22	48664661	+	N	N	N	N	Y	N	del_inss	u	FoSTeS	intronic	PAM	+	protein_cod	0	intergenic	LL22NC03-121E8.3,MIR	+,+	lincRNA,miF	125615;5515
1319	chr5	141379670	+	chr3	105828862	+	Y	N	N	N	N	N	del_inss	d	FoSTeS	intronic	GNPDA1	-	protein_cod	0	intergenic	CBLB,RP11-93B21.1	-,-	protein_cod	240466;215722
1320	chr5	13827596	+	chr6	58204340	+	N	N	N	Y	N	N	del_inss	u	FoSTeS	intronic;intrc	DNAH5	-	protein_cod	0	intergenic;ir	XXbac-BPGBPG55C20.1	-,-	lincRNA,linc	21594;24547
1321	chr5	110286657	+	chr6	136610828	+	N	N	N	Y	N	Y	del_inss	d	FoSTeS	upstream	CTD-2089O24.1	-	pseudogene	0	intronic	BCLAF1	-	protein_cod	0
1322	chr6	139666818	+	chr11	56474806	+	N	N	N	N	N	Y	del_inss	d	FoSTeS	intergenic	RP11-12A2.1,CITED2	+,-	pseudogene	6101;26575	intergenic	OR9G1,RP11-100N3.2	+,-	protein_cod	6025;28986
1323	chr6	139666825	+	chr11	56474873	+	N	N	N	Y	N	N	del_inss	d	FoSTeS	intergenic	RP11-12A2.1,CITED2	+,-	pseudogene	6108;26568	intergenic	OR9G1,RP11-100N3.2	+,-	protein_cod	6092;28919
1324	chr6	136582615	+	chr5	110284081	+	N	N	N	Y	N	N	del_inss	d	FoSTeS	exonic;exon	BCLAF1	-	protein_cod	0	ncRNA_exo	CTD-2089O24.1	-	pseudogene	0
1325	chr6	136599863	+	chr5	110286326	+	N	N	N	Y	N	Y	del_inss	u	FoSTeS	exonic	BCLAF1	-	protein_cod	0	ncRNA_exo	CTD-2089O24.1	-	pseudogene	0
1326	chr6	160640072	+	chrX	106846427	+	Y	N	N	N	N	N	del_inss	u	FoSTeS	intronic	SLC22A2	-	protein_cod	0	exonic	FRMPD3	+	protein_cod	0
1327	chr7	56403050	+	chr1	93321960	+	N	N	Y	N	N	N	del_inss	d	FoSTeS	intergenic	RP11-814E24.1,RNU6	+,-	pseudogene	11756;4874	intronic	FAM69A	-	protein_cod	0
1328	chr7	157092418	+	chr13	114425994	+	Y	N	N	N	N	N	del_inss	d	FoSTeS	intergenic	RP5-1142J19.1,RN7SL	-,+	lincRNA,mis	17038;6069	intronic	GRK1	+	protein_cod	0
1329	chr7	157092547	+	chr13	114426331	+	N	N	N	Y	N	N	del_inss	d	FoSTeS	intergenic	RP5-1142J19.1,RN7SL	-,+	lincRNA,mis	17167;5940	intronic	GRK1	+	protein_cod	0
1330	chr7	57918474	+	chr16	33522124	+	N	N	N	N	N	Y	del_inss	d	FoSTeS	intergenic	RP11-548K12.12,NON	+,NONE	pseudogene	21722;NONE	intergenic	BMS1P8,ENPP7P13	-,+	pseudogene	21153;49764
1331	chr7	65112553	+	chr17	25290259	+	N	N	Y	N	N	N	del_inss	d	FoSTeS	exonic	AC104057.1	-	protein_cod	0	intergenic	NONE,RP11-260A9.1	NONE,+	NONE,pseu	NONE;18461
1332	chr7	65112743	+	chr17	25290221	+	N	N	N	N	N	Y	del_inss	d	FoSTeS	exonic	AC104057.1	-	protein_cod	0	intergenic	NONE,RP11-260A9.1	NONE,+	NONE,pseu	NONE;18499
1333	chr7	135542558	+	chr2	104694503	+	N	N	N	N	Y	N	del_inss	d	FoSTeS	intergenic	AC091736.1,AC01598	-,+	miRNA,antis	59701;68645	downstream	RP11-76I14.1	+	lincRNA	0
1334	chr7	152085948	+	chr21	11092581	+	N	N	N	N	Y	Y	del_inss	u	FoSTeS	intronic	KMT2C	-	protein_cod	0	ncRNA_intr	BAGE2	-	pseudogene	0
1335	chr7	63818268	+	chr8	47706613	+	N	Y	N	N	N	N	del_inss	d	FoSTeS	intergenic	TRIM60P18,YWHAEP	+,+	pseudogene	1691;75940	intergenic	TRIM60P15,LINC00293	+,+	pseudogene	1927;27246
1336	chr7	109087830	+	chr8	103643985	+	N	N	N	N	N	Y	del_inss	d	FoSTeS	intergenic	AC002386.1,AC07307	-,-	lincRNA,linc	121458;74208	intergenic	POU5F1P2,KLF10	-,-	pseudogene	9426;17022
1337	chr8	144263502	+	chr10	50463098	+	N	N	N	Y	N	N	del_inss	d	FoSTeS	intergenic	LY6H,GPIHBP1	-,+	protein_cod	21374;31566	intergenic	C10orf128,C10orf71-AS1	-,-	protein_cod	66662;41059
1338	chr8	144263611	+	chr10	50463022	+	N	N	N	N	N	Y	del_inss	d	FoSTeS	intergenic	LY6H,GPIHBP1	-,+	protein_cod	21483;31457	intergenic	C10orf128,C10orf71-AS1	-,-	protein_cod	66586;41135

1339	chr8	14346419	+	chr12	42477173	+	Y	Y	N	N	N	N	del_inss	d	FoSTeS	intronic	SGCZ	-	protein_cod	0	UTR3	GXYLT1	-	protein_cod	0
1340	chr8	68316461	+	chr12	79270616	+	N	N	N	N	Y	N	del_inss	u	FoSTeS	intergenic	RP11-7F18.2,RP11-7F	+,+	lincRNA,pse	59139;2289	intronic	SYT1	+	protein_cod	0
1341	chr8	142837554	+	chr12	131879862	+	N	Y	N	N	N	N	del_inss	d	FoSTeS	intergenic	AC138647.1,AC10441	+,+	protein_cod	308717;189306	intergenic	RP13-507P19.1,RP11-89	+,-	lincRNA,linc	24258;73994
1342	chr8	146253477	+	chr4	191027811	+	N	N	Y	N	N	N	del_inss	d	FoSTeS	intergenic	ZNF252P-AS1,AC139'	+,+	antisense,p:	22045;19769	intergenic	DUX4L2,NONE	+,NONE	protein_cod	14092;NONE
1343	chr8	146300855	+	chr4	191043760	+	N	N	N	Y	N	N	del_inss	d	FoSTeS	intergenic	C8orf33,NONE	+,NONE	protein_cod	19439;NONE	intergenic	DUX4L2,NONE	+,NONE	protein_cod	30041;NONE
1344	chr8	101715287	+	chrX	73802880	+	N	N	N	Y	N	N	del_inss	d	FoSTeS	UTR3	PABPC1	-	protein_cod	0	downstream	PABPC1P3	-	pseudogene	0
1345	chr9	23242910	+	chr11	71274440	+	N	N	N	N	N	Y	del_inss	u	FoSTeS	intergenic	AL391117.1,SUMO2P2	-,+	miRNA,pset	289808;389194	intergenic	KRTAP5-9,KRTAP5-10	+,+	protein_cod	13787;2169
1346	chr9	34904399	+	chr21	14602603	+	N	Y	N	N	N	N	del_inss	u	FoSTeS	intergenic	FAM205CP,GLULP4	-,+	pseudogene	8624;12773	intergenic	AJ239321.3,FGF7P2	+,+	pseudogene	109475;118983
1347	chr9	109550840	+	chr4	171599033	+	N	N	Y	N	N	N	del_inss	u	FoSTeS	intergenic	RP11-308N19.5,RNA5	+,-	pseudogene	65629;55190	intergenic	HSP90AA6P,RP11-322J2	-,-	pseudogene	72432;64587
1348	chr9	66457946	+	chrY	9930325	+	N	Y	N	N	N	N	del_inss	u	FoSTeS	ncRNA_exc	RP11-262H14.1	+	lincRNA	0	downstream	RNA5SP519	-	rRNA	0
1349	chrX	133182350	+	chr1	164989751	+	N	Y	N	N	N	N	del_inss	d	FoSTeS	intergenic	AF003529.2,Y_RNA	+,-	pseudogene	58237;93908	intergenic	AL390119.1,SNORD112	-,-	miRNA,snol	36522;51959
1350	chrX	10159335	+	chr11	63660830	+	N	N	N	N	N	Y	del_inss	d	FoSTeS	intronic	CLCN4	+	protein_cod	0	intronic;intro	MARK2	+	protein_cod	0
1351	chrX	26050060	+	chr14	94846161	+	Y	N	N	N	N	N	del_inss	d	FoSTeS	intergenic	RP11-86A5.1,MAGEB'	+,+	lincRNA,pro	138399;106400	intronic	SERPINA1	-	protein_cod	0
1352	chrX	108796416	+	chr6	160656534	+	Y	N	N	N	N	N	del_inss	d	FoSTeS	intergenic	NXT2,RP1-136J15.5	+,+	protein_cod	8497;15291	intronic	SLC22A2	-	protein_cod	0
1353	chrY	13204402	+	chr11	70835639	+	N	N	N	N	N	Y	del_inss	d	FoSTeS	intergenic	NONE,RP1-85D24.3	NONE,-	NONE,pseu	NONE;58339	intronic	SHANK2	-	protein_cod	0
1354	chrY	10019433	+	chr16	33953007	+	N	N	N	N	N	Y	del_inss	u	FoSTeS	intergenic	PCMTD1P1,CDC27P2	+,-	pseudogene	7617;8553	intergenic	AC136932.2,LINC00273	-,-	miRNA,lincl	6986;8045
1355	chrY	7588505	+	chr3	16406533	+	N	Y	N	N	N	N	del_inss	u	FoSTeS	ncRNA_intr	RFTN1P1	-	pseudogene	0	intronic	RFTN1	-	protein_cod	0
1356	chrY	9930499	+	chr9	66458213	+	N	Y	N	N	N	N	del_inss	d	FoSTeS	ncRNA_exc	RNA5SP519	-	rRNA	0	ncRNA_exc	RNA5SP283	-	rRNA	0
1357	chr1	21019524	+	chr1	147391975	+	N	N	Y	N	N	N	del_inssd	u	FoSTeS	ncRNA_intr	RP5-930J4.5	-	pseudogene	0	intergenic	GJA8,GPR89B	+,+	protein_cod	10582;8531
1358	chr1	24997299	+	chr1	24994642	+	N	N	Y	N	N	N	del_inssd	d	FoSTeS	intronic;intro	SRRM1	+	protein_cod	0	intronic;intro	SRRM1	+	protein_cod	0
1359	chr10	38467917	+	chr10	51752858	+	N	N	N	N	N	Y	del_inssd	u	FoSTeS	ncRNA_intr	RP11-508N22.8	+	processed_	0	intronic	AGAP6	+	protein_cod	0
1360	chr11	18268098	+	chr11	31666353	+	Y	N	N	N	N	N	del_inssd	u	FoSTeS	ncRNA_intr	SAA2-SAA4	-	processed_	0	ncRNA_intr	Z83001.1	-	antisense	0
1361	chr11	18355029	+	chr11	31802856	+	Y	N	N	N	N	N	del_inssd	u	FoSTeS	intronic	GTF2H1	+	protein_cod	0	intronic	ELP4	+	protein_cod	0
1362	chr11	31803181	+	chr11	18749951	+	Y	N	N	N	N	N	del_inssd	d	FoSTeS	intronic	ELP4	+	protein_cod	0	ncRNA_intr	RP11-1081L13.4	+	antisense	0
1363	chr11	48609308	+	chr11	55241409	+	N	N	N	N	Y	N	del_inssd	u	FoSTeS	intergenic;ir	OR4A45P,OR4A41P	+,+	pseudogene	7420;2004	intergenic;ir	OR4A13P,OR4A50P	+,+	pseudogene	6221;2010
1364	chr11	55241495	+	chr11	48609331	+	N	N	N	N	Y	N	del_inssd	d	FoSTeS	intergenic	OR4A13P,OR4A50P	+,+	pseudogene	6307;1924	intergenic;ir	OR4A45P,OR4A41P	+,+	pseudogene	7443;1981
1365	chr11	133223792	+	chr11	119518986	+	N	N	N	Y	N	N	del_inssd	d	FoSTeS	intronic	OPCML	-	protein_cod	0	ncRNA_intr	RP11-196E1.3	+	antisense	0
1366	chr11	133223936	+	chr11	119518961	+	Y	N	N	N	N	N	del_inssd	d	FoSTeS	intronic	OPCML	-	protein_cod	0	ncRNA_intr	RP11-196E1.3	+	antisense	0
1367	chr11	133223941	+	chr11	119519011	+	N	N	Y	N	N	N	del_inssd	d	FoSTeS	intronic	OPCML	-	protein_cod	0	ncRNA_intr	RP11-196E1.3	+	antisense	0
1368	chr11	133223981	+	chr11	119518919	+	N	N	N	N	N	Y	del_inssd	d	FoSTeS	intronic	OPCML	-	protein_cod	0	ncRNA_intr	RP11-196E1.3	+	antisense	0
1369	chr11	133223997	+	chr11	119519034	+	N	N	N	N	Y	N	del_inssd	d	FoSTeS	intronic	OPCML	-	protein_cod	0	ncRNA_intr	RP11-196E1.3	+	antisense	0
1370	chr11	133224219	+	chr11	119519052	+	N	Y	N	N	N	N	del_inssd	d	FoSTeS	intronic	OPCML	-	protein_cod	0	ncRNA_intr	RP11-196E1.3	+	antisense	0
1371	chr12	9559054	+	chr12	31271218	+	N	N	N	Y	N	N	del_inssd	u	FoSTeS	ncRNA_intr	RP11-599J14.2	-	pseudogene	0	ncRNA_intr	RP11-551L14.1	-	pseudogene	0
1372	chr12	11178341	+	chr12	11233957	+	N	N	N	N	N	Y	del_inssd	u	FoSTeS	intergenic	TAS2R19,TAS2R31	-,-	protein_cod	3122;4645	intergenic	TAS2R64P,TAS2R43	-,-	pseudogene	2187;9929
1373	chr12	11178538	+	chr12	11205497	+	N	Y	N	N	N	N	del_inssd	u	FoSTeS	intergenic;ir	TAS2R19,TAS2R31	-,-	protein_cod	3319;4448	intergenic;ir	TAS2R63P,TAS2R46	-,-	pseudogene	3642;8467
1374	chr12	11284305	+	chr12	11181660	+	N	N	N	N	N	Y	del_inssd	d	FoSTeS	intergenic;ir	RP11-673D15.7,TAS2F	-,-	pseudogene	34432;1252	intergenic	TAS2R19,TAS2R31	-,-	protein_cod	6441;1326
1375	chr12	31272494	+	chr12	9559639	+	N	N	N	Y	N	N	del_inssd	d	FoSTeS	ncRNA_exc	RP11-551L14.1	-	pseudogene	0	ncRNA_intr	RP11-599J14.2	-	pseudogene	0
1376	chr12	59935795	+	chr12	59952861	+	Y	Y	Y	Y	Y	Y	del_inssd	u	FoSTeS	intergenic	AC108721.1,RNU4-20	-,-	miRNA,snR	13746;44779	intergenic	AC108721.1,RNU4-20P	-,-	miRNA,snR	30812;27713
1377	chr12	59952987	+	chr12	59946633	+	Y	Y	Y	Y	Y	Y	del_inssd	d	FoSTeS	intergenic	AC108721.1,RNU4-20	-,-	miRNA,snR	30938;27587	intergenic	AC108721.1,RNU4-20P	-,-	miRNA,snR	24584;33941
1378	chr13	36531242	+	chr13	36532169	+	N	N	N	N	Y	N	del_inssd	u	FoSTeS	intronic	DCLK1	-	protein_cod	0	intronic	DCLK1	-	protein_cod	0
1379	chr13	78508913	+	chr13	84484043	+	N	Y	N	N	N	N	del_inssd	u	_LTR_HERVI	ncRNA_intr	RNF219-AS1	+	antisense	0	intergenic	VENTXP2,UBE2D3P4	-,+	pseudogene	2127;110419
1380	chr13	84484521	+	chr13	78509327	+	N	Y	N	N	N	N	del_inssd	d	_LTR_HERVI	intergenic	VENTXP2,UBE2D3P4	-,+	pseudogene	2605;109941	ncRNA_intr	RNF219-AS1	+	antisense	0
1381	chr15	79220652	+	chr15	79233878	+	N	N	N	Y	N	N	del_inssd	u	FoSTeS	intronic;intro	CTSH	-	protein_cod	0	intronic;intro	CTSH	-	protein_cod	0
1382	chr15	91981578	+	chr15	91989267	+	Y	Y	Y	Y	Y	Y	del_inssd	u	FoSTeS	ncRNA_intr	RP11-661P17.1	+	lincRNA	0	ncRNA_intr	RP11-661P17.1	+	lincRNA	0
1383	chr15	91989617	+	chr15	91983461	+	Y	Y	Y	Y	Y	Y	del_inssd	d	FoSTeS	ncRNA_intr	RP11-661P17.1	+	lincRNA	0	ncRNA_intr	RP11-661P17.1	+	lincRNA	0
1384	chr19	605299	+	chr19	608434	+	N	N	Y	N	N	N	del_inssd	u	FoSTeS	intronic;intro	HCN2	+	protein_cod	0	intronic;intro	HCN2	+	protein_cod	0
1385	chr19	608624	+	chr19	605316	+	N	N	Y	Y	N	N	del_inssd	d	FoSTeS	intronic	HCN2	+	protein_cod	0	intronic	HCN2	+	protein_cod	0
1386	chr19	53936267	+	chr19	53973204	+	N	N	Y	N	N	N	del_inssd	u	FoSTeS	ncRNA_intr	ZNF761,TPM3P9	+,+	processed_	0	intronic;intro	ZNF813	+	protein_cod	0
1387	chr19	53973485	+	chr19	53936696	+	N	N	Y	N	N	N	del_inssd	d	FoSTeS	intronic	ZNF813	+	protein_cod	0	ncRNA_intr	ZNF761,TPM3P9	+,+	processed_	0
1388	chr2	42964157	+	chr2	214171095	+	N	N	N	N	Y	N	del_inssd	u	FoSTeS	intronic	MTA3	+	protein_cod	0	intronic;intro	SPAG16	+	protein_cod	0
1389	chr22	25692175	+	chr22	25988830	+	N	Y	N	N	N	N	del_inssd	u	FoSTeS	intergenic	RP3-462D8.2,IGLL3P	-,+	lincRNA,pse	13534;22048	intronic	ADRBK2	+	protein_cod	0
1390	chr22	25988947	+	chr22	25692343	+	N	Y	N	N	N	N	del_inssd	d	FoSTeS	intronic	ADRBK2	+	protein_cod	0	intergenic	RP3-462D8.2,IGLL3P	-,+	lincRNA,pse	13702;21880
1391	chr3	95465766	+	chr3	95470766	+	Y	Y	Y	Y	Y	Y	del_inssd	u	FoSTeS	intergenic	MTHFD2P1,AC10873(	-,-	pseudogene	63729;419895	intergenic	MTHFD2P1,AC108739.1	-,-	pseudogene	68729;414895
1392	chr3	95471000	+	chr3	95468117	+	Y	Y	Y	Y	Y	Y	del_inssd	d	FoSTeS	intergenic	MTHFD2P1,AC10873(	-,-	pseudogene	68963;414661	intergenic	MTHFD2P1,AC108739.1	-,-	pseudogene	66080;417544
1393	chr3	146385189	+	chr3	146394863	+	Y	Y	Y	Y	Y	Y	del_inssd	u	FoSTeS	intergenic	PLSCR5,RP11-649A1(	-,-	protein_cod	61186;242283	intergenic	PLSCR5,RP11-649A16.1	-,-	protein_cod	70860;232609
1394	chr3	146395165	+	chr3	146390405	+	Y	Y	Y	Y	Y	Y	del_inssd	d	FoSTeS	intergenic	PLSCR5,RP11-649A1(	-,-	protein_cod	71162;232307	intergenic	PLSCR5,RP11-649A16.1	-,-	protein_cod	66402;237067
1395	chr3	194543292	+	chr3	194546259	+	Y	Y	Y	Y	Y	Y	del_inssd	u	FoSTeS	intergenic	AC090505.6,AC09050	+,+	lincRNA,linc	40395;5327	intergenic	AC090505.6,AC090505.'	+,+	lincRNA,linc	43362;2360
1396	chr3	194546432	+	chr3	194543309	+	Y	Y	Y	Y	Y	Y	del_inssd	d	FoSTeS	intergenic	AC090505.6,AC09050	+,+	lincRNA,linc	43535;2187	intergenic	AC090505.6,AC090505.'	+,+	lincRNA,linc	40412;5310
1397	chr7	63205312	+	chr7	65303812	+	N	N	N	N	N	Y	del_inssd	u	FoSTeS	intergenic	CTD-2526L21.2,CICP2	+,-	pseudogene	44493;23323	intergenic	RNU6-912P,RP13-254B1	+,+	snRNA,pset	24050;1387
1398	chr7	65309770	+	chr7	63238863	+	N	N	N	N	N	Y	del_inssd	d	FoSTeS	intergenic;ir	RP13-254B10.1,RNU6	+,-	pseudogene	4187;14877	intergenic;ir	CICP24,CTD-2526L21.3	-,+	pseudogene	7438;11424
1399	chr7	88516540	+	chr7	88518422	+	Y	N	N	Y	N	N	del_inssd	u	FoSTeS	intronic;intro	ZNF804B	+	protein_cod	0	intronic	ZNF804B	+	protein_cod	0
1400	chr7	88516540	+	chr7	88518949	+	N	N	Y	N	Y	N	del_inssd	u	FoSTeS	intronic;intro	ZNF804B	+	protein_cod	0	intronic	ZNF804B	+	protein_cod	0
1401	chr7	88519179	+	chr7	88516704	+	N	N	Y	N	Y	N	del_inssd	d	FoSTeS	intronic	ZNF804B	+	protein_cod	0	intronic	ZNF804B	+	protein_cod	0
1402	chr7	142012833	+	chr7	142044625	+	N	N	N	N	N	Y	del_inssd	u	FoSTeS	upstream	TRBV4-1	+	TR_V_gene	0	upstream	TRBV4-2	+	TR_V_gene	0
1403	chr7	142045993	+	chr7	142013497	+	N	N	N	N	N	Y	del_inssd	d	FoSTeS	downstream	TRBV4-2	+	TR_V_gene	0	downstream	TRBV4-1	+	TR_V_gene	0
1404	chr7	142143247	+	chr7	142180301	+	Y	N	N	N	N	N	del_inssd	u	FoSTeS	downstream	TRBV6-7	-	TR_V_gene	0	downstream	TRBV6-5	-	TR_V_gene	0
1405	chr7	142180367	+	chr7	142143589	+	Y	N	N	N	N	N	del_inssd	d	FoSTeS	downstream	TRBV6-5	-	TR_V_gene	0	downstream	TRBV6-7	-	TR_V_gene	0
1406	chr8	27777171	+	chr8	135255761	+	N	N	N	N	Y	N	del_inssd	u	FoSTeS	intronic	SCARA5	-	protein_cod	0	ncRNA_intr	RP11-513H8.1	-	lincRNA	0
1407	chr8	58116883	+	chr8	58118300	+	N	N	N	N	N	Y	del_inssd	u	FoSTeS	ncRNA_intr	RP11-513O17.2	+	lincRNA	0	ncRNA_intr	RP11-513O17.2	+	lincRNA	0
1408	chr8	58116904	+	chr8	58118279	+	N	N	Y	N	Y	N	del_inssd	u	FoSTeS	ncRNA_intr	RP11-513O17.2	+	lincRNA	0	ncRNA_intr	RP11-513O17.2	+	lincRNA	0
1409	chr8	58116933	+	chr8	58118309	+	N	N	N	Y	N	N	del_inssd	u	FoSTeS	ncRNA_intr	RP11-513O17.2	+	lincRNA	0	ncRNA_intr	RP11-513O17.2	+	lincRNA	0
1410	chr8	58128150	+	chr8	58118227	+	N	N	Y	Y	Y	Y	del_inssd	d	FoSTeS	ncRNA_intr	RP11-513O17.2	+	lincRNA	0	ncRNA_intr	RP11-513O17.2	+	lincRNA	0
1411	chr9	113024576	+	chr9	113037094	+	Y	Y	Y	Y	Y	Y	del_inssd	u	FoSTeS	intergenic	TXN,TXNDC8	-,-	protein_cod	5656;41225	intergenic	TXN,TXNDC8	-,-	protein_cod	18174;28707
1412	chr9	113037288	+	chr9	113029969	+	Y	Y	Y	Y	Y	Y	del_inssd	d	FoSTeS	intergenic	TXN,TXNDC8	-,-	protein_cod	18368;28513	intergenic	TXN,TXNDC8	-,-	protein_cod	11049;35832
1413	chr1	2587856	+	chr1	2628538	+	N	N	Y	N	N	N	del_inssu	u	FoSTeS	intronic	TTC34	-	protein_cod	0	intronic	TTC34	-	protein_cod	0
1414	chr1	2619074	+	chr1	2625830	+	N	N	Y	N	N	N	del_inssu	d	FoSTeS	intronic	TTC34	-	protein_cod	0	intronic	TTC34	-	protein_cod	0
1415	chr1	2623564	+	chr1	2583063	+	N	N	Y	N	N	N	del_inssu	u	FoSTeS	intronic	TTC34	-	protein_cod	0	intronic	TTC34	-	protein_cod	0
1416	chr1	2623574	+	chr1	2617426	+	N	N	Y	N	N	N	del_inssu	u	FoSTeS	intronic	TTC34	-	protein_cod	0	intronic	TTC34	-	protein_cod	0
1417	chr1	86005699	+	chr1	146486078	+	N	Y	Y	Y	Y	Y	del_inssu	d	FoSTeS	intronic	DDAH1	-	protein_cod	0	intergenic	RP4-704D21.2,NBPF13F	-,-	lincRNA,pse	10901;4817
1418	chr1	120393298	+	chr1	147389638	+	Y	N	N	N	N	N	del_inssu	d	FoSTeS	intergenic;ir	NBPF7,PFN1P9	-,+	pseudogene	5519;2641	intergenic;ir	GJA8,GPR89B	+,+	protein_cod	8245;10868
1419	chr1	144849317	+	chr1	85980122	+	N	Y	N	N	N	N	del_inssu	u	FoSTeS	intergenic	RP4-791M13.3,PDE4D	-,-	antisense,p	13450;2107	intronic	DDAH1	-	protein_cod	0
1420	chr1	144849381	+	chr1	85980164	+	Y	N	Y	Y	Y	Y	del_inssu	u	FoSTeS	intergenic	RP4-791M13.3,PDE4D	-,-	antisense,p	13514;2043	intronic	DDAH1	-	protein_cod	0
1421	chr1	225133580	+	chr1	225249016	+	N	N	N	Y	N	N	del_inssu	d	FoSTeS	intronic	DNAH14	+	protein_cod	0	intronic	DNAH14	+	protein_cod	0
1422	chr1	225133653	+	chr1	225248466	+	N	N	N	N	N	Y	del_inssu	d	FoSTeS	intronic	DNAH14	+	protein_cod	0	intronic	DNAH14	+	protein_cod	0

1423	chr1	225248160	+	chr1	225133123	+	N	Y	N	N	N	N	del_inssu	u	FoSTeS	intronic	DNAH14	+	protein_cod	0	intronic	DNAH14	+	protein_cod	0
1424	chr1	225248398	+	chr1	225133380	+	N	N	N	Y	N	Y	del_inssu	u	FoSTeS	intronic	DNAH14	+	protein_cod	0	intronic	DNAH14	+	protein_cod	0
1425	chr10	38285872	+	chr10	78607612	+	N	N	N	N	Y	N	del_inssu	d	FoSTeS	intergenic	Y_RNA,ZNF33A	+,+	misc_RNA,p	1969;13706	intergenic	ATP5G1P8,KCNMA1	+,-	pseudogene	10993;21747
1426	chr10	38816527	+	chr10	38818875	+	Y	N	N	N	N	N	del_inssu	d	FoSTeS	intergenic;ir	RP11-291L22.4,ABCD	+,+	lincRNA,pse	61423;78022	intergenic;ir	RP11-291L22.4,ABCD1P	+,+	lincRNA,pse	63771;75674
1427	chr10	38818664	+	chr10	38815181	+	Y	N	N	N	N	N	del_inssu	u	FoSTeS	intergenic	RP11-291L22.4,ABCD	+,+	lincRNA,pse	63560;75885	intergenic	RP11-291L22.4,ABCD1P	+,+	lincRNA,pse	60077;79368
1428	chr10	76654961	+	chr10	19211100	+	N	N	N	N	Y	N	del_inssu	u	FoSTeS	intronic;intrc	KAT6B	+	protein_cod	0	intergenic;ir	RP11-139J15.5,RP11-28	+,+	lincRNA,pse	174318;18330
1429	chr11	9644770	+	chr11	10154921	+	N	Y	N	N	Y	N	del_inssu	d	FoSTeS	intergenic;ir	RP11-16F15.1,RP11-1	+,-	pseudogene	16614;7785	intronic;intrc	SBF2	-	protein_cod	0
1430	chr11	10154081	+	chr11	9643842	+	N	N	N	N	Y	N	del_inssu	u	FoSTeS	intronic	SBF2	-	protein_cod	0	intergenic	RP11-16F15.1,RP11-16F	+,-	pseudogene	15686;8713
1431	chr11	10154155	+	chr11	9643771	+	N	Y	N	N	N	N	del_inssu	u	FoSTeS	intronic	SBF2	-	protein_cod	0	intergenic	RP11-16F15.1,RP11-16F	+,-	pseudogene	15615;8784
1432	chr11	48545131	+	chr11	48609331	+	N	N	N	Y	N	N	del_inssu	d	FoSTeS	intergenic	OR4A40P,OR4A43P	+,+	pseudogene	10359;2485	intergenic;ir	OR4A45P,OR4A41P	+,+	pseudogene	7443;1981
1433	chr11	48609285	+	chr11	48545017	+	N	N	N	Y	N	N	del_inssu	u	FoSTeS	intergenic	OR4A45P,OR4A41P	+,+	pseudogene	7397;2027	intergenic	OR4A40P,OR4A43P	+,+	pseudogene	10245;2599
1434	chr12	11205575	+	chr12	11276606	+	N	N	N	N	N	Y	del_inssu	d	FoSTeS	intergenic	TAS2R63P,TAS2R46	-,-	pseudogene	3720;8389	intergenic	RP11-673D15.7,TAS2R3	-,-	pseudogene	26733;8951
1435	chr12	11275888	+	chr12	11204715	+	N	N	N	N	N	Y	del_inssu	u	FoSTeS	intergenic;ir	RP11-673D15.7,TAS2F	-,-	pseudogene	26015;9669	intergenic;ir	TAS2R63P,TAS2R46	-,-	pseudogene	2860;9249
1436	chr14	106932641	+	chr14	107174926	+	Y	Y	Y	N	Y	N	del_inssu	d	FoSTeS	intergenic	IGHVII-43-1,IGHVIII-44	-,-	IG_V_pseuc	3753;1585	intergenic	IGHV1-69,IGHV2-70	-,-	IG_V_gene,	4498;3894
1437	chr14	107094692	+	chr14	106804954	+	Y	Y	Y	N	N	N	del_inssu	u	FoSTeS	upstream;dc	IGHVII-60-1,IGHV4-61	-,-	IG_V_pseuc	0	downstream	IGHV4-31	-	IG_V_gene	0
1438	chr14	107094770	+	chr14	106804822	+	N	N	N	N	Y	N	del_inssu	u	FoSTeS	upstream;dc	IGHVII-60-1,IGHV4-61	-,-	IG_V_pseuc	0	downstream	IGHV4-31	-	IG_V_gene	0
1439	chr15	24492714	+	chr15	24805859	+	N	N	N	N	N	Y	del_inssu	d	FoSTeS	ncRNA_intr	RP11-580I1.2	+	lincRNA	0	ncRNA_intr	PWRN1	+	lincRNA	0
1440	chr15	24799349	+	chr15	24486265	+	N	N	N	N	N	Y	del_inssu	u	FoSTeS	ncRNA_intr	PWRN1	+	lincRNA	0	ncRNA_intr	RP11-580I1.2	+	lincRNA	0
1441	chr17	10886860	+	chr17	10895730	+	Y	Y	Y	Y	Y	Y	del_inssu	d	FoSTeS	intergenic	RP11-963H4.3,AC0052	+,+	lincRNA,miF	102648;70245	intergenic	RP11-963H4.3,AC00528	+,+	lincRNA,miF	111518;61375
1442	chr17	10890416	+	chr17	10886446	+	Y	Y	Y	Y	Y	Y	del_inssu	u	FoSTeS	intergenic	RP11-963H4.3,AC0052	+,+	lincRNA,miF	106204;66689	intergenic	RP11-963H4.3,AC00528	+,+	lincRNA,miF	102234;70659
1443	chr19	15473230	+	chr19	58657873	+	N	N	Y	N	Y	N	del_inssu	d	FoSTeS	intronic	AKAP8	-	protein_cod	0	intronic	ZNF329	-	protein_cod	0
1444	chr2	27616460	+	chr2	181738244	+	N	N	N	Y	N	N	del_inssu	d	FoSTeS	intronic	PPM1G	-	protein_cod	0	upstream	FTH1P20	-	pseudogene	0
1445	chr2	90449668	+	chr2	91670354	+	N	N	Y	N	N	N	del_inssu	d	FoSTeS	intergenic	AC113612.1,CH17-132	+,+	pseudogene	149266;8533	intergenic	AC018696.1,IGKV1OR2-	+,+	pseudogene	34205;8733
1446	chr2	181738223	+	chr2	27616320	+	N	N	N	Y	N	N	del_inssu	u	FoSTeS	upstream	FTH1P20	-	pseudogene	0	intronic	PPM1G	-	protein_cod	0
1447	chr2	240564102	+	chr2	240565286	+	N	N	N	Y	N	N	del_inssu	d	FoSTeS	intergenic	AC079612.2,RP11-315	+,+	lincRNA,linc	11569;92548	intergenic	AC079612.2,RP11-315F2	+,+	lincRNA,linc	12753;91364
1448	chr2	240564981	+	chr2	240564049	+	N	N	N	Y	N	N	del_inssu	u	FoSTeS	intergenic	AC079612.2,RP11-315	+,+	lincRNA,linc	12448;91669	intergenic	AC079612.2,RP11-315F2	+,+	lincRNA,linc	11516;92601
1449	chr22	22707485	+	chr22	22730576	+	Y	Y	N	N	N	Y	del_inssu	d	FoSTeS	exonic	IGLV5-48	+	IG_V_gene	0	exonic	IGLV5-45	+	IG_V_gene	0
1450	chr22	22729248	+	chr22	22706445	+	Y	Y	N	N	N	Y	del_inssu	u	FoSTeS	intergenic	LL22NC03-22A12.12,I	+,+	pseudogene	3760;1107	upstream	IGLV5-48	+	IG_V_gene	0
1451	chr22	22731629	+	chr22	22783091	+	N	N	Y	N	N	N	del_inssu	d	FoSTeS	downstream	IGLV5-45	+	IG_V_gene	0	downstream	IGLV5-37	+	IG_V_gene	0
1452	chr22	22752559	+	chr22	22703224	+	N	N	Y	N	N	N	del_inssu	u	FoSTeS	upstream;dc	IGLVVII-41-1,IGLVI-42	+,+	IG_V_pseuc	0	downstream	LL22NC03-75A1.9	+	pseudogene	0
1453	chr4	32065877	+	chr4	32071042	+	Y	Y	Y	Y	N	N	del_inssu	d	FoSTeS	ncRNA_intr	RP11-734I18.1	+	lincRNA	0	ncRNA_intr	RP11-734I18.1	+	lincRNA	0
1454	chr4	32070976	+	chr4	32065773	+	Y	Y	Y	Y	N	N	del_inssu	u	FoSTeS	ncRNA_intr	RP11-734I18.1	+	lincRNA	0	ncRNA_intr	RP11-734I18.1	+	lincRNA	0
1455	chr4	91933840	+	chr4	91936472	+	Y	N	N	N	Y	N	del_inssu	d	FoSTeS	intronic	CCSER1	+	protein_cod	0	intronic	CCSER1	+	protein_cod	0
1456	chr4	91933858	+	chr4	91936481	+	N	N	N	Y	N	Y	del_inssu	d	FoSTeS	intronic;intrc	CCSER1	+	protein_cod	0	intronic	CCSER1	+	protein_cod	0
1457	chr4	91936098	+	chr4	91933643	+	Y	N	N	Y	Y	Y	del_inssu	u	FoSTeS	intronic;intrc	CCSER1	+	protein_cod	0	intronic;intrc	CCSER1	+	protein_cod	0
1458	chr5	151362245	+	chr5	164610022	+	Y	N	Y	Y	Y	Y	del_inssu	d	FoSTeS	ncRNA_intr	CTB-12O2.1	+	lincRNA	0	intergenic	CTC-340A15.2,CTB-181I	+,-	antisense,lir	11373;37561
1459	chr5	154018773	+	chr5	126064717	+	N	N	N	N	Y	N	del_inssu	u	FoSTeS	intergenic	MIR3141,MIR1303	-,+	miRNA,miR	43141;46563	intergenic	RP11-772E11.1,HSPE1P	-,-	pseudogene	65457;8413
1460	chr5	154018784	+	chr5	126065053	+	Y	N	Y	Y	N	Y	del_inssu	u	FoSTeS	intergenic	MIR3141,MIR1303	-,+	miRNA,miR	43152;46552	intergenic	RP11-772E11.1,HSPE1P	-,-	pseudogene	65793;8077
1461	chr6	53924806	+	chr6	53934828	+	Y	Y	Y	Y	Y	Y	del_inssu	d	FoSTeS	ncRNA_intr	MLIP-AS1	-	antisense	0	ncRNA_intr	MLIP-AS1	-	antisense	0
1462	chr6	53928709	+	chr6	53924658	+	Y	Y	Y	Y	Y	Y	del_inssu	u	FoSTeS	ncRNA_intr	MLIP-AS1	-	antisense	0	ncRNA_intr	MLIP-AS1	-	antisense	0
1463	chr7	62518861	+	chr7	55830760	+	N	N	N	N	N	Y	del_inssu	u	FoSTeS	ncRNA_intr	RP11-196D18.2	+	pseudogene	0	intergenic	CICP11,PSPHP1	+,+	pseudogene	23471;1730
1464	chr7	140189221	+	chr7	140181588	+	Y	N	Y	Y	N	N	del_inssu	u	FoSTeS	intergenic;ir	MKRN1,DENND2A	-,-	protein_cod	9852;28999	intergenic;ir	MKRN1,DENND2A	-,-	protein_cod	2219;36632
1465	chr7	142099580	+	chr7	142247245	+	N	N	Y	N	N	N	del_inssu	d	FoSTeS	exonic;exon	TRBV7-8	-	TR_V_gene	0	exonic;exon	TRBV7-3	-	TR_V_gene	0
1466	chr7	142175098	+	chr7	142098243	+	N	N	Y	N	N	N	del_inssu	u	FoSTeS	intergenic	TRBV5-4,TRBV7-4	-,-	TR_V_gene	6053;1231	intergenic	TRBV4-2,TRBV7-8	+,-	TR_V_gene	52427;1212
1467	chr7	142206449	+	chr7	142231481	+	Y	N	N	N	N	N	del_inssu	d	FoSTeS	downstream	TRBV10-2	-	TR_V_gene	0	downstream	TRBV10-1	-	TR_V_gene	0
1468	chr7	142223548	+	chr7	142196395	+	Y	N	N	N	Y	N	del_inssu	u	FoSTeS	downstream	TRBV11-1	-	TR_V_gene	0	intergenic	TRBV12-2,TRBV11-2	-,-	TR_V_pseu	5315;1175
1469	chr8	80136511	+	chr8	80028972	+	N	N	N	N	Y	N	del_inssu	u	FoSTeS	intergenic	AC009941.1,RP11-111	+,+	miRNA,lincF	126823;73441	intergenic	AC009941.1,RP11-1114I	+,+	miRNA,lincF	19284;180980
1470	chr9	93179673	+	chr9	90030026	+	N	N	N	N	N	Y	del_inssu	u	FoSTeS	ncRNA_intr	RP11-389K14.3	-	lincRNA	0	intergenic	SNORA26,RP11-497K15	+,+	snoRNA,ps	154526;8523
1471	chr9	112286045	+	chr9	112286687	+	Y	Y	Y	Y	Y	Y	del_inssu	d	FoSTeS	intergenic	MIR3927,YBX1P6	-,-	miRNA,pseu	12220;9065	intergenic	MIR3927,YBX1P6	-,-	miRNA,pseu	12862;8423
1472	chr9	112286562	+	chr9	112285846	+	Y	Y	Y	Y	Y	Y	del_inssu	u	FoSTeS	intergenic	MIR3927,YBX1P6	-,-	miRNA,pseu	12737;8548	intergenic	MIR3927,YBX1P6	-,-	miRNA,pseu	12021;9264
1473	chrY	28794557	+	chrY	28815990	+	N	Y	N	N	N	N	del_inssu	d	FoSTeS	intergenic	PARP4P1,NONE	-,NONE	pseudogene	13758;NONE	intergenic	PARP4P1,NONE	-,NONE	pseudogene	35191;NONE
1474	chrY	28803184	+	chrY	28787254	+	N	Y	N	N	N	N	del_inssu	u	FoSTeS	intergenic	PARP4P1,NONE	-,NONE	pseudogene	22385;NONE	intergenic	PARP4P1,NONE	-,NONE	pseudogene	6455;NONE
1475	chr1	17051740	-	chr1	234912188	+	N	Y	N	Y	Y	N	del_invers	d	FoSTeS	intergenic	ESPNP,FAM231C	-,-	pseudogene	5088;8707	intergenic	LINC01132,RNY4P16	+,+	lincRNA,mis	44798;61532
1476	chr1	16871850	+	chr1	17054470	-	N	Y	N	Y	Y	N	del_invers	u	FoSTeS	intergenic	FAM231B,RP5-875O1	+,-	protein_cod	5780;3296	intergenic	ESPNP,FAM231C	-,-	pseudogene	7818;5977
1477	chr10	59257658	-	chr10	59257982	+	Y	Y	Y	Y	Y	Y	del_invers	d	FoSTeS	intergenic	MIR3924,RP11-550A9	-,+	miRNA,pseu	193339;15259	intergenic	MIR3924,RP11-550A9.1	-,+	miRNA,pseu	193663;14935
1478	chr10	59256945	+	chr10	59257945	-	Y	Y	Y	Y	Y	Y	del_invers	u	FoSTeS	intergenic	MIR3924,RP11-550A9	-,+	miRNA,pseu	192626;15972	intergenic	MIR3924,RP11-550A9.1	-,+	miRNA,pseu	193626;14972
1479	chr11	55444014	-	chr11	55457753	+	Y	Y	Y	Y	Y	Y	del_invers	d	FoSTeS	intergenic	OR4V1P,OR4P1P	+,+	pseudogene	1863;6701	intergenic	RP11-674C21.9,OR5D2F	-,+	lincRNA,pse	4117;24719
1480	chr11	55365426	+	chr11	55445866	-	Y	Y	Y	Y	Y	Y	del_invers	u	FoSTeS	intergenic	OR4C16,OR4C11	+,-	protein_cod	24890;5404	intergenic	OR4V1P,OR4P1P	+,+	pseudogene	3715;4849
1481	chr12	47030464	-	chr12	47033307	+	Y	Y	Y	Y	Y	Y	del_invers	d	FoSTeS	ncRNA_intr	RP11-446N19.1	+	lincRNA	0	ncRNA_intr	RP11-446N19.1	+	lincRNA	0
1482	chr12	47028575	+	chr12	47030624	-	Y	Y	Y	Y	Y	Y	del_invers	u	FoSTeS	ncRNA_intr	RP11-446N19.1	+	lincRNA	0	ncRNA_intr	RP11-446N19.1	+	lincRNA	0
1483	chr14	65842590	-	chr14	65843805	+	N	N	N	Y	N	N	del_invers	d	FoSTeS	intergenic	MIR4708,FUT8	-,+	miRNA,prot	40689;34720	intergenic	MIR4708,FUT8	-,+	miRNA,prot	41904;33505
1484	chr14	65842845	-	chr14	65843273	+	N	N	N	N	Y	N	del_invers	d	FoSTeS	intergenic	MIR4708,FUT8	-,+	miRNA,prot	40944;34465	intergenic	MIR4708,FUT8	-,+	miRNA,prot	41372;34037
1485	chr14	65842536	+	chr14	65843134	-	N	N	N	Y	Y	N	del_invers	u	FoSTeS	intergenic;ir	MIR4708,FUT8	-,+	miRNA,prot	40635;34774	intergenic;ir	MIR4708,FUT8	-,+	miRNA,prot	41233;34176
1486	chr19	22763388	-	chr19	22763593	+	Y	N	N	N	N	N	del_invers	d	FoSTeS	intergenic	RNU6-1179P,CTC-457	+,-	snRNA,pseu	25219;14893	intergenic	RNU6-1179P,CTC-457E2	+,-	snRNA,pseu	25424;14688
1487	chr19	22762656	+	chr19	22763590	-	Y	N	N	N	N	N	del_invers	u	FoSTeS	intergenic	RNU6-1179P,CTC-457	+,-	snRNA,pseu	24487;15625	intergenic	RNU6-1179P,CTC-457E2	+,-	snRNA,pseu	25421;14691
1488	chr2	225292981	-	chr2	225293952	+	Y	Y	Y	Y	Y	Y	del_invers	d	FoSTeS	intergenic;ir	FAM124B,CUL3	-,-	protein_cod	26179;41886	intergenic;ir	FAM124B,CUL3	-,-	protein_cod	27150;40915
1489	chr2	130818753	+	chr2	132030106	-	N	N	Y	N	N	N	del_invers	u	FoSTeS	intergenic	FAR2P1,POTEF	-,-	pseudogene	10049;12355	intronic	PLEKHB2	+	protein_cod	0
1490	chr2	225292951	+	chr2	225293082	-	Y	Y	Y	Y	Y	Y	del_invers	u	FoSTeS	intergenic	FAM124B,CUL3	-,-	protein_cod	26149;41916	intergenic	FAM124B,CUL3	-,-	protein_cod	26280;41785
1491	chr3	57377841	-	chr3	142471157	+	N	Y	N	N	N	N	del_invers	d	FoSTeS	intronic	DNAH12	-	protein_cod	0	intronic	TRPC1	+	protein_cod	0
1492	chr4	137510197	-	chr4	137552592	+	Y	N	N	N	N	N	del_invers	d	FoSTeS	intergenic	RP11-775H9.2,AC093	-,-	lincRNA,miF	193571;118136	intergenic	RP11-775H9.2,AC09387	-,-	lincRNA,miF	235966;75741
1493	chr4	137509447	+	chr4	137544372	-	Y	N	N	N	N	N	del_invers	u	FoSTeS	intergenic	RP11-775H9.2,AC093	-,-	lincRNA,miF	192821;118886	intergenic	RP11-775H9.2,AC09387	-,-	lincRNA,miF	227746;83961
1494	chr5	170260575	-	chr5	178183082	+	N	N	N	N	Y	N	del_invers	d	FoSTeS	intergenic	GABRP,RANBP17	+,+	protein_cod	19524;28299	intergenic	ZNF354A,AACSP1	-,-	protein_cod	25379;8780
1495	chr7	124662610	-	chr7	147571592	+	N	N	Y	N	N	N	del_invers	d	FoSTeS	ncRNA_intr	POT1-AS1	+	antisense	0	intronic;intrc	CNTNAP2	+	protein_cod	0
1496	chrX	131007721	-	chrX	131008631	+	Y	N	N	Y	Y	Y	del_invers	d	FoSTeS	intergenic	RP11-453F18__B.1,RF	-,+	lincRNA,pse	43050;63977	intergenic	RP11-453F18__B.1,RP1-	-,+	lincRNA,pse	43960;63067
1497	chrX	131005307	+	chrX	131007921	-	Y	Y	N	Y	Y	Y	del_invers	u	FoSTeS	intergenic	RP11-453F18__B.1,RF	-,+	lincRNA,pse	40636;66391	intergenic	RP11-453F18__B.1,RP1-	-,+	lincRNA,pse	43250;63777
1498	chrX	131005423	+	chrX	131007845	-	N	N	Y	N	N	N	del_invers	u	FoSTeS	intergenic	RP11-453F18__B.1,RF	-,+	lincRNA,pse	40752;66275	intergenic	RP11-453F18__B.1,RP1-	-,+	lincRNA,pse	43174;63853
1499	chr1	9121444	+	chr14	93713178	-	Y	Y	Y	Y	Y	Y	inso	u	NA	intronic	SLC2A5	-	protein_cod	0	intronic	BTBD7	-	protein_cod	0
1500	chr1	121137800	-	chr16	32822737	+	Y	N	N	N	N	N	inso	d	NA	ncRNA_intr	RP11-343N15.1	-	lincRNA	0	ncRNA_intr	RP11-67H24.2	+	lincRNA	0
1501	chr1	43547030	+	chr17	38296670	-	N	Y	N	N	N	N	inso	u	VNTR	intergenic;ir	RNU6-870P,FAM183A	-,+	snRNA,prot	57722;63794	UTR5;UTR5	CASC3	+	protein_cod	0
1502	chr1	200067551	-	chr2	23698251	+	Y	Y	N	N	Y	Y	inso	d	NA	intronic	NR5A2	+	protein_cod	0	intronic;intrc	KLHL29	+	protein_cod	0
1503	chr1	247869911	+	chr22	29065912	-	N	N	N	N	Y	N	inso	u	NA	ncRNA_intr	RP11-634B7.4	+	antisense	0	intronic;intrc	TTC28	-	protein_cod	0
1504	chr1	247568995	+	chr3	196050113	-	N	N	N	N	Y	N	inso	u	NA	intergenic	ZNF496,NLRP3	-,+	protein_cod	73950;10463	ncRNA_intr	TM4SF19-AS1	+	antisense	0
1505	chr1	121215018	+	chr5	49771389	-	N	N	N	N	N	Y	inso	u	NA	intergenic	AL592494.5,RP11-344	+,-	lincRNA,linc	10035;21159	intergenic	EMB,RP11-269M20.2	-,+	protein_cod	34188;127674
1506	chr1	121215340	+	chr5	49771339	-	Y	N	N	N	N	N	inso	u	NA	intergenic	AL592494.5,RP11-344	+,-	lincRNA,linc	10357;20837	intergenic	EMB,RP11-269M20.2	-,+	protein_cod	34138;127724

1507	chr10	109799527	-	chr2	231802114	+	N	N	Y	N	N	N	inso	d	I_LTR_LTR1(	ncRNA_intr	RP11-215N21.1	-	lincRNA	0	intronic	GPR55	-	protein_cod	0
1508	chr10	109799586	-	chr2	231802134	+	Y	Y	N	Y	Y	Y	inso	d	NA	ncRNA_intr	RP11-215N21.1	-	lincRNA	0	intronic	GPR55	-	protein_cod	0
1509	chr10	85222525	-	chr4	4350755	+	N	N	N	N	Y	N	inso	d	NA	intergenic;ir	AL356140.1,RP11-344	-,-	miRNA,lincR	25640;209637	intronic;intrc	NSG1	+	protein_cod	0
1510	chr10	60902409	-	chr7	81789148	+	Y	Y	Y	Y	Y	Y	inso	d	NA	intergenic	TRAF6P1,PHYHIPL	-,+	pseudogene	4467;33941	intronic	CACNA2D1	-	protein_cod	0
1511	chr11	13023176	+	chr18	53590853	-	Y	Y	Y	Y	Y	Y	inso	u	NA	ncRNA_intr	CTC-497E21.3	-	lincRNA	0	ncRNA_intr	CTD-2008L17.1	+	lincRNA	0
1512	chr11	41093789	+	chr20	44244741	-	Y	Y	Y	Y	Y	Y	inso	u	NA	intronic	LRRC4C	-	protein_cod	0	intronic	WFDC9	-	protein_cod	0
1513	chr11	131178152	+	chr20	48731505	-	N	Y	Y	N	Y	N	inso	u	NA	ncRNA_intr	AP002856.5	+	lincRNA	0	intronic	TMEM189-UBE2V1,TME	-,-,-	protein_cod	0
1514	chr11	103792603	+	chr6	108906546	-	N	Y	N	Y	Y	Y	inso	u	I_SINE_AluY	intronic;intrc	PDGFD	-	protein_cod	0	intronic;intrc	FOXO3	+	protein_cod	0
1515	chr12	73290995	+	chr3	95726962	-	Y	Y	Y	Y	Y	Y	inso	u	NA	intergenic	AC131213.1,RP11-314	-,-	miRNA,proc	106570;218742	intergenic	MTHFD2P1,AC108739.1	-,-	pseudogene	324925;158699
1516	chr13	44069838	+	chr15	41871536	-	Y	N	N	N	Y	Y	inso	u	NA	intronic;intrc	ENOX1	-	protein_cod	0	downstream	TYRO3	+	protein_cod	0
1517	chr13	74313856	-	chr8	15289365	+	Y	Y	Y	Y	Y	Y	inso	d	NA	intronic	KLF12	-	protein_cod	0	intergenic	RP11-782K4.1,AC09155	+,+	pseudogene	106287;90028
1518	chr13	68823792	+	chr8	6239912	-	N	N	N	Y	Y	N	inso	u	NA	intergenic;ir	OR7E33P,ELL2P3	+,-	pseudogene	337712;40019	intergenic;ir	RP11-124B13.1,RP11-11	-,-	lincRNA,ant	124854;21160
1519	chr13	91149415	+	chrX	43448546	-	N	Y	Y	N	Y	Y	inso	u	NA	ncRNA_intr	LINC01049	+	lincRNA	0	intergenic	NANOGP10,MAOA	-,+	pseudogene	180738;66921
1520	chr14	93712480	-	chr1	9121447	+	Y	Y	Y	Y	Y	Y	inso	d	NA	exonic	BTBD7	-	protein_cod	0	intronic	SLC2A5	-	protein_cod	0
1521	chr14	55382195	-	chr20	49282703	+	N	N	Y	N	N	Y	inso	d	VNTR	intergenic	GCH1,WDHD1	-,-	protein_cod	12625;23473	intronic	FAM65C	-	protein_cod	0
1522	chr14	93652601	-	chr3	169729734	+	Y	Y	Y	Y	Y	Y	inso	d	NA	intronic	TMEM251,RP11-371E	+,+	protein_cod	0	intergenic;ir	SEC62,GPR160	+,+	protein_cod	13573;25983
1523	chr15	41851396	-	chr13	44069827	+	Y	N	N	N	Y	Y	inso	d	NA	UTR5	TYRO3	+	protein_cod	0	intronic	ENOX1	-	protein_cod	0
1524	chr15	79784432	+	chr5	96849706	-	N	Y	N	Y	Y	N	inso	u	NA	intergenic;ir	KIAA1024,RP11-621H	+,-	protein_cod	19800;67166	ncRNA_intr	RP11-1E3.1	+	lincRNA	0
1525	chr15	40854194	+	chr7	26252971	-	Y	N	Y	Y	N	N	inso	u	NA	intronic;intrc	C15orf57	-	protein_cod	0	UTR3;UTR3	CBX3	+	protein_cod	0
1526	chr16	32823564	+	chr1	121138779	-	Y	N	N	N	N	N	inso	u	NA	ncRNA_intr	RP11-67H24.2	+	lincRNA	0	ncRNA_exo	AL592494.5	+	lincRNA	0
1527	chr17	25290520	+	chr18	15198115	-	N	Y	N	N	N	N	inso	u	NA	intergenic	NONE,RP11-260A9.1	NONE,+	NONE,pseu	NONE;18200	upstream	RP11-454P7.3	-	pseudogene	0
1528	chr18	53590610	-	chr11	13023168	+	Y	Y	Y	Y	Y	Y	inso	d	NA	ncRNA_intr	CTD-2008L17.1	+	lincRNA	0	ncRNA_intr	CTC-497E21.3	-	lincRNA	0
1529	chr18	15197856	-	chr17	25290257	+	N	Y	N	N	N	N	inso	d	NA	upstream	RP11-454P7.3	-	pseudogene	0	intergenic	NONE,RP11-260A9.1	NONE,+	NONE,pseu	NONE;18463
1530	chr18	39769758	-	chr6	153748709	+	Y	N	Y	N	N	N	inso	d	:I_LTR_LTR2	ncRNA_intr	LINC00907	+	lincRNA	0	intergenic;ir	RNA5SP225,RP11-331O	+,+	rRNA,pseuc	7002;238133
1531	chr18	71711514	+	chr9	127885509	-	N	N	Y	N	N	N	inso	u	NA	intergenic;ir	RP11-25L3.3,FBXO15	-,-	lincRNA,pro	10118;29074	intronic;intrc	SCAI	-	protein_cod	0
1532	chr19	21480236	-	chr7	64289938	+	Y	N	N	N	N	N	inso	d	NA	intronic;intrc	ZNF708	-	protein_cod	0	intronic;intrc	ZNF138	+	protein_cod	0
1533	chr2	23698250	+	chr1	200067677	-	Y	Y	N	N	Y	Y	inso	u	NA	intronic	KLHL29	+	protein_cod	0	intronic	NR5A2	+	protein_cod	0
1534	chr2	231802143	+	chr10	109799890	-	Y	Y	Y	Y	Y	Y	inso	u	NA	intronic	GPR55	-	protein_cod	0	ncRNA_exo	RP11-215N21.1	-	lincRNA	0
1535	chr2	47810004	+	chr22	24198551	-	Y	Y	N	Y	Y	N	inso	u	TEI_complex	intergenic	AC138655.1,AC07925	+,-	protein_cod	8006;107851	upstream;dc	SLC2A11,KB-1125A3.11	+,-	protein_cod	0
1536	chr2	41453286	-	chr3	83756635	+	N	N	Y	N	N	N	inso	d	NA	intergenic	HNRNPA1P57,AC0107	+,+	pseudogene	68591;370240	intergenic	CYP51A1P1,SRRM1P2	-,+	pseudogene	899463;229561
1537	chr2	160148181	-	chr3	58772434	+	N	Y	Y	Y	Y	Y	inso	d	NA	intergenic	WDSUB1,BAZ2B	-,-	protein_cod	4871;27309	intronic	C3orf67	-	protein_cod	0
1538	chr2	78091920	-	chr4	174044215	+	N	N	Y	N	N	N	inso	d	EI_SINE_Alu	intergenic	AC105399.2,AC07362	+,+	pseudogene	71707;51140	intergenic	GALNTL6,RP11-10K16.1	+,-	protein_cod	81505;8864
1539	chr20	44244483	-	chr11	41093789	+	Y	Y	Y	Y	Y	Y	inso	d	NA	intronic	WFDC9	-	protein_cod	0	intronic	LRRC4C	-	protein_cod	0
1540	chr20	48731311	-	chr11	131178153	+	N	Y	Y	N	Y	N	inso	d	NA	intronic	TMEM189-UBE2V1,TM	-,-,-	protein_cod	0	ncRNA_intr	AP002856.5	+	lincRNA	0
1541	chr20	54914483	-	chr4	147953943	+	Y	Y	Y	Y	N	N	inso	d	NA	intergenic	RP11-380D15.3,FAM2	-,+	lincRNA,pro	40267;19488	intergenic	RP11-292D4.3,MIR548G	+,-	lincRNA,miR	23646;311838
1542	chr20	52021438	-	chr6	88217551	+	Y	Y	Y	Y	Y	Y	inso	d	NA	ncRNA_intr	RP4-669H2.1,RP4-678	-,-	antisense,ar	0	intronic	SLC35A1	+	protein_cod	0
1543	chr22	29065701	-	chr1	247869898	+	N	N	N	N	Y	N	inso	d	NA	intronic	TTC28	-	protein_cod	0	ncRNA_intr	RP11-634B7.4	+	antisense	0
1544	chr22	24195862	-	chr2	47809988	+	Y	Y	N	Y	Y	N	inso	d	TEI_complex	intergenic	DERL3,KB-1125A3.11	-,-	protein_cod	14663;2752	intergenic	AC138655.1,AC079250.1	+,-	protein_cod	7990;107867
1545	chr3	196049944	-	chr1	247568918	+	N	N	N	N	Y	N	inso	d	NA	ncRNA_intr	TM4SF19-AS1	+	antisense	0	intergenic	ZNF496,NLRP3	-,+	protein_cod	73873;10540
1546	chr3	95726711	-	chr12	73290997	+	Y	Y	Y	Y	Y	Y	inso	d	NA	intergenic	MTHFD2P1,AC108739	-,-	pseudogene	324674;158950	intergenic	AC131213.1,RP11-314D7	-,-	miRNA,proc	106572;218740
1547	chr3	169729756	+	chr14	93653426	-	Y	Y	Y	Y	Y	Y	inso	u	NA	intergenic	SEC62,GPR160	+,+	protein_cod	13595;25961	UTR3	TMEM251	+	protein_cod	0
1548	chr3	58772448	+	chr2	160148934	-	N	Y	Y	Y	Y	Y	inso	u	NA	intronic	C3orf67	-	protein_cod	0	intergenic	WDSUB1,BAZ2B	-,-	protein_cod	5624;26556
1549	chr3	7977706	+	chr6	18358570	-	N	N	Y	N	N	N	inso	u	NA	intergenic	GRM7,LMCD1-AS1	+,-	protein_cod	194491;16786	intergenic;ir	RNU6-263P,RP11-528A1	-,-	snRNA,pseu	51260;5078
1550	chr4	4350924	+	chr10	85222652	-	N	N	N	N	Y	N	inso	u	NA	intronic	NSG1	+	protein_cod	0	intergenic	AL356140.1,RP11-344L1	-,-	miRNA,lincR	25767;209510
1551	chr4	147953941	+	chr20	54914679	-	Y	Y	Y	Y	N	N	inso	u	NA	intergenic	RP11-292D4.3,MIR548	+,-	lincRNA,miR	23644;311840	intergenic	RP11-380D15.3,FAM210	-,+	lincRNA,pro	40463;19292
1552	chr5	49771162	-	chr1	121214999	+	Y	N	N	N	N	Y	inso	d	NA	intergenic	EMB,RP11-269M20.2	-,+	protein_cod	33961;127901	intergenic	AL592494.5,RP11-344P1	+,-	lincRNA,linc	10016;21178
1553	chr5	96849601	-	chr15	79784427	+	N	Y	N	Y	Y	N	inso	d	NA	ncRNA_intr	RP11-1E3.1	+	lincRNA	0	intergenic	KIAA1024,RP11-621H8.2	+,-	protein_cod	19795;67171
1554	chr6	108906228	-	chr11	103792587	+	N	Y	N	Y	Y	Y	inso	d	I_SINE_AluY	intronic	FOXO3	+	protein_cod	0	intronic	PDGFD	-	protein_cod	0
1555	chr6	10762811	+	chr11	118293792	-	N	Y	N	N	N	Y	inso	u	NA	intronic	TMEM14B,SYCP2L,RF	+,+,+	protein_cod	0	intronic	ATP5L	+	protein_cod	0
1556	chr6	153748714	+	chr18	39770209	-	Y	N	Y	N	N	N	inso	u	:I_LTR_LTR2	intergenic	RNA5SP225,RP11-331	+,+	rRNA,pseuc	7007;238128	ncRNA_intr	LINC00907	+	lincRNA	0
1557	chr6	88217546	+	chr20	52021643	-	Y	Y	Y	Y	Y	Y	inso	u	NA	intronic	SLC35A1	+	protein_cod	0	ncRNA_intr	RP4-669H2.1,RP4-678D	-,-	antisense,ar	0
1558	chr6	18358315	-	chr3	7977695	+	N	N	Y	N	N	N	inso	d	NA	intergenic	RNU6-263P,RP11-528	-,-	snRNA,pseu	51005;5333	intergenic	GRM7,LMCD1-AS1	+,-	protein_cod	194480;16797
1559	chr7	81789163	+	chr10	60902930	-	Y	Y	Y	Y	Y	Y	inso	u	NA	intronic	CACNA2D1	-	protein_cod	0	intergenic	TRAF6P1,PHYHIPL	-,+	pseudogene	4988;33420
1560	chr7	26241365	-	chr15	40854180	+	Y	N	Y	Y	N	N	inso	d	NA	UTR5	CBX3	+	protein_cod	0	intronic;intrc	C15orf57	-	protein_cod	0
1561	chr7	64289931	+	chr19	22201713	-	Y	N	N	N	N	N	inso	u	NA	intronic	ZNF138	+	protein_cod	0	ncRNA_intr	AC003973.4	-	lincRNA	0
1562	chr8	15289361	+	chr13	74314059	-	Y	Y	Y	Y	Y	Y	inso	u	NA	intergenic	RP11-782K4.1,AC0915	+,+	pseudogene	106283;90032	intronic	KLF12	-	protein_cod	0
1563	chr9	100675547	+	chr1	86399021	-	N	N	Y	Y	Y	N	inso	u	NA	UTR3	C9orf156	-	protein_cod	0	intronic	COL24A1	-	protein_cod	0
1564	chr9	127885332	-	chr18	71711499	+	N	N	Y	N	N	N	inso	d	NA	intronic	SCAI	-	protein_cod	0	intergenic	RP11-25L3.3,FBXO15	-,-	lincRNA,pro	10103;29089
1565	chrX	43448483	-	chr13	91149407	+	N	Y	Y	N	Y	Y	inso	d	NA	intergenic	NANOGP10,MAOA	-,+	pseudogene	180675;66984	ncRNA_intr	LINC01049	+	lincRNA	0
1566	chrX	3195458	-	chrY	14172718	+	N	N	N	N	Y	N	inso	d	NA	intronic	CXorf28	+	protein_cod	0	intergenic	MXRA5P1,RPS24P1	+,+	pseudogene	64626;192739
1567	chrX	3196038	-	chrY	14172692	+	N	N	N	Y	N	N	inso	d	NA	intronic	CXorf28	+	protein_cod	0	intergenic	MXRA5P1,RPS24P1	+,+	pseudogene	64600;192765
1568	chrY	14172715	+	chrX	3196115	-	N	N	N	Y	Y	N	inso	u	NA	intergenic	MXRA5P1,RPS24P1	+,+	pseudogene	64623;192742	intronic	CXorf28	+	protein_cod	0
1569	chr1	81661371	-	chr1	81660357	+	Y	Y	Y	N	Y	Y	insod	d	NA	intergenic;ir	RPL7P10,RP3-445O1(	-,+	pseudogene	96681;12882	intergenic;ir	RPL7P10,RP3-445O10.1	-,+	pseudogene	95667;13896
1570	chr1	81660349	+	chr1	81661557	-	Y	Y	Y	N	Y	Y	insod	u	NA	intergenic	RPL7P10,RP3-445O1(	-,+	pseudogene	95659;13904	intergenic	RPL7P10,RP3-445O10.1	-,+	pseudogene	96867;12696
1571	chr10	12867180	-	chr10	12867113	+	N	N	Y	N	N	N	insod	d	NA	intronic	CAMK1D	+	protein_cod	0	intronic	CAMK1D	+	protein_cod	0
1572	chr10	43055265	-	chr10	38376457	+	N	Y	N	Y	N	N	insod	d	NA	intergenic	FXYD6P1,EIF3LP2	+,+	pseudogene	4093;6860	intergenic	EIF3LP3,FXYD6P2	-,-	pseudogene	6652;4235
1573	chr10	12867170	+	chr10	12867245	-	N	N	Y	N	N	N	insod	u	NA	intronic	CAMK1D	+	protein_cod	0	intronic	CAMK1D	+	protein_cod	0
1574	chr10	38376454	+	chr10	43055665	-	N	Y	N	Y	N	N	insod	u	NA	intergenic;ir	EIF3LP3,FXYD6P2	-,-	pseudogene	6649;4238	intergenic;ir	FXYD6P1,EIF3LP2	+,+	pseudogene	4493;6460
1575	chr12	133141109	+	chr12	133344190	-	N	N	N	N	Y	N	insod	u	NA	intronic;intrc	FBRSL1	+	protein_cod	0	intergenic;ir	ANKLE2,GOLGA3	-,-	protein_cod	5716;1305
1576	chr13	89112603	-	chr13	89110969	+	N	N	Y	N	Y	N	insod	d	NA	intergenic	RPL29P29,LINC00433	-,+	pseudogene	223379;80516	intergenic	RPL29P29,LINC00433	-,+	pseudogene	221745;82150
1577	chr13	89110970	+	chr13	89112881	-	N	N	Y	N	Y	N	insod	u	NA	intergenic;ir	RPL29P29,LINC00433	-,+	pseudogene	221746;82149	intergenic;ir	RPL29P29,LINC00433	-,+	pseudogene	223657;80238
1578	chr14	67171710	-	chr14	67170334	+	Y	Y	Y	Y	Y	Y	insod	d	NA	intronic	GPHN	+	protein_cod	0	intronic	GPHN	+	protein_cod	0
1579	chr14	67170329	+	chr14	67171997	-	Y	Y	Y	Y	Y	Y	insod	u	NA	intronic	GPHN	+	protein_cod	0	intronic	GPHN	+	protein_cod	0
1580	chr2	61703127	-	chr2	61700745	+	Y	Y	Y	Y	Y	N	insod	d	NA	ncRNA_intr	RP11-355B11.2	+	antisense	0	ncRNA_intr	RP11-355B11.2	+	antisense	0
1581	chr2	125052915	-	chr2	125051760	+	Y	Y	Y	Y	Y	Y	insod	d	NA	intronic	CNTNAP5	+	protein_cod	0	intronic	CNTNAP5	+	protein_cod	0
1582	chr2	61700751	+	chr2	61703457	-	Y	Y	Y	Y	Y	N	insod	u	NA	ncRNA_intr	RP11-355B11.2	+	antisense	0	ncRNA_intr	RP11-355B11.2	+	antisense	0
1583	chr2	125051756	+	chr2	125053240	-	Y	Y	Y	Y	Y	Y	insod	u	NA	intronic	CNTNAP5	+	protein_cod	0	intronic	CNTNAP5	+	protein_cod	0
1584	chr6	167411275	-	chr6	167411215	+	N	N	Y	N	N	N	insod	d	NA	ncRNA_intr	RP1-167A14.2	-	antisense	0	ncRNA_intr	RP1-167A14.2	-	antisense	0
1585	chr6	167411249	+	chr6	167411432	-	N	N	Y	N	N	N	insod	u	NA	ncRNA_intr	RP1-167A14.2	-	antisense	0	ncRNA_intr	RP1-167A14.2	-	antisense	0
1586	chr8	71767772	-	chr8	68667791	+	N	N	N	N	Y	N	insod	d	NA	intergenic	Y_RNA,RP11-326E22.	-,+	misc_RNA,a	74529;299920	intergenic	CPA6,NDUFS5P6	-,+	protein_cod	9171;93488
1587	chr8	100157921	-	chr8	100157820	+	Y	Y	Y	Y	Y	Y	insod	d	NA	intronic	VPS13B	+	protein_cod	0	intronic	VPS13B	+	protein_cod	0
1588	chr8	68667943	+	chr8	71768006	-	N	N	N	N	Y	N	insod	u	NA	intergenic	CPA6,NDUFS5P6	-,+	protein_cod	9323;93336	intergenic	Y_RNA,RP11-326E22.1	-,+	misc_RNA,a	74763;299686
1589	chr8	100157810	+	chr8	100158248	-	Y	Y	Y	Y	Y	Y	insod	u	NA	intronic	VPS13B	+	protein_cod	0	intronic	VPS13B	+	protein_cod	0
1590	chr1	17225522	-	chr1	149220851	+	N	N	N	N	N	Y	insou	d	NA	intergenic	RNU1-2,RP11-108M9.	+,-	snRNA,lincR	2884;5312	intergenic	RNU1-92P,RNVU1-18	-,-	snRNA,snR	5551;3207

1591	chr1	149220989	+	chr1	17225875	-	N	N	N	N	N	Y	insou	u	NA	intergenic	RNU1-92P,RNVU1-18	-,-	snRNA,snR	5689;3069	intergenic	RNU1-2,RP11-108M9.6	+,-	snRNA,linc	3237;4959
1592	chr12	25956127	-	chr12	125801148	+	Y	N	N	N	N	N	insou	d	NA	ncRNA_intr	RP11-443N24.2	-	lincRNA	0	intergenic;ir	NONE,TMEM132B	NONE,+	NONE,prote	NONE;10014
1593	chr12	104359630	-	chr12	125801148	+	N	Y	Y	Y	Y	N	insou	d	NA	UTR5	TDG	+	protein_cod	0	intergenic;ir	NONE,TMEM132B	NONE,+	NONE,prote	NONE;10014
1594	chr12	125801164	+	chr12	25959140	-	Y	N	N	N	N	N	insou	u	NA	intergenic;ir	NONE,TMEM132B	NONE,+	NONE,prote	NONE;9998	ncRNA_intr	RP11-443N24.2	-	lincRNA	0
1595	chr12	125801164	+	chr12	104382542	-	N	Y	Y	Y	Y	N	insou	u	NA	intergenic;ir	NONE,TMEM132B	NONE,+	NONE,prote	NONE;9998	UTR3	TDG	+	protein_cod	0
1596	chr14	47117868	-	chr14	63226284	+	N	Y	N	N	N	N	insou	d	EI_SINE_Alu	intergenic	LINC00871,RPL10L	+,-	lincRNA,pro	146842;2354	intronic	KCNH5	-	protein_cod	0
1597	chr2	116974339	-	chr2	116981881	+	N	N	Y	N	N	N	insou	d	NA	intergenic	DPP10,RP11-338I24.1	+,-	protein_cod	371011;470455	intergenic	DPP10,RP11-338I24.1	+,-	protein_cod	378553;462913
1598	chr2	131886577	-	chr2	131887307	+	N	Y	N	N	N	N	insou	d	NA	intronic	PLEKHB2	+	protein_cod	0	intronic	PLEKHB2	+	protein_cod	0
1599	chr2	116982308	+	chr2	116978298	-	N	N	Y	N	N	N	insou	u	NA	intergenic	DPP10,RP11-338I24.1	+,-	protein_cod	378980;462486	intergenic	DPP10,RP11-338I24.1	+,-	protein_cod	374970;466496
1600	chr2	131887330	+	chr2	131886643	-	N	Y	N	N	N	N	insou	u	NA	intronic	PLEKHB2	+	protein_cod	0	intronic	PLEKHB2	+	protein_cod	0
1601	chr5	169597468	-	chr5	169598756	+	Y	Y	Y	Y	Y	Y	insou	d	NA	intergenic	KRT18P41,CTB-27N1.	-,-	pseudogene	28524;21115	intergenic	KRT18P41,CTB-27N1.1	-,-	pseudogene	29812;19827
1602	chr5	169598750	+	chr5	169597696	-	Y	Y	Y	Y	Y	Y	insou	u	NA	intergenic	KRT18P41,CTB-27N1.	-,-	pseudogene	29806;19833	intergenic	KRT18P41,CTB-27N1.1	-,-	pseudogene	28752;20887
1603	chr7	151010035	-	chr7	151012106	+	Y	Y	Y	Y	Y	Y	insou	d	NA	intergenic	SMARCD3,NUB1	-,+	protein_cod	35053;28750	intergenic	SMARCD3,NUB1	-,+	protein_cod	37124;26679
1604	chr7	151012103	+	chr7	151010436	-	Y	Y	Y	Y	Y	Y	insou	u	NA	intergenic	SMARCD3,NUB1	-,+	protein_cod	37121;26682	intergenic	SMARCD3,NUB1	-,+	protein_cod	35454;28349
1605	chr9	107816637	-	chr9	107817347	+	Y	Y	Y	Y	Y	Y	insou	d	NA	intergenic	RP11-217B7.3,RN7SK	-,+	lincRNA,mis	62575;42400	intergenic	RP11-217B7.3,RN7SKP1	-,+	lincRNA,mis	63285;41690
1606	chr9	107817341	+	chr9	107816979	-	Y	Y	Y	Y	Y	Y	insou	u	NA	intergenic	RP11-217B7.3,RN7SK	-,+	lincRNA,mis	63279;41696	intergenic	RP11-217B7.3,RN7SKP1	-,+	lincRNA,mis	62917;42058
1607	chr1	168025732	+	chr19	24033178	+	Y	Y	Y	Y	Y	Y	inss	d	NA	intronic	DCAF6	+	protein_cod	0	ncRNA_intr	RP11-255H23.4	-	lincRNA	0
1608	chr1	37938378	+	chr2	68914881	+	Y	N	Y	Y	N	N	inss	d	NA	ncRNA_intr	LINC01137	-	antisense	0	intronic	ARHGAP25	+	protein_cod	0
1609	chr1	218884596	+	chr3	25100012	+	Y	Y	Y	Y	Y	Y	inss	d	NA	intergenic	U3,RP11-412H9.1	-,-	snoRNA,pse	169355;170346	ncRNA_intr	AC133680.1	+	lincRNA	0
1610	chr10	112178163	+	chr7	92976835	+	N	N	N	N	N	Y	inss	d	TEI_complex	intergenic;ir	SMNDC1,RP11-525A1	-,-	protein_cod	113454;10435	intronic;intrc	CCDC132	+	protein_cod	0
1611	chr11	48378753	+	chr12	45460238	+	N	N	Y	Y	Y	Y	inss	u	NA	intergenic;ir	OR4C4P,OR4C5	-,-	pseudogene	4754;8284	ncRNA_intr	RP11-478B9.1	+	antisense	0
1612	chr11	61735119	+	chr2	181738245	+	N	Y	N	N	N	Y	inss	d	NA	UTR5	FTH1	-	protein_cod	0	upstream	FTH1P20	-	pseudogene	0
1613	chr11	38812669	+	chr8	52730143	+	Y	Y	Y	Y	Y	Y	inss	u	NA	intergenic	RP11-64I17.1,RP11-31	+,+	lincRNA,pse	116878;370334	UTR5;UTR3	AC090186.1,PCMTD1	+,-	protein_cod	0
1614	chr11	63660830	+	chrX	10159335	+	N	Y	N	N	N	N	inss	u	NA	intronic;intrc	MARK2	+	protein_cod	0	intronic;intrc	CLCN4	+	protein_cod	0
1615	chr11	63660831	+	chrX	109628649	+	N	N	N	Y	Y	N	inss	u	NA	intronic	MARK2	+	protein_cod	0	intronic	AMMECR1,RGAG1	-,+	protein_cod	0
1616	chr11	70835439	+	chrY	13203786	+	N	N	Y	N	N	N	inss	u	NA	intronic	SHANK2	-	protein_cod	0	intergenic	NONE,RP1-85D24.3	NONE,-	NONE,pseu	NONE;58955
1617	chr12	45460369	+	chr11	48378753	+	N	N	Y	Y	Y	Y	inss	d	NA	ncRNA_intr	RP11-478B9.1	+	antisense	0	intergenic	OR4C4P,OR4C5	-,-	pseudogene	4754;8284
1618	chr12	56990096	+	chr15	39994625	+	Y	Y	Y	Y	Y	Y	inss	d	NA	UTR3	BAZ2A	-	protein_cod	0	intronic	FSIP1	-	protein_cod	0
1619	chr14	64066516	+	chr18	13152438	+	N	N	N	N	N	Y	inss	d	NA	exonic	WDR89	-	protein_cod	0	ncRNA_exo	RP11-807E13.2	-	pseudogene	0
1620	chr14	67968244	+	chr19	4965544	+	N	N	N	Y	N	N	inss	d	NA	intronic	TMEM229B	-	protein_cod	0	intergenic	UHRF1,KDM4B	+,+	processed_	3379;3581
1621	chr14	72962479	+	chr4	60491838	+	N	N	N	N	Y	N	inss	d	NA	intronic;intrc	RGS6	+	protein_cod	0	ncRNA_intr	RP11-725D20.1	-	lincRNA	0
1622	chr15	39994637	+	chr12	56989716	+	Y	Y	Y	Y	Y	Y	inss	u	NA	intronic	FSIP1	-	protein_cod	0	UTR3	BAZ2A	-	protein_cod	0
1623	chr17	32673386	+	chr10	74767364	+	N	N	N	N	Y	N	inss	u	NA	intergenic	RP11-521P1.1,CCL13	-,+	pseudogene	14139;10085	UTR3	P4HA1	-	protein_cod	0
1624	chr17	74377422	+	chr2	153997624	+	Y	N	N	Y	N	Y	inss	d	NA	intronic	PRPSAP1,SPHK1	-,+	protein_cod	0	intergenic	UBQLN4P2,ATP5F1P4	+,+	pseudogene	263343;17828
1625	chr17	7167959	+	chr8	30145402	+	Y	Y	Y	Y	Y	Y	inss	u	NA	upstream	CLDN7	-	protein_cod	0	intergenic	CTD-2345M20.2,CTD-31	-,-	pseudogene	37794;44075
1626	chr18	13152439	+	chr14	64066317	+	N	N	N	N	N	Y	inss	u	NA	ncRNA_exo	RP11-807E13.2	-	pseudogene	0	exonic	WDR89	-	protein_cod	0
1627	chr18	23467619	+	chr2	209970708	+	N	Y	N	N	N	N	inss	u	NA	intergenic	RN7SL97P,RP11-737G	-,-	misc_RNA,p	69123;65561	intergenic	HSPA8P6,CRYGFP	-,+	pseudogene	31836;39257
1628	chr19	24033181	+	chr1	168024587	+	Y	Y	Y	Y	Y	Y	inss	u	NA	ncRNA_intr	RP11-255H23.4	-	lincRNA	0	intronic	DCAF6	+	protein_cod	0
1629	chr19	4965591	+	chr14	67968123	+	N	N	N	Y	N	N	inss	u	NA	intergenic	UHRF1,KDM4B	+,+	processed_	3426;3534	intronic	TMEM229B	-	protein_cod	0
1630	chr2	68914879	+	chr1	37938257	+	Y	N	Y	Y	N	N	inss	u	NA	intronic	ARHGAP25	+	protein_cod	0	ncRNA_intr	LINC01137	-	antisense	0
1631	chr2	181738517	+	chr11	61734941	+	N	Y	N	N	N	Y	inss	u	NA	upstream	FTH1P20	-	pseudogene	0	UTR5	FTH1	-	protein_cod	0
1632	chr2	137698271	+	chr16	19263768	+	N	N	N	N	Y	N	inss	d	NA	intronic	THSD7B	+	protein_cod	0	intronic	SYT17	+	protein_cod	0
1633	chr2	153997621	+	chr17	74377274	+	Y	N	N	Y	N	Y	inss	u	NA	intergenic	UBQLN4P2,ATP5F1P4	+,+	pseudogene	263340;17831	intronic	PRPSAP1,SPHK1	-,+	protein_cod	0
1634	chr2	209970999	+	chr18	23467604	+	N	Y	N	N	N	N	inss	d	NA	intergenic	HSPA8P6,CRYGFP	-,+	pseudogene	32127;38966	intergenic	RN7SL97P,RP11-737G2	-,-	misc_RNA,p	69108;65576
1635	chr2	204056057	+	chr9	132191814	+	N	Y	Y	Y	N	Y	inss	d	NA	intronic	NBEAL1	+	protein_cod	0	intergenic	RP11-65J3.6,RP11-65J3.	-,-	pseudogene	49065;1117
1636	chr21	44757304	+	chr7	44294744	+	N	N	N	N	Y	N	inss	d	NA	intergenic;ir	LINC00322,AP001046	-,-	lincRNA,linc	5385;20723	intronic	CAMK2B	-	protein_cod	0
1637	chr21	44757304	+	chr7	44294746	+	Y	N	N	N	N	N	inss	d	NA	intergenic;ir	LINC00322,AP001046	-,-	lincRNA,linc	5385;20723	intronic	CAMK2B	-	protein_cod	0
1638	chr22	32928560	+	chr6	24683988	+	Y	Y	Y	Y	Y	Y	inss	d	NA	intronic	SYN3	-	protein_cod	0	intronic	ACOT13	+	protein_cod	0
1639	chr3	25100001	+	chr1	218883358	+	Y	Y	Y	Y	Y	Y	inss	u	NA	ncRNA_intr	AC133680.1	+	lincRNA	0	intergenic	U3,RP11-412H9.1	-,-	snoRNA,pse	168117;171584
1640	chr3	56765638	+	chr7	147539102	+	Y	Y	Y	Y	Y	Y	inss	u	NA	UTR3	ARHGEF3	-	protein_cod	0	intronic	CNTNAP2	+	protein_cod	0
1641	chr4	60491828	+	chr14	72962329	+	N	N	N	N	Y	N	inss	u	NA	ncRNA_intr	RP11-725D20.1	-	lincRNA	0	intronic	RGS6	+	protein_cod	0
1642	chr4	80894475	+	chr5	21207714	+	Y	Y	Y	Y	Y	Y	inss	d	EI_LINE_L1H	intronic	ANTXR2	-	protein_cod	0	intergenic	RP11-811J10.1,RP11-37	+,-	pseudogene	10745;116268
1643	chr5	9024647	+	chr20	22863398	+	N	Y	N	N	N	N	inss	d	VNTR	ncRNA_intr	CTD-2215L10.1	+	antisense	0	intergenic	KRT18P3,CYB5P4	-,-	pseudogene	148671;2910
1644	chr5	21207730	+	chr4	80888060	+	Y	Y	Y	Y	Y	Y	inss	u	EI_LINE_L1H	intergenic	RP11-811J10.1,RP11-3	+,-	pseudogene	10761;116252	intronic	ANTXR2	-	protein_cod	0
1645	chr6	24684003	+	chr22	32927895	+	Y	Y	Y	Y	Y	Y	inss	u	NA	intronic	ACOT13	+	protein_cod	0	intronic	SYN3	-	protein_cod	0
1646	chr6	43962453	+	chr8	14835893	+	Y	Y	N	Y	N	N	inss	d	NA	intergenic	RP11-344J7.2,RP5-11	-,-	lincRNA,ant	142383;1007	intronic	SGCZ	-	protein_cod	0
1647	chr6	89435365	+	chrX	119277793	+	N	Y	Y	N	N	N	inss	d	NA	intronic;intrc	RNGTT	-	protein_cod	0	ncRNA_exo	RP4-755D9.1	+	antisense	0
1648	chr7	147539665	+	chr3	56765621	+	Y	Y	Y	Y	Y	Y	inss	d	NA	intronic	CNTNAP2	+	protein_cod	0	UTR3	ARHGEF3	-	protein_cod	0
1649	chr8	52731484	+	chr11	38812656	+	Y	Y	Y	Y	Y	Y	inss	d	NA	UTR3	PCMTD1	-	protein_cod	0	intergenic	RP11-64I17.1,RP11-313M	+,+	lincRNA,pse	116865;370347
1650	chr8	30145623	+	chr17	7167890	+	Y	Y	Y	Y	Y	Y	inss	d	NA	intergenic	CTD-2345M20.2,CTD-	-,-	pseudogene	38015;43854	upstream	CLDN7	-	protein_cod	0
1651	chr8	20479568	+	chr21	43250843	+	Y	N	N	N	N	N	inss	u	NA	intergenic	U3,RP11-108E14.1	-,-	snoRNA,linc	7131;33634	intronic	PRDM15	-	protein_cod	0
1652	chr8	14835894	+	chr6	43962299	+	Y	Y	N	Y	N	N	inss	u	NA	intronic	SGCZ	-	protein_cod	0	intergenic	RP11-344J7.2,RP5-1120	-,-	lincRNA,ant	142229;1161
1653	chr9	132191830	+	chr2	204055415	+	N	Y	Y	Y	N	Y	inss	u	NA	intergenic	RP11-65J3.6,RP11-65.	-,-	pseudogene	49081;1101	intronic	NBEAL1	+	protein_cod	0
1654	chrX	109628851	+	chr11	63660830	+	N	Y	N	Y	Y	N	inss	d	NA	intronic	AMMECR1,RGAG1	-,+	protein_cod	0	intronic;intrc	MARK2	+	protein_cod	0
1655	chrX	119277813	+	chr6	89435091	+	N	Y	N	N	N	N	inss	u	NA	ncRNA_intr	NKAPP1,RP4-755D9.1	-,+	pseudogene	0	intronic	RNGTT	-	protein_cod	0
1656	chrX	119277813	+	chr6	89435304	+	N	N	Y	N	N	N	inss	u	NA	ncRNA_intr	NKAPP1,RP4-755D9.1	-,+	pseudogene	0	intronic	RNGTT	-	protein_cod	0
1657	chr1	16376517	+	chr1	16386259	+	Y	Y	N	Y	Y	Y	inssd	u	NA	intronic	CLCNKB	+	protein_cod	0	intronic	FAM131C	-	protein_cod	0
1658	chr1	16386334	+	chr1	16376519	+	Y	Y	N	Y	Y	Y	inssd	d	NA	intronic;intrc	FAM131C	-	protein_cod	0	intronic;intrc	CLCNKB	+	protein_cod	0
1659	chr1	24994644	+	chr1	24997171	+	N	N	N	Y	N	N	inssd	u	NA	intronic;intrc	SRRM1	+	protein_cod	0	intronic;intrc	SRRM1	+	protein_cod	0
1660	chr1	24997299	+	chr1	24994642	+	N	N	N	Y	N	N	inssd	d	NA	intronic;intrc	SRRM1	+	protein_cod	0	intronic;intrc	SRRM1	+	protein_cod	0
1661	chr11	48609308	+	chr11	55241409	+	N	N	Y	N	N	N	inssd	u	NA	intergenic;ir	OR4A45P,OR4A41P	+,+	pseudogene	7420;2004	intergenic;ir	OR4A13P,OR4A50P	+,+	pseudogene	6221;2010
1662	chr11	55241611	+	chr11	48609297	+	N	N	Y	N	N	N	inssd	d	NA	intergenic	OR4A13P,OR4A50P	+,+	pseudogene	6423;1808	intergenic	OR4A45P,OR4A41P	+,+	pseudogene	7409;2015
1663	chr11	63698908	+	chr11	63701654	+	N	Y	N	Y	Y	Y	inssd	u	NA	intergenic	RCOR2,NAA40	-,+	protein_cod	14592;7560	intergenic	RCOR2,NAA40	-,+	protein_cod	17338;4814
1664	chr11	63701944	+	chr11	63698907	+	N	Y	N	Y	Y	Y	inssd	d	NA	intergenic	RCOR2,NAA40	-,+	protein_cod	17628;4524	intergenic	RCOR2,NAA40	-,+	protein_cod	14591;7561
1665	chr13	34811552	+	chr13	34815510	+	Y	Y	Y	Y	Y	Y	inssd	u	NA	intergenic	SNORA25,RP11-16D2	-,+	snoRNA,linc	136577;110628	intergenic	SNORA25,RP11-16D22.	-,+	snoRNA,linc	140535;106670
1666	chr13	34815595	+	chr13	34811549	+	Y	Y	Y	Y	Y	Y	inssd	d	NA	intergenic	SNORA25,RP11-16D2	-,+	snoRNA,linc	140620;106585	intergenic	SNORA25,RP11-16D22.	-,+	snoRNA,linc	136574;110631
1667	chr15	52388395	+	chr15	52389649	+	Y	Y	Y	Y	Y	Y	inssd	u	NA	ncRNA_intr	CTD-2184D3.5	-	antisense	0	ncRNA_intr	CTD-2184D3.5	-	antisense	0
1668	chr15	52389802	+	chr15	52388397	+	Y	Y	Y	Y	Y	Y	inssd	d	NA	ncRNA_intr	CTD-2184D3.5	-	antisense	0	ncRNA_intr	CTD-2184D3.5	-	antisense	0
1669	chr2	90448284	+	chr2	91669435	+	N	N	N	N	N	Y	inssd	u	NA	intergenic	AC113612.1,CH17-132	+,+	pseudogene	147882;9917	intergenic	AC018696.1,IGKV1OR2-	+,+	pseudogene	33286;9652
1670	chr2	91670744	+	chr2	90447574	+	N	N	Y	N	N	N	inssd	d	NA	intergenic	AC018696.1,IGKV1OR	+,+	pseudogene	34595;8343	intergenic	AC113612.1,CH17-132F	+,+	pseudogene	147172;10627
1671	chr2	91671108	+	chr2	90447610	+	N	N	N	N	N	Y	inssd	d	NA	intergenic	AC018696.1,IGKV1OR	+,+	pseudogene	34959;7979	intergenic	AC113612.1,CH17-132F	+,+	pseudogene	147208;10591
1672	chr2	125766560	+	chr2	125768248	+	Y	Y	Y	Y	Y	Y	inssd	u	NA	intergenic	RNU6-259P,snosnR60	+,+	snRNA,snol	19658;119849	intergenic	RNU6-259P,snosnR60_Z	+,+	snRNA,snol	21346;118161
1673	chr2	125768401	+	chr2	125766562	+	Y	Y	Y	Y	Y	Y	inssd	d	NA	intergenic	RNU6-259P,snosnR60	+,+	snRNA,snol	21499;118008	intergenic	RNU6-259P,snosnR60_Z	+,+	snRNA,snol	19660;119847
1674	chr20	40841903	+	chr20	40844324	+	Y	Y	Y	Y	Y	Y	inssd	u	NA	intronic	PTPRT	-	protein_cod	0	intronic	PTPRT	-	protein_cod	0

1675	chr20	40844386	+	chr20	40841904	+	Y	Y	Y	Y	Y	Y	inssd	d	NA	intronic	PTPRT	-	protein_cod	0	intronic	PTPRT	-	protein_cod	0
1676	chr4	32071051	+	chr4	32074511	+	Y	Y	Y	N	Y	Y	inssd	u	NA	ncRNA_intr	RP11-734I18.1	+	lincRNA	0	ncRNA_intr	RP11-734I18.1	+	lincRNA	0
1677	chr4	32074611	+	chr4	32071041	+	Y	Y	Y	N	Y	Y	inssd	d	NA	ncRNA_intr	RP11-734I18.1	+	lincRNA	0	ncRNA_intr	RP11-734I18.1	+	lincRNA	0
1678	chr7	142191338	+	chr7	142216434	+	N	N	N	Y	N	Y	inssd	u	NA	upstream	TRBV12-2	-	TR_V_pseu	0	ncRNA_exc	TRBV12-1	-	TR_V_pseu	0
1679	chr7	142216934	+	chr7	142191267	+	N	N	N	Y	N	Y	inssd	d	NA	upstream;u	TRBV12-1	-	TR_V_pseu	0	upstream;u	TRBV12-2	-	TR_V_pseu	0
1680	chr9	69786732	+	chr9	69787287	+	N	N	N	N	Y	N	inssd	u	NA	intergenic	IGKV1OR-2,AL359955	-,-	IG_V_pseu	8779;35028	intergenic	IGKV1OR-2,AL359955.1	-,-	IG_V_pseu	9334;34473
1681	chr1	84056667	+	chr1	84057333	+	Y	Y	Y	Y	Y	Y	inssu	d	NA	ncRNA_intr	RP11-475O6.1	-	lincRNA	0	ncRNA_intr	RP11-475O6.1	-	lincRNA	0
1682	chr1	84057335	+	chr1	84056214	+	Y	Y	Y	Y	Y	Y	inssu	u	NA	ncRNA_intr	RP11-475O6.1	-	lincRNA	0	ncRNA_intr	RP11-475O6.1	-	lincRNA	0
1683	chr12	116077525	+	chr12	116079906	+	Y	Y	Y	Y	Y	Y	inssu	d	NA	ncRNA_exc	RP11-1028N23.4	-	lincRNA	0	intergenic	RP11-1028N23.4,RN7SL	-,+	lincRNA,mis	2367;75624
1684	chr12	116079899	+	chr12	116077448	+	Y	Y	Y	Y	Y	Y	inssu	u	NA	intergenic	RP11-1028N23.4,RN7	-,+	lincRNA,mis	2360;75631	ncRNA_spli	RP11-1028N23.4	-	lincRNA	0
1685	chr12	129192745	+	chr12	129193257	+	N	N	N	N	N	Y	inssu	d	NA	downstream	TMEM132C	+	protein_cod	0	downstream	TMEM132C	+	protein_cod	0
1686	chr12	129193325	+	chr12	129192677	+	N	N	N	N	N	Y	inssu	u	NA	downstream	TMEM132C	+	protein_cod	0	downstream	TMEM132C	+	protein_cod	0
1687	chr17	46615804	+	chr17	46617219	+	Y	Y	Y	Y	Y	Y	inssu	d	NA	intergenic	HOXB1,HOXB2	-,-	protein_cod	7445;4213	intergenic	HOXB1,HOXB2	-,-	protein_cod	8860;2798
1688	chr17	46617218	+	chr17	46615633	+	Y	Y	Y	Y	Y	Y	inssu	u	NA	intergenic	HOXB1,HOXB2	-,-	protein_cod	8859;2799	intergenic	HOXB1,HOXB2	-,-	protein_cod	7274;4384
1689	chr2	90450634	+	chr2	90448894	+	N	N	N	N	Y	N	inssu	u	NA	intergenic	AC113612.1,CH17-132	+,+	pseudogene	150232;7567	intergenic	AC113612.1,CH17-132F	+,+	pseudogene	148492;9307
1690	chr2	120417201	+	chr2	120418494	+	Y	Y	Y	Y	Y	Y	inssu	d	NA	intronic	PCDP1	+	protein_cod	0	UTR3	PCDP1	+	protein_cod	0
1691	chr2	120418493	+	chr2	120417082	+	Y	Y	Y	Y	Y	Y	inssu	u	NA	UTR3	PCDP1	+	protein_cod	0	intronic	PCDP1	+	protein_cod	0
1692	chr20	2359903	+	chr20	2360609	+	Y	Y	Y	Y	Y	Y	inssu	d	NA	intergenic	TGM3,TGM6	+,+	protein_cod	38179;1651	upstream	TGM6	+	protein_cod	0
1693	chr20	2360614	+	chr20	2359702	+	Y	Y	Y	Y	Y	Y	inssu	u	NA	upstream	TGM6	+	protein_cod	0	intergenic	TGM3,TGM6	+,+	protein_cod	37978;1852
1694	chr3	57522710	+	chr3	57524052	+	N	N	N	N	Y	N	inssu	d	NA	intronic;intr	DNAH12	-	protein_cod	0	intronic;intr	DNAH12	-	protein_cod	0
1695	chr3	57524041	+	chr3	57522647	+	N	N	N	N	Y	N	inssu	u	NA	intronic;intr	DNAH12	-	protein_cod	0	intronic;intr	DNAH12	-	protein_cod	0
1696	chr4	32065877	+	chr4	32071042	+	N	N	N	N	Y	Y	inssu	d	NA	ncRNA_intr	RP11-734I18.1	+	lincRNA	0	ncRNA_intr	RP11-734I18.1	+	lincRNA	0
1697	chr4	32071049	+	chr4	32065772	+	N	N	N	N	Y	Y	inssu	u	NA	ncRNA_intr	RP11-734I18.1	+	lincRNA	0	ncRNA_intr	RP11-734I18.1	+	lincRNA	0
1698	chr4	91933858	+	chr4	91936102	+	N	Y	Y	N	N	N	inssu	d	NA	intronic;intr	CCSER1	+	protein_cod	0	intronic	CCSER1	+	protein_cod	0
1699	chr4	91936098	+	chr4	91933643	+	N	Y	Y	N	N	N	inssu	u	NA	intronic;intr	CCSER1	+	protein_cod	0	intronic;intr	CCSER1	+	protein_cod	0
1700	chr5	143512867	+	chr5	143515048	+	Y	Y	Y	Y	Y	Y	inssu	d	NA	intergenic	CTB-57H20.1,RN7SL8	+,+	sense_over	304530;7577	intergenic	CTB-57H20.1,RN7SL87F	+,+	sense_over	306711;5396
1701	chr5	143515052	+	chr5	143512490	+	Y	Y	Y	Y	Y	Y	inssu	u	NA	intergenic	CTB-57H20.1,RN7SL8	+,+	sense_over	306715;5392	intergenic	CTB-57H20.1,RN7SL87F	+,+	sense_over	304153;7954
1702	chr6	119012288	+	chr6	119013928	+	Y	Y	Y	Y	Y	Y	inssu	d	NA	intronic	CEP85L	-	protein_cod	0	intronic	CEP85L	-	protein_cod	0
1703	chr6	119013927	+	chr6	119011720	+	Y	Y	Y	Y	Y	Y	inssu	u	NA	intronic	CEP85L	-	protein_cod	0	intronic	CEP85L	-	protein_cod	0
1704	chr7	63296722	+	chr7	67427886	+	N	N	Y	N	N	Y	inssu	d	NA	intergenic	CTD-2526L21.3,RP11-	+,+	pseudogene	46246;6970	intergenic	RP11-421N10.1,RP11-35	-,+	pseudogene	192431;57354
1705	chr7	63296759	+	chr7	67427873	+	Y	Y	N	Y	N	N	inssu	d	NA	intergenic	CTD-2526L21.3,RP11-	+,+	pseudogene	46283;6933	intergenic	RP11-421N10.1,RP11-35	-,+	pseudogene	192418;57367
1706	chr7	67427889	+	chr7	63296477	+	Y	Y	Y	Y	N	Y	inssu	u	NA	intergenic	RP11-421N10.1,RP11-	-,+	pseudogene	192434;57351	intergenic	CTD-2526L21.3,RP11-34	+,+	pseudogene	46001;7215
1707	chr9	20077963	+	chr9	20079073	+	Y	Y	Y	Y	Y	Y	inssu	d	NA	intergenic	RP11-378I6.2,AL5126	-,+	pseudogene	119818;217039	intergenic	RP11-378I6.2,AL512635.	-,+	pseudogene	120928;215929
1708	chr9	20079065	+	chr9	20077627	+	Y	Y	Y	Y	Y	Y	inssu	u	NA	intergenic	RP11-378I6.2,AL5126	-,+	pseudogene	120920;215937	intergenic	RP11-378I6.2,AL512635.	-,+	pseudogene	119482;217375
1709	chrX	52892022	+	chrX	55678935	+	N	N	N	Y	N	N	inssu	d	TEI_complex	intronic	XAGE3	-	protein_cod	0	intergenic	RP11-167P23.4,RP11-16	-,-	pseudogene	16846;2207
1710	chrX	55678954	+	chrX	52886725	+	N	N	N	Y	N	N	inssu	u	TEI_complex	intergenic;ir	RP11-167P23.4,RP11-	-,-	pseudogene	16865;2188	intergenic;ir	EIF4A2P4,XAGE3	-,-	pseudogene	23776;4832
1711	chr1	197756787	+	chr1	197757986	-	Y	Y	Y	Y	Y	Y	invers	na	NA	intergenic	DENND1B,C1orf53	-,+	protein_cod	12024;114990	intergenic	DENND1B,C1orf53	-,+	protein_cod	13223;113791
1712	chr1	205178621	+	chr1	205178785	-	Y	Y	Y	Y	Y	Y	invers	na	NA	intronic	DSTYK	-	protein_cod	0	intronic	DSTYK	-	protein_cod	0
1713	chr10	39086497	+	chr10	39143238	-	N	N	Y	N	N	N	invers	na	NA	intergenic	RP11-96F8.1,NONE	+,NONE	pseudogene	10258;NONE	intergenic	RP11-96F8.1,NONE	+,NONE	pseudogene	66999;NONE
1714	chr10	75419713	+	chr10	75419910	-	Y	Y	Y	Y	Y	Y	invers	na	NA	ncRNA_intr	RP11-464F9.21	+	antisense	0	ncRNA_intr	RP11-464F9.21	+	antisense	0
1715	chr13	82356870	+	chr13	82356977	-	N	Y	Y	Y	Y	N	invers	na	NA	intergenic	PTMAP5,GYG1P2	+,+	pseudogene	91663;1040396	intergenic	PTMAP5,GYG1P2	+,+	pseudogene	91770;1040289
1716	chr13	89110970	+	chr13	89112881	-	N	Y	N	N	N	N	invers	na	NA	intergenic;ir	RPL29P29,LINC00433	-,+	pseudogene	221746;82149	intergenic;ir	RPL29P29,LINC00433	-,+	pseudogene	223657;80238
1717	chr2	153459917	+	chr2	153461433	-	N	N	Y	N	N	N	invers	na	NA	intronic;intr	FMNL2	+	protein_cod	0	intronic;intr	FMNL2	+	protein_cod	0
1718	chr21	27374154	+	chr21	27374703	-	Y	Y	Y	Y	Y	Y	invers	na	NA	intronic	APP	-	protein_cod	0	intronic	APP	-	protein_cod	0
1719	chr21	28020682	+	chr21	28021726	-	Y	N	Y	N	Y	N	invers	na	NA	intergenic	CYYR1,ADAMTS1	-,-	protein_cod	75079;187384	intergenic	CYYR1,ADAMTS1	-,-	protein_cod	76123;186340
1720	chr4	190585812	+	chr4	190613114	-	Y	Y	Y	Y	Y	Y	invers	na	NA	intergenic	RP11-462G22.1,RP11-	+,-	lincRNA,linc	3172;39223	intergenic	RP11-462G22.1,RP11-46	+,-	lincRNA,linc	30474;11921
1721	chr6	6312653	+	chr6	9433406	-	N	N	N	N	Y	N	invers	na	NA	intronic	F13A1	-	protein_cod	0	intergenic	RP11-354I10.1,OFCC1	-,-	lincRNA,pro	271396;163117
1722	chr6	130848185	+	chr6	130852295	-	Y	Y	Y	Y	Y	Y	invers	na	NA	intergenic	TMEM200A,Y_RNA	+,-	protein_cod	83977;46956	intergenic	TMEM200A,Y_RNA	+,-	protein_cod	88087;42846
1723	chr6	167411249	+	chr6	167411432	-	N	Y	N	N	N	N	invers	na	NA	ncRNA_intr	RP1-167A14.2	-	antisense	0	ncRNA_intr	RP1-167A14.2	-	antisense	0
1724	chr7	47654102	+	chr7	47654954	-	Y	N	Y	N	Y	N	invers	na	NA	intergenic	TNS3,AC095067.1	-,+	protein_cod	31946;7435	intergenic	TNS3,AC095067.1	-,+	protein_cod	32798;6583
1725	chr7	156387082	+	chr7	156394320	-	N	N	Y	N	N	N	invers	na	NA	ncRNA_intr	LINC01006	-	processed_	0	ncRNA_intr	LINC01006	-	processed_	0
1726	chr8	25912101	+	chr8	25912580	-	Y	N	N	N	N	N	invers	na	NA	intergenic	EBF2,RNA5SP258	-,+	protein_cod	9188;82295	intergenic;ir	EBF2,RNA5SP258	-,+	protein_cod	9667;81816
1727	chr9	89154557	+	chr9	89155930	-	N	N	N	Y	N	N	invers	na	NA	intergenic	RNU2-36P,RP11-395D	-,+	snRNA,lincF	116514;92505	intergenic	RNU2-36P,RP11-395D3.	-,+	snRNA,lincF	117887;91132
1728	chr1	12909375	+	chr1	12950025	-	Y	Y	Y	Y	N	N	invers_f	na	NA	upstream	HNRNPCL1	-	protein_cod	0	intergenic	PRAMEF4,PRAMEF10	-,-	protein_cod	4000;2702
1729	chr1	16407468	+	chr1	16408060	-	N	N	Y	N	N	N	invers_f	na	NA	intergenic	FAM131C,EPHA2	-,-	protein_cod	7347;43364	intergenic	FAM131C,EPHA2	-,-	protein_cod	7939;42772
1730	chr1	16407625	+	chr1	16408023	-	Y	N	N	N	N	N	invers_f	na	NA	intergenic	FAM131C,EPHA2	-,-	protein_cod	7504;43207	intergenic	FAM131C,EPHA2	-,-	protein_cod	7902;42809
1731	chr1	16407661	+	chr1	16407961	-	N	N	N	Y	N	N	invers_f	na	NA	intergenic	FAM131C,EPHA2	-,-	protein_cod	7540;43171	intergenic	FAM131C,EPHA2	-,-	protein_cod	7840;42871
1732	chr1	16407686	+	chr1	16408763	-	N	Y	N	N	N	N	invers_f	na	NA	intergenic	FAM131C,EPHA2	-,-	protein_cod	7565;43146	intergenic	FAM131C,EPHA2	-,-	protein_cod	8642;42069
1733	chr1	36733250	+	chr1	36734642	-	Y	Y	Y	Y	Y	Y	invers_f	na	NA	intronic	THRAP3	+	protein_cod	0	intronic	THRAP3	+	protein_cod	0
1734	chr1	44059296	+	chr1	44059886	-	Y	N	N	Y	Y	N	invers_f	na	NA	intronic	PTPRF	+	protein_cod	0	intronic	PTPRF	+	protein_cod	0
1735	chr1	74322173	+	chr1	161153042	-	N	N	Y	N	N	N	invers_f	na	NA	intergenic	RNA5SP50,RNU4ATA	-,+	rRNA,snRN	106867;27223	intergenic	B4GALT3,ADAMTS4	-,-	protein_cod	5755;1056
1736	chr1	93261904	+	chr1	93262741	-	N	Y	N	N	N	N	invers_f	na	NA	intergenic	EVI5,RPL5	-,+	protein_cod	3943;35678	intergenic	EVI5,RPL5	-,+	protein_cod	4780;34841
1737	chr1	167866781	+	chr1	225233279	-	Y	Y	N	N	Y	Y	invers_f	na	NA	intronic	ADCY10	-	protein_cod	0	intronic	DNAH14	+	protein_cod	0
1738	chr1	174312154	+	chr1	232869259	-	Y	Y	Y	Y	Y	Y	invers_f	na	NA	intronic	RABGAP1L	+	protein_cod	0	ncRNA_intr	RP11-66A2.2	-	lincRNA	0
1739	chr1	181043243	+	chr1	181043851	-	N	N	N	Y	N	N	invers_f	na	NA	intergenic	MR1,IER5	+,+	protein_cod	12169;14395	intergenic	MR1,IER5	+,+	protein_cod	12777;13787
1740	chr1	181043901	+	chr1	181044092	-	N	Y	N	N	Y	Y	invers_f	na	NA	intergenic	MR1,IER5	+,+	protein_cod	12827;13737	intergenic	MR1,IER5	+,+	protein_cod	13018;13546
1741	chr1	185963183	+	chr1	187106943	-	Y	Y	Y	Y	Y	N	invers_f	na	NA	intronic	HMCN1	+	protein_cod	0	ncRNA_intr	LINC01036	+	lincRNA	0
1742	chr1	187464828	+	chr1	187466727	-	N	Y	N	Y	N	Y	invers_f	na	NA	intergenic	RP11-445P19.3,RP11-	+,-	lincRNA,pse	15082;11142	intergenic	RP11-445P19.3,RP11-44	+,-	lincRNA,pse	16981;9243
1743	chr1	192799507	+	chr1	193043036	-	Y	Y	N	N	N	N	invers_f	na	NA	intergenic	RGS2,RN7SKP126	+,-	protein_cod	18104;45309	intronic	TROVE2	+	protein_cod	0
1744	chr1	237566104	+	chr1	237566208	-	Y	Y	Y	Y	Y	Y	invers_f	na	NA	intronic	RYR2	+	protein_cod	0	intronic	RYR2	+	protein_cod	0
1745	chr10	38376454	+	chr10	43055665	-	Y	N	Y	N	Y	Y	invers_f	na	NA	intergenic;ir	EIF3LP3,FXYD6P2	-,-	pseudogene	6649;4238	intergenic;ir	FXYD6P1,EIF3LP2	+,+	pseudogene	4493;6460
1746	chr10	39087718	+	chr10	39143879	-	N	N	N	N	Y	N	invers_f	na	NA	intergenic	RP11-96F8.1,NONE	+,NONE	pseudogene	11479;NONE	intergenic	RP11-96F8.1,NONE	+,NONE	pseudogene	67640;NONE
1747	chr10	127190677	+	chr10	127197223	-	N	N	N	N	N	N	invers_f	na	NA	intergenic	RPS27P18,TEX36-AS	-,+	pseudogene	28176;72263	intergenic	RPS27P18,TEX36-AS1	-,+	pseudogene	34722;65717
1748	chr11	389239	+	chr11	574285	-	N	N	Y	N	N	N	invers_f	na	NA	intergenic	B4GALNT4,PKP3	+,+	protein_cod	7123;3375	intergenic	MIR210HG,PHRF1	-,+	lincRNA,pro	5828;2201
1749	chr11	1620272	+	chr11	71237572	-	N	Y	N	N	N	Y	invers_f	na	NA	ncRNA_exc	KRTAP5-AS1	+	antisense	0	upstream	KRTAP5-7	+	protein_cod	0
1750	chr11	2154327	+	chr11	69526187	-	N	Y	N	N	Y	Y	invers_f	na	NA	exonic	IGF2	-	protein_cod	0	intergenic	FGF19,RP11-300I6.5	-,-	protein_cod	6777;25879
1751	chr11	9104550	+	chr11	83028903	-	N	N	N	Y	N	N	invers_f	na	NA	intronic	SCUBE2	-	protein_cod	0	ncRNA_intr	RP11-727A23.10	+	antisense	0
1752	chr11	18267989	+	chr11	18751702	-	Y	N	N	N	N	N	invers_f	na	NA	ncRNA_intr	SAA2-SAA4	-	processed_	0	ncRNA_intr	RP11-1081L13.4	+	antisense	0
1753	chr11	48388934	+	chr11	50039334	-	Y	N	N	N	N	N	invers_f	na	NA	upstream	OR4C5	-	protein_cod	0	intergenic	OR4C12,RP11-227P3.1	-,-	protein_cod	35263;20729
1754	chr11	55364519	+	chr11	55434496	-	N	Y	Y	N	Y	Y	invers_f	na	NA	intergenic;ir	OR4C16,OR4C11	+,-	protein_cod	23983;6311	downstream	OR4C6	+	protein_cod	0
1755	chr11	90421439	+	chr11	91809130	-	Y	N	N	N	N	N	invers_f	na	NA	ncRNA_intr	DISC1FP1	+	processed_	0	intergenic;ir	TUBB4BP4,RPL7AP57	-,+	pseudogene	125930;85142
1756	chr11	99690483	+	chr11	99691307	-	N	Y	N	Y	Y	Y	invers_f	na	NA	exonic	CNTN5	+	protein_cod	0	intronic	CNTN5	+	protein_cod	0
1757	chr12	10732131	+	chr12	10732296	-	N	N	N	Y	Y	Y	invers_f	na	NA	intergenic	RP11-291B21.2,KLRAI	+,-	lincRNA,pse	21483;8946	intergenic	RP11-291B21.2,KLRAP1	+,-	lincRNA,pse	21648;8781
1758	chr12	30404007	+	chr12	30501742	-	Y	Y	N	Y	Y	N	invers_f	na	NA	ncRNA_intr	RP11-776A13.3	-	lincRNA	0	intergenic;ir	RP11-155I9.1,RP11-776A	+,+	lincRNA,pse	27192;106110

1759	chr12	30404042	+	chr12	30501742	-	N	N	Y	N	N	N	invers_f	na	NA	ncRNA_intr	RP11-776A13.3	-	lincRNA	0	intergenic;ir	RP11-155I9.1,RP11-776	+,+	lincRNA,pse	27192;106110
1760	chr12	50182140	+	chr12	50182613	-	Y	N	N	N	N	N	invers_f	na	NA	intergenic	LSM6P2,NCKAP5L	+,-	pseudogene	17221;2789	intergenic	LSM6P2,NCKAP5L	+,-	pseudogene	17694;2316
1761	chr12	50182498	+	chr12	50182979	-	N	N	N	Y	N	N	invers_f	na	NA	intergenic	LSM6P2,NCKAP5L	+,-	pseudogene	17579;2431	intergenic	LSM6P2,NCKAP5L	+,-	pseudogene	18060;1950
1762	chr12	50182595	+	chr12	50183005	-	N	N	Y	N	N	Y	invers_f	na	NA	intergenic	LSM6P2,NCKAP5L	+,-	pseudogene	17676;2334	intergenic	LSM6P2,NCKAP5L	+,-	pseudogene	18086;1924
1763	chr12	50182605	+	chr12	50182963	-	N	Y	N	N	N	N	invers_f	na	NA	intergenic	LSM6P2,NCKAP5L	+,-	pseudogene	17686;2324	intergenic	LSM6P2,NCKAP5L	+,-	pseudogene	18044;1966
1764	chr12	50182607	+	chr12	50182884	-	N	N	N	N	Y	N	invers_f	na	NA	intergenic	LSM6P2,NCKAP5L	+,-	pseudogene	17688;2322	intergenic	LSM6P2,NCKAP5L	+,-	pseudogene	17965;2045
1765	chr12	104382542	+	chr12	125801164	-	N	N	N	N	N	Y	invers_f	na	NA	UTR3	TDG	+	protein_cod	0	intergenic	NONE,TMEM132B	NONE,+	NONE,prote	NONE;9998
1766	chr12	133141109	+	chr12	133344190	-	Y	Y	Y	N	N	N	invers_f	na	NA	intronic;intro	FBRSL1	+	protein_cod	0	intergenic;ir	ANKLE2,GOLGA3	-,-	protein_cod	5716;1305
1767	chr13	20124074	+	chr13	21848635	-	N	N	N	N	Y	N	invers_f	na	NA	intergenic	TPTE2,MRPS31P2	-,-	protein_cod	13171;2637	ncRNA_intr	ESRRAP2	+	pseudogene	0
1768	chr13	20124109	+	chr13	21848635	-	N	N	Y	N	N	N	invers_f	na	NA	intergenic	TPTE2,MRPS31P2	-,-	protein_cod	13206;2602	ncRNA_intr	ESRRAP2	+	pseudogene	0
1769	chr13	60870092	+	chr13	66279828	-	N	N	Y	N	N	N	invers_f	na	NA	intergenic	TARDBPP2,TDRD3	+,+	pseudogene	19952;100499	intergenic	STARP1,HNRNPA3P5	-,-	pseudogene	394743;82236
1770	chr13	89110969	+	chr13	89112603	-	Y	N	N	N	N	N	invers_f	na	NA	intergenic	RPL29P29,LINC00433	-,+	pseudogene	221745;82150	intergenic	RPL29P29,LINC00433	-,+	pseudogene	223379;80516
1771	chr13	89110988	+	chr13	89112602	-	N	N	N	N	N	Y	invers_f	na	NA	intergenic	RPL29P29,LINC00433	-,+	pseudogene	221764;82131	intergenic	RPL29P29,LINC00433	-,+	pseudogene	223378;80517
1772	chr14	21332108	+	chr14	22226321	-	N	N	N	N	N	Y	invers_f	na	NA	intergenic	RNASE1,RP11-219E7.	-,-	protein_cod	60671;6329	intergenic	RPL4P1,TRAV6	+,+	pseudogene	6491;10408
1773	chr14	24098368	+	chr14	97971495	-	Y	Y	Y	Y	Y	Y	invers_f	na	NA	upstream	DHRS2	+	protein_cod	0	ncRNA_intr	CTD-2506J14.1	+	lincRNA	0
1774	chr14	65842536	+	chr14	65843134	-	Y	Y	Y	N	N	Y	invers_f	na	NA	intergenic;ir	MIR4708,FUT8	-,+	miRNA,prot	40635;34774	intergenic;ir	MIR4708,FUT8	-,+	miRNA,prot	41233;34176
1775	chr14	70945242	+	chr14	90912038	-	Y	Y	N	Y	Y	Y	invers_f	na	NA	ncRNA_intr	RP11-486O13.4,ADAM	-,-	lincRNA,linc	0	intergenic;ir	CALM1,RP11-1078H9.1	+,-	protein_cod	37433;6369
1776	chr14	93926007	+	chr14	93926766	-	N	Y	N	N	N	N	invers_f	na	NA	intronic	UNC79	+	protein_cod	0	intronic	UNC79	+	protein_cod	0
1777	chr15	31033857	+	chr15	31956256	-	N	N	N	Y	N	N	invers_f	na	NA	intergenic	ARHGAP11B,GOLGA8	+,+	protein_cod	56047;49919	intronic	OTUD7A	-	protein_cod	0
1778	chr15	41853488	+	chr15	76553757	-	N	N	Y	N	N	N	invers_f	na	NA	exonic;exon	TYRO3	+	protein_cod	0	ncRNA_exo	TYRO3P	-	pseudogene	0
1779	chr15	75624536	+	chr15	78241040	-	Y	Y	Y	N	N	N	invers_f	na	NA	intergenic	ANP32BP1,COMMD4	-,+	pseudogene	9502;3696	intergenic	RP11-114H24.4,RP11-11	-,-	pseudogene	4116;5377
1780	chr15	75624591	+	chr15	78240567	-	N	N	N	Y	N	Y	invers_f	na	NA	intergenic	ANP32BP1,COMMD4	-,+	pseudogene	9557;3641	intergenic	RP11-114H24.4,RP11-11	-,-	pseudogene	3643;5850
1781	chr15	75624630	+	chr15	78240562	-	N	N	N	N	Y	N	invers_f	na	NA	intergenic	ANP32BP1,COMMD4	-,+	pseudogene	9596;3602	intergenic	RP11-114H24.4,RP11-11	-,-	pseudogene	3638;5855
1782	chr15	78276930	+	chr15	79060392	-	Y	N	N	N	N	N	invers_f	na	NA	ncRNA_exo	ADAMTS7P3	+	pseudogene	0	intronic	ADAMTS7	-	protein_cod	0
1783	chr15	78276964	+	chr15	79060427	-	N	N	N	N	Y	Y	invers_f	na	NA	ncRNA_intr	ADAMTS7P3	+	pseudogene	0	intronic	ADAMTS7	-	protein_cod	0
1784	chr15	78277288	+	chr15	79060354	-	N	Y	Y	N	N	N	invers_f	na	NA	ncRNA_intr	ADAMTS7P3	+	pseudogene	0	intronic	ADAMTS7	-	protein_cod	0
1785	chr16	21594442	+	chr16	22710751	-	N	Y	Y	Y	Y	Y	invers_f	na	NA	intergenic	CTB-31N19.2,SCARN	-,+	pseudogene	32748;4506	intergenic	RN7SL245P,HS3ST2	-,+	misc_RNA,p	62686;114747
1786	chr16	32354173	+	chr16	33512831	-	N	Y	Y	N	N	Y	invers_f	na	NA	intergenic	RP11-17M15.2,RP11-1	-,-	pseudogene	32297;14129	intergenic	BMS1P8,ENPP7P13	-,+	pseudogene	11860;59057
1787	chr16	47219290	+	chr16	47715630	-	N	Y	N	N	N	N	invers_f	na	NA	intronic	ITFG1	-	protein_cod	0	intronic	PHKB	+	protein_cod	0
1788	chr16	48650446	+	chr16	48650966	-	N	Y	N	N	N	N	invers_f	na	NA	intergenic	N4BP1,CTC-527H23.2	-,+	protein_cod	6326;3784	intergenic	N4BP1,CTC-527H23.2	-,+	protein_cod	6846;3264
1789	chr16	48650805	+	chr16	48651345	-	N	N	Y	N	N	N	invers_f	na	NA	intergenic	N4BP1,CTC-527H23.2	-,+	protein_cod	6685;3425	intergenic	N4BP1,CTC-527H23.2	-,+	protein_cod	7225;2885
1790	chr17	15641441	+	chr17	18541849	-	N	N	N	Y	N	N	invers_f	na	NA	ncRNA_intr	AC005324.6	-	antisense	0	intronic	TBC1D28	-	protein_cod	0
1791	chr17	36350387	+	chr17	36406169	-	Y	Y	Y	Y	N	Y	invers_f	na	NA	intronic;intro	RP11-1407O15.2,TBC	-,-	protein_cod	0	intronic;intro	RP11-1407O15.2	-	protein_cod	0
1792	chr17	39240659	+	chr17	39296495	-	N	N	N	Y	N	N	invers_f	na	NA	exonic	KRTAP4-7	+	protein_cod	0	exonic	KRTAP4-6	-	protein_cod	0
1793	chr17	73249717	+	chr17	74606941	-	N	N	Y	N	N	N	invers_f	na	NA	intronic	GGA3	-	protein_cod	0	intergenic	RP11-666A8.11,ST6GAL	-,-	pseudogene	15087;13902
1794	chr19	15822375	+	chr19	16015182	-	N	Y	N	N	N	N	invers_f	na	NA	intergenic;ir	CYP4F12,OR10H2	+,+	protein_cod	14391;16459	intergenic;ir	AC005336.5,AC005336.4	-,-	pseudogene	1134;6210
1795	chr19	21495759	+	chr19	21708833	-	N	N	Y	N	N	Y	invers_f	na	NA	intronic	ZNF708	-	protein_cod	0	intronic	ZNF429	+	protein_cod	0
1796	chr19	44915513	+	chr19	44962408	-	N	Y	N	N	N	N	invers_f	na	NA	ncRNA_intr	CTC-512J12.4	-	processed_	0	intergenic	ZNF229,ZNF285B	-,+	protein_cod	9642;9450
1797	chr2	87586698	+	chr2	91740722	-	N	N	N	N	Y	N	invers_f	na	NA	upstream	AC068279.2	-	pseudogene	0	ncRNA_exo	AC018696.7	+	pseudogene	0
1798	chr2	87642508	+	chr2	90482241	-	N	Y	N	N	N	N	invers_f	na	NA	intergenic	AC068279.3,LINC0015	-,+	pseudogene	34618;112379	intergenic	CH17-132F21.1,AC2332	+,-	protein_cod	23570;30254
1799	chr2	87644971	+	chr2	90482245	-	Y	N	N	N	N	N	invers_f	na	NA	intergenic	AC068279.3,LINC0015	-,+	pseudogene	37081;109916	intergenic	CH17-132F21.1,AC2332	+,-	protein_cod	23574;30250
1800	chr2	97932710	+	chr2	98107256	-	N	Y	N	N	N	N	invers_f	na	NA	intergenic	ANKRD36,IGKV1OR2-	+,-	protein_cod	2452;54621	intergenic	AC159540.2,ANKRD36B	+,-	lincRNA,pse	6848;1722
1801	chr2	116978297	+	chr2	116982309	-	Y	Y	N	Y	Y	Y	invers_f	na	NA	intergenic	DPP10,RP11-338I24.1	+,-	protein_cod	374969;466497	intergenic	DPP10,RP11-338I24.1	+,-	protein_cod	378981;462485
1802	chr2	129703594	+	chr2	129722265	-	N	Y	N	N	N	N	invers_f	na	NA	intergenic	AC012451.1,AC06848	-,-	lincRNA,linc	364444;117825	intergenic	AC012451.1,AC068483.1	-,-	lincRNA,linc	383115;99154
1803	chr2	129703664	+	chr2	129722844	-	N	N	N	Y	N	N	invers_f	na	NA	intergenic	AC012451.1,AC06848	-,-	lincRNA,linc	364514;117755	intergenic	AC012451.1,AC068483.1	-,-	lincRNA,linc	383694;98575
1804	chr2	129703701	+	chr2	129722216	-	N	N	Y	N	N	N	invers_f	na	NA	intergenic	AC012451.1,AC06848	-,-	lincRNA,linc	364551;117718	intergenic	AC012451.1,AC068483.1	-,-	lincRNA,linc	383066;99203
1805	chr2	129703832	+	chr2	129722238	-	N	N	N	N	N	Y	invers_f	na	NA	intergenic	AC012451.1,AC06848	-,-	lincRNA,linc	364682;117587	intergenic	AC012451.1,AC068483.1	-,-	lincRNA,linc	383088;99181
1806	chr2	131886772	+	chr2	131887113	-	N	N	Y	N	N	N	invers_f	na	NA	intronic	PLEKHB2	+	protein_cod	0	intronic	PLEKHB2	+	protein_cod	0
1807	chr2	153459917	+	chr2	153461433	-	N	N	N	Y	N	Y	invers_f	na	NA	intronic;intro	FMNL2	+	protein_cod	0	intronic;intro	FMNL2	+	protein_cod	0
1808	chr2	228075134	+	chr2	228076143	-	N	N	N	N	N	Y	invers_f	na	NA	intronic;intro	COL4A3	+	protein_cod	0	intronic	COL4A3	+	protein_cod	0
1809	chr2	228075149	+	chr2	228075928	-	N	N	N	N	Y	N	invers_f	na	NA	intronic	COL4A3	+	protein_cod	0	intronic	COL4A3	+	protein_cod	0
1810	chr2	228075279	+	chr2	228075926	-	N	Y	N	N	N	N	invers_f	na	NA	intronic	COL4A3	+	protein_cod	0	intronic	COL4A3	+	protein_cod	0
1811	chr21	23626205	+	chr21	29293560	-	Y	N	Y	N	Y	Y	invers_f	na	NA	ncRNA_intr	AP000705.7	+	lincRNA	0	intergenic	LINC00113,AJ006995.3	+,+	lincRNA,linc	170008;33680
1812	chr21	25764454	+	chr21	25764658	-	N	Y	N	N	N	N	invers_f	na	NA	intergenic	AP000469.2,AP00047	-,+	lincRNA,linc	70764;36600	intergenic	AP000469.2,AP000476.1	-,+	lincRNA,linc	70968;36396
1813	chr21	28020677	+	chr21	28023462	-	N	N	N	N	N	Y	invers_f	na	NA	intergenic	CYYR1,ADAMTS1	-,-	protein_cod	75074;187389	intergenic	CYYR1,ADAMTS1	-,-	protein_cod	77859;184604
1814	chr22	17037101	+	chr22	22613416	-	N	N	N	N	N	Y	invers_f	na	NA	intergenic	KB-67B5.17,KB-67B5.	+,+	lincRNA,pse	28974;15816	intergenic	VPREB1,LL22NC03-2H8	+,-	protein_cod	13489;24896
1815	chr22	37355469	+	chr22	48075284	-	N	N	N	N	N	Y	invers_f	na	NA	intergenic	CSF2RBP1,LL22NC01	-,+	pseudogene	5705;5820	ncRNA_intr	RP11-191L9.4	+	lincRNA	0
1816	chr3	22751278	+	chr3	22782547	-	N	N	Y	N	N	N	invers_f	na	NA	intergenic	AC092421.1,RP11-103	-,-	pseudogene	39076;101603	intergenic	AC092421.1,RP11-1037D	-,-	pseudogene	70345;70334
1817	chr3	45174956	+	chr3	124331321	-	Y	N	N	N	Y	N	invers_f	na	NA	intronic	CDCP1	-	protein_cod	0	intronic	KALRN	+	protein_cod	0
1818	chr3	122466069	+	chr3	122499616	-	N	N	Y	N	N	N	invers_f	na	NA	intronic	HSPBAP1	-	protein_cod	0	intronic	HSPBAP1	-	protein_cod	0
1819	chr3	122466109	+	chr3	122499930	-	N	N	N	Y	N	N	invers_f	na	NA	intronic	HSPBAP1	-	protein_cod	0	intronic	HSPBAP1	-	protein_cod	0
1820	chr3	122466535	+	chr3	122499620	-	N	Y	N	N	N	N	invers_f	na	NA	intronic	HSPBAP1	-	protein_cod	0	intronic	HSPBAP1	-	protein_cod	0
1821	chr3	122466614	+	chr3	122499673	-	N	N	N	N	N	Y	invers_f	na	NA	intronic	HSPBAP1	-	protein_cod	0	intronic	HSPBAP1	-	protein_cod	0
1822	chr3	131708252	+	chr3	131708413	-	N	N	N	Y	Y	N	invers_f	na	NA	intronic;intro	CPNE4	-	protein_cod	0	intronic	CPNE4	-	protein_cod	0
1823	chr4	4006778	+	chr4	9641395	-	Y	Y	Y	Y	Y	Y	invers_f	na	NA	intergenic	ENPP7P9,AC116562.2	-,+	pseudogene	37391;32033	intergenic	AC097493.1,ENPP7P11	-,+	miRNA,pseu	39344;37537
1824	chr4	28857249	+	chr4	28960289	-	N	N	N	N	Y	N	invers_f	na	NA	intergenic	MESTP3,RP11-292B1.	-,+	pseudogene	31416;140897	intergenic	MESTP3,RP11-292B1.2	-,+	pseudogene	134456;37857
1825	chr4	28857267	+	chr4	28960537	-	N	N	N	Y	N	N	invers_f	na	NA	intergenic	MESTP3,RP11-292B1.	-,+	pseudogene	31434;140879	intergenic	MESTP3,RP11-292B1.2	-,+	pseudogene	134704;37609
1826	chr4	70052434	+	chr4	70167809	-	Y	N	N	N	N	N	invers_f	na	NA	ncRNA_intr	RP11-704M14.1	+	antisense	0	intergenic	UGT2B28,RP11-618I10.4	+,-	protein_cod	7041;4378
1827	chr4	70052493	+	chr4	70167623	-	N	N	N	N	N	Y	invers_f	na	NA	ncRNA_intr	RP11-704M14.1	+	antisense	0	intergenic	UGT2B28,RP11-618I10.4	+,-	protein_cod	6855;4564
1828	chr4	70086216	+	chr4	70249423	-	Y	N	N	N	N	Y	invers_f	na	NA	intergenic	RP11-704M14.1,RP11-	+,+	antisense,ps	3732;22130	intergenic	RP11-618I10.2,RP11-618	+,+	pseudogene	25528;3875
1829	chr4	78178693	+	chr4	79036211	-	N	N	N	Y	N	N	invers_f	na	NA	intergenic	CTB-179I1.3,CTB-179I	-,-	pseudogene	40662;53857	intronic	FRAS1	+	protein_cod	0
1830	chr4	78905965	+	chr4	190684480	-	N	N	N	N	Y	N	invers_f	na	NA	intergenic;ir	HMGB1P44,HNRNPA1	+,-	pseudogene	20300;3049	intergenic;ir	RNU1-51P,AF146191.4	-,-	snRNA,lincR	53612;17010
1831	chr4	163578583	+	chr4	163579619	-	Y	Y	Y	Y	N	Y	invers_f	na	NA	intergenic;ir	TOMM22P4,AC005150	+,+	pseudogene	135390;83237	intergenic;ir	TOMM22P4,AC005150.1	+,+	pseudogene	136426;82201
1832	chr4	168109161	+	chr4	168115264	-	N	Y	N	Y	N	N	invers_f	na	NA	intronic	SPOCK3	-	protein_cod	0	intronic	SPOCK3	-	protein_cod	0
1833	chr4	188426860	+	chr4	190537960	-	N	Y	N	N	N	N	invers_f	na	NA	intergenic	RP11-91J3.1,RP11-23	-,-	pseudogene	61408;10447	intergenic	HSP90AA4P,RP11-462G	+,+	pseudogene	141614;42799
1834	chr4	188427383	+	chr4	190537635	-	N	N	Y	N	N	N	invers_f	na	NA	intergenic	RP11-91J3.1,RP11-23	-,-	pseudogene	61931;9924	intergenic	HSP90AA4P,RP11-462G	+,+	pseudogene	141289;43124
1835	chr5	143406083	+	chr5	143406745	-	Y	Y	Y	Y	Y	Y	invers_f	na	NA	intergenic	CTB-57H20.1,RN7SL8	+,+	sense_over	197746;114361	intergenic	CTB-57H20.1,RN7SL87P	+,+	sense_over	198408;113699
1836	chr6	25647708	+	chr6	25647927	-	N	Y	N	N	Y	Y	invers_f	na	NA	intergenic	LRRC16A,SCGN	+,+	protein_cod	26950;4756	intergenic	LRRC16A,SCGN	+,+	protein_cod	27169;4537
1837	chr6	67492238	+	chr6	67492726	-	N	Y	N	N	N	N	invers_f	na	NA	intergenic	RNU7-66P,AL590874.1	-,-	snRNA,miR	53441;367164	intergenic	RNU7-66P,AL590874.1	-,-	snRNA,miR	53929;366676
1838	chr6	89408255	+	chr6	94728966	-	N	N	N	Y	N	N	invers_f	na	NA	intronic	RNGTT	-	protein_cod	0	intergenic	RP11-524K14.1,RP3-463	+,+	lincRNA,linc	129838;144672
1839	chr6	89408306	+	chr6	94728992	-	N	N	Y	N	N	N	invers_f	na	NA	intronic	RNGTT	-	protein_cod	0	intergenic	RP11-524K14.1,RP3-463	+,+	lincRNA,linc	129864;144646
1840	chr6	112950565	+	chr6	114091216	-	Y	Y	Y	Y	Y	Y	invers_f	na	NA	intergenic	RP1-159M24.1,RP11-5	-,+	pseudogene	11483;196576	intergenic	AL357519.1,U3	+,-	miRNA,snol	65091;11302
1841	chr6	157559467	+	chr6	157641237	-	N	N	N	Y	N	N	invers_f	na	NA	intergenic	ARID1B,TMEM242	+,-	protein_cod	27554;150951	intergenic	ARID1B,TMEM242	+,-	protein_cod	109324;69181
1842	chr6	170429014	+	chr6	170429353	-	N	N	Y	N	N	N	invers_f	na	NA	intergenic	RP1-182D15.2,RP11-3	-,+	lincRNA,ant	218816;48727	intergenic	RP1-182D15.2,RP11-302	-,+	lincRNA,ant	219155;48388

1843	chr7	23815882	+	chr7	23838271	-	N	N	N	N	N	Y	invers_f	na	NA	intronic	STK31	+	protein_cod	0	intronic	STK31	+	protein_cod	0
1844	chr7	23816021	+	chr7	23837611	-	Y	N	N	N	N	N	invers_f	na	NA	intronic	STK31	+	protein_cod	0	intronic	STK31	+	protein_cod	0
1845	chr7	28877846	+	chr7	139205831	-	Y	Y	N	N	Y	N	invers_f	na	NA	intergenic	CREB5,TRIL	+,-	protein_cod	12335;115128	intergenic	RP4-701O16.5,CLEC2L	+,+	pseudogene	17273;2771
1846	chr7	34857498	+	chr7	37692886	-	Y	N	N	N	N	N	invers_f	na	NA	ncRNA_intro	NPSR1-AS1	-	processed_	0	intergenic;in	NECAP1P1,GPR141	-,+	pseudogene	66954;30514
1847	chr7	34857749	+	chr7	37692886	-	N	N	N	Y	N	N	invers_f	na	NA	ncRNA_intro	NPSR1-AS1	-	processed_	0	intergenic;in	NECAP1P1,GPR141	-,+	pseudogene	66954;30514
1848	chr7	45887436	+	chr7	100908661	-	Y	Y	Y	Y	Y	N	invers_f	na	NA	intergenic	RNU6-326P,AC096582	+,-	snRNA,prot	4097;15248	intergenic	FIS1,RNU6-1104P	-,-	protein_cod	13064;4558
1849	chr7	47653664	+	chr7	47654992	-	N	N	N	Y	N	N	invers_f	na	NA	intergenic	TNS3,AC095067.1	-,+	protein_cod	31508;7873	intergenic	TNS3,AC095067.1	-,+	protein_cod	32836;6545
1850	chr7	57555150	+	chr7	64954550	-	N	N	N	Y	N	N	invers_f	na	NA	intergenic	RP11-1324A7.2,NCOR	+,+	pseudogene	9549;104350	intergenic	RP11-667F9.2,RP11-328	+,-	lincRNA,pse	26199;19136
1851	chr7	67635773	+	chr7	98083201	-	Y	Y	Y	Y	N	N	invers_f	na	NA	ncRNA_intro	RP5-945F2.3	-	lincRNA	0	downstream	AC091654.7	-	pseudogene	0
1852	chr7	70420968	+	chr7	70438886	-	Y	Y	Y	Y	Y	Y	invers_f	na	NA	intergenic	RP11-575M4.1,WBSCI	+,+	pseudogene	117969;176187	intergenic	RP11-575M4.1,WBSCR1	+,+	pseudogene	135887;158269
1853	chr7	91110975	+	chr7	91137129	-	N	N	N	Y	N	N	invers_f	na	NA	ncRNA_intro	RP11-142A5.1,RP11-1	-,+	lincRNA,linc	0	ncRNA_intro	RP11-142A5.1,RP11-115	-,+	lincRNA,linc	0
1854	chr7	91111002	+	chr7	91137139	-	N	N	N	N	N	Y	invers_f	na	NA	ncRNA_intro	RP11-142A5.1,RP11-1	-,+	lincRNA,linc	0	ncRNA_intro	RP11-142A5.1,RP11-115	-,+	lincRNA,linc	0
1855	chr7	124663200	+	chr7	147571358	-	N	Y	N	N	N	N	invers_f	na	NA	ncRNA_intro	POT1-AS1	+	antisense	0	intronic	CNTNAP2	+	protein_cod	0
1856	chr7	134506345	+	chr7	134520900	-	N	Y	Y	Y	N	Y	invers_f	na	NA	intronic	CALD1	+	protein_cod	0	intronic	CALD1	+	protein_cod	0
1857	chr7	142031078	+	chr7	142247826	-	N	N	N	N	Y	N	invers_f	na	NA	upstream	TRBV7-1	+	TR_V_gene	0	upstream	TRBV7-3	-	TR_V_gene	0
1858	chr7	142034597	+	chr7	142276197	-	Y	Y	Y	Y	Y	Y	invers_f	na	NA	intergenic	TRBV7-1,TRBV4-2	+,+	TR_V_gene	2070;10655	intergenic	TRBV8-1,TRBV19	-,+	TR_V_pseu	2863;50166
1859	chr7	142048164	+	chr7	142251708	-	N	N	N	N	Y	N	invers_f	na	NA	intergenic	TRBV4-2,TRBV7-8	+,-	TR_V_gene	2348;51291	upstream	TRBV6-4	-	TR_V_gene	0
1860	chr7	149736275	+	chr7	153757820	-	N	N	N	N	Y	N	invers_f	na	NA	intergenic	RP11-728K20.2,RP11-	+,+	lincRNA,linc	34062;1335	intronic	DPP6	+	protein_cod	0
1861	chr7	156387082	+	chr7	156394320	-	Y	N	Y	Y	Y	N	invers_f	na	NA	ncRNA_intro	LINC01006	-	processed_	0	ncRNA_intro	LINC01006	-	processed_	0
1862	chr8	25911942	+	chr8	25913025	-	N	N	N	N	N	Y	invers_f	na	NA	intergenic	EBF2,RNA5SP258	-,+	protein_cod	9029;82454	intergenic	EBF2,RNA5SP258	-,+	protein_cod	10112;81371
1863	chr8	25912025	+	chr8	25912580	-	N	N	Y	N	N	N	invers_f	na	NA	intergenic	EBF2,RNA5SP258	-,+	protein_cod	9112;82371	intergenic;in	EBF2,RNA5SP258	-,+	protein_cod	9667;81816
1864	chr8	26969096	+	chr8	26969218	-	N	N	N	N	Y	N	invers_f	na	NA	intergenic	RP11-521M14.2,AC09	+,+	protein_cod	24330;36125	intergenic	RP11-521M14.2,AC0901	+,+	protein_cod	24452;36003
1865	chr8	68667474	+	chr8	71768187	-	N	N	N	N	N	Y	invers_f	na	NA	intergenic	CPA6,NDUFS5P6	-,+	protein_cod	8854;93805	intergenic	Y_RNA,RP11-326E22.1	-,+	misc_RNA,a	74944;299505
1866	chr8	68667603	+	chr8	71768023	-	N	N	N	Y	N	N	invers_f	na	NA	intergenic	CPA6,NDUFS5P6	-,+	protein_cod	8983;93676	intergenic	Y_RNA,RP11-326E22.1	-,+	misc_RNA,a	74780;299669
1867	chr9	2279611	+	chr9	3812868	-	N	Y	N	N	N	N	invers_f	na	NA	intergenic	RN7SL592P,RP11-125	-,-	misc_RNA,a	50370;143091	intergenic	RP11-509J21.3,GLIS3	+,-	lincRNA,pro	121054;11259
1868	chr9	66462153	+	chr9	66474386	-	N	N	N	N	Y	Y	invers_f	na	NA	ncRNA_exo	RP11-262H14.1	+	lincRNA	0	downstream	AL512625.1	+	pseudogene	0
1869	chr9	66491185	+	chr9	68377525	-	N	N	N	N	N	Y	invers_f	na	NA	intergenic	AL512625.1,RP11-262	+,-	pseudogene	17512;2387	intergenic	PTGER4P3,RP11-764K9	-,-	pseudogene	5648;20353
1870	chr9	68421834	+	chr9	68429244	-	N	Y	Y	Y	N	Y	invers_f	na	NA	intergenic	MIR4477A,RP11-764K	+,-	miRNA,pseu	6446;6027	ncRNA_intro	RP11-764K9.4	-	pseudogene	0
1871	chrX	27031046	+	chrX	27031893	-	N	N	Y	Y	N	N	invers_f	na	NA	intergenic	RNU1-142P,RP11-268	+,-	snRNA,lincF	222643;29978	intergenic	RNU1-142P,RP11-268G1	+,-	snRNA,lincF	223490;29131
1872	chrX	51280359	+	chrX	51965838	-	Y	N	N	N	N	N	invers_f	na	NA	intergenic	RP11-56H2.2,CENPVF	-,-	lincRNA,pse	26502;80602	intergenic	RP11-363G10.2,XAGE2E	+,+	protein_cod	19480;146335
1873	chrX	69934767	+	chrX	69950083	-	N	N	N	Y	N	N	invers_f	na	NA	intronic	TEX11	-	protein_cod	0	intronic	TEX11	-	protein_cod	0
1874	chrX	69935312	+	chrX	69949500	-	N	N	N	N	N	Y	invers_f	na	NA	intronic	TEX11	-	protein_cod	0	intronic	TEX11	-	protein_cod	0
1875	chrX	76653692	+	chrX	140499290	-	Y	Y	N	N	Y	Y	invers_f	na	NA	intergenic	BX682235.1,FGF16	+,+	pseudogene	51910;55956	intergenic	GS1-164F24.1,SPANXA2	+,+	pseudogene	24019;91553
1876	chr1	16408763	-	chr1	16407686	+	Y	N	Y	Y	Y	Y	invers_r	na	NA	intergenic	FAM131C,EPHA2	-,-	protein_cod	8642;42069	intergenic	FAM131C,EPHA2	-,-	protein_cod	7565;43146
1877	chr1	17027884	-	chr1	6507221	+	N	N	N	N	Y	N	invers_r	na	NA	ncRNA_intro	ESPNP	-	pseudogene	0	intronic	ESPN	+	protein_cod	0
1878	chr1	17236159	-	chr1	16830442	+	N	N	N	N	N	Y	invers_r	na	NA	intergenic	RP11-108M9.6,CROC	-,+	lincRNA,pro	4888;12286	intergenic	CROCCP3,RNU1-1	-,-	pseudogene	4690;10175
1879	chr1	30878819	-	chr1	29720344	+	N	N	N	Y	N	Y	invers_r	na	NA	intergenic;in	RP3-357I16.1,RP4-59	+,-	pseudogene	179357;3588	intergenic	RP3-437I16.1,AL139151	-,+	lincRNA,miF	43718;38649
1880	chr1	30878819	-	chr1	30878513	+	Y	Y	N	Y	Y	Y	invers_r	na	NA	intergenic;in	RP3-357I16.1,RP4-59	+,-	pseudogene	179357;3588	intergenic	RP3-357I16.1,RP4-591L	+,-	pseudogene	179051;3894
1881	chr1	30879252	-	chr1	29720380	+	N	Y	N	Y	N	Y	invers_r	na	NA	intergenic	RP3-357I16.1,RP4-59	+,-	pseudogene	179790;3155	intergenic	RP3-437I16.1,AL139151	-,+	lincRNA,miF	43754;38613
1882	chr1	81661371	-	chr1	81660357	+	N	N	N	Y	N	N	invers_r	na	NA	intergenic;in	RPL7P10,RP3-445O1	-,+	pseudogene	96681;12882	intergenic;in	RPL7P10,RP3-445O10.1	-,+	pseudogene	95667;13896
1883	chr1	147775456	-	chr1	147752758	+	Y	Y	Y	Y	Y	Y	invers_r	na	NA	intergenic	RP11-495P10.5,RP11-	+,-	lincRNA,pse	5793;14343	ncRNA_intro	RP11-495P10.6	-	lincRNA	0
1884	chr1	149215463	-	chr1	17231355	+	Y	N	Y	N	N	N	invers_r	na	NA	upstream	RNU1-92P	-	snRNA	0	upstream	RP11-108M9.6	-	lincRNA	0
1885	chr1	149215898	-	chr1	17231095	+	N	Y	N	N	N	N	invers_r	na	NA	upstream	RNU1-92P	-	snRNA	0	ncRNA_exo	RP11-108M9.6	-	lincRNA	0
1886	chr1	149217436	-	chr1	17228765	+	N	N	N	N	N	Y	invers_r	na	NA	intergenic	RNU1-92P,RNVU1-18	-,-	snRNA,snR	2136;6622	intergenic;in	RNU1-2,RP11-108M9.6	+,-	snRNA,lincF	6127;2069
1887	chr1	149217483	-	chr1	17228789	+	N	N	Y	N	Y	N	invers_r	na	NA	intergenic	RNU1-92P,RNVU1-18	-,-	snRNA,snR	2183;6575	intergenic	RNU1-2,RP11-108M9.6	+,-	snRNA,lincF	6151;2045
1888	chr1	149217533	-	chr1	17228765	+	Y	N	N	N	N	N	invers_r	na	NA	intergenic	RNU1-92P,RNVU1-18	-,-	snRNA,snR	2233;6525	intergenic;in	RNU1-2,RP11-108M9.6	+,-	snRNA,lincF	6127;2069
1889	chr1	149220851	-	chr1	17225522	+	Y	Y	N	Y	Y	N	invers_r	na	NA	intergenic	RNU1-92P,RNVU1-18	-,-	snRNA,snR	5551;3207	intergenic	RNU1-2,RP11-108M9.6	+,-	snRNA,lincF	2884;5312
1890	chr1	165552835	-	chr1	165552432	+	N	N	Y	N	N	N	invers_r	na	NA	intergenic	RP11-306I11.1,RP11-3C	+,+	pseudogene	1122;14865	downstream	RP11-306I1.1	+	pseudogene	0
1891	chr1	167175201	-	chr1	113206277	+	N	N	N	N	Y	Y	invers_r	na	NA	intergenic	RP11-277B15.2,RP11-	-,-	antisense,ar	10172;13867	intronic	CAPZA1	+	protein_cod	0
1892	chr1	170251339	-	chr1	166676095	+	N	N	N	N	N	Y	invers_r	na	NA	ncRNA_intro	LINC01142	-	lincRNA	0	intergenic	FMO10P,RPL4P2	+,+	pseudogene	24662;40521
1893	chr1	181044092	-	chr1	181043901	+	N	N	N	Y	N	N	invers_r	na	NA	intergenic;in	MR1,IER5	+,+	protein_cod	13018;13546	intergenic;in	MR1,IER5	+,+	protein_cod	12827;13737
1894	chr1	181044529	-	chr1	181044089	+	N	Y	N	N	Y	N	invers_r	na	NA	intergenic	MR1,IER5	+,+	protein_cod	13455;13109	intergenic	MR1,IER5	+,+	protein_cod	13015;13549
1895	chr1	181044540	-	chr1	181044161	+	N	N	N	N	N	Y	invers_r	na	NA	intergenic	MR1,IER5	+,+	protein_cod	13466;13098	intergenic	MR1,IER5	+,+	protein_cod	13087;13477
1896	chr1	187466731	-	chr1	187466474	+	N	N	N	N	N	Y	invers_r	na	NA	intergenic;in	RP11-445P19.3,RP11-	+,-	lincRNA,pse	16985;9239	intergenic;in	RP11-445P19.3,RP11-44	+,-	lincRNA,pse	16728;9496
1897	chr1	207293196	-	chr1	207292347	+	Y	Y	Y	Y	Y	Y	invers_r	na	NA	intronic	C4BPA	+	protein_cod	0	intronic	C4BPA	+	protein_cod	0
1898	chr1	234912188	-	chr1	17051740	+	N	N	Y	N	N	Y	invers_r	na	NA	intergenic	LINC01132,RNY4P16	+,+	lincRNA,mis	44798;61532	intergenic	ESPNP,FAM231C	-,-	pseudogene	5088;8707
1899	chr1	237566206	-	chr1	237566102	+	Y	Y	Y	Y	Y	Y	invers_r	na	NA	intronic	RYR2	+	protein_cod	0	intronic	RYR2	+	protein_cod	0
1900	chr10	13105272	-	chr10	13105037	+	Y	N	N	Y	Y	Y	invers_r	na	NA	intronic	CCDC3	-	protein_cod	0	intronic	CCDC3	-	protein_cod	0
1901	chr10	13105278	-	chr10	13104977	+	N	Y	N	N	N	N	invers_r	na	NA	intronic	CCDC3	-	protein_cod	0	intronic	CCDC3	-	protein_cod	0
1902	chr10	36301836	-	chr10	36148115	+	Y	N	N	N	N	Y	invers_r	na	NA	intergenic	RP11-309N24.1,RP11-	-,-	lincRNA,linc	113945;76178	intergenic	RPL7P37,RP11-309N24.	+,-	pseudogene	161150;37687
1903	chr10	36302105	-	chr10	36148562	+	N	Y	N	N	Y	N	invers_r	na	NA	intergenic	RP11-309N24.1,RP11-	-,-	lincRNA,linc	114214;75909	intergenic	RPL7P37,RP11-309N24.	+,-	pseudogene	161597;37240
1904	chr10	36302123	-	chr10	36148469	+	N	N	N	Y	N	N	invers_r	na	NA	intergenic	RP11-309N24.1,RP11-	-,-	lincRNA,linc	114232;75891	intergenic	RPL7P37,RP11-309N24.	+,-	pseudogene	161504;37333
1905	chr10	36302136	-	chr10	36148533	+	N	N	Y	N	N	N	invers_r	na	NA	intergenic	RP11-309N24.1,RP11-	-,-	lincRNA,linc	114245;75878	intergenic	RPL7P37,RP11-309N24.	+,-	pseudogene	161568;37269
1906	chr10	39143264	-	chr10	39086506	+	N	Y	N	N	N	N	invers_r	na	NA	intergenic	RP11-96F8.1,NONE	+,NONE	pseudogene	67025;NONE	intergenic	RP11-96F8.1,NONE	+,NONE	pseudogene	10267;NONE
1907	chr10	47059138	-	chr10	47023628	+	N	N	N	N	Y	N	invers_r	na	NA	ncRNA_intro	LINC00842	-	lincRNA	0	ncRNA_intro	LINC00842	-	lincRNA	0
1908	chr10	54712086	-	chr10	38228943	+	N	Y	Y	Y	Y	N	invers_r	na	NA	intergenic;in	Y_RNA,RP11-319F12.	+,-	misc_RNA,l	99623;21162	intergenic;in	ZNF33AP1,ZNF25	+,-	pseudogene	43349;9557
1909	chr10	108031865	-	chr10	84978863	+	Y	N	N	N	N	N	invers_r	na	NA	intergenic	RP11-298H24.1,RP11-	+,+	lincRNA,pse	83385;277999	intergenic	RNU6-478P,MARK2P15	-,-	snRNA,pseu	151036;92521
1910	chr11	1707842	-	chr11	1685834	+	N	N	N	Y	N	Y	invers_r	na	NA	upstream	FAM99B	-	antisense	0	upstream	FAM99A	+	processed_	0
1911	chr11	1707928	-	chr11	1686054	+	N	Y	N	N	N	N	invers_r	na	NA	intergenic	FAM99B,AP006285.6	-,+	antisense,lir	1069;1599	upstream	FAM99A	+	processed_	0
1912	chr11	1961055	-	chr11	1915253	+	N	N	N	N	N	Y	invers_r	na	NA	intergenic	TNNT3,MRPL23	+,+	protein_cod	1119;7453	intergenic	LSP1,LINC01150	+,+	protein_cod	1760;8624
1913	chr11	1961091	-	chr11	1915615	+	N	N	N	N	Y	N	invers_r	na	NA	intergenic	TNNT3,MRPL23	+,+	protein_cod	1155;7417	intergenic	LSP1,LINC01150	+,+	protein_cod	2122;8262
1914	chr11	24287579	-	chr11	24285122	+	N	N	N	N	N	Y	invers_r	na	NA	intergenic;in	RP11-2F20.1,Y_RNA	-,+	lincRNA,mis	3815;189878	intergenic;in	RP11-2F20.1,Y_RNA	-,+	lincRNA,mis	1358;192335
1915	chr11	24287914	-	chr11	24284731	+	N	N	N	Y	N	N	invers_r	na	NA	intergenic	RP11-2F20.1,Y_RNA	-,+	lincRNA,mis	4150;189543	upstream	RP11-2F20.1	-	lincRNA	0
1916	chr11	31803611	-	chr11	18267977	+	Y	N	N	N	N	N	invers_r	na	NA	intronic	ELP4	+	protein_cod	0	ncRNA_intro	SAA2-SAA4	-	processed_	0
1917	chr11	68924896	-	chr11	1722794	+	N	Y	Y	Y	Y	N	invers_r	na	NA	ncRNA_intro	RP11-554A11.9,RP11-	+,-	antisense,lir	0	intergenic	KRTAP5-6,IFITM10	+,-	protein_cod	3809;30846
1918	chr11	89079635	-	chr11	49381860	+	N	N	N	Y	N	N	invers_r	na	NA	intronic	NOX4	-	protein_cod	0	ncRNA_intro	CTD-2026G22.1	+	pseudogene	0
1919	chr11	89079636	-	chr11	49381318	+	Y	N	N	N	N	N	invers_r	na	NA	intronic	NOX4	-	protein_cod	0	ncRNA_intro	CTD-2026G22.1	+	pseudogene	0
1920	chr11	91808777	-	chr11	90421928	+	N	N	N	N	N	Y	invers_r	na	NA	intergenic	TUBB4BP4,RPL7AP57	-,+	pseudogene	125577;85495	ncRNA_intro	DISC1FP1	+	processed_	0
1921	chr11	106143790	-	chr11	106143086	+	Y	Y	Y	Y	Y	Y	invers_r	na	NA	intergenic	RP11-680E19.1,RP11-	+,+	pseudogene	8158;36982	intergenic	RP11-680E19.1,RP11-68	+,+	pseudogene	7454;37686
1922	chr11	122708411	-	chr11	70649927	+	N	N	N	N	N	Y	invers_r	na	NA	upstream	CRTAM	+	protein_cod	0	intronic	SHANK2	-	protein_cod	0
1923	chr12	9721880	-	chr12	9555638	+	N	N	Y	N	N	N	invers_r	na	NA	ncRNA_intro	RP11-726G1.1	+	pseudogene	0	ncRNA_intro	RP11-599J14.2	-	pseudogene	0
1924	chr12	9721884	-	chr12	9555397	+	N	N	N	N	N	Y	invers_r	na	NA	ncRNA_intro	RP11-726G1.1	+	pseudogene	0	ncRNA_intro	RP11-599J14.2	-	pseudogene	0
1925	chr12	9721891	-	chr12	9555627	+	N	N	N	N	Y	N	invers_r	na	NA	ncRNA_intro	RP11-726G1.1	+	pseudogene	0	ncRNA_intro	RP11-599J14.2	-	pseudogene	0
1926	chr12	25260554	-	chr12	25259494	+	Y	Y	Y	Y	Y	Y	invers_r	na	NA	intronic	LRMP	+	protein_cod	0	intronic	LRMP	+	protein_cod	0

1927	chr12	50183077	-	chr12	50182563	+	N	Y	N	N	N	N	invers_r	na	NA	intergenic	LSM6P2,NCKAP5L	+,-	pseudogene	18158;1852	intergenic	LSM6P2,NCKAP5L	+,-	pseudogene	17644;2366
1928	chr12	66554626	-	chr12	58058997	+	N	N	N	Y	N	N	invers_r	na	NA	intronic	TMBIM4,TMBIM4	-,-	protein_cod	0	intergenic	B4GALNT1,snoU13	-,-	protein_cod	31859;3730
1929	chr12	125801148	-	chr12	25956127	+	N	N	Y	Y	Y	Y	invers_r	na	NA	intergenic;ir	NONE,TMEM132B	NONE,+	NONE,prote	NONE;10014	ncRNA_intr	RP11-443N24.2	-	lincRNA	0
1930	chr12	125801148	-	chr12	104359630	+	Y	N	N	N	N	N	invers_r	na	NA	intergenic;ir	NONE,TMEM132B	NONE,+	NONE,prote	NONE;10014	UTR5	TDG	+	protein_cod	0
1931	chr13	25564594	-	chr13	21789417	+	N	N	N	Y	N	N	invers_r	na	NA	intergenic	RPL34P27,LSP1	-,+	pseudogene	1531;26947	intergenic	MRP63,RP11-101P17.14	+,+	protein_cod	36194;1277
1932	chr13	47039539	-	chr13	45952099	+	N	N	Y	N	N	N	invers_r	na	NA	ncRNA_intr	RP11-189B4.6	-	lincRNA	0	ncRNA_intr	TPT1-AS1	+	antisense	0
1933	chr13	86917308	-	chr13	86914888	+	Y	Y	N	Y	Y	Y	invers_r	na	NA	intergenic	RP11-30L8.1,DDX6P2	+,+	lincRNA,pse	172283;10447	intergenic	RP11-30L8.1,DDX6P2	+,+	lincRNA,pse	169863;12867
1934	chr13	87840282	-	chr13	87015014	+	N	N	N	N	N	Y	invers_r	na	NA	intergenic	LINC00430,UBBP5	+,+	lincRNA,pse	251011;30977	intergenic	DDX6P2,TXNL1P1	+,+	pseudogene	85490;362741
1935	chr13	89112881	-	chr13	89110970	+	Y	N	N	N	N	Y	invers_r	na	NA	intergenic;ir	RPL29P29,LINC00433	-,+	pseudogene	223657;80238	intergenic;ir	RPL29P29,LINC00433	-,+	pseudogene	221746;82149
1936	chr14	58412160	-	chr14	58259279	+	Y	Y	Y	Y	Y	Y	invers_r	na	NA	intergenic	SLC35F4,RP11-999E2	-,-	protein_cod	79380;48103	intronic	SLC35F4	-	protein_cod	0
1937	chr14	60941377	-	chr14	60913734	+	N	N	N	N	N	Y	invers_r	na	NA	intronic	C14orf39	-	protein_cod	0	intronic	C14orf39	-	protein_cod	0
1938	chr14	63226284	-	chr14	47117868	+	Y	N	Y	Y	N	N	invers_r	na	NA	intronic	KCNH5	-	protein_cod	0	intergenic	LINC00871,RPL10L	+,-	lincRNA,pro	146842;2354
1939	chr14	93926722	-	chr14	93926105	+	N	N	Y	N	N	N	invers_r	na	NA	intronic	UNC79	+	protein_cod	0	intronic	UNC79	+	protein_cod	0
1940	chr14	97971695	-	chr14	24098400	+	N	N	Y	Y	N	Y	invers_r	na	NA	ncRNA_intr	CTD-2506J14.1	+	lincRNA	0	upstream	DHRS2	+	protein_cod	0
1941	chr15	31956457	-	chr15	31033637	+	N	Y	Y	N	Y	N	invers_r	na	NA	intronic	OTUD7A	-	protein_cod	0	intergenic	ARHGAP11B,GOLGA8U	+,+	protein_cod	55827;50139
1942	chr15	97899864	-	chr15	97899741	+	N	N	N	Y	N	N	invers_r	na	NA	ncRNA_intr	CTD-2147F2.1	+	lincRNA	0	ncRNA_intr	CTD-2147F2.1	+	lincRNA	0
1943	chr16	48651331	-	chr16	48650866	+	N	Y	Y	N	N	N	invers_r	na	NA	intergenic	N4BP1,CTC-527H23.2	-,+	protein_cod	7211;2899	intergenic	N4BP1,CTC-527H23.2	-,+	protein_cod	6746;3364
1944	chr16	80277320	-	chr16	24120359	+	N	N	N	N	Y	N	invers_r	na	NA	ncRNA_intr	RP11-525K10.3	-	antisense	0	intronic	PRKCB	+	protein_cod	0
1945	chr16	85189800	-	chr16	85188713	+	Y	Y	Y	N	Y	Y	invers_r	na	NA	intergenic	FAM92B,CTC-786C10	-,+	protein_cod	43686;15082	intergenic	FAM92B,CTC-786C10.1	-,+	protein_cod	42599;16169
1946	chr17	21828533	-	chr17	21303090	+	Y	N	N	N	N	N	invers_r	na	NA	upstream	RP11-1109M24.13	-	pseudogene	0	intronic	KCNJ12	+	protein_cod	0
1947	chr17	41401056	-	chr17	41379303	+	Y	Y	Y	Y	Y	Y	invers_r	na	NA	intergenic	LINC00854,LINC0091(	-,-	processed_	17718;46157	ncRNA_intr	LINC00854	-	processed_	0
1948	chr17	41463434	-	chr17	41381590	+	Y	Y	Y	Y	Y	Y	invers_r	na	NA	ncRNA_exc	LINC00910	-	lincRNA	0	ncRNA_intr	LINC00854	-	processed_	0
1949	chr17	74606806	-	chr17	73249716	+	N	N	N	N	N	Y	invers_r	na	NA	intergenic	RP11-666A8.11,ST6G/	-,-	pseudogene	14952;14037	intronic	GGA3	-	protein_cod	0
1950	chr17	74607007	-	chr17	73249567	+	N	Y	N	N	N	N	invers_r	na	NA	intergenic	RP11-666A8.11,ST6G/	-,-	pseudogene	15153;13836	intronic	GGA3	-	protein_cod	0
1951	chr18	14172168	-	chr18	12089182	+	N	Y	N	N	N	N	invers_r	na	NA	intergenic	NF1P5,ANKRD20A5P	-,+	pseudogene	14827;6928	intergenic	RP11-815J4.1,RP11-815	+,-	lincRNA,pse	3916;2491
1952	chr18	22922754	-	chr18	22446131	+	Y	N	N	N	N	N	invers_r	na	NA	intronic	ZNF521	-	protein_cod	0	ncRNA_intr	RP11-449D8.5	+	lincRNA	0
1953	chr19	16014991	-	chr19	15822763	+	N	N	Y	N	N	N	invers_r	na	NA	upstream	AC005336.5	-	pseudogene	0	intergenic	CYP4F12,OR10H2	+,+	protein_cod	14779;16071
1954	chr19	34739822	-	chr19	34737017	+	Y	N	N	N	N	N	invers_r	na	NA	intergenic	LSM14A,KIAA0355	+,+	protein_cod	19402;5620	intergenic	LSM14A,KIAA0355	+,+	protein_cod	16597;8425
1955	chr19	44963092	-	chr19	44914099	+	N	N	Y	N	N	N	invers_r	na	NA	intergenic	ZNF229,ZNF285B	-,+	protein_cod	10326;8766	ncRNA_intr	CTC-512J12.4	-	processed_	0
1956	chr19	44963108	-	chr19	44914140	+	N	N	N	N	Y	N	invers_r	na	NA	intergenic	ZNF229,ZNF285B	-,+	protein_cod	10342;8750	ncRNA_intr	CTC-512J12.4	-	processed_	0
1957	chr2	16407765	-	chr2	16406393	+	Y	Y	Y	Y	Y	Y	invers_r	na	NA	ncRNA_intr	AC010745.2	+	lincRNA	0	ncRNA_intr	AC010745.2	+	lincRNA	0
1958	chr2	61703127	-	chr2	61700745	+	N	N	N	N	N	Y	invers_r	na	NA	ncRNA_intr	RP11-355B11.2	+	antisense	0	ncRNA_intr	RP11-355B11.2	+	antisense	0
1959	chr2	116981881	-	chr2	116974339	+	Y	Y	N	Y	Y	Y	invers_r	na	NA	intergenic	DPP10,RP11-338I24.1	+,-	protein_cod	378553;462913	intergenic	DPP10,RP11-338I24.1	+,-	protein_cod	371011;470455
1960	chr2	131887201	-	chr2	131886791	+	N	N	Y	N	N	N	invers_r	na	NA	intronic	PLEKHB2	+	protein_cod	0	intronic	PLEKHB2	+	protein_cod	0
1961	chr2	131887269	-	chr2	131886619	+	N	N	N	Y	N	N	invers_r	na	NA	intronic	PLEKHB2	+	protein_cod	0	intronic	PLEKHB2	+	protein_cod	0
1962	chr2	131887307	-	chr2	131886577	+	Y	N	N	N	Y	Y	invers_r	na	NA	intronic	PLEKHB2	+	protein_cod	0	intronic	PLEKHB2	+	protein_cod	0
1963	chr2	153461446	-	chr2	153461095	+	N	N	N	N	N	Y	invers_r	na	NA	intronic	FMNL2	+	protein_cod	0	intronic	FMNL2	+	protein_cod	0
1964	chr2	216474151	-	chr2	216473154	+	Y	N	N	N	N	N	invers_r	na	NA	ncRNA_intr	AC012668.2	+	lincRNA	0	ncRNA_intr	AC012668.2	+	lincRNA	0
1965	chr2	228075904	-	chr2	228075156	+	N	N	N	N	Y	N	invers_r	na	NA	intronic;intrc	COL4A3	+	protein_cod	0	intronic	COL4A3	+	protein_cod	0
1966	chr2	228075904	-	chr2	228075219	+	N	Y	N	N	N	N	invers_r	na	NA	intronic;intrc	COL4A3	+	protein_cod	0	intronic	COL4A3	+	protein_cod	0
1967	chr20	18595822	-	chr20	18591558	+	N	N	N	Y	N	N	invers_r	na	NA	intronic;intrc	DTD1	+	protein_cod	0	intronic;intrc	DTD1	+	protein_cod	0
1968	chr21	41405547	-	chr21	41400241	+	N	N	N	N	N	Y	invers_r	na	NA	intronic	DSCAM	-	protein_cod	0	intronic	DSCAM	-	protein_cod	0
1969	chr21	41406898	-	chr21	41398962	+	N	N	Y	N	N	N	invers_r	na	NA	intronic	DSCAM	-	protein_cod	0	intronic	DSCAM	-	protein_cod	0
1970	chr21	41407177	-	chr21	41398726	+	N	Y	N	N	N	N	invers_r	na	NA	intronic	DSCAM	-	protein_cod	0	intronic	DSCAM	-	protein_cod	0
1971	chr22	29065782	-	chr22	29065291	+	Y	N	N	Y	N	N	invers_r	na	NA	intronic	TTC28	-	protein_cod	0	intronic	TTC28	-	protein_cod	0
1972	chr22	38506347	-	chr22	37724999	+	N	N	N	N	N	Y	invers_r	na	NA	intronic	BAIAP2L2	-	protein_cod	0	intergenic	CYTH4,RP1-63G5.5	+,-	protein_cod	13617;10625
1973	chr3	22782477	-	chr3	22751299	+	Y	N	N	N	N	N	invers_r	na	NA	intergenic	AC092421.1,RP11-103	-,-	pseudogene	70275;70404	intergenic	AC092421.1,RP11-1037I	-,-	pseudogene	39097;101582
1974	chr3	82204007	-	chr3	82203550	+	N	N	N	N	Y	N	invers_r	na	NA	ncRNA_intr	RP11-260O18.1	+	lincRNA	0	ncRNA_intr	RP11-260O18.1	+	lincRNA	0
1975	chr3	131713384	-	chr3	131708358	+	N	Y	N	N	N	N	invers_r	na	NA	intronic	CPNE4	-	protein_cod	0	intronic	CPNE4	-	protein_cod	0
1976	chr4	3627529	-	chr4	3598722	+	Y	Y	Y	Y	Y	Y	invers_r	na	NA	intergenic	LINC00955,RP3-368B!	+,+	protein_cod	35798;7227	intergenic	LINC00955,RP3-368B9.2	+,+	protein_cod	6991;36034
1977	chr4	4004836	-	chr4	3932025	+	N	N	N	Y	N	N	invers_r	na	NA	intergenic	ENPP7P9,AC116562.2	-,+	pseudogene	35449;33975	ncRNA_intr	AC226119.5	-	processed_	0
1978	chr4	4005095	-	chr4	3931580	+	Y	N	N	N	N	N	invers_r	na	NA	intergenic	ENPP7P9,AC116562.2	-,+	pseudogene	35708;33716	ncRNA_intr	AC226119.5	-	processed_	0
1979	chr4	9641431	-	chr4	4007090	+	Y	N	N	N	N	N	invers_r	na	NA	intergenic	AC097493.1,ENPP7P1	-,+	miRNA,pseu	39380;37501	intergenic	ENPP7P9,AC116562.2	-,+	pseudogene	37703;31721
1980	chr4	79035960	-	chr4	78178993	+	N	N	N	N	Y	N	invers_r	na	NA	intronic	FRAS1	+	protein_cod	0	intergenic	CTB-179I1.3,CTB-179I1.	-,-	pseudogene	40962;53557
1981	chr4	88858700	-	chr4	88847164	+	Y	Y	Y	Y	Y	Y	invers_r	na	NA	intergenic	HSP90AB3P,SPP1	+,+	pseudogene	43533;38119	intergenic	HSP90AB3P,SPP1	+,+	pseudogene	31997;49655
1982	chr4	91936102	-	chr4	91933591	+	N	N	Y	N	N	Y	invers_r	na	NA	intronic	CCSER1	+	protein_cod	0	intronic	CCSER1	+	protein_cod	0
1983	chr5	8998701	-	chr5	7262631	+	N	N	N	Y	N	N	invers_r	na	NA	intergenic	RP11-143A12.3,CTD-2	+,+	lincRNA,ant	117064;3185	intergenic	RP11-122F24.1,RP11-40	+,-	lincRNA,pse	71706;25406
1984	chr5	178183075	-	chr5	170260554	+	N	N	N	N	N	Y	invers_r	na	NA	intergenic	ZNF354A,AACSP1	-,-	protein_cod	25372;8787	intergenic	GABRP,RANBP17	+,+	protein_cod	19503;28320
1985	chr5	178183082	-	chr5	170260575	+	Y	Y	Y	N	N	N	invers_r	na	NA	intergenic	ZNF354A,AACSP1	-,-	protein_cod	25379;8780	intergenic	GABRP,RANBP17	+,+	protein_cod	19524;28299
1986	chr5	180100887	-	chr5	178266107	+	N	N	N	N	N	Y	invers_r	na	NA	intergenic	RP11-451H23.2,RP11-	-,+	pseudogene	1289;10879	upstream	RP11-21I4.2	+	pseudogene	0
1987	chr6	26762449	-	chr6	26739397	+	N	N	N	N	N	Y	invers_r	na	NA	intergenic	RP11-457M11.5,GUSE	+,-	lincRNA,pse	74257;76814	intergenic	RP11-457M11.5,GUSBP2	+,-	lincRNA,pse	51205;99866
1988	chr6	26762529	-	chr6	26739173	+	Y	N	Y	N	N	N	invers_r	na	NA	intergenic	RP11-457M11.5,GUSE	+,-	lincRNA,pse	74337;76734	intergenic	RP11-457M11.5,GUSBP2	+,-	lincRNA,pse	50981;100090
1989	chr6	26762768	-	chr6	26739097	+	N	N	N	Y	Y	N	invers_r	na	NA	intergenic	RP11-457M11.5,GUSE	+,-	lincRNA,pse	74576;76495	intergenic	RP11-457M11.5,GUSBP2	+,-	lincRNA,pse	50905;100166
1990	chr6	57585275	-	chr6	57572693	+	N	N	Y	N	N	N	invers_r	na	NA	intergenic	PRIM2,GAPDHP41	+,-	protein_cod	71900;101694	intergenic	PRIM2,GAPDHP41	+,-	protein_cod	59318;114276
1991	chr6	62197720	-	chr6	62178582	+	N	N	N	Y	N	N	invers_r	na	NA	intergenic	AL356131.1,MTRNR2L	+,+	miRNA,prot	140838;86288	intergenic	AL356131.1,MTRNR2L9	+,+	miRNA,prot	121700;105426
1992	chr6	131884784	-	chr6	131884381	+	N	Y	N	N	N	N	invers_r	na	NA	intergenic	RPL21P67,ARG1	+,+	pseudogene	94108;9500	intergenic	RPL21P67,ARG1	+,+	pseudogene	93705;9903
1993	chr6	131884831	-	chr6	131884674	+	N	N	Y	N	N	N	invers_r	na	NA	intergenic	RPL21P67,ARG1	+,+	pseudogene	94155;9453	intergenic	RPL21P67,ARG1	+,+	pseudogene	93998;9610
1994	chr6	131884983	-	chr6	131884172	+	Y	N	N	N	N	N	invers_r	na	NA	intergenic	RPL21P67,ARG1	+,+	pseudogene	94307;9301	intergenic	RPL21P67,ARG1	+,+	pseudogene	93496;10112
1995	chr6	131884988	-	chr6	131884225	+	N	N	N	Y	N	N	invers_r	na	NA	intergenic	RPL21P67,ARG1	+,+	pseudogene	94312;9296	intergenic	RPL21P67,ARG1	+,+	pseudogene	93549;10059
1996	chr6	170429210	-	chr6	170429097	+	N	N	N	N	N	Y	invers_r	na	NA	intergenic	RP1-182D15.2,RP11-3	-,+	lincRNA,ant	219012;48531	intergenic	RP1-182D15.2,RP11-302	-,+	lincRNA,ant	218899;48644
1997	chr7	63792201	-	chr7	63442369	+	Y	N	Y	N	Y	N	invers_r	na	NA	intronic	ZNF736	+	protein_cod	0	upstream	RP5-1193P9.4	-	pseudogene	0
1998	chr7	63792265	-	chr7	63442391	+	N	Y	N	Y	N	Y	invers_r	na	NA	intronic	ZNF736	+	protein_cod	0	upstream	RP5-1193P9.4	-	pseudogene	0
1999	chr7	70438880	-	chr7	70426184	+	Y	Y	Y	Y	Y	N	invers_r	na	NA	intergenic	RP11-575M4.1,WBSCI	+,+	pseudogene	135881;158275	intergenic	RP11-575M4.1,WBSCR1	+,+	pseudogene	123185;170971
2000	chr7	72019692	-	chr7	66725133	+	N	N	Y	N	N	N	invers_r	na	NA	intergenic	CALN1,TYW1B	-,-	protein_cod	107556;19808	intergenic	TYW1,RP11-166O4.1	+,+	protein_cod	20632;19632
2001	chr7	72019706	-	chr7	66725202	+	N	N	N	Y	N	N	invers_r	na	NA	intergenic	CALN1,TYW1B	-,-	protein_cod	107570;19794	intergenic	TYW1,RP11-166O4.1	+,+	protein_cod	20701;19563
2002	chr7	98082528	-	chr7	75108686	+	N	N	Y	N	N	N	invers_r	na	NA	downstream	AC091654.7	-	pseudogene	0	ncRNA_intr	AC006014.7	-	pseudogene	0
2003	chr7	98408740	-	chr7	6675056	+	N	N	N	Y	N	N	invers_r	na	NA	intergenic	RNU6-393P,TMEM130	-,-	snRNA,prot	16293;35371	intergenic	ZNF853,ZNF316	+,+	protein_cod	11135;1897
2004	chr7	98409191	-	chr7	6675112	+	N	Y	N	N	N	N	invers_r	na	NA	intergenic	RNU6-393P,TMEM130	-,-	snRNA,prot	16744;34920	intergenic	ZNF853,ZNF316	+,+	protein_cod	11191;1841
2005	chr7	142033836	-	chr7	142032999	+	N	Y	Y	Y	N	N	invers_r	na	NA	intergenic	TRBV7-1,TRBV4-2	+,+	TR_V_gene	1309;11416	downstream	TRBV7-1	+	TR_V_gene	0
2006	chr7	142033883	-	chr7	142032999	+	N	N	N	N	Y	Y	invers_r	na	NA	intergenic	TRBV7-1,TRBV4-2	+,+	TR_V_gene	1356;11369	downstream	TRBV7-1	+	TR_V_gene	0
2007	chr7	142100157	-	chr7	142031237	+	Y	N	N	N	N	N	invers_r	na	NA	upstream	TRBV7-8	-	TR_V_gene	0	upstream	TRBV7-1	+	TR_V_gene	0
2008	chr7	142183747	-	chr7	142045948	+	N	N	N	N	Y	N	invers_r	na	NA	intergenic;ir	TRBV6-5,TRBV12-2	-,-	TR_V_gene	2731;6891	downstream	TRBV4-2	+	TR_V_gene	0
2009	chr7	147571555	-	chr7	124662693	+	N	Y	N	N	N	N	invers_r	na	NA	intronic	CNTNAP2	+	protein_cod	0	ncRNA_intr	POT1-AS1	+	antisense	0
2010	chr7	147571592	-	chr7	124662610	+	Y	N	N	Y	N	N	invers_r	na	NA	intronic;intrc	CNTNAP2	+	protein_cod	0	ncRNA_intr	POT1-AS1	+	antisense	0

2011	chr7	154943264	-	chr7	151768311	+	Y	Y	Y	Y	Y	N	invers_r	na	NA	intergenic	AC092628.3,RN7SKP2	+,+	pseudogene	44903;30327	intronic	GALNT11	+	protein_cod	0
2012	chr7	154943307	-	chr7	151768268	+	N	N	N	N	N	Y	invers_r	na	NA	intergenic	AC092628.3,RN7SKP2	+,+	pseudogene	44946;30284	intronic	GALNT11	+	protein_cod	0
2013	chr7	158956388	-	chr7	158925728	+	N	N	N	Y	Y	N	invers_r	na	NA	intergenic	VIPR2,PIP5K1P2	-,+	protein_cod	18739;67737	intronic	VIPR2	-	protein_cod	0
2014	chr8	36161662	-	chr8	36160827	+	Y	N	N	N	N	N	invers_r	na	NA	intergenic	RP11-593P24.3,RP11-	-,+	pseudogene	24448;4101	intergenic	RP11-593P24.3,RP11-13	-,+	pseudogene	23613;4936
2015	chr8	71768003	-	chr8	68667664	+	Y	N	N	N	N	N	invers_r	na	NA	intergenic	Y_RNA,RP11-326E22.	-,+	misc_RNA,a	74760;299689	intergenic	CPA6,NDUFS5P6	-,+	protein_cod	9044;93615
2016	chr8	144926458	-	chr8	143791732	+	N	N	N	N	Y	N	invers_r	na	NA	downstream	AC105049.1	+	miRNA	0	ncRNA_intr	CTD-2292P10.4	-	antisense	0
2017	chr8	144926483	-	chr8	143791675	+	Y	N	N	N	N	N	invers_r	na	NA	downstream	AC105049.1	+	miRNA	0	ncRNA_intr	CTD-2292P10.4	-	antisense	0
2018	chr9	66478619	-	chr9	66469907	+	Y	N	N	N	N	N	invers_r	na	NA	intergenic	AL512625.1,RP11-262	+,-	pseudogene	4946;14953	downstream	RP11-262H14.1	+	lincRNA	0
2019	chr9	68414094	-	chr9	66455691	+	Y	Y	Y	Y	Y	Y	invers_r	na	NA	ncRNA_exc	LINC00537	-	pseudogene	0	intergenic	RP11-459O16.7,RP11-26	+,+	pseudogene	58578;1594
2020	chr9	95646217	-	chr9	90748590	+	N	Y	N	Y	N	Y	invers_r	na	NA	ncRNA_exc	ANKRD19P,RP11-526I	+,+	pseudogene	0	ncRNA_intr	SPATA31C2	-	pseudogene	0
2021	chr9	95646224	-	chr9	90748565	+	N	N	Y	N	Y	N	invers_r	na	NA	ncRNA_exc	ANKRD19P,RP11-526I	+,+	pseudogene	0	ncRNA_exc	SPATA31C2	-	pseudogene	0
2022	chrX	27032048	-	chrX	27031233	+	N	N	N	N	N	Y	invers_r	na	NA	intergenic	RNU1-142P,RP11-268	+,-	snRNA,lincR	223645;28976	intergenic	RNU1-142P,RP11-268G1	+,-	snRNA,lincR	222830;29791
2023	chrX	29270196	-	chrX	29269148	+	Y	Y	Y	Y	Y	Y	invers_r	na	NA	intronic	IL1RAPL1	+	protein_cod	0	intronic	IL1RAPL1	+	protein_cod	0
2024	chrX	51965509	-	chrX	51279977	+	N	N	N	N	Y	N	invers_r	na	NA	intergenic	RP11-363G10.2,XAGE	+,+	protein_cod	19151;146664	intergenic	RP11-56H2.2,CENPVP3	-,-	lincRNA,pse	26120;80984
2025	chrX	51965767	-	chrX	51279899	+	N	N	Y	N	N	N	invers_r	na	NA	intergenic	RP11-363G10.2,XAGE	+,+	protein_cod	19409;146406	intergenic	RP11-56H2.2,CENPVP3	-,-	lincRNA,pse	26042;81062
2026	chrX	72281484	-	chrX	72255695	+	N	N	Y	N	N	N	invers_r	na	NA	intergenic	PABPC1L2B,PABPC1L	+,-	protein_cod	55933;15631	intergenic	PABPC1L2B,PABPC1L2A	+,-	protein_cod	30144;41420
2027	chrX	115294239	-	chrX	115180688	+	N	N	Y	N	N	N	invers_r	na	NA	intergenic	API5P1,AGTR2	-,+	pseudogene	54556;7736	intergenic	AKR1B1P8,API5P1	+,-	pseudogene	5017;57486
2028	chr1	2585587	+	chr1	2584784	+	N	N	N	N	N	Y	tandem_dup	na	NA	intronic	TTC34	-	protein_cod	0	intronic	TTC34	-	protein_cod	0
2029	chr1	2615874	+	chr1	2583521	+	N	N	Y	N	N	N	tandem_dup	na	NA	intronic	TTC34	-	protein_cod	0	intronic	TTC34	-	protein_cod	0
2030	chr1	2621352	+	chr1	2615734	+	Y	N	N	N	N	N	tandem_dup	na	NA	intronic	TTC34	-	protein_cod	0	intronic	TTC34	-	protein_cod	0
2031	chr1	2622924	+	chr1	2620353	+	N	N	N	N	Y	N	tandem_dup	na	NA	intronic	TTC34	-	protein_cod	0	intronic	TTC34	-	protein_cod	0
2032	chr1	2629920	+	chr1	2615097	+	N	Y	N	N	N	N	tandem_dup	na	NA	intronic	TTC34	-	protein_cod	0	intronic	TTC34	-	protein_cod	0
2033	chr1	2630063	+	chr1	2584345	+	Y	N	N	N	N	N	tandem_dup	na	NA	intronic	TTC34	-	protein_cod	0	intronic	TTC34	-	protein_cod	0
2034	chr1	2630586	+	chr1	2615248	+	N	N	N	N	N	Y	tandem_dup	na	NA	intronic	TTC34	-	protein_cod	0	intronic	TTC34	-	protein_cod	0
2035	chr1	2631124	+	chr1	2615191	+	N	N	N	Y	N	N	tandem_dup	na	NA	intronic	TTC34	-	protein_cod	0	intronic	TTC34	-	protein_cod	0
2036	chr1	14438565	+	chr1	14436656	+	N	N	N	N	Y	Y	tandem_dup	na	NA	intergenic	PRDM2,RNU6-1265P	+,-	protein_cod	286991;12163	intergenic	PRDM2,RNU6-1265P	+,-	protein_cod	285082;14072
2037	chr1	14438567	+	chr1	14436307	+	N	N	Y	Y	N	N	tandem_dup	na	NA	intergenic	PRDM2,RNU6-1265P	+,-	protein_cod	286993;12161	intergenic	PRDM2,RNU6-1265P	+,-	protein_cod	284733;14421
2038	chr1	16386334	+	chr1	16376519	+	N	N	Y	N	N	N	tandem_dup	na	NA	intronic;intro	FAM131C	-	protein_cod	0	intronic;intro	CLCNKB	+	protein_cod	0
2039	chr1	16894068	+	chr1	16890792	+	Y	Y	Y	Y	N	Y	tandem_dup	na	NA	intronic	NBPF1	-	protein_cod	0	intronic	NBPF1	-	protein_cod	0
2040	chr1	24997299	+	chr1	24994642	+	N	Y	N	N	N	N	tandem_dup	na	NA	intronic;intro	SRRM1	+	protein_cod	0	intronic;intro	SRRM1	+	protein_cod	0
2041	chr1	25732109	+	chr1	25613677	+	N	N	Y	N	N	N	tandem_dup	na	NA	intronic	RHCE	-	protein_cod	0	intronic	C1orf63,RHD	-,+	protein_cod	0
2042	chr1	30878565	+	chr1	29720840	+	Y	Y	Y	Y	Y	Y	tandem_dup	na	NA	intergenic	RP3-357I16.1,RP4-59	+,-	pseudogene	179103;3842	intergenic	RP3-437I16.1,AL139151.	-,+	lincRNA,miR	44214;38153
2043	chr1	121138274	+	chr1	121137871	+	N	N	N	N	N	Y	tandem_dup	na	NA	ncRNA_intr	RP11-343N15.1	-	lincRNA	0	ncRNA_intr	RP11-343N15.1	-	lincRNA	0
2044	chr1	121138520	+	chr1	121137795	+	Y	N	Y	N	N	N	tandem_dup	na	NA	ncRNA_intr	RP11-343N15.1	-	lincRNA	0	ncRNA_intr	RP11-343N15.1	-	lincRNA	0
2045	chr1	121138779	+	chr1	121137800	+	N	Y	N	Y	N	N	tandem_dup	na	NA	ncRNA_exc	AL592494.5	+	lincRNA	0	ncRNA_intr	RP11-343N15.1	-	lincRNA	0
2046	chr1	121355584	+	chr1	121351586	+	N	N	N	N	N	Y	tandem_dup	na	VNTR	intergenic	RP11-344P13.1,NONE	-,NONE	lincRNA,NC	33262;NONE	intergenic	RP11-344P13.1,NONE	-,NONE	lincRNA,NC	29264;NONE
2047	chr1	142539510	+	chr1	142537156	+	N	N	N	N	N	Y	tandem_dup	na	NA	intergenic	NONE,RP11-417J8.1	NONE,+	NONE,lincR	NONE;13783	intergenic	NONE,RP11-417J8.1	NONE,+	NONE,lincR	NONE;16137
2048	chr1	144954614	+	chr1	144680038	+	N	N	N	Y	Y	N	tandem_dup	na	NA	intronic	PDE4DIP	-	protein_cod	0	upstream	WI2-1896O14.1	-	pseudogene	0
2049	chr1	148855745	+	chr1	148854275	+	N	N	Y	N	N	N	tandem_dup	na	NA	ncRNA_intr	RP11-763B22.9	+	pseudogene	0	ncRNA_intr	RP11-763B22.9	+	pseudogene	0
2050	chr1	162435796	+	chr1	150753785	+	N	N	N	N	Y	N	tandem_dup	na	NA	intergenic	SLAMF6P1,UHMK1	+,+	pseudogene	20120;31245	downstream	RP11-363I22.3	+	pseudogene	0
2051	chr1	189441263	+	chr1	188724848	+	N	N	N	Y	N	N	tandem_dup	na	NA	intergenic	RP11-373J16.2,RNA5S	-,-	pseudogene	338840;194016	intergenic	RP11-316I3.1,RP11-316I	-,-	lincRNA,linc	44837;113760
2052	chr1	202594536	+	chr1	202594186	+	N	N	N	Y	N	N	tandem_dup	na	NA	intronic	SYT2	-	protein_cod	0	intronic	SYT2	-	protein_cod	0
2053	chr1	208553076	+	chr1	103913905	+	Y	Y	N	Y	N	N	tandem_dup	na	NA	intergenic	RP11-2P2.1,RP11-565	-,-	pseudogene	124355;227369	intergenic	RP11-347K2.2,RP11-153	-,+	lincRNA,linc	77919;43596
2054	chr1	213087828	+	chr1	209627142	+	N	Y	N	N	N	N	tandem_dup	na	NA	intergenic	FLVCR1,VASH2	+,+	protein_cod	15123;36034	intergenic	MIR205HG,RP1-272L16.	+,-	lincRNA,linc	20959;74658
2055	chr1	225248414	+	chr1	225133381	+	N	N	N	N	Y	N	tandem_dup	na	NA	intronic	DNAH14	+	protein_cod	0	intronic	DNAH14	+	protein_cod	0
2056	chr1	225248440	+	chr1	225133514	+	Y	N	N	N	N	N	tandem_dup	na	NA	intronic	DNAH14	+	protein_cod	0	intronic	DNAH14	+	protein_cod	0
2057	chr1	225248447	+	chr1	225133422	+	N	N	Y	N	N	N	tandem_dup	na	NA	intronic	DNAH14	+	protein_cod	0	intronic	DNAH14	+	protein_cod	0
2058	chr1	225293694	+	chr1	225247315	+	N	N	N	N	N	Y	tandem_dup	na	NA	intronic	DNAH14	+	protein_cod	0	intronic	DNAH14	+	protein_cod	0
2059	chr1	225293736	+	chr1	225247384	+	N	N	N	N	Y	N	tandem_dup	na	NA	intronic	DNAH14	+	protein_cod	0	intronic	DNAH14	+	protein_cod	0
2060	chr1	239440575	+	chr1	239424007	+	N	Y	Y	N	Y	Y	tandem_dup	na	NA	upstream	RP11-544D21.1	-	lincRNA	0	intergenic	RP11-544D21.2,RP11-54	-,-	lincRNA,linc	9889;9018
2061	chr1	246410320	+	chr1	246407289	+	N	N	N	N	N	Y	tandem_dup	na	NA	intronic	SMYD3	-	protein_cod	0	intronic	SMYD3	-	protein_cod	0
2062	chr1	246410733	+	chr1	246407293	+	Y	Y	Y	Y	Y	N	tandem_dup	na	NA	intronic	SMYD3	-	protein_cod	0	intronic	SMYD3	-	protein_cod	0
2063	chr1	247383775	+	chr1	246526396	+	N	N	N	N	Y	N	tandem_dup	na	NA	ncRNA_intr	RP11-488L18.8	-	lincRNA	0	intronic	SMYD3	-	protein_cod	0
2064	chr1	247383999	+	chr1	246526383	+	Y	Y	N	N	N	N	tandem_dup	na	NA	ncRNA_intr	RP11-488L18.8	-	lincRNA	0	intronic	SMYD3	-	protein_cod	0
2065	chr10	3500943	+	chr10	3500521	+	N	N	N	N	N	Y	tandem_dup	na	NA	intergenic	RP11-195B3.1,RP11-4	+,-	lincRNA,linc	191736;28143	intergenic	RP11-195B3.1,RP11-482	+,-	lincRNA,linc	191314;28565
2066	chr10	3501152	+	chr10	3500535	+	N	N	Y	N	N	N	tandem_dup	na	NA	intergenic	RP11-195B3.1,RP11-4	+,-	lincRNA,linc	191945;27934	intergenic	RP11-195B3.1,RP11-482	+,-	lincRNA,linc	191328;28551
2067	chr10	7568745	+	chr10	7568619	+	Y	N	N	N	N	N	tandem_dup	na	NA	intergenic	RNU6-535P,RP11-385I	-,+	snRNA,antis	39974;6424	intergenic	RNU6-535P,RP11-385N2	-,+	snRNA,antis	39848;6550
2068	chr10	7568947	+	chr10	7568450	+	N	N	N	N	N	Y	tandem_dup	na	NA	intergenic	RNU6-535P,RP11-385I	-,+	snRNA,antis	40176;6222	intergenic	RNU6-535P,RP11-385N2	-,+	snRNA,antis	39679;6719
2069	chr10	7568978	+	chr10	7568396	+	N	N	Y	N	N	N	tandem_dup	na	NA	intergenic	RNU6-535P,RP11-385I	-,+	snRNA,antis	40207;6191	intergenic	RNU6-535P,RP11-385N2	-,+	snRNA,antis	39625;6773
2070	chr10	14610733	+	chr10	14610291	+	N	N	N	Y	N	N	tandem_dup	na	NA	intronic	FAM107B	-	protein_cod	0	intronic	FAM107B	-	protein_cod	0
2071	chr10	14610773	+	chr10	14610259	+	N	N	Y	N	Y	N	tandem_dup	na	NA	intronic	FAM107B	-	protein_cod	0	intronic	FAM107B	-	protein_cod	0
2072	chr10	39098719	+	chr10	39080756	+	N	N	N	Y	N	N	tandem_dup	na	NA	intergenic	RP11-96F8.1,NONE	+,NONE	pseudogene	22480;NONE	intergenic	RP11-96F8.1,NONE	+,NONE	pseudogene	4517;NONE
2073	chr10	42365055	+	chr10	42357633	+	Y	N	N	N	N	N	tandem_dup	na	NA	intergenic	NONE,KSR1P1	NONE,-	NONE,pseu	NONE;279703	intergenic	NONE,KSR1P1	NONE,-	NONE,pseu	NONE;287125
2074	chr10	42891748	+	chr10	38560670	+	Y	N	Y	Y	Y	Y	tandem_dup	na	NA	intergenic	RP11-313J2.1,CCNYL	-,-	pseudogene	28326;11868	ncRNA_intr	RP11-672F9.1	+	lincRNA	0
2075	chr10	54034307	+	chr10	53984566	+	Y	N	N	Y	N	N	tandem_dup	na	NA	ncRNA_intr	PRKG1-AS1	-	antisense	0	intronic	PRKG1	+	protein_cod	0
2076	chr10	54034310	+	chr10	53984595	+	N	Y	N	N	N	N	tandem_dup	na	NA	ncRNA_intr	PRKG1-AS1	-	antisense	0	intronic	PRKG1	+	protein_cod	0
2077	chr10	64994469	+	chr10	64993985	+	Y	Y	Y	Y	Y	Y	tandem_dup	na	NA	intronic	JMJD1C	-	protein_cod	0	intronic	JMJD1C	-	protein_cod	0
2078	chr10	76654961	+	chr10	19211100	+	N	Y	Y	Y	N	Y	tandem_dup	na	NA	intronic;intro	KAT6B	+	protein_cod	0	intergenic;ir	RP11-139J15.5,RP11-28	+,+	lincRNA,pse	174318;18330
2079	chr10	127201278	+	chr10	127190693	+	N	N	Y	N	N	Y	tandem_dup	na	NA	intergenic	RPS27P18,TEX36-AS	-,+	pseudogene	38777;61662	intergenic	RPS27P18,TEX36-AS1	-,+	pseudogene	28192;72247
2080	chr10	132589415	+	chr10	132588255	+	N	Y	N	N	N	Y	tandem_dup	na	NA	intergenic	Y_RNA,MIR378C	+,-	misc_RNA,r	285950;171436	intergenic	Y_RNA,MIR378C	+,-	misc_RNA,r	284790;172596
2081	chr10	132975004	+	chr10	132974640	+	N	Y	N	N	N	N	tandem_dup	na	NA	intronic	TCERG1L	-	protein_cod	0	intronic	TCERG1L	-	protein_cod	0
2082	chr10	133732171	+	chr10	133730087	+	N	N	Y	N	N	N	tandem_dup	na	NA	intergenic	AL450307.1,PPP2R2D	-,+	protein_cod	109636;15784	intergenic	AL450307.1,PPP2R2D	-,+	protein_cod	107552;17868
2083	chr11	475260	+	chr11	475156	+	Y	N	N	N	N	N	tandem_dup	na	NA	intronic	PTDSS2	+	protein_cod	0	intronic	PTDSS2	+	protein_cod	0
2084	chr11	1018254	+	chr11	1017074	+	Y	N	N	N	N	N	tandem_dup	na	NA	exonic	MUC6	-	protein_cod	0	exonic	MUC6	-	protein_cod	0
2085	chr11	1018288	+	chr11	1017615	+	N	Y	N	N	N	N	tandem_dup	na	NA	exonic	MUC6	-	protein_cod	0	exonic	MUC6	-	protein_cod	0
2086	chr11	1093282	+	chr11	1092837	+	N	Y	N	N	N	N	tandem_dup	na	NA	exonic	MUC2	+	protein_cod	0	exonic	MUC2	+	protein_cod	0
2087	chr11	1213574	+	chr11	1212850	+	Y	N	Y	Y	N	Y	tandem_dup	na	NA	intergenic	MUC5AC,MUC5B	+,+	protein_cod	51213;30722	intergenic	MUC5AC,MUC5B	+,+	protein_cod	50489;31446
2088	chr11	2193175	+	chr11	1735701	+	N	Y	Y	Y	Y	Y	tandem_dup	na	NA	upstream	TH	-	protein_cod	0	intergenic	KRTAP5-6,IFITM10	+,-	protein_cod	16716;17939
2089	chr11	24287790	+	chr11	24284963	+	N	Y	N	N	Y	Y	tandem_dup	na	NA	intergenic	RP11-2F20.1,Y_RNA	-,+	lincRNA,mis	4026;189667	intergenic	RP11-2F20.1,Y_RNA	-,+	lincRNA,mis	1199;192494
2090	chr11	24288237	+	chr11	24285250	+	N	N	Y	Y	N	N	tandem_dup	na	NA	intergenic	RP11-2F20.1,Y_RNA	-,+	lincRNA,mis	4473;189220	intergenic	RP11-2F20.1,Y_RNA	-,+	lincRNA,mis	1486;192207
2091	chr11	48609331	+	chr11	48545131	+	N	Y	N	N	N	N	tandem_dup	na	NA	intergenic	OR4A45P,OR4A41P	+,+	pseudogene	7443;1981	intergenic	OR4A40P,OR4A43P	+,+	pseudogene	10359;2485
2092	chr11	60867667	+	chr11	60866902	+	N	Y	N	N	N	N	tandem_dup	na	NA	intergenic	RP11-881M11.8,CD5	+,+	lincRNA,pro	32660;2200	intergenic	RP11-881M11.8,CD5	+,+	lincRNA,pro	31895;2965
2093	chr11	60884356	+	chr11	2209392	+	N	N	Y	N	N	N	tandem_dup	na	NA	intronic	CD5	+	protein_cod	0	intergenic	MIR4686,ASCL2	+,-	miRNA,prot	15024;80333
2094	chr11	63701653	+	chr11	63698909	+	Y	N	Y	N	N	N	tandem_dup	na	NA	intergenic	RCOR2,NAA40	-,+	protein_cod	17337;4815	intergenic	RCOR2,NAA40	-,+	protein_cod	14593;7559

2095	chr11	67592760	+	chr11	3463795	+	Y	N	N	N	N	N	tandem_dup	na	NA	intergenic	FAM86C2P,RP11-119D	-,+	pseudogene	19953;15653	intergenic	FAM86GP,RP13-726E6.1	-,+	pseudogene	20069;26754
2096	chr11	76904211	+	chr11	76903855	+	N	N	N	N	N	Y	tandem_dup	na	NA	intronic	MYO7A	+	protein_cod	0	intronic	MYO7A	+	protein_cod	0
2097	chr11	76904529	+	chr11	76904241	+	N	N	N	Y	N	N	tandem_dup	na	NA	intronic	MYO7A	+	protein_cod	0	intronic	MYO7A	+	protein_cod	0
2098	chr11	114482431	+	chr11	114479623	+	N	Y	N	N	N	N	tandem_dup	na	NA	intergenic	NXPE4,NXPE2	-,+	protein_cod	15947;66677	intergenic	NXPE4,NXPE2	-,+	protein_cod	13139;69485
2099	chr11	114482450	+	chr11	114479507	+	N	N	N	Y	N	N	tandem_dup	na	NA	intergenic	NXPE4,NXPE2	-,+	protein_cod	15966;66658	intergenic	NXPE4,NXPE2	-,+	protein_cod	13023;69601
2100	chr11	114482518	+	chr11	114479590	+	Y	N	N	N	N	N	tandem_dup	na	NA	intergenic	NXPE4,NXPE2	-,+	protein_cod	16034;66590	intergenic	NXPE4,NXPE2	-,+	protein_cod	13106;69518
2101	chr11	124121310	+	chr11	124110110	+	Y	Y	Y	Y	Y	Y	tandem_dup	na	NA	exonic	OR8G1	+	polymorphic	0	ncRNA_exo	OR8G7P	+	pseudogene	0
2102	chr11	131126487	+	chr11	130625478	+	N	Y	N	N	N	N	tandem_dup	na	NA	ncRNA_intr	AP002856.5	+	lincRNA	0	intergenic	C11orf44,PPP1R10P1	+,-	protein_cod	38231;33648
2103	chr11	131126716	+	chr11	130625776	+	N	N	N	N	Y	N	tandem_dup	na	NA	ncRNA_intr	AP002856.5	+	lincRNA	0	intergenic	C11orf44,PPP1R10P1	+,-	protein_cod	38529;33350
2104	chr12	8548959	+	chr12	8541751	+	Y	Y	Y	Y	N	N	tandem_dup	na	NA	ncRNA_intr	LINC00937	-	lincRNA	0	ncRNA_intr	LINC00937	-	lincRNA	0
2105	chr12	11200308	+	chr12	11173502	+	N	N	N	N	Y	N	tandem_dup	na	NA	downstream	TAS2R63P	-	pseudogene	0	downstream	TAS2R19	-	protein_cod	0
2106	chr12	11213856	+	chr12	11182894	+	N	N	N	Y	N	N	tandem_dup	na	NA	downstream	TAS2R46	-	protein_cod	0	downstream	TAS2R31	-	protein_cod	0
2107	chr12	11274230	+	chr12	11174098	+	N	N	N	N	N	Y	tandem_dup	na	NA	intergenic	RP11-673D15.7,TAS2F	-,-	pseudogene	24357;11327	downstream	TAS2R19	-	protein_cod	0
2108	chr12	11275888	+	chr12	11204715	+	Y	N	Y	N	Y	N	tandem_dup	na	NA	intergenic;ir	RP11-673D15.7,TAS2F	-,-	pseudogene	26015;9669	intergenic;ir	TAS2R63P,TAS2R46	-,-	pseudogene	2860;9249
2109	chr12	11284305	+	chr12	11181649	+	N	N	N	N	Y	N	tandem_dup	na	NA	intergenic;ir	RP11-673D15.7,TAS2F	-,-	pseudogene	34432;1252	intergenic	TAS2R19,TAS2R31	-,-	protein_cod	6430;1337
2110	chr12	21288183	+	chr12	21238731	+	Y	N	N	N	N	N	tandem_dup	na	NA	intronic	SLCO1B1,RP11-125O	+,+	protein_cod	0	intronic	SLCO1B3,SLCO1B7,LST	+,+,+,+	protein_cod	0
2111	chr12	21288217	+	chr12	21238871	+	N	Y	Y	N	N	Y	tandem_dup	na	NA	intronic	SLCO1B1,RP11-125O	+,+	protein_cod	0	intronic	SLCO1B3,SLCO1B7,LST	+,+,+,+	protein_cod	0
2112	chr12	21288433	+	chr12	21239099	+	N	N	N	Y	N	N	tandem_dup	na	NA	intronic	SLCO1B1,RP11-125O	+,+	protein_cod	0	intronic	SLCO1B3,SLCO1B7,LST	+,+,+,+	protein_cod	0
2113	chr12	31272203	+	chr12	9560034	+	Y	N	N	N	N	N	tandem_dup	na	NA	ncRNA_exo	RP11-551L14.1	-	pseudogene	0	ncRNA_intr	RP11-599J14.2	-	pseudogene	0
2114	chr12	31272487	+	chr12	9559077	+	N	N	N	N	N	Y	tandem_dup	na	NA	ncRNA_exo	RP11-551L14.1	-	pseudogene	0	ncRNA_intr	RP11-599J14.2	-	pseudogene	0
2115	chr12	31272542	+	chr12	9559824	+	N	Y	N	N	N	N	tandem_dup	na	NA	ncRNA_intr	RP11-551L14.1	-	pseudogene	0	ncRNA_intr	RP11-599J14.2	-	pseudogene	0
2116	chr12	31273640	+	chr12	31270878	+	N	N	N	N	N	Y	tandem_dup	na	NA	ncRNA_intr	RP11-551L14.1	-	pseudogene	0	ncRNA_intr	RP11-551L14.1	-	pseudogene	0
2117	chr12	31273656	+	chr12	9558553	+	N	N	Y	N	N	N	tandem_dup	na	NA	ncRNA_intr	RP11-551L14.1	-	pseudogene	0	ncRNA_intr	RP11-599J14.2	-	pseudogene	0
2118	chr12	31273989	+	chr12	31270871	+	N	N	N	Y	N	N	tandem_dup	na	NA	ncRNA_intr	RP11-551L14.1	-	pseudogene	0	ncRNA_intr	RP11-551L14.1	-	pseudogene	0
2119	chr12	31274023	+	chr12	9558742	+	N	Y	N	N	N	N	tandem_dup	na	NA	ncRNA_intr	RP11-551L14.1	-	pseudogene	0	ncRNA_intr	RP11-599J14.2	-	pseudogene	0
2120	chr12	31274028	+	chr12	31270911	+	Y	N	N	N	N	N	tandem_dup	na	NA	ncRNA_intr	RP11-551L14.1	-	pseudogene	0	ncRNA_intr	RP11-551L14.1	-	pseudogene	0
2121	chr12	31274046	+	chr12	9558548	+	N	N	N	Y	N	N	tandem_dup	na	NA	ncRNA_intr	RP11-551L14.1	-	pseudogene	0	ncRNA_intr	RP11-599J14.2	-	pseudogene	0
2122	chr12	31274074	+	chr12	9558702	+	Y	N	N	N	N	N	tandem_dup	na	NA	ncRNA_intr	RP11-551L14.1	-	pseudogene	0	ncRNA_intr	RP11-599J14.2	-	pseudogene	0
2123	chr12	38720293	+	chr12	34185572	+	N	Y	N	N	N	N	tandem_dup	na	NA	intergenic	ALG10B,AC087897.1	+,+	protein_cod	2509;146353	ncRNA_intr	RP11-847H18.2	-	antisense	0
2124	chr12	108757741	+	chr12	108757565	+	N	N	N	N	N	Y	tandem_dup	na	VNTR	intergenic	CMKLR1,RP11-13G14	-,-	protein_cod	24623;70166	intergenic	CMKLR1,RP11-13G14.4	-,-	protein_cod	24447;70342
2125	chr12	124923589	+	chr12	124923341	+	N	N	Y	N	N	N	tandem_dup	na	NA	intronic	NCOR2	-	protein_cod	0	intronic	NCOR2	-	protein_cod	0
2126	chr12	126085333	+	chr12	126062628	+	Y	Y	Y	Y	Y	Y	tandem_dup	na	NA	intronic	TMEM132B	+	protein_cod	0	intronic	TMEM132B	+	protein_cod	0
2127	chr13	27547704	+	chr13	27546837	+	Y	Y	Y	Y	Y	Y	tandem_dup	na	NA	intergenic	FGFR1OP2P1,RPS21	+,+	pseudogene	67706;3976	intergenic	FGFR1OP2P1,RPS21P8	+,+	pseudogene	66839;4843
2128	chr13	28509336	+	chr13	28509187	+	Y	N	N	N	N	N	tandem_dup	na	NA	intergenic	PDX1,ATP5EP2	+,+	protein_cod	8968;10007	intergenic	PDX1,ATP5EP2	+,+	protein_cod	8819;10156
2129	chr13	28509417	+	chr13	28508805	+	N	Y	N	Y	Y	Y	tandem_dup	na	NA	intergenic;ir	PDX1,ATP5EP2	+,+	protein_cod	9049;9926	intergenic	PDX1,ATP5EP2	+,+	protein_cod	8437;10538
2130	chr13	28509417	+	chr13	28509010	+	N	N	Y	N	N	N	tandem_dup	na	NA	intergenic;ir	PDX1,ATP5EP2	+,+	protein_cod	9049;9926	intergenic	PDX1,ATP5EP2	+,+	protein_cod	8642;10333
2131	chr13	29920761	+	chr13	29785545	+	N	N	N	N	Y	N	tandem_dup	na	NA	intronic	MTUS2	+	protein_cod	0	intronic	MTUS2	+	protein_cod	0
2132	chr13	36532289	+	chr13	36530759	+	N	Y	N	N	N	N	tandem_dup	na	NA	intronic	DCLK1	-	protein_cod	0	intronic	DCLK1	-	protein_cod	0
2133	chr13	42947655	+	chr13	42947268	+	Y	Y	Y	Y	Y	Y	tandem_dup	na	NA	intergenic	FABP3P2,TNFSF11	-,+	pseudogene	3862;189217	intergenic	FABP3P2,TNFSF11	-,+	pseudogene	3475;189604
2134	chr13	97159872	+	chr13	97159357	+	Y	N	N	N	N	N	tandem_dup	na	NA	intronic	HS6ST3	+	protein_cod	0	intronic	HS6ST3	+	protein_cod	0
2135	chr13	98505032	+	chr13	98504919	+	N	Y	Y	Y	N	N	tandem_dup	na	VNTR	intergenic	RP11-120E13.1,snoU1	-,+	lincRNA,snc	174815;33031	intergenic	RP11-120E13.1,snoU13	-,+	lincRNA,snc	174702;33144
2136	chr13	98505313	+	chr13	98504921	+	Y	N	N	N	N	Y	tandem_dup	na	VNTR	intergenic	RP11-120E13.1,snoU1	-,+	lincRNA,snc	175096;32750	intergenic	RP11-120E13.1,snoU13	-,+	lincRNA,snc	174704;33142
2137	chr14	33347576	+	chr14	33329684	+	Y	Y	Y	Y	Y	Y	tandem_dup	na	NA	intergenic	AKAP6,NPAS3	+,+	protein_cod	47009;56563	intergenic	AKAP6,NPAS3	+,+	protein_cod	29117;74455
2138	chr14	39600174	+	chr14	39282625	+	N	Y	Y	N	Y	Y	tandem_dup	na	NA	intronic	GEMIN2	+	protein_cod	0	ncRNA_intr	LINC00639	-	lincRNA	0
2139	chr14	48839640	+	chr14	20294415	+	Y	Y	Y	Y	Y	Y	tandem_dup	na	NA	intergenic	RNU6-297P,RP11-2G1	-,-	snRNA,linc	29105;26071	intronic	OR4N2	+	protein_cod	0
2140	chr14	50192356	+	chr14	50192041	+	N	Y	Y	N	Y	Y	tandem_dup	na	NA	intronic	KLHDC1	+	protein_cod	0	intronic	KLHDC1	+	protein_cod	0
2141	chr14	94846684	+	chr14	94830087	+	N	N	N	N	Y	N	tandem_dup	na	NA	intronic	SERPINA1	-	protein_cod	0	ncRNA_exo	SERPINA2P	-	pseudogene	0
2142	chr14	94846743	+	chr14	94830068	+	N	Y	Y	N	N	Y	tandem_dup	na	NA	intronic	SERPINA1	-	protein_cod	0	ncRNA_exo	SERPINA2P	-	pseudogene	0
2143	chr14	106227299	+	chr14	106082702	+	Y	N	N	N	N	N	tandem_dup	na	NA	intergenic	IGHG1,IGHG3	-,-	IG_C_gene,	17891;8140	intergenic	RP11-731F5.1,IGHG4	-,-	lincRNA,IG_	11008;7985
2144	chr14	106227330	+	chr14	106082739	+	N	N	Y	Y	N	N	tandem_dup	na	NA	intergenic	IGHG1,IGHG3	-,-	IG_C_gene,	17922;8109	intergenic	RP11-731F5.1,IGHG4	-,-	lincRNA,IG_	11045;7948
2145	chr14	106227334	+	chr14	106082667	+	N	Y	N	N	Y	Y	tandem_dup	na	NA	intergenic	IGHG1,IGHG3	-,-	IG_C_gene,	17926;8105	intergenic	RP11-731F5.1,IGHG4	-,-	lincRNA,IG_	10973;8020
2146	chr14	106598867	+	chr14	106465542	+	N	Y	N	N	N	N	tandem_dup	na	NA	downstream	IGHVIII-13-1	-	IG_V_pseu	0	intergenic	IGHV1-2,IGHVIII-2-1	-,-	IG_V_gene,	12372;2135
2147	chr14	106598875	+	chr14	106465798	+	Y	N	Y	N	N	N	tandem_dup	na	NA	downstream	IGHVIII-13-1	-	IG_V_pseu	0	intergenic	IGHV1-2,IGHVIII-2-1	-,-	IG_V_gene,	12628;1879
2148	chr15	24799349	+	chr15	24486265	+	N	N	N	N	Y	N	tandem_dup	na	NA	ncRNA_intr	PWRN1	+	lincRNA	0	ncRNA_intr	RP11-580I1.2	+	lincRNA	0
2149	chr15	58777684	+	chr15	58776414	+	Y	Y	Y	Y	Y	Y	tandem_dup	na	NA	ncRNA_intr	RP11-355N15.1	-	antisense	0	ncRNA_intr	RP11-355N15.1	-	antisense	0
2150	chr15	77992104	+	chr15	77991666	+	Y	Y	N	N	Y	Y	tandem_dup	na	NA	intronic	LINGO1	-	protein_cod	0	intronic	LINGO1	-	protein_cod	0
2151	chr15	92293062	+	chr15	92292497	+	N	N	N	N	N	Y	tandem_dup	na	NA	intergenic	CTD-2343P13.1,snoU	-,+	pseudogene	5877;85547	intergenic	CTD-2343P13.1,snoU13	-,+	pseudogene	5312;86112
2152	chr15	92293435	+	chr15	92292535	+	Y	N	N	N	N	N	tandem_dup	na	NA	intergenic	CTD-2343P13.1,snoU	-,+	pseudogene	6250;85174	intergenic	CTD-2343P13.1,snoU13	-,+	pseudogene	5350;86074
2153	chr16	22700792	+	chr16	22699322	+	N	N	N	Y	N	N	tandem_dup	na	NA	intergenic	RN7SL245P,HS3ST2	-,+	misc_RNA,	52727;124706	intergenic	RN7SL245P,HS3ST2	-,+	misc_RNA,	51257;126176
2154	chr16	26386328	+	chr16	26385365	+	N	N	N	Y	Y	N	tandem_dup	na	NA	intergenic	AC130464.1,AC00233	-,-	pseudogene	16652;209748	intergenic	AC130464.1,AC002331.	-,-	pseudogene	15689;210711
2155	chr16	26386374	+	chr16	26385335	+	N	N	Y	N	N	N	tandem_dup	na	NA	intergenic	AC130464.1,AC00233	-,-	pseudogene	16698;209702	intergenic	AC130464.1,AC002331.	-,-	pseudogene	15659;210741
2156	chr16	46454874	+	chr16	46449683	+	N	N	N	N	N	Y	tandem_dup	na	NA	intergenic	NONE,ANKRD26P1	NONE,-	NONE,pseu	NONE;48379	intergenic	NONE,ANKRD26P1	NONE,-	NONE,pseu	NONE;53570
2157	chr16	48281375	+	chr16	48280923	+	Y	Y	Y	Y	Y	Y	tandem_dup	na	NA	intronic	LONP2,ABCC11	+,-	protein_cod	0	intronic	LONP2,ABCC11	+,-	protein_cod	0
2158	chr16	81799238	+	chr16	81798646	+	Y	Y	Y	Y	Y	Y	tandem_dup	na	NA	ncRNA_intr	RP11-960L18.1	+	lincRNA	0	ncRNA_intr	RP11-960L18.1	+	lincRNA	0
2159	chr17	6465395	+	chr17	6464556	+	N	N	N	N	Y	N	tandem_dup	na	NA	intergenic	PITPNM3,KIAA0753	-,-	protein_cod	5581;16073	intergenic	PITPNM3,KIAA0753	-,-	protein_cod	4742;16912
2160	chr17	25330716	+	chr17	21349671	+	N	N	N	N	Y	N	tandem_dup	na	NA	upstream	PDLIM1P3	-	pseudogene	0	upstream	PDLIM1P2	-	pseudogene	0
2161	chr17	41382500	+	chr17	992968	+	Y	N	Y	N	N	N	tandem_dup	na	NA	ncRNA_intr	LINC00854	-	processed_	0	intronic	ABR	-	protein_cod	0
2162	chr17	41382590	+	chr17	992878	+	N	Y	N	Y	Y	Y	tandem_dup	na	NA	ncRNA_intr	LINC00854	-	processed_	0	intronic	ABR	-	protein_cod	0
2163	chr17	62997003	+	chr17	62929876	+	Y	Y	Y	Y	Y	Y	tandem_dup	na	NA	intergenic	AMZ2P1,GNA13	-,-	pseudogene	25309;9830	ncRNA_intr	RP11-583F2.1	+	pseudogene	0
2164	chr17	67958084	+	chr17	67957684	+	Y	Y	Y	Y	Y	Y	tandem_dup	na	NA	ncRNA_intr	AC004562.1	+	lincRNA	0	upstream	AC004562.1	+	lincRNA	0
2165	chr17	72729574	+	chr17	72728508	+	N	N	N	Y	N	N	tandem_dup	na	NA	intronic	RAB37	+	protein_cod	0	intronic	RAB37	+	protein_cod	0
2166	chr17	73763302	+	chr17	73763001	+	Y	N	Y	N	Y	Y	tandem_dup	na	NA	intergenic	GALK1,H3F3B	-,-	protein_cod	1510;9213	intergenic	GALK1,H3F3B	-,-	protein_cod	1209;9514
2167	chr17	78904342	+	chr17	78904199	+	N	N	N	N	Y	N	tandem_dup	na	VNTR	intronic	RPTOR	+	protein_cod	0	intronic	RPTOR	+	protein_cod	0
2168	chr17	79399820	+	chr17	79398930	+	N	N	Y	N	N	N	tandem_dup	na	NA	intronic	RP11-1055B8.7	+	protein_cod	0	intronic	RP11-1055B8.7	+	protein_cod	0
2169	chr17	80035482	+	chr17	79589963	+	N	Y	Y	N	Y	Y	tandem_dup	na	NA	downstream	FASN	-	protein_cod	0	intronic	NPLOC4	-	protein_cod	0
2170	chr18	76799336	+	chr18	76798769	+	N	N	Y	N	N	N	tandem_dup	na	NA	intergenic	SALL3,ATP9B	+,+	protein_cod	36659;29949	intergenic	SALL3,ATP9B	+,+	protein_cod	36092;30516
2171	chr18	76799455	+	chr18	76798583	+	N	Y	N	N	N	N	tandem_dup	na	NA	intergenic	SALL3,ATP9B	+,+	protein_cod	36778;29830	intergenic	SALL3,ATP9B	+,+	protein_cod	35906;30702
2172	chr19	871610	+	chr19	447528	+	N	Y	Y	Y	Y	Y	tandem_dup	na	NA	exonic	MED16	-	protein_cod	0	intronic	SHC2	-	protein_cod	0
2173	chr19	7396504	+	chr19	7395968	+	N	N	Y	N	N	Y	tandem_dup	na	VNTR	intergenic	AC125387.1,CTB-133	+,+	miRNA,prot	87142;17344	intergenic	AC125387.1,CTB-133G6	+,+	miRNA,prot	86606;17880
2174	chr19	7516022	+	chr19	7515158	+	Y	N	N	N	N	N	tandem_dup	na	NA	intronic	ARHGEF18,CTD-2207	+,+	protein_cod	0	intronic	ARHGEF18,CTD-2207O	+,+	protein_cod	0
2175	chr19	13100622	+	chr19	13100004	+	N	Y	Y	Y	Y	N	tandem_dup	na	NA	intergenic	DAND5,NFIX	+,+	protein_cod	15055;5800	intergenic	DAND5,NFIX	+,+	protein_cod	14437;6418
2176	chr19	21010244	+	chr19	20993330	+	N	N	N	Y	N	N	tandem_dup	na	NA	intergenic	ZNF66,CTD-2542C24.	+,-	protein_cod	18322;11077	intergenic	ZNF66,CTD-2542C24.3	+,-	protein_cod	1408;27991
2177	chr19	21010358	+	chr19	20993526	+	N	N	N	N	Y	N	tandem_dup	na	NA	intergenic	ZNF66,CTD-2542C24.	+,-	protein_cod	18436;10963	intergenic	ZNF66,CTD-2542C24.3	+,-	protein_cod	1604;27795
2178	chr19	21010500	+	chr19	20993567	+	N	N	N	N	N	Y	tandem_dup	na	NA	intergenic	ZNF66,CTD-2542C24.	+,-	protein_cod	18578;10821	intergenic	ZNF66,CTD-2542C24.3	+,-	protein_cod	1645;27754

2179	chr19	22363750	+	chr19	22363077	+	N	Y	N	N	N	N	tandem_dup	na	NA	exonic	ZNF676	-	protein_cod	0	exonic	ZNF676	-	protein_cod	0
2180	chr19	22485656	+	chr19	20497144	+	Y	N	Y	N	Y	N	tandem_dup	na	NA	intronic	ZNF729	+	protein_cod	0	ncRNA_intr	ZNF826P	-	pseudogene	0
2181	chr19	23936465	+	chr19	23935892	+	N	Y	N	N	N	Y	tandem_dup	na	NA	intronic	ZNF681	-	protein_cod	0	intronic	ZNF681	-	protein_cod	0
2182	chr19	23936529	+	chr19	23935870	+	Y	N	Y	Y	Y	N	tandem_dup	na	NA	intronic	ZNF681	-	protein_cod	0	intronic	ZNF681	-	protein_cod	0
2183	chr19	28310253	+	chr19	23481928	+	N	N	Y	N	N	Y	tandem_dup	na	NA	ncRNA_intr	CTC-459F4.7,CTC-45	-,+,+	pseudogene	0	intergenic	VN1R91P,CTB-176F20.3	+,+	pseudogene	8109;24842
2184	chr19	34883230	+	chr19	34882967	+	N	N	N	N	N	Y	tandem_dup	na	NA	intronic	GPI	+	protein_cod	0	intronic	GPI	+	protein_cod	0
2185	chr19	34883321	+	chr19	34883075	+	N	N	N	Y	N	N	tandem_dup	na	NA	intronic	GPI	+	protein_cod	0	intronic	GPI	+	protein_cod	0
2186	chr19	34883500	+	chr19	34882888	+	N	N	Y	N	N	N	tandem_dup	na	NA	intronic	GPI	+	protein_cod	0	intronic	GPI	+	protein_cod	0
2187	chr19	43833267	+	chr19	43828009	+	N	N	N	N	Y	N	tandem_dup	na	NA	downstrear	CTC-490G23.2	-	lincRNA	0	intergenic	CEACAMP4,CTC-490G2	-,-	pseudogene	19334;5438
2188	chr19	43833288	+	chr19	43827783	+	N	N	N	Y	N	Y	tandem_dup	na	NA	downstrear	CTC-490G23.2	-	lincRNA	0	intergenic	CEACAMP4,CTC-490G2	-,-	pseudogene	19108;5664
2189	chr2	2754595	+	chr2	2754264	+	N	N	N	Y	N	N	tandem_dup	na	VNTR	intergenic	AC018685.2,AC01199	-,-	lincRNA,linc	19866;83441	intergenic	AC018685.2,AC011995.1	-,-	lincRNA,linc	19535;83772
2190	chr2	3184840	+	chr2	3184197	+	N	N	N	N	N	Y	tandem_dup	na	NA	intergenic	AC019118.4,TSSC1	+,-	lincRNA,pro	23272;7856	intergenic	AC019118.4,TSSC1	+,-	lincRNA,pro	22629;8499
2191	chr2	3184904	+	chr2	3184481	+	N	N	Y	N	N	N	tandem_dup	na	NA	intergenic	AC019118.4,TSSC1	+,-	lincRNA,pro	23336;7792	intergenic	AC019118.4,TSSC1	+,-	lincRNA,pro	22913;8215
2192	chr2	3184970	+	chr2	3184465	+	Y	N	N	N	N	N	tandem_dup	na	NA	intergenic	AC019118.4,TSSC1	+,-	lincRNA,pro	23402;7726	intergenic	AC019118.4,TSSC1	+,-	lincRNA,pro	22897;8231
2193	chr2	16407988	+	chr2	16406393	+	Y	Y	N	Y	Y	Y	tandem_dup	na	NA	ncRNA_intr	AC010745.2	+	lincRNA	0	ncRNA_intr	AC010745.2	+	lincRNA	0
2194	chr2	55155154	+	chr2	54594886	+	N	Y	Y	N	Y	Y	tandem_dup	na	NA	intronic	EML6	+	protein_cod	0	intronic	C2orf73	+	protein_cod	0
2195	chr2	55976296	+	chr2	55860446	+	N	N	N	N	N	Y	tandem_dup	na	NA	intergenic	PNPT1,EFEMP1	-,-	protein_cod	55251;116806	downstrear	PNPT1	-	protein_cod	0
2196	chr2	87593674	+	chr2	87587076	+	Y	N	N	N	N	N	tandem_dup	na	NA	intergenic	AC068279.2,AC06827	-,+	pseudogene	7307;2682	upstream	AC068279.2	-	pseudogene	0
2197	chr2	89877547	+	chr2	89876621	+	N	N	N	Y	N	N	tandem_dup	na	NA	intergenic	IGKV2-40,IGKV2D-40	-,+	IG_V_gene,	247361;13054	intergenic	IGKV2-40,IGKV2D-40	-,+	IG_V_gene,	246435;13980
2198	chr2	90450117	+	chr2	90449017	+	N	N	N	Y	N	Y	tandem_dup	na	NA	intergenic	AC113612.1,CH17-132	+,+	pseudogene	149715;8084	intergenic	AC113612.1,CH17-132F	+,+	pseudogene	148615;9184
2199	chr2	91671483	+	chr2	90449537	+	Y	N	N	N	N	N	tandem_dup	na	NA	intergenic	AC018696.1,IGKV1OR	+,+	pseudogene	35334;7604	intergenic	AC113612.1,CH17-132F	+,+	pseudogene	149135;8664
2200	chr2	118326427	+	chr2	118325651	+	N	N	Y	N	N	N	tandem_dup	na	NA	intergenic	AC092170.1,AC00931	-,-	pseudogene	387289;188712	intergenic	AC092170.1,AC009312.1	-,-	pseudogene	386513;189488
2201	chr2	131425938	+	chr2	131422564	+	N	N	Y	N	N	N	tandem_dup	na	NA	intergenic	POTEJ,CYP4F30P	+,+	protein_cod	10274;11685	intergenic	POTEJ,CYP4F30P	+,+	protein_cod	6900;15059
2202	chr2	131426172	+	chr2	131422234	+	N	Y	N	N	N	N	tandem_dup	na	NA	intergenic	POTEJ,CYP4F30P	+,+	protein_cod	10508;11451	intergenic	POTEJ,CYP4F30P	+,+	protein_cod	6570;15389
2203	chr2	131426325	+	chr2	131422479	+	N	N	N	N	N	Y	tandem_dup	na	NA	intergenic	POTEJ,CYP4F30P	+,+	protein_cod	10661;11298	intergenic	POTEJ,CYP4F30P	+,+	protein_cod	6815;15144
2204	chr2	132796151	+	chr2	132767555	+	N	N	N	N	N	N	tandem_dup	na	NA	upstream	AC093787.1	-	pseudogene	0	intergenic	RP1-156L9.1,AC093787.	-,-	lincRNA,pse	15821;27805
2205	chr2	133024413	+	chr2	62957828	+	N	N	Y	N	N	N	tandem_dup	na	NA	intergenic	CDC27P1,AC097532.2	+,-	pseudogene	3798;18955	intronic	EHBP1	+	protein_cod	0
2206	chr2	133024631	+	chr2	62958215	+	N	Y	N	N	Y	N	tandem_dup	na	NA	intergenic;ir	CDC27P1,AC097532.2	+,-	pseudogene	4016;18737	intronic	EHBP1	+	protein_cod	0
2207	chr2	133024631	+	chr2	62958281	+	N	N	N	Y	N	N	tandem_dup	na	NA	intergenic;ir	CDC27P1,AC097532.2	+,-	pseudogene	4016;18737	intronic	EHBP1	+	protein_cod	0
2208	chr2	133024754	+	chr2	62958148	+	Y	N	N	N	N	Y	tandem_dup	na	NA	intergenic	CDC27P1,AC097532.2	+,-	pseudogene	4139;18614	intronic	EHBP1	+	protein_cod	0
2209	chr2	206498633	+	chr2	206494752	+	Y	N	N	N	N	N	tandem_dup	na	NA	intergenic	PARD3B,NRP2	+,+	protein_cod	13747;48591	intergenic	PARD3B,NRP2	+,+	protein_cod	9866;52472
2210	chr2	220180587	+	chr2	208472402	+	N	N	N	Y	N	N	tandem_dup	na	NA	intergenic	PTPRN,RESP18	-,-	protein_cod	6217;11544	intronic	METTL21A	-	protein_cod	0
2211	chr2	234696801	+	chr2	13211651	+	Y	N	N	N	N	N	tandem_dup	na	NA	intronic	MROH2A	+	protein_cod	0	intergenic	AC064875.2,AC092635.1	-,+	processed_	64513;466147
2212	chr2	240564997	+	chr2	240563718	+	N	N	Y	N	N	N	tandem_dup	na	NA	intergenic	AC079612.2,RP11-315	+,+	lincRNA,linc	12464;91653	intergenic	AC079612.2,RP11-315F2	+,+	lincRNA,linc	11185;92932
2213	chr2	240565099	+	chr2	240563735	+	Y	N	N	N	N	N	tandem_dup	na	NA	intergenic	AC079612.2,RP11-315	+,+	lincRNA,linc	12566;91551	intergenic	AC079612.2,RP11-315F2	+,+	lincRNA,linc	11202;92915
2214	chr2	240565154	+	chr2	240564080	+	N	Y	N	N	N	N	tandem_dup	na	NA	intergenic	AC079612.2,RP11-315	+,+	lincRNA,linc	12621;91496	intergenic	AC079612.2,RP11-315F2	+,+	lincRNA,linc	11547;92570
2215	chr2	241467200	+	chr2	241466439	+	N	Y	Y	Y	Y	N	tandem_dup	na	VNTR	intronic;intrc	ANKMY1	-	protein_cod	0	intronic	ANKMY1	-	protein_cod	0
2216	chr2	241467200	+	chr2	241466510	+	Y	N	N	N	N	N	tandem_dup	na	VNTR	intronic;intrc	ANKMY1	-	protein_cod	0	intronic	ANKMY1	-	protein_cod	0
2217	chr20	16169497	+	chr20	16169195	+	N	N	N	N	Y	N	tandem_dup	na	VNTR	intergenic	MACROD2,PPIAP17	+,-	protein_cod	135655;34898	intergenic	MACROD2,PPIAP17	+,-	protein_cod	135353;35200
2218	chr20	29830924	+	chr20	29813239	+	Y	N	N	Y	N	N	tandem_dup	na	NA	intergenic	MLLT10P1,DEFB115	-,+	pseudogene	192864;14543	intergenic	MLLT10P1,DEFB115	-,+	pseudogene	175179;32228
2219	chr20	37092964	+	chr20	37092140	+	Y	N	N	N	N	N	tandem_dup	na	NA	intergenic	SNHG11,RALGAPB	+,+	processed_	13400;8495	intergenic	SNHG11,RALGAPB	+,+	processed_	12576;9319
2220	chr20	42325670	+	chr20	42325099	+	Y	Y	Y	Y	Y	Y	tandem_dup	na	NA	intronic	MYBL2	+	protein_cod	0	intronic	MYBL2	+	protein_cod	0
2221	chr20	57270229	+	chr20	57270064	+	Y	Y	N	Y	Y	N	tandem_dup	na	VNTR	intronic	NPEPL1,STX16-NPEF	+,+	protein_cod	0	intronic	NPEPL1,STX16-NPEPL1	+,+	protein_cod	0
2222	chr20	57270279	+	chr20	57270073	+	N	N	N	N	N	Y	tandem_dup	na	NA	intronic	NPEPL1,STX16-NPEF	+,+	protein_cod	0	intronic	NPEPL1,STX16-NPEPL1	+,+	protein_cod	0
2223	chr20	59076070	+	chr20	59075598	+	N	Y	N	N	N	N	tandem_dup	na	NA	ncRNA_intr	RP5-1043L13.1	+	lincRNA	0	ncRNA_intr	RP5-1043L13.1	+	lincRNA	0
2224	chr20	59958947	+	chr20	59958154	+	N	N	N	N	Y	Y	tandem_dup	na	NA	intronic	CDH4	+	protein_cod	0	intronic	CDH4	+	protein_cod	0
2225	chr20	59958949	+	chr20	59958117	+	N	N	Y	Y	N	N	tandem_dup	na	NA	intronic	CDH4	+	protein_cod	0	intronic	CDH4	+	protein_cod	0
2226	chr22	18589210	+	chr22	18588921	+	N	N	N	N	Y	N	tandem_dup	na	NA	intronic	PEX26	+	protein_cod	0	intronic	PEX26	+	protein_cod	0
2227	chr22	18589228	+	chr22	18588843	+	N	N	Y	N	N	N	tandem_dup	na	NA	intronic	PEX26	+	protein_cod	0	intronic	PEX26	+	protein_cod	0
2228	chr22	22729292	+	chr22	22706512	+	N	N	N	Y	N	N	tandem_dup	na	NA	intergenic	LL22NC03-22A12.12,I	+,+	pseudogene	3804;1063	upstream	IGLV5-48	+	IG_V_gene	0
2229	chr22	22729334	+	chr22	22706359	+	N	N	Y	N	Y	N	tandem_dup	na	NA	intergenic	LL22NC03-22A12.12,I	+,+	pseudogene	3846;1021	upstream	IGLV5-48	+	IG_V_gene	0
2230	chr22	22752616	+	chr22	22703167	+	Y	Y	N	Y	Y	Y	tandem_dup	na	NA	upstream;dc	IGLVVII-41-1,IGLVI-42	+,+	IG_V_pseu	0	downstrear	LL22NC03-75A1.9	+	pseudogene	0
2231	chr22	25056879	+	chr22	22987359	+	N	N	N	N	Y	N	tandem_dup	na	NA	intergenic	POM121L10P,ARL5AF	-,+	pseudogene	1765;25256	upstream	POM121L1P	-	pseudogene	0
2232	chr22	25673559	+	chr22	24713331	+	N	Y	N	N	N	N	tandem_dup	na	NA	intergenic	CRYBB2,RP3-462D8.2	+,-	protein_cod	45723;1937	intronic	SPECC1L,SPECC1L-AD	+,+	protein_cod	0
2233	chr22	25673859	+	chr22	24712926	+	N	N	N	N	N	Y	tandem_dup	na	NA	intergenic	CRYBB2,RP3-462D8.2	+,-	protein_cod	46023;1637	intronic	SPECC1L,SPECC1L-AD	+,+	protein_cod	0
2234	chr22	25988830	+	chr22	25692175	+	N	N	Y	Y	N	N	tandem_dup	na	NA	intronic	ADRBK2	+	protein_cod	0	intergenic	RP3-462D8.2,IGLL3P	-,+	lincRNA,pse	13534;22048
2235	chr22	29527232	+	chr22	29242778	+	N	Y	Y	Y	Y	Y	tandem_dup	na	NA	intronic	KREMEN1	+	protein_cod	0	ncRNA_intr	CTA-292E10.6	+	antisense	0
2236	chr22	41964071	+	chr22	41960142	+	N	Y	N	Y	Y	Y	tandem_dup	na	NA	intronic	CSDC2	+	protein_cod	0	intronic	CSDC2	+	protein_cod	0
2237	chr22	45746425	+	chr22	45746291	+	N	N	N	N	N	Y	tandem_dup	na	NA	intronic	SMC1B	-	protein_cod	0	intronic	SMC1B	-	protein_cod	0
2238	chr22	45746494	+	chr22	45745363	+	N	Y	N	N	N	N	tandem_dup	na	NA	intronic	SMC1B	-	protein_cod	0	intronic	SMC1B	-	protein_cod	0
2239	chr3	1032768	+	chr3	1032315	+	N	Y	N	Y	N	N	tandem_dup	na	VNTR	intergenic	AC090044.2,AC08743	+,+	lincRNA,linc	132994;17051	intergenic	AC090044.2,AC087430.1	+,+	lincRNA,linc	132541;17504
2240	chr3	11911992	+	chr3	10088139	+	Y	N	N	N	N	N	tandem_dup	na	NA	ncRNA_intr	FANCD2P2	+	pseudogene	0	intronic	FANCD2	+	protein_cod	0
2241	chr3	11984798	+	chr3	11984145	+	N	Y	N	N	N	N	tandem_dup	na	NA	intergenic	MARK2P14,RN7SL147	+,-	pseudogene	31011;9466	intergenic	MARK2P14,RN7SL147P	+,-	pseudogene	30358;10119
2242	chr3	14650366	+	chr3	14637994	+	N	Y	Y	Y	Y	Y	tandem_dup	na	NA	intergenic	RHBDF1P1,AC090952	-,-	pseudogene	34067;39335	intergenic	RHBDF1P1,AC090952.5	-,-	pseudogene	21695;51707
2243	chr3	57524041	+	chr3	57522647	+	Y	N	N	N	N	N	tandem_dup	na	NA	intronic;intrc	DNAH12	-	protein_cod	0	intronic;intrc	DNAH12	-	protein_cod	0
2244	chr3	57524052	+	chr3	57522710	+	N	N	N	Y	N	Y	tandem_dup	na	NA	intronic	DNAH12	-	protein_cod	0	intronic	DNAH12	-	protein_cod	0
2245	chr3	194664345	+	chr3	194663944	+	N	N	N	N	N	Y	tandem_dup	na	NA	intergenic	AC090505.1,XXYLT1	+,-	lincRNA,pro	111024;124663	intergenic	AC090505.1,XXYLT1	+,-	lincRNA,pro	110623;125064
2246	chr3	195226045	+	chr3	195225081	+	N	N	Y	N	N	N	tandem_dup	na	NA	intergenic	ACAP2,PPP1R2	-,-	protein_cod	62238;15176	intergenic	ACAP2,PPP1R2	-,-	protein_cod	61274;16140
2247	chr3	195727815	+	chr3	195667661	+	N	N	Y	N	N	N	tandem_dup	na	NA	intergenic	AC024937.4,TFRC	+,-	pseudogene	2711;26239	intergenic	AC024937.6,RP11-480A	+,-	pseudogene	2051;8398
2248	chr3	195728270	+	chr3	195667790	+	N	N	N	N	N	Y	tandem_dup	na	NA	intergenic	AC024937.4,TFRC	+,-	pseudogene	3166;25784	intergenic	AC024937.6,RP11-480A	+,-	pseudogene	2180;8269
2249	chr3	197114920	+	chr3	197112594	+	N	N	N	N	Y	N	tandem_dup	na	NA	intergenic	snoU13,AC128709.3	-,-	snoRNA,linc	48498;57012	intergenic	snoU13,AC128709.3	-,-	snoRNA,linc	46172;59338
2250	chr4	74116	+	chr4	73133	+	Y	Y	Y	Y	Y	Y	tandem_dup	na	NA	intergenic	ZNF595,Z95704.2	+,+	protein_cod	5724;11505	intergenic	ZNF595,Z95704.2	+,+	protein_cod	4741;12488
2251	chr4	2338736	+	chr4	2338364	+	N	Y	N	N	N	N	tandem_dup	na	NA	intronic	ZFYVE28	-	protein_cod	0	intronic	ZFYVE28	-	protein_cod	0
2252	chr4	32074611	+	chr4	32071041	+	N	N	N	Y	N	N	tandem_dup	na	NA	ncRNA_intr	RP11-734I18.1	+	lincRNA	0	ncRNA_intr	RP11-734I18.1	+	lincRNA	0
2253	chr4	49129130	+	chr4	49119669	+	N	Y	N	N	N	N	tandem_dup	na	NA	intergenic	CWH43,AC118282.3	+,+	protein_cod	65032;70090	intergenic	CWH43,AC118282.3	+,+	protein_cod	55571;79551
2254	chr4	49136842	+	chr4	49119675	+	N	N	Y	N	N	N	tandem_dup	na	NA	intergenic	CWH43,AC118282.3	+,+	protein_cod	72744;62378	intergenic	CWH43,AC118282.3	+,+	protein_cod	55577;79545
2255	chr4	74253782	+	chr4	73841403	+	N	Y	Y	Y	Y	Y	tandem_dup	na	NA	intergenic	RP11-580P21.1,ALB	-,+	pseudogene	50294;9049	intergenic	RNU4ATAC9P,RNU6ATA	+,+	snRNA,snR	10383;51062
2256	chr4	144894290	+	chr4	144773210	+	N	Y	N	N	N	N	tandem_dup	na	NA	ncRNA_exc	RP11-673E1.1	+	lincRNA	0	ncRNA_intr	RP13-578N3.3	+	antisense	0
2257	chr4	188427150	+	chr4	187246124	+	N	N	N	N	Y	N	tandem_dup	na	NA	intergenic	RP11-91J3.1,RP11-23	-,-	pseudogene	61698;10157	ncRNA_intr	F11-AS1	-	antisense	0
2258	chr4	190607294	+	chr4	190606114	+	N	Y	N	N	N	N	tandem_dup	na	NA	intergenic	RP11-462G22.1,RP11-	+,-	lincRNA,linc	24654;17741	intergenic	RP11-462G22.1,RP11-46	+,-	lincRNA,linc	23474;18921
2259	chr5	282814	+	chr5	282594	+	N	N	N	N	Y	N	tandem_dup	na	NA	intronic	PDCD6	+	protein_cod	0	intronic	PDCD6	+	protein_cod	0
2260	chr5	754683	+	chr5	754511	+	N	N	Y	N	N	N	tandem_dup	na	NA	intronic	ZDHHC11,ZDHHC11B	-,-	protein_cod	0	intronic	ZDHHC11,ZDHHC11B	-,-	protein_cod	0
2261	chr5	774688	+	chr5	682597	+	N	N	N	N	Y	N	tandem_dup	na	NA	intronic	ZDHHC11	-	protein_cod	0	intronic	TPPP	-	protein_cod	0
2262	chr5	774714	+	chr5	682433	+	N	N	Y	N	N	N	tandem_dup	na	NA	intronic	ZDHHC11	-	protein_cod	0	intronic	TPPP	-	protein_cod	0

2263	chr5	45351337	+	chr5	45317364	+	N	Y	N	N	N	N	tandem_dup	na	NA	intronic	HCN1	-	protein_cod	0	intronic	HCN1	-	protein_cod	0
2264	chr5	98872008	+	chr5	21496124	+	N	Y	N	N	N	N	tandem_dup	na	NA	intergenic	GUSBP8,CTD-2151A2	+,+	pseudogene	1693;13128	ncRNA_intr	GUSBP1	+	pseudogene	0
2265	chr5	101429114	+	chr5	101427916	+	N	Y	N	Y	N	Y	tandem_dup	na	NA	intergenic	RP11-1152B5.1,RNA5	+,-	pseudogene	116612;37597	intergenic	RP11-1152B5.1,RNA5SP	+,-	pseudogene	115414;38795
2266	chr5	101429140	+	chr5	101427996	+	Y	N	Y	N	Y	N	tandem_dup	na	NA	intergenic	RP11-1152B5.1,RNA5	+,-	pseudogene	116638;37571	intergenic	RP11-1152B5.1,RNA5SP	+,-	pseudogene	115494;38715
2267	chr5	147688079	+	chr5	147640926	+	Y	Y	Y	Y	Y	Y	tandem_dup	na	NA	ncRNA_intr	RP11-373N22.3	-	antisense	0	intergenic	CTC-295J13.3,SPINK13	+,+	lincRNA,pro	18769;6817
2268	chr5	164609911	+	chr5	151362181	+	N	Y	N	N	N	N	tandem_dup	na	NA	intergenic	CTC-340A15.2,CTB-1	+,-	antisense,li	11262;37672	ncRNA_intr	CTB-12O2.1	+	lincRNA	0
2269	chr6	3268813	+	chr6	2825712	+	N	Y	N	N	Y	Y	tandem_dup	na	NA	intronic	PSMG4	+	protein_cod	0	intergenic	WRNIP1,SERPINB1	+,-	protein_cod	38526;6854
2270	chr6	35766789	+	chr6	35754559	+	Y	Y	Y	Y	Y	Y	tandem_dup	na	NA	intergenic	CLPS,LHFPL5	-,+	protein_cod	1701;6281	intronic	CLPSL1	+	protein_cod	0
2271	chr6	66465180	+	chr6	66464364	+	N	N	N	N	Y	N	tandem_dup	na	NA	intergenic	EYS,SLC25A51P1	-,+	protein_cod	48062;33130	intergenic	EYS,SLC25A51P1	-,+	protein_cod	47246;33946
2272	chr6	66465284	+	chr6	66464372	+	N	N	N	Y	N	N	tandem_dup	na	NA	intergenic	EYS,SLC25A51P1	-,+	protein_cod	48166;33026	intergenic	EYS,SLC25A51P1	-,+	protein_cod	47254;33938
2273	chr6	66465292	+	chr6	66464445	+	N	N	Y	N	N	N	tandem_dup	na	NA	intergenic	EYS,SLC25A51P1	-,+	protein_cod	48174;33018	intergenic	EYS,SLC25A51P1	-,+	protein_cod	47327;33865
2274	chr6	93743740	+	chr6	93706817	+	N	Y	N	N	N	N	tandem_dup	na	NA	intergenic	ATF1P1,RP1-23E21.2	-,+	pseudogene	145441;36365	intergenic	ATF1P1,RP1-23E21.2	-,+	pseudogene	108518;73288
2275	chr6	93743804	+	chr6	93706485	+	Y	N	N	N	N	N	tandem_dup	na	NA	intergenic	ATF1P1,RP1-23E21.2	-,+	pseudogene	145505;36301	intergenic	ATF1P1,RP1-23E21.2	-,+	pseudogene	108186;73620
2276	chr6	93743870	+	chr6	93706892	+	N	N	N	N	Y	N	tandem_dup	na	NA	intergenic	ATF1P1,RP1-23E21.2	-,+	pseudogene	145571;36235	intergenic	ATF1P1,RP1-23E21.2	-,+	pseudogene	108593;73213
2277	chr6	106930227	+	chr6	106929717	+	Y	Y	Y	Y	Y	Y	tandem_dup	na	NA	intergenic	Y_RNA,AIM1	+,+	misc_RNA,p	27423;29503	intergenic	Y_RNA,AIM1	+,+	misc_RNA,p	26913;30013
2278	chr6	119262321	+	chr6	119261990	+	Y	N	Y	Y	N	Y	tandem_dup	na	NA	ncRNA_intr	RP11-351A11.1	+	antisense	0	ncRNA_intr	RP11-351A11.1	+	antisense	0
2279	chr6	150521381	+	chr6	2376481	+	N	N	N	Y	N	N	tandem_dup	na	NA	intronic	PPP1R14C	+	protein_cod	0	ncRNA_intr	GMDS-AS1	+	lincRNA	0
2280	chr6	160535392	+	chr6	158630560	+	N	N	Y	N	N	N	tandem_dup	na	NA	intergenic	RP11-288H12.4,SLC2	+,+	lincRNA,pro	2856;7429	intergenic	GTF2H5,SRP72P2	+,-	protein_cod	10184;27808
2281	chr6	160640129	+	chr6	160639541	+	N	Y	Y	Y	N	N	tandem_dup	na	NA	intronic	SLC22A2	-	protein_cod	0	intronic	SLC22A2	-	protein_cod	0
2282	chr6	160956501	+	chr6	160877824	+	Y	N	N	Y	N	N	tandem_dup	na	NA	intronic	LPA	-	protein_cod	0	ncRNA_intr	LPAL2	-	pseudogene	0
2283	chr6	166863912	+	chr6	166863327	+	Y	N	N	N	N	N	tandem_dup	na	NA	intronic	RPS6KA2	-	protein_cod	0	intronic	RPS6KA2	-	protein_cod	0
2284	chr6	166864285	+	chr6	166863286	+	N	Y	N	N	N	N	tandem_dup	na	NA	intronic	RPS6KA2	-	protein_cod	0	intronic	RPS6KA2	-	protein_cod	0
2285	chr6	167846904	+	chr6	167846743	+	N	N	Y	N	N	N	tandem_dup	na	VNTR	intergenic	TCP10,RP11-351J23.2	-,-	protein_cod	48906;220616	intergenic	TCP10,RP11-351J23.2	-,-	protein_cod	48745;220777
2286	chr6	168520654	+	chr6	168520244	+	N	N	N	N	N	Y	tandem_dup	na	NA	intergenic	FRMD1,RP11-503C24	-,+	protein_cod	38417;74384	intergenic	FRMD1,RP11-503C24.1	-,+	protein_cod	38007;74794
2287	chr6	169060758	+	chr6	169060159	+	N	N	N	N	Y	N	tandem_dup	na	NA	intronic;intrc	SMOC2	+	protein_cod	0	intronic	SMOC2	+	protein_cod	0
2288	chr6	169060758	+	chr6	169060249	+	N	N	Y	N	N	N	tandem_dup	na	NA	intronic;intrc	SMOC2	+	protein_cod	0	intronic	SMOC2	+	protein_cod	0
2289	chr6	169060772	+	chr6	169060063	+	N	N	N	Y	N	N	tandem_dup	na	NA	intronic	SMOC2	+	protein_cod	0	intronic	SMOC2	+	protein_cod	0
2290	chr7	522865	+	chr7	522505	+	N	N	N	N	Y	N	tandem_dup	na	NA	downstream	AC147651.1	-	lincRNA	0	intergenic	AC226118.1,AC147651.1	+,-	lincRNA,linc	99827;1239
2291	chr7	523278	+	chr7	522247	+	N	Y	N	N	N	Y	tandem_dup	na	NA	downstream	AC147651.1	-	lincRNA	0	intergenic	AC226118.1,AC147651.1	+,-	lincRNA,linc	99569;1497
2292	chr7	523279	+	chr7	522504	+	Y	N	Y	Y	N	N	tandem_dup	na	NA	downstream	AC147651.1	-	lincRNA	0	intergenic	AC226118.1,AC147651.1	+,-	lincRNA,linc	99826;1240
2293	chr7	47709533	+	chr7	47708717	+	Y	Y	Y	Y	Y	Y	tandem_dup	na	NA	intergenic	C7orf65,HUS1	+,-	protein_cod	8287;25795	intergenic	C7orf65,HUS1	+,-	protein_cod	7471;26611
2294	chr7	52397660	+	chr7	52171400	+	N	N	N	N	N	Y	tandem_dup	na	NA	intergenic	snoU13,RP11-398K14.	-,-	snoRNA,ps	6075;561870	intergenic	RN7SL292P,RP11-153N	+,-	misc_RNA,l	386785;61531
2295	chr7	52397913	+	chr7	52171569	+	N	Y	N	N	N	N	tandem_dup	na	NA	intergenic	snoU13,RP11-398K14.	-,-	snoRNA,ps	6328;561617	intergenic	RN7SL292P,RP11-153N	+,-	misc_RNA,l	386954;61362
2296	chr7	56186102	+	chr7	45810468	+	N	N	Y	N	N	N	tandem_dup	na	NA	intergenic	NUPR1L,RP4-725G10	-,+	protein_cod	2009;44736	intergenic	SEPT7P2,AC096582.7	-,+	pseudogene	1851;2726
2297	chr7	56894863	+	chr7	56890912	+	N	Y	N	N	Y	N	tandem_dup	na	NA	intergenic	RP11-10F11.2,RP13-5	-,-	pseudogene	176474;48215	intergenic	RP11-10F11.2,RP13-580	-,-	pseudogene	172523;52166
2298	chr7	57477926	+	chr7	56557273	+	Y	Y	N	N	N	N	tandem_dup	na	NA	intergenic	MIR3147,VN1R28P	+,-	miRNA,pseu	5130;4608	intergenic	RP11-760D2.1,RBM22P	-,+	lincRNA,ps	6285;1102
2299	chr7	57477938	+	chr7	56557238	+	N	N	Y	Y	Y	N	tandem_dup	na	NA	intergenic	MIR3147,VN1R28P	+,-	miRNA,pseu	5142;4596	intergenic	RP11-760D2.1,RBM22P	-,+	lincRNA,ps	6250;1137
2300	chr7	62135350	+	chr7	62133392	+	N	N	N	N	Y	N	tandem_dup	na	NA	intergenic	RP11-715L17.1,RNU6	+,-	pseudogene	313164;336491	intergenic	RP11-715L17.1,RNU6-41	+,-	pseudogene	311206;338449
2301	chr7	62382194	+	chr7	62369709	+	N	N	N	N	N	Y	tandem_dup	na	NA	intergenic	RP11-715L17.1,RNU6	+,-	pseudogene	560008;89647	intergenic	RP11-715L17.1,RNU6-41	+,-	pseudogene	547523;102132
2302	chr7	62518893	+	chr7	55830728	+	Y	Y	Y	N	Y	N	tandem_dup	na	NA	ncRNA_intr	RP11-196D18.2	+	pseudogene	0	intergenic	CICP11,PSPHP1	+,+	pseudogene	23439;1762
2303	chr7	63818249	+	chr7	63548202	+	N	N	Y	N	N	N	tandem_dup	na	NA	intergenic	TRIM60P18,YWHAEP	+,+	pseudogene	1672;75959	intergenic	TRIM60P17,GUSBP6	+,+	pseudogene	1248;12481
2304	chr7	63818256	+	chr7	63548407	+	N	N	N	N	Y	N	tandem_dup	na	NA	intergenic	TRIM60P18,YWHAEP	+,+	pseudogene	1679;75952	intergenic	TRIM60P17,GUSBP6	+,+	pseudogene	1453;12276
2305	chr7	65262811	+	chr7	65262467	+	N	Y	N	N	Y	Y	tandem_dup	na	NA	ncRNA_intr	RP11-479O9.2	+	pseudogene	0	ncRNA_intr	RP11-479O9.2	+	pseudogene	0
2306	chr7	65309770	+	chr7	63238863	+	Y	Y	N	Y	Y	N	tandem_dup	na	NA	intergenic;ir	RP13-254B10.1,RNU6	+,-	pseudogene	4187;14877	intergenic;ir	CICP24,CTD-2526L21.3	-,+	pseudogene	7438;11424
2307	chr7	73381928	+	chr7	66193705	+	Y	N	N	N	N	N	tandem_dup	na	NA	intergenic	WBSCR28,RP11-731K	+,+	protein_cod	101705;18394	intronic	RABGEF1,KCTD7	+,+	protein_cod	0
2308	chr7	76628315	+	chr7	76128808	+	Y	N	Y	Y	Y	Y	tandem_dup	na	NA	ncRNA_intr	DTX2P1,DTX2P1-UPK	+,+	pseudogene	0	intronic	DTX2	+	protein_cod	0
2309	chr7	88518949	+	chr7	88516540	+	N	N	N	N	N	Y	tandem_dup	na	NA	intronic	ZNF804B	+	protein_cod	0	intronic	ZNF804B	+	protein_cod	0
2310	chr7	100550782	+	chr7	100550529	+	N	N	N	N	N	Y	tandem_dup	na	NA	upstream;dc	MUC3A,MUC3A	+,+	protein_cod	0	upstream;dc	MUC3A,MUC3A	+,+	protein_cod	0
2311	chr7	140189221	+	chr7	140181588	+	N	Y	N	N	Y	Y	tandem_dup	na	NA	intergenic;ir	MKRN1,DENND2A	-,-	protein_cod	9852;28999	intergenic;ir	MKRN1,DENND2A	-,-	protein_cod	2219;36632
2312	chr7	142044613	+	chr7	142011954	+	N	N	Y	N	N	N	tandem_dup	na	NA	upstream	TRBV4-2	+	TR_V_gene	0	intergenic	TRBV3-1,TRBV4-1	+,+	TR_V_gene	3083;1053
2313	chr7	142045993	+	chr7	142013497	+	Y	Y	Y	Y	N	N	tandem_dup	na	NA	downstream	TRBV4-2	+	TR_V_gene	0	downstream	TRBV4-1	+	TR_V_gene	0
2314	chr7	142145357	+	chr7	142125856	+	Y	N	N	N	N	Y	tandem_dup	na	NA	intergenic	TRBV6-7,TRBV5-5	-,-	TR_V_gene	1273;3571	intergenic	TRBV6-8,TRBV5-6	-,-	TR_V_gene	1291;5556
2315	chr7	142170543	+	chr7	142133564	+	Y	Y	Y	Y	Y	Y	tandem_dup	na	NA	intergenic	TRBV5-4,TRBV7-4	-,-	TR_V_gene	1498;5786	intergenic	TRBV5-6,TRBV7-6	-,-	TR_V_gene	1646;5714
2316	chr7	142180607	+	chr7	142124228	+	N	N	N	Y	Y	Y	tandem_dup	na	NA	exonic	TRBV6-5	-	TR_V_gene	0	exonic	TRBV6-8	-	TR_V_gene	0
2317	chr7	142216934	+	chr7	142191267	+	Y	Y	Y	N	Y	N	tandem_dup	na	NA	upstream;u	TRBV12-1	-	TR_V_pseu	0	upstream;u	TRBV12-2	-	TR_V_pseu	0
2318	chr7	142223548	+	chr7	142196392	+	N	Y	Y	N	N	N	tandem_dup	na	NA	downstream	TRBV11-1	-	TR_V_gene	0	intergenic	TRBV12-2,TRBV11-2	-,-	TR_V_pseu	5312;1178
2319	chr7	142471961	+	chr7	142461138	+	N	N	N	N	Y	N	tandem_dup	na	NA	downstream	PRSS3P1	+	pseudogene	0	downstream	PRSS1	+	protein_cod	0
2320	chr7	142472003	+	chr7	142461082	+	N	N	Y	Y	N	N	tandem_dup	na	NA	downstream	PRSS3P1	+	pseudogene	0	downstream	PRSS1	+	protein_cod	0
2321	chr7	149738678	+	chr7	149735974	+	N	Y	N	N	N	N	tandem_dup	na	NA	ncRNA_intr	RP11-545G3.1	+	lincRNA	0	intergenic	RP11-728K20.2,RP11-54	+,+	lincRNA,linc	33761;1636
2322	chr7	156124733	+	chr7	156124609	+	N	N	Y	N	N	Y	tandem_dup	na	NA	intergenic	RP11-362B23.1,AC07	+,-	pseudogene	68237;100484	intergenic	RP11-362B23.1,AC0731	+,-	pseudogene	68113;100608
2323	chr7	158130668	+	chr7	158123828	+	N	Y	N	N	N	N	tandem_dup	na	NA	intronic	PTPRN2	-	protein_cod	0	intronic	PTPRN2	-	protein_cod	0
2324	chr8	1237052	+	chr8	1236918	+	N	Y	N	N	N	N	tandem_dup	na	NA	intergenic	CTD-2281E23.2,CTD-	+,-	processed_	24472;7240	intergenic	CTD-2281E23.2,CTD-22	+,-	processed_	24338;7374
2325	chr8	47667871	+	chr8	47655520	+	Y	N	N	Y	N	N	tandem_dup	na	NA	intergenic	ASNSP4,RP11-101E1	+,+	pseudogene	57561;28551	intergenic	ASNSP4,RP11-101E19.8	+,+	pseudogene	45210;40902
2326	chr8	49060982	+	chr8	49060764	+	N	N	N	N	Y	N	tandem_dup	na	NA	intergenic	UBE2V2,RP11-769N2	+,-	protein_cod	83714;45428	intergenic	UBE2V2,RP11-769N21.2	+,-	protein_cod	83496;45646
2327	chr8	49061266	+	chr8	49060755	+	N	N	Y	N	N	N	tandem_dup	na	NA	intergenic	UBE2V2,RP11-769N2	+,-	protein_cod	83998;45144	intergenic	UBE2V2,RP11-769N21.2	+,-	protein_cod	83487;45655
2328	chr8	58123054	+	chr8	58116317	+	Y	Y	Y	N	Y	Y	tandem_dup	na	NA	ncRNA_intr	RP11-513O17.2	+	lincRNA	0	ncRNA_intr	RP11-513O17.2	+	lincRNA	0
2329	chr8	58128150	+	chr8	58118227	+	N	Y	N	N	N	N	tandem_dup	na	NA	ncRNA_intr	RP11-513O17.2	+	lincRNA	0	ncRNA_intr	RP11-513O17.2	+	lincRNA	0
2330	chr8	63677286	+	chr8	28542461	+	N	N	Y	N	N	N	tandem_dup	na	NA	intronic	NKAIN3	+	protein_cod	0	intronic	EXTL3	+	protein_cod	0
2331	chr8	80136498	+	chr8	80028977	+	N	Y	N	N	N	N	tandem_dup	na	NA	intergenic	AC009941.1,RP11-111	+,+	miRNA,lincF	126810;73454	intergenic	AC009941.1,RP11-1114I	+,+	miRNA,lincF	19289;180975
2332	chr8	142565261	+	chr8	142565046	+	Y	N	N	N	N	N	tandem_dup	na	NA	intergenic	AC138647.1,AC10441	+,+	protein_cod	36424;461599	intergenic	AC138647.1,AC104417.1	+,+	protein_cod	36209;461814
2333	chr8	145180952	+	chr8	145180479	+	N	Y	N	N	N	N	tandem_dup	na	NA	intergenic	KIAA1875,FAM203A	+,+	protein_cod	7734;11720	intergenic	KIAA1875,FAM203A	+,+	protein_cod	7261;12193
2334	chr8	145181024	+	chr8	145180564	+	N	N	N	Y	N	N	tandem_dup	na	NA	intergenic	KIAA1875,FAM203A	+,+	protein_cod	7806;11648	intergenic	KIAA1875,FAM203A	+,+	protein_cod	7346;12108
2335	chr9	4190155	+	chr9	3107292	+	N	N	Y	N	N	N	tandem_dup	na	NA	intronic	GLIS3	-	protein_cod	0	intergenic	CARM1P1,RP11-32F11.2	-,+	pseudogene	53884;74297
2336	chr9	4190173	+	chr9	3107498	+	N	Y	N	N	N	N	tandem_dup	na	NA	intronic	GLIS3	-	protein_cod	0	intergenic	CARM1P1,RP11-32F11.2	-,+	pseudogene	54090;74091
2337	chr9	4714202	+	chr9	4713963	+	N	N	Y	N	N	N	tandem_dup	na	VNTR	intronic	AK3	-	protein_cod	0	intronic	AK3	-	protein_cod	0
2338	chr9	4714246	+	chr9	4714003	+	N	N	N	N	Y	N	tandem_dup	na	NA	intronic	AK3	-	protein_cod	0	intronic	AK3	-	protein_cod	0
2339	chr9	34681979	+	chr9	34681480	+	Y	Y	Y	Y	Y	Y	tandem_dup	na	NA	downstream	RP11-195F19.9	+	antisense	0	downstream	RP11-195F19.9	+	antisense	0
2340	chr9	68998466	+	chr9	68989339	+	Y	N	N	N	N	Y	tandem_dup	na	NA	upstream	AL353763.2	-	miRNA	0	intergenic	PGM5P1,AL353763.2	-,-	pseudogene	179433;8070
2341	chr9	70402972	+	chr9	69785871	+	N	N	N	N	N	Y	tandem_dup	na	NA	intergenic	IGKV1OR-3,FOXD4L4	-,-	IG_V_pseu	7882;23651	intergenic	IGKV1OR-2,AL359955.1	-,-	IG_V_pseu	7918;35889
2342	chr9	70403658	+	chr9	69785803	+	N	Y	N	N	N	N	tandem_dup	na	NA	intergenic	IGKV1OR-3,FOXD4L4	-,-	IG_V_pseu	8568;22965	intergenic	IGKV1OR-2,AL359955.1	-,-	IG_V_pseu	7850;35957
2343	chr9	88668613	+	chr9	88668222	+	N	Y	N	N	N	Y	tandem_dup	na	NA	intronic	GOLM1	-	protein_cod	0	intronic	GOLM1	-	protein_cod	0
2344	chr9	91589338	+	chr9	91588657	+	N	Y	N	N	Y	N	tandem_dup	na	NA	ncRNA_intr	RP11-791O21.5	+	lincRNA	0	ncRNA_intr	RP11-791O21.5	+	lincRNA	0
2345	chr9	108478902	+	chr9	108476251	+	N	N	N	Y	N	N	tandem_dup	na	NA	intronic	TMEM38B	+	protein_cod	0	intronic	TMEM38B	+	protein_cod	0
2346	chr9	112147936	+	chr9	111523125	+	N	Y	Y	Y	Y	Y	tandem_dup	na	NA	intronic	PTPN3	-	protein_cod	0	intergenic	RP11-339N8.1,RPL36AP	-,+	lincRNA,ps	57753;60852

2347	chrX	30819814	+	chrX	30813677	+	N	N	N	Y	N	N	tandem_dup	na	NA	intergenic	GK,TAB3	+,-	protein_cod	71089;25745	intergenic	GK,TAB3	+,-	protein_cod	64952;31882
2348	chrX	55678954	+	chrX	52886725	+	Y	Y	Y	N	Y	Y	tandem_dup	na	NA	intergenic;in	RP11-167P23.4,RP11-	-,-	pseudogene	16865;2188	intergenic;in	EIF4A2P4,XAGE3	-,-	pseudogene	23776;4832
2349	chrX	77745119	+	chrX	68971070	+	N	N	N	N	N	Y	tandem_dup	na	NA	intergenic	CYSLTR1,HMGN1P34	-,-	protein_cod	162139;29971	intronic	EDA	+	protein_cod	0
2350	chrX	84129628	+	chrX	65919838	+	N	N	Y	N	N	N	tandem_dup	na	NA	intergenic	SETP4,UBE2DNL	+,+	pseudogene	118617;59529	intergenic	RNU6-394P,RP6-22P16.	-,+	snRNA,pseu	23632;673577
2351	chrY	6403694	+	chrY	6402153	+	N	Y	N	N	N	N	tandem_dup	na	NA	intergenic	TSPY17P,SRIP3	+,-	pseudogene	14724;183309	intergenic	TSPY17P,SRIP3	+,-	pseudogene	13183;184850
2352	chrY	13470467	+	chrY	13461984	+	N	Y	N	N	N	N	tandem_dup	na	NA	upstream	DUX4L17	+	pseudogene	0	upstream	DUX4L16	+	pseudogene	0
2353	chrY	13870110	+	chrY	13846941	+	N	Y	N	N	Y	N	tandem_dup	na	NA	intergenic	RP11-295P22.2,RCC2	-,-	pseudogene	240197;31648	intergenic	RP11-295P22.2,RCC2P1	-,-	pseudogene	217028;54817
2354	chrY	28801542	+	chrY	28798546	+	N	N	N	N	N	Y	tandem_dup	na	NA	intergenic	PARP4P1,NONE	-,NONE	pseudogene	20743;NONE	intergenic	PARP4P1,NONE	-,NONE	pseudogene	17747;NONE
2355	chr1	115585748	-	chr10	54589551	-	N	Y	N	N	N	N	transl_inter	na	NA	intergenic;in	TSHB,TSPAN2	+,-	protein_cod	8807;4884	intergenic;in	MBL2,Y_RNA	-,+	protein_cod	58091;22816
2356	chr1	147391924	-	chr10	69636695	-	Y	Y	Y	Y	Y	Y	transl_inter	na	NA	intergenic;in	GJA8,GPR89B	+,+	protein_cod	10531;8582	intergenic;in	RPL12P8,SIRT1	+,+	pseudogene	2023;7732
2357	chr1	181868941	-	chr10	32421225	-	N	N	Y	N	N	N	transl_inter	na	NA	intergenic	RN7SKP229,ZNF648	-,-	misc_RNA,	60032;154764	intergenic	RP11-241I20.5,RNU7-22	-,+	pseudogene	20508;41387
2358	chr1	235798830	-	chr10	33437068	-	N	N	N	N	N	Y	transl_inter	na	NA	intronic	GNG4	-	protein_cod	0	intergenic	RP11-342D11.3,NRP1	-,-	lincRNA,pro	31468;29352
2359	chr1	4393640	-	chr10	9934575	+	N	N	Y	N	N	N	transl_inter	na	NA	intergenic	RP11-374C13.1,RP5-1	-,+	pseudogene	157681;78471	intergenic	RP5-1051H14.2,RP5-93	-,+	lincRNA,linc	133375;166110
2360	chr1	66910812	-	chr10	5378076	+	Y	N	N	N	N	N	transl_inter	na	NA	intergenic	PDE4B,SGIP1	+,+	protein_cod	70553;88254	intergenic	RP11-445P17.5,UCN3	-,+	pseudogene	31248;28896
2361	chr1	66910901	-	chr10	5377785	+	N	N	N	N	N	Y	transl_inter	na	NA	intergenic	PDE4B,SGIP1	+,+	protein_cod	70642;88165	intergenic	RP11-445P17.5,UCN3	-,+	pseudogene	30957;29187
2362	chr1	66911227	-	chr10	5377717	+	N	N	N	Y	N	N	transl_inter	na	NA	intergenic	PDE4B,SGIP1	+,+	protein_cod	70968;87839	intergenic	RP11-445P17.5,UCN3	-,+	pseudogene	30889;29255
2363	chr1	212471932	-	chr10	32259244	+	Y	N	N	N	N	N	transl_inter	na	NA	ncRNA_intr	RP11-384C4.6	+	sense_intro	0	intergenic	ARHGAP12,RP11-241I2	-,+	protein_cod	41502;13569
2364	chr1	230920653	-	chr10	134432850	+	N	Y	N	N	N	N	transl_inter	na	NA	ncRNA_intr	RP11-99J16__A.2	-	antisense	0	intronic	INPP5A	+	protein_cod	0
2365	chr1	48402840	+	chr10	133968849	-	N	N	Y	N	N	N	transl_inter	na	NA	intronic	TRABD2B	-	protein_cod	0	intronic	JAKMIP3	+	protein_cod	0
2366	chr1	109067045	+	chr10	64130705	-	Y	N	N	N	N	N	transl_inter	na	NA	intergenic	RP5-964H19.2,FAM10	+,+	pseudogene	15180;35666	intergenic	RN7SL591P,ZNF365	+,+	misc_RNA,	20657;3246
2367	chr1	115585748	+	chr10	54589551	+	N	N	N	N	Y	N	transl_inter	na	NA	intergenic;in	TSHB,TSPAN2	+,-	protein_cod	8807;4884	intergenic;in	MBL2,Y_RNA	-,+	protein_cod	58091;22816
2368	chr1	147391924	+	chr10	69636695	+	N	N	N	Y	N	N	transl_inter	na	NA	intergenic;in	GJA8,GPR89B	+,+	protein_cod	10531;8582	intergenic;in	RPL12P8,SIRT1	+,+	pseudogene	2023;7732
2369	chr1	224200802	+	chr10	38776986	+	N	N	N	Y	N	N	transl_inter	na	NA	intergenic	RP11-504P24.6,AC13	+,-	lincRNA,pro	2488;14958	intergenic	RP11-291L22.4,ABCD1P	+,+	lincRNA,pse	21882;117563
2370	chr1	231007841	-	chr11	57713902	-	N	N	N	N	Y	N	transl_inter	na	NA	intergenic	C1orf198,RP5-858B6.	-,+	protein_cod	2506;2751	upstream	OR5BD1P	-	pseudogene	0
2371	chr1	226415473	-	chr11	59283672	+	N	N	Y	N	N	Y	transl_inter	na	NA	downstream	MIXL1	+	protein_cod	0	downstream	OR4D9	+	protein_cod	0
2372	chr1	247685315	-	chr11	68242933	+	N	N	N	Y	N	N	transl_inter	na	NA	intronic	GCSAML	+	protein_cod	0	intronic	PPP6R3	+	protein_cod	0
2373	chr1	3754993	+	chr11	119124249	-	N	Y	Y	Y	Y	Y	transl_inter	na	NA	intronic;intr	CEP104	-	protein_cod	0	intronic;intr	CBL	+	protein_cod	0
2374	chr1	226415473	+	chr11	59283672	-	N	Y	N	N	N	Y	transl_inter	na	NA	downstream	MIXL1	+	protein_cod	0	downstream	OR4D9	+	protein_cod	0
2375	chr1	17481865	-	chr12	30403690	+	N	N	Y	N	N	N	transl_inter	na	NA	intergenic	PADI2,Y_RNA	-,+	protein_cod	35917;2827	ncRNA_intr	RP11-776A13.3	-	lincRNA	0
2376	chr1	43174852	-	chr12	31737773	+	N	N	Y	N	N	N	transl_inter	na	NA	intergenic	YBX1,CLDN19	+,-	protein_cod	6832;23912	intronic	DENND5B	-	protein_cod	0
2377	chr1	46219432	+	chr12	11236584	-	N	N	N	N	N	Y	transl_inter	na	NA	intergenic	IPP,RP11-630I5.1	-,+	protein_cod	3110;27517	intergenic	TAS2R64P,TAS2R43	-,-	pseudogene	4814;7302
2378	chr1	219161615	+	chr12	90617710	-	N	Y	N	N	N	N	transl_inter	na	NA	intergenic	RP11-412H9.2,RP11-1	-,-	lincRNA,ant	72089;98329	intergenic	RP11-567C2.1,RP11-753	+,+	lincRNA,linc	111154;56961
2379	chr1	6997960	+	chr12	133319419	+	N	N	N	N	N	Y	transl_inter	na	NA	intronic	CAMTA1	+	protein_cod	0	intronic	ANKLE2	-	protein_cod	0
2380	chr1	247568931	+	chr12	107305911	+	Y	N	N	N	N	N	transl_inter	na	NA	intergenic	ZNF496,NLRP3	-,+	protein_cod	73886;10527	intergenic	RPL30P12,RP11-412D9.	+,-	pseudogene	1024;41896
2381	chr1	191666306	-	chr13	114639954	-	N	N	N	N	N	Y	transl_inter	na	NA	intergenic	RP11-309H21.2,RP11-	-,-	lincRNA,linc	468709;161531	intergenic	LINC00565,RASA3	-,-	lincRNA,pro	8137;107240
2382	chr1	102114765	+	chr13	64826021	+	N	N	N	N	N	Y	transl_inter	na	NA	intergenic;in	RP11-157N3.1,RP11-4	+,+	lincRNA,linc	258906;74533	intergenic;in	LINC00355,LGMNP1	-,-	lincRNA,pse	175877;706208
2383	chr1	102538958	-	chr14	20294676	-	N	Y	N	N	Y	Y	transl_inter	na	NA	intergenic	OLFM3,RP11-202K23.	-,-	protein_cod	76372;126337	intronic	OR4N2	+	protein_cod	0
2384	chr1	158998559	-	chr14	97660985	-	N	N	N	Y	N	N	transl_inter	na	NA	intronic	IFI16	+	protein_cod	0	intergenic	RP11-359N5.1,CTD-250	+,+	lincRNA,linc	35912;264168
2385	chr1	188457229	-	chr14	71558408	-	N	N	N	Y	N	N	transl_inter	na	NA	intergenic	RP11-405P11.1,RP11-	-,+	pseudogene	245420;20440	intronic	PCNX	+	protein_cod	0
2386	chr1	213083588	-	chr14	99451394	-	N	N	N	N	N	Y	transl_inter	na	NA	intergenic	FLVCR1,VASH2	+,+	protein_cod	10883;40274	intergenic	AL162151.3,AL162151.4	+,+	pseudogene	11586;173359
2387	chr1	213083594	-	chr14	99451818	-	N	Y	N	N	N	N	transl_inter	na	NA	intergenic	FLVCR1,VASH2	+,+	protein_cod	10889;40268	intergenic	AL162151.3,AL162151.4	+,+	pseudogene	12010;172935
2388	chr1	213714502	-	chr14	34497104	-	Y	N	N	N	N	N	transl_inter	na	NA	ncRNA_intr	RP11-554K11.2	+	lincRNA	0	intronic	EGLN3	-	protein_cod	0
2389	chr1	213714502	-	chr14	34497153	-	N	N	N	N	N	Y	transl_inter	na	NA	ncRNA_intr	RP11-554K11.2	+	lincRNA	0	intronic	EGLN3	-	protein_cod	0
2390	chr1	202609729	-	chr14	99887273	+	N	N	N	N	Y	N	transl_inter	na	NA	intronic	SYT2	-	protein_cod	0	intronic	SETD3	-	protein_cod	0
2391	chr1	13984901	+	chr14	31906434	-	N	N	N	Y	N	N	transl_inter	na	NA	ncRNA_intr	CTA-520D8.2	-	lincRNA	0	ncRNA_intr	CTD-2213F21.2	+	antisense	0
2392	chr1	22771487	+	chr14	32719223	-	N	N	N	N	Y	N	transl_inter	na	NA	intergenic	RP11-415K20.1,ZBTB	-,+	pseudogene	121663;6857	intergenic	RP11-134E15.1,RP11-13	-,-	pseudogene	6924;21838
2393	chr1	23952531	+	chr14	104698426	-	Y	N	N	N	N	N	transl_inter	na	NA	intergenic	ID3,MDS2	-,+	protein_cod	66246;1293	ncRNA_intr	RP11-260M19.2	+	lincRNA	0
2394	chr1	162231708	+	chr14	77736037	-	N	N	N	N	Y	N	transl_inter	na	NA	intronic	NOS1AP	+	protein_cod	0	intronic	NGB	-	protein_cod	0
2395	chr1	20708973	+	chr14	79723828	+	N	Y	N	N	N	N	transl_inter	na	NA	ncRNA_intr	LINC01141	-	lincRNA	0	intronic	NRXN3	+	protein_cod	0
2396	chr1	156109929	+	chr14	66078023	+	Y	Y	N	Y	Y	Y	transl_inter	na	NA	downstream	LMNA	+	protein_cod	0	intronic	FUT8	+	protein_cod	0
2397	chr1	156705347	+	chr14	61033234	+	Y	Y	Y	Y	Y	Y	transl_inter	na	NA	intronic	RRNAD1	+	protein_cod	0	intergenic	RP11-1042B17.3,RP11-1	+,-	antisense,p	11600;5508
2398	chr1	45678065	-	chr15	67156987	-	N	N	N	Y	Y	N	transl_inter	na	NA	intergenic	ZSWIM5,AL592294.1	-,-	protein_cod	5837;19825	intergenic	RP11-622C24.1,RP11-79	+,+	pseudogene	54089;55162
2399	chr1	45678091	-	chr15	67156988	-	Y	N	N	N	N	N	transl_inter	na	NA	intergenic;in	ZSWIM5,AL592294.1	-,-	protein_cod	5863;19799	intergenic;in	RP11-622C24.1,RP11-79	+,+	pseudogene	54090;55161
2400	chr1	17230525	+	chr15	75867613	-	N	Y	N	N	N	N	transl_inter	na	NA	downstream	RP11-108M9.6	-	lincRNA	0	ncRNA_intr	CTD-2323K18.1	-	processed_	0
2401	chr1	45678091	+	chr15	67156988	+	N	N	N	Y	Y	Y	transl_inter	na	NA	intergenic;in	ZSWIM5,AL592294.1	-,-	protein_cod	5863;19799	intergenic;in	RP11-622C24.1,RP11-79	+,+	pseudogene	54090;55161
2402	chr1	119401194	+	chr15	51189243	+	Y	Y	N	N	Y	Y	transl_inter	na	NA	intergenic	RP4-630J13.1,TBX15	+,-	pseudogene	144893;24475	intergenic	RP11-507J18.1,AP4E1	+,+	pseudogene	98095;11626
2403	chr1	149216152	+	chr15	75867352	+	N	Y	Y	N	Y	N	transl_inter	na	NA	upstream	RNU1-92P	-	snRNA	0	ncRNA_intr	CTD-2323K18.1	-	processed_	0
2404	chr1	142536514	-	chr16	33866158	-	N	N	N	N	N	Y	transl_inter	na	NA	intergenic	NONE,RP11-417J8.1	NONE,+	NONE,lincR	NONE;16779	intergenic	RP11-598D12.2,CTD-25	+,+	pseudogene	12330;56779
2405	chr1	148855714	-	chr16	32490058	-	N	Y	N	N	N	N	transl_inter	na	NA	ncRNA_intr	RP11-763B22.9	+	pseudogene	0	upstream	ABCD1P3	-	pseudogene	0
2406	chr1	149034034	-	chr16	34191797	-	N	Y	N	N	N	N	transl_inter	na	NA	intergenic	RP11-14N7.2,AL7323	+,-	lincRNA,mi	82439;2278	intergenic	RNA5-8SP2,CTD-2144E	+,-	rRNA,lincR	226220;21318
2407	chr1	121137800	-	chr16	32822737	+	N	N	N	Y	N	N	transl_inter	na	NA	ncRNA_intr	RP11-343N15.1	-	lincRNA	0	ncRNA_intr	RP11-67H24.2	+	lincRNA	0
2408	chr1	121137862	-	chr16	32823025	+	N	Y	N	N	N	N	transl_inter	na	NA	ncRNA_intr	RP11-343N15.1	-	lincRNA	0	ncRNA_intr	RP11-67H24.2	+	lincRNA	0
2409	chr1	98978492	+	chr16	28388864	-	N	N	N	N	N	Y	transl_inter	na	NA	intergenic	AL160056.1,SNX7	+,+	miRNA,prot	139437;148744	intergenic	RP11-435I10.5,EIF3CL	+,-	pseudogene	12210;2036
2410	chr1	148855765	+	chr16	32127477	-	N	N	N	Y	N	N	transl_inter	na	NA	ncRNA_intr	RP11-763B22.9	+	pseudogene	0	ncRNA_intr	HERC2P4	-	pseudogene	0
2411	chr1	207449833	+	chr16	81084769	-	Y	N	N	N	N	N	transl_inter	na	NA	ncRNA_intr	RP11-6J21.2	+	lincRNA	0	intergenic	ATMIN,C16orf46	+,-	protein_cod	3806;2333
2412	chr1	142536080	+	chr16	33866600	+	N	N	Y	N	N	N	transl_inter	na	NA	intergenic	NONE,RP11-417J8.1	NONE,+	NONE,lincR	NONE;17213	intergenic	RP11-598D12.2,CTD-25	+,+	pseudogene	12772;56337
2413	chr1	149029763	+	chr16	32117552	+	N	N	Y	Y	N	N	transl_inter	na	NA	intergenic	RP11-14N7.2,AL7323	+,-	lincRNA,mi	78168;6549	ncRNA_intr	HERC2P4	-	pseudogene	0
2414	chr1	177642022	+	chr16	33970988	+	Y	Y	N	N	N	Y	transl_inter	na	NA	intergenic	RP1-35C21.2,RP11-63	-,+	lincRNA,linc	75178;27637	intergenic	RNA5-8SP2,CTD-2144E	+,-	rRNA,lincR	5411;242127
2415	chr1	246048065	+	chr16	21301415	+	N	N	Y	N	Y	N	transl_inter	na	NA	intronic	SMYD3	-	protein_cod	0	intronic	CRYM	-	protein_cod	0
2416	chr1	246048340	+	chr16	21301439	+	N	N	N	N	N	Y	transl_inter	na	NA	intronic	SMYD3	-	protein_cod	0	intronic	CRYM	-	protein_cod	0
2417	chr1	209891523	-	chr17	28483266	+	Y	N	N	Y	N	N	transl_inter	na	NA	ncRNA_intr	RP1-28O10.1	-	antisense	0	intronic	NSRP1	+	protein_cod	0
2418	chr1	43547030	+	chr17	38296670	-	N	N	Y	Y	N	Y	transl_inter	na	NA	intergenic;in	RNU6-870P,FAM183A	-,+	snRNA,prot	57722;63794	UTR5;UTR	CASC3	+	protein_cod	0
2419	chr1	53891069	+	chr17	74606676	-	Y	N	N	N	N	Y	transl_inter	na	NA	ncRNA_intr	SLC25A3P1	-	pseudogene	0	intergenic	RP11-666A8.11,ST6GAL	-,-	pseudogene	14822;14167
2420	chr1	179651079	+	chr17	41589752	-	N	N	N	Y	N	N	transl_inter	na	NA	intronic;intr	TDRD5	+	protein_cod	0	intronic	DHX8	+	protein_cod	0
2421	chr1	168177012	-	chr18	48643574	-	N	N	N	N	N	Y	transl_inter	na	NA	intergenic	TIPRL,RPL34P1	+,+	protein_cod	7062;2842	intergenic	RN7SL695P,SRSF10P1	-,-	misc_RNA,	10158;41078
2422	chr1	166384193	-	chr18	6166483	+	N	N	N	N	N	Y	transl_inter	na	NA	ncRNA_intr	RP11-479J7.1	-	lincRNA	0	intronic	L3MBTL4	-	protein_cod	0
2423	chr1	213714501	-	chr18	56359738	+	N	Y	N	N	Y	N	transl_inter	na	NA	ncRNA_intr	RP11-554K11.2	+	lincRNA	0	ncRNA_intr	RP11-126O1.4	-	antisense	0
2424	chr1	166384036	+	chr18	6166335	-	N	N	Y	N	N	N	transl_inter	na	NA	ncRNA_intr	RP11-479J7.1	-	lincRNA	0	intronic	L3MBTL4	-	protein_cod	0
2425	chr1	166384544	+	chr18	6166164	-	Y	N	N	N	N	N	transl_inter	na	NA	ncRNA_intr	RP11-479J7.1	-	lincRNA	0	intronic	L3MBTL4	-	protein_cod	0
2426	chr1	38241383	-	chr19	11422016	-	Y	N	Y	Y	Y	Y	transl_inter	na	NA	upstream	ACTN4P2	+	pseudogene	0	ncRNA_intr	CTC-510F12.4	-	3prime_ove	0
2427	chr1	161251641	-	chr19	57994529	+	N	Y	N	N	N	N	transl_inter	na	NA	intronic	PCP4L1	+	protein_cod	0	downstream	AC003005.2	+	lincRNA	0
2428	chr1	161251687	-	chr19	57994530	+	N	N	Y	N	N	N	transl_inter	na	NA	intronic	PCP4L1	+	protein_cod	0	downstream	AC003005.2	+	lincRNA	0
2429	chr1	162234527	-	chr19	57599179	+	N	N	N	N	N	Y	transl_inter	na	NA	intronic	NOS1AP	+	protein_cod	0	intergenic	RPL7AP69,USP29	+,+	pseudogene	21672;31327
2430	chr1	185025679	-	chr19	45828385	+	Y	N	Y	N	Y	Y	transl_inter	na	NA	intronic;intr	RNF2	+	protein_cod	0	intergenic;in	CKM,RPS16P9	-,-	protein_cod	2150;7571

2431	chr1	226415473	-	chr19	44776096	+	Y	N	Y	N	Y	N	transl_inter	na	NA	downstream	MIXL1	+	protein_cod	0	intronic;intrc	ZNF233,ZNF235	+,-	protein_cod	0
2432	chr1	28515672	+	chr19	24027934	-	Y	Y	Y	Y	Y	Y	transl_inter	na	NA	intronic	PTAFR	-	protein_cod	0	ncRNA_intr	RP11-255H23.4	-	lincRNA	0
2433	chr1	121138647	+	chr19	33444599	-	Y	Y	N	N	Y	Y	transl_inter	na	NA	ncRNA_exo	AL592494.5	+	lincRNA	0	exonic;exon	CEP89	-	protein_cod	0
2434	chr1	162234674	+	chr19	57599175	-	N	N	Y	N	N	N	transl_inter	na	NA	intronic	NOS1AP	+	protein_cod	0	intergenic	RPL7AP69,USP29	+,+	pseudogene	21668;31331
2435	chr1	210891699	+	chr19	45732603	-	N	N	N	N	Y	Y	transl_inter	na	NA	intronic	KCNH1	-	protein_cod	0	intronic	MARK4,EXOC3L2	+,-	protein_cod	0
2436	chr1	226415473	+	chr19	44776096	-	N	N	N	N	N	Y	transl_inter	na	NA	downstream	MIXL1	+	protein_cod	0	intronic;intrc	ZNF233,ZNF235	+,-	protein_cod	0
2437	chr1	155664255	+	chr19	7861196	+	N	N	N	Y	N	N	transl_inter	na	NA	ncRNA_intr	MSTO2P	+	pseudogene	0	intergenic	CLEC4GP1,EXOSC3P2	+,-	pseudogene	5689;17305
2438	chr1	179269020	+	chr19	36377271	+	N	N	N	N	Y	N	transl_inter	na	NA	intronic	SOAT1	+	protein_cod	0	intergenic	RN7SL402P,NFKBID	+,-	misc_RNA,p	4218;1284
2439	chr1	198366573	+	chr19	13366213	+	Y	N	N	N	N	N	transl_inter	na	NA	intergenic	NEK7,ATP6V1G3	+,-	protein_cod	75023;125779	intronic	CACNA1A	-	protein_cod	0
2440	chr1	207521646	+	chr19	15977203	+	N	Y	Y	N	N	N	transl_inter	na	NA	intronic	CD55	+	protein_cod	0	intergenic	AC004791.2,CYP4F36P	-,-	processed_	1489;5274
2441	chr1	37938374	-	chr2	68914885	-	N	N	N	N	Y	N	transl_inter	na	NA	ncRNA_intr	LINC01137	-	antisense	0	intronic;intrc	ARHGAP25	+	protein_cod	0
2442	chr1	164240436	-	chr2	190999445	-	Y	N	N	N	N	N	transl_inter	na	NA	intergenic;ir	U3,NMNAT1P2	+,+	snoRNA,pse	347327;71806	intronic;intrc	C2orf88	+	protein_cod	0
2443	chr1	164240562	-	chr2	190999443	-	N	Y	Y	Y	Y	Y	transl_inter	na	NA	intergenic;ir	U3,NMNAT1P2	+,+	snoRNA,pse	347453;71680	intronic;intrc	C2orf88	+	protein_cod	0
2444	chr1	27129922	-	chr2	65566030	+	N	N	N	N	Y	N	transl_inter	na	NA	intergenic	PIGV,RN7SL165P	+,+	protein_cod	5033;11391	intronic	SPRED2	-	protein_cod	0
2445	chr1	27129923	-	chr2	65566105	+	N	N	N	N	N	Y	transl_inter	na	NA	intergenic	PIGV,RN7SL165P	+,+	protein_cod	5034;11390	intronic	SPRED2	-	protein_cod	0
2446	chr1	27129924	-	chr2	65566028	+	N	N	Y	Y	N	N	transl_inter	na	NA	intergenic	PIGV,RN7SL165P	+,+	protein_cod	5035;11389	intronic	SPRED2	-	protein_cod	0
2447	chr1	27129933	-	chr2	65566095	+	Y	N	N	N	N	N	transl_inter	na	NA	intergenic	PIGV,RN7SL165P	+,+	protein_cod	5044;11380	intronic	SPRED2	-	protein_cod	0
2448	chr1	103913905	-	chr2	112733750	+	N	Y	N	N	N	N	transl_inter	na	NA	intergenic	RP11-347K2.2,RP11-1	-,+	lincRNA,linc	77919;43596	intronic	MERTK	+	protein_cod	0
2449	chr1	119401193	-	chr2	141852922	+	Y	N	N	N	N	N	transl_inter	na	NA	intergenic;ir	RP4-630J13.1,TBX15	+,-	pseudogene	144892;24476	intronic;intrc	LRP1B	-	protein_cod	0
2450	chr1	200067550	-	chr2	23698252	+	N	N	N	Y	N	N	transl_inter	na	NA	intronic;intrc	NR5A2	+	protein_cod	0	intronic;intrc	KLHL29	+	protein_cod	0
2451	chr1	200067676	-	chr2	23698251	+	N	N	Y	N	N	N	transl_inter	na	NA	intronic;intrc	NR5A2	+	protein_cod	0	intronic;intrc	KLHL29	+	protein_cod	0
2452	chr1	11365951	+	chr2	25857316	-	N	N	Y	N	N	N	transl_inter	na	NA	intergenic	UBIAD1,RP11-149P14	+,-	protein_cod	9845;119363	intronic	DTNB	-	protein_cod	0
2453	chr1	108565817	+	chr2	13475219	-	N	Y	N	N	N	N	transl_inter	na	NA	intergenic	AL390036.1,RP11-356	-,+	miRNA,lincF	4424;48287	intergenic	AC064875.2,AC092635.1	-,+	processed_	328081;202579
2454	chr1	108566723	+	chr2	13474178	-	N	N	N	N	N	Y	transl_inter	na	NA	intergenic	AL390036.1,RP11-356	-,+	miRNA,lincF	5330;47381	intergenic	AC064875.2,AC092635.1	-,+	processed_	327040;203620
2455	chr1	119401193	+	chr2	141852922	-	Y	Y	Y	Y	Y	Y	transl_inter	na	NA	intergenic;ir	RP4-630J13.1,TBX15	+,-	pseudogene	144892;24476	intronic;intrc	LRP1B	-	protein_cod	0
2456	chr1	200067550	+	chr2	23698252	-	N	N	Y	N	N	N	transl_inter	na	NA	intronic;intrc	NR5A2	+	protein_cod	0	intronic;intrc	KLHL29	+	protein_cod	0
2457	chr1	200067676	+	chr2	23698251	-	N	N	N	Y	N	N	transl_inter	na	NA	intronic;intrc	NR5A2	+	protein_cod	0	intronic;intrc	KLHL29	+	protein_cod	0
2458	chr1	10379	+	chr2	243152434	+	N	N	Y	N	N	N	transl_inter	na	NA	intergenic	NONE,DDX11L1	NONE,+	NONE,pseu	NONE;1490	intergenic	AC093642.5,ABC7-4304	+,-	pseudogene	50130;7938
2459	chr1	37938257	+	chr2	68914879	+	N	N	N	N	Y	N	transl_inter	na	NA	ncRNA_intr	LINC01137	-	antisense	0	intronic	ARHGAP25	+	protein_cod	0
2460	chr1	37938374	+	chr2	68914885	+	N	Y	N	N	N	N	transl_inter	na	NA	ncRNA_intr	LINC01137	-	antisense	0	intronic;intrc	ARHGAP25	+	protein_cod	0
2461	chr1	142965172	+	chr2	11929915	+	Y	Y	Y	N	Y	Y	transl_inter	na	NA	intergenic	RP11-423O2.7,RP11-7	-,-	lincRNA,linc	8456;153532	intronic	LPIN1	+	protein_cod	0
2462	chr1	164240436	+	chr2	190999445	+	N	Y	Y	Y	Y	Y	transl_inter	na	NA	intergenic;ir	U3,NMNAT1P2	+,+	snoRNA,pse	347327;71806	intronic;intrc	C2orf88	+	protein_cod	0
2463	chr1	164240562	+	chr2	190999443	+	Y	N	N	N	N	N	transl_inter	na	NA	intergenic;ir	U3,NMNAT1P2	+,+	snoRNA,pse	347453;71680	intronic;intrc	C2orf88	+	protein_cod	0
2464	chr1	7489684	-	chr20	59912358	-	N	N	Y	N	Y	N	transl_inter	na	NA	intronic;intrc	CAMTA1	+	protein_cod	0	intronic;intrc	CDH4	+	protein_cod	0
2465	chr1	75536317	-	chr20	60562962	-	N	N	N	N	N	Y	transl_inter	na	NA	intergenic	RP11-93N20.1,RP11-5	+,-	lincRNA,linc	105515;51886	intronic	TAF4	-	protein_cod	0
2466	chr1	35007077	-	chr20	4515777	+	N	Y	N	N	N	N	transl_inter	na	NA	intergenic;ir	RP4-657M3.2,MIR552	-,-	pseudogene	264184;128123	intergenic	RP5-1164C1.2,RPL7AL2	+,-	lincRNA,pse	9931;55862
2467	chr1	63860601	+	chr20	35592731	-	N	N	N	Y	N	N	transl_inter	na	NA	intronic	ALG6	+	protein_cod	0	intergenic	SAMHD1,RBL1	-,-	protein_cod	12485;32021
2468	chr1	7489684	+	chr20	59912358	+	Y	N	N	Y	N	N	transl_inter	na	NA	intronic;intrc	CAMTA1	+	protein_cod	0	intronic;intrc	CDH4	+	protein_cod	0
2469	chr1	142540731	-	chr21	10779260	-	Y	N	N	N	N	N	transl_inter	na	NA	intergenic	NONE,RP11-417J8.1	NONE,+	NONE,lincR	NONE;12562	intergenic	bP-21201H5.1,IGHV1OR	-,+	pseudogene	303199;83362
2470	chr1	143130550	-	chr21	9660838	-	Y	Y	Y	Y	Y	Y	transl_inter	na	NA	ncRNA_intr	RP11-782C8.1,RP11-7	+,-	lincRNA,linc	0	intergenic	NONE,CR381670.1	NONE,+	NONE,miRN	NONE;22353
2471	chr1	142536303	+	chr21	10085991	+	N	N	N	N	Y	N	transl_inter	na	NA	intergenic	NONE,RP11-417J8.1	NONE,+	NONE,lincR	NONE;16990	intergenic	TEKT4P2,CR381653.1	-,+	pseudogene	117406;113952
2472	chr1	142541099	+	chr21	10087091	+	N	N	N	Y	N	N	transl_inter	na	NA	intergenic	NONE,RP11-417J8.1	NONE,+	NONE,lincR	NONE;12194	intergenic	TEKT4P2,CR381653.1	-,+	pseudogene	118506;112852
2473	chr1	121137798	-	chr22	16867924	-	N	N	N	N	N	Y	transl_inter	na	NA	ncRNA_intr	RP11-343N15.1	-	lincRNA	0	upstream	ABCD1P4	+	pseudogene	0
2474	chr1	221827804	-	chr22	42997398	-	N	N	N	Y	Y	Y	transl_inter	na	NA	intergenic	RP11-103C3.2,DUSP1	-,-	pseudogene	103819;46962	intronic	POLDIP3	-	protein_cod	0
2475	chr1	169962744	-	chr22	41212898	+	Y	N	N	N	Y	Y	transl_inter	na	NA	intronic	KIFAP3	-	protein_cod	0	intronic	SLC25A17	-	protein_cod	0
2476	chr1	247869898	-	chr22	29065701	+	N	Y	N	N	N	N	transl_inter	na	NA	ncRNA_intr	RP11-634B7.4	+	antisense	0	intronic	TTC28	-	protein_cod	0
2477	chr1	5285367	-	chr3	59514853	-	N	N	N	Y	N	N	transl_inter	na	NA	intergenic	RP11-542C10.1,RP1-5	-,+	lincRNA,linc	134408;267671	intergenic	RP11-719N22.2,RP11-71	-,+	lincRNA,pse	18449;6736
2478	chr1	20371434	-	chr3	87619831	+	N	N	N	N	Y	Y	transl_inter	na	NA	intergenic	PLA2G2A,PLA2G5	-,+	protein_cod	64502;25267	intergenic	KRT8P25,APOOP2	+,+	pseudogene	245958;24157
2479	chr1	20371538	-	chr3	87619945	+	N	N	Y	N	N	N	transl_inter	na	NA	intergenic	PLA2G2A,PLA2G5	-,+	protein_cod	64606;25163	intergenic	KRT8P25,APOOP2	+,+	pseudogene	246072;24043
2480	chr1	55809978	-	chr3	144790737	+	N	N	Y	N	N	N	transl_inter	na	NA	intergenic	RP11-101C11.1,GOT2	+,+	lincRNA,pse	110128;23161	intergenic	RNA5SP144,RP11-622L2	-,+	rRNA,pseud	884875;450588
2481	chr1	63705366	-	chr3	55788567	+	Y	N	N	N	N	N	transl_inter	na	NA	ncRNA_intr	LINC00466	-	lincRNA	0	intronic	ERC2	-	protein_cod	0
2482	chr1	184183167	-	chr3	126775070	+	N	N	N	Y	N	Y	transl_inter	na	NA	intergenic	Y_RNA,Y_RNA	-,+	misc_RNA,r	42210;101625	intergenic	PLXNA1,C3orf56	+,+	protein_cod	18835;136904
2483	chr1	51664902	+	chr3	127876008	-	N	N	N	N	Y	N	transl_inter	na	NA	ncRNA_intr	RP11-296A18.3	-	lincRNA	0	intronic	EEFSEC	+	protein_cod	0
2484	chr1	98991338	+	chr3	1629499	-	N	Y	N	N	N	N	transl_inter	na	NA	intergenic	AL160056.1,SNX7	+,+	miRNA,prot	152283;135898	intergenic	CNTN6,AC090043.1	+,-	protein_cod	183598;7962
2485	chr1	247133181	+	chr3	113965272	-	N	N	N	N	N	Y	transl_inter	na	NA	intronic	ZNF670,ZNF695	-,-	protein_cod	0	intergenic	ZNF80,TIGIT	-,+	protein_cod	8847;30488
2486	chr1	247685193	+	chr3	56618666	-	N	N	N	N	N	Y	transl_inter	na	NA	intronic	GCSAML	+	protein_cod	0	intronic	CCDC66	+	protein_cod	0
2487	chr1	247685523	+	chr3	56617990	-	N	N	N	N	Y	N	transl_inter	na	NA	intronic	GCSAML	+	protein_cod	0	intronic	CCDC66	+	protein_cod	0
2488	chr1	104480855	+	chr3	113965502	+	Y	Y	Y	N	N	Y	transl_inter	na	NA	intergenic;ir	RP11-97C18.1,RP11-3	+,+	pseudogene	10427;134777	intergenic;ir	ZNF80,TIGIT	-,+	protein_cod	9077;30258
2489	chr1	147392538	+	chr3	48706151	+	Y	Y	N	Y	Y	Y	transl_inter	na	NA	intergenic;ir	GJA8,GPR89B	+,+	protein_cod	11145;7968	ncRNA_intr	RP11-148G20.1	+	antisense	0
2490	chr1	43382320	-	chr4	58165638	-	N	N	N	N	N	Y	transl_inter	na	NA	intergenic	ATP6V1E1P1,SLC2A1	+,-	pseudogene	12737;8732	intergenic	IGFBP7-AS1,RPS26P24	+,-	antisense,ps	93962;53527
2491	chr1	213714501	-	chr4	124927547	-	N	Y	Y	N	Y	N	transl_inter	na	NA	ncRNA_intr	RP11-554K11.2	+	lincRNA	0	intergenic	LINC01091,RP11-728C8	+,+	lincRNA,pse	75986;56070
2492	chr1	28243374	-	chr4	154143306	+	N	N	Y	N	N	N	transl_inter	na	NA	intergenic	RPA2,SMPDL3B	-,+	protein_cod	2117;18130	intronic	TRIM2	+	protein_cod	0
2493	chr1	11031152	+	chr4	15134832	-	N	N	Y	Y	N	N	transl_inter	na	NA	intronic	C1orf127	-	protein_cod	0	ncRNA_intr	RP11-665G4.1	-	antisense	0
2494	chr1	55705907	+	chr4	93827724	-	Y	Y	Y	Y	Y	Y	transl_inter	na	NA	intergenic	RP11-101C11.1,GOT2	+,+	lincRNA,pse	6057;127232	intronic	GRID2	+	protein_cod	0
2495	chr1	175811991	+	chr4	128835353	-	Y	N	N	Y	N	N	transl_inter	na	NA	intergenic	TNR,RP11-222A5.1	-,+	protein_cod	99085;34488	intergenic	PLK4,MFSD8	+,-	protein_cod	15003;3607
2496	chr1	103737726	+	chr4	149966734	+	Y	Y	Y	Y	Y	N	transl_inter	na	NA	intergenic	COL11A1,RP11-347K2	-,+	protein_cod	163674;80043	ncRNA_intr	RP11-563M4.1	-	lincRNA	0
2497	chr1	146480344	+	chr4	142757082	+	N	N	N	Y	N	N	transl_inter	na	NA	intergenic	RP4-704D21.2,NBPF1	-,-	lincRNA,pse	5167;10551	intergenic	IL15,INPP4B	+,-	protein_cod	101942;187231
2498	chr1	146480395	+	chr4	142756991	+	N	N	N	N	N	Y	transl_inter	na	NA	intergenic	RP4-704D21.2,NBPF1	-,-	lincRNA,pse	5218;10500	intergenic	IL15,INPP4B	+,-	protein_cod	101851;187322
2499	chr1	203754141	+	chr4	169074633	+	N	N	Y	N	N	N	transl_inter	na	NA	intergenic	LAX1,ZC3H11A	+,+	protein_cod	8896;10641	intronic	ANXA10	+	protein_cod	0
2500	chr1	203754161	+	chr4	169074583	+	Y	Y	N	Y	N	Y	transl_inter	na	NA	intergenic	LAX1,ZC3H11A	+,+	protein_cod	8916;10621	intronic	ANXA10	+	protein_cod	0
2501	chr1	147392061	-	chr5	10607142	-	Y	Y	N	N	N	N	transl_inter	na	NA	intergenic	GJA8,GPR89B	+,+	protein_cod	10668;8445	intronic	ANKRD33B	+	protein_cod	0
2502	chr1	236602595	-	chr5	32974370	-	Y	Y	Y	Y	Y	Y	transl_inter	na	NA	intronic	EDARADD	+	protein_cod	0	ncRNA_intr	CTD-2066L21.3	-	lincRNA	0
2503	chr1	121214967	-	chr5	49771194	+	N	N	N	N	Y	N	transl_inter	na	NA	intergenic;ir	AL592494.5,RP11-344	+,-	lincRNA,linc	9984;21210	intergenic;ir	EMB,RP11-269M20.2	-,+	protein_cod	33993;127869
2504	chr1	121215656	-	chr5	49770855	+	N	Y	N	N	N	N	transl_inter	na	NA	intergenic	AL592494.5,RP11-344	+,-	lincRNA,linc	10673;20521	intergenic	EMB,RP11-269M20.2	-,+	protein_cod	33654;128208
2505	chr1	150593722	-	chr5	55447995	+	N	N	Y	Y	N	N	transl_inter	na	NA	intronic	ENSA	-	protein_cod	0	intronic	ANKRD55	-	protein_cod	0
2506	chr1	194964452	-	chr5	24150525	+	N	N	Y	Y	N	N	transl_inter	na	NA	intergenic;ir	RP11-489C13.1,AL357	+,+	pseudogene	276086;131359	intronic;intrc	C5orf17	+	protein_cod	0
2507	chr1	59649558	+	chr5	74829048	-	N	N	N	N	N	Y	transl_inter	na	NA	ncRNA_intr	RP11-470E16.1	-	processed_	0	intronic	POLK	+	protein_cod	0
2508	chr1	88901174	+	chr5	32698873	-	N	N	N	N	Y	N	transl_inter	na	NA	intergenic	CTA-21C21.1,RN7SL5	+,-	pseudogene	122189;42340	intronic	NPR3	+	protein_cod	0
2509	chr1	194964452	+	chr5	24150525	-	N	N	Y	N	Y	N	transl_inter	na	NA	intergenic;ir	RP11-489C13.1,AL357	+,+	pseudogene	276086;131359	intronic;intrc	C5orf17	+	protein_cod	0
2510	chr1	199440228	+	chr5	33633347	-	Y	Y	Y	Y	Y	Y	transl_inter	na	NA	intergenic	RP11-382E9.1,AL4502	+,-	lincRNA,miF	77793;144088	intronic	ADAMTS12	-	protein_cod	0
2511	chr1	237097197	+	chr5	707022	-	Y	N	N	N	N	N	transl_inter	na	NA	intergenic	MTR,RPL35P1	+,+	protein_cod	29916;47440	intergenic	TPPP,ZDHHC11	-,-	protein_cod	13512;3449
2512	chr1	237097662	+	chr5	706564	-	N	N	Y	N	Y	Y	transl_inter	na	NA	intergenic	MTR,RPL35P1	+,+	protein_cod	30381;46975	intergenic	TPPP,ZDHHC11	-,-	protein_cod	13054;3907
2513	chr1	248118434	+	chr5	85877916	-	N	N	N	Y	N	N	transl_inter	na	NA	intronic	OR2L13	+	protein_cod	0	intergenic	CTD-2299E8.1,COX7C	+,+	pseudogene	179138;35805
2514	chr1	4100823	+	chr5	123549695	+	N	N	Y	N	N	N	transl_inter	na	NA	intergenic	RP13-614K11.1,RP11-	+,-	lincRNA,pse	85501;134765	intergenic	KRT18P16,HMGB1P29	+,-	pseudogene	576598;6577

2515	chr1	199575586	-	chr6	116668158	-	N	Y	N	N	N	N	transl_inter	na	NA	intergenic	RP11-382E9.1,AL4502	+,-	lincRNA,miF	213151;8730	ncRNA_intro	RP11-93H18.1	+	lincRNA	0
2516	chr1	86003004	-	chr6	42375931	+	N	N	Y	N	Y	N	transl_inter	na	NA	intronic	DDAH1	-	protein_cod	0	intronic;intro	TRERF1	-	protein_cod	0
2517	chr1	144842328	-	chr6	146835380	+	Y	N	N	N	N	N	transl_inter	na	NA	intergenic	RP4-791M13.3,PDE4D	-,-	antisense,pi	6461;9096	intergenic	GRM1,RAB32	+,+	protein_cod	76646;29449
2518	chr1	144842511	-	chr6	146835444	+	N	N	N	N	Y	N	transl_inter	na	NA	intergenic	RP4-791M13.3,PDE4D	-,-	antisense,pi	6644;8913	intergenic	GRM1,RAB32	+,+	protein_cod	76710;29285
2519	chr1	85983489	+	chr6	122001289	-	N	N	N	N	Y	Y	transl_inter	na	NA	intronic;intro	DDAH1	-	protein_cod	0	ncRNA_exo	RP11-129H15.3	-	pseudogene	0
2520	chr1	86003022	+	chr6	42375931	-	N	N	N	N	N	Y	transl_inter	na	NA	intronic	DDAH1	-	protein_cod	0	intronic;intro	TRERF1	-	protein_cod	0
2521	chr1	155663073	+	chr6	27161314	-	N	N	N	N	N	Y	transl_inter	na	NA	ncRNA_intro	MSTO2P	+	pseudogene	0	intergenic	MIR3143,RP11-209A2.1	+,+	miRNA,pseu	45847;17709
2522	chr1	166077344	+	chr6	48815047	-	N	Y	N	N	N	N	transl_inter	na	NA	intronic	FAM78B	-	protein_cod	0	intergenic	RBMXP1,AL391538.1	-,+	pseudogene	632517;2513
2523	chr1	207579635	+	chr6	57494233	-	Y	Y	Y	Y	Y	Y	transl_inter	na	NA	intergenic	CD55,CR2	+,+	protein_cod	45324;47940	intronic	PRIM2	+	protein_cod	0
2524	chr1	231935662	+	chr6	54380955	-	Y	N	N	N	Y	N	transl_inter	na	NA	intronic	DISC1,TSNAX-DISC1	+,+	protein_cod	0	intergenic	CLNS1AP1,RP3-334F4.2	-,-	pseudogene	30307;86330
2525	chr1	236350853	+	chr6	116669055	-	N	N	N	N	Y	N	transl_inter	na	NA	intronic	GPR137B	+	protein_cod	0	ncRNA_intro	RP11-93H18.1	+	lincRNA	0
2526	chr1	15829578	-	chr7	148633371	-	N	Y	N	Y	N	N	transl_inter	na	NA	intronic	CASP9	-	protein_cod	0	intergenic	RN7SL569P,RNY5	+,+	misc_RNA,r	45764;5209
2527	chr1	29731512	-	chr7	130372013	-	N	N	Y	N	Y	N	transl_inter	na	NA	intergenic	RP3-437I16.1,AL13915	-,+	lincRNA,miF	54886;27481	UTR5	TSGA13	-	protein_cod	0
2528	chr1	60870955	-	chr7	36252152	-	N	N	N	N	Y	N	transl_inter	na	NA	intergenic	RP11-575B7.3,RP11-7	+,-	lincRNA,linc	255604;134966	intronic	EEPD1	+	protein_cod	0
2529	chr1	93321960	-	chr7	35964865	-	N	N	N	N	N	Y	transl_inter	na	NA	intronic;intro	FAM69A	-	protein_cod	0	intergenic	SEPT7,RNU6-1085P	+,-	protein_cod	19948;4903
2530	chr1	93321960	-	chr7	56403050	-	Y	N	Y	Y	Y	N	transl_inter	na	NA	intronic;intro	FAM69A	-	protein_cod	0	intergenic	RP11-814E24.1,RNU6-13	+,-	pseudogene	11756;4874
2531	chr1	142540285	-	chr7	57552189	-	N	N	Y	N	N	N	transl_inter	na	NA	intergenic	NONE,RP11-417J8.1	NONE,+	NONE,lincR	NONE;13008	intergenic	RP11-1324A7.2,NCOR1F	+,+	pseudogene	6588;107311
2532	chr1	46168906	-	chr7	26337532	+	Y	Y	Y	Y	N	N	transl_inter	na	NA	intronic	IPP	-	protein_cod	0	intronic	SNX10	+	protein_cod	0
2533	chr1	230007144	-	chr7	157274857	+	Y	N	N	N	N	N	transl_inter	na	NA	intergenic	HMGB1P26,RP11-286	-,-	pseudogene	165536;4153	ncRNA_intro	AC006372.4	+	lincRNA	0
2534	chr1	149028989	+	chr7	57939250	-	N	N	N	N	N	Y	transl_inter	na	NA	intergenic	RP11-14N7.2,AL73236	+,-	lincRNA,miF	77394;7323	intergenic	RP11-548K12.12,NONE	+,NONE	pseudogene	42498;NONE
2535	chr1	158962770	+	chr7	15834186	-	N	N	N	Y	N	N	transl_inter	na	NA	intergenic	PYHIN1,IFI16	+,+	protein_cod	15926;6988	intergenic	RPL36AP26,AC006041.1	-,-	pseudogene	92809;46736
2536	chr1	188545957	+	chr7	33221759	-	N	N	N	N	Y	N	transl_inter	na	NA	intergenic	RP11-669M2.1,RP11-6	+,+	lincRNA,pse	40361;94527	intronic	BBS9	+	protein_cod	0
2537	chr1	146712219	+	chr7	48029733	+	N	N	Y	N	N	N	transl_inter	na	NA	ncRNA_intro	RP11-337C18.10	+	processed_	0	intronic	SUN3	-	protein_cod	0
2538	chr1	202764747	+	chr7	942066	+	N	N	N	Y	N	N	transl_inter	na	NA	intronic	KDM5B	-	protein_cod	0	intronic	ADAP1,COX19	-,-	protein_cod	0
2539	chr1	85983489	-	chr8	23064697	-	N	N	N	Y	Y	N	transl_inter	na	NA	intronic;intro	DDAH1	-	protein_cod	0	ncRNA_exo	RP11-1149O23.1	+	pseudogene	0
2540	chr1	202641860	-	chr8	131083323	-	Y	N	N	N	N	Y	transl_inter	na	NA	intronic	SYT2	-	protein_cod	0	intronic	ASAP1	-	protein_cod	0
2541	chr1	202642263	-	chr8	131083311	-	N	N	Y	N	N	N	transl_inter	na	NA	intronic	SYT2	-	protein_cod	0	intronic	ASAP1	-	protein_cod	0
2542	chr1	11031088	-	chr8	133253092	+	Y	N	N	N	N	N	transl_inter	na	NA	intronic	C1orf127	-	protein_cod	0	intronic	KCNQ3	-	protein_cod	0
2543	chr1	47827314	-	chr8	30597959	+	Y	Y	Y	Y	Y	Y	transl_inter	na	NA	intronic	CMPK1	+	protein_cod	0	ncRNA_intro	UBXN8	+	processed_	0
2544	chr1	53554926	-	chr8	67684363	+	N	N	Y	Y	N	N	transl_inter	na	NA	intronic	SLC1A7	-	protein_cod	0	intronic	SGK3,C8orf44-SGK3	+,+	protein_cod	0
2545	chr1	159419555	-	chr8	68079507	+	N	N	Y	N	N	N	transl_inter	na	NA	ncRNA_intro	RP11-550P17.5	-	antisense	0	intronic	CSPP1	+	protein_cod	0
2546	chr1	4827759	+	chr8	142837414	-	N	N	Y	N	N	N	transl_inter	na	NA	intronic;intro	AJAP1	+	protein_cod	0	intergenic;ir	AC138647.1,AC104417.1	+,+	protein_cod	308577;189446
2547	chr1	85983489	+	chr8	23064697	+	Y	Y	Y	Y	N	N	transl_inter	na	NA	intronic;intro	DDAH1	-	protein_cod	0	ncRNA_exo	RP11-1149O23.1	+	pseudogene	0
2548	chr1	95132990	+	chr8	64004615	+	N	N	Y	N	Y	N	transl_inter	na	NA	ncRNA_intro	LINC01057	-	lincRNA	0	intergenic	TTPA,RP11-16E18.1	-,-	protein_cod	6003;44167
2549	chr1	70926108	-	chr9	68418844	-	Y	N	N	Y	Y	N	transl_inter	na	NA	intergenic	RP11-42O15.3,RP11-4	+,+	lincRNA,pse	14889;70101	intergenic;ir	MIR4477A,RP11-764K9.4	+,-	miRNA,pseu	3456;9017
2550	chr1	80168979	+	chr9	68418844	-	Y	Y	Y	Y	Y	Y	transl_inter	na	NA	intergenic	ADH5P2,RP11-339A11	+,-	pseudogene	181091;388809	intergenic;ir	MIR4477A,RP11-764K9.4	+,-	miRNA,pseu	3456;9017
2551	chr1	86399021	+	chr9	100675547	-	Y	Y	N	N	N	Y	transl_inter	na	NA	intronic	COL24A1	-	protein_cod	0	UTR3	C9orf156	-	protein_cod	0
2552	chr1	101027320	+	chr9	2781617	-	N	N	Y	N	N	N	transl_inter	na	NA	intergenic	GPR88,RP11-84O12.2	+,-	protein_cod	19746;24885	intronic	KIAA0020	-	protein_cod	0
2553	chr1	160622887	+	chr9	68418844	-	Y	N	N	N	N	N	transl_inter	na	NA	intergenic	SLAMF1,SETP9	-,+	protein_cod	5802;17051	intergenic;ir	MIR4477A,RP11-764K9.4	+,-	miRNA,pseu	3456;9017
2554	chr1	31153049	+	chr9	132276979	+	Y	N	N	N	N	Y	transl_inter	na	NA	intergenic	RP4-591L5.2,MATN1	+,-	lincRNA,pro	259094;31075	intergenic	RP11-492E3.51,RP11-49	+,+	lincRNA,ant	1014;60709
2555	chr1	179651079	+	chr9	16965318	+	Y	Y	N	N	Y	N	transl_inter	na	NA	intronic;intro	TDRD5	+	protein_cod	0	intergenic	BNC2,RP11-132E11.2	-,+	protein_cod	94477;83599
2556	chr1	179651089	+	chr9	16965312	+	N	N	Y	N	N	N	transl_inter	na	NA	intronic	TDRD5	+	protein_cod	0	intergenic	BNC2,RP11-132E11.2	-,+	protein_cod	94471;83605
2557	chr1	228001325	+	chr9	16394002	+	N	N	N	N	Y	N	transl_inter	na	NA	intergenic	SNAP47,PRSS38	+,+	protein_cod	32398;2069	intergenic	C9orf92,BNC2	-,-	protein_cod	117691;15499
2558	chr1	164989715	-	chrX	133182299	-	N	N	N	N	Y	N	transl_inter	na	NA	intergenic	AL390119.1,SNORD11	-,-	miRNA,snol	36486;51995	intergenic	AF003529.2,Y_RNA	+,-	pseudogene	58186;93959
2559	chr1	35007077	-	chrX	109301022	+	Y	N	Y	Y	Y	Y	transl_inter	na	NA	intergenic;ir	RP4-657M3.2,MIR552	-,-	pseudogene	264184;128123	intronic	TMEM164	+	protein_cod	0
2560	chr1	77833408	-	chrX	127603684	+	N	N	N	N	N	Y	transl_inter	na	NA	intronic	AK5	+	protein_cod	0	ncRNA_intro	RP1-30E17.2	-	lincRNA	0
2561	chr1	164989581	+	chrX	133182188	+	Y	N	N	N	N	N	transl_inter	na	NA	intergenic	AL390119.1,SNORD11	-,-	miRNA,snol	36352;52129	intergenic	AF003529.2,Y_RNA	+,-	pseudogene	58075;94070
2562	chr1	33392070	-	chrY	14482300	-	N	N	N	N	Y	N	transl_inter	na	NA	downstream	AL031602.1	+	miRNA	0	ncRNA_intro	GYG2P1,ARSDP1	-,+	pseudogene	0
2563	chr1	142537607	-	chrY	58982136	+	N	N	N	N	Y	N	transl_inter	na	NA	intergenic	NONE,RP11-417J8.1	NONE,+	NONE,lincR	NONE;15686	intergenic	NONE,CTBP2P1	NONE,+	NONE,pseu	NONE;19255
2564	chr1	142541956	-	chrY	13446778	+	N	N	N	Y	N	N	transl_inter	na	NA	intergenic	NONE,RP11-417J8.1	NONE,+	NONE,lincR	NONE;11337	intergenic	AC134878.1,DUX4L16	-,+	miRNA,pseu	106145;15816
2565	chr1	142536050	+	chrY	58977992	-	N	N	Y	N	N	N	transl_inter	na	NA	intergenic	NONE,RP11-417J8.1	NONE,+	NONE,lincR	NONE;17243	intergenic	NONE,CTBP2P1	NONE,+	NONE,pseu	NONE;23399
2566	chr1	142536825	+	chrY	58977986	-	N	N	N	N	N	Y	transl_inter	na	NA	intergenic	NONE,RP11-417J8.1	NONE,+	NONE,lincR	NONE;16468	intergenic	NONE,CTBP2P1	NONE,+	NONE,pseu	NONE;23405
2567	chr1	142543016	+	chrY	13447010	-	N	N	N	N	Y	N	transl_inter	na	NA	intergenic	NONE,RP11-417J8.1	NONE,+	NONE,lincR	NONE;10277	intergenic	AC134878.1,DUX4L16	-,+	miRNA,pseu	106377;15584
2568	chr10	81865790	-	chr11	121108082	-	N	N	Y	N	N	N	transl_inter	na	NA	intergenic	TMEM254,RPL22P18	+,+	protein_cod	13477;19907	upstream	RP11-775A1.2	+	pseudogene	0
2569	chr10	12455553	+	chr11	3228044	+	N	N	N	N	N	Y	transl_inter	na	NA	intronic	CAMK1D	+	protein_cod	0	intergenic	AC108448.3,MRGPRG	+,-	antisense,pi	16885;11130
2570	chr10	12455588	+	chr11	3228033	+	N	N	N	N	Y	N	transl_inter	na	NA	intronic	CAMK1D	+	protein_cod	0	intergenic	AC108448.3,MRGPRG	+,-	antisense,pi	16874;11141
2571	chr10	12455960	+	chr11	3227910	+	Y	N	N	N	N	N	transl_inter	na	NA	intronic	CAMK1D	+	protein_cod	0	intergenic	AC108448.3,MRGPRG	+,-	antisense,pi	16751;11264
2572	chr10	47059384	+	chr11	89183607	+	N	N	N	N	N	Y	transl_inter	na	NA	ncRNA_intro	LINC00842	-	lincRNA	0	intronic	NOX4	-	protein_cod	0
2573	chr10	93792890	-	chr12	9476177	+	N	Y	N	N	N	N	transl_inter	na	NA	intergenic	BTAF1,CPEB3	+,-	protein_cod	2808;13559	intergenic	RP11-22B23.2,RP13-735	-,+	pseudogene	7408;43883
2574	chr10	97745956	-	chr12	29954615	+	Y	N	N	Y	N	N	transl_inter	na	NA	ncRNA_intro	ENTPD1-AS1	-	antisense	0	intergenic	TMTC1,U3	-,+	protein_cod	16923;98221
2575	chr10	97745960	-	chr12	29955068	+	N	N	Y	N	N	N	transl_inter	na	NA	ncRNA_intro	ENTPD1-AS1	-	antisense	0	intergenic	TMTC1,U3	-,+	protein_cod	17376;97768
2576	chr10	97745968	-	chr12	29954624	+	N	Y	N	N	N	N	transl_inter	na	NA	ncRNA_intro	ENTPD1-AS1	-	antisense	0	intergenic	TMTC1,U3	-,+	protein_cod	16932;98212
2577	chr10	97746006	-	chr12	29954991	+	N	N	N	N	Y	N	transl_inter	na	NA	ncRNA_intro	ENTPD1-AS1	-	antisense	0	intergenic	TMTC1,U3	-,+	protein_cod	17299;97845
2578	chr10	120108403	-	chr12	95786656	+	N	Y	Y	Y	N	Y	transl_inter	na	NA	intergenic	FAM204A,LINC00867	-,+	protein_cod	6571;8217	ncRNA_intro	RP11-167N24.4	-	lincRNA	0
2579	chr10	27775891	+	chr12	11191377	-	Y	Y	Y	Y	Y	Y	transl_inter	na	NA	intergenic	PTCHD3,RAB18	-,+	protein_cod	72594;17306	intergenic	AC018630.1,TAS2R63P	+,-	protein_cod	4102;9554
2580	chr10	120108404	+	chr12	95786655	-	N	Y	N	Y	Y	N	transl_inter	na	NA	intergenic	FAM204A,LINC00867	-,+	protein_cod	6572;8216	ncRNA_intro	RP11-167N24.4	-	lincRNA	0
2581	chr10	113001705	-	chr13	86917624	-	Y	Y	N	Y	Y	Y	transl_inter	na	NA	intergenic	RP11-479A21.1,BTBD7	+,+	pseudogene	136699;80831	intergenic;ir	RP11-30L8.1,DDX6P2	+,+	lincRNA,pse	172599;10131
2582	chr10	113001773	-	chr13	86917624	-	N	N	Y	N	N	N	transl_inter	na	NA	intergenic	RP11-479A21.1,BTBD7	+,+	pseudogene	136767;80763	intergenic;ir	RP11-30L8.1,DDX6P2	+,+	lincRNA,pse	172599;10131
2583	chr10	110217892	+	chr13	21817193	-	N	N	N	N	N	Y	transl_inter	na	NA	intergenic	PTGES3P5,RP11-163I	+,-	pseudogene	161602;89841	intergenic	LINC01046,ESRRAP2	+,+	lincRNA,pse	7850;16961
2584	chr10	9027371	+	chr13	109869231	+	N	N	Y	N	N	N	transl_inter	na	NA	intergenic	RP11-428L9.2,LINC00	+,+	lincRNA,linc	11940;290425	intergenic	MYO16,LINC00370	+,+	protein_cod	8876;65090
2585	chr10	9027394	+	chr13	109869254	+	N	Y	N	N	N	N	transl_inter	na	NA	intergenic	RP11-428L9.2,LINC00	+,+	lincRNA,linc	11963;290402	intergenic;ir	MYO16,LINC00370	+,+	protein_cod	8899;65067
2586	chr10	9027398	+	chr13	109869395	+	N	N	N	Y	N	N	transl_inter	na	NA	intergenic	RP11-428L9.2,LINC00	+,+	lincRNA,linc	11967;290398	intergenic	MYO16,LINC00370	+,+	protein_cod	9040;64926
2587	chr10	9027836	+	chr13	109869254	+	Y	N	N	N	Y	N	transl_inter	na	NA	intergenic	RP11-428L9.2,LINC00	+,+	lincRNA,linc	12405;289960	intergenic;ir	MYO16,LINC00370	+,+	protein_cod	8899;65067
2588	chr10	8911073	-	chr14	20778864	-	N	N	N	N	N	Y	transl_inter	na	NA	intergenic	RP11-575N15.1,RP11-	+,+	pseudogene	144822;28879	downstream	CCNB1IP1	-	protein_cod	0
2589	chr10	102281054	-	chr14	74928583	-	N	N	N	Y	N	N	transl_inter	na	NA	intronic	SEC31B,NDUFB8,NDU	-,-,-	protein_cod	0	intergenic	SYNDIG1L,RP3-449M8.9	-,-	protein_cod	35778;10050
2590	chr10	1393781	+	chr14	59913838	+	N	Y	N	N	N	N	transl_inter	na	NA	intronic	ADARB2	-	protein_cod	0	intronic	GPR135	-	protein_cod	0
2591	chr10	130017782	+	chr14	104911314	+	N	N	N	N	Y	N	transl_inter	na	NA	intergenic	MKI67,RP11-264E18.1	-,-	protein_cod	93133;96764	intergenic	RNU6-684P,CEND1P1	-,+	snRNA,pseu	43929;6002
2592	chr10	42660297	+	chr15	20114573	-	N	Y	N	N	N	N	transl_inter	na	NA	intergenic	KSR1P1,IGKV1OR10-	-,+	pseudogene	15300;20490	intergenic	RP11-173D3.3,IGHV1OR	+,-	pseudogene	9761;55346
2593	chr10	63180598	+	chr15	29850898	-	Y	N	N	N	N	N	transl_inter	na	NA	intronic	TMEM26	-	protein_cod	0	intronic	FAM189A1	-	protein_cod	0
2594	chr10	42645794	-	chr16	32096547	-	N	N	N	Y	N	N	transl_inter	na	NA	upstream	KSR1P1	-	pseudogene	0	upstream	RP11-1166P10.6	-	pseudogene	0
2595	chr10	42646014	-	chr16	32096777	-	N	N	N	N	N	Y	transl_inter	na	NA	intergenic	KSR1P1,IGKV1OR10-	-,+	pseudogene	1017;34773	upstream	RP11-1166P10.6	-	pseudogene	0
2596	chr10	42646039	-	chr16	32096775	-	Y	N	N	N	N	N	transl_inter	na	NA	intergenic	KSR1P1,IGKV1OR10-	-,+	pseudogene	1042;34748	upstream	RP11-1166P10.6	-	pseudogene	0
2597	chr10	42646407	-	chr16	32097047	-	N	N	Y	N	N	N	transl_inter	na	NA	intergenic;ir	KSR1P1,IGKV1OR10-	-,+	pseudogene	1410;34380	upstream	RP11-1166P10.6	-	pseudogene	0
2598	chr10	42646873	-	chr16	33041116	-	N	N	N	N	Y	N	transl_inter	na	NA	intergenic;ir	KSR1P1,IGKV1OR10-	-,+	pseudogene	1876;33914	intergenic;ir	RP11-19N8.2,RP11-19N8	-,-	pseudogene	1758;13814

2599	chr10	42646906	-	chr16	33041083	-	N	Y	N	N	N	N	transl_inter	na	NA	intergenic	KSR1P1,IGKV1OR10-	-,+	pseudogene	1909;33881	intergenic	RP11-19N8.2,RP11-19N	-,-	pseudogene	1725;13847
2600	chr10	42647269	-	chr16	33041116	-	Y	N	N	N	N	N	transl_inter	na	NA	intergenic	KSR1P1,IGKV1OR10-	-,+	pseudogene	2272;33518	intergenic;ir	RP11-19N8.2,RP11-19N	-,-	pseudogene	1758;13814
2601	chr10	42657874	-	chr16	33053452	-	N	N	N	N	Y	N	transl_inter	na	NA	intergenic	KSR1P1,IGKV1OR10-	-,+	pseudogene	12877;22913	intergenic	RP11-19N8.2,RP11-19N	-,-	pseudogene	14094;1478
2602	chr10	24846865	-	chr16	59084128	+	N	N	Y	N	N	N	transl_inter	na	NA	intergenic	KIAA1217,ARHGAP21	+,-	protein_cod	10093;25673	ncRNA_intr	RP11-410D17.2	+	lincRNA	0
2603	chr10	43330032	-	chr16	33486239	+	N	Y	Y	N	N	N	transl_inter	na	NA	UTR3	BMS1	+	protein_cod	0	intergenic	RP11-293B20.2,BMS1P8	+,-	pseudogene	61228;3253
2604	chr10	36759286	+	chr16	58102588	-	Y	Y	Y	Y	Y	Y	transl_inter	na	NA	intergenic	SNORA40,NAMPTL	+,-	lincRNA,pro	27966;51363	intergenic	MMP15,Y_RNA	+,-	protein_cod	21783;14384
2605	chr10	38483878	+	chr16	32558390	-	N	Y	Y	N	N	Y	transl_inter	na	NA	ncRNA_intr	RP11-508N22.8	+	processed_	0	intergenic	ABCD1P3,RP11-652G5.	-,+	pseudogene	68819;53230
2606	chr10	42660287	+	chr16	33863205	-	N	N	N	N	N	Y	transl_inter	na	NA	intergenic	KSR1P1,IGKV1OR10-	-,+	pseudogene	15290;20500	intergenic	RP11-598D12.2,CTD-252	+,+	pseudogene	9377;59732
2607	chr10	43330031	+	chr16	33486240	-	Y	N	N	Y	N	N	transl_inter	na	NA	UTR3	BMS1	+	protein_cod	0	intergenic	RP11-293B20.2,BMS1P8	+,-	pseudogene	61229;3252
2608	chr10	94137647	+	chr16	79864797	-	Y	Y	Y	Y	Y	Y	transl_inter	na	NA	intergenic;ir	MARCH5,MARK2P9	+,+	protein_cod	23926;40777	intergenic	RP11-345M22.3,RP11-14	-,+	lincRNA,linc	3750;209762
2609	chr10	42608148	+	chr16	46461626	+	N	N	N	Y	N	N	transl_inter	na	NA	intergenic	NONE,KSR1P1	NONE,-	NONE,pseu	NONE;36610	intergenic	NONE,ANKRD26P1	NONE,-	NONE,pseu	NONE;41627
2610	chr10	42608342	+	chr16	46462438	+	Y	N	N	N	N	N	transl_inter	na	NA	intergenic	NONE,KSR1P1	NONE,-	NONE,pseu	NONE;36416	intergenic	NONE,ANKRD26P1	NONE,-	NONE,pseu	NONE;40815
2611	chr10	42646394	+	chr16	32097252	+	N	N	Y	N	N	N	transl_inter	na	NA	intergenic	KSR1P1,IGKV1OR10-	-,+	pseudogene	1397;34393	intergenic	RP11-1166P10.6,HERC2	-,-	pseudogene	1146;17314
2612	chr10	74767463	-	chr17	32673451	-	N	N	N	N	N	Y	transl_inter	na	NA	UTR3	P4HA1	-	protein_cod	0	intergenic	RP11-521P1.1,CCL13	-,+	pseudogene	14204;10020
2613	chr10	74767482	-	chr17	32673374	+	Y	Y	Y	Y	Y	Y	transl_inter	na	NA	UTR3	P4HA1	-	protein_cod	0	intergenic	RP11-521P1.1,CCL13	-,+	pseudogene	14127;10097
2614	chr10	7608833	+	chr17	41394529	-	N	N	Y	Y	N	Y	transl_inter	na	NA	intronic	ITIH5	-	protein_cod	0	intergenic	LINC00854,LINC00910	-,-	processed_	11191;52684
2615	chr10	29612712	+	chr17	71709833	-	N	N	N	Y	N	N	transl_inter	na	NA	intergenic	LYZL1,PTCHD3P1	+,+	protein_cod	5455;85619	intergenic;ir	RP11-277J6.3,CTD-2532	-,+	lincRNA,linc	49846;24160
2616	chr10	74767073	+	chr17	32673664	-	N	N	Y	N	N	N	transl_inter	na	NA	UTR3	P4HA1	-	protein_cod	0	intergenic	RP11-521P1.1,CCL13	-,+	pseudogene	14417;9807
2617	chr10	74767371	+	chr17	32673166	-	N	Y	N	N	N	N	transl_inter	na	NA	UTR3	P4HA1	-	protein_cod	0	intergenic	RP11-521P1.1,CCL13	-,+	pseudogene	13919;10305
2618	chr10	74767537	+	chr17	32673377	+	Y	Y	Y	N	N	N	transl_inter	na	NA	UTR3	P4HA1	-	protein_cod	0	intergenic	RP11-521P1.1,CCL13	-,+	pseudogene	14130;10094
2619	chr10	103124746	+	chr17	41464740	+	N	N	N	N	N	Y	transl_inter	na	NA	ncRNA_exo	RNU2-59P	-	snRNA	0	ncRNA_exo	LINC00910	-	lincRNA	0
2620	chr10	101384882	-	chr18	24804906	-	Y	N	N	N	N	N	transl_inter	na	NA	intergenic;ir	RP11-85A1.3,RP11-48	+,-	antisense,ai	3207;26864	upstream;u	RP11-57J16.1	+	lincRNA	0
2621	chr10	101385129	-	chr18	24805124	-	N	N	N	N	N	Y	transl_inter	na	NA	intergenic	RP11-85A1.3,RP11-48	+,-	antisense,ai	3454;26617	upstream	RP11-57J16.1	+	lincRNA	0
2622	chr10	101384882	+	chr18	24804906	+	N	N	Y	N	N	N	transl_inter	na	NA	intergenic;ir	RP11-85A1.3,RP11-48	+,-	antisense,ai	3207;26864	upstream;u	RP11-57J16.1	+	lincRNA	0
2623	chr10	38739517	-	chr19	200277	+	N	N	N	N	Y	N	transl_inter	na	NA	intergenic	LINC00999,CICP9	+,-	protein_cod	2697;2398	ncRNA_intr	AC092299.7	+	pseudogene	0
2624	chr10	38894335	+	chr19	33444599	-	N	N	N	N	Y	N	transl_inter	na	NA	upstream	ABCD1P2	+	pseudogene	0	exonic;exon	CEP89	-	protein_cod	0
2625	chr10	12736478	-	chr2	108797385	-	N	N	N	Y	N	N	transl_inter	na	NA	intronic	CAMK1D	+	protein_cod	0	ncRNA_intr	AC019100.3	-	antisense	0
2626	chr10	84891868	-	chr2	132885378	-	N	N	N	N	N	Y	transl_inter	na	NA	intergenic	RNU6-478P,MARK2P1	-,-	snRNA,pseu	64041;179516	intergenic	AC098826.4,AC098826.	+,-	pseudogene	25241;5051
2627	chr10	85336922	-	chr2	213520421	-	N	N	Y	N	N	N	transl_inter	na	NA	intergenic	AL356140.1,RP11-344	-,-	miRNA,lincF	140037;95240	ncRNA_intr	AC079610.1	-	sense_over	0
2628	chr10	3139030	-	chr2	117853347	+	Y	Y	Y	Y	Y	Y	transl_inter	na	NA	intronic;intr	PFKP	+	protein_cod	0	intergenic;ir	AC012365.3,RNU7-190P	+,-	pseudogene	68747;43944
2629	chr10	42606075	-	chr2	92263715	+	Y	N	N	N	N	N	transl_inter	na	NA	intergenic	NONE,KSR1P1	NONE,-	NONE,pseu	NONE;38683	intergenic	IGKV1OR2-2,NONE	-,NONE	pseudogene	40689;NONE
2630	chr10	42607916	-	chr2	92261893	+	N	N	N	N	Y	N	transl_inter	na	NA	intergenic	NONE,KSR1P1	NONE,-	NONE,pseu	NONE;36842	intergenic	IGKV1OR2-2,NONE	-,NONE	pseudogene	38867;NONE
2631	chr10	42608093	-	chr2	92261733	+	N	Y	N	N	N	N	transl_inter	na	NA	intergenic	NONE,KSR1P1	NONE,-	NONE,pseu	NONE;36665	intergenic	IGKV1OR2-2,NONE	-,NONE	pseudogene	38707;NONE
2632	chr10	42645475	-	chr2	90413164	+	N	N	N	N	N	Y	transl_inter	na	NA	upstream	KSR1P1	-	pseudogene	0	intergenic	AC113612.1,CH17-132F	+,+	pseudogene	112762;45037
2633	chr10	42646873	-	chr2	90412547	+	N	N	N	N	N	Y	transl_inter	na	NA	intergenic;ir	KSR1P1,IGKV1OR10-	-,+	pseudogene	1876;33914	intergenic	AC113612.1,CH17-132F	+,+	pseudogene	112145;45654
2634	chr10	84855768	-	chr2	241602862	+	N	Y	N	Y	N	N	transl_inter	na	NA	intergenic;ir	RNU6-478P,MARK2P1	-,-	snRNA,pseu	27941;215616	intergenic;ir	GPR35,AQP12B	+,-	protein_cod	32186;12973
2635	chr10	3139030	+	chr2	117853347	-	Y	Y	Y	N	N	Y	transl_inter	na	NA	intronic;intr	PFKP	+	protein_cod	0	intergenic;ir	AC012365.3,RNU7-190P	+,-	pseudogene	68747;43944
2636	chr10	39127190	+	chr2	89857170	-	N	N	N	N	Y	N	transl_inter	na	NA	intergenic	RP11-96F8.1,NONE	+,NONE	pseudogene	50951;NONE	intergenic	IGKV2-40,IGKV2D-40	-,+	IG_V_gene,	226984;33431
2637	chr10	42722369	+	chr2	92179441	-	N	N	N	Y	N	N	transl_inter	na	NA	intergenic	AL031601.4,AL031601	+,-	pseudogene	16538;7982	intergenic	CHEK2P3,AC128677.3	+,-	pseudogene	27805;15265
2638	chr10	84855768	+	chr2	241602862	-	N	N	N	N	Y	N	transl_inter	na	NA	intergenic;ir	RNU6-478P,MARK2P1	-,-	snRNA,pseu	27941;215616	intergenic;ir	GPR35,AQP12B	+,-	protein_cod	32186;12973
2639	chr10	127586442	+	chr2	116809441	-	N	Y	Y	Y	Y	Y	transl_inter	na	NA	intronic	FANK1	+	protein_cod	0	intergenic	DPP10,RP11-338I24.1	+,-	protein_cod	206113;635353
2640	chr10	5423229	+	chr2	239778460	+	N	N	Y	N	N	N	transl_inter	na	NA	intergenic	UCN3,TUBAL3	+,-	protein_cod	7060;11832	intronic	TWIST2	+	protein_cod	0
2641	chr10	29538660	-	chr20	53465849	-	N	N	Y	N	N	N	transl_inter	na	NA	intergenic	RP11-492M23.2,LYZL	-,+	pseudogene	350238;39330	intergenic	DOK5,RNU4ATAC7P	+,-	protein_cod	198139;10378
2642	chr10	54593908	-	chr20	59958287	+	N	N	Y	N	N	N	transl_inter	na	NA	intergenic	MBL2,Y_RNA	-,+	protein_cod	62448;18459	intronic	CDH4	+	protein_cod	0
2643	chr10	25743163	+	chr20	21159035	+	N	Y	Y	N	N	N	transl_inter	na	NA	intronic	GPR158	+	protein_cod	0	ncRNA_intr	PLK1S1,RP4-777D9.2	+,-	processed_	0
2644	chr10	38817034	-	chr21	10855366	-	N	N	N	N	N	Y	transl_inter	na	NA	intergenic	RP11-291L22.4,ABCD	+,+	lincRNA,pse	61930;77515	intergenic	bP-21201H5.1,IGHV1OR	-,+	pseudogene	379305;7256
2645	chr10	38804636	+	chr21	10828081	+	N	N	Y	Y	N	N	transl_inter	na	NA	intergenic	RP11-291L22.4,ABCD	+,+	lincRNA,pse	49532;89913	intergenic	bP-21201H5.1,IGHV1OR	-,+	pseudogene	352020;34541
2646	chr10	78376914	+	chr21	22497502	+	N	Y	N	N	N	N	transl_inter	na	NA	intergenic	RP11-325D15.2,SNOR	-,-	antisense,si	56162;218221	intronic	NCAM2	+	protein_cod	0
2647	chr10	38909050	-	chr22	16884287	-	N	N	N	Y	N	N	transl_inter	na	NA	intergenic	ABCD1P2,PABPC1P1	+,+	pseudogene	10310;19989	intergenic	ABCD1P4,LA16c-17H1.3	+,+	pseudogene	12674;1769
2648	chr10	74700212	-	chr22	39582334	-	N	Y	N	Y	N	N	transl_inter	na	NA	intronic	PLA2G12B	-	protein_cod	0	intergenic	FUNDC2P4,PDGFB	+,-	pseudogene	30384;37030
2649	chr10	45321123	-	chr22	30785509	+	N	N	N	N	N	Y	transl_inter	na	NA	ncRNA_intr	TMEM72-AS1	-	antisense	0	intronic	RNF215	-	protein_cod	0
2650	chr10	50950782	+	chr22	50777155	-	N	N	N	N	N	Y	transl_inter	na	NA	intronic	OGDHL	-	protein_cod	0	intergenic	DENND6B,PPP6R2	-,+	protein_cod	11666;4578
2651	chr10	96291350	-	chr3	183429590	-	N	Y	N	N	N	N	transl_inter	na	NA	intronic	TBC1D12	+	protein_cod	0	intronic	YEATS2	+	protein_cod	0
2652	chr10	96291474	-	chr3	183430039	-	N	N	N	Y	N	N	transl_inter	na	NA	intronic	TBC1D12	+	protein_cod	0	intronic	YEATS2	+	protein_cod	0
2653	chr10	101851839	-	chr3	12696232	-	Y	Y	Y	Y	Y	Y	transl_inter	na	NA	intergenic	CPN1,TPM4P1	-,-	protein_cod	10205;22335	intronic	RAF1	-	protein_cod	0
2654	chr10	65545582	-	chr3	192376526	+	N	N	Y	Y	N	N	transl_inter	na	NA	ncRNA_intr	RP11-170M17.1	+	lincRNA	0	intronic	FGF12	-	protein_cod	0
2655	chr10	129512705	-	chr3	165027233	+	N	N	Y	N	N	N	transl_inter	na	NA	intergenic	NPS,AL391005.1	+,-	protein_cod	161770;21618	ncRNA_intr	RP11-85M11.2	+	lincRNA	0
2656	chr10	96291381	+	chr3	183430030	+	N	N	N	N	Y	N	transl_inter	na	NA	intronic	TBC1D12	+	protein_cod	0	intronic	YEATS2	+	protein_cod	0
2657	chr10	96291519	+	chr3	183429991	+	Y	N	N	N	N	N	transl_inter	na	NA	intronic	TBC1D12	+	protein_cod	0	intronic	YEATS2	+	protein_cod	0
2658	chr10	53364405	-	chr4	187971441	+	N	N	N	N	Y	N	transl_inter	na	NA	intronic	PRKG1	+	protein_cod	0	intergenic	RP11-217E13.1,RP11-69	-,+	lincRNA,linc	28314;9812
2659	chr10	53364455	-	chr4	187971458	+	Y	Y	Y	N	N	Y	transl_inter	na	NA	intronic	PRKG1	+	protein_cod	0	intergenic	RP11-217E13.1,RP11-69	-,+	lincRNA,linc	28331;9795
2660	chr10	53364535	-	chr4	187971468	+	N	N	N	Y	N	N	transl_inter	na	NA	intronic	PRKG1	+	protein_cod	0	intergenic	RP11-217E13.1,RP11-69	-,+	lincRNA,linc	28341;9785
2661	chr10	85222519	-	chr4	4350735	+	N	N	Y	N	N	N	transl_inter	na	NA	intergenic	AL356140.1,RP11-344	-,-	miRNA,lincF	25634;209643	intronic	NSG1	+	protein_cod	0
2662	chr10	85222525	-	chr4	4350755	+	N	Y	N	N	N	Y	transl_inter	na	NA	intergenic;ir	AL356140.1,RP11-344	-,-	miRNA,lincF	25640;209637	intronic;intr	NSG1	+	protein_cod	0
2663	chr10	85222585	-	chr4	4350811	+	N	N	N	Y	N	N	transl_inter	na	NA	intergenic	AL356140.1,RP11-344	-,-	miRNA,lincF	25700;209577	intronic	NSG1	+	protein_cod	0
2664	chr10	85222459	+	chr4	4350731	-	N	Y	N	N	N	N	transl_inter	na	NA	intergenic	AL356140.1,RP11-344	-,-	miRNA,lincF	25574;209703	intronic	NSG1	+	protein_cod	0
2665	chr10	85222494	+	chr4	4350705	-	N	N	N	N	N	Y	transl_inter	na	NA	intergenic	AL356140.1,RP11-344	-,-	miRNA,lincF	25609;209668	intronic	NSG1	+	protein_cod	0
2666	chr10	133522667	+	chr4	183881405	+	N	N	N	N	Y	N	transl_inter	na	NA	intergenic	AL450307.2,AL450307	+,-	miRNA,prot	33083;82067	intergenic	DCTD,RP11-335L23.2	-,-	protein_cod	42316;68349
2667	chr10	26045353	-	chr5	110229191	+	N	Y	N	N	N	N	transl_inter	na	NA	intergenic	HIRAP1,RNA5SP306	+,-	pseudogene	64101;57387	intergenic	NONE,CTD-2026D23.1	NONE,+	NONE,pseu	NONE;14792
2668	chr10	26045372	-	chr5	110229242	+	Y	N	N	N	N	N	transl_inter	na	NA	intergenic	HIRAP1,RNA5SP306	+,-	pseudogene	64120;57368	intergenic	NONE,CTD-2026D23.1	NONE,+	NONE,pseu	NONE;14741
2669	chr10	43677628	-	chr5	45151782	+	N	N	N	Y	N	N	transl_inter	na	NA	intronic	CSGALNACT2	+	protein_cod	0	intergenic	RP11-357F12.1,HCN1	+,-	lincRNA,pro	53033;107567
2670	chr10	84978863	-	chr5	103363719	+	N	N	N	N	Y	N	transl_inter	na	NA	intergenic	RNU6-478P,MARK2P1	-,-	snRNA,pseu	151036;92521	intergenic	NUDT12,RP11-138J23.1	-,+	protein_cod	465225;51893
2671	chr10	84978899	-	chr5	103363827	+	N	N	Y	N	N	Y	transl_inter	na	NA	intergenic	RNU6-478P,MARK2P1	-,-	snRNA,pseu	151072;92485	intergenic	NUDT12,RP11-138J23.1	-,+	protein_cod	465333;51785
2672	chr10	26045421	+	chr5	110229493	-	N	N	N	N	Y	N	transl_inter	na	NA	intergenic	HIRAP1,RNA5SP306	+,-	pseudogene	64169;57319	intergenic	NONE,CTD-2026D23.1	NONE,+	NONE,pseu	NONE;14490
2673	chr10	128616059	+	chr5	141945799	-	Y	Y	Y	Y	Y	N	transl_inter	na	NA	intronic	DOCK1	+	protein_cod	0	ncRNA_intr	AC005592.2	+	antisense	0
2674	chr10	12373331	+	chr5	59387597	+	N	Y	N	N	Y	Y	transl_inter	na	NA	intergenic	RN7SL198P,CAMK1D	-,+	misc_RNA,	2943;18150	intronic	PDE4D	-	protein_cod	0
2675	chr10	54594174	-	chr6	70631253	-	N	N	N	N	N	Y	transl_inter	na	NA	intergenic	MBL2,Y_RNA	-,+	protein_cod	62714;18193	intronic	COL19A1	+	protein_cod	0
2676	chr10	99804295	-	chr6	160640221	-	N	N	N	N	N	Y	transl_inter	na	NA	intergenic	CRTAC1,R3HCC1L	-,+	protein_cod	13710;90092	intronic	SLC22A2	-	protein_cod	0
2677	chr10	43824753	-	chr6	69831525	+	N	Y	N	N	N	N	transl_inter	na	NA	intergenic	RP11-168L22.2,RNU6	+,+	lincRNA,snF	1373;12481	intronic	BAI3	+	protein_cod	0
2678	chr10	43833103	-	chr6	151776828	+	N	Y	N	N	N	N	transl_inter	na	NA	intergenic;ir	RP11-168L22.2,RNU6	+,+	lincRNA,snF	9723;4131	intronic;intr	C6orf211	+	protein_cod	0
2679	chr10	43833147	-	chr6	151776815	+	N	N	N	Y	N	N	transl_inter	na	NA	intergenic	RP11-168L22.2,RNU6	+,+	lincRNA,snF	9767;4087	intronic	C6orf211	+	protein_cod	0
2680	chr10	43833314	-	chr6	151776899	+	N	N	N	N	Y	Y	transl_inter	na	NA	intergenic	RP11-168L22.2,RNU6	+,+	lincRNA,snF	9934;3920	intronic	C6orf211	+	protein_cod	0
2681	chr10	8787667	+	chr6	3635616	-	N	N	Y	N	N	N	transl_inter	na	NA	intergenic	RP11-575N15.1,RP11-	+,+	pseudogene	21416;152285	intergenic	RP1-223B1.1,RP1-72E1	-,-	lincRNA,pse	10635;58541
2682	chr10	23174686	+	chr6	51005005	-	N	Y	N	N	N	N	transl_inter	na	NA	intergenic	RNU6-413P,ARMC3	+,+	snRNA,prot	98258;42267	intergenic	FTH1P5,RP3-437C15.2	-,-	pseudogene	124036;245075

2683	chr10	23174839	+	chr6	51004999	-	N	N	N	N	Y	N	transl_inter	na	NA	intergenic	RNU6-413P,ARMC3	+,+	snRNA,prot	98411;42114	intergenic	FTH1P5,RP3-437C15.2	-,-	pseudogene	124030;245081
2684	chr10	43833103	+	chr6	151776828	-	N	N	N	N	Y	N	transl_inter	na	NA	intergenic;ir	RP11-168L22.2,RNU6	+,+	lincRNA,snF	9723;4131	intronic;intro	C6orf211	+	protein_cod	0
2685	chr10	81364791	+	chr6	119717007	+	N	Y	Y	N	Y	N	transl_inter	na	NA	intergenic	SFTPA3P,SFTPA1	-,+	pseudogene	9375;5904	intergenic	MAN1A1,MIR3144	-,+	protein_cod	46081;619318
2686	chr10	94137647	+	chr6	153429840	+	Y	Y	Y	Y	Y	N	transl_inter	na	NA	intergenic;ir	MARCH5,MARK2P9	+,+	protein_cod	23926;40777	intronic	RGS17	-	protein_cod	0
2687	chr10	100107199	+	chr6	167383381	+	N	N	N	N	N	Y	transl_inter	na	NA	intergenic	LOXL4,PYROXD2	-,-	protein_cod	79192;36123	ncRNA_intr	RP1-167A14.2	-	antisense	0
2688	chr10	42655433	-	chr7	64955715	-	Y	Y	N	Y	N	N	transl_inter	na	NA	intergenic	KSR1P1,IGKV1OR10-	-,+	pseudogene	10436;25354	intergenic	RP11-667F9.2,RP11-328	+,-	lincRNA,pse	27364;17971
2689	chr10	42641419	-	chr7	61825350	+	Y	N	Y	N	N	N	transl_inter	na	NA	intergenic	NONE,KSR1P1	NONE,-	NONE,pseu	NONE;3339	intergenic	RP11-715L17.1,RNU6-41	+,-	pseudogene	3164;646491
2690	chr10	42651756	-	chr7	61815117	+	Y	N	Y	N	Y	N	transl_inter	na	NA	intergenic	KSR1P1,IGKV1OR10-	-,+	pseudogene	6759;29031	intergenic	NONE,RP11-715L17.1	NONE,+	NONE,pseu	NONE;6752
2691	chr10	88214596	-	chr7	45032213	+	N	N	N	N	Y	N	transl_inter	na	NA	intronic	WAPAL	-	protein_cod	0	intergenic	SNHG15,CCM2	-,+	lincRNA,pro	5653;7571
2692	chr10	96413440	-	chr7	136260862	+	N	N	N	N	N	Y	transl_inter	na	NA	intergenic	CTBP2P2,CYP2C18	-,+	pseudogene	5687;29811	intergenic	AC009541.1,hsa-mir-490	+,-	pseudogene	87797;109444
2693	chr10	129425706	-	chr7	142326198	+	N	N	N	N	Y	N	transl_inter	na	NA	intergenic	NPS,AL391005.1	+,-	protein_cod	74771;108617	upstream	TRBV19	+	TR_V_gene	0
2694	chr10	42645025	+	chr7	61821762	-	N	N	N	Y	Y	N	transl_inter	na	NA	upstream	KSR1P1	-	pseudogene	0	upstream	RP11-715L17.1	+	pseudogene	0
2695	chr10	42645197	+	chr7	61821651	-	Y	N	N	N	N	N	transl_inter	na	NA	upstream	KSR1P1	-	pseudogene	0	upstream	RP11-715L17.1	+	pseudogene	0
2696	chr10	42646390	+	chr7	61820593	-	N	N	N	N	Y	N	transl_inter	na	NA	intergenic	KSR1P1,IGKV1OR10-	-,+	pseudogene	1393;34397	intergenic	NONE,RP11-715L17.1	NONE,+	NONE,pseu	NONE;1276
2697	chr10	42646407	+	chr7	61820920	-	N	N	N	N	N	Y	transl_inter	na	NA	intergenic;ir	KSR1P1,IGKV1OR10-	-,+	pseudogene	1410;34380	upstream	RP11-715L17.1	+	pseudogene	0
2698	chr10	112178163	+	chr7	92976835	+	Y	Y	Y	Y	Y	N	transl_inter	na	NA	intergenic;ir	SMNDC1,RP11-525A1	-,-	protein_cod	113454;10435	intronic;intro	CCDC132	+	protein_cod	0
2699	chr10	50462280	-	chr8	144263200	-	Y	N	N	N	N	N	transl_inter	na	NA	intergenic;ir	C10orf128,C10orf71-A	-,-	protein_cod	65844;41877	intergenic	LY6H,GPIHBP1	-,+	protein_cod	21072;31868
2700	chr10	50463099	-	chr8	144263502	-	N	Y	N	N	N	N	transl_inter	na	NA	intergenic	C10orf128,C10orf71-A	-,-	protein_cod	66663;41058	intergenic	LY6H,GPIHBP1	-,+	protein_cod	21374;31566
2701	chr10	128190491	+	chr8	108569292	-	Y	Y	N	Y	Y	N	transl_inter	na	NA	intronic	C10orf90	-	protein_cod	0	intergenic	ANGPT1,PGAM1P13	-,+	protein_cod	59009;90215
2702	chr10	128190812	+	chr8	108569293	-	N	N	Y	N	N	Y	transl_inter	na	NA	intronic	C10orf90	-	protein_cod	0	intergenic	ANGPT1,PGAM1P13	-,+	protein_cod	59010;90214
2703	chr10	50462103	+	chr8	144263599	+	N	N	N	N	Y	N	transl_inter	na	NA	intergenic	C10orf128,C10orf71-A	-,-	protein_cod	65667;42054	intergenic	LY6H,GPIHBP1	-,+	protein_cod	21471;31469
2704	chr10	50462206	+	chr8	144263625	+	N	N	Y	N	N	N	transl_inter	na	NA	intergenic	C10orf128,C10orf71-A	-,-	protein_cod	65770;41951	intergenic	LY6H,GPIHBP1	-,+	protein_cod	21497;31443
2705	chr10	2284614	-	chr9	31430900	-	N	N	Y	N	N	Y	transl_inter	na	NA	intergenic	RNU6-576P,LINC0070	+,-	snRNA,lincF	42932;62663	intergenic	RP11-271O3.1,RP11-291	-,+	lincRNA,pse	49412;74378
2706	chr10	6248458	-	chr9	6798834	-	Y	N	N	N	N	N	transl_inter	na	NA	intronic	PFKFB3	+	protein_cod	0	intronic	KDM4C	+	protein_cod	0
2707	chr10	16000947	-	chr9	115937378	-	Y	N	Y	N	Y	Y	transl_inter	na	NA	intergenic	snoU13,SNORA31	+,+	snoRNA,sno	72483;46860	intronic	FKBP15	-	protein_cod	0
2708	chr10	84978886	-	chr9	30282818	-	N	N	Y	N	N	N	transl_inter	na	NA	intergenic	RNU6-478P,MARK2P1	-,-	snRNA,pseu	151059;92498	intergenic	snoU13,RP11-255N24.4	-,-	snoRNA,pse	104243;276060
2709	chr10	133431405	-	chr9	79870097	-	Y	N	Y	N	Y	Y	transl_inter	na	NA	intergenic	TCERG1L,AL450307.2	-,+	protein_cod	321421;58110	intronic	VPS13A	+	protein_cod	0
2710	chr10	64131349	+	chr9	77952725	+	N	N	N	Y	N	N	transl_inter	na	NA	intergenic	RN7SL591P,ZNF365	+,+	misc_RNA,p	21301;2602	intergenic	RNU6-1228P,RP11-323A	+,-	snRNA,pse	139632;64080
2711	chr10	133431402	-	chrX	115833392	-	Y	Y	Y	Y	Y	Y	transl_inter	na	NA	intergenic	TCERG1L,AL450307.2	-,+	protein_cod	321418;58113	intergenic	RP11-232D9.3,RP11-232	-,-	lincRNA,pse	5236;49106
2712	chr10	82972199	-	chrX	96997205	+	N	N	Y	N	N	N	transl_inter	na	NA	intergenic	AL356154.1,NRG3	+,+	miRNA,prot	67712;662871	intergenic	DIAPH2-AS1,ERVWE2	-,+	antisense,p	99617;105056
2713	chr10	117006341	+	chrX	128670973	-	N	N	Y	N	N	Y	transl_inter	na	NA	intronic	ATRNL1	+	protein_cod	0	intergenic	SMARCA1,OCRL	-,+	protein_cod	13496;3279
2714	chr10	39105298	-	chrY	13825037	+	N	Y	Y	N	Y	Y	transl_inter	na	NA	intergenic	RP11-96F8.1,NONE	+,NONE	pseudogene	29059;NONE	intergenic	RP11-295P22.2,RCC2P1	-,-	pseudogene	195124;76721
2715	chr10	39111210	-	chrY	13866228	+	N	N	N	N	Y	N	transl_inter	na	NA	intergenic	RP11-96F8.1,NONE	+,NONE	pseudogene	34971;NONE	intergenic	RP11-295P22.2,RCC2P1	-,-	pseudogene	236315;35530
2716	chr10	42436315	-	chrY	9951878	+	N	Y	N	N	N	N	transl_inter	na	NA	intergenic	NONE,KSR1P1	NONE,-	NONE,pseu	NONE;208443	intergenic;ir	RNA5SP519,AC006987.(	-,-	rRNA,pseud	21276;55519
2717	chr10	39088020	+	chrY	13866909	-	N	N	N	Y	N	N	transl_inter	na	NA	intergenic	RP11-96F8.1,NONE	+,NONE	pseudogene	11781;NONE	intergenic	RP11-295P22.2,RCC2P1	-,-	pseudogene	236996;34849
2718	chr10	39134809	+	chrY	13810534	+	N	Y	N	N	Y	N	transl_inter	na	NA	intergenic	RP11-96F8.1,NONE	+,NONE	pseudogene	58570;NONE	intergenic	RP11-295P22.2,RCC2P1	-,-	pseudogene	180621;91224
2719	chr11	48378753	-	chr12	45460372	-	Y	Y	N	N	N	N	transl_inter	na	NA	intergenic;ir	OR4C4P,OR4C5	-,-	pseudogene	4754;8284	ncRNA_intr	RP11-478B9.1	+	antisense	0
2720	chr11	71989347	-	chr12	120357813	-	Y	Y	N	Y	Y	N	transl_inter	na	NA	intergenic	AP000593.5,AP00059:	-,+	pseudogene	27015;1848	intergenic	CIT,RPL35AP30	-,+	protein_cod	42718;39415
2721	chr11	68956514	-	chr12	108621766	+	N	N	N	N	N	Y	transl_inter	na	NA	intergenic	RP11-554A11.8,MYEO	-,+	lincRNA,pro	17482;105091	intronic	WSCD2	+	protein_cod	0
2722	chr11	18676976	+	chr12	52662948	+	N	Y	N	N	N	N	transl_inter	na	NA	intergenic	SPTY2D1,RP11-137N:	-,+	protein_cod	20936;9621	intronic	KRT86	+	protein_cod	0
2723	chr11	48378764	+	chr12	45460358	+	Y	Y	N	N	N	N	transl_inter	na	NA	intergenic	OR4C4P,OR4C5	-,-	pseudogene	4765;8273	ncRNA_intr	RP11-478B9.1	+	antisense	0
2724	chr11	108585748	-	chr13	21750661	-	Y	N	Y	Y	Y	N	transl_inter	na	NA	intronic	DDX10	+	protein_cod	0	UTR5	SKA3	-	protein_cod	0
2725	chr11	83126518	+	chr13	91319580	-	N	N	N	N	N	Y	transl_inter	na	NA	ncRNA_intr	RP11-727A23.10	+	antisense	0	intergenic	LINC01049,RNU6-75P	+,-	lincRNA,snF	132246;114440
2726	chr11	83126557	+	chr13	91319541	-	N	N	Y	N	N	N	transl_inter	na	NA	ncRNA_intr	RP11-727A23.10	+	antisense	0	intergenic	LINC01049,RNU6-75P	+,-	lincRNA,snF	132207;114479
2727	chr11	100529280	+	chr13	21817188	-	N	N	N	N	Y	N	transl_inter	na	NA	intergenic	RN7SL222P,AP00062(	-,+	misc_RNA,p	16276;7910	intergenic	LINC01046,ESRRAP2	+,+	lincRNA,pse	7845;16966
2728	chr11	108585765	+	chr13	21727734	+	Y	Y	Y	Y	Y	Y	transl_inter	na	NA	intronic	DDX10	+	protein_cod	0	UTR3	SKA3	-	protein_cod	0
2729	chr11	42740528	-	chr14	53433231	+	N	N	N	N	N	Y	transl_inter	na	NA	intergenic	CTD-2572N17.1,CTD-:	-,+	lincRNA,pse	465266;220797	intergenic	FERMT2,RP11-368P15.1	-,+	protein_cod	14078;45458
2730	chr11	73828083	-	chr14	25599042	+	Y	Y	N	Y	N	N	transl_inter	na	NA	intronic	C2CD3	-	protein_cod	0	intergenic	RP11-89K22.1,HMGN2P	-,-	lincRNA,pse	2838;137228
2731	chr11	503565	+	chr14	77736125	-	N	N	N	N	N	Y	transl_inter	na	NA	UTR5	RNH1	-	protein_cod	0	intronic	NGB	-	protein_cod	0
2732	chr11	14863243	+	chr14	56319805	-	Y	Y	N	N	Y	Y	transl_inter	na	NA	intronic;intro	PDE3B	+	protein_cod	0	intergenic;ir	LINC00520,RP11-1012E	-,+	processed_	56399;62640
2733	chr11	20196273	-	chr15	24871894	-	Y	N	N	N	N	N	transl_inter	na	NA	intergenic	DBX1,HTATIP2	-,+	protein_cod	14114;188958	ncRNA_intr	PWRN1	+	lincRNA	0
2734	chr11	79901973	-	chr15	24871850	-	Y	N	N	N	N	N	transl_inter	na	NA	intergenic	RP11-885L14.1,CTD-2	+,-	pseudogene	201300;130691	ncRNA_intr	PWRN1	+	lincRNA	0
2735	chr11	117240874	+	chr15	67156988	-	N	N	N	N	N	Y	transl_inter	na	NA	intronic	CEP164	+	protein_cod	0	intergenic;ir	RP11-622C24.1,RP11-79	+,+	pseudogene	54090;55161
2736	chr11	47660244	-	chr16	53044944	-	N	N	N	N	Y	N	transl_inter	na	NA	intronic	MTCH2	-	protein_cod	0	ncRNA_exo	RP11-467J12.3	-	pseudogene	0
2737	chr11	70649890	+	chr16	88489557	-	N	N	N	Y	N	N	transl_inter	na	NA	intronic	SHANK2	-	protein_cod	0	intergenic	LA16c-444G7.1,ZNF469	+,+	lincRNA,pro	153440;4322
2738	chr11	68956479	+	chr16	86621521	+	N	N	N	N	N	Y	transl_inter	na	NA	intergenic	RP11-554A11.8,MYEO	-,+	lincRNA,pro	17447;105126	intergenic	FOXL1,RP11-463O9.6	+,-	protein_cod	6218;48413
2739	chr11	68956757	+	chr16	86621278	+	N	N	N	Y	N	N	transl_inter	na	NA	intergenic	RP11-554A11.8,MYEO	-,+	lincRNA,pro	17725;104848	intergenic	FOXL1,RP11-463O9.6	+,-	protein_cod	5975;48656
2740	chr11	67427107	-	chr17	41401198	-	N	N	N	N	Y	N	transl_inter	na	NA	intergenic	AP003385.2,ALDH3B2	-,-	pseudogene	6232;2526	intergenic	LINC00854,LINC00910	-,-	processed_	17860;46015
2741	chr11	13565875	+	chr17	4538201	-	N	Y	N	N	N	N	transl_inter	na	NA	intergenic	PTH,HMGN2P36	-,-	protein_cod	48147;66035	intronic	ALOX15	-	protein_cod	0
2742	chr11	96457582	+	chr17	16738996	-	N	Y	N	N	N	Y	transl_inter	na	NA	intergenic	RP11-49K4.2,RP11-36	+,+	lincRNA,linc	209670;3735	intergenic	KRT16P2,KRT17P1	-,-	pseudogene	2849;5061
2743	chr11	104556130	+	chr17	9525359	-	Y	N	Y	Y	Y	Y	transl_inter	na	NA	intergenic	RP11-681H10.1,RP11-	-,+	pseudogene	2821;187861	intronic	WDR16	+	protein_cod	0
2744	chr11	76903926	-	chr19	14706814	-	N	Y	N	Y	N	N	transl_inter	na	NA	intronic	MYO7A	+	protein_cod	0	intronic	CLEC17A	+	protein_cod	0
2745	chr11	74064879	-	chr19	12473393	+	Y	Y	Y	Y	N	Y	transl_inter	na	NA	intronic	PGM2L1	-	protein_cod	0	intronic	ZNF442	-	protein_cod	0
2746	chr11	74064911	-	chr19	12473508	+	N	N	N	N	Y	N	transl_inter	na	NA	intronic	PGM2L1	-	protein_cod	0	intronic	ZNF442	-	protein_cod	0
2747	chr11	89872358	-	chr19	39511139	+	N	Y	N	N	N	N	transl_inter	na	NA	intronic	NAALAD2	+	protein_cod	0	intronic	FBXO27	-	protein_cod	0
2748	chr11	47924185	+	chr19	57838823	-	N	N	Y	N	N	N	transl_inter	na	NA	intergenic	NUP160,RP11-793I11.	-,-	protein_cod	54078;2690	intronic	ZNF543	+	protein_cod	0
2749	chr11	74064906	+	chr19	12473232	-	N	N	Y	N	N	N	transl_inter	na	NA	intronic	PGM2L1	-	protein_cod	0	intronic	ZNF442	-	protein_cod	0
2750	chr11	61735087	-	chr2	181738277	-	Y	N	Y	N	Y	N	transl_inter	na	NA	UTR5	FTH1	-	protein_cod	0	upstream;u	FTH1P20	-	pseudogene	0
2751	chr11	18523067	+	chr2	117708124	-	Y	Y	Y	Y	Y	Y	transl_inter	na	NA	intronic	TSG101	-	protein_cod	0	intergenic	AC062016.3,MTND1P28	+,+	pseudogene	131787;73370
2752	chr11	124246389	+	chr2	229533447	-	Y	Y	Y	Y	Y	Y	transl_inter	na	NA	downstream	OR8C1P	-	pseudogene	0	intergenic	AC009410.1,AC074019.	-,-	lincRNA,linc	57336;14806
2753	chr11	47915008	+	chr2	89219563	+	N	N	N	N	Y	N	transl_inter	na	NA	intergenic	NUP160,RP11-793I11.	-,-	protein_cod	44901;11867	intergenic	IGKV7-3,PGBD4P5	-,-	IG_V_pseu	4669;2281
2754	chr11	131178153	-	chr20	48731311	+	N	N	N	Y	N	Y	transl_inter	na	NA	ncRNA_intr	AP002856.5	+	lincRNA	0	intronic;intro	TMEM189-UBE2V1,TME	-,-,-	protein_cod	0
2755	chr11	67267014	+	chr20	2592460	-	N	N	N	N	N	Y	transl_inter	na	NA	intronic	PITPNM1	-	protein_cod	0	intronic	TMC2	+	protein_cod	0
2756	chr11	127713971	+	chr20	52032234	-	N	Y	N	N	N	N	transl_inter	na	NA	intergenic	RP11-822P1.1,RP11-6	+,-	pseudogene	186199;96837	ncRNA_intr	RP4-678D15.1	-	antisense	0
2757	chr11	131178153	+	chr20	48731311	-	Y	N	N	N	N	N	transl_inter	na	NA	ncRNA_intr	AP002856.5	+	lincRNA	0	intronic;intro	TMEM189-UBE2V1,TME	-,-,-	protein_cod	0
2758	chr11	42740511	+	chr20	38700439	+	Y	Y	Y	Y	Y	Y	transl_inter	na	NA	intergenic;ir	CTD-2572N17.1,CTD-:	-,+	lincRNA,pse	465249;220814	intergenic	RP11-101E14.3,AL00905	+,-	lincRNA,miF	27908;31421
2759	chr11	56395254	-	chr22	29065611	-	Y	Y	Y	Y	Y	Y	transl_inter	na	NA	intergenic	OR5AM1P,OR5M12P	-,+	pseudogene	6968;1154	intronic	TTC28	-	protein_cod	0
2760	chr11	3236135	-	chr22	37355467	+	N	N	Y	N	N	N	transl_inter	na	NA	intergenic	AC108448.3,MRGPRG	+,-	antisense,p	24976;3039	intergenic	CSF2RBP1,LL22NC01-8	-,+	pseudogene	5703;5822
2761	chr11	103570370	-	chr22	44126079	+	N	N	N	N	Y	Y	transl_inter	na	NA	ncRNA_intr	RP11-563P16.1	-	lincRNA	0	intronic	EFCAB6	-	protein_cod	0
2762	chr11	56395263	+	chr22	29065912	-	Y	Y	Y	Y	Y	Y	transl_inter	na	NA	intergenic	OR5AM1P,OR5M12P	-,+	pseudogene	6977;1145	intronic;intro	TTC28	-	protein_cod	0
2763	chr11	23038137	-	chr3	81055241	-	N	Y	Y	Y	Y	Y	transl_inter	na	NA	intergenic;ir	RP11-17A1.3,RP11-26	+,+	antisense,lir	71198;148309	ncRNA_intr	RP11-6B4.1	+	lincRNA	0
2764	chr11	47914462	-	chr3	195971404	-	N	Y	N	N	N	Y	transl_inter	na	NA	intergenic	NUP160,RP11-793I11.	-,-	protein_cod	44355;12413	intronic	PCYT1A	-	protein_cod	0
2765	chr11	47914577	-	chr3	195971412	-	N	N	N	N	N	Y	transl_inter	na	NA	intergenic	NUP160,RP11-793I11.	-,-	protein_cod	44470;12298	intronic	PCYT1A	-	protein_cod	0
2766	chr11	47914893	-	chr3	195971372	-	N	N	N	Y	N	N	transl_inter	na	NA	intergenic	NUP160,RP11-793I11.	-,-	protein_cod	44786;11982	intronic	PCYT1A	-	protein_cod	0

2767	chr11	69139814	-	chr3	128165155	-	N	N	N	Y	N	N	transl_inter	na	NA	intergenic	IFITM9P,RP11-211G23	-,+	pseudogene	68540;46417	intergenic	EEFSEC,DNAJB8	+,-	protein_cod	37670;16127
2768	chr11	98640894	-	chr3	56618171	-	N	Y	N	N	N	N	transl_inter	na	NA	intergenic	RP11-115E19.1,RP11-	+,-	lincRNA,linc	86429;168748	intronic	CCDC66	+	protein_cod	0
2769	chr11	98640944	-	chr3	56617885	-	N	N	N	N	Y	N	transl_inter	na	NA	intergenic	RP11-115E19.1,RP11-	+,-	lincRNA,linc	86479;168698	intronic	CCDC66	+	protein_cod	0
2770	chr11	101089882	-	chr3	32084103	-	N	Y	N	N	N	N	transl_inter	na	NA	intergenic	RP11-788M5.3,TRPC6	+,-	pseudogene	9560;232413	intergenic	SNORA25,Y_RNA	+,+	snoRNA,mis	4958;3262
2771	chr11	110801941	-	chr3	50022157	-	N	N	N	N	Y	N	transl_inter	na	NA	intergenic	RP11-97I14.1,RNA5SF	-,-	pseudogene	54040;108881	intronic	RBM6	+	protein_cod	0
2772	chr11	45399251	-	chr3	196318459	+	N	N	Y	N	N	N	transl_inter	na	NA	ncRNA_intr	RP11-430H10.2	+	lincRNA	0	intergenic;ir	FBXO45,LINC01063	+,-	protein_cod	2529;39910
2773	chr11	62609240	-	chr3	73160183	+	N	Y	Y	Y	Y	Y	transl_inter	na	NA	ncRNA_exc	RNU2-2P	-	snRNA	0	ncRNA_exc	RNU2-64P	+	snRNA	0
2774	chr11	36157463	+	chr3	51630116	-	N	Y	Y	Y	Y	N	transl_inter	na	NA	intronic	LDLRAD3	+	protein_cod	0	intronic	RAD54L2	+	protein_cod	0
2775	chr11	41510677	+	chr3	144991045	-	N	N	N	N	N	Y	transl_inter	na	NA	intergenic	LRRC4C,RP11-124G5	-,-	protein_cod	29354;29768	intergenic	RNA5SP144,RP11-622L	-,+	rRNA,pseuc	1085183;250280
2776	chr11	45399251	+	chr3	196318459	-	Y	N	N	N	N	N	transl_inter	na	NA	ncRNA_intr	RP11-430H10.2	+	lincRNA	0	intergenic;ir	FBXO45,LINC01063	+,-	protein_cod	2529;39910
2777	chr11	48363988	+	chr3	97919584	-	Y	N	N	N	N	N	transl_inter	na	NA	intergenic	OR4C3,OR4C4P	+,-	protein_cod	16481;2912	intergenic	OR5H5P,OR5H3P	+,+	pseudogene	2602;6683
2778	chr11	48364073	+	chr3	97919189	-	N	N	Y	N	N	N	transl_inter	na	NA	intergenic	OR4C3,OR4C4P	+,-	protein_cod	16566;2827	intergenic	OR5H5P,OR5H3P	+,+	pseudogene	2207;7078
2779	chr11	62609281	+	chr3	73160133	-	N	Y	Y	Y	Y	Y	transl_inter	na	NA	ncRNA_UTI	WDR74,RNU2-2P	-,-	protein_cod	0	upstream	RNU2-64P	+	snRNA	0
2780	chr11	23038137	+	chr3	81055241	+	N	Y	Y	Y	Y	Y	transl_inter	na	NA	intergenic;ir	RP11-17A1.3,RP11-26	+,+	antisense,lir	71198;148309	ncRNA_intr	RP11-6B4.1	+	lincRNA	0
2781	chr11	47640420	+	chr3	137254465	+	Y	Y	N	N	Y	N	transl_inter	na	NA	exonic	MTCH2	-	protein_cod	0	ncRNA_exc	RP11-575C1.1	-	pseudogene	0
2782	chr11	47640453	+	chr3	137254480	+	N	N	Y	N	N	N	transl_inter	na	NA	exonic	MTCH2	-	protein_cod	0	ncRNA_exc	RP11-575C1.1	-	pseudogene	0
2783	chr11	80835400	+	chr3	125585081	+	N	N	N	N	N	Y	transl_inter	na	NA	intergenic	RP11-170L9.1,RP11-6	+,+	pseudogene	166723;427868	ncRNA_intr	ENPP7P4,RP11-379B18.	+,-	pseudogene	0
2784	chr11	91777746	+	chr3	21769125	+	N	N	N	N	N	Y	transl_inter	na	NA	intergenic	TUBB4BP4,RPL7AP57	-,+	pseudogene	94546;116526	intronic	ZNF385D	-	protein_cod	0
2785	chr11	98640865	+	chr3	56617947	+	N	N	N	N	Y	N	transl_inter	na	NA	intergenic	RP11-115E19.1,RP11-	+,-	lincRNA,linc	86400;168777	intronic	CCDC66	+	protein_cod	0
2786	chr11	73777267	-	chr4	148181587	-	N	N	N	Y	N	N	transl_inter	na	NA	intronic	C2CD3	-	protein_cod	0	intergenic	RP11-292D4.3,MIR548G	+,-	lincRNA,miF	251290;84194
2787	chr11	101090232	+	chr4	70059023	-	N	N	Y	N	N	N	transl_inter	na	NA	intergenic	RP11-788M5.3,TRPC6	+,-	pseudogene	9910;232063	ncRNA_intr	RP11-704M14.1	+	antisense	0
2788	chr11	62232318	+	chr4	97727792	+	N	Y	N	Y	N	N	transl_inter	na	NA	intronic	AHNAK	-	protein_cod	0	ncRNA_intr	RP11-145G20.1	+	lincRNA	0
2789	chr11	88040149	+	chr4	187971474	+	N	Y	Y	N	N	Y	transl_inter	na	NA	intronic	CTSC	-	protein_cod	0	intergenic	RP11-217E13.1,RP11-69	-,+	lincRNA,linc	28347;9779
2790	chr11	39001735	-	chr5	152632712	-	N	N	Y	Y	N	Y	transl_inter	na	NA	intergenic	RP11-64I17.1,RP11-31	+,+	lincRNA,pse	305944;181268	intergenic	AC091969.1,GRIA1	-,+	lincRNA,pro	29609;237394
2791	chr11	40493160	-	chr5	63771939	-	N	Y	N	Y	Y	N	transl_inter	na	NA	intronic	LRRC4C	-	protein_cod	0	ncRNA_intr	AC016561.1	-	pseudogene	0
2792	chr11	102685813	-	chr5	87420970	+	N	N	Y	N	N	N	transl_inter	na	NA	ncRNA_intr	WTAPP1	+	pseudogene	0	intergenic	CTD-2316B1.2,TMEM16	-,-	lincRNA,pro	391884;64480
2793	chr11	102686290	-	chr5	87420923	+	Y	N	N	N	N	N	transl_inter	na	NA	ncRNA_intr	WTAPP1	+	pseudogene	0	intergenic	CTD-2316B1.2,TMEM16	-,-	lincRNA,pro	391837;64527
2794	chr11	76889781	+	chr5	180061849	-	N	N	N	Y	N	N	transl_inter	na	NA	intronic	MYO7A	+	protein_cod	0	intronic	FLT4	-	protein_cod	0
2795	chr11	40493095	+	chr5	63771996	+	Y	N	N	N	N	N	transl_inter	na	NA	intronic	LRRC4C	-	protein_cod	0	ncRNA_intr	AC016561.1	-	pseudogene	0
2796	chr11	56474806	-	chr6	139666818	-	N	Y	Y	N	N	N	transl_inter	na	NA	intergenic	OR9G1,RP11-100N3.2	+,-	protein_cod	6025;28986	intergenic	RP11-12A2.1,CITED2	+,-	pseudogene	6101;26575
2797	chr11	89992728	-	chr6	146528283	-	Y	N	N	N	Y	Y	transl_inter	na	NA	ncRNA_intr	DISC1FP1	+	processed_	0	intronic	GRM1	+	protein_cod	0
2798	chr11	30696488	-	chr6	51003201	+	N	N	N	N	N	Y	transl_inter	na	NA	intergenic	RP5-1024C24.1,DCDC	+,-	antisense,pi	44433;155428	intergenic	FTH1P5,RP3-437C15.2	-,-	pseudogene	122232;246879
2799	chr11	71863065	-	chr6	160656494	+	N	N	N	N	N	Y	transl_inter	na	NA	intergenic	FOLR3,RP11-807H22.	+,+	protein_cod	12129;6801	intronic;intrc	SLC22A2	-	protein_cod	0
2800	chr11	71863126	-	chr6	160656494	+	N	N	Y	N	N	N	transl_inter	na	NA	intergenic	FOLR3,RP11-807H22.	+,+	protein_cod	12190;6740	intronic;intrc	SLC22A2	-	protein_cod	0
2801	chr11	71863159	-	chr6	160656527	+	N	N	N	N	Y	N	transl_inter	na	NA	intergenic	FOLR3,RP11-807H22.	+,+	protein_cod	12223;6707	intronic	SLC22A2	-	protein_cod	0
2802	chr11	103792603	-	chr6	108906546	+	N	N	Y	N	N	N	transl_inter	na	NA	intronic;intrc	PDGFD	-	protein_cod	0	intronic;intrc	FOXO3	+	protein_cod	0
2803	chr11	129179381	-	chr6	66561417	+	N	N	Y	Y	N	N	transl_inter	na	NA	intergenic	ZNF123P,BARX2	-,+	pseudogene	13005;66454	intergenic;ir	ADH5P4,NUFIP1P	-,-	pseudogene	13470;241907
2804	chr11	129179413	-	chr6	66561417	+	N	Y	N	N	N	N	transl_inter	na	NA	intergenic	ZNF123P,BARX2	-,+	pseudogene	13037;66422	intergenic;ir	ADH5P4,NUFIP1P	-,-	pseudogene	13470;241907
2805	chr11	103792603	+	chr6	108906546	-	Y	N	Y	N	N	N	transl_inter	na	NA	intronic;intrc	PDGFD	-	protein_cod	0	intronic;intrc	FOXO3	+	protein_cod	0
2806	chr11	118293792	+	chr6	10762811	-	N	N	Y	N	Y	N	transl_inter	na	NA	intronic	ATP5L	+	protein_cod	0	intronic	TMEM14B,SYCP2L,RP1	+,+,+	protein_cod	0
2807	chr11	4420586	+	chr6	133938218	+	N	N	N	Y	N	N	transl_inter	na	NA	intergenic	TRIM21,OR51R1P	-,+	protein_cod	5660;18704	ncRNA_intr	RP3-323P13.2	-	antisense	0
2808	chr11	18522922	-	chr7	142930319	-	N	N	N	N	Y	N	transl_inter	na	NA	intronic	TSG101	-	protein_cod	0	intergenic	TAS2R40,GSTK1	+,+	protein_cod	10157;10867
2809	chr11	27655293	-	chr7	83089911	+	N	N	N	N	N	Y	transl_inter	na	NA	ncRNA_intr	BDNF-AS,LINC00678	+,-	antisense,lir	0	intronic	SEMA3E	-	protein_cod	0
2810	chr11	27655327	-	chr7	83090089	+	N	N	Y	N	N	N	transl_inter	na	NA	ncRNA_intr	BDNF-AS,LINC00678	+,-	antisense,lir	0	intronic	SEMA3E	-	protein_cod	0
2811	chr11	37072875	-	chr7	121298038	+	N	N	N	N	Y	N	transl_inter	na	NA	intergenic	CTD-2119L1.1,SNORA	+,-	pseudogene	353458;650800	intergenic	RP4-745K6.1,RN7SKP27	-,+	lincRNA,mis	7624;78459
2812	chr11	27655412	+	chr7	83089930	-	Y	N	N	N	N	N	transl_inter	na	NA	ncRNA_intr	BDNF-AS,LINC00678	+,-	antisense,lir	0	intronic	SEMA3E	-	protein_cod	0
2813	chr11	131230891	+	chr7	57922944	-	N	N	Y	N	N	N	transl_inter	na	NA	intergenic	AP002856.5,NTM	+,+	lincRNA,pro	10079;9482	intergenic	RP11-548K12.12,NONE	+,NONE	pseudogene	26192;NONE
2814	chr11	85211622	+	chr7	107829251	+	Y	Y	Y	Y	Y	Y	transl_inter	na	NA	intronic	DLG2	-	protein_cod	0	intronic	NRCAM	-	protein_cod	0
2815	chr11	103201267	+	chr7	32226014	+	N	Y	N	N	N	N	transl_inter	na	NA	intronic	DYNC2H1	+	protein_cod	0	intronic	PDE1C	-	protein_cod	0
2816	chr11	3560915	-	chr8	12474524	-	N	N	N	Y	N	N	transl_inter	na	NA	ncRNA_intr	RP13-726E6.2	-	processed_	0	intergenic	RP11-303G3.6,RPS3AP3	+,-	pseudogene	35952;11461
2817	chr11	48446710	-	chr8	135320972	-	Y	N	N	N	N	N	transl_inter	na	NA	intergenic	OR4C2P,OR4C10P	+,-	pseudogene	3885;7059	ncRNA_intr	RP11-513H8.1	-	lincRNA	0
2818	chr11	131127850	-	chr8	3099366	-	N	Y	N	N	N	N	transl_inter	na	NA	ncRNA_intr	AP002856.5	+	lincRNA	0	intronic	CSMD1	-	protein_cod	0
2819	chr11	134343365	-	chr8	5377762	-	Y	Y	Y	Y	Y	Y	transl_inter	na	NA	ncRNA_intr	RP11-627G23.1	+	processed_	0	intergenic	RP5-991O23.1,RP11-281	-,-	lincRNA,linc	11114;139439
2820	chr11	50758969	+	chr8	43767098	-	N	Y	N	N	N	N	transl_inter	na	NA	intergenic	RP11-574M7.2,AC110	+,-	pseudogene	377482;505040	intergenic	RP11-643N23.1,NONE	-,NONE	pseudogene	237364;NONE
2821	chr11	94489274	-	chr9	93051706	+	N	Y	N	N	N	N	transl_inter	na	NA	intronic	AMOTL1	+	protein_cod	0	intergenic	OR7E116P,RP11-389K14	+,-	pseudogene	56283;11478
2822	chr11	55826098	+	chr9	68418844	-	N	Y	Y	Y	N	N	transl_inter	na	NA	intergenic	OR5AQ1P,OR5J1P	+,+	pseudogene	3319;12408	intergenic;ir	MIR4477A,RP11-764K9.	+,-	miRNA,pseu	3456;9017
2823	chr11	62095038	+	chr9	68418844	-	N	Y	N	N	N	Y	transl_inter	na	NA	intergenic	SCGB1D4,NPM1P35	-,+	protein_cod	28502;3380	intergenic;ir	MIR4477A,RP11-764K9.	+,-	miRNA,pseu	3456;9017
2824	chr11	24393106	+	chr9	31877038	+	N	Y	N	N	Y	N	transl_inter	na	NA	intergenic	RP11-2F20.1,Y_RNA	-,+	lincRNA,mis	109342;84351	intergenic	HMGB3P23,RNA5SP281	-,+	pseudogene	231880;416518
2825	chr11	37456333	-	chrX	109143697	-	N	N	N	N	N	Y	transl_inter	na	NA	intergenic	CTD-2119L1.1,SNORA	+,-	pseudogene	736916;267342	intergenic	RPS5P7,TMEM164	+,+	pseudogene	46549;102166
2826	chr11	63660830	-	chrX	109628851	-	N	N	Y	N	N	N	transl_inter	na	NA	intronic;intrc	MARK2	+	protein_cod	0	intronic;intrc	AMMECR1,RGAG1	-,+	protein_cod	0
2827	chr11	89992728	-	chrX	50187785	-	N	N	Y	N	N	N	transl_inter	na	NA	ncRNA_intr	DISC1FP1	+	processed_	0	ncRNA_intr	DGKK	-	processed_	0
2828	chr11	93858679	-	chrX	27875652	+	N	N	N	Y	N	N	transl_inter	na	NA	intergenic	HEPHL1,PANX1	+,+	protein_cod	11762;3415	intergenic	RP11-12D5.4,RP11-12D	+,+	pseudogene	2182;6358
2829	chr11	109372720	-	chrX	116557962	+	Y	N	N	N	N	N	transl_inter	na	NA	ncRNA_intr	RP11-708B6.2	-	antisense	0	intergenic	RP3-378P9.2,KLHL13	+,-	pseudogene	23918;473814
2830	chr11	109372720	-	chrX	116557987	+	N	Y	N	N	N	N	transl_inter	na	NA	ncRNA_intr	RP11-708B6.2	-	antisense	0	intergenic	RP3-378P9.2,KLHL13	+,-	pseudogene	23943;473789
2831	chr11	114586213	-	chrX	148968893	+	N	N	N	Y	N	N	transl_inter	na	NA	intergenic	NXPE2,ACA59	+,-	protein_cod	6851;412725	intergenic	DUTP4,MAGEA8-AS1	+,-	pseudogene	69587;38743
2832	chr11	132898054	-	chrX	67872790	+	Y	N	N	N	N	N	transl_inter	na	NA	intronic	OPCML	-	protein_cod	0	intronic	STARD8	+	protein_cod	0
2833	chr11	42740511	+	chrX	13522399	-	N	N	N	N	N	Y	transl_inter	na	NA	intergenic;ir	CTD-2572N17.1,CTD-	-,+	lincRNA,pse	465249;220814	intergenic	RP11-1L9.1,EGFL6	+,+	lincRNA,pro	101261;65325
2834	chr11	63660830	+	chrX	10159335	+	N	N	Y	N	N	N	transl_inter	na	NA	intronic;intrc	MARK2	+	protein_cod	0	intronic;intrc	CLCN4	+	protein_cod	0
2835	chr11	63660830	+	chrX	109628851	+	Y	N	N	N	N	N	transl_inter	na	NA	intronic;intrc	MARK2	+	protein_cod	0	intronic;intrc	AMMECR1,RGAG1	-,+	protein_cod	0
2836	chr11	70835267	-	chrY	13204446	-	N	N	N	Y	N	N	transl_inter	na	NA	intronic	SHANK2	-	protein_cod	0	intergenic	NONE,RP1-85D24.3	NONE,-	NONE,pseu	NONE;58295
2837	chr11	70835641	-	chrY	13204536	-	N	N	N	N	Y	N	transl_inter	na	NA	intronic	SHANK2	-	protein_cod	0	intergenic	NONE,RP1-85D24.3	NONE,-	NONE,pseu	NONE;58205
2838	chr12	30403690	-	chr13	50037477	+	N	Y	N	N	N	N	transl_inter	na	NA	ncRNA_intr	RP11-776A13.3	-	lincRNA	0	intronic	SETDB2	+	protein_cod	0
2839	chr12	115032719	+	chr13	93190882	-	N	N	N	Y	N	N	transl_inter	na	NA	intergenic	OSTF1P1,RP11-162N	+,+	pseudogene	6112;26847	intronic	GPC5	+	protein_cod	0
2840	chr12	120025793	-	chr14	77735903	-	Y	N	N	N	N	N	transl_inter	na	NA	ncRNA_intr	RP11-768F21.1	-	lincRNA	0	intronic	NGB	-	protein_cod	0
2841	chr12	127642147	-	chr14	34913337	+	Y	Y	Y	Y	N	N	transl_inter	na	NA	intergenic	RP11-575F12.2,AC079	-,-	lincRNA,miF	11070;8469	intronic	SPTSSA	-	protein_cod	0
2842	chr12	23542313	+	chr14	88593846	-	N	Y	N	N	Y	N	transl_inter	na	NA	intergenic	RP11-153K16.2,SOX5	-,-	lincRNA,pro	137880;140127	intergenic	LINC01146,RP11-753D2	+,-	lincRNA,linc	30570;9231
2843	chr12	23542360	+	chr14	88593986	-	N	N	N	Y	N	N	transl_inter	na	NA	intergenic	RP11-153K16.2,SOX5	-,-	lincRNA,pro	137927;140080	intergenic	LINC01146,RP11-753D2	+,-	lincRNA,linc	30710;9091
2844	chr12	23542420	+	chr14	88593854	-	N	N	Y	N	N	Y	transl_inter	na	NA	intergenic	RP11-153K16.2,SOX5	-,-	lincRNA,pro	137987;140020	intergenic	LINC01146,RP11-753D2	+,-	lincRNA,linc	30578;9223
2845	chr12	55173727	+	chr14	88591684	-	Y	N	N	Y	N	Y	transl_inter	na	NA	intergenic	RP11-850F7.7,VDAC1	+,-	lincRNA,pse	92509;22803	intergenic	LINC01146,RP11-753D2	+,-	lincRNA,linc	28408;11393
2846	chr12	129123702	+	chr14	104698810	-	N	Y	N	N	N	Y	transl_inter	na	NA	intronic	TMEM132C	+	protein_cod	0	ncRNA_intr	RP11-260M19.2	+	lincRNA	0
2847	chr12	129123756	+	chr14	104698412	-	N	N	N	Y	N	N	transl_inter	na	NA	intronic	TMEM132C	+	protein_cod	0	ncRNA_intr	RP11-260M19.2	+	lincRNA	0
2848	chr12	95680678	+	chr15	40854180	+	Y	Y	Y	Y	N	Y	transl_inter	na	NA	intronic	VEZT	+	protein_cod	0	intronic;intrc	C15orf57	-	protein_cod	0
2849	chr12	112997536	-	chr16	5820333	-	N	N	Y	N	N	Y	transl_inter	na	NA	intergenic	PTPN11,RPH3A	+,+	protein_cod	49819;10648	ncRNA_intr	RP11-420N3.2	+	processed_	0
2850	chr12	96071648	-	chr17	41463978	-	Y	N	N	Y	Y	Y	transl_inter	na	NA	intronic	NTN4	-	protein_cod	0	ncRNA_exc	LINC00910	-	lincRNA	0

2851	chr12	1730083	-	chr17	36594925	+	N	Y	N	N	N	N	transl_inter	na	NA	intronic	WNT5B	+	protein_cod	0	intronic	ARHGAP23	+	protein_cod	0
2852	chr12	95262289	-	chr18	31513994	+	N	Y	N	N	N	N	transl_inter	na	NA	intergenic	KRT19P2,NDUFA12	+,-	pseudogene	33355;28542	intronic	NOL4	-	protein_cod	0
2853	chr12	127638174	-	chr19	15941560	-	N	N	Y	Y	Y	Y	transl_inter	na	NA	intergenic	RP11-575F12.2,AC079	-,-	lincRNA,miF	7097;12442	ncRNA_intro	UCA1,AC004510.3	+,-	processed_	0
2854	chr12	2792006	-	chr19	6718017	+	N	Y	N	Y	N	N	transl_inter	na	NA	ncRNA_intro	CACNA1C-AS1	-	antisense	0	intronic	C3	-	protein_cod	0
2855	chr12	25012826	-	chr19	42993930	+	N	N	N	N	Y	N	transl_inter	na	NA	intronic	BCAT1	-	protein_cod	0	ncRNA_intro	LIPE-AS1	+	antisense	0
2856	chr12	28226412	-	chr2	194083179	-	Y	N	Y	N	N	Y	transl_inter	na	NA	intergenic	PTHLH,CCDC91	-,+	protein_cod	100774;59770	intergenic	PCGEM1,AC092638.1	+,+	lincRNA,pse	441554;38029
2857	chr12	42499859	-	chr2	95401075	-	N	N	N	N	N	Y	transl_inter	na	NA	exonic	GXYLT1	-	protein_cod	0	ncRNA_exo	AC073464.4	-	pseudogene	0
2858	chr12	79565099	-	chr2	30731752	-	N	N	Y	N	N	N	transl_inter	na	NA	intronic	SYT1	+	protein_cod	0	intronic	LCLAT1	+	protein_cod	0
2859	chr12	127638154	-	chr2	41607088	-	N	N	Y	N	N	N	transl_inter	na	NA	intergenic	RP11-575F12.2,AC079	-,-	lincRNA,miF	7077;12462	intergenic	HNRNPA1P57,AC01073	+,+	pseudogene	222393;216438
2860	chr12	30729894	-	chr2	170609570	+	Y	N	Y	N	N	N	transl_inter	na	NA	intergenic	RP11-776A13.2,IPO8	+,-	pseudogene	84659;52028	intronic	KLHL23	+	protein_cod	0
2861	chr12	49509913	-	chr2	110394935	+	N	N	N	N	N	Y	transl_inter	na	NA	intergenic	LMBR1L,TUBA1B	-,-	protein_cod	5232;11652	intergenic	SOWAHC,AC011753.6	+,+	protein_cod	18372;27851
2862	chr12	119632776	+	chr2	167746375	-	N	Y	N	Y	Y	N	transl_inter	na	NA	ncRNA_exo	RP11-64B16.2	+	pseudogene	0	ncRNA_exo	AC092601.1	-	pseudogene	0
2863	chr12	51222873	+	chr2	214171095	+	N	N	Y	N	N	N	transl_inter	na	NA	intergenic	ATF1,RN7SL519P	+,-	protein_cod	7968;12410	intronic;intro	SPAG16	+	protein_cod	0
2864	chr12	130508235	+	chr2	72650298	+	N	N	N	N	Y	N	transl_inter	na	NA	downstream	RP11-474D1.4	-	lincRNA	0	intronic	EXOC6B	-	protein_cod	0
2865	chr12	34838519	-	chr20	26319569	-	N	N	Y	N	N	N	transl_inter	na	NA	intergenic	RP13-7D7.1,NONE	+,NONE	pseudogene	435626;NONE	intergenic	MIR663A,NONE	-,NONE	processed_	87407;NONE
2866	chr12	92038058	-	chr20	4427307	-	N	N	Y	Y	N	N	transl_inter	na	NA	intergenic	RP11-121E16.1,RP11-	+,+	lincRNA,linc	275675;35850	intergenic	RP11-307O10.1,RP5-116	-,+	lincRNA,linc	14987;28978
2867	chr12	110616564	-	chr20	13571339	-	N	Y	N	Y	Y	N	transl_inter	na	NA	intronic	IFT81	+	protein_cod	0	intronic	TASP1	-	protein_cod	0
2868	chr12	85009944	-	chr20	26210976	+	N	N	N	N	N	Y	transl_inter	na	NA	intergenic	RP11-228G3.1,RP11-7	-,+	pseudogene	166287;55433	ncRNA_intro	MIR663A	-	processed_	0
2869	chr12	85010632	-	chr20	26210875	+	N	N	Y	N	N	N	transl_inter	na	NA	intergenic	RP11-228G3.1,RP11-7	-,+	pseudogene	166975;54745	ncRNA_intro	MIR663A	-	processed_	0
2870	chr12	110617821	-	chr20	13571318	+	Y	Y	N	N	Y	N	transl_inter	na	NA	intronic	IFT81	+	protein_cod	0	intronic	TASP1	-	protein_cod	0
2871	chr12	7355771	+	chr20	41564381	-	N	N	N	Y	Y	N	transl_inter	na	NA	intronic	PEX5	+	protein_cod	0	intronic	PTPRT	-	protein_cod	0
2872	chr12	89238523	+	chr20	61263370	+	Y	Y	Y	Y	Y	Y	transl_inter	na	NA	intergenic	RNU1-117P,RP11-13A	+,-	snRNA,lincF	262950;165935	intergenic	WI2-87327B8.1,RP13-30	+,-	lincRNA,ant	88656;1663
2873	chr12	30310724	-	chr21	38654835	+	N	Y	N	N	N	Y	transl_inter	na	NA	intergenic	U3,RP11-776A13.1	+,+	snoRNA,linc	257784;43192	intergenic	AP001412.1,AP001437.1	+,-	antisense,a	14050;82944
2874	chr12	8594657	+	chr21	44375669	+	Y	Y	N	Y	N	Y	transl_inter	na	NA	intergenic	OR7E149P,CLEC6A	-,+	pseudogene	3636;13865	intergenic	MIR5692B,AP001630.5	-,-	miRNA,antis	4545;9080
2875	chr12	82340459	-	chr22	20062967	+	Y	Y	N	N	N	Y	transl_inter	na	NA	intergenic	RP11-275O18.1,RP11-	+,+	pseudogene	77634;7039	intergenic	AC006547.13,DGCR8	-,+	antisense,p	4901;4788
2876	chr12	82340460	+	chr22	20062965	-	Y	N	Y	N	Y	N	transl_inter	na	NA	intergenic	RP11-275O18.1,RP11-	+,+	pseudogene	77635;7038	intergenic	AC006547.13,DGCR8	-,+	antisense,p	4899;4790
2877	chr12	43917955	-	chr3	158575574	+	Y	N	N	N	N	N	transl_inter	na	NA	intronic	ADAMTS20	-	protein_cod	0	intergenic	GPR79,NONE	-,NONE	pseudogene	15701;NONE
2878	chr12	43917982	+	chr3	158576711	-	N	N	N	N	N	Y	transl_inter	na	NA	intronic	ADAMTS20	-	protein_cod	0	intergenic	GPR79,NONE	-,NONE	pseudogene	16838;NONE
2879	chr12	107606777	-	chr4	96749304	-	Y	N	N	N	N	N	transl_inter	na	NA	intergenic	RP11-797M17.1,SETP	+,+	antisense,p	89685;58394	intergenic	RPL30P6,PDHA2	-,+	pseudogene	182860;11935
2880	chr12	54478896	+	chr4	3636135	-	N	Y	Y	Y	Y	Y	transl_inter	na	NA	ncRNA_intro	RP11-834C11.3,RP11-	+,+	lincRNA,linc	0	downstream	RP3-368B9.2	+	lincRNA	0
2881	chr12	53542035	-	chr5	38027137	-	N	N	Y	N	N	N	transl_inter	na	NA	intergenic	HIGD1AP1,RP11-1136	+,+	pseudogene	5924;5021	ncRNA_intro	CTD-2116N24.1	+	lincRNA	0
2882	chr12	127638186	-	chr5	8416110	-	Y	N	Y	Y	Y	N	transl_inter	na	NA	intergenic	RP11-575F12.2,AC079	-,-	lincRNA,miF	7109;12430	ncRNA_intro	RP11-480D4.1,RP11-480	-,-	lincRNA,linc	0
2883	chr12	127638193	-	chr5	8416181	-	N	Y	N	N	N	N	transl_inter	na	NA	intergenic	RP11-575F12.2,AC079	-,-	lincRNA,miF	7116;12423	ncRNA_intro	RP11-480D4.1,RP11-480	-,-	lincRNA,linc	0
2884	chr12	14504079	-	chr5	13827738	+	N	N	N	N	Y	N	transl_inter	na	NA	intergenic	RN7SL676P,RPL30P1	+,-	misc_RNA,p	39530;5697	intronic	DNAH5	-	protein_cod	0
2885	chr12	14504176	-	chr5	13827578	+	N	Y	N	Y	N	N	transl_inter	na	NA	intergenic	RN7SL676P,RPL30P1	+,-	misc_RNA,p	39627;5600	intronic	DNAH5	-	protein_cod	0
2886	chr12	118926224	-	chr5	103926648	+	N	Y	Y	Y	Y	Y	transl_inter	na	NA	intergenic	SUDS3,RP11-121J20.	+,-	protein_cod	70384;156896	ncRNA_intro	RP11-6N13.1	-	lincRNA	0
2887	chr12	70597580	+	chr5	78426550	+	N	N	N	N	Y	N	transl_inter	na	NA	ncRNA_intro	RP11-611E13.2	-	antisense	0	intronic	BHMT	+	protein_cod	0
2888	chr12	130539339	-	chr6	166501739	+	N	Y	N	N	N	N	transl_inter	na	NA	intergenic	RP11-474D1.1,RP11-4	+,+	lincRNA,linc	6418;15531	intergenic	GAPDHP72,SNORD45	+,+	pseudogene	22951;11414
2889	chr12	130539658	-	chr6	166501878	+	N	N	N	Y	N	N	transl_inter	na	NA	intergenic	RP11-474D1.1,RP11-4	+,+	lincRNA,linc	6737;15212	intergenic	GAPDHP72,SNORD45	+,+	pseudogene	23090;11275
2890	chr12	19940304	+	chr6	131013597	-	N	N	N	Y	Y	N	transl_inter	na	NA	ncRNA_intro	RP11-405A12.2	+	lincRNA	0	intergenic;ir	Y_RNA,RP11-102N11.1	-,+	misc_RNA,l	118340;4860
2891	chr12	25230261	+	chr6	94729059	-	N	N	N	N	N	Y	transl_inter	na	NA	intronic	LRMP	+	protein_cod	0	intergenic	RP11-524K14.1,RP3-463	+,+	lincRNA,linc	129931;144579
2892	chr12	25230273	+	chr6	94729036	-	N	N	N	N	Y	N	transl_inter	na	NA	intronic	LRMP	+	protein_cod	0	intergenic	RP11-524K14.1,RP3-463	+,+	lincRNA,linc	129908;144602
2893	chr12	72102024	+	chr6	119715833	+	N	N	N	N	Y	N	transl_inter	na	NA	downstream	RP11-498M15.1	-	lincRNA	0	intergenic	MAN1A1,MIR3144	-,+	protein_cod	44907;620492
2894	chr12	2866250	-	chr7	71170105	+	Y	N	N	N	Y	N	transl_inter	na	NA	ncRNA_intro	RP11-885B4.1	-	lincRNA	0	intronic	WBSCR17	+	protein_cod	0
2895	chr12	43292977	-	chr7	64882870	+	N	N	Y	N	N	N	transl_inter	na	NA	intergenic	RP11-25I15.3,RP11-51	+,-	lincRNA,pse	182324;30674	intergenic	ZNF92,RP11-667F9.2	+,+	protein_cod	16832;45114
2896	chr12	106170202	-	chr7	8343375	+	N	Y	N	N	N	N	transl_inter	na	NA	intergenic	CASC18,ST13P3	+,+	lincRNA,pse	32362;239473	ncRNA_exo	RP4-594A5.1	+	lincRNA	0
2897	chr12	119145698	-	chr7	44294374	+	N	N	N	N	Y	N	transl_inter	na	NA	intergenic	RP11-121J20.1,RPL17	-,-	lincRNA,pse	59169;30071	intronic	CAMK2B	-	protein_cod	0
2898	chr12	132926076	-	chr7	628861	+	N	N	N	N	N	Y	transl_inter	na	NA	intergenic	RP13-895J2.7,RP11-5	+,+	antisense,lir	17915;107670	intronic	PRKAR1B	-	protein_cod	0
2899	chr12	132926185	-	chr7	628448	+	N	Y	N	N	N	N	transl_inter	na	NA	intergenic	RP13-895J2.7,RP11-5	+,+	antisense,lir	18024;107561	intronic	PRKAR1B	-	protein_cod	0
2900	chr12	132926206	-	chr7	628784	+	N	N	Y	N	N	N	transl_inter	na	NA	intergenic	RP13-895J2.7,RP11-5	+,+	antisense,lir	18045;107540	intronic	PRKAR1B	-	protein_cod	0
2901	chr12	132926310	-	chr7	628481	+	Y	N	N	N	N	N	transl_inter	na	NA	intergenic	RP13-895J2.7,RP11-5	+,+	antisense,lir	18149;107436	intronic	PRKAR1B	-	protein_cod	0
2902	chr12	38020321	+	chr7	58022009	-	N	Y	N	N	N	N	transl_inter	na	NA	intergenic	RP11-125N22.2,RP11-	+,-	pseudogene	50135;452010	intergenic	RP11-548K12.12,NONE	+,NONE	pseudogene	125257;NONE
2903	chr12	43292291	+	chr7	64883123	-	Y	N	N	N	N	N	transl_inter	na	NA	intergenic	RP11-25I15.3,RP11-51	+,-	lincRNA,pse	181638;31360	intergenic	ZNF92,RP11-667F9.2	+,+	protein_cod	17085;44861
2904	chr12	43292442	+	chr7	64882991	-	N	N	N	N	Y	N	transl_inter	na	NA	intergenic	RP11-25I15.3,RP11-51	+,-	lincRNA,pse	181789;31209	intergenic	ZNF92,RP11-667F9.2	+,+	protein_cod	16953;44993
2905	chr12	43292498	+	chr7	64882873	-	N	N	Y	N	N	N	transl_inter	na	NA	intergenic	RP11-25I15.3,RP11-51	+,-	lincRNA,pse	181845;31153	intergenic	ZNF92,RP11-667F9.2	+,+	protein_cod	16835;45111
2906	chr12	30403679	+	chr7	143686161	+	N	N	N	N	N	Y	transl_inter	na	NA	ncRNA_intro	RP11-776A13.3	-	lincRNA	0	ncRNA_intro	RP4-669B10.3	+	lincRNA	0
2907	chr12	33365457	+	chr7	146035843	+	N	N	N	Y	N	N	transl_inter	na	NA	intergenic	AC026357.1,SNORD1	-,-	miRNA,snol	170702;150061	intronic;intro	CNTNAP2	+	protein_cod	0
2908	chr12	42477173	-	chr8	14346419	-	N	N	Y	Y	Y	Y	transl_inter	na	NA	UTR3	GXYLT1	-	protein_cod	0	intronic	SGCZ	-	protein_cod	0
2909	chr12	47917987	-	chr8	120997933	-	N	N	N	Y	N	Y	transl_inter	na	NA	intergenic	RP1-90J4.3,RP3-432I1	+,+	pseudogene	38482;69004	intronic	DEPTOR	+	protein_cod	0
2910	chr12	1730087	-	chr8	128860636	+	N	N	N	N	N	Y	transl_inter	na	NA	intronic	WNT5B	+	protein_cod	0	ncRNA_intro	PVT1	+	processed_	0
2911	chr12	129621140	-	chr8	142837476	+	N	Y	Y	N	N	N	transl_inter	na	NA	intronic	TMEM132D	-	protein_cod	0	intergenic	AC138647.1,AC104417.1	+,+	protein_cod	308639;189384
2912	chr12	129621181	-	chr8	142837489	+	N	N	N	Y	N	N	transl_inter	na	NA	intronic	TMEM132D	-	protein_cod	0	intergenic	AC138647.1,AC104417.1	+,+	protein_cod	308652;189371
2913	chr12	64217262	+	chr8	101719008	-	N	Y	N	N	N	N	transl_inter	na	NA	ncRNA_exo	PABPC1P4	+	pseudogene	0	exonic	PABPC1	-	protein_cod	0
2914	chr12	25013035	+	chr8	11284031	+	N	N	Y	Y	N	Y	transl_inter	na	NA	intronic	BCAT1	-	protein_cod	0	intronic	FAM167A,C8orf12	-,+	protein_cod	0
2915	chr12	30403690	-	chr9	123567153	+	Y	N	N	Y	Y	Y	transl_inter	na	NA	ncRNA_intro	RP11-776A13.3	-	lincRNA	0	intergenic	RP11-27I1.4,RP11-27I1.6	+,-	pseudogene	5305;7600
2916	chr12	54485336	-	chr9	44889312	+	N	Y	N	N	N	Y	transl_inter	na	NA	ncRNA_intro	RP11-834C11.3,RP11-	+,+,-	lincRNA,linc	0	intergenic	RP11-160N1.8,FAM27C	+,+	pseudogene	15086;101002
2917	chr12	92760940	-	chr9	74645249	+	Y	N	N	N	N	N	transl_inter	na	NA	intergenic	RP11-796E2.4,CLLU1	+,-	antisense,p	177504;52930	intergenic	HSPB1P1,C9orf57	-,-	pseudogene	22197;21043
2918	chr12	10732293	-	chrX	73872856	+	N	Y	Y	Y	Y	Y	transl_inter	na	NA	intergenic	RP11-291B21.2,KLRAI	+,-	lincRNA,pse	21645;8784	intergenic	RLIM,RNU6-330P	-,+	protein_cod	38404;27032
2919	chr12	67811304	+	chrX	52890700	+	N	N	Y	N	N	N	transl_inter	na	NA	intergenic	MRPL40P1,NTAN1P3	+,-	pseudogene	65485;6748	downstream	XAGE3	-	protein_cod	0
2920	chr12	34333209	-	chrY	9929859	+	N	Y	N	N	N	N	transl_inter	na	NA	intergenic	TUBB8P4,RP11-313F2	-,-	pseudogene	13320;10763	downstream	RNA5SP519	-	rRNA	0
2921	chr13	41974494	-	chr14	78036100	+	N	N	Y	N	N	N	transl_inter	na	NA	intergenic	TUBBP2,OR7E36P	-,-	pseudogene	15175;30931	intronic	SPTLC2	-	protein_cod	0
2922	chr13	37855525	+	chr14	90912038	-	Y	Y	Y	Y	Y	Y	transl_inter	na	NA	intergenic	RPS12P24,LINC0104	-,-	pseudogene	60142;200079	intergenic;ir	CALM1,RP11-1078H9.1	+,-	protein_cod	37433;6369
2923	chr13	38371148	+	chr14	53814722	+	N	N	Y	N	N	N	transl_inter	na	NA	intronic	TRPC4	-	protein_cod	0	ncRNA_intro	AL163953.3	+	lincRNA	0
2924	chr13	38371159	+	chr14	53814700	+	Y	N	N	N	Y	N	transl_inter	na	NA	intronic	TRPC4	-	protein_cod	0	ncRNA_intro	AL163953.3	+	lincRNA	0
2925	chr13	44069827	-	chr15	41851396	+	N	Y	Y	Y	N	N	transl_inter	na	NA	intronic	ENOX1	-	protein_cod	0	UTR5	TYRO3	+	protein_cod	0
2926	chr13	44069831	+	chr15	41871536	-	N	Y	Y	N	N	N	transl_inter	na	NA	intronic	ENOX1	-	protein_cod	0	downstream	TYRO3	+	protein_cod	0
2927	chr13	44069838	+	chr15	41871536	-	N	N	N	Y	N	N	transl_inter	na	NA	intronic;intro	ENOX1	-	protein_cod	0	downstream	TYRO3	+	protein_cod	0
2928	chr13	92918997	+	chr15	23707570	+	Y	Y	Y	Y	N	Y	transl_inter	na	NA	intronic	GPC5	+	protein_cod	0	intergenic	GOLGA6L2,MIR4508	-,-	protein_cod	15189;99639
2929	chr13	52931617	+	chr16	27953707	-	N	N	Y	N	N	N	transl_inter	na	NA	intergenic	RP13-444H2.1,THSD1	+,-	lincRNA,pro	15586;19688	intronic	GSG1L	-	protein_cod	0
2930	chr13	97722135	+	chr16	69225316	+	N	Y	Y	Y	Y	N	transl_inter	na	NA	intergenic;ir	snoU13,LINC00456	+,-	snoRNA,linc	12401;102588	intronic;intro	SNTB2	+	protein_cod	0
2931	chr13	64825866	-	chr17	64174882	-	N	N	Y	Y	Y	N	transl_inter	na	NA	intergenic	LINC00355,LGMNP1	-,-	lincRNA,pse	175722;706363	intronic	CEP112	-	protein_cod	0
2932	chr13	97228634	+	chr17	16181932	-	N	N	N	N	Y	N	transl_inter	na	NA	intronic	HS6ST3	+	protein_cod	0	intronic	PIGL	+	protein_cod	0
2933	chr13	98716318	+	chr17	80596095	-	Y	Y	N	N	N	N	transl_inter	na	NA	intergenic	IPO5,FARP1	+,+	protein_cod	39767;78498	intronic	WDR45B	-	protein_cod	0
2934	chr13	115108156	-	chr18	78017157	-	Y	Y	N	Y	N	Y	transl_inter	na	NA	intergenic	LINC01054,NONE	-,NONE	antisense,N	8733;NONE	intergenic	PARD6G,NONE	-,NONE	protein_cod	11728;NONE

2935	chr13	29393966	+	chr18	20032162	-	N	N	N	N	Y	N	transl_inter	na	NA	intergenic	POM121L13P,MTUS2	+,+	pseudogene	40892;204782	intergenic	RP11-863N1.4,RPS4XP1	-,+	pseudogene	32905;134266
2936	chr13	63638953	-	chr2	207822080	-	Y	Y	Y	Y	Y	Y	transl_inter	na	NA	intergenic	LINC00448,LINC0037(	-,-	lincRNA,linc	332461;118281	intronic;intro	CPO	+	protein_cod	0
2937	chr13	37518195	-	chr2	14414603	+	N	N	N	N	N	Y	transl_inter	na	NA	intergenic	EIF4A1P5,ALG5	-,-	pseudogene	4564;5717	ncRNA_intro	LINC00276	-	lincRNA	0
2938	chr13	63638950	+	chr2	207822080	+	N	N	Y	N	N	N	transl_inter	na	NA	intergenic	LINC00448,LINC0037(	-,-	lincRNA,linc	332458;118284	intronic;intro	CPO	+	protein_cod	0
2939	chr13	79161382	+	chr2	133034109	+	Y	N	N	N	N	N	transl_inter	na	NA	ncRNA_intro	RNF219-AS1	+	antisense	0	intergenic	CDC27P1,AC097532.2	+,-	pseudogene	13494;9259
2940	chr13	48850354	-	chr20	21918625	-	Y	N	N	N	N	N	transl_inter	na	NA	intergenic	ITM2B,LINC00441	+,-	protein_cod	13291;20295	intergenic	RPL41P1,RP5-1185K9.1	+,+	pseudogene	182454;9660
2941	chr13	63649022	+	chr20	26149746	-	Y	Y	Y	Y	Y	Y	transl_inter	na	NA	intergenic	LINC00448,LINC0037(	-,-	lincRNA,linc	342530;108212	intergenic	RP13-329D4.3,MIR663A	-,-	lincRNA,pro	34912;17810
2942	chr13	19477052	-	chr21	14890400	+	N	N	Y	N	N	N	transl_inter	na	NA	intergenic	SNX19P2,LINC00408	-,+	pseudogene	7682;2527	intergenic	GTF2IP2,SNX19P1	-,+	pseudogene	28903;7520
2943	chr13	100589245	-	chr21	34846963	+	N	N	N	N	Y	N	transl_inter	na	NA	intergenic	CLYBL,ZIC5	+,-	protein_cod	39858;25973	intronic	TMEM50B,IFNGR2	-,+	protein_cod	0
2944	chr13	47089550	-	chr3	98555608	-	N	N	N	N	N	Y	transl_inter	na	NA	intergenic	FKBP1AP3,LRCH1	+,+	pseudogene	9979;37753	intronic	DCBLD2	-	protein_cod	0
2945	chr13	47089576	-	chr3	98556019	-	N	N	Y	N	N	N	transl_inter	na	NA	intergenic	FKBP1AP3,LRCH1	+,+	pseudogene	10005;37727	intronic	DCBLD2	-	protein_cod	0
2946	chr13	47089691	-	chr3	98555914	-	Y	N	N	N	N	N	transl_inter	na	NA	intergenic	FKBP1AP3,LRCH1	+,+	pseudogene	10120;37612	intronic	DCBLD2	-	protein_cod	0
2947	chr13	25670680	-	chr3	155027208	+	Y	N	N	N	N	N	transl_inter	na	NA	exonic	PABPC3	+	protein_cod	0	downstream	PABPC1P10	-	pseudogene	0
2948	chr13	49635688	-	chr3	65841633	+	Y	N	N	N	N	N	transl_inter	na	NA	intronic	FNDC3A	+	protein_cod	0	intronic	MAGI1	-	protein_cod	0
2949	chr13	49635728	-	chr3	65841277	+	N	N	Y	N	Y	N	transl_inter	na	NA	intronic	FNDC3A	+	protein_cod	0	intronic	MAGI1	-	protein_cod	0
2950	chr13	58340088	-	chr3	114472229	+	N	Y	N	N	N	Y	transl_inter	na	NA	intergenic	PCDH17,RNA5SP30	+,+	protein_cod	36643;225396	intronic	ZBTB20	-	protein_cod	0
2951	chr13	49892578	+	chr4	105191283	-	N	N	Y	Y	N	N	transl_inter	na	NA	intronic	CAB39L	-	protein_cod	0	ncRNA_intro	RP11-729M20.1	+	lincRNA	0
2952	chr13	51703922	+	chr4	8361687	+	N	N	N	Y	N	N	transl_inter	na	NA	ncRNA_intro	LINC00371	-	lincRNA	0	intergenic	RP11-774O3.3,ACOX3	-,-	lincRNA,pro	1622;6322
2953	chr13	82367698	-	chr5	141456960	-	Y	Y	Y	Y	Y	Y	transl_inter	na	NA	intergenic	PTMAP5,GYG1P2	+,+	pseudogene	102491;1029568	intergenic	AC005740.4,NDFIP1	-,+	pseudogene	40270;31110
2954	chr13	111396065	+	chr5	13827596	-	N	N	N	N	Y	N	transl_inter	na	NA	intergenic	ING1,RPL21P107	+,-	protein_cod	22644;18956	intronic	DNAH5	-	protein_cod	0
2955	chr13	19793618	+	chr6	57603564	+	N	N	Y	N	N	N	transl_inter	na	NA	ncRNA_intro	PSPC1P1	-	pseudogene	0	intergenic	PRIM2,GAPDHP41	+,-	protein_cod	90189;83405
2956	chr13	40880223	-	chr7	46563233	+	N	N	N	N	N	Y	transl_inter	na	NA	intergenic;ir	RNY3P9,RP11-172E9.	+,-	misc_RNA,l	79154;37871	intergenic	AC004869.2,HMGN1P19	-,+	lincRNA,pse	42057;110979
2957	chr13	40880223	-	chr7	46563336	+	N	Y	N	N	N	N	transl_inter	na	NA	intergenic;ir	RNY3P9,RP11-172E9.	+,-	misc_RNA,l	79154;37871	intergenic	AC004869.2,HMGN1P19	-,+	lincRNA,pse	42160;110876
2958	chr13	114212471	-	chr7	156374779	+	N	N	N	N	N	Y	transl_inter	na	NA	intergenic	TMCO3,TFDP1	+,+	protein_cod	7929;26542	ncRNA_intro	LINC01006	-	processed_	0
2959	chr13	63637902	+	chr7	57606290	+	N	N	Y	Y	N	N	transl_inter	na	NA	intergenic;ir	LINC00448,LINC0037(	-,-	lincRNA,linc	331410;119332	intergenic;ir	RP11-1324A7.2,NCOR1F	+,+	pseudogene	60689;53210
2960	chr13	84108455	-	chr8	64237693	-	N	Y	Y	Y	N	N	transl_inter	na	NA	intergenic	RNU6-67P,SLITRK1	-,-	snRNA,prot	236153;342889	intergenic	RN7SL135P,CTD-3046C	-,-	misc_RNA,l	90224;59704
2961	chr13	108539741	-	chr8	116021661	-	N	N	Y	N	N	N	transl_inter	na	NA	intergenic	FAM155A,LIG4	-,-	protein_cod	20658;320046	intergenic	RP11-192P9.1,TRPS1	-,-	pseudogene	217503;399063
2962	chr13	68823792	-	chr8	6239912	+	N	N	Y	N	N	N	transl_inter	na	NA	intergenic;ir	OR7E33P,ELL2P3	+,-	pseudogene	337712;40019	intergenic;ir	RP11-124B13.1,RP11-11	-,-	lincRNA,ant	124854;21160
2963	chr13	22171351	+	chr8	36161658	-	Y	N	N	N	N	N	transl_inter	na	NA	intronic	MICU2	-	protein_cod	0	intergenic	RP11-593P24.3,RP11-13	-,+	pseudogene	24444;4105
2964	chr13	25671493	+	chr8	101721664	-	N	N	Y	N	N	N	transl_inter	na	NA	exonic	PABPC3	+	protein_cod	0	intronic	PABPC1	-	protein_cod	0
2965	chr13	68823792	+	chr8	6239912	-	Y	Y	N	N	N	N	transl_inter	na	NA	intergenic;ir	OR7E33P,ELL2P3	+,-	pseudogene	337712;40019	intergenic;ir	RP11-124B13.1,RP11-11	-,-	lincRNA,ant	124854;21160
2966	chr13	19298091	-	chr9	66342044	-	N	N	Y	N	N	N	transl_inter	na	NA	downstream	GXYLT1P1	-	pseudogene	0	downstream	RP11-459O16.1	-	pseudogene	0
2967	chr13	110875124	-	chr9	68434050	-	Y	N	N	N	N	Y	transl_inter	na	NA	intronic	COL4A1	-	protein_cod	0	ncRNA_intro	RP11-764K9.4	-	pseudogene	0
2968	chr13	19642492	+	chr9	96387045	-	N	Y	N	N	N	N	transl_inter	na	NA	intergenic	PHF2P2,RNA5SP24	-,+	pseudogene	5634;20135	intronic	PHF2	+	protein_cod	0
2969	chr13	60825785	+	chr9	30107143	-	N	N	N	N	Y	N	transl_inter	na	NA	intergenic	LINC00434,TARDBPP.	-,+	lincRNA,pse	36705;23125	intergenic	RP11-460C6.1,snoU13	-,-	pseudogene	280434;71330
2970	chr13	114639942	+	chr9	15138483	+	N	N	Y	Y	Y	N	transl_inter	na	NA	intergenic	LINC00565,RASA3	-,-	lincRNA,pro	8125;107252	ncRNA_intro	RP11-54D18.3	-	pseudogene	0
2971	chr13	91149407	-	chrX	43448483	+	Y	N	N	N	N	N	transl_inter	na	NA	ncRNA_intro	LINC01049	+	lincRNA	0	intergenic;ir	NANOGP10,MAOA	-,+	pseudogene	180675;66984
2972	chr13	91149407	+	chrX	43448483	-	N	N	N	Y	N	N	transl_inter	na	NA	ncRNA_intro	LINC01049	+	lincRNA	0	intergenic;ir	NANOGP10,MAOA	-,+	pseudogene	180675;66984
2973	chr13	21816779	-	chrY	9951887	-	Y	Y	Y	Y	Y	Y	transl_inter	na	NA	intergenic	LINC01046,ESRRAP2	+,+	lincRNA,pse	7436;17375	intergenic	RNA5SP519,AC006987.(	-,-	rRNA,pseuc	21285;55510
2974	chr14	106771832	-	chr15	22491488	-	Y	N	N	N	Y	N	transl_inter	na	NA	upstream	IGHVII-26-2	-	IG_V_pseuc	0	upstream	RP11-603B24.6	-	pseudogene	0
2975	chr14	89177671	-	chr15	29945746	+	N	N	N	N	Y	N	transl_inter	na	NA	intronic	EML5	-	protein_cod	0	intergenic	TUBBP8,RP11-680F8.1	+,+	pseudogene	39268;21448
2976	chr14	77278458	+	chr15	87836923	+	N	N	N	N	N	Y	transl_inter	na	NA	intronic;intro	ANGEL1	-	protein_cod	0	intergenic;ir	RP11-138H10.2,RP11-64	+,-	lincRNA,linc	246531;138366
2977	chr14	106484225	+	chr15	22486810	+	Y	Y	Y	Y	Y	Y	transl_inter	na	NA	intergenic	IGHV4-4,IGHV2-5	-,-	IG_V_gene,	5622;9910	intergenic	IGHV1OR15-4,RP11-603	-,-	IG_V_pseuc	3542;3533
2978	chr14	106484416	+	chr15	22479842	+	N	N	N	N	Y	Y	transl_inter	na	NA	intergenic	IGHV4-4,IGHV2-5	-,-	IG_V_gene,	5813;9719	intergenic	IGHV4OR15-8,IGHV1OR	-,-	IG_V_gene,	6489;2994
2979	chr14	24322908	+	chr16	25926251	-	N	N	N	Y	N	Y	transl_inter	na	NA	intergenic	RP11-388E23.3,LINC0	-,-	pseudogene	26303;68548	intronic	HS3ST4	+	protein_cod	0
2980	chr14	37769602	+	chr16	61079285	-	N	Y	Y	Y	Y	N	transl_inter	na	NA	intronic;intro	MIPOL1	+	protein_cod	0	intergenic;ir	RP11-90K18.1,RP11-51C	-,-	pseudogene	116840;10018
2981	chr14	20975808	+	chr17	19941644	-	Y	Y	N	N	Y	N	transl_inter	na	NA	intronic	RNASE10	+	protein_cod	0	intronic	SPECC1	+	protein_cod	0
2982	chr14	20976100	+	chr17	19941642	-	N	N	N	N	N	Y	transl_inter	na	NA	intronic	RNASE10	+	protein_cod	0	intronic	SPECC1	+	protein_cod	0
2983	chr14	67082974	+	chr17	54137347	+	Y	Y	Y	N	N	N	transl_inter	na	NA	intronic	GPHN	+	protein_cod	0	intergenic	AC011073.1,ANKFN1	+,+	miRNA,prot	5100;93489
2984	chr14	91982026	+	chr17	28483268	+	Y	Y	Y	Y	Y	Y	transl_inter	na	NA	intergenic	SMEK1,AL133373.1	-,-	protein_cod	5128;56762	intronic	NSRP1	+	protein_cod	0
2985	chr14	103363409	+	chr17	71709833	+	N	Y	N	N	N	N	transl_inter	na	NA	intronic	TRAF3	+	protein_cod	0	intergenic;ir	RP11-277J6.3,CTD-2532	-,+	lincRNA,linc	49846;24160
2986	chr14	24322569	-	chr18	5368992	-	N	N	N	Y	Y	Y	transl_inter	na	NA	intergenic	RP11-388E23.3,LINC0	-,-	pseudogene	25964;68887	intergenic	CTD-3096M3.1,CTD-309	+,-	lincRNA,linc	51329;12648
2987	chr14	64066317	-	chr18	13152439	-	N	Y	N	N	N	N	transl_inter	na	NA	exonic	WDR89	-	protein_cod	0	ncRNA_exo	RP11-807E13.2	-	pseudogene	0
2988	chr14	73570603	-	chr18	66682540	+	N	N	N	Y	N	N	transl_inter	na	NA	intronic	RBM25	+	protein_cod	0	intronic	CCDC102B	+	protein_cod	0
2989	chr14	92100802	+	chr18	62150019	-	Y	N	Y	N	N	N	transl_inter	na	NA	intronic	CATSPERB	-	protein_cod	0	intergenic	RP11-146N18.1,RNU7-1	+,-	lincRNA,snF	59183;66671
2990	chr14	92101053	+	chr18	62149857	-	N	N	N	N	N	Y	transl_inter	na	NA	intronic	CATSPERB	-	protein_cod	0	intergenic	RP11-146N18.1,RNU7-1	+,-	lincRNA,snF	59021;66833
2991	chr14	24322571	+	chr18	5368990	+	Y	N	N	N	Y	N	transl_inter	na	NA	intergenic	RP11-388E23.3,LINC0	-,-	pseudogene	25966;68885	intergenic	CTD-3096M3.1,CTD-309	+,-	lincRNA,linc	51327;12650
2992	chr14	77736124	+	chr18	46843581	+	N	N	N	Y	N	N	transl_inter	na	NA	intronic	NGB	-	protein_cod	0	intronic	DYM	-	protein_cod	0
2993	chr14	34516674	-	chr19	31150120	-	N	N	N	N	Y	N	transl_inter	na	NA	ncRNA_intro	EGLN3-AS1	+	antisense	0	intronic	ZNF536	+	protein_cod	0
2994	chr14	67968123	-	chr19	4965591	-	N	Y	Y	N	N	N	transl_inter	na	NA	intronic	TMEM229B	-	protein_cod	0	intergenic	UHRF1,KDM4B	+,+	processed_	3426;3534
2995	chr14	67968215	-	chr19	4965573	-	Y	N	N	N	N	N	transl_inter	na	NA	intronic;intro	TMEM229B	-	protein_cod	0	intergenic;ir	UHRF1,KDM4B	+,+	processed_	3408;3552
2996	chr14	19494133	-	chr19	19632141	+	Y	Y	Y	Y	Y	N	transl_inter	na	NA	ncRNA_intro	NF1P4	-	pseudogene	0	intronic;intro	NDUFA13,YJEFN3,CTC-	+,+,+	protein_cod	0
2997	chr14	67968215	+	chr19	4965573	+	N	Y	N	N	N	N	transl_inter	na	NA	intronic;intro	TMEM229B	-	protein_cod	0	intergenic;ir	UHRF1,KDM4B	+,+	processed_	3408;3552
2998	chr14	102149809	-	chr2	237615426	-	N	Y	N	N	N	N	transl_inter	na	NA	ncRNA_intro	RP11-1029J19.5	-	lincRNA	0	intergenic	ACKR3,AC011286.1	+,+	protein_cod	124425;26599
2999	chr14	22259178	-	chr2	22966673	+	N	N	N	N	N	Y	transl_inter	na	NA	intergenic	TRAV7,TRAV8-1	+,+	TR_V_gene	7458;6281	intergenic	AC096570.1,RN7SKP27	+,+	lincRNA,mis	205513;117751
3000	chr14	22259141	+	chr2	22967243	-	N	N	N	Y	N	N	transl_inter	na	NA	intergenic	TRAV7,TRAV8-1	+,+	TR_V_gene	7421;6318	intergenic	AC096570.1,RN7SKP27	+,+	lincRNA,mis	206083;117181
3001	chr14	71376743	-	chr20	19643818	-	N	Y	N	N	N	N	transl_inter	na	NA	intronic	PCNX	+	protein_cod	0	intronic	SLC24A3	+	protein_cod	0
3002	chr14	104765460	-	chr20	36823076	-	Y	N	Y	Y	N	N	transl_inter	na	NA	intergenic	RP11-260M19.2,RP11-	+,-	lincRNA,linc	11054;77407	intergenic	TGM2,KIAA1755	-,-	protein_cod	28096;15814
3003	chr14	55382313	-	chr20	49282752	+	Y	Y	N	Y	Y	N	transl_inter	na	NA	intergenic	GCH1,WDHD1	-,-	protein_cod	12743;23355	intronic	FAM65C	-	protein_cod	0
3004	chr14	58412412	-	chr20	16307410	+	N	N	N	Y	N	N	transl_inter	na	NA	intergenic	SLC35F4,RP11-999E2	-,-	protein_cod	79632;47851	intronic	KIF16B	-	protein_cod	0
3005	chr14	55382327	+	chr20	49282746	-	N	N	N	Y	Y	N	transl_inter	na	NA	intergenic	GCH1,WDHD1	-,-	protein_cod	12757;23341	intronic	FAM65C	-	protein_cod	0
3006	chr14	90912037	+	chr20	25294227	-	Y	N	Y	Y	N	N	transl_inter	na	NA	intergenic	CALM1,RP11-1078H9.	+,-	protein_cod	37432;6370	intronic	ABHD12	-	protein_cod	0
3007	chr14	73502270	-	chr21	15811538	+	N	Y	N	N	N	N	transl_inter	na	NA	intergenic	ZFYVE1,RN7SL586P	-,-	protein_cod	8350;5059	intergenic	HSPA13,SAMSN1	-,-	protein_cod	56030;46011
3008	chr14	73502660	-	chr21	15811737	+	N	Y	N	Y	Y	Y	transl_inter	na	NA	intergenic	ZFYVE1,RN7SL586P	-,-	protein_cod	8740;4669	intergenic	HSPA13,SAMSN1	-,-	protein_cod	56229;45812
3009	chr14	48135718	+	chr22	30993448	+	N	Y	N	N	N	N	transl_inter	na	NA	intronic	MDGA2,MDGA2	-,-	protein_cod	0	intronic	PES1	-	protein_cod	0
3010	chr14	55491676	+	chr22	18934788	+	N	N	N	N	Y	N	transl_inter	na	NA	intronic	WDHD1	-	protein_cod	0	intergenic	PRODH,DGCR5	-,+	protein_cod	10722;23239
3011	chr14	55491728	+	chr22	18934856	+	N	N	N	N	N	Y	transl_inter	na	NA	intronic	WDHD1	-	protein_cod	0	intergenic	PRODH,DGCR5	-,+	protein_cod	10790;23171
3012	chr14	24322674	-	chr3	95372991	+	N	N	N	N	Y	N	transl_inter	na	NA	intergenic;ir	RP11-388E23.3,LINC0	-,-	pseudogene	26069;68782	downstream	MTHFD2P1	-	pseudogene	0
3013	chr14	93651318	-	chr3	169729734	+	Y	N	N	Y	N	Y	transl_inter	na	NA	UTR5	TMEM251,RP11-371E(	+,+	protein_cod	0	intergenic;ir	SEC62,GPR160	+,+	protein_cod	13573;25983
3014	chr14	24322674	+	chr3	95372991	-	N	N	N	N	Y	N	transl_inter	na	NA	intergenic;ir	RP11-388E23.3,LINC0	-,-	pseudogene	26069;68782	downstream	MTHFD2P1	-	pseudogene	0
3015	chr14	27118023	+	chr3	185734654	-	N	N	N	Y	N	N	transl_inter	na	NA	ncRNA_intro	NOVA1-AS1	+	antisense	0	intergenic	RN7SL637P,ETV5	-,-	misc_RNA,p	34571;29443
3016	chr14	50227821	+	chr3	137154962	-	N	Y	N	N	N	N	transl_inter	na	NA	intergenic	KLHDC1,KLHDC2	+,+	protein_cod	7951;6505	intergenic	IL20RB,RNA5SP142	+,-	protein_cod	425035;82014
3017	chr14	72962479	-	chr4	60491838	-	N	Y	N	N	N	N	transl_inter	na	NA	intronic;intro	RGS6	+	protein_cod	0	ncRNA_intro	RP11-725D20.1	-	lincRNA	0
3018	chr14	78559026	-	chr4	55036120	+	Y	Y	Y	Y	Y	Y	transl_inter	na	NA	intergenic	FRDAP,RNA5SP388	-,-	pseudogene	32405;85120	intronic	FIP1L1	+	protein_cod	0

3019	chr14	24263525	+	chr4	42678272	-	N	Y	N	N	N	N	transl_inter	na	NA	intergenic	RN7SKP205,RP11-38(	+,-	misc_RNA,p	2080;31320	intergenic	RP11-109E24.2,RP11-10	+,+	antisense,li	18327;29852
3020	chr14	52667767	+	chr4	170280994	+	Y	Y	Y	Y	Y	Y	transl_inter	na	NA	intergenic	RP11-1033H12.3,PTG	+,+	pseudogene	44870;66664	intergenic	SH3RF1,NEK1	-,-	protein_cod	88738;33432
3021	chr14	72962479	+	chr4	60491838	+	N	N	Y	N	N	N	transl_inter	na	NA	intronic;intro	RGS6	+	protein_cod	0	ncRNA_intro	RP11-725D20.1	-	lincRNA	0
3022	chr14	27118023	+	chr5	151671608	+	N	Y	Y	Y	N	N	transl_inter	na	NA	ncRNA_intro	NOVA1-AS1	+	antisense	0	intergenic	CTB-12O2.1,NMUR2	+,-	lincRNA,pro	21599;99485
3023	chr14	50227806	+	chr5	107791421	+	N	N	Y	N	N	N	transl_inter	na	NA	intergenic	KLHDC1,KLHDC2	+,+	protein_cod	7936;6520	intergenic	FBXL17,RP11-120B7.1	-,+	protein_cod	73622;137889
3024	chr14	100492730	+	chr5	88271357	+	N	N	N	Y	N	N	transl_inter	na	NA	intronic	EVL	+	protein_cod	0	ncRNA_intro	MEF2C-AS1	+	antisense	0
3025	chr14	44886653	-	chr6	78846808	-	N	N	N	N	N	Y	transl_inter	na	NA	intergenic	RP11-99L13.2,RP11-9	-,-	lincRNA,linc	23734;27297	intergenic	SNORD112,RP3-390M24	+,-	snoRNA,linc	199820;467376
3026	chr14	19036950	-	chr6	68100064	+	Y	Y	Y	Y	Y	Y	transl_inter	na	NA	intergenic	NONE,RP11-754I20.1	NONE,+	NONE,pseu	NONE;73253	intergenic	SNORD65,RP11-409K15	-,-	snoRNA,ps	179691;66175
3027	chr14	27117709	-	chr6	168285635	+	Y	Y	Y	Y	Y	Y	transl_inter	na	NA	ncRNA_intro	NOVA1-AS1	+	antisense	0	intronic	MLLT4	+	protein_cod	0
3028	chr14	77736064	+	chr6	84266489	-	N	N	Y	N	N	N	transl_inter	na	NA	intronic	NGB	-	protein_cod	0	intronic	SNAP91	-	protein_cod	0
3029	chr14	77736106	+	chr6	84266492	-	N	Y	N	N	N	N	transl_inter	na	NA	intronic	NGB	-	protein_cod	0	intronic	SNAP91	-	protein_cod	0
3030	chr14	49797015	+	chr6	67073055	+	N	N	N	N	Y	Y	transl_inter	na	NA	intergenic	RP11-816J8.1,RP11-3	-,+	lincRNA,pse	252792;43669	intergenic	AC002485.1,RP11-24C1	+,-	miRNA,pse	3663;347080
3031	chr14	77736123	+	chr6	17094332	+	Y	N	N	N	N	N	transl_inter	na	NA	intronic	NGB	-	protein_cod	0	intergenic	RP1-151F17.2,STMND1	+,+	lincRNA,pro	327218;8157
3032	chr14	100394236	-	chr7	158387983	-	N	N	N	N	Y	N	transl_inter	na	NA	intronic	EML1	+	protein_cod	0	intergenic;ir	MIR5707,THAP5P1	+,-	miRNA,pse	3595;27897
3033	chr14	100394261	-	chr7	158387983	-	N	N	N	Y	N	N	transl_inter	na	NA	intronic	EML1	+	protein_cod	0	intergenic;ir	MIR5707,THAP5P1	+,-	miRNA,pse	3595;27897
3034	chr14	100394294	-	chr7	158387918	-	Y	N	N	N	N	N	transl_inter	na	NA	intronic	EML1	+	protein_cod	0	intergenic	MIR5707,THAP5P1	+,-	miRNA,pse	3530;27962
3035	chr14	88065102	-	chr7	156875689	+	N	N	N	N	N	Y	transl_inter	na	NA	intergenic	RP11-594C13.1,CTD-2	-,-	lincRNA,linc	25736;35621	intergenic	RP4-639J15.1,RP4-639J	-,-	lincRNA,pse	24481;4960
3036	chr14	90911815	-	chr7	58017479	+	Y	N	Y	Y	N	N	transl_inter	na	NA	intergenic;ir	CALM1,RP11-1078H9.	+,-	protein_cod	37210;6592	intergenic	RP11-548K12.12,NONE	+,NONE	pseudogene	120727;NONE
3037	chr14	102511700	-	chr7	100938136	+	Y	Y	Y	Y	N	Y	transl_inter	na	NA	ncRNA_intro	RP11-1017G21.4	-	antisense	0	intergenic	AZGP1P2,RP11-132A1.3	-,+	pseudogene	5084;4758
3038	chr14	32719234	+	chr7	153094150	-	N	Y	N	N	N	Y	transl_inter	na	NA	intergenic	RP11-134E15.1,RP11-	-,-	pseudogene	6935;21827	intergenic	AC079809.2,AC073236.3	-,-	pseudogene	252477;2855
3039	chr14	35128039	+	chr7	109872933	+	N	N	Y	N	Y	Y	transl_inter	na	NA	intergenic	SNX6,RNU6-1261P	-,+	protein_cod	28650;17058	intergenic	RPL3P8,AC003088.1	-,-	pseudogene	233252;199363
3040	chr14	37233279	+	chr7	97688572	+	Y	N	N	N	N	N	transl_inter	na	NA	intronic	SLC25A21	-	protein_cod	0	intergenic	RN7SL13P,LMTK2	+,+	misc_RNA,p	35284;47625
3041	chr14	48030144	+	chr7	87333191	+	N	N	Y	N	N	N	transl_inter	na	NA	intronic	MDGA2,MDGA2	-,-	protein_cod	0	intronic	ABCB1,RUNDC3B	-,+	protein_cod	0
3042	chr14	70944697	+	chr7	138786219	+	N	Y	N	N	N	N	transl_inter	na	NA	ncRNA_intro	RP11-486O13.4,ADAM	-,-	lincRNA,linc	0	intronic	ZC3HAV1	-	protein_cod	0
3043	chr14	36674745	-	chr8	43226576	+	Y	Y	Y	Y	Y	Y	transl_inter	na	NA	intergenic;ir	AL162511.1,RP11-259	-,+	miRNA,lincF	8971;9068	intergenic;ir	AC022616.1,RP11-726G	-,+	miRNA,pse	4646;1126
3044	chr14	36674745	+	chr8	43226576	-	Y	Y	Y	Y	Y	Y	transl_inter	na	NA	intergenic;ir	AL162511.1,RP11-259	-,+	miRNA,lincF	8971;9068	intergenic;ir	AC022616.1,RP11-726G	-,+	miRNA,pse	4646;1126
3045	chr14	90911815	-	chr9	66836089	-	Y	N	Y	N	N	N	transl_inter	na	NA	intergenic;ir	CALM1,RP11-1078H9.	+,-	protein_cod	37210;6592	upstream	RP11-318K12.3	+	pseudogene	0
3046	chr14	77736144	+	chr9	99002429	-	N	N	Y	N	N	N	transl_inter	na	NA	intronic	NGB	-	protein_cod	0	intronic	HSD17B3	-	protein_cod	0
3047	chr14	70692482	+	chr9	13424341	+	N	Y	N	N	N	N	transl_inter	na	NA	ncRNA_intro	RP11-486O13.2	+	antisense	0	ncRNA_intro	RP11-536O18.2	-	lincRNA	0
3048	chr14	94846039	-	chrX	26049902	-	N	Y	N	Y	Y	Y	transl_inter	na	NA	intronic	SERPINA1	-	protein_cod	0	intergenic	RP11-86A5.1,MAGEB18	+,+	lincRNA,pro	138241;106558
3049	chr14	94846161	-	chrX	26050060	-	N	N	Y	N	N	N	transl_inter	na	NA	intronic	SERPINA1	-	protein_cod	0	intergenic	RP11-86A5.1,MAGEB18	+,+	lincRNA,pro	138399;106400
3050	chr14	44886683	-	chrX	77471136	+	N	Y	N	N	N	N	transl_inter	na	NA	intergenic	RP11-99L13.2,RP11-9	-,-	lincRNA,linc	23764;27267	intergenic	FNDC3CP,CYSLTR1	-,-	pseudogene	43217;55825
3051	chr14	40882900	+	chrX	21110230	-	N	N	N	N	N	Y	transl_inter	na	NA	intergenic	RP11-662J14.2,RP11-	+,+	lincRNA,linc	24556;541203	intergenic	RN7SKP183,RNU6-133P	+,+	misc_RNA,s	639681;122525
3052	chr14	63420594	-	chrY	18594207	-	Y	Y	N	Y	Y	Y	transl_inter	na	NA	intronic	KCNH5	-	protein_cod	0	intergenic	RNU6-184P,SURF6P1	-,+	snRNA,pse	145938;710499
3053	chr14	63420592	+	chrY	18594227	+	Y	Y	Y	Y	Y	Y	transl_inter	na	NA	intronic	KCNH5	-	protein_cod	0	intergenic	RNU6-184P,SURF6P1	-,+	snRNA,pse	145958;710479
3054	chr15	20452666	-	chr16	33406185	+	N	N	N	N	N	Y	transl_inter	na	NA	ncRNA_intro	RP11-492D6.3	-	pseudogene	0	ncRNA_intro	RP11-293B20.2	+	pseudogene	0
3055	chr15	20108513	+	chr16	32819045	-	N	N	N	N	N	Y	transl_inter	na	NA	intergenic	RP11-173D3.3,IGHV1(	+,-	pseudogene	3701;61406	intergenic	HERC2P5,RP11-67H24.2	+,+	pseudogene	31528;1832
3056	chr15	22412531	+	chr16	21835137	-	N	N	Y	Y	Y	N	transl_inter	na	NA	ncRNA_intro	RP11-69H14.6	+	sense_over	0	intergenic	RRN3P1,NPIPB4	-,-	pseudogene	3406;10753
3057	chr15	20618287	+	chr16	32849634	+	N	Y	N	N	N	N	transl_inter	na	NA	ncRNA_intro	HERC2P3	-	pseudogene	0	intergenic	RP11-67H24.2,RP11-989	+,-	lincRNA,pse	13668;7651
3058	chr15	69257218	-	chr17	78511693	-	N	N	N	N	Y	N	transl_inter	na	NA	ncRNA_intro	RP11-809H16.2	+	processed_	0	intergenic	CTD-2526A2.6,RPL32P3	+,+	pseudogene	22081;4310
3059	chr15	69257899	+	chr17	78511839	+	N	N	Y	N	N	Y	transl_inter	na	NA	ncRNA_intro	RP11-809H16.2	+	processed_	0	intergenic	CTD-2526A2.6,RPL32P3	+,+	pseudogene	22227;4164
3060	chr15	54939522	-	chr18	23467619	-	Y	N	N	N	N	Y	transl_inter	na	NA	intergenic	UNC13C,RP11-548M1	+,+	protein_cod	18716;409423	intergenic	RN7SL97P,RP11-737G2	-,-	misc_RNA,p	69123;65561
3061	chr15	83373435	-	chr18	56311562	+	N	N	N	N	N	Y	transl_inter	na	NA	intronic	AP3B2	-	protein_cod	0	intergenic	RPL9P31,RP11-126O1.5	+,-	pseudogene	12210;26150
3062	chr15	26864829	+	chr18	50612366	-	N	N	Y	N	N	N	transl_inter	na	NA	intronic	GABRB3	-	protein_cod	0	intronic	DCC	+	protein_cod	0
3063	chr15	52362742	-	chr19	34441681	-	N	Y	N	N	Y	N	transl_inter	na	NA	ncRNA_intro	CTD-2184D3.5	-	antisense	0	intergenic	RN7SL150P,RPS4XP23	-,+	misc_RNA,p	23151;70883
3064	chr15	97418531	-	chr19	54337092	+	N	Y	N	N	N	N	transl_inter	na	NA	intergenic	RN7SKP181,AC05587	+,-	misc_RNA,r	32917;215961	intergenic	NLRP12,AC008753.6	-,-	protein_cod	9444;20743
3065	chr15	21338798	-	chr2	91727906	-	N	N	Y	N	Y	N	transl_inter	na	NA	intergenic	RP11-854K16.3,BMS1	-,+	lincRNA,pse	3374;26836	intergenic	AC018696.5,AC018696.6	-,-	pseudogene	3990;6268
3066	chr15	41323328	-	chr2	207583	-	N	N	N	N	N	Y	transl_inter	na	NA	intronic	INO80	-	protein_cod	0	intergenic	AC079779.7,SH3YL1	+,-	antisense,p	4978;10553
3067	chr15	40963887	-	chr2	5628675	+	N	N	Y	Y	N	N	transl_inter	na	NA	intergenic	CASC5,RAD51-AS1	+,-	protein_cod	7347;15035	intergenic	AC073143.1,AC107057.1	-,-	pseudogene	174408;61237
3068	chr15	52362717	+	chr2	24843628	-	N	Y	Y	N	Y	N	transl_inter	na	NA	ncRNA_intro	CTD-2184D3.5	-	antisense	0	intronic	NCOA1	+	protein_cod	0
3069	chr15	89213025	+	chr2	84728529	-	N	N	N	Y	N	N	transl_inter	na	NA	intergenic	ISG20,RP11-343B18.2	+,-	protein_cod	13311;127619	intergenic	SUCLG1,DNAH6	-,+	protein_cod	41925;15050
3070	chr15	89213491	+	chr2	84728617	-	N	N	N	N	N	Y	transl_inter	na	NA	intergenic	ISG20,RP11-343B18.2	+,-	protein_cod	13777;127153	intergenic	SUCLG1,DNAH6	-,+	protein_cod	42013;14962
3071	chr15	101250531	-	chr20	62325194	+	N	N	Y	N	N	N	transl_inter	na	NA	intergenic	ASB7,RP11-66B24.5	+,+	protein_cod	58621;5923	intronic	RTEL1-TNFRSF6B,RTEL	+,+	protein_cod	0
3072	chr15	101250497	+	chr20	62324912	-	N	N	N	N	N	Y	transl_inter	na	NA	intergenic	ASB7,RP11-66B24.5	+,+	protein_cod	58587;5957	intronic	RTEL1-TNFRSF6B,RTEL	+,+	protein_cod	0
3073	chr15	20148812	+	chr22	17392952	-	Y	Y	Y	N	N	N	transl_inter	na	NA	intergenic	RP11-173D3.3,IGHV1(	+,-	pseudogene	44000;21107	intergenic	IGKV1OR22-5,IGKV2OR	-,-	IG_V_pseu	7366;2173
3074	chr15	23707592	+	chr22	42798895	+	Y	N	N	N	Y	Y	transl_inter	na	NA	intergenic	GOLGA6L2,MIR4508	-,-	protein_cod	15211;99617	intronic	NFAM1	-	protein_cod	0
3075	chr15	78892114	+	chr22	31416887	+	Y	Y	Y	Y	Y	Y	transl_inter	na	NA	intronic	CHRNA3	-	protein_cod	0	intergenic	TUG1,RN7SL633P	+,+	antisense,m	41506;39088
3076	chr15	84364017	+	chr22	50777759	+	N	Y	N	N	N	N	transl_inter	na	NA	intronic	ADAMTSL3	+	protein_cod	0	intergenic	DENND6B,PPP6R2	-,+	protein_cod	12270;3974
3077	chr15	84364027	+	chr22	50776291	+	N	N	Y	N	N	N	transl_inter	na	NA	intronic	ADAMTSL3	+	protein_cod	0	intergenic	DENND6B,PPP6R2	-,+	protein_cod	10802;5442
3078	chr15	84364327	+	chr22	50777898	+	N	N	N	N	Y	N	transl_inter	na	NA	intronic	ADAMTSL3	+	protein_cod	0	intergenic	DENND6B,PPP6R2	-,+	protein_cod	12409;3835
3079	chr15	35948467	-	chr3	126090202	-	N	N	Y	N	N	Y	transl_inter	na	NA	ncRNA_intro	DPH6-AS1	+	lincRNA	0	intergenic	KLF15,RP11-71E19.5	-,-	protein_cod	13917;21673
3080	chr15	71021993	-	chr3	84433395	-	Y	Y	Y	N	N	Y	transl_inter	na	NA	intronic	UACA	-	protein_cod	0	intergenic	AC108696.1,AC107025.1	-,+	miRNA,miR	93991;112332
3081	chr15	91112793	+	chr3	142283122	+	Y	N	Y	N	Y	Y	transl_inter	na	NA	intronic;intro	CRTC3	+	protein_cod	0	intronic;intro	ATR	-	protein_cod	0
3082	chr15	54509132	+	chr4	142654869	+	N	N	Y	N	N	N	transl_inter	na	NA	intronic	UNC13C	+	protein_cod	0	UTR3	IL15	+	protein_cod	0
3083	chr15	36380873	-	chr5	146587668	-	Y	Y	Y	Y	Y	Y	transl_inter	na	NA	ncRNA_intro	RP11-184D12.1	-	lincRNA	0	ncRNA_intro	CTC-255N20.1	-	antisense	0
3084	chr15	68688089	-	chr5	83724296	+	N	N	N	N	Y	N	transl_inter	na	NA	intronic	ITGA11	-	protein_cod	0	ncRNA_intro	CTD-2269F5.1	+	antisense	0
3085	chr15	79784427	-	chr5	96849601	+	N	N	N	N	N	Y	transl_inter	na	NA	intergenic;ir	KIAA1024,RP11-621H	+,-	protein_cod	19795;67171	ncRNA_intro	RP11-1E3.1	+	lincRNA	0
3086	chr15	79784427	+	chr5	96849601	-	N	N	Y	N	N	N	transl_inter	na	NA	intergenic;ir	KIAA1024,RP11-621H	+,-	protein_cod	19795;67171	ncRNA_intro	RP11-1E3.1	+	lincRNA	0
3087	chr15	79784432	+	chr5	96849706	-	Y	N	N	N	N	N	transl_inter	na	NA	intergenic;ir	KIAA1024,RP11-621H	+,-	protein_cod	19800;67166	ncRNA_intro	RP11-1E3.1	+	lincRNA	0
3088	chr15	40854180	-	chr6	116774818	-	Y	Y	Y	Y	Y	Y	transl_inter	na	NA	intronic;intro	C15orf57	-	protein_cod	0	upstream	CBX3P9	-	pseudogene	0
3089	chr15	70839566	-	chr6	123171431	-	Y	Y	Y	Y	Y	Y	transl_inter	na	NA	intergenic	RP11-96C21.1,RP11-6	-,+	lincRNA,pse	41299;54897	intergenic	SMPDL3A,ATP5LP2	+,-	protein_cod	40566;9392
3090	chr15	42431621	+	chr6	153748709	+	Y	Y	N	Y	N	N	transl_inter	na	NA	intergenic	PLA2G4D,PLA2G4F	-,-	protein_cod	44869;1711	intergenic;ir	RNA5SP225,RP11-331O	+,+	rRNA,pseu	7002;238133
3091	chr15	78892087	-	chr7	92962216	-	Y	Y	N	N	N	N	transl_inter	na	NA	intronic	CHRNA3	-	protein_cod	0	intronic	CCDC132	+	protein_cod	0
3092	chr15	40854180	-	chr7	26241365	+	N	Y	N	N	Y	Y	transl_inter	na	NA	intronic;intro	C15orf57	-	protein_cod	0	UTR5	CBX3	+	protein_cod	0
3093	chr15	45339240	-	chr7	100616362	+	Y	N	N	N	N	N	transl_inter	na	NA	ncRNA_intro	RP11-109D20.1	-	antisense	0	intronic;intro	MUC12	+	protein_cod	0
3094	chr15	20126961	+	chr7	65059464	-	N	N	N	Y	N	N	transl_inter	na	NA	intergenic	RP11-173D3.3,IGHV1(	+,-	pseudogene	22149;42958	intergenic	RP11-667F9.1,RP13-157	-,+	pseudogene	39807;22526
3095	chr15	20149936	+	chr7	57927941	-	N	Y	Y	Y	Y	Y	transl_inter	na	NA	intergenic	RP11-173D3.3,IGHV1(	+,-	pseudogene	45124;19983	intergenic	RP11-548K12.12,NONE	+,NONE	pseudogene	31189;NONE
3096	chr15	40854194	+	chr7	26252971	-	N	Y	N	N	Y	Y	transl_inter	na	NA	intronic;intro	C15orf57	-	protein_cod	0	UTR3;UTR3	CBX3	+	protein_cod	0
3097	chr15	45339240	+	chr7	100616362	-	N	N	Y	Y	Y	Y	transl_inter	na	NA	ncRNA_intro	RP11-109D20.1	-	antisense	0	intronic;intro	MUC12	+	protein_cod	0
3098	chr15	86440349	-	chr8	144263624	-	N	N	N	N	Y	N	transl_inter	na	NA	intergenic	MIR548AP,RP11-23A2	+,-	miRNA,pseu	71388;61599	intergenic	LY6H,GPIHBP1	-,+	protein_cod	21496;31444
3099	chr15	45764452	-	chr9	132204424	-	Y	N	N	Y	Y	N	transl_inter	na	NA	ncRNA_intro	RP11-519G16.3	+	antisense	0	ncRNA_intro	RP11-65J3.3	-	lincRNA	0
3100	chr15	36380904	-	chr9	99002215	+	N	N	N	Y	N	N	transl_inter	na	NA	ncRNA_intro	RP11-184D12.1	-	lincRNA	0	intronic;intro	HSD17B3	-	protein_cod	0
3101	chr15	20127166	+	chr9	69809434	-	Y	N	N	N	N	N	transl_inter	na	NA	intergenic	RP11-173D3.3,IGHV1(	+,-	pseudogene	22354;42753	intergenic	IGKV1OR-2,AL359955.1	-,-	IG_V_pseu	31481;12326
3102	chr15	20401657	+	chr9	21127601	-	N	N	N	Y	N	N	transl_inter	na	NA	intergenic	BMS1P15,RP11-492D	+,-	pseudogene	35578;32216	intergenic	IFNWP4,IFNW1	+,-	pseudogene	20766;13030

3103	chr15	61363571	+	chr9	137114639	-	N	Y	N	N	N	N	transl_inter	na	NA	intronic	RORA	-	protein_cod	0	intergenic;ir	LL09NC01-139C3.1,RP1	+,-	lincRNA,linc	80177;71138
3104	chr15	61363607	+	chr9	137114639	-	N	N	N	N	Y	N	transl_inter	na	NA	intronic	RORA	-	protein_cod	0	intergenic;ir	LL09NC01-139C3.1,RP1	+,-	lincRNA,linc	80177;71138
3105	chr15	86440164	+	chr9	74036144	-	N	N	N	N	Y	N	transl_inter	na	NA	intergenic	MIR548AP,RP11-23A2	+,-	miRNA,pseu	71203;61784	intronic	TRPM3	-	protein_cod	0
3106	chr15	75867612	+	chr9	115151079	+	N	Y	N	N	Y	N	transl_inter	na	NA	ncRNA_intr	CTD-2323K18.1	-	processed_	0	intronic	HSDL2	+	protein_cod	0
3107	chr15	24871846	-	chrX	147336144	-	Y	N	N	N	N	Y	transl_inter	na	NA	ncRNA_intr	PWRN1	+	lincRNA	0	intergenic	U40455.1,RPL7L1P11	-,+	pseudogene	55746;210084
3108	chr15	24871859	-	chrX	147336170	-	N	N	N	N	Y	N	transl_inter	na	NA	ncRNA_intr	PWRN1	+	lincRNA	0	intergenic	U40455.1,RPL7L1P11	-,+	pseudogene	55772;210058
3109	chr15	24871861	-	chrX	147336567	-	N	N	Y	N	N	N	transl_inter	na	NA	ncRNA_intr	PWRN1	+	lincRNA	0	intergenic	U40455.1,RPL7L1P11	-,+	pseudogene	56169;209661
3110	chr16	88624311	-	chr17	7233203	+	N	Y	Y	Y	Y	Y	transl_inter	na	NA	intergenic	ZFPM1,ZC3H18	+,+	protein_cod	20887;12478	upstream	NEURL4,AC026954.6	-,+	protein_cod	0
3111	chr16	88943425	+	chr17	7683145	-	N	Y	Y	Y	Y	Y	transl_inter	na	NA	exonic	CBFA2T3	-	protein_cod	0	intronic	DNAH2	+	protein_cod	0
3112	chr16	33852728	+	chr17	25289488	+	N	N	Y	N	N	N	transl_inter	na	NA	ncRNA_intr	RP11-598D12.2	+	pseudogene	0	intergenic	NONE,RP11-260A9.1	NONE,+	NONE,pseu	NONE;19232
3113	chr16	34178197	+	chr17	25281298	+	Y	Y	N	N	N	N	transl_inter	na	NA	intergenic	RNA5-8SP2,CTD-2144	+,-	rRNA,lincRN	212620;34918	intergenic	NONE,RP11-260A9.1	NONE,+	NONE,pseu	NONE;27422
3114	chr16	33041116	-	chr18	15165808	-	N	N	Y	N	Y	Y	transl_inter	na	NA	intergenic;ir	RP11-19N8.2,RP11-19	-,-	pseudogene	1758;13814	intergenic	RP11-805F19.1,RP11-45	-,-	lincRNA,pse	1342;31190
3115	chr16	33041116	-	chr18	15166384	-	N	Y	N	N	N	N	transl_inter	na	NA	intergenic;ir	RP11-19N8.2,RP11-19	-,-	pseudogene	1758;13814	intergenic	RP11-805F19.1,RP11-45	-,-	lincRNA,pse	1918;30614
3116	chr16	81084831	-	chr19	7820332	+	N	N	Y	N	Y	N	transl_inter	na	NA	intergenic	ATMIN,C16orf46	+,-	protein_cod	3868;2271	intergenic	RPL21P129,CLEC4M	+,+	pseudogene	4285;7703
3117	chr16	33853339	+	chr19	33444599	-	N	Y	Y	Y	Y	N	transl_inter	na	NA	ncRNA_intr	RP11-598D12.2	+	pseudogene	0	exonic;exon	CEP89	-	protein_cod	0
3118	chr16	49885315	+	chr19	17870049	+	Y	Y	N	Y	Y	Y	transl_inter	na	NA	intronic	ZNF423	-	protein_cod	0	intronic	FCHO1	+	protein_cod	0
3119	chr16	19251826	-	chr2	208963272	-	N	N	Y	N	N	Y	transl_inter	na	NA	intronic	SYT17	+	protein_cod	0	intergenic	snoU13,CRYGEP	-,-	snoRNA,psi	24386;9685
3120	chr16	19251865	-	chr2	208963273	-	N	N	N	N	Y	N	transl_inter	na	NA	intronic	SYT17	+	protein_cod	0	intergenic	snoU13,CRYGEP	-,-	snoRNA,psi	24387;9684
3121	chr16	19263768	-	chr2	137698271	-	Y	N	N	Y	N	Y	transl_inter	na	NA	intronic	SYT17	+	protein_cod	0	intronic	THSD7B	+	protein_cod	0
3122	chr16	46443644	-	chr2	89842368	-	N	N	Y	N	Y	N	transl_inter	na	NA	intergenic	NONE,ANKRD26P1	NONE,-	NONE,pseu	NONE;59609	intergenic	IGKV2-40,IGKV2D-40	-,+	IG_V_gene,	212182;48233
3123	chr16	33961395	-	chr2	133014882	+	N	N	N	Y	N	N	transl_inter	na	NA	ncRNA_exc	LINC00273	-	lincRNA	0	upstream	MIR663B	-	miRNA	0
3124	chr16	33964526	-	chr2	133011773	+	Y	N	N	N	N	N	transl_inter	na	NA	upstream	RNA5-8SP2	+	rRNA	0	upstream	RNA5-8SP5	-	rRNA	0
3125	chr16	33970816	-	chr2	133007551	+	N	N	N	Y	N	Y	transl_inter	na	NA	intergenic	RNA5-8SP2,CTD-2144	+,-	rRNA,lincRN	5239;242299	intergenic	ANKRD30BL,RNA5-8SP	-,-	protein_cod	88023;3176
3126	chr16	19263648	+	chr2	204407328	-	Y	Y	N	N	Y	N	transl_inter	na	NA	intronic	SYT17	+	protein_cod	0	intergenic	RAPH1,AC125238.2	-,-	protein_cod	7195;91972
3127	chr16	33953538	+	chr2	133029738	-	N	N	N	Y	N	N	transl_inter	na	NA	intergenic	AC136932.2,LINC0027	-,-	miRNA,lincF	7517;7514	intergenic	CDC27P1,AC097532.2	+,-	pseudogene	9123;13630
3128	chr16	33958399	+	chr2	133025115	-	N	N	N	N	Y	N	transl_inter	na	NA	intergenic	AC136932.2,LINC0027	-,-	miRNA,lincF	12378;2653	intergenic	CDC27P1,AC097532.2	+,-	pseudogene	4500;18253
3129	chr16	33964365	+	chr2	133011830	-	Y	N	N	N	N	Y	transl_inter	na	NA	intergenic	LINC00273,RNA5-8SF	-,+	lincRNA,rRN	1862;1061	upstream	RNA5-8SP5	-	rRNA	0
3130	chr16	33966412	+	chr2	133009949	-	N	N	N	N	N	Y	transl_inter	na	NA	downstream	RNA5-8SP2	+	rRNA	0	downstream	RNA5-8SP5	-	rRNA	0
3131	chr16	46477706	+	chr2	91980978	-	N	N	N	Y	Y	Y	transl_inter	na	NA	intergenic	NONE,ANKRD26P1	NONE,-	NONE,pseu	NONE;25547	intergenic	GGT8P,IGKV1OR-1	+,+	pseudogene	11783;24819
3132	chr16	33970145	+	chr2	76668102	+	N	Y	N	Y	N	N	transl_inter	na	NA	intergenic	RNA5-8SP2,CTD-2144	+,-	rRNA,lincRN	4568;242970	intergenic	RP11-335E8.1,RN7SKP2	-,-	pseudogene	179315;4103
3133	chr16	33961192	-	chr20	26189231	+	Y	N	N	N	N	N	transl_inter	na	NA	ncRNA_exc	LINC00273	-	lincRNA	0	ncRNA_intr	MIR663A	-	processed_	0
3134	chr16	32419921	+	chr21	17311988	+	N	N	N	N	N	Y	transl_inter	na	NA	ncRNA_intr	RP11-626K17.5	+	pseudogene	0	intergenic	USP25,RNU6-426P	+,-	protein_cod	59611;95745
3135	chr16	10924342	+	chr22	23852644	-	N	N	N	N	Y	N	transl_inter	na	NA	intergenic	TVP23A,RP11-876N24	-,-	protein_cod	11700;9899	intergenic	AP000345.1,AP000345.4	-,-	lincRNA,linc	23477;2037
3136	chr16	32823660	+	chr22	16867653	-	N	N	Y	N	N	N	transl_inter	na	NA	ncRNA_intr	RP11-67H24.2	+	lincRNA	0	upstream	ABCD1P4	+	pseudogene	0
3137	chr16	32127303	+	chr22	16867402	+	Y	N	Y	N	N	N	transl_inter	na	NA	ncRNA_intr	HERC2P4	-	pseudogene	0	intergenic	LA16c-13E4.3,ABCD1P4	-,+	pseudogene	180157;1018
3138	chr16	32127401	+	chr22	16867611	+	N	N	Y	N	N	N	transl_inter	na	NA	ncRNA_intr	HERC2P4	-	pseudogene	0	upstream	ABCD1P4	+	pseudogene	0
3139	chr16	32127752	+	chr22	16867332	+	N	N	N	N	Y	N	transl_inter	na	NA	ncRNA_intr	HERC2P4	-	pseudogene	0	intergenic	LA16c-13E4.3,ABCD1P4	-,+	pseudogene	180087;1088
3140	chr16	32127861	+	chr22	16867394	+	N	N	N	N	N	Y	transl_inter	na	NA	ncRNA_intr	HERC2P4	-	pseudogene	0	intergenic	LA16c-13E4.3,ABCD1P4	-,+	pseudogene	180149;1026
3141	chr16	46500066	+	chr22	16850167	+	Y	N	N	N	Y	N	transl_inter	na	NA	intergenic	NONE,ANKRD26P1	NONE,-	NONE,pseu	NONE;3187	intergenic	LA16c-13E4.3,ABCD1P4	-,+	pseudogene	162922;18253
3142	chr16	87256635	+	chr22	45380071	+	N	N	N	Y	N	N	transl_inter	na	NA	ncRNA_intr	RP11-899L11.1	+	lincRNA	0	intronic	PHF21B	-	protein_cod	0
3143	chr16	19332992	-	chr3	25939094	-	Y	Y	N	Y	Y	Y	transl_inter	na	NA	intronic	CLEC19A	+	protein_cod	0	intergenic	LINC00692,RPEP2	-,+	protein_cod	23905;118066
3144	chr16	64354116	-	chr4	181791411	-	N	Y	N	N	N	N	transl_inter	na	NA	ncRNA_intr	AC012322.1	-	lincRNA	0	intergenic	RP11-460H9.1,LINC0029	-,-	lincRNA,linc	111220;193831
3145	chr16	22092926	+	chr4	7949654	+	Y	Y	Y	Y	Y	Y	transl_inter	na	NA	UTR3	C16orf52	+	protein_cod	0	intergenic	AC097381.1,ABLIM2	+,-	protein_cod	7632;17385
3146	chr16	27952974	-	chr5	174884958	-	N	N	N	N	Y	N	transl_inter	na	NA	intronic	GSG1L	-	protein_cod	0	intergenic	snoU13,SFXN1	-,+	snoRNA,prc	7037;19107
3147	chr16	33992443	+	chr5	123269399	+	N	N	N	N	Y	N	transl_inter	na	NA	intergenic	RNA5-8SP2,CTD-2144	+,-	rRNA,lincRN	26866;220672	intergenic	KRT18P16,HMGB1P29	+,-	pseudogene	296302;286873
3148	chr16	34229729	+	chr5	118935405	+	N	Y	N	N	N	N	transl_inter	na	NA	intergenic	CTD-2144E22.8,AC13	-,+	lincRNA,pse	15514;2377	intergenic	FABP5P6,snoU13	-,-	pseudogene	44052;10492
3149	chr16	62522384	-	chr6	165196148	-	Y	Y	Y	N	N	Y	transl_inter	na	NA	intergenic	snoU13,AC009110.1	+,+	snoRNA,linc	84126;107892	intergenic	RP11-347L18.1,RP11-30	-,+	lincRNA,linc	426471;45091
3150	chr16	33237322	-	chr6	256765	+	Y	Y	N	N	N	N	transl_inter	na	NA	intronic	TP53TG3C	+	protein_cod	0	intergenic	RP3-416J7.5,DUSP22	+,+	lincRNA,prc	50373;34865
3151	chr16	86498129	-	chr6	3222645	+	Y	N	N	N	N	N	transl_inter	na	NA	intergenic	RP11-158I3.1,FENDRI	-,-	pseudogene	28639;10006	intergenic	RP1-40E16.9,TUBB2B	-,-	lincRNA,prc	26655;1850
3152	chr16	13037188	+	chr6	4979236	-	N	N	N	N	Y	N	transl_inter	na	NA	intronic	SHISA9	+	protein_cod	0	upstream	RP11-428J1.2	+	pseudogene	0
3153	chr16	33428529	+	chr6	382460	-	Y	Y	Y	Y	Y	Y	transl_inter	na	NA	intergenic	RP11-293B20.2,BMS1	+,-	pseudogene	3518;60963	intergenic	DUSP22,IRF4	+,+	protein_cod	31105;9279
3154	chr16	33480458	+	chr6	95680455	-	Y	N	Y	N	N	Y	transl_inter	na	NA	intergenic	RP11-293B20.2,BMS1	+,-	pseudogene	55447;9034	intergenic	RP3-399J4.1,CYCSP17	-,+	pseudogene	523558;271603
3155	chr16	33522631	-	chr7	61078733	-	N	N	N	Y	N	N	transl_inter	na	NA	intergenic	BMS1P8,ENPP7P13	-,+	pseudogene	21660;49257	intergenic	NONE,RP11-715L17.1	NONE,+	NONE,pseu	NONE;743136
3156	chr16	33522675	-	chr7	61078731	-	Y	N	N	N	N	N	transl_inter	na	NA	intergenic	BMS1P8,ENPP7P13	-,+	pseudogene	21704;49213	intergenic	NONE,RP11-715L17.1	NONE,+	NONE,pseu	NONE;743138
3157	chr16	33854185	-	chr7	61822573	-	N	N	N	Y	N	N	transl_inter	na	NA	downstream	RP11-598D12.2	+	pseudogene	0	downstream	RP11-715L17.1	+	pseudogene	0
3158	chr16	46437163	-	chr7	61744660	-	N	Y	N	N	N	N	transl_inter	na	NA	intergenic	NONE,ANKRD26P1	NONE,-	NONE,pseu	NONE;66090	intergenic	NONE,RP11-715L17.1	NONE,+	NONE,pseu	NONE;77209
3159	chr16	88365209	-	chr7	155110261	-	N	N	Y	N	N	N	transl_inter	na	NA	intergenic	LA16c-444G7.1,ZNF46	+,+	lincRNA,prc	29092;128670	intergenic	INSIG1,BLACE	+,-	protein_cod	8316;39419
3160	chr16	33038386	-	chr7	61822454	+	N	N	N	Y	N	N	transl_inter	na	NA	ncRNA_intr	RP11-19N8.2	-	pseudogene	0	downstream	RP11-715L17.1	+	pseudogene	0
3161	chr16	32852146	+	chr7	61827672	-	N	N	N	N	N	Y	transl_inter	na	NA	intergenic	RP11-67H24.2,RP11-9	+,-	lincRNA,pse	16180;5139	intergenic	RP11-715L17.1,RNU6-41	+,-	pseudogene	5486;644169
3162	chr16	32852355	+	chr7	61827755	-	N	Y	N	N	N	N	transl_inter	na	NA	intergenic	RP11-67H24.2,RP11-9	+,-	lincRNA,pse	16389;4930	intergenic	RP11-715L17.1,RNU6-41	+,-	pseudogene	5569;644086
3163	chr16	33040587	+	chr7	61820917	-	N	N	N	Y	N	N	transl_inter	na	NA	intergenic	RP11-19N8.2,RP11-19	-,-	pseudogene	1229;14343	upstream	RP11-715L17.1	+	pseudogene	0
3164	chr16	33041626	+	chr7	61819970	-	N	N	N	N	N	Y	transl_inter	na	NA	intergenic	RP11-19N8.2,RP11-19	-,-	pseudogene	2268;13304	intergenic;ir	NONE,RP11-715L17.1	NONE,+	NONE,pseu	NONE;1899
3165	chr16	33041651	+	chr7	61819970	-	N	N	N	Y	N	N	transl_inter	na	NA	intergenic	RP11-19N8.2,RP11-19	-,-	pseudogene	2293;13279	intergenic;ir	NONE,RP11-715L17.1	NONE,+	NONE,pseu	NONE;1899
3166	chr16	33853005	+	chr7	57928329	-	Y	N	N	N	N	N	transl_inter	na	NA	ncRNA_intr	RP11-598D12.2	+	pseudogene	0	intergenic	RP11-548K12.12,NONE	+,NONE	pseudogene	31577;NONE
3167	chr16	33893266	+	chr7	61753229	-	N	N	N	N	Y	N	transl_inter	na	NA	intergenic	RP11-598D12.2,CTD-2	+,+	pseudogene	39438;29671	intergenic	NONE,RP11-715L17.1	NONE,+	NONE,pseu	NONE;68640
3168	chr16	33977019	+	chr7	32127623	-	N	Y	Y	N	N	Y	transl_inter	na	NA	intergenic	RNA5-8SP2,CTD-2144	+,-	rRNA,lincRN	11442;236096	intronic	PDE1C	-	protein_cod	0
3169	chr16	46413461	+	chr7	61754021	-	N	N	Y	N	N	N	transl_inter	na	NA	intergenic	NONE,ANKRD26P1	NONE,-	NONE,pseu	NONE;90089	intergenic	NONE,RP11-715L17.1	NONE,+	NONE,pseu	NONE;67848
3170	chr16	71384453	+	chr7	100619396	-	Y	N	Y	Y	Y	Y	transl_inter	na	NA	intergenic	AC106736.1,CALB2	+,+	miRNA,prot	19251;8163	intronic	MUC12	+	protein_cod	0
3171	chr16	33521338	+	chr7	57918040	+	Y	Y	N	Y	Y	N	transl_inter	na	NA	intergenic;ir	BMS1P8,ENPP7P13	-,+	pseudogene	20367;50550	intergenic;ir	RP11-548K12.12,NONE	+,NONE	pseudogene	21288;NONE
3172	chr16	33575506	+	chr7	61823944	+	N	Y	N	Y	Y	Y	transl_inter	na	NA	intergenic	RP11-104C4.4,IGHV3(	+,+	pseudogene	1791;29725	intergenic	RP11-715L17.1,RNU6-41	+,-	pseudogene	1758;647897
3173	chr16	46454292	+	chr7	61055485	+	N	Y	N	N	N	N	transl_inter	na	NA	intergenic	NONE,ANKRD26P1	NONE,-	NONE,pseu	NONE;48961	intergenic	NONE,RP11-715L17.1	NONE,+	NONE,pseu	NONE;766384
3174	chr16	46497336	+	chr7	61756422	+	N	Y	N	N	N	N	transl_inter	na	NA	intergenic	NONE,ANKRD26P1	NONE,-	NONE,pseu	NONE;5917	intergenic	NONE,RP11-715L17.1	NONE,+	NONE,pseu	NONE;65447
3175	chr16	23787312	-	chr8	118976905	-	N	N	Y	N	N	N	transl_inter	na	NA	intergenic	CHP2,PRKCB	+,+	protein_cod	17040;60010	intronic	EXT1	-	protein_cod	0
3176	chr16	33949875	-	chr8	65020692	+	Y	N	N	N	Y	N	transl_inter	na	NA	intergenic	AC136932.2,LINC0027	-,-	miRNA,lincF	3854;11177	ncRNA_intr	RP11-32K4.1	-	antisense	0
3177	chr16	69674	+	chr8	146301108	-	N	N	Y	N	N	N	transl_inter	na	NA	ncRNA_intr	Z84812.4	-	processed_	0	intergenic	C8orf33,NONE	+,NONE	protein_cod	19692;NONE
3178	chr16	61325	+	chr9	11230	+	N	Y	Y	N	N	N	transl_inter	na	NA	upstream	DDX11L10	+	pseudogene	0	upstream	DDX11L5	+	pseudogene	0
3179	chr16	33943702	+	chr9	68413469	+	N	N	N	N	Y	N	transl_inter	na	NA	downstream	CTD-2522B17.4	-	pseudogene	0	downstream	LINC00537	-	pseudogene	0
3180	chr16	33944016	+	chr9	68413459	+	N	N	N	N	N	Y	transl_inter	na	NA	ncRNA_exc	CTD-2522B17.4	-	pseudogene	0	downstream	LINC00537	-	pseudogene	0
3181	chr16	58406134	-	chrX	17910987	+	N	Y	N	N	N	N	transl_inter	na	NA	intergenic	PRSS54,RNU6-269P	-,-	protein_cod	77183;5822	intergenic	RAI2,RP1-272E8.1	-,+	protein_cod	31530;40482
3182	chr16	49711341	+	chrX	19470079	+	Y	N	N	N	N	N	transl_inter	na	NA	intronic	ZNF423	-	protein_cod	0	intronic	MAP3K15	-	protein_cod	0
3183	chr16	33899086	-	chrY	58978109	-	N	N	Y	N	N	N	transl_inter	na	NA	intergenic	RP11-598D12.2,CTD-2	+,+	pseudogene	45258;23851	intergenic	NONE,CTBP2P1	NONE,+	NONE,pseu	NONE;23282
3184	chr16	33899652	-	chrY	58978597	-	N	N	N	N	Y	N	transl_inter	na	NA	intergenic	RP11-598D12.2,CTD-2	+,+	pseudogene	45824;23285	intergenic	NONE,CTBP2P1	NONE,+	NONE,pseu	NONE;22794
3185	chr16	33943713	-	chrY	10013527	-	N	N	N	N	Y	N	transl_inter	na	NA	downstream	CTD-2522B17.4	-	pseudogene	0	intergenic	PCMTD1P1,CDC27P2	+,-	pseudogene	1711;14459
3186	chr16	33943766	-	chrY	10013568	-	N	N	Y	N	N	N	transl_inter	na	NA	ncRNA_exc	CTD-2522B17.4	-	pseudogene	0	intergenic	PCMTD1P1,CDC27P2	+,-	pseudogene	1752;14418

3187	chr16	33949118	-	chrY	10016004	-	N	N	N	N	N	Y	transl_inter	na	NA	intergenic	AC136932.2,LINC0027	-,-	miRNA,lincF	3097;11934	intergenic	PCMTD1P1,CDC27P2	+,-	pseudogene	4188;11982
3188	chr16	33950510	-	chrY	10017060	-	N	N	Y	N	N	N	transl_inter	na	NA	intergenic	AC136932.2,LINC0027	-,-	miRNA,lincF	4489;10542	intergenic	PCMTD1P1,CDC27P2	+,-	pseudogene	5244;10926
3189	chr16	33950511	-	chrY	10017572	-	N	N	N	N	Y	N	transl_inter	na	NA	intergenic	AC136932.2,LINC0027	-,-	miRNA,lincF	4490;10541	intergenic	PCMTD1P1,CDC27P2	+,-	pseudogene	5756;10414
3190	chr16	33950541	-	chrY	10017107	-	N	N	N	Y	N	N	transl_inter	na	NA	intergenic	AC136932.2,LINC0027	-,-	miRNA,lincF	4520;10511	intergenic	PCMTD1P1,CDC27P2	+,-	pseudogene	5291;10879
3191	chr16	33950602	-	chrY	10017055	-	N	N	N	N	N	Y	transl_inter	na	NA	intergenic	AC136932.2,LINC0027	-,-	miRNA,lincF	4581;10450	intergenic	PCMTD1P1,CDC27P2	+,-	pseudogene	5239;10931
3192	chr16	33950675	-	chrY	10017099	-	N	Y	N	N	N	N	transl_inter	na	NA	intergenic	AC136932.2,LINC0027	-,-	miRNA,lincF	4654;10377	intergenic	PCMTD1P1,CDC27P2	+,-	pseudogene	5283;10887
3193	chr16	33953116	-	chrY	10019602	-	N	N	N	Y	N	N	transl_inter	na	NA	intergenic	AC136932.2,LINC0027	-,-	miRNA,lincF	7095;7936	intergenic	PCMTD1P1,CDC27P2	+,-	pseudogene	7786;8384
3194	chr16	33953161	-	chrY	10019557	-	N	N	N	N	Y	N	transl_inter	na	NA	intergenic	AC136932.2,LINC0027	-,-	miRNA,lincF	7140;7891	intergenic	PCMTD1P1,CDC27P2	+,-	pseudogene	7741;8429
3195	chr16	33954658	-	chrY	10021427	-	N	N	Y	N	N	N	transl_inter	na	NA	intergenic	AC136932.2,LINC0027	-,-	miRNA,lincF	8637;6394	intergenic	PCMTD1P1,CDC27P2	+,-	pseudogene	9611;6559
3196	chr16	33961219	-	chrY	10033630	-	Y	Y	Y	Y	N	N	transl_inter	na	NA	ncRNA_exc	LINC00273	-	lincRNA	0	upstream	AC010970.1	+	miRNA	0
3197	chr16	33964336	-	chrY	10036688	-	Y	N	N	N	N	N	transl_inter	na	NA	intergenic	LINC00273,RNA5-8SF	-,+	lincRNA,rRI	1833;1090	ncRNA_exc	AC010970.2	-	pseudogene	0
3198	chr16	33964358	-	chrY	10036710	-	N	Y	Y	N	N	N	transl_inter	na	NA	intergenic	LINC00273,RNA5-8SF	-,+	lincRNA,rRI	1855;1068	ncRNA_exc	AC010970.2	-	pseudogene	0
3199	chr16	33965511	-	chrY	10037976	-	Y	N	N	N	N	N	transl_inter	na	NA	ncRNA_exc	RNA5-8SP2	+	rRNA	0	downstream	RNA5-8SP6	+	rRNA	0
3200	chr16	33965786	-	chrY	10038130	-	N	N	Y	N	N	N	transl_inter	na	NA	downstream	RNA5-8SP2	+	rRNA	0	downstream	RNA5-8SP6	+	rRNA	0
3201	chr16	34508166	-	chrY	14479141	-	Y	Y	N	N	Y	N	transl_inter	na	NA	ncRNA_intr	RP11-488I20.8	+	lincRNA	0	ncRNA_intr	GYG2P1,ARSDP1	-,+	pseudogene	0
3202	chr16	34508308	-	chrY	14479326	-	N	N	N	Y	N	N	transl_inter	na	NA	ncRNA_intr	RP11-488I20.8	+	lincRNA	0	ncRNA_intr	GYG2P1,ARSDP1	-,+	pseudogene	0
3203	chr16	34508332	-	chrY	14479350	-	N	N	Y	N	N	N	transl_inter	na	NA	ncRNA_intr	RP11-488I20.8	+	lincRNA	0	ncRNA_intr	GYG2P1,ARSDP1	-,+	pseudogene	0
3204	chr16	35135870	-	chrY	14482278	-	Y	Y	Y	N	Y	Y	transl_inter	na	NA	upstream	AC116553.1	+	miRNA	0	ncRNA_intr	GYG2P1,ARSDP1	-,+	pseudogene	0
3205	chr16	33901117	+	chrY	13451808	+	N	N	N	N	Y	Y	transl_inter	na	NA	intergenic;ir	RP11-598D12.2,CTD-2	+,+	pseudogene	47289;21820	intergenic	AC134878.1,DUX4L16	-,+	miRNA,pse	111175;10786
3206	chr16	33901117	+	chrY	13451832	+	Y	N	N	N	N	N	transl_inter	na	NA	intergenic;ir	RP11-598D12.2,CTD-2	+,+	pseudogene	47289;21820	intergenic	AC134878.1,DUX4L16	-,+	miRNA,pse	111199;10762
3207	chr16	33950513	+	chrY	10017313	+	Y	N	Y	N	Y	Y	transl_inter	na	NA	intergenic	AC136932.2,LINC0027	-,-	miRNA,lincF	4492;10539	intergenic	PCMTD1P1,CDC27P2	+,-	pseudogene	5497;10673
3208	chr16	33998847	+	chrY	10065417	+	N	N	N	N	Y	N	transl_inter	na	NA	intergenic	RNA5-8SP2,CTD-2144	+,-	rRNA,lincRI	33270;214268	intergenic	RNA5-8SP6,NONE	+,NONE	rRNA,NONE	27502;NONE
3209	chr17	25279199	-	chr18	15208546	+	N	N	N	N	N	Y	transl_inter	na	NA	intergenic;ir	NONE,RP11-260A9.1	NONE,+	NONE,pseu	NONE;29521	intergenic	RP11-454P7.3,BNIP3P3	-,-	pseudogene	10772;38882
3210	chr17	25279359	-	chr18	15208650	+	N	N	N	N	Y	N	transl_inter	na	NA	intergenic	NONE,RP11-260A9.1	NONE,+	NONE,pseu	NONE;29361	intergenic	RP11-454P7.3,BNIP3P3	-,-	pseudogene	10876;38778
3211	chr17	25290257	-	chr18	15197856	+	Y	N	Y	Y	N	Y	transl_inter	na	NA	intergenic	NONE,RP11-260A9.1	NONE,+	NONE,pseu	NONE;18463	upstream	RP11-454P7.3	-	pseudogene	0
3212	chr17	40846523	-	chr18	3111053	+	N	N	Y	N	N	Y	transl_inter	na	NA	ncRNA_intr	CTD-3193K9.3	-	antisense	0	intronic	MYOM1	-	protein_cod	0
3213	chr17	25279199	+	chr18	15208572	-	N	N	N	N	Y	N	transl_inter	na	NA	intergenic;ir	NONE,RP11-260A9.1	NONE,+	NONE,pseu	NONE;29521	intergenic	RP11-454P7.3,BNIP3P3	-,-	pseudogene	10798;38856
3214	chr17	25279333	+	chr18	15208858	-	N	Y	N	N	N	N	transl_inter	na	NA	intergenic	NONE,RP11-260A9.1	NONE,+	NONE,pseu	NONE;29387	intergenic	RP11-454P7.3,BNIP3P3	-,-	pseudogene	11084;38570
3215	chr17	25279358	+	chr18	15208713	-	Y	N	N	Y	N	N	transl_inter	na	NA	intergenic	NONE,RP11-260A9.1	NONE,+	NONE,pseu	NONE;29362	intergenic	RP11-454P7.3,BNIP3P3	-,-	pseudogene	10939;38715
3216	chr17	25282476	+	chr18	15205218	-	N	N	N	N	N	Y	transl_inter	na	NA	intergenic	NONE,RP11-260A9.1	NONE,+	NONE,pseu	NONE;26244	intergenic	RP11-454P7.3,BNIP3P3	-,-	pseudogene	7444;42210
3217	chr17	25289931	+	chr18	15198276	-	N	N	N	N	Y	N	transl_inter	na	NA	intergenic	NONE,RP11-260A9.1	NONE,+	NONE,pseu	NONE;18789	upstream	RP11-454P7.3	-	pseudogene	0
3218	chr17	25289998	+	chr18	15197896	-	N	N	N	Y	N	N	transl_inter	na	NA	intergenic	NONE,RP11-260A9.1	NONE,+	NONE,pseu	NONE;18722	upstream	RP11-454P7.3	-	pseudogene	0
3219	chr17	81194938	-	chr19	59118540	-	N	N	Y	N	N	N	transl_inter	na	NA	intergenic	AC139099.3,NONE	+,NONE	pseudogene	6365;NONE	intergenic	CENPBD1P1,NONE	+,NONE	pseudogene	7372;NONE
3220	chr17	81195449	-	chr19	59118920	-	Y	N	N	N	N	N	transl_inter	na	NA	intergenic	AC139099.3,NONE	+,NONE	pseudogene	6876;NONE	intergenic	CENPBD1P1,NONE	+,NONE	pseudogene	7752;NONE
3221	chr17	4972484	+	chr19	53676640	+	N	N	N	Y	N	Y	transl_inter	na	NA	intergenic	RP11-46I8.1,RP11-46I	+,-	pseudogene	7066;6489	intronic	ZNF665	-	protein_cod	0
3222	chr17	4972527	+	chr19	53676676	+	N	N	Y	N	N	N	transl_inter	na	NA	intergenic	RP11-46I8.1,RP11-46I	+,-	pseudogene	7109;6446	intronic	ZNF665	-	protein_cod	0
3223	chr17	51286189	-	chr2	195227034	-	N	N	N	N	Y	Y	transl_inter	na	NA	intergenic;ir	RP11-750B16.1,RP11-	+,-	pseudogene	102470;144599	ncRNA_intr	AC018799.1	+	lincRNA	0
3224	chr17	74377420	-	chr2	153997626	-	N	Y	Y	N	Y	N	transl_inter	na	NA	intronic	PRPSAP1,SPHK1	-,+	protein_cod	0	intergenic	UBQLN4P2,ATP5F1P4	+,+	pseudogene	263345;17826
3225	chr17	25290221	-	chr2	132795565	+	Y	Y	Y	Y	Y	N	transl_inter	na	NA	intergenic	NONE,RP11-260A9.1	NONE,+	NONE,pseu	NONE;18499	ncRNA_exc	AC093787.1	-	pseudogene	0
3226	chr17	19995620	+	chr2	209033721	-	Y	Y	Y	Y	Y	N	transl_inter	na	NA	intronic;intrc	SPECC1	+	protein_cod	0	intronic;intrc	C2orf80	-	protein_cod	0
3227	chr17	28863967	+	chr2	25857285	-	N	N	N	N	Y	N	transl_inter	na	NA	ncRNA_intr	ALOX12P1	+	pseudogene	0	intronic	DTNB	-	protein_cod	0
3228	chr17	28863976	+	chr2	25857250	-	Y	N	N	N	N	N	transl_inter	na	NA	ncRNA_intr	ALOX12P1	+	pseudogene	0	intronic	DTNB	-	protein_cod	0
3229	chr17	38601161	+	chr2	62957728	+	N	Y	N	N	N	N	transl_inter	na	NA	intronic	IGFBP4	+	protein_cod	0	intronic	EHBP1	+	protein_cod	0
3230	chr17	38601170	+	chr2	62957482	+	N	N	N	N	Y	N	transl_inter	na	NA	intronic	IGFBP4	+	protein_cod	0	intronic	EHBP1	+	protein_cod	0
3231	chr17	38601663	+	chr2	62957975	+	Y	N	N	N	N	N	transl_inter	na	NA	intronic	IGFBP4	+	protein_cod	0	intronic	EHBP1	+	protein_cod	0
3232	chr17	48381716	+	chr2	238128212	+	Y	N	Y	Y	Y	Y	transl_inter	na	NA	intergenic	RP11-893F2.9,XYLT2	-,+	antisense,p	16500;41737	intergenic	AC107079.1,AC112715.2	-,+	lincRNA,pro	95472;37522
3233	chr17	51286189	+	chr2	195227034	+	Y	N	N	Y	N	N	transl_inter	na	NA	intergenic;ir	RP11-750B16.1,RP11-	+,-	pseudogene	102470;144599	ncRNA_intr	AC018799.1	+	lincRNA	0
3234	chr17	74377273	+	chr2	153997622	+	N	Y	Y	N	Y	N	transl_inter	na	NA	intronic	PRPSAP1,SPHK1	-,+	protein_cod	0	intergenic	UBQLN4P2,ATP5F1P4	+,+	pseudogene	263341;17830
3235	chr17	62410760	+	chr20	15006367	+	Y	Y	Y	Y	Y	Y	transl_inter	na	NA	intergenic	RPL31P57,MILR1	-,+	pseudogene	24710;50809	intronic	MACROD2	+	protein_cod	0
3236	chr17	79499112	+	chr20	60957317	+	N	N	N	N	N	Y	transl_inter	na	NA	intronic	FSCN2	+	protein_cod	0	intergenic	LAMA5,RPS21	-,+	protein_cod	14949;4855
3237	chr17	25277254	-	chr21	14525540	-	N	N	N	N	Y	N	transl_inter	na	NA	intergenic	NONE,RP11-260A9.1	NONE,+	NONE,pseu	NONE;31466	intergenic	AJ239321.3,FGF7P2	+,+	pseudogene	32412;196046
3238	chr17	41465773	-	chr3	73159850	+	N	N	Y	N	N	Y	transl_inter	na	NA	ncRNA_exc	LINC00910	-	lincRNA	0	upstream;u	RNU2-64P	+	snRNA	0
3239	chr17	41467150	+	chr3	46877492	-	N	Y	N	N	N	N	transl_inter	na	NA	upstream	LINC00910	-	lincRNA	0	intergenic;ir	PRSS42,MYL3	-,-	protein_cod	1907;21870
3240	chr17	41467210	+	chr3	46877492	-	Y	N	N	N	N	N	transl_inter	na	NA	upstream	LINC00910	-	lincRNA	0	intergenic;ir	PRSS42,MYL3	-,-	protein_cod	1907;21870
3241	chr17	41401166	+	chr3	46877154	+	Y	Y	N	Y	N	Y	transl_inter	na	NA	intergenic	LINC00854,LINC0091(	-,-	processed_	17828;46047	intergenic	PRSS42,MYL3	-,-	protein_cod	1569;22208
3242	chr17	4287308	-	chr4	54192535	-	Y	N	N	N	Y	N	transl_inter	na	NA	intergenic	UBE2G1,SPNS3	-,+	protein_cod	17385;49675	intronic	SCFD2	-	protein_cod	0
3243	chr17	33007470	-	chr4	20887010	-	N	N	Y	N	Y	N	transl_inter	na	NA	intergenic	TMEM132E,RP11-212I	+,-	protein_cod	41133;45058	intronic	KCNIP4	-	protein_cod	0
3244	chr17	3802531	-	chr5	32418333	-	Y	N	Y	Y	Y	Y	transl_inter	na	NA	intronic	P2RX1	-	protein_cod	0	intronic	ZFR	-	protein_cod	0
3245	chr17	16031093	-	chr5	163756291	-	N	N	N	Y	N	N	transl_inter	na	NA	intronic	NCOR1	-	protein_cod	0	ncRNA_intr	CTC-340A15.2	+	antisense	0
3246	chr17	39300420	+	chr5	58239397	+	Y	Y	Y	N	Y	Y	transl_inter	na	NA	intergenic	KRTAP4-6,KRTAP4-5	-,-	protein_cod	3681;4758	intergenic	CTD-2176I21.2,PDE4D	-,-	antisense,p	80156;25468
3247	chr17	26192126	-	chr6	95680488	-	N	N	N	N	Y	N	transl_inter	na	NA	intronic	RP1-66C13.4	-	protein_cod	0	intergenic	RP3-399J4.1,CYCSP17	-,+	pseudogene	523591;271570
3248	chr17	26192145	-	chr6	95680544	-	N	Y	N	N	N	Y	transl_inter	na	NA	intronic	RP1-66C13.4	-	protein_cod	0	intergenic	RP3-399J4.1,CYCSP17	-,+	pseudogene	523647;271514
3249	chr17	40549456	-	chr6	21125688	-	N	N	N	Y	N	N	transl_inter	na	NA	intergenic	STAT3,AC107993.1	-,+	protein_cod	8870;1571	intronic	CDKAL1	+	protein_cod	0
3250	chr17	40549500	-	chr6	21125842	-	N	N	N	N	N	Y	transl_inter	na	NA	intergenic	STAT3,AC107993.1	-,+	protein_cod	8914;1527	intronic	CDKAL1	+	protein_cod	0
3251	chr17	41379304	-	chr6	27162797	+	Y	N	N	N	N	Y	transl_inter	na	NA	ncRNA_intr	LINC00854	-	processed_	0	intergenic	MIR3143,RP11-209A2.1	+,+	miRNA,pse	47330;16226
3252	chr17	41379304	-	chr6	27162865	+	N	Y	N	N	N	N	transl_inter	na	NA	ncRNA_intr	LINC00854	-	processed_	0	intergenic	MIR3143,RP11-209A2.1	+,+	miRNA,pse	47398;16158
3253	chr17	41467151	+	chr6	27163561	-	N	Y	Y	Y	Y	Y	transl_inter	na	NA	upstream	LINC00910	-	lincRNA	0	intergenic	MIR3143,RP11-209A2.1	+,+	miRNA,pse	48094;15462
3254	chr17	41401173	+	chr6	27162840	+	N	N	Y	N	N	N	transl_inter	na	NA	intergenic;ir	LINC00854,LINC0091(	-,-	processed_	17835;46040	intergenic	MIR3143,RP11-209A2.1	+,+	miRNA,pse	47373;16183
3255	chr17	21303090	-	chr7	57712325	-	Y	Y	Y	Y	Y	Y	transl_inter	na	NA	intronic	KCNJ12	+	protein_cod	0	upstream;d	RP11-368M16.6,RP11-36	+,-	pseudogene	0
3256	chr17	21532097	-	chr7	57655988	-	N	Y	Y	N	N	Y	transl_inter	na	NA	intergenic	RP11-822E23.5,NCOR	+,+	pseudogene	34651;3917	intergenic	RP11-1324A7.2,NCOR1F	+,+	pseudogene	110387;3512
3257	chr17	16072319	-	chr7	57665790	+	N	N	Y	N	Y	Y	transl_inter	na	NA	intronic	NCOR1	-	protein_cod	0	ncRNA_intr	NCOR1P3	+	pseudogene	0
3258	chr17	16738686	-	chr7	48029723	+	N	N	N	N	Y	N	transl_inter	na	NA	intergenic	KRT16P2,KRT17P1	-,-	pseudogene	2539;5371	intronic	SUN3	-	protein_cod	0
3259	chr17	25286130	-	chr7	57932723	+	N	N	N	N	Y	N	transl_inter	na	NA	intergenic	NONE,RP11-260A9.1	NONE,+	NONE,pseu	NONE;22590	intergenic	RP11-548K12.12,NONE	+,NONE	pseudogene	35971;NONE
3260	chr17	25300225	-	chr7	64962398	+	N	N	N	Y	N	N	transl_inter	na	NA	intergenic	NONE,RP11-260A9.1	NONE,+	NONE,pseu	NONE;8495	intergenic	RP11-667F9.2,RP11-328	+,-	lincRNA,pse	34047;11288
3261	chr17	48118482	-	chr7	95846742	+	Y	Y	Y	Y	Y	Y	transl_inter	na	NA	intergenic	RP11-1094H24.3,RP1	+,-	lincRNA,linc	24274;9231	intronic	SLC25A13	-	protein_cod	0
3262	chr17	20782775	+	chr7	57628264	-	N	Y	N	N	N	N	transl_inter	na	NA	ncRNA_intr	RP11-344E13.3	+	antisense	0	intergenic	RP11-1324A7.2,NCOR1F	+,+	pseudogene	82663;31236
3263	chr17	25298029	+	chr7	57925864	-	N	N	N	N	Y	N	transl_inter	na	NA	intergenic	NONE,RP11-260A9.1	NONE,+	NONE,pseu	NONE;10691	intergenic	RP11-548K12.12,NONE	+,NONE	pseudogene	29112;NONE
3264	chr17	25298154	+	chr7	57925865	-	N	N	Y	N	N	N	transl_inter	na	NA	intergenic	NONE,RP11-260A9.1	NONE,+	NONE,pseu	NONE;10566	intergenic	RP11-548K12.12,NONE	+,NONE	pseudogene	29113;NONE
3265	chr17	25298172	+	chr7	57925914	-	N	Y	N	N	N	N	transl_inter	na	NA	intergenic	NONE,RP11-260A9.1	NONE,+	NONE,pseu	NONE;10548	intergenic	RP11-548K12.12,NONE	+,NONE	pseudogene	29162;NONE
3266	chr17	25304658	+	chr7	61078846	-	N	N	Y	N	N	N	transl_inter	na	NA	intergenic	NONE,RP11-260A9.1	NONE,+	NONE,pseu	NONE;4062	intergenic	NONE,RP11-715L17.1	NONE,+	NONE,pseu	NONE;743023
3267	chr17	25304730	+	chr7	61078732	-	N	Y	N	N	Y	N	transl_inter	na	NA	intergenic	NONE,RP11-260A9.1	NONE,+	NONE,pseu	NONE;3990	intergenic	NONE,RP11-715L17.1	NONE,+	NONE,pseu	NONE;743137
3268	chr17	25305283	+	chr7	61078709	-	N	N	N	N	N	Y	transl_inter	na	NA	intergenic	NONE,RP11-260A9.1	NONE,+	NONE,pseu	NONE;3437	intergenic	NONE,RP11-715L17.1	NONE,+	NONE,pseu	NONE;743160
3269	chr17	25307096	+	chr7	57917134	-	N	N	N	N	Y	N	transl_inter	na	NA	intergenic	NONE,RP11-260A9.1	NONE,+	NONE,pseu	NONE;1624	intergenic	RP11-548K12.12,NONE	+,NONE	pseudogene	20382;NONE
3270	chr17	21514739	+	chr7	57637907	+	Y	N	N	N	N	N	transl_inter	na	NA	intergenic	RP11-822E23.5,NCOR	+,+	pseudogene	17293;21275	intergenic	RP11-1324A7.2,NCOR1F	+,+	pseudogene	92306;21593

3271	chr17	25274659	+	chr7	61666149	+	Y	N	N	N	N	Y	transl_inter	na	NA	intergenic;ir	NONE,RP11-260A9.1	NONE,+	NONE,pseu	NONE;34061	intergenic;ir	NONE,RP11-715L17.1	NONE,+	NONE,pseu	NONE;155720
3272	chr17	16071617	-	chr8	3615589	-	N	N	N	N	Y	N	transl_inter	na	NA	intronic	NCOR1	-	protein_cod	0	intronic	CSMD1	-	protein_cod	0
3273	chr17	39300399	-	chr8	9387283	+	Y	N	Y	Y	Y	Y	transl_inter	na	NA	intergenic	KRTAP4-6,KRTAP4-5	-,-	protein_cod	3660;4779	intergenic	RP11-375N15.1,RP11-37	+,-	pseudogene	72355;25373
3274	chr17	41401148	+	chr9	140405606	-	Y	Y	Y	Y	Y	Y	transl_inter	na	NA	intergenic	LINC00854,LINC0091(	-,-	processed_	17810;46065	intronic	PNPLA7	-	protein_cod	0
3275	chr17	38296616	+	chr9	90186340	+	Y	N	N	N	N	N	transl_inter	na	NA	UTR5	CASC3	+	protein_cod	0	intronic	DAPK1	+	protein_cod	0
3276	chr17	45209251	+	chr9	37062234	+	N	N	N	Y	Y	Y	transl_inter	na	NA	intronic	CDC27	-	protein_cod	0	intergenic	RP11-297B17.2,RP11-46	+,-	pseudogene	15000;11294
3277	chr17	41401173	+	chrY	17224393	+	Y	Y	Y	Y	Y	Y	transl_inter	na	NA	intergenic;ir	LINC00854,LINC0091(	-,-	processed_	17835;46040	intergenic	AC006989.2,HDHD1P1	+,-	pseudogene	169798;236149
3278	chr18	15198100	-	chr19	33444226	-	Y	N	N	N	N	N	transl_inter	na	NA	upstream	RP11-454P7.3	-	pseudogene	0	intronic	CEP89	-	protein_cod	0
3279	chr18	23467604	-	chr2	209970999	-	Y	N	N	Y	Y	N	transl_inter	na	NA	intergenic	RN7SL97P,RP11-737C	-,-	misc_RNA,p	69108;65576	intergenic	HSPA8P6,CRYGFP	-,+	pseudogene	32127;38966
3280	chr18	23468010	-	chr2	209971149	-	N	N	N	N	N	Y	transl_inter	na	NA	intergenic	RN7SL97P,RP11-737C	-,-	misc_RNA,p	69514;65170	intergenic	HSPA8P6,CRYGFP	-,+	pseudogene	32277;38816
3281	chr18	29387393	-	chr2	165156024	-	Y	N	N	N	N	N	transl_inter	na	NA	intergenic	SLC25A52,TRAPPC8	-,-	protein_cod	46550;21743	ncRNA_intro	AC092684.1	-	lincRNA	0
3282	chr18	47205536	-	chr2	166888711	+	N	N	Y	N	N	N	transl_inter	na	NA	intergenic	SMUG1P1,RP11-813F	+,-	pseudogene	28006;60657	ncRNA_intro	AC010127.3	+	antisense	0
3283	chr18	46156415	+	chr2	138747359	-	N	Y	Y	Y	Y	Y	transl_inter	na	NA	intronic	CTIF	+	protein_cod	0	intronic	HNMT	+	protein_cod	0
3284	chr18	73702325	+	chr2	207688320	-	N	N	Y	N	N	N	transl_inter	na	NA	intergenic	RP11-173L6.1,RP11-3:	-,-	lincRNA,linc	122118;17748	intergenic	RP11-95H11.1,AC00826(	-,+	lincRNA,linc	1302;43199
3285	chr18	28338774	+	chr2	84141818	+	N	N	Y	N	N	N	transl_inter	na	NA	intergenic	RP11-675P14.1,RP11-	+,-	lincRNA,linc	44522;196012	intergenic	AC104395.1,AC016908.1	-,+	pseudogene	36141;116446
3286	chr18	29387427	+	chr2	165155994	+	Y	N	Y	Y	N	N	transl_inter	na	NA	intergenic	SLC25A52,TRAPPC8	-,-	protein_cod	46584;21709	ncRNA_intro	AC092684.1	-	lincRNA	0
3287	chr18	47337315	+	chr2	41973164	+	Y	N	Y	N	N	N	transl_inter	na	NA	intronic	ACAA2,RP11-886H22.	-,-	protein_cod	0	intergenic	snoZ247,LDHAP3	+,+	snoRNA,ps(	11568;73723
3288	chr18	12087235	-	chr21	15358108	-	N	N	N	N	N	Y	transl_inter	na	NA	intergenic	RP11-815J4.1,RP11-8	+,-	lincRNA,pse	1969;4438	downstream	AP001634.1	+	miRNA	0
3289	chr18	14860313	-	chr21	14751201	+	Y	N	N	N	N	N	transl_inter	na	NA	intergenic	ANKRD30B,RP11-527I	+,+	protein_cod	7834;17297	intergenic	AL050302.1,ANKRD30BI	-,-	protein_cod	5815;5369
3290	chr18	12980004	-	chr22	41690895	+	N	N	N	N	N	Y	transl_inter	na	NA	intronic	SEH1L	+	protein_cod	0	intergenic	AL035681.1,ZC3H7B	-,+	protein_cod	5209;6631
3291	chr18	13691906	-	chr4	155069745	+	N	N	Y	N	N	N	transl_inter	na	NA	intronic	FAM210A	-	protein_cod	0	intergenic	AC020703.1,AC079298.1	-,+	miRNA,miR	275142;70316
3292	chr18	28136585	-	chr5	122104086	-	N	N	N	N	N	Y	transl_inter	na	NA	intergenic	RP11-784B15.1,RP11-	+,+	lincRNA,linc	1008379;156656	intergenic	RP11-166A12.1,SNX2	-,+	lincRNA,pro	37706;6605
3293	chr18	46198363	-	chr5	177595868	-	Y	N	N	N	N	N	transl_inter	na	NA	intronic	CTIF	+	protein_cod	0	intergenic	RP11-889L3.4,GMCL1P1	+,-	pseudogene	2305;16852
3294	chr18	55381573	+	chr5	175733409	-	N	Y	N	N	N	Y	transl_inter	na	NA	ncRNA_intro	RP11-35G9.3,RP11-35	+,+	antisense,a	0	intronic;intro	SIMC1	+	protein_cod	0
3295	chr18	55381819	+	chr5	175733390	-	Y	N	N	Y	N	N	transl_inter	na	NA	ncRNA_intro	RP11-35G9.3,RP11-35	+,+	antisense,a	0	intronic	SIMC1	+	protein_cod	0
3296	chr18	66572501	+	chr5	78731981	-	Y	N	Y	Y	N	Y	transl_inter	na	NA	intronic	CCDC102B	+	protein_cod	0	intronic	HOMER1	-	protein_cod	0
3297	chr18	30668455	+	chr5	32974596	+	Y	N	N	N	N	N	transl_inter	na	NA	intronic	CCDC178	-	protein_cod	0	ncRNA_intro	CTD-2066L21.3	-	lincRNA	0
3298	chr18	30668703	+	chr5	32974588	+	N	N	Y	Y	N	N	transl_inter	na	NA	intronic	CCDC178	-	protein_cod	0	ncRNA_intro	CTD-2066L21.3	-	lincRNA	0
3299	chr18	30668724	+	chr5	32974588	+	N	Y	N	N	Y	Y	transl_inter	na	NA	intronic	CCDC178	-	protein_cod	0	ncRNA_intro	CTD-2066L21.3	-	lincRNA	0
3300	chr18	9980081	-	chr6	96945634	-	N	N	N	Y	N	N	transl_inter	na	NA	intergenic	VAPA,AP005271.1	+,-	protein_cod	20063;25016	ncRNA_intro	UFL1-AS1	-	antisense	0
3301	chr18	11806088	+	chr6	149425271	-	N	N	N	Y	N	N	transl_inter	na	NA	intronic	GNAL	+	protein_cod	0	intergenic	UST,RP11-365H23.1	+,-	protein_cod	27145;7874
3302	chr18	39770209	+	chr6	153748714	-	N	Y	N	N	Y	Y	transl_inter	na	NA	ncRNA_intro	LINC00907	+	lincRNA	0	intergenic	RNA5SP225,RP11-331O	+,+	rRNA,pseuc	7007;238128
3303	chr18	29384591	-	chr7	109236787	+	N	N	N	N	Y	N	transl_inter	na	NA	intergenic	SLC25A52,TRAPPC8	-,-	protein_cod	43748;24545	ncRNA_intro	AC073071.1	-	lincRNA	0
3304	chr18	29384761	-	chr7	109236696	+	N	N	N	N	N	Y	transl_inter	na	NA	intergenic	SLC25A52,TRAPPC8	-,-	protein_cod	43918;24375	ncRNA_intro	AC073071.1	-	lincRNA	0
3305	chr18	77780755	-	chr7	63971332	+	Y	N	Y	Y	Y	Y	transl_inter	na	NA	intronic	TXNL4A	-	protein_cod	0	intergenic	HNRNPCP7,ZNF680	+,-	pseudogene	9225;8930
3306	chr18	22222853	-	chr8	11099039	+	N	N	Y	N	N	Y	transl_inter	na	NA	ncRNA_intro	RP11-449D8.1	+	lincRNA	0	intergenic	AF131215.8,LINC00529	+,-	lincRNA,linc	37859;6096
3307	chr18	3572482	+	chr8	134325214	-	Y	N	N	Y	Y	N	transl_inter	na	NA	intronic	DLGAP1	-	protein_cod	0	intergenic	NDRG1,CTC-458A3.1	-,+	protein_cod	10949;13026
3308	chr18	22222371	+	chr8	11098987	-	N	N	Y	Y	N	N	transl_inter	na	NA	ncRNA_intro	RP11-449D8.1	+	lincRNA	0	intergenic;ir	AF131215.8,LINC00529	+,-	lincRNA,linc	37807;6148
3309	chr18	21867768	-	chr9	818000	-	N	N	N	N	Y	N	transl_inter	na	NA	intronic	OSBPL1A	-	protein_cod	0	intergenic	KANK1,DMRT1	+,+	protein_cod	71895;23690
3310	chr18	49492	+	chr9	141069873	-	N	N	N	N	N	Y	transl_inter	na	NA	intronic	RP11-683L23.1	-	protein_cod	0	ncRNA_exo	TUBBP5	+	pseudogene	0
3311	chr18	49556	+	chr9	141069873	-	Y	N	Y	N	Y	N	transl_inter	na	NA	exonic	RP11-683L23.1	-	protein_cod	0	ncRNA_exo	TUBBP5	+	pseudogene	0
3312	chr18	49577	+	chr9	141069873	-	N	N	N	Y	N	N	transl_inter	na	NA	intronic	RP11-683L23.1	-	protein_cod	0	ncRNA_exo	TUBBP5	+	pseudogene	0
3313	chr18	9277339	+	chr9	68434050	-	N	Y	N	N	N	N	transl_inter	na	NA	intronic	ANKRD12	+	protein_cod	0	ncRNA_intro	RP11-764K9.4	-	pseudogene	0
3314	chr18	71711514	+	chr9	127885509	-	Y	Y	N	Y	Y	Y	transl_inter	na	NA	intergenic;ir	RP11-25L3.3,FBXO15	-,-	lincRNA,pro	10118;29074	intronic;intro	SCAI	-	protein_cod	0
3315	chr18	15240800	+	chr9	69817517	+	N	N	N	N	N	Y	transl_inter	na	NA	intergenic	RP11-454P7.3,BNIP3P	-,-	pseudogene	43026;6628	intergenic	IGKV1OR-2,AL359955.1	-,-	IG_V_pseu	39564;4243
3316	chr18	40776992	-	chrX	77458203	+	N	Y	N	N	N	N	transl_inter	na	NA	intergenic	RIT2,SYT4	-,-	protein_cod	81335;70851	intergenic	FNDC3CP,CYSLTR1	-,-	pseudogene	30284;68758
3317	chr18	48949793	-	chrX	54282452	+	Y	Y	Y	Y	N	Y	transl_inter	na	NA	ncRNA_intro	RP11-267C16.1	+	lincRNA	0	intronic	WNK3	-	protein_cod	0
3318	chr19	51998626	+	chr2	9916714	+	N	Y	N	N	N	N	transl_inter	na	NA	intronic	SIGLEC12	-	protein_cod	0	intergenic	RP11-521D12.5,TAF1B	-,+	lincRNA,pro	6244;66769
3319	chr19	33444265	-	chr20	26220635	-	N	Y	N	N	N	N	transl_inter	na	NA	intronic;intro	CEP89	-	protein_cod	0	ncRNA_intro	MIR663A	-	processed_	0
3320	chr19	54925721	+	chr21	44756093	+	N	N	N	Y	N	N	transl_inter	na	NA	upstream	TTYH1	+	protein_cod	0	intergenic	LINC00322,AP001046.5	-,-	lincRNA,linc	4174;21934
3321	chr19	53917707	+	chr22	42905848	-	N	N	N	N	Y	Y	transl_inter	na	NA	intronic	ZNF765	+	protein_cod	0	ncRNA_intro	SERHL	+	processed_	0
3322	chr19	53917736	+	chr22	42906257	-	N	Y	Y	N	N	N	transl_inter	na	NA	intronic	ZNF765	+	protein_cod	0	ncRNA_intro	SERHL	+	processed_	0
3323	chr19	19632428	+	chr22	16347242	+	Y	Y	Y	Y	Y	Y	transl_inter	na	NA	intronic	NDUFA13,YJEFN3,CT	+,+,+	protein_cod	0	ncRNA_intro	NF1P6	+	pseudogene	0
3324	chr19	33444265	-	chr3	75983387	+	Y	Y	Y	Y	Y	Y	transl_inter	na	NA	intronic;intro	CEP89	-	protein_cod	0	intronic	ROBO2	+	protein_cod	0
3325	chr19	52453840	-	chr4	137859689	+	N	N	Y	N	N	N	transl_inter	na	NA	ncRNA_intro	HCCAT3	+	antisense	0	ncRNA_intro	RP11-138I17.1	-	lincRNA	0
3326	chr19	28819016	+	chr4	43139160	+	Y	N	N	N	N	N	transl_inter	na	NA	ncRNA_intro	AC005780.1	-	lincRNA	0	ncRNA_intro	RP11-395F4.1	+	lincRNA	0
3327	chr19	1037858	-	chr6	111180310	-	N	N	N	N	N	Y	transl_inter	na	NA	exonic	CNN2	+	protein_cod	0	ncRNA_exo	CNN2P9	+	pseudogene	0
3328	chr19	58938639	-	chr6	113730621	+	N	N	Y	N	N	Y	transl_inter	na	NA	intergenic	CTD-2619J13.16,CTD-	+,-	lincRNA,linc	7962;1360	intergenic	RP11-437J19.1,RP1-124	-,-	lincRNA,linc	41475;19121
3329	chr19	21851909	-	chr7	46080418	-	Y	N	N	N	N	N	transl_inter	na	NA	intergenic	RP11-420K14.1,RP11-	+,-	pseudogene	12807;8443	intergenic	AC073115.7,AC023669.2	+,+	lincRNA,pse	39921;104117
3330	chr19	21851946	-	chr7	46080003	-	N	N	Y	N	N	N	transl_inter	na	NA	intergenic	RP11-420K14.1,RP11-	+,-	pseudogene	12844;8406	intergenic	AC073115.7,AC023669.2	+,+	lincRNA,pse	39506;104532
3331	chr19	23325027	-	chr7	64289940	-	N	N	N	N	N	Y	transl_inter	na	NA	intronic	ZNF730	+	protein_cod	0	intronic	ZNF138	+	protein_cod	0
3332	chr19	27821413	-	chr7	58022015	-	N	N	N	N	N	Y	transl_inter	na	NA	intergenic	NONE,CTB-151G24.1	NONE,-	NONE,lincR	NONE;307978	intergenic	RP11-548K12.12,NONE	+,NONE	pseudogene	125263;NONE
3333	chr19	21480236	-	chr7	64289938	+	N	Y	N	N	Y	Y	transl_inter	na	NA	intronic;intro	ZNF708	-	protein_cod	0	intronic;intro	ZNF138	+	protein_cod	0
3334	chr19	22314335	-	chr7	64289907	+	N	Y	N	N	Y	Y	transl_inter	na	NA	ncRNA_intro	ZNF92P2	-	pseudogene	0	intronic	ZNF138	+	protein_cod	0
3335	chr19	15000136	+	chr7	142014574	-	N	N	N	N	N	Y	transl_inter	na	NA	intergenic	OR7A18P,OR7A1P	-,-	pseudogene	2209;2571	intergenic;ir	TRBV4-1,TRBV5-1	+,+	TR_V_gene	1085;5922
3336	chr19	22201601	+	chr7	64290289	-	N	N	N	Y	N	N	transl_inter	na	NA	ncRNA_intro	AC003973.4	-	lincRNA	0	intronic	ZNF138	+	protein_cod	0
3337	chr19	22201712	+	chr7	64289932	-	N	Y	Y	N	Y	N	transl_inter	na	NA	ncRNA_intro	AC003973.4	-	lincRNA	0	intronic	ZNF138	+	protein_cod	0
3338	chr19	23482124	+	chr7	64891288	-	N	N	Y	Y	Y	Y	transl_inter	na	NA	intergenic	VN1R91P,CTB-176F2(	+,+	pseudogene	8305;24646	intergenic	ZNF92,RP11-667F9.2	+,+	protein_cod	25250;36696
3339	chr19	28310194	+	chr7	64891453	-	N	Y	N	N	N	N	transl_inter	na	NA	ncRNA_intro	CTC-459F4.7,CTC-45(	-,+,+	pseudogene	0	intergenic	ZNF92,RP11-667F9.2	+,+	protein_cod	25415;36531
3340	chr19	28310213	+	chr7	64891432	-	N	N	N	N	N	Y	transl_inter	na	NA	ncRNA_intro	CTC-459F4.7,CTC-45(	-,+,+	pseudogene	0	intergenic	ZNF92,RP11-667F9.2	+,+	protein_cod	25394;36552
3341	chr19	59118820	+	chr7	10476	-	N	N	N	N	N	Y	transl_inter	na	NA	intergenic	CENPBD1P1,NONE	+,NONE	pseudogene	7652;NONE	intergenic	NONE,AC093627.7	NONE,-	NONE,lincR	NONE;60496
3342	chr19	59097929	-	chr8	146300855	-	N	Y	N	N	N	N	transl_inter	na	NA	ncRNA_intro	CENPBD1P1	+	pseudogene	0	intergenic;ir	C8orf33,NONE	+,NONE	protein_cod	19439;NONE
3343	chr19	21871004	-	chr8	14344934	+	Y	Y	Y	Y	Y	N	transl_inter	na	NA	intergenic	RP11-420K14.2,AC092	-,+	pseudogene	9981;9205	intronic	SGCZ	-	protein_cod	0
3344	chr19	56908011	+	chr8	144325532	+	N	N	N	N	Y	Y	transl_inter	na	NA	ncRNA_intro	ZNF582-AS1	+	lincRNA	0	intergenic	GPIHBP1,ZFP41	+,+	protein_cod	26488;3459
3345	chr19	35412022	+	chr9	68418844	-	N	N	N	Y	N	N	transl_inter	na	NA	intergenic	CTC-523E23.6,CTC-5:	+,-	lincRNA,linc	12930;2181	intergenic;ir	MIR4477A,RP11-764K9.4	+,-	miRNA,pse	3456;9017
3346	chr19	23751048	+	chr9	107078713	+	N	N	N	N	Y	N	transl_inter	na	NA	intronic	ZNF675	-	protein_cod	0	intergenic	RP11-86L19.2,RP11-217	+,-	pseudogene	29477;11888
3347	chr19	23751102	+	chr9	107078321	+	Y	N	N	N	N	N	transl_inter	na	NA	intronic	ZNF675	-	protein_cod	0	intergenic	RP11-86L19.2,RP11-217	+,-	pseudogene	29085;12280
3348	chr19	24027922	-	chrX	53604462	+	Y	N	Y	N	Y	N	transl_inter	na	NA	ncRNA_intro	RP11-255H23.4	-	lincRNA	0	intronic	HUWE1	-	protein_cod	0
3349	chr19	24027922	-	chrX	64676300	+	N	N	N	N	N	Y	transl_inter	na	NA	ncRNA_intro	RP11-255H23.4	-	lincRNA	0	intergenic	TLE1P1,RP3-323B6.1	+,+	pseudogene	47735;12667
3350	chr2	15820438	-	chr20	3000167	-	N	N	N	N	Y	N	transl_inter	na	NA	ncRNA_exo	AC008278.3	-	antisense	0	intronic	PTPRA	+	protein_cod	0
3351	chr2	133015030	-	chr20	26189314	-	N	N	N	N	Y	N	transl_inter	na	NA	upstream	MIR663B	-	miRNA	0	ncRNA_intro	MIR663A	-	processed_	0
3352	chr2	208963316	+	chr20	54852855	-	Y	Y	Y	Y	Y	N	transl_inter	na	NA	intergenic	snoU13,CRYGEP	-,-	snoRNA,ps(	24430;9641	intergenic	RP11-380D15.2,RP11-38	+,-	pseudogene	8568;18168
3353	chr2	219874344	+	chr20	47078200	+	Y	N	N	N	N	N	transl_inter	na	NA	ncRNA_intro	AC097468.4	+	antisense	0	intergenic	RP1-66N13.1,RNU7-144	-,-	lincRNA,snF	64562;44280
3354	chr2	237615485	-	chr21	39080026	+	N	N	N	Y	N	N	transl_inter	na	NA	intergenic	ACKR3,AC011286.1	+,+	protein_cod	124484;26540	intronic	KCNJ6	-	protein_cod	0

3355	chr2	237615489	-	chr21	39080014	+	N	Y	N	N	N	N	transl_inter	na	NA	intergenic	ACKR3,AC011286.1	+,+	protein_cod	124488;26536	intronic;intro	KCNJ6	-	protein_cod	0
3356	chr2	237615575	-	chr21	39080014	+	Y	N	Y	N	N	Y	transl_inter	na	NA	intergenic	ACKR3,AC011286.1	+,+	protein_cod	124574;26450	intronic;intro	KCNJ6	-	protein_cod	0
3357	chr2	85714118	-	chr22	49425468	-	Y	N	N	Y	Y	Y	transl_inter	na	NA	intergenic	Y_RNA,AC016753.7	-,-	misc_RNA,p	26743;3935	intergenic	WI2-81516E3.1,RPL35P	+,-	lincRNA,pse	131270;184369
3358	chr2	91725260	-	chr22	22621352	-	N	N	N	N	N	Y	transl_inter	na	NA	intergenic	AC018696.5,AC01869	-,-	pseudogene	1344;8914	intergenic	VPREB1,LL22NC03-2H8	+,-	protein_cod	21425;16960
3359	chr2	138521333	-	chr22	40403419	-	N	Y	N	N	N	N	transl_inter	na	NA	intergenic	Y_RNA,AC020601.1	+,-	misc_RNA,l	48322;114991	intronic	FAM83F	+	protein_cod	0
3360	chr2	138521731	-	chr22	40403480	-	N	N	N	N	Y	N	transl_inter	na	NA	intergenic	Y_RNA,AC020601.1	+,-	misc_RNA,l	48720;114593	intronic	FAM83F	+	protein_cod	0
3361	chr2	47809988	-	chr22	24195862	+	N	N	Y	N	N	Y	transl_inter	na	NA	intergenic	AC138655.1,AC07925	+,-	protein_cod	7990;107867	intergenic	DERL3,KB-1125A3.11	-,-	protein_cod	14663;2752
3362	chr2	92177709	-	chr22	22639645	+	N	N	N	N	Y	N	transl_inter	na	NA	intergenic	CHEK2P3,AC128677.3	+,-	pseudogene	26073;16997	ncRNA_exo	LL22NC03-2H8.5	-	lincRNA	0
3363	chr2	91694631	+	chr22	17048928	-	N	N	N	N	Y	N	transl_inter	na	NA	upstream	AC018696.2	-	pseudogene	0	intergenic	KB-67B5.17,KB-67B5.12	+,+	lincRNA,pse	40801;3989
3364	chr2	174563055	+	chr22	43871887	-	N	N	N	Y	N	N	transl_inter	na	NA	intergenic	AC013410.1,AC01341	-,-	pseudogene	121986;7621	intronic	MPPED1	+	protein_cod	0
3365	chr2	92045115	+	chr22	16883838	+	N	N	Y	N	N	N	transl_inter	na	NA	intergenic	ABCD1P5,AC127391.3	+,+	pseudogene	13287;2295	intergenic	ABCD1P4,LA16c-17H1.3	+,+	pseudogene	12225;2218
3366	chr2	92045320	+	chr22	16883781	+	N	N	N	N	Y	N	transl_inter	na	NA	intergenic	ABCD1P5,AC127391.3	+,+	pseudogene	13492;2090	intergenic	ABCD1P4,LA16c-17H1.3	+,+	pseudogene	12168;2275
3367	chr2	151053655	-	chr3	95544397	-	N	Y	Y	Y	N	Y	transl_inter	na	NA	ncRNA_intro	AC016682.1	+	lincRNA	0	intergenic	MTHFD2P1,AC108739.1	-,-	pseudogene	142360;341264
3368	chr2	5628590	+	chr3	27992677	-	N	N	N	N	Y	N	transl_inter	na	NA	intergenic	AC073143.1,AC10705	-,-	pseudogene	174323;61322	intergenic	AC098973.2,AC092415.1	+,-	lincRNA,linc	59885;46024
3369	chr2	5628684	+	chr3	27992659	-	Y	Y	Y	Y	N	Y	transl_inter	na	NA	intergenic	AC073143.1,AC10705	-,-	pseudogene	174417;61228	intergenic	AC098973.2,AC092415.1	+,-	lincRNA,linc	59867;46042
3370	chr2	41453302	+	chr3	83756618	-	Y	Y	N	N	N	N	transl_inter	na	NA	intergenic	HNRNPA1P57,AC0107	+,+	pseudogene	68607;370224	intergenic	CYP51A1P1,SRRM1P2	-,+	pseudogene	899446;229578
3371	chr2	160148933	+	chr3	58772449	-	Y	N	N	N	N	N	transl_inter	na	NA	intergenic	WDSUB1,BAZ2B	-,-	protein_cod	5623;26557	intronic	C3orf67	-	protein_cod	0
3372	chr2	5628691	+	chr3	140373031	+	N	N	N	N	N	Y	transl_inter	na	NA	intergenic;ir	AC073143.1,AC10705	-,-	pseudogene	174424;61221	intergenic	RP11-13L2.1,TRIM42	-,+	pseudogene	34352;23850
3373	chr2	223680378	+	chr3	149962939	+	N	N	N	Y	N	N	transl_inter	na	NA	intergenic	AC016712.2,ACSL3	+,+	pseudogene	88825;45274	intergenic	RP11-167H9.6,RP11-483	+,-	lincRNA,linc	4943;20260
3374	chr2	26556299	-	chr4	90644970	-	N	N	N	N	N	Y	transl_inter	na	NA	intronic;intro	EPT1,GPR113	+,-	protein_cod	0	ncRNA_intro	RP11-115D19.1	+	antisense	0
3375	chr2	26556299	-	chr4	130703268	-	Y	Y	Y	Y	Y	Y	transl_inter	na	NA	intronic;intro	EPT1,GPR113	+,-	protein_cod	0	ncRNA_intro	RP11-422J15.1	+	lincRNA	0
3376	chr2	27611576	-	chr4	80704775	-	Y	Y	Y	N	N	Y	transl_inter	na	NA	intronic	PPM1G	-	protein_cod	0	intergenic	RP11-452C8.1,PCAT4	+,+	lincRNA,linc	86784;43850
3377	chr2	228185886	-	chr4	127411743	-	N	N	Y	N	N	N	transl_inter	na	NA	ncRNA_intro	AC097662.2	-	antisense	0	intergenic	RP11-318I4.2,RBM48P1	-,+	pseudogene	383665;72921
3378	chr2	228186046	-	chr4	127412018	-	N	N	N	N	N	Y	transl_inter	na	NA	ncRNA_intro	AC097662.2	-	antisense	0	intergenic	RP11-318I4.2,RBM48P1	-,+	pseudogene	383940;72646
3379	chr2	14414570	-	chr4	36298642	+	Y	N	N	N	N	N	transl_inter	na	NA	ncRNA_intro	LINC00276	-	lincRNA	0	intronic	DTHD1	+	protein_cod	0
3380	chr2	37608440	-	chr4	11808084	+	N	N	N	N	N	Y	transl_inter	na	NA	intergenic	QPCT,RNU6-1116P	+,-	protein_cod	7975;54213	ncRNA_intro	RP11-281P23.2	+	lincRNA	0
3381	chr2	116695706	+	chr4	39128560	-	Y	Y	Y	Y	Y	Y	transl_inter	na	NA	intergenic	DPP10,RP11-338I24.1	+,-	protein_cod	92378;749088	upstream;do	RP11-360F5.1,KLHL5	-,+	antisense,p	0
3382	chr2	162138903	+	chr4	49289276	-	N	N	N	N	N	Y	transl_inter	na	NA	ncRNA_exo	AC009299.4	+	pseudogene	0	intergenic	RP11-1281K21.7,RP11-2	+,-	pseudogene	40344;199667
3383	chr2	183914145	+	chr4	91967031	-	N	Y	N	N	N	N	transl_inter	na	NA	intergenic	NCKAP1,SNORA77	-,-	protein_cod	10559;14818	intronic	CCSER1	+	protein_cod	0
3384	chr2	183914211	+	chr4	91967078	-	N	N	N	Y	N	N	transl_inter	na	NA	intergenic	NCKAP1,SNORA77	-,-	protein_cod	10625;14752	intronic	CCSER1	+	protein_cod	0
3385	chr2	42052606	+	chr4	66413931	+	Y	Y	Y	Y	Y	Y	transl_inter	na	NA	intergenic	LDHAP3,AC009413.2	+,-	pseudogene	4712;24737	intronic	EPHA5	-	protein_cod	0
3386	chr2	63935288	+	chr5	118935641	-	Y	N	N	N	N	N	transl_inter	na	NA	intergenic	AC096664.3,AC09666	-,+	miRNA,pseu	12619;8968	intergenic	FABP5P6,snoU13	-,-	pseudogene	44288;10256
3387	chr2	14414570	-	chr6	130218879	+	N	Y	Y	Y	N	N	transl_inter	na	NA	ncRNA_intro	LINC00276	-	lincRNA	0	intergenic;ir	TMEM244,L3MBTL3	-,+	protein_cod	36187;115965
3388	chr2	139140877	-	chr6	91673347	+	N	Y	N	N	N	N	transl_inter	na	NA	intergenic	AC097523.3,AC09772	+,-	pseudogene	75337;28759	intergenic	MAP3K7,RP1-177I10.1	-,-	protein_cod	376583;426684
3389	chr2	26673611	-	chr7	19518322	-	Y	Y	Y	Y	Y	Y	transl_inter	na	NA	intronic	DRC1	+	protein_cod	0	ncRNA_intro	AC007091.1	+	lincRNA	0
3390	chr2	104694503	-	chr7	135542558	-	N	N	N	Y	N	Y	transl_inter	na	NA	downstream	RP11-76I14.1	+	lincRNA	0	intergenic;ir	AC091736.1,AC015987.1	-,+	miRNA,antis	59701;68645
3391	chr2	104694523	-	chr7	135542529	-	N	Y	Y	N	N	N	transl_inter	na	NA	downstream	RP11-76I14.1	+	lincRNA	0	intergenic	AC091736.1,AC015987.1	-,+	miRNA,antis	59672;68674
3392	chr2	132767884	-	chr7	57927960	-	N	N	N	Y	N	N	transl_inter	na	NA	intergenic	RP1-156L9.1,AC09378	-,-	lincRNA,pse	16150;27476	intergenic	RP11-548K12.12,NONE	+,NONE	pseudogene	31208;NONE
3393	chr2	132800199	-	chr7	57932777	-	N	N	N	N	Y	N	transl_inter	na	NA	intergenic	AC093787.1,RP11-629	-,-	pseudogene	4633;37664	intergenic;ir	RP11-548K12.12,NONE	+,NONE	pseudogene	36025;NONE
3394	chr2	132800204	-	chr7	57932733	-	Y	N	N	N	N	N	transl_inter	na	NA	intergenic;ir	AC093787.1,RP11-629	-,-	pseudogene	4638;37659	intergenic	RP11-548K12.12,NONE	+,NONE	pseudogene	35981;NONE
3395	chr2	132800204	-	chr7	57932777	-	N	Y	N	N	N	N	transl_inter	na	NA	intergenic;ir	AC093787.1,RP11-629	-,-	pseudogene	4638;37659	intergenic;ir	RP11-548K12.12,NONE	+,NONE	pseudogene	36025;NONE
3396	chr2	132813253	-	chr7	65054324	-	N	N	Y	N	N	N	transl_inter	na	NA	intergenic	AC093787.1,RP11-629	-,-	pseudogene	17687;24610	intergenic	RP11-667F9.1,RP13-157	-,+	pseudogene	34667;27666
3397	chr2	214171067	-	chr7	28380206	-	Y	Y	Y	Y	Y	N	transl_inter	na	NA	intronic	SPAG16	+	protein_cod	0	intronic	CREB5	+	protein_cod	0
3398	chr2	214171089	-	chr7	28380086	-	N	N	N	N	N	Y	transl_inter	na	NA	intronic	SPAG16	+	protein_cod	0	intronic	CREB5	+	protein_cod	0
3399	chr2	11899899	-	chr7	138786213	+	N	N	N	Y	N	N	transl_inter	na	NA	intronic	LPIN1	+	protein_cod	0	intronic	ZC3HAV1	-	protein_cod	0
3400	chr2	91662944	-	chr7	64981468	+	N	N	N	N	N	Y	transl_inter	na	NA	intergenic	AC018696.1,IGKV1OF	+,+	pseudogene	26795;16143	intergenic	RP11-328P23.3,RP11-66	-,-	pseudogene	7611;9074
3401	chr2	104694503	+	chr7	135542558	+	N	N	Y	N	N	N	transl_inter	na	NA	downstream	RP11-76I14.1	+	lincRNA	0	intergenic;ir	AC091736.1,AC015987.1	-,+	miRNA,antis	59701;68645
3402	chr2	229533447	+	chr7	128077973	+	N	N	Y	N	N	Y	transl_inter	na	NA	intergenic;ir	AC009410.1,AC07401	-,-	lincRNA,linc	57336;14806	intergenic	RP11-155G14.1,RNU7-5	-,-	pseudogene	4206;5530
3403	chr2	229533447	+	chr7	128078020	+	N	Y	N	Y	Y	N	transl_inter	na	NA	intergenic;ir	AC009410.1,AC07401	-,-	lincRNA,linc	57336;14806	intergenic	RP11-155G14.1,RNU7-5	-,-	pseudogene	4253;5483
3404	chr2	32046156	-	chr8	97440448	-	N	Y	N	N	Y	N	transl_inter	na	NA	intergenic	AL121652.2,KRT18P5	-,-	miRNA,pseu	10799;1504	intergenic	KB-1448A5.1,RNU6-1172	-,-	lincRNA,snR	40782;2834
3405	chr2	32046191	-	chr8	97440541	-	Y	N	N	N	N	N	transl_inter	na	NA	intergenic	AL121652.2,KRT18P5	-,-	miRNA,pseu	10834;1469	intergenic	KB-1448A5.1,RNU6-1172	-,-	lincRNA,snR	40875;2741
3406	chr2	32046266	-	chr8	97440665	-	N	N	N	N	N	Y	transl_inter	na	NA	intergenic	AL121652.2,KRT18P5	-,-	miRNA,pseu	10909;1394	intergenic	KB-1448A5.1,RNU6-1172	-,-	lincRNA,snR	40999;2617
3407	chr2	221057276	-	chr8	141960213	-	N	N	N	N	N	Y	transl_inter	na	NA	ncRNA_intro	AC114765.2,AC11476	+,+	lincRNA,linc	0	intronic	PTK2	-	protein_cod	0
3408	chr2	95418904	-	chr8	38382379	+	N	N	Y	Y	N	N	transl_inter	na	NA	intergenic	AC073464.6,CYP4F32	+,-	pseudogene	1866;6100	intronic	C8orf86	-	protein_cod	0
3409	chr2	130968479	-	chr9	114404927	-	Y	Y	Y	Y	Y	N	transl_inter	na	NA	ncRNA_intro	AC018804.6	+	pseudogene	0	intronic	DNAJC25,DNAJC25-GN	+,+	protein_cod	0
3410	chr2	131777980	-	chr9	1407543	-	N	N	N	Y	N	N	transl_inter	na	NA	intronic	ARHGEF4	+	protein_cod	0	intergenic	RNA5SP279,RP11-443B	+,-	rRNA,pseuc	73590;570241
3411	chr2	38505902	+	chr9	68418844	-	Y	Y	Y	Y	Y	Y	transl_inter	na	NA	intergenic	AC009229.5,GAPDHP	-,+	lincRNA,pse	39170;6650	intergenic;ir	MIR4477A,RP11-764K9.4	+,-	miRNA,pseu	3456;9017
3412	chr2	5628691	+	chr9	117653033	+	Y	Y	N	N	Y	N	transl_inter	na	NA	intergenic;ir	AC073143.1,AC10705	-,-	pseudogene	174424;61221	intergenic	RP11-78H18.2,TNFSF8	+,-	pseudogene	42168;12091
3413	chr2	35879354	+	chr9	98459861	+	Y	Y	Y	Y	N	Y	transl_inter	na	NA	intergenic	AC083939.1,MRPL50F	+,-	pseudogene	182447;70471	ncRNA_intro	RP11-180I4.2	+	lincRNA	0
3414	chr2	133110564	+	chr9	68377382	+	Y	Y	N	N	N	N	transl_inter	na	NA	ncRNA_exo	FAM201B	+	pseudogene	0	intergenic	PTGER4P3,RP11-764K9	-,-	pseudogene	5505;20496
3415	chr2	204056053	+	chr9	132191818	+	Y	N	N	N	Y	N	transl_inter	na	NA	intronic	NBEAL1	+	protein_cod	0	intergenic	RP11-65J3.6,RP11-65J3	-,-	pseudogene	49069;1113
3416	chr2	234085782	+	chr9	135394971	+	N	N	N	N	N	N	transl_inter	na	NA	intronic	INPP5D	+	protein_cod	0	intronic	C9orf171	+	protein_cod	0
3417	chr2	97851222	-	chrX	101835466	-	Y	Y	Y	Y	Y	Y	transl_inter	na	NA	exonic	ANKRD36	+	protein_cod	0	intergenic	NXF4,ARMCX5	+,+	pseudogene	8843;18630
3418	chr2	47319280	-	chrX	153684967	+	N	Y	Y	N	Y	Y	transl_inter	na	NA	intronic;intro	C2orf61,RP11-761B3.1	-,-	protein_cod	0	intergenic;ir	FAM50A,PLXNA3	+,+	protein_cod	5965;1654
3419	chr2	4497046	+	chrX	130270612	-	N	N	N	N	N	Y	transl_inter	na	NA	intergenic	AC068292.1,NPM1P4	-,-	miRNA,pseu	220938;64748	intergenic	RN7SL191P,OR11Q1P	-,+	misc_RNA,p	2248;67490
3420	chr2	4497701	+	chrX	130270570	-	N	N	Y	N	N	N	transl_inter	na	NA	intergenic	AC068292.1,NPM1P4	-,-	miRNA,pseu	221593;64093	intergenic	RN7SL191P,OR11Q1P	-,+	misc_RNA,p	2206;67532
3421	chr2	28353914	+	chrX	116838846	-	N	N	Y	N	N	N	transl_inter	na	NA	intronic	BRE	+	protein_cod	0	intergenic	RP3-378P9.2,KLHL13	+,-	pseudogene	304802;192930
3422	chr2	47319280	+	chrX	153684967	-	N	N	Y	N	N	N	transl_inter	na	NA	intronic;intro	C2orf61,RP11-761B3.1	-,-	protein_cod	0	intergenic;ir	FAM50A,PLXNA3	+,+	protein_cod	5965;1654
3423	chr2	133011728	-	chrY	10036904	+	N	N	N	N	Y	N	transl_inter	na	NA	upstream	RNA5-8SP5	-	rRNA	0	upstream	AC010970.2,RNA5-8SP6	-,+	pseudogene	0
3424	chr2	133015071	-	chrY	10033585	+	N	Y	N	N	N	N	transl_inter	na	NA	upstream	MIR663B	-	miRNA	0	upstream	AC010970.1	+	miRNA	0
3425	chr2	133015086	-	chrY	10033570	+	N	N	N	N	Y	N	transl_inter	na	NA	upstream	MIR663B	-	miRNA	0	upstream	AC010970.1	+	miRNA	0
3426	chr2	133028833	-	chrY	10020713	+	N	N	N	Y	N	N	transl_inter	na	NA	intergenic	CDC27P1,AC097532.2	+,-	pseudogene	8218;14535	intergenic	PCMTD1P1,CDC27P2	+,-	pseudogene	8897;7273
3427	chr2	133011813	+	chrY	10036648	-	N	N	Y	N	Y	N	transl_inter	na	NA	upstream	RNA5-8SP5	-	rRNA	0	ncRNA_exo	AC010970.2	-	pseudogene	0
3428	chr20	3349405	-	chr3	195194095	-	N	N	N	N	Y	N	transl_inter	na	NA	intronic	C20orf194	-	protein_cod	0	intergenic	ACAP2,PPP1R2	-,-	protein_cod	30288;47126
3429	chr20	54859326	-	chr3	185172692	-	N	Y	N	Y	N	N	transl_inter	na	NA	intergenic;ir	RP11-380D15.2,RP11-	+,-	pseudogene	15039;11697	intronic	MAP3K13	+	protein_cod	0
3430	chr20	54859326	+	chr3	138541391	+	Y	N	N	N	N	N	transl_inter	na	NA	intergenic;ir	RP11-380D15.2,RP11-	+,-	pseudogene	15039;11697	intronic	PIK3CB	-	protein_cod	0
3431	chr20	29653331	-	chr4	190201589	+	Y	N	N	Y	N	N	transl_inter	na	NA	intergenic;ir	MLLT10P1,DEFB115	-,+	pseudogene	15271;192136	intergenic	RP11-706F1.1,RP11-706	+,-	lincRNA,linc	75558;8242
3432	chr20	29653342	-	chr4	190201650	+	N	N	N	N	Y	N	transl_inter	na	NA	intergenic	MLLT10P1,DEFB115	-,+	pseudogene	15282;192125	intergenic	RP11-706F1.1,RP11-706	+,-	lincRNA,linc	75619;8181
3433	chr20	26138580	+	chr4	33846330	-	N	N	Y	N	N	N	transl_inter	na	NA	intergenic;ir	RP13-329D4.3,MIR66	-,-	lincRNA,pro	23746;28976	ncRNA_intro	RP11-79E3.3	-	lincRNA	0
3434	chr20	29653331	+	chr4	190201659	-	N	N	N	N	Y	N	transl_inter	na	NA	intergenic;ir	MLLT10P1,DEFB115	-,+	pseudogene	15271;192136	intergenic	RP11-706F1.1,RP11-706	+,-	lincRNA,linc	75628;8172
3435	chr20	29598898	+	chr4	190840803	+	Y	N	N	N	N	N	transl_inter	na	NA	intergenic	RP4-610C12.1,FRG1B	-,+	antisense,p	2492;12959	ncRNA_intro	AF146191.4	-	lincRNA	0
3436	chr20	30014051	-	chr5	126065136	-	N	Y	N	N	N	N	transl_inter	na	NA	ncRNA_intro	DEFB122	-	pseudogene	0	intergenic	RP11-772E11.1,HSPE1P	-,-	pseudogene	65876;7994
3437	chr20	47596876	+	chr5	10005642	-	N	N	N	N	Y	N	transl_inter	na	NA	intronic	ARFGEF2	+	protein_cod	0	intergenic	CTD-2143L24.1,CTD-21	-,+	lincRNA,linc	101704;131608
3438	chr20	4427307	+	chr5	24449296	+	Y	Y	Y	N	Y	Y	transl_inter	na	NA	intergenic	RP11-307O10.1,RP5-1	-,+	lincRNA,linc	14987;28978	intergenic	CTD-2074D8.1,CDH10	-,-	lincRNA,pro	95744;37913

3439	chr20	13915466	+	chr5	132961595	+	Y	N	N	N	N	N	transl_inter	na	NA	intronic	SEL1L2	-	protein_cod	0	ncRNA_exo	AC005195.1	-	miRNA	0
3440	chr20	22863396	+	chr5	9024649	+	N	N	Y	Y	Y	Y	transl_inter	na	NA	intergenic	KRT18P3,CYB5P4	-,-	pseudogene	148669;2912	ncRNA_intr	CTD-2215L10.1	+	antisense	0
3441	chr20	25840708	+	chr5	154275111	+	Y	N	Y	Y	Y	N	transl_inter	na	NA	intronic	FAM182B	-	protein_cod	0	intronic;intr	GEMIN5	-	protein_cod	0
3442	chr20	25844028	+	chr5	154275111	+	N	Y	N	N	N	Y	transl_inter	na	NA	intronic	FAM182B	-	protein_cod	0	intronic;intr	GEMIN5	-	protein_cod	0
3443	chr20	25888637	-	chr6	154216091	-	Y	Y	Y	Y	Y	Y	transl_inter	na	NA	intergenic	FAM182B,CFTRP1	-,-	protein_cod	39851;11498	intergenic	RNU6-896P,HMGB3P19	+,-	snRNA,pse	213403;43477
3444	chr20	33248517	-	chr6	67782166	+	Y	Y	N	Y	Y	N	transl_inter	na	NA	intronic	PIGU	-	protein_cod	0	intergenic	RNU7-66P,AL590874.1	-,-	snRNA,miR	343369;77236
3445	chr20	54859608	-	chr6	94728887	+	Y	N	N	N	N	N	transl_inter	na	NA	intergenic	RP11-380D15.2,RP11-	+,-	pseudogene	15321;11415	intergenic	RP11-524K14.1,RP3-463	+,+	lincRNA,linc	129759;144751
3446	chr20	18670567	-	chr7	5809597	-	Y	N	Y	N	N	N	transl_inter	na	NA	ncRNA_intr	RP11-379J5.5	-	antisense	0	intronic;intr	RNF216	-	protein_cod	0
3447	chr20	22064187	-	chr7	153203509	-	Y	N	N	N	N	N	transl_inter	na	NA	intergenic	RP11-125P18.1,RP5-1	+,+	lincRNA,linc	8949;137005	intergenic	AC091652.2,DPP6	+,+	pseudogene	56549;380673
3448	chr20	25831927	-	chr7	57703955	-	N	N	N	N	Y	N	transl_inter	na	NA	intronic	FAM182B	-	protein_cod	0	ncRNA_exo	RP11-368M16.3	-	pseudogene	0
3449	chr20	25864860	-	chr7	57731047	-	N	N	N	Y	N	N	transl_inter	na	NA	intergenic;ir	FAM182B,CFTRP1	-,-	protein_cod	16074;35275	intergenic	RP11-368M16.7,RP11-54	+,-	pseudogene	15949;99278
3450	chr20	5632309	-	chr7	46566840	+	Y	N	N	Y	N	N	transl_inter	na	NA	intergenic	GPCPD1,C20orf196	-,+	protein_cod	40637;98730	intergenic	AC004869.2,HMGN1P19	-,+	lincRNA,pse	45664;107372
3451	chr20	26146311	-	chr7	57608296	+	Y	N	Y	N	N	Y	transl_inter	na	NA	intergenic	RP13-329D4.3,MIR663	-,-	lincRNA,pro	31477;21245	intergenic	RP11-1324A7.2,NCOR1P	+,+	pseudogene	62695;51204
3452	chr20	26244567	-	chr7	57732562	+	Y	N	N	N	N	N	transl_inter	na	NA	intergenic	MIR663A,NONE	-,NONE	processed_	12405;NONE	intergenic	RP11-368M16.7,RP11-54	+,-	pseudogene	17464;97763
3453	chr20	25856289	+	chr7	57872102	-	N	Y	N	Y	N	N	transl_inter	na	NA	intergenic	FAM182B,CFTRP1	-,-	protein_cod	7503;43846	intergenic	RP11-548K12.4,RP11-54	-,-	pseudogene	34875;5205
3454	chr20	18670567	+	chr7	5809597	+	N	Y	N	Y	Y	N	transl_inter	na	NA	ncRNA_intr	RP11-379J5.5	-	antisense	0	intronic;intr	RNF216	-	protein_cod	0
3455	chr20	25864859	+	chr7	57730098	+	Y	N	N	N	N	N	transl_inter	na	NA	intergenic;ir	FAM182B,CFTRP1	-,-	protein_cod	16073;35276	intergenic	RP11-368M16.7,RP11-54	+,-	pseudogene	15000;100227
3456	chr20	25864859	+	chr7	57730641	+	N	N	N	N	Y	Y	transl_inter	na	NA	intergenic;ir	FAM182B,CFTRP1	-,-	protein_cod	16073;35276	intergenic	RP11-368M16.7,RP11-54	+,-	pseudogene	15543;99684
3457	chr20	25864860	+	chr7	57731102	+	N	N	Y	Y	N	N	transl_inter	na	NA	intergenic;ir	FAM182B,CFTRP1	-,-	protein_cod	16074;35275	intergenic	RP11-368M16.7,RP11-54	+,-	pseudogene	16004;99223
3458	chr20	48875003	-	chr8	144018695	-	N	Y	Y	N	N	N	transl_inter	na	NA	intergenic	CEBPB,RP11-290F20.	+,+	protein_cod	65791;9020	intergenic	CYP11B2,RP11-273G15.	-,-	protein_cod	19436;44460
3459	chr20	48875004	-	chr8	144018724	-	Y	N	N	N	N	N	transl_inter	na	NA	intergenic	CEBPB,RP11-290F20.	+,+	protein_cod	65792;9019	intergenic	CYP11B2,RP11-273G15.	-,-	protein_cod	19465;44431
3460	chr20	53466092	-	chr8	42006955	-	N	N	N	N	Y	N	transl_inter	na	NA	intergenic	DOK5,RNU4ATAC7P	+,-	protein_cod	198382;10135	intergenic	RP11-589C21.6,RP11-58	-,-	lincRNA,ant	9685;2335
3461	chr20	60370116	-	chr8	142724303	-	N	N	N	N	Y	N	transl_inter	na	NA	intronic	CDH4	+	protein_cod	0	intergenic	AC138647.1,AC104417.1	+,+	protein_cod	195466;302557
3462	chr20	23248605	-	chr8	103954656	+	N	N	N	N	N	Y	transl_inter	na	NA	intergenic	RP4-737E23.2,RP3-32	+,-	lincRNA,ant	77661;78973	intergenic	RPL5P24,Y_RNA	-,-	pseudogene	18173;8039
3463	chr20	20720371	-	chr9	19671058	+	N	N	N	Y	Y	N	transl_inter	na	NA	upstream	RN7SL607P	-	misc_RNA	0	intronic	SLC24A2	-	protein_cod	0
3464	chr20	54384170	-	chrX	154387895	-	N	N	N	Y	N	N	transl_inter	na	NA	intergenic	RP5-1010E17.1,CBLN	+,-	lincRNA,pro	299418;188326	intergenic	RP11-143H17.1,VBP1	+,+	pseudogene	3117;37389
3465	chr20	11281584	-	chrX	81651235	+	N	Y	Y	N	N	N	transl_inter	na	NA	ncRNA_intr	RP4-734C18.1	-	lincRNA	0	intergenic;ir	RNU6-974P,RP4-581F12	-,-	snRNA,pse	394459;101883
3466	chr20	18562243	-	chrX	151692595	+	Y	N	N	N	N	N	transl_inter	na	NA	intergenic	LINC00493,DTD1	+,+	lincRNA,pro	12036;6294	intergenic	KRT8P8,GABRQ	+,+	pseudogene	43099;114042
3467	chr20	11281584	+	chrX	81651235	-	N	Y	Y	N	N	N	transl_inter	na	NA	ncRNA_intr	RP4-734C18.1	-	lincRNA	0	intergenic;ir	RNU6-974P,RP4-581F12	-,-	snRNA,pse	394459;101883
3468	chr20	18689348	+	chrX	86280019	+	N	N	N	N	Y	N	transl_inter	na	NA	intronic	DTD1	+	protein_cod	0	intergenic	RP3-431C21.1,RP11-192	+,-	pseudogene	136200;118838
3469	chr20	18689482	+	chrX	86280080	+	Y	N	N	N	N	N	transl_inter	na	NA	intronic	DTD1	+	protein_cod	0	intergenic	RP3-431C21.1,RP11-192	+,-	pseudogene	136261;118777
3470	chr20	26188767	-	chrY	10034159	+	N	N	Y	Y	N	N	transl_inter	na	NA	ncRNA_intr	MIR663A	-	processed_	0	downstream	AC010970.1	+	miRNA	0
3471	chr21	11022748	-	chr22	45991517	+	Y	N	N	Y	N	N	transl_inter	na	NA	ncRNA_intr	BAGE2	-	pseudogene	0	intronic;intr	FBLN1	+	protein_cod	0
3472	chr21	11022748	+	chr22	45991517	-	Y	N	N	Y	N	N	transl_inter	na	NA	ncRNA_intr	BAGE2	-	pseudogene	0	intronic;intr	FBLN1	+	protein_cod	0
3473	chr21	30382776	-	chr3	119431791	-	N	N	N	Y	N	N	transl_inter	na	NA	intronic	RWDD2B	-	protein_cod	0	intronic	MAATS1	+	protein_cod	0
3474	chr21	11152404	+	chr4	183542724	+	N	N	N	Y	N	N	transl_inter	na	NA	intergenic	BAGE2,VN1R7P	-,-	pseudogene	53424;1433	intronic	TENM3	+	protein_cod	0
3475	chr21	30050011	-	chr5	123549613	-	Y	N	N	N	N	N	transl_inter	na	NA	intergenic	AF131217.1,RNU6-872	-,+	lincRNA,snR	2841;65519	intergenic	KRT18P16,HMGB1P29	+,-	pseudogene	576516;6659
3476	chr21	19093229	-	chr5	167286073	+	N	N	N	N	N	Y	transl_inter	na	NA	intergenic	AP000432.2,C21orf91-	-,-	lincRNA,linc	60527;42403	intronic	TENM2	+	protein_cod	0
3477	chr21	11067249	-	chr7	151979931	-	Y	Y	Y	N	N	Y	transl_inter	na	NA	ncRNA_intr	BAGE2	-	pseudogene	0	intronic	KMT2C	-	protein_cod	0
3478	chr21	11067283	-	chr7	151979897	-	N	N	N	Y	N	N	transl_inter	na	NA	ncRNA_intr	BAGE2	-	pseudogene	0	intronic;intr	KMT2C	-	protein_cod	0
3479	chr21	23546941	+	chr7	75642667	-	N	N	N	N	N	Y	transl_inter	na	NA	intergenic	AP000705.6,RNU4-45I	-,+	pseudogene	20322;30570	intronic	STYXL1	-	protein_cod	0
3480	chr21	11067283	+	chr7	151979897	+	Y	Y	Y	Y	N	Y	transl_inter	na	NA	ncRNA_intr	BAGE2	-	pseudogene	0	intronic;intr	KMT2C	-	protein_cod	0
3481	chr21	11097503	+	chr7	152099150	+	Y	Y	N	N	N	N	transl_inter	na	NA	ncRNA_intr	BAGE2	-	pseudogene	0	intronic	KMT2C	-	protein_cod	0
3482	chr21	25300215	+	chr7	71318612	+	N	N	N	N	N	Y	transl_inter	na	NA	intergenic	AP000474.1,AP000477	-,-	lincRNA,linc	37358;33005	intronic	CALN1	-	protein_cod	0
3483	chr21	24191239	-	chr8	141500799	-	N	N	Y	N	N	N	transl_inter	na	NA	intergenic	RN7SL609P,AP000949	+,+	misc_RNA,p	137953;63664	intergenic	TRAPPC9,CTA-204B4.2	-,-	protein_cod	32121;15113
3484	chr21	24191502	-	chr8	141500934	-	N	Y	N	N	N	N	transl_inter	na	NA	intergenic	RN7SL609P,AP000949	+,+	misc_RNA,p	138216;63401	intergenic	TRAPPC9,CTA-204B4.2	-,-	protein_cod	32256;14978
3485	chr21	24191877	-	chr8	141500916	-	N	N	N	N	Y	N	transl_inter	na	NA	intergenic	RN7SL609P,AP000949	+,+	misc_RNA,p	138591;63026	intergenic	TRAPPC9,CTA-204B4.2	-,-	protein_cod	32238;14996
3486	chr21	43250843	-	chr8	20479605	-	N	Y	Y	N	Y	Y	transl_inter	na	NA	intronic	PRDM15	-	protein_cod	0	intergenic	U3,RP11-108E14.1	-,-	snoRNA,linc	7168;33597
3487	chr21	18553568	-	chr8	64238092	+	N	N	N	N	N	Y	transl_inter	na	NA	intergenic	AF212831.2,NEK4P1	-,-	lincRNA,pse	307557;29219	intergenic	RN7SL135P,CTD-3046C4	-,-	misc_RNA,l	90623;59305
3488	chr21	18553575	-	chr8	64238060	+	N	N	Y	Y	N	N	transl_inter	na	NA	intergenic;ir	AF212831.2,NEK4P1	-,-	lincRNA,pse	307564;29212	intergenic;ir	RN7SL135P,CTD-3046C4	-,-	misc_RNA,l	90591;59337
3489	chr21	42458069	+	chr8	107246401	+	N	Y	Y	N	N	N	transl_inter	na	NA	intergenic	YRDCP3,LINC00323	+,-	pseudogene	221670;55358	intergenic	SLC16A14P1,RP11-778D	+,-	pseudogene	16955;31262
3490	chr21	10804586	-	chrY	28813711	-	N	N	N	Y	N	N	transl_inter	na	NA	intergenic	bP-21201H5.1,IGHV1C	-,+	pseudogene	328525;58036	intergenic	PARP4P1,NONE	-,NONE	pseudogene	32912;NONE
3491	chr21	11054315	-	chrY	59001011	+	Y	Y	Y	Y	Y	Y	transl_inter	na	NA	ncRNA_intr	BAGE2	-	pseudogene	0	upstream	CTBP2P1	+	pseudogene	0
3492	chr22	17500831	+	chr4	183542903	+	N	N	N	N	N	Y	transl_inter	na	NA	intergenic	AC006548.26,CECR7	+,+	pseudogene	7009;16629	intronic	TENM3	+	protein_cod	0
3493	chr22	31751806	-	chr6	131678295	+	N	Y	Y	N	Y	N	transl_inter	na	NA	intergenic	AC005003.1,RNU6-33	+,-	protein_cod	7136;32913	intergenic	AKAP7,RPL21P67	+,+	protein_cod	73620;111904
3494	chr22	20324158	+	chr6	90002554	-	N	N	N	N	N	Y	transl_inter	na	NA	intergenic	XXbac-B444P24.14,XX	+,+	lincRNA,ant	15432;2170	intronic	GABRR2	-	protein_cod	0
3495	chr22	20324278	+	chr6	90002539	-	N	N	N	N	Y	N	transl_inter	na	NA	intergenic	XXbac-B444P24.14,XX	+,+	lincRNA,ant	15552;2050	intronic	GABRR2	-	protein_cod	0
3496	chr22	39608025	-	chr7	137485122	-	N	Y	N	N	N	N	transl_inter	na	NA	intergenic	FUNDC2P4,PDGFB	+,-	pseudogene	56075;11339	intronic	DGKI	-	protein_cod	0
3497	chr22	39608426	-	chr7	137485141	-	N	N	Y	N	N	N	transl_inter	na	NA	intergenic	FUNDC2P4,PDGFB	+,-	pseudogene	56476;10938	intronic	DGKI	-	protein_cod	0
3498	chr22	24077834	-	chr7	83929121	+	N	N	N	N	Y	Y	transl_inter	na	NA	intergenic	GUSBP11,LL22NC03-I	-,-	processed_	18291;3031	intronic	SEMA3A	-	protein_cod	0
3499	chr22	17142902	+	chr7	61827490	+	N	Y	N	N	N	N	transl_inter	na	NA	ncRNA_intr	TPTEP1,ANKRD62P1-	+,-	lincRNA,pro	0	intergenic	RP11-715L17.1,RNU6-41	+,-	pseudogene	5304;644351
3500	chr22	23709378	-	chr9	132204424	-	Y	Y	Y	N	N	Y	transl_inter	na	NA	ncRNA_intr	CES5AP1	-	pseudogene	0	ncRNA_intr	RP11-65J3.3	-	lincRNA	0
3501	chr22	23852560	-	chr9	132186744	+	Y	Y	Y	Y	Y	N	transl_inter	na	NA	intergenic;ir	AP000345.1,AP000345	-,-	lincRNA,linc	23393;2121	intergenic;ir	RP11-65J3.6,RP11-65J3.	-,-	pseudogene	43995;6187
3502	chr22	17392951	+	chr9	70152165	+	N	N	N	N	N	Y	transl_inter	na	NA	intergenic	IGKV1OR22-5,IGKV2C	-,-	IG_V_pseu	7365;2174	intergenic	IGKV1OR9-1,FOXD4L5	-,-	IG_V_pseu	7733;23542
3503	chr22	32575906	+	chr9	101475185	+	Y	Y	Y	Y	Y	Y	transl_inter	na	NA	intergenic	RP1-90G24.8,RFPL2	-,-	lincRNA,pro	19527;10516	intergenic	GABBR2,ANKS6	-,-	protein_cod	3706;18426
3504	chr3	46115105	-	chr4	152301652	-	N	N	Y	N	N	N	transl_inter	na	NA	intergenic	XCR1,FLT1P1	-,-	protein_cod	45871;68745	intergenic	RP11-731D1.4,RP11-610	-,-	lincRNA,linc	27276;27051
3505	chr3	68009565	+	chr4	188725831	+	N	Y	N	N	N	N	transl_inter	na	NA	intergenic	RP11-81N13.1,FAM19A	+,+	lincRNA,pro	11428;43794	intergenic	ADAM20P3,RP11-713C1	-,+	pseudogene	56478;165596
3506	chr3	89590548	+	chr4	158442569	+	Y	N	N	N	N	N	transl_inter	na	NA	intergenic	EPHA3,RP11-91A15.2	+,-	protein_cod	59264;46488	intergenic	GRIA2,RP11-364P22.1	+,+	protein_cod	155342;51073
3507	chr3	89590824	+	chr4	158442629	+	N	N	N	N	N	Y	transl_inter	na	NA	intergenic	EPHA3,RP11-91A15.2	+,-	protein_cod	59540;46212	intergenic	GRIA2,RP11-364P22.1	+,+	protein_cod	155402;51013
3508	chr3	117895731	+	chr4	188614301	+	Y	N	N	N	N	N	transl_inter	na	NA	ncRNA_intr	RP11-384F7.1	-	lincRNA	0	intergenic	RP11-565A3.2,ADAM20P	-,-	lincRNA,pse	20506;52947
3509	chr3	117895756	+	chr4	188614276	+	N	N	N	Y	N	N	transl_inter	na	NA	ncRNA_intr	RP11-384F7.1	-	lincRNA	0	intergenic;ir	RP11-565A3.2,ADAM20P	-,-	lincRNA,pse	20481;52972
3510	chr3	105828861	-	chr5	141379670	-	N	Y	Y	Y	Y	Y	transl_inter	na	NA	intergenic	CBLB,RP11-93B21.1	-,-	protein_cod	240465;215723	intronic	GNPDA1	-	protein_cod	0
3511	chr3	172749004	-	chr5	160435680	-	Y	Y	Y	Y	Y	Y	transl_inter	na	NA	intronic	SPATA16	-	protein_cod	0	intergenic	RP11-109J4.1,AC022120	-,-	lincRNA,miR	70047;221377
3512	chr3	32084038	-	chr5	51826922	+	N	Y	Y	Y	Y	Y	transl_inter	na	NA	intergenic	SNORA25,Y_RNA	+,+	snoRNA,mis	4893;3327	intergenic;ir	RPS17P11,CTD-2141G3	+,-	pseudogene	248229;48467
3513	chr3	151148544	-	chr5	39787751	+	Y	Y	Y	Y	Y	Y	transl_inter	na	NA	intronic	MED12L,IGSF10	+,-	protein_cod	0	intergenic	INTS6P1,GCSHP1	-,-	pseudogene	66136;100927
3514	chr3	166440135	-	chr5	51221445	+	N	N	Y	N	N	N	transl_inter	na	NA	downstream	RP11-450H5.3	+	pseudogene	0	intergenic;ir	RNA5SP182,KATNBL1P4	+,+	rRNA,pseu	38842;5197
3515	chr3	166440187	-	chr5	51221445	+	Y	Y	N	Y	Y	Y	transl_inter	na	NA	downstream	RP11-450H5.3	+	pseudogene	0	intergenic;ir	RNA5SP182,KATNBL1P4	+,+	rRNA,pseu	38842;5197
3516	chr3	195670748	-	chr5	281912	+	N	Y	N	N	N	N	transl_inter	na	NA	intergenic	AC024937.6,RP11-480	+,-	pseudogene	5138;5311	intronic	PDCD6	+	protein_cod	0
3517	chr3	46877231	-	chr6	27163025	-	N	N	Y	N	N	N	transl_inter	na	NA	intergenic	PRSS42,MYL3	-,-	protein_cod	1646;22131	intergenic	MIR3143,RP11-209A2.1	+,+	miRNA,pse	47558;15998
3518	chr3	7977464	-	chr6	18358570	+	N	N	N	N	Y	N	transl_inter	na	NA	intergenic	GRM7,LMCD1-AS1	+,-	protein_cod	194249;17028	intergenic;ir	RNU6-263P,RP11-528A1	-,-	snRNA,pse	51260;5078
3519	chr3	26470316	-	chr6	77722512	+	N	Y	Y	Y	Y	Y	transl_inter	na	NA	intergenic;ir	VENTXP4,AC099754.1	-,-	pseudogene	81187;190505	intergenic;ir	RP11-354K4.2,HTR1B	+,-	lincRNA,pro	236935;449436
3520	chr3	7977434	+	chr6	35301331	+	N	N	N	Y	N	Y	transl_inter	na	NA	intergenic	GRM7,LMCD1-AS1	+,-	protein_cod	194219;17058	intergenic	DEF6,PPARD	+,+	protein_cod	11783;9004
3521	chr3	48255017	+	chr6	153429837	+	N	Y	N	N	N	Y	transl_inter	na	NA	intergenic	MIR4443,CAMP	+,+	miRNA,prot	16911;9820	intronic	RGS17	-	protein_cod	0
3522	chr3	83756611	-	chr7	17157113	-	N	N	N	N	N	Y	transl_inter	na	NA	intergenic	CYP51A1P1,SRRM1P	-,+	pseudogene	899439;229585	intergenic	AC098592.8,AC003075.4	-,-	pseudogene	84011;162345

3523	chr3	83756625	-	chr7	17157068	-	Y	N	N	Y	N	N	transl_inter	na	NA	intergenic	CYP51A1P1,SRRM1P	-,+	pseudogene	899453;229571	intergenic	AC098592.8,AC003075.4	-,-	pseudogene	83966;162390
3524	chr3	83756711	-	chr7	17157013	-	N	Y	N	N	N	N	transl_inter	na	NA	intergenic	CYP51A1P1,SRRM1P	-,+	pseudogene	899539;229485	intergenic	AC098592.8,AC003075.4	-,-	pseudogene	83911;162445
3525	chr3	169203729	-	chr7	9055327	-	N	N	N	N	N	Y	transl_inter	na	NA	intronic	MECOM	-	protein_cod	0	intergenic	AC009500.2,RP4-668E1(	+,+	pseudogene	76328;68325
3526	chr3	104163408	+	chr7	24437016	-	N	N	N	N	Y	N	transl_inter	na	NA	intergenic	RP11-40M23.1,RP11-2	+,+	pseudogene	380982;58136	intergenic	NPY,RNU6-1103P	+,+	protein_cod	105532;139935
3527	chr3	104163474	+	chr7	24436814	-	N	N	N	N	N	Y	transl_inter	na	NA	intergenic	RP11-40M23.1,RP11-2	+,+	pseudogene	381048;58070	intergenic	NPY,RNU6-1103P	+,+	protein_cod	105330;140137
3528	chr3	2747421	+	chr7	113682926	+	N	N	Y	N	N	N	transl_inter	na	NA	intronic	CNTN4	+	protein_cod	0	intronic	PPP1R3A	-	protein_cod	0
3529	chr3	80590176	+	chr7	96475902	+	N	Y	Y	Y	Y	N	transl_inter	na	NA	intergenic;ir	RP11-481N16.1,RP11-	+,-	pseudogene	98721;220017	intergenic	SHFM1,MARK2P10	-,+	protein_cod	136699;11592
3530	chr3	119478564	+	chr7	146035843	+	N	N	N	N	N	Y	transl_inter	na	NA	intronic	MAATS1	+	protein_cod	0	intronic;intrc	CNTNAP2	+	protein_cod	0
3531	chr3	165132343	-	chr8	25463341	-	N	N	N	N	N	Y	transl_inter	na	NA	ncRNA_intr	RP11-85M11.2	+	lincRNA	0	intergenic	CDCA2,RP11-219J21.1	+,+	protein_cod	97905;79973
3532	chr3	111274086	-	chr8	128533830	+	Y	Y	Y	Y	Y	Y	transl_inter	na	NA	intronic	CD96	+	protein_cod	0	intergenic	CASC8,CASC11	-,-	antisense,lir	39446;164758
3533	chr3	10266852	+	chr8	33898508	+	N	Y	N	N	N	N	transl_inter	na	NA	intronic	IRAK2	+	protein_cod	0	intergenic	RP11-317N12.1,RP11-43	+,+	lincRNA,linc	1982;133896
3534	chr3	137221705	-	chr9	2397101	-	Y	Y	Y	N	Y	Y	transl_inter	na	NA	intergenic	IL20RB,RNA5SP142	+,-	protein_cod	491778;15271	intergenic	RN7SL592P,RP11-125B2	-,-	misc_RNA,a	167860;25601
3535	chr3	137221811	-	chr9	2397149	-	N	N	N	Y	N	N	transl_inter	na	NA	intergenic	IL20RB,RNA5SP142	+,-	protein_cod	491884;15165	intergenic	RN7SL592P,RP11-125B2	-,-	misc_RNA,a	167908;25553
3536	chr3	144734889	-	chr9	98993629	+	N	N	N	Y	N	N	transl_inter	na	NA	intergenic	RNA5SP144,RP11-622	-,+	rRNA,pseuc	829027;506436	intergenic	EIF4BP3,HSD17B3	+,-	pseudogene	83491;3959
3537	chr3	73160814	+	chr9	99002312	-	N	Y	N	N	N	N	transl_inter	na	NA	downstream	RNU2-64P	+	snRNA	0	intronic	HSD17B3	-	protein_cod	0
3538	chr3	35510723	+	chr9	103742576	+	N	N	N	N	Y	N	transl_inter	na	NA	intergenic	RNU6-243P,ARPP21	-,+	snRNA,prot	212285;169714	intergenic	GAPDHP26,LPPR1	+,+	pseudogene	3612;48415
3539	chr3	80590176	+	chr9	12556850	+	Y	N	N	N	N	Y	transl_inter	na	NA	intergenic;ir	RP11-481N16.1,RP11-	+,-	pseudogene	98721;220017	intergenic	RNU2-47P,TYRP1	-,+	snRNA,prot	256399;128589
3540	chr3	83756629	-	chrX	45672668	-	N	Y	N	N	N	N	transl_inter	na	NA	intergenic	CYP51A1P1,SRRM1P	-,+	pseudogene	899457;229567	intergenic	RP6-99M1.2,RP5-1158E	-,-	lincRNA,linc	42991;34660
3541	chr3	178286522	-	chrX	3233474	-	N	Y	N	N	N	N	transl_inter	na	NA	ncRNA_intr	RP11-385J1.2	-	antisense	0	intronic	MXRA5	-	protein_cod	0
3542	chr3	150537184	-	chrX	26922363	+	N	Y	N	N	N	N	transl_inter	na	NA	intergenic	SIAH2,CLRN1-AS1	-,+	protein_cod	55966;33087	intergenic	RNU1-142P,RP11-268G1	+,-	snRNA,lincF	113960;138661
3543	chr3	66335898	+	chrX	97208008	-	Y	Y	N	Y	Y	Y	transl_inter	na	NA	intronic	SLC25A26	+	protein_cod	0	intergenic	RP13-75G22.1,RP13-13(	+,-	pseudogene	44920;22868
3544	chr3	174514510	+	chrX	97221559	+	Y	N	N	N	Y	N	transl_inter	na	NA	intronic	NAALADL2	+	protein_cod	0	intergenic	RP13-75G22.1,RP13-13(	+,-	pseudogene	58471;9317
3545	chr3	16409885	+	chrY	7588542	+	Y	N	Y	N	Y	Y	transl_inter	na	NA	intronic;intrc	RFTN1	-	protein_cod	0	ncRNA_intr	RFTN1P1	-	pseudogene	0
3546	chr4	177134027	+	chr5	51826922	+	Y	N	N	N	N	N	transl_inter	na	NA	downstream	ASB5	-	protein_cod	0	intergenic;ir	RPS17P11,CTD-2141G3	+,-	pseudogene	248229;48467
3547	chr4	75641494	-	chr6	98652131	-	Y	Y	Y	Y	Y	Y	transl_inter	na	NA	intergenic	AC142293.3,BTC	-,-	antisense,pi	126830;28475	ncRNA_intr	RP11-436D23.1	+	lincRNA	0
3548	chr4	157768894	-	chr6	155707848	+	N	N	N	Y	N	N	transl_inter	na	NA	intronic	PDGFC	-	protein_cod	0	intergenic	RP1-257I9.2,NOX3	-,-	lincRNA,pro	5531;8656
3549	chr4	179198312	-	chr6	142378771	+	N	Y	N	N	N	N	transl_inter	na	NA	intergenic	RNU1-45P,RP11-84H6	-,+	snRNA,pseu	160297;9537	intergenic	RPS3AP23,RP11-137J7.	-,+	pseudogene	421131;5083
3550	chr4	11719072	+	chr6	57604993	-	Y	Y	N	N	N	N	transl_inter	na	NA	ncRNA_intr	RP11-281P23.2	+	lincRNA	0	intergenic	PRIM2,GAPDHP41	+,-	protein_cod	91618;81976
3551	chr4	68150856	+	chr6	160656412	-	Y	Y	N	Y	N	Y	transl_inter	na	NA	intergenic	RNU6-699P,RP11-584I	+,-	snRNA,lincF	387767;132167	intronic	SLC22A2	-	protein_cod	0
3552	chr4	6005888	-	chr7	48233675	-	N	N	N	N	N	Y	transl_inter	na	NA	intergenic	C4orf50,JAKMIP1	-,-	protein_cod	14343;22038	intronic	ABCA13	+	protein_cod	0
3553	chr4	9704831	-	chr7	20709422	-	N	N	Y	N	N	N	transl_inter	na	NA	downstream	FAM86MP,ALG1L3P	+,-	pseudogene	0	intronic	ABCB5	+	protein_cod	0
3554	chr4	33837031	-	chr7	57608334	-	N	N	N	N	Y	N	transl_inter	na	NA	ncRNA_intr	RP11-79E3.3	-	lincRNA	0	intergenic	RP11-1324A7.2,NCOR1F	+,+	pseudogene	62733;51166
3555	chr4	33843475	-	chr7	57613466	-	N	Y	N	N	N	N	transl_inter	na	NA	ncRNA_intr	RP11-79E3.3	-	lincRNA	0	intergenic	RP11-1324A7.2,NCOR1F	+,+	pseudogene	67865;46034
3556	chr4	39350031	-	chr7	28150150	-	N	Y	N	Y	N	Y	transl_inter	na	NA	intronic	RFC1	-	protein_cod	0	intronic	JAZF1	-	protein_cod	0
3557	chr4	88843882	-	chr7	83090042	-	N	Y	N	N	N	N	transl_inter	na	NA	intergenic	HSP90AB3P,SPP1	+,+	pseudogene	28715;52937	intronic	SEMA3E	-	protein_cod	0
3558	chr4	175884836	-	chr7	62473459	-	N	N	Y	Y	Y	N	transl_inter	na	NA	intronic	ADAM29	+	protein_cod	0	intergenic	RNU6-417P,RP11-196D1	-,-	snRNA,lincF	1512;31746
3559	chr4	191043572	-	chr7	159128671	-	N	N	N	N	Y	N	transl_inter	na	NA	intergenic	DUX4L2,NONE	+,NONE	protein_cod	29853;NONE	intergenic	PIP5K1P2,NONE	+,NONE	pseudogene	102604;NONE
3560	chr4	115442436	-	chr7	15394023	+	N	N	Y	N	N	Y	transl_inter	na	NA	intergenic	RP11-362M19.1,UGT8	+,+	pseudogene	417055;77175	intronic	AGMO	-	protein_cod	0
3561	chr4	66955034	+	chr7	136260958	+	Y	N	N	N	N	N	transl_inter	na	NA	ncRNA_intr	RP11-25H12.1	+	lincRNA	0	intergenic	AC009541.1,hsa-mir-490	+,-	pseudogene	87893;109348
3562	chr4	109844627	+	chr7	76044147	+	Y	Y	Y	Y	Y	Y	transl_inter	na	NA	intronic	COL25A1	-	protein_cod	0	intronic	ZP3	+	protein_cod	0
3563	chr4	191027672	-	chr8	146253608	-	N	N	N	N	N	Y	transl_inter	na	NA	intergenic;ir	DUX4L2,NONE	+,NONE	protein_cod	13953;NONE	intergenic;ir	ZNF252P-AS1,AC13910	+,+	antisense,p	22176;19638
3564	chr4	191027811	-	chr8	146253477	-	N	N	N	N	Y	N	transl_inter	na	NA	intergenic;ir	DUX4L2,NONE	+,NONE	protein_cod	14092;NONE	intergenic;ir	ZNF252P-AS1,AC13910	+,+	antisense,p	22045;19769
3565	chr4	191043760	-	chr8	146300855	-	Y	N	N	N	N	Y	transl_inter	na	NA	intergenic	DUX4L2,NONE	+,NONE	protein_cod	30041;NONE	intergenic;ir	C8orf33,NONE	+,NONE	protein_cod	19439;NONE
3566	chr4	177613256	-	chr8	73065385	+	N	N	Y	Y	N	N	transl_inter	na	NA	ncRNA_intr	RP11-313E19.2	+	antisense	0	intergenic	TRPA1,RP11-142A23.1	-,+	protein_cod	77533;43184
3567	chr4	95990463	+	chr8	5224791	+	Y	Y	N	Y	Y	Y	transl_inter	na	NA	intronic;intrc	BMPR1B	+	protein_cod	0	intergenic;ir	RN7SL318P,RP11-745K9	-,-	misc_RNA,p	224562;87949
3568	chr4	191027672	+	chr8	146253608	+	N	Y	N	N	Y	N	transl_inter	na	NA	intergenic;ir	DUX4L2,NONE	+,NONE	protein_cod	13953;NONE	intergenic;ir	ZNF252P-AS1,AC13910	+,+	antisense,p	22176;19638
3569	chr4	159355363	-	chr9	18714806	-	N	N	N	N	Y	N	transl_inter	na	NA	intronic	RXFP1	+	protein_cod	0	intronic	ADAMTSL1	+	protein_cod	0
3570	chr4	171598998	-	chr9	109550767	-	Y	N	N	N	N	N	transl_inter	na	NA	intergenic	HSP90AA6P,RP11-322	-,-	pseudogene	72397;64622	intergenic	RP11-308N19.5,RNA5SF	+,-	pseudogene	65556;55263
3571	chr4	171599195	-	chr9	109550876	-	N	N	N	N	Y	N	transl_inter	na	NA	intergenic	HSP90AA6P,RP11-322	-,-	pseudogene	72594;64425	intergenic	RP11-308N19.5,RNA5SF	+,-	pseudogene	65665;55154
3572	chr4	190194847	-	chr9	68420017	-	N	N	N	N	Y	N	transl_inter	na	NA	intergenic	RP11-706F1.1,RP11-7	+,-	lincRNA,linc	68816;14984	intergenic	MIR4477A,RP11-764K9.4	+,-	miRNA,pseu	4629;7844
3573	chr4	190197882	-	chr9	68424607	-	N	N	Y	N	N	N	transl_inter	na	NA	intergenic	RP11-706F1.1,RP11-7	+,-	lincRNA,linc	71851;11949	intergenic	MIR4477A,RP11-764K9.4	+,-	miRNA,pseu	9219;3254
3574	chr4	190197983	-	chr9	68424688	-	N	N	N	Y	N	N	transl_inter	na	NA	intergenic	RP11-706F1.1,RP11-7	+,-	lincRNA,linc	71952;11848	intergenic	MIR4477A,RP11-764K9.4	+,-	miRNA,pseu	9300;3173
3575	chr4	190200118	-	chr9	68425910	-	N	N	N	N	N	Y	transl_inter	na	NA	intergenic	RP11-706F1.1,RP11-7	+,-	lincRNA,linc	74087;9713	intergenic	MIR4477A,RP11-764K9.4	+,-	miRNA,pseu	10522;1951
3576	chr4	14776617	+	chr9	76898393	-	Y	Y	N	Y	Y	Y	transl_inter	na	NA	ncRNA_intr	LINC00504	-	lincRNA	0	intergenic	RP11-401G5.1,RP11-171	-,+	pseudogene	410180;88054
3577	chr4	190819465	+	chr9	68491996	-	N	N	Y	N	N	N	transl_inter	na	NA	ncRNA_intr	AF146191.4	-	lincRNA	0	intergenic	RP11-764K9.4,CR78658	-,+	pseudogene	36621;20350
3578	chr4	171599149	+	chr9	109550927	+	N	Y	N	N	N	N	transl_inter	na	NA	intergenic	HSP90AA6P,RP11-322	-,-	pseudogene	72548;64471	intergenic	RP11-308N19.5,RNA5SF	+,-	pseudogene	65716;55103
3579	chr4	48240980	-	chrX	143557292	+	N	Y	N	N	N	N	transl_inter	na	NA	intronic	TEC	-	protein_cod	0	intergenic	RRM2P4,HNRNPCP10	+,+	pseudogene	164474;365006
3580	chr4	171010218	+	chrX	76653692	-	N	N	N	Y	N	N	transl_inter	na	NA	intronic	AADAT	-	protein_cod	0	intergenic	BX682235.1,FGF16	+,+	pseudogene	51910;55956
3581	chr4	11719073	+	chrX	14455922	+	Y	Y	N	Y	Y	N	transl_inter	na	NA	ncRNA_intr	RP11-281P23.2	+	lincRNA	0	intergenic	GS1-257G1.1,GLRA2	+,+	pseudogene	192378;91498
3582	chr4	70945398	+	chrX	36729741	+	N	Y	N	N	N	Y	transl_inter	na	NA	ncRNA_intr	CSN1S2AP	+	pseudogene	0	intergenic;ir	AL590285.1,RP11-492O8	+,+	miRNA,pseu	134176;8250
3583	chr4	70945713	+	chrX	36729741	+	Y	N	N	N	Y	N	transl_inter	na	NA	ncRNA_intr	CSN1S2AP	+	pseudogene	0	intergenic;ir	AL590285.1,RP11-492O8	+,+	miRNA,pseu	134176;8250
3584	chr4	49268613	+	chrY	13267548	-	N	N	N	N	N	Y	transl_inter	na	NA	intergenic	RP11-1281K21.7,RP11	+,-	pseudogene	19681;220330	intergenic	RP1-85D24.1,AC134878	-,-	pseudogene	3985;72811
3585	chr5	110284081	-	chr6	136582615	-	N	Y	N	N	Y	Y	transl_inter	na	NA	ncRNA_exo	CTD-2089O24.1	-	pseudogene	0	exonic;exon	BCLAF1	-	protein_cod	0
3586	chr5	110286232	-	chr6	136599957	-	N	N	Y	N	N	N	transl_inter	na	NA	ncRNA_exo	CTD-2089O24.1	-	pseudogene	0	intronic	BCLAF1	-	protein_cod	0
3587	chr5	124755628	-	chr6	41186584	-	N	Y	N	N	N	N	transl_inter	na	NA	intergenic	RP11-395P13.6,RP11-	-,+	lincRNA,linc	23955;73326	ncRNA_intr	TREML3P	-	pseudogene	0
3588	chr5	49863695	-	chr6	94741076	+	N	Y	N	N	N	N	transl_inter	na	NA	intergenic	EMB,RP11-269M20.2	-,+	protein_cod	126494;35368	intergenic	RP11-524K14.1,RP3-463	+,+	lincRNA,linc	141948;132562
3589	chr5	146587498	-	chr6	42230183	+	N	N	N	N	Y	Y	transl_inter	na	NA	ncRNA_intr	CTC-255N20.1	-	antisense	0	intronic	TRERF1	-	protein_cod	0
3590	chr5	21573436	+	chr6	57575918	-	Y	Y	Y	Y	Y	Y	transl_inter	na	NA	ncRNA_intr	GUSBP1	+	pseudogene	0	intergenic	PRIM2,GAPDHP41	+,-	protein_cod	62543;111051
3591	chr5	109480266	+	chr6	117429372	-	Y	Y	N	Y	Y	Y	transl_inter	na	NA	intergenic	PGAM5P1,NONE	-,NONE	pseudogene	259142;NONE	intergenic	RNA5SP214,VGLL2	+,+	rRNA,protei	47410;157349
3592	chr5	13827596	+	chr6	58204340	+	Y	Y	N	N	Y	Y	transl_inter	na	NA	intronic;intrc	DNAH5	-	protein_cod	0	intergenic;ir	XXbac-BPGBPG55C20.1	-,-	lincRNA,linc	21594;24547
3593	chr5	110284081	+	chr6	136582615	+	N	N	Y	N	N	N	transl_inter	na	NA	ncRNA_exo	CTD-2089O24.1	-	pseudogene	0	exonic;exon	BCLAF1	-	protein_cod	0
3594	chr5	110286602	+	chr6	136610859	+	Y	N	N	N	N	N	transl_inter	na	NA	upstream	CTD-2089O24.1	-	pseudogene	0	UTR5	BCLAF1	-	protein_cod	0
3595	chr5	110286625	+	chr6	136610882	+	N	N	Y	N	N	N	transl_inter	na	NA	upstream	CTD-2089O24.1	-	pseudogene	0	UTR5	BCLAF1	-	protein_cod	0
3596	chr5	110286690	+	chr6	136610817	+	N	Y	N	N	Y	N	transl_inter	na	NA	upstream	CTD-2089O24.1	-	pseudogene	0	intronic	BCLAF1	-	protein_cod	0
3597	chr5	8089827	+	chr7	32505381	-	N	N	Y	N	Y	N	transl_inter	na	NA	intergenic	RNU1-76P,RP11-215I1	+,-	snRNA,pseu	109410;67827	intergenic	AC018641.7,SLC25A5P5	-,+	pseudogene	8011;5744
3598	chr5	18699136	+	chr7	142014574	-	Y	N	N	N	N	N	transl_inter	na	NA	intergenic	RN7SL58P,CTD-2023N	+,-	misc_RNA,l	151424;5406	intergenic;ir	TRBV4-1,TRBV5-1	+,+	TR_V_gene	1085;5922
3599	chr5	162236961	+	chr7	154943266	+	N	N	Y	N	N	N	transl_inter	na	NA	intergenic	ARL2BPP5,RP11-541F	+,-	pseudogene	47642;202310	intergenic	AC092628.3,RN7SKP28(	+,+	pseudogene	44905;30325
3600	chr5	55148122	-	chr8	145894748	+	N	N	N	Y	N	N	transl_inter	na	NA	UTR5	IL31RA	+	protein_cod	0	intronic	ARHGAP39	-	protein_cod	0
3601	chr5	59633205	-	chr8	9144649	+	N	N	Y	N	Y	N	transl_inter	na	NA	intronic	PDE4D	-	protein_cod	0	ncRNA_intr	RP11-115J16.1	+	lincRNA	0
3602	chr5	70848196	-	chr8	94051225	+	N	N	N	Y	N	N	transl_inter	na	NA	intronic	BDP1	+	protein_cod	0	intergenic	TRIQK,C8orf87	-,-	protein_cod	21324;95099
3603	chr5	39787752	-	chr9	7741406	-	Y	Y	Y	Y	Y	Y	transl_inter	na	NA	intergenic	INTS6P1,GCSHP1	-,-	pseudogene	66137;100926	intergenic	RP11-366I21.1,RP11-77E	+,-	pseudogene	143029;44699
3604	chr5	3446348	+	chr9	138225486	+	Y	N	N	N	N	N	transl_inter	na	NA	ncRNA_intr	LINC01019	-	lincRNA	0	intergenic	AL390778.1,C9orf62	-,+	protein_cod	74211;9609
3605	chr5	180321191	+	chr9	21127655	+	N	N	N	N	Y	N	transl_inter	na	NA	intergenic	ZFP62,BTNL8	-,+	protein_cod	32905;4886	intergenic	IFNWP4,IFNW1	+,-	pseudogene	20820;12976
3606	chr5	7230442	-	chrX	30639866	-	N	N	N	Y	N	Y	transl_inter	na	NA	intergenic	RP11-122F24.1,RP11-	+,-	lincRNA,pse	39517;57595	intergenic	CKS1BP6,FTLP2	+,+	pseudogene	4056;8549

3607	chr5	91686129	-	chrX	95087573	-	N	N	N	Y	N	N	transl_inter	na	NA	ncRNA_intr	RP11-348J24.2	+	lincRNA	0	intergenic	RP11-471G13.5,RP5-961	+,-	pseudogene	111430;140754
3608	chr5	131452633	-	chrX	75379508	-	N	N	N	Y	N	N	transl_inter	na	NA	intergenic	snoZ6,AC063976.1	+,-	snoRNA,pse	36807;30551	intergenic	RP3-481A17.1,PBDC1	+,+	pseudogene	129343;13263
3609	chr5	91635949	+	chrX	51714511	-	N	N	Y	Y	N	Y	transl_inter	na	NA	ncRNA_intr	RP11-348J24.2	+	lincRNA	0	intergenic	RP11-234P3.4,TPMTP3	+,-	pseudogene	47675;7808
3610	chr5	174058012	+	chrX	126744477	-	Y	Y	Y	N	Y	N	transl_inter	na	NA	intergenic	RP11-267A15.3,HIGD	+,+	lincRNA,pse	5234;1923	intergenic	RP3-428A13.1,CTD-207	+,-	pseudogene	217969;50135
3611	chr5	153327690	+	chrX	121274244	+	N	N	N	N	N	N	transl_inter	na	NA	intergenic	RP11-461L18.1,FAM11	+,-	lincRNA,pro	44907;41998	intergenic	RP11-417D4.1,AL513487	-,-	pseudogene	128779;331334
3612	chr6	114862579	-	chr7	80672613	+	N	N	Y	N	N	N	transl_inter	na	NA	intergenic	RP3-399L15.2,RNU6-	-,-	lincRNA,snR	5540;325458	intergenic	SEMA3C,AC005008.2	-,+	protein_cod	120938;132211
3613	chr6	144120424	-	chr7	153002532	+	Y	N	N	N	N	N	transl_inter	na	NA	intronic	PHACTR2	+	protein_cod	0	intergenic	AC079809.2,AC073236.	-,-	pseudogene	160859;94473
3614	chr6	144120502	-	chr7	153002458	+	N	N	Y	N	N	N	transl_inter	na	NA	intronic	PHACTR2	+	protein_cod	0	intergenic	AC079809.2,AC073236.	-,-	pseudogene	160785;94547
3615	chr6	13947500	+	chr7	46567002	-	N	Y	N	N	Y	Y	transl_inter	na	NA	intronic	RNF182	+	protein_cod	0	intergenic	AC004869.2,HMGN1P19	-,+	lincRNA,pse	45826;107210
3616	chr6	13947936	+	chr7	46567443	-	N	N	Y	Y	N	N	transl_inter	na	NA	intronic	RNF182	+	protein_cod	0	intergenic	AC004869.2,HMGN1P19	-,+	lincRNA,pse	46267;106769
3617	chr6	47396994	+	chr7	130372012	-	N	N	N	Y	N	N	transl_inter	na	NA	intergenic	RP11-157D6.1,AL3553	+,-	pseudogene	60028;33593	UTR5	TSGA13	-	protein_cod	0
3618	chr6	144120748	+	chr7	153002102	-	N	N	N	Y	N	N	transl_inter	na	NA	intronic	PHACTR2	+	protein_cod	0	intergenic	AC079809.2,AC073236.	-,-	pseudogene	160429;94903
3619	chr6	167365347	+	chr7	142014574	-	N	Y	Y	N	Y	N	transl_inter	na	NA	intronic	RNASET2,RP11-514O	-,-	protein_cod	0	intergenic;ir	TRBV4-1,TRBV5-1	+,+	TR_V_gene	1085;5922
3620	chr6	24812046	+	chr7	93146611	+	N	N	N	Y	N	N	transl_inter	na	NA	intronic	FAM65B	-	protein_cod	0	intronic	CALCR	-	protein_cod	0
3621	chr6	36003386	+	chr7	124879111	+	Y	Y	Y	N	N	Y	transl_inter	na	NA	intronic	MAPK14	+	protein_cod	0	ncRNA_intr	RP11-3B12.3,RP11-3B12	-,+	lincRNA,linc	0
3622	chr6	129706247	+	chr7	71888533	+	Y	N	Y	N	N	N	transl_inter	na	NA	intronic	LAMA2	+	protein_cod	0	intronic	CALN1	-	protein_cod	0
3623	chr6	150353710	+	chr7	7062416	+	N	N	N	N	N	Y	transl_inter	na	NA	upstream	RAET1M	+	pseudogene	0	intergenic	AC079804.1,AC092104.	+,-	miRNA,miR	27467;43826
3624	chr6	160640135	+	chr7	114270067	+	Y	N	N	N	N	N	transl_inter	na	NA	intronic	SLC22A2	-	protein_cod	0	intronic	FOXP2	+	protein_cod	0
3625	chr6	43962298	-	chr8	14835895	-	N	N	Y	N	N	N	transl_inter	na	NA	intergenic	RP11-344J7.2,RP5-11	-,-	lincRNA,ant	142228;1162	intronic	SGCZ	-	protein_cod	0
3626	chr6	118142597	-	chr8	141361286	-	N	N	Y	Y	N	N	transl_inter	na	NA	intergenic	NUS1,SLC35F1	+,+	protein_cod	110794;86092	intronic	TRAPPC9	-	protein_cod	0
3627	chr6	132467953	-	chr8	116410955	-	N	N	Y	N	N	N	transl_inter	na	NA	ncRNA_exc	LINC01013	+	lincRNA	0	intergenic;ir	RP11-192P9.1,TRPS1	-,-	pseudogene	606797;9769
3628	chr6	153748709	-	chr8	5656494	-	N	N	N	N	Y	N	transl_inter	na	NA	intergenic	RNA5SP225,RP11-33	+,+	rRNA,pseuc	7002;238133	intergenic	RP11-281H11.1,RP11-72	-,+	lincRNA,pse	128895;49725
3629	chr6	57603490	-	chr8	71720588	+	N	N	N	N	N	Y	transl_inter	na	NA	intergenic	PRIM2,GAPDHP41	+,-	protein_cod	90115;83479	intergenic	Y_RNA,RP11-326E22.1	-,+	misc_RNA,	27345;347104
3630	chr6	27759148	+	chr8	73959115	+	Y	Y	Y	Y	Y	Y	transl_inter	na	NA	intergenic	RSL24D1P1,HIST1H4	-,+	pseudogene	10258;15705	UTR3	TERF1	+	protein_cod	0
3631	chr6	43962452	+	chr8	14835894	+	N	N	N	N	Y	N	transl_inter	na	NA	intergenic	RP11-344J7.2,RP5-11	-,-	lincRNA,ant	142382;1008	intronic	SGCZ	-	protein_cod	0
3632	chr6	110181726	+	chr8	146184564	+	N	Y	N	N	N	N	transl_inter	na	NA	intergenic	RP1-249I4.2,GPR6	+,+	lincRNA,pro	31738;117788	intergenic	ZNF16,ZNF252P	-,-	protein_cod	8290;14411
3633	chr6	132467953	+	chr8	116410955	+	N	Y	Y	Y	N	Y	transl_inter	na	NA	ncRNA_exc	LINC01013	+	lincRNA	0	intergenic;ir	RP11-192P9.1,TRPS1	-,-	pseudogene	606797;9769
3634	chr6	13128406	-	chr9	21841794	-	Y	Y	Y	Y	Y	Y	transl_inter	na	NA	intronic	PHACTR1	+	protein_cod	0	intronic	MTAP,RP11-145E5.5	+,+	protein_cod	0
3635	chr6	152217773	+	chr9	97340216	+	N	N	N	N	Y	N	transl_inter	na	NA	intronic	ESR1	+	protein_cod	0	intronic	FBP2	-	protein_cod	0
3636	chr6	89435091	-	chrX	119277813	-	Y	N	N	N	N	N	transl_inter	na	NA	intronic	RNGTT	-	protein_cod	0	ncRNA_intr	NKAPP1,RP4-755D9.1	-,+	pseudogene	0
3637	chr6	89435365	-	chrX	119277793	-	N	N	N	Y	Y	Y	transl_inter	na	NA	intronic;intr	RNGTT	-	protein_cod	0	ncRNA_exc	RP4-755D9.1	+	antisense	0
3638	chr6	148991012	-	chrX	69737065	-	Y	N	Y	N	N	N	transl_inter	na	NA	intergenic	RP11-461H3.2,UST	-,+	pseudogene	21279;77452	intergenic	DLG3,TEX11	+,-	protein_cod	11728;11725
3639	chr6	160656534	-	chrX	108796416	-	N	Y	Y	N	Y	Y	transl_inter	na	NA	intronic	SLC22A2	-	protein_cod	0	intergenic	NXT2,RP1-136J15.5	+,+	protein_cod	8497;15291
3640	chr6	170664467	-	chrX	54392713	-	N	N	N	Y	N	N	transl_inter	na	NA	intronic	FAM120B	+	protein_cod	0	intergenic	WNK3,TSR2	-,+	protein_cod	7638;74121
3641	chr6	63720560	-	chrX	102685356	+	Y	N	N	N	N	N	transl_inter	na	NA	intergenic	AL590558.1,RP11-184	-,-	miRNA,pseu	27485;182417	intergenic	NGFRAP1,LL0XNC01-25	+,+	protein_cod	52351;67095
3642	chr6	89435365	+	chrX	119277793	+	N	N	N	Y	N	Y	transl_inter	na	NA	intronic;intr	RNGTT	-	protein_cod	0	ncRNA_exc	RP4-755D9.1	+	antisense	0
3643	chr6	89435383	+	chrX	119277935	+	N	N	N	N	Y	N	transl_inter	na	NA	intronic	RNGTT	-	protein_cod	0	ncRNA_intr	NKAPP1,RP4-755D9.1	-,+	pseudogene	0
3644	chr6	170911097	+	chrY	23901402	-	N	Y	N	Y	Y	Y	transl_inter	na	NA	intergenic	PDCD2,OR4F7P	-,+	protein_cod	17317;37597	intergenic	CDY11P,AC007322.1	+,+	pseudogene	41296;116076
3645	chr6	76821355	+	chrY	9951878	+	Y	Y	Y	N	Y	Y	transl_inter	na	NA	intergenic	RNU6-248P,RNU6-261	+,-	snRNA,snR	18698;335350	intergenic;ir	RNA5SP519,AC006987.	-,-	rRNA,pseuc	21276;55519
3646	chr7	2889934	-	chr8	143267234	-	N	N	N	N	N	Y	transl_inter	na	NA	intergenic	GNA12,CARD11	-,-	protein_cod	5976;55841	intergenic	MIR4472-1,LINC00051	+,+	miRNA,antis	9455;12483
3647	chr7	2890358	-	chr8	143267120	-	N	N	Y	N	N	N	transl_inter	na	NA	intergenic	GNA12,CARD11	-,-	protein_cod	6400;55417	intergenic	MIR4472-1,LINC00051	+,+	miRNA,antis	9341;12597
3648	chr7	14511052	-	chr8	38902944	-	N	N	N	N	Y	N	transl_inter	na	NA	intronic	DGKB	-	protein_cod	0	intronic	ADAM9	+	protein_cod	0
3649	chr7	63818216	-	chr8	47706457	-	Y	N	N	Y	N	N	transl_inter	na	NA	intergenic	TRIM60P18,YWHAEP	+,+	pseudogene	1639;75992	intergenic	TRIM60P15,LINC00293	+,+	pseudogene	1771;27402
3650	chr7	16830671	-	chr8	134769343	+	Y	N	N	N	N	N	transl_inter	na	NA	downstream	AGR2	-	protein_cod	0	downstream	CTD-2309H9.2	+	pseudogene	0
3651	chr7	100938136	-	chr8	114583754	+	Y	N	Y	Y	Y	Y	transl_inter	na	NA	intergenic	AZGP1P2,RP11-132A	-,+	pseudogene	5084;4758	intergenic	CSMD3,RP11-67H2.1	-,+	protein_cod	134426;28785
3652	chr7	100939180	-	chr8	37502878	+	Y	N	N	N	N	Y	transl_inter	na	NA	intergenic;ir	AZGP1P2,RP11-132A	-,+	pseudogene	6128;3714	ncRNA_intr	RP11-346L1.2	-	antisense	0
3653	chr7	52478344	+	chr8	54528719	-	N	N	N	Y	N	N	transl_inter	na	NA	intergenic	snoU13,RP11-398K14.	-,-	snoRNA,pse	86759;481186	intergenic	RP11-400K9.2,RP11-463	+,+	pseudogene	75859;96538
3654	chr7	63239408	+	chr8	128024315	-	N	N	N	N	N	Y	transl_inter	na	NA	intergenic	CICP24,CTD-2526L21	-,+	pseudogene	7983;10879	ncRNA_intr	PCAT1	+	lincRNA	0
3655	chr7	100939180	+	chr8	37502878	-	N	N	N	N	Y	N	transl_inter	na	NA	intergenic;ir	AZGP1P2,RP11-132A	-,+	pseudogene	6128;3714	ncRNA_intr	RP11-346L1.2	-	antisense	0
3656	chr7	63818177	+	chr8	47706706	+	N	N	N	N	N	Y	transl_inter	na	NA	intergenic	TRIM60P18,YWHAEP	+,+	pseudogene	1600;76031	intergenic	TRIM60P15,LINC00293	+,+	pseudogene	2020;27153
3657	chr7	63818200	+	chr8	47706439	+	Y	N	N	N	N	N	transl_inter	na	NA	intergenic	TRIM60P18,YWHAEP	+,+	pseudogene	1623;76008	intergenic	TRIM60P15,LINC00293	+,+	pseudogene	1753;27420
3658	chr7	66083944	-	chr9	68418844	-	Y	N	Y	N	Y	N	transl_inter	na	NA	intergenic	RPL35P5,KCTD7	-,+	pseudogene	11850;9924	intergenic;ir	MIR4477A,RP11-764K9.	+,-	miRNA,pseu	3456;9017
3659	chr7	142192393	-	chr9	130185384	-	N	Y	Y	N	N	N	transl_inter	na	NA	intergenic	TRBV12-2,TRBV11-2	-,-	TR_V_pseu	1313;5177	intergenic	SLC2A8,ZNF79	+,+	protein_cod	15207;1277
3660	chr7	124616084	-	chr9	21127565	+	N	N	N	N	N	Y	transl_inter	na	NA	ncRNA_intr	POT1-AS1	+	antisense	0	intergenic	IFNWP4,IFNW1	+,-	pseudogene	20730;13066
3661	chr7	48030236	+	chr9	68418844	-	Y	N	Y	N	N	N	transl_inter	na	NA	intronic	SUN3	-	protein_cod	0	intergenic;ir	MIR4477A,RP11-764K9.	+,-	miRNA,pseu	3456;9017
3662	chr7	62471645	+	chr9	19043007	-	N	Y	Y	N	N	Y	transl_inter	na	NA	downstream	RNU6-417P	-	snRNA	0	intergenic	FAM154A,RRAGA	-,+	protein_cod	9756;6365
3663	chr7	65060304	+	chr9	69810212	+	Y	N	Y	Y	N	Y	transl_inter	na	NA	intergenic	RP11-667F9.1,RP13-1	-,+	pseudogene	40647;21686	intergenic	IGKV1OR-2,AL359955.1	-,-	IG_V_pseu	32259;11548
3664	chr7	65060337	+	chr9	69810181	+	N	N	N	N	Y	N	transl_inter	na	NA	intergenic	RP11-667F9.1,RP13-1	-,+	pseudogene	40680;21653	intergenic	IGKV1OR-2,AL359955.1	-,-	IG_V_pseu	32228;11579
3665	chr7	142468073	+	chr9	33795555	+	N	N	N	N	Y	N	transl_inter	na	NA	upstream	PRSS3P1	+	pseudogene	0	ncRNA_intr	RP11-133O22.6	-	antisense	0
3666	chr7	1238379	-	chrX	40774305	-	Y	N	N	N	N	N	transl_inter	na	NA	intergenic	AC091729.9,UNCX	+,+	antisense,pr	33476;34164	intergenic	RP11-77G22.2,RPS2P55	+,-	pseudogene	6661;19893
3667	chr7	19518602	-	chrX	18757244	-	Y	Y	N	N	N	N	transl_inter	na	NA	ncRNA_intr	AC007091.1	+	lincRNA	0	intronic;intr	PPEF1	+	protein_cod	0
3668	chr7	19518811	-	chrX	18757244	-	N	N	N	N	Y	N	transl_inter	na	NA	ncRNA_intr	AC007091.1	+	lincRNA	0	intronic;intr	PPEF1	+	protein_cod	0
3669	chr7	45032187	-	chrX	53749052	+	N	N	N	Y	N	Y	transl_inter	na	NA	intergenic	SNHG15,CCM2	-,+	lincRNA,pro	5627;7597	intergenic	HUWE1,RP11-272G22.1	-,+	protein_cod	35379;36472
3670	chr7	45032249	-	chrX	53748644	+	N	N	Y	N	N	N	transl_inter	na	NA	intergenic	SNHG15,CCM2	-,+	lincRNA,pro	5689;7535	intergenic	HUWE1,RP11-272G22.1	-,+	protein_cod	34971;36880
3671	chr7	62472988	-	chrX	54178455	+	Y	Y	Y	Y	N	N	transl_inter	na	NA	intergenic	RNU6-417P,RP11-196	-,-	snRNA,linc	1041;32217	intronic	FAM120C	-	protein_cod	0
3672	chr7	64955026	-	chrY	58978347	-	N	N	N	N	Y	N	transl_inter	na	NA	intergenic	RP11-667F9.2,RP11-3	+,-	lincRNA,pse	26675;18660	intergenic	NONE,CTBP2P1	NONE,+	NONE,pseu	NONE;23044
3673	chr7	64955559	-	chrY	58978359	-	N	N	Y	N	N	N	transl_inter	na	NA	intergenic	RP11-667F9.2,RP11-3	+,-	lincRNA,pse	27208;18127	intergenic	NONE,CTBP2P1	NONE,+	NONE,pseu	NONE;23032
3674	chr7	64955739	-	chrY	58978537	-	N	N	N	Y	N	N	transl_inter	na	NA	intergenic	RP11-667F9.2,RP11-3	+,-	lincRNA,pse	27388;17947	intergenic	NONE,CTBP2P1	NONE,+	NONE,pseu	NONE;22854
3675	chr7	29680031	-	chrY	6759794	+	N	N	N	N	N	Y	transl_inter	na	NA	ncRNA_intr	ZNRF2P2	-	pseudogene	0	intergenic	AMELY,ATP5JP1	-,+	protein_cod	17726;9000
3676	chr7	57547301	-	chrY	58978365	+	N	N	N	N	N	Y	transl_inter	na	NA	intergenic	RP11-1324A7.2,NCOR	+,+	pseudogene	1700;112199	intergenic	NONE,CTBP2P1	NONE,+	NONE,pseu	NONE;23026
3677	chr7	57547381	-	chrY	58978563	+	Y	N	N	N	N	N	transl_inter	na	NA	intergenic	RP11-1324A7.2,NCOR	+,+	pseudogene	1780;112119	intergenic	NONE,CTBP2P1	NONE,+	NONE,pseu	NONE;22828
3678	chr7	57547574	+	chrY	58978538	-	N	N	N	N	Y	N	transl_inter	na	NA	intergenic	RP11-1324A7.2,NCOR	+,+	pseudogene	1973;111926	intergenic	NONE,CTBP2P1	NONE,+	NONE,pseu	NONE;22853
3679	chr8	39508313	-	chr9	68418844	-	N	N	N	N	N	Y	transl_inter	na	NA	intronic	ADAM18	+	protein_cod	0	intergenic;ir	MIR4477A,RP11-764K9.	+,-	miRNA,pseu	3456;9017
3680	chr8	30597969	+	chr9	117837816	+	N	N	N	N	Y	N	transl_inter	na	NA	ncRNA_intr	UBXN8	+	processed_	0	intronic	TNC	-	protein_cod	0
3681	chr8	60451973	-	chrX	11731429	-	Y	N	N	N	N	Y	transl_inter	na	NA	intergenic	RNA5SP267,RP11-37	+,-	rRNA,pseuc	83404;18114	intergenic	GS1-590J6.2,MSL3	-,+	pseudogene	7719;44849
3682	chr8	94524001	-	chrX	78455982	-	N	N	N	N	Y	N	transl_inter	na	NA	ncRNA_intr	LINC00535	-	antisense	0	intergenic	RP11-159L8.2,KIF4CP	-,+	pseudogene	22817;122961
3683	chr9	132205202	-	chrX	71953981	-	Y	Y	N	N	N	N	transl_inter	na	NA	ncRNA_intr	RP11-65J3.3	-	lincRNA	0	intergenic	RP1-172N19.4,RNU1-11	-,-	pseudogene	5335;6549
3684	chr9	37808419	-	chrX	101814393	+	N	Y	N	N	N	N	transl_inter	na	NA	intronic	DCAF10,RP11-613M1	+,-	protein_cod	0	ncRNA_intr	NXF4	+	pseudogene	0
3685	chr9	37808426	-	chrX	101814368	+	N	N	N	Y	N	Y	transl_inter	na	NA	intronic	DCAF10,RP11-613M1	+,-	protein_cod	0	ncRNA_intr	NXF4	+	pseudogene	0
3686	chr9	130644826	-	chrX	65296781	+	N	Y	N	N	N	Y	transl_inter	na	NA	ncRNA_intr	RP11-203J24.9	-	processed_	0	upstream	EIF4BP9	-	pseudogene	0
3687	chr9	132205203	-	chrX	138424074	+	N	N	N	Y	N	N	transl_inter	na	NA	ncRNA_intr	RP11-65J3.3	-	lincRNA	0	intergenic	FGF13,SNURFL	-,+	protein_cod	119135;20125
3688	chr9	66458213	-	chrY	9930499	-	N	N	Y	Y	N	N	transl_inter	na	NA	ncRNA_exc	RNA5SP283	-	rRNA	0	ncRNA_exc	RNA5SP519	-	rRNA	0
3689	chr9	66470709	-	chrY	9943121	-	N	Y	N	N	N	N	transl_inter	na	NA	intergenic	RP11-262H14.1,AL512	+,+	lincRNA,pse	1399;2666	intergenic	RNA5SP519,AC006987.	-,-	rRNA,pseuc	12519;64276
3690	chr9	68413506	-	chrY	10013553	-	N	N	N	Y	N	Y	transl_inter	na	NA	ncRNA_exc	LINC00537	-	pseudogene	0	intergenic	PCMTD1P1,CDC27P2	+,-	pseudogene	1737;14433

3691	chr9	68393484	-	chrY	9945980	+	N	N	N	Y	N	N	transl_inter	na	NA	intergenic	PTGER4P3,RP11-764	-,-	pseudogen	21607;4394	intergenic	RNA5SP519,AC006987.	-,-	rRNA,pseu	15378;61417
3692	chr9	68404526	-	chrY	9934928	+	N	N	N	N	Y	N	transl_inter	na	NA	ncRNA_intr	RP11-764K9.1	-	lincRNA	0	intergenic	RNA5SP519,AC006987.	-,-	rRNA,pseu	4326;72469
3693	chr9	68404580	-	chrY	9934900	+	N	N	Y	N	N	N	transl_inter	na	NA	ncRNA_intr	RP11-764K9.1	-	lincRNA	0	intergenic	RNA5SP519,AC006987.	-,-	rRNA,pseu	4298;72497
3694	chr9	69000623	-	chrY	9957176	+	N	N	Y	N	N	N	transl_inter	na	NA	intergenic	AL353763.2,AL353763	-,-	miRNA,miR	3139;1376	intergenic	RNA5SP519,AC006987.	-,-	rRNA,pseu	26574;50221
3695	chr9	68396459	+	chrY	9943015	-	N	Y	N	N	N	N	transl_inter	na	NA	intergenic	PTGER4P3,RP11-764	-,-	pseudogen	24582;1419	intergenic	RNA5SP519,AC006987.	-,-	rRNA,pseu	12413;64382
3696	chr9	66457697	+	chrY	9930001	+	Y	N	N	N	N	N	transl_inter	na	NA	ncRNA_intr	RP11-262H14.1	+	lincRNA	0	downstrear	RNA5SP519	-	rRNA	0
3697	chr9	66462540	+	chrY	9935037	+	N	N	N	Y	N	N	transl_inter	na	NA	ncRNA_intr	RP11-262H14.1	+	lincRNA	0	intergenic	RNA5SP519,AC006987.	-,-	rRNA,pseu	4435;72360
3698	chr9	68418788	+	chrY	9961756	+	N	N	N	N	N	Y	transl_inter	na	NA	intergenic	MIR4477A,RP11-764K	+,-	miRNA,pseu	3400;9073	intergenic	RNA5SP519,AC006987.	-,-	rRNA,pseu	31154;45641
3699	chr9	68418844	+	chrY	9962011	+	Y	N	N	N	N	N	transl_inter	na	NA	intergenic	MIR4477A,RP11-764K	+,-	miRNA,pseu	3456;9017	intergenic	RNA5SP519,AC006987.	-,-	rRNA,pseu	31409;45386
3700	chrX	88461979	-	chrY	6402258	-	N	N	N	N	Y	N	transl_inter	na	NA	intergenic	RP13-324D24.1,SRIP	-,-	pseudogen	224625;160455	intergenic	TSPY17P,SRIP3	+,-	pseudogen	13288;184745
3701	chrX	88462160	-	chrY	6402635	-	N	Y	N	N	N	N	transl_inter	na	NA	intergenic	RP13-324D24.1,SRIP	-,-	pseudogen	224806;160274	intergenic	TSPY17P,SRIP3	+,-	pseudogen	13665;184368
3702	chrX	2832367	-	chrY	14491286	+	Y	Y	N	N	N	N	transl_inter	na	NA	intronic	ARSD	-	protein_cod	0	ncRNA_intr	GYG2P1,ARSDP1	-,+	pseudogen	0
3703	chrX	2832405	-	chrY	14491286	+	N	N	N	N	Y	N	transl_inter	na	NA	intronic	ARSD	-	protein_cod	0	ncRNA_intr	GYG2P1,ARSDP1	-,+	pseudogen	0
3704	chrX	3351404	-	chrY	7431237	+	Y	N	N	N	N	N	transl_inter	na	NA	intergenic	ASS1P4,SNORA48	+,-	pseudogen	64981;98754	intergenic	RNU6-521P,RBMY2HP	-,-	snRNA,pseu	140038;108547
3705	chrX	42510334	-	chrY	9950347	+	N	N	Y	N	N	N	transl_inter	na	NA	ncRNA_intr	RP1-154K9.2	+	lincRNA	0	intergenic	RNA5SP519,AC006987.	-,-	rRNA,pseu	19745;57050
3706	chrX	3196115	+	chrY	14172715	-	Y	Y	Y	N	N	Y	transl_inter	na	NA	intronic	CXorf28	+	protein_cod	0	intergenic	MXRA5P1,RPS24P1	+,+	pseudogen	64623;192742
3707	chrX	4965678	+	chrY	18680348	-	N	Y	Y	Y	N	N	transl_inter	na	NA	intergenic	AC074035.1,MTND6P	-,-	miRNA,pseu	328723;121281	intergenic	RNU6-184P,SURF6P1	-,+	snRNA,pseu	232079;624358
3708	chrX	88462263	+	chrY	6401459	+	N	N	Y	N	N	N	transl_inter	na	NA	intergenic;ir	RP13-324D24.1,SRIP	-,-	pseudogen	224909;160171	intergenic	TSPY17P,SRIP3	+,-	pseudogen	12489;185544
3709	chrX	88462263	+	chrY	6401518	+	N	Y	N	N	N	N	transl_inter	na	NA	intergenic;ir	RP13-324D24.1,SRIP	-,-	pseudogen	224909;160171	intergenic	TSPY17P,SRIP3	+,-	pseudogen	12548;185485
3710	chrX	88462480	+	chrY	6400797	+	N	Y	N	N	N	N	transl_inter	na	NA	intergenic	RP13-324D24.1,SRIP	-,-	pseudogen	225126;159954	intergenic	TSPY17P,SRIP3	+,-	pseudogen	11827;186206
3711	chrX	88462502	+	chrY	6402064	+	N	N	N	N	Y	N	transl_inter	na	NA	intergenic	RP13-324D24.1,SRIP	-,-	pseudogen	225148;159932	intergenic	TSPY17P,SRIP3	+,-	pseudogen	13094;184939
